# Electrochemically Generated Carbanions Enable Isomerizing Allylation and Allenylation of Aldehydes with Alkenes and Alkynes

**DOI:** 10.1021/jacs.3c04864

**Published:** 2023-06-15

**Authors:** Sheng Zhang, Yating Liang, Ke Liu, Xuan Zhan, Weigang Fan, Man-Bo Li, Michael Findlater

**Affiliations:** † Institutes of Physical Science and Information Technology, Key Laboratory of Structure and Functional Regulation of Hybrid Materials of Ministry of Education, Anhui University, Hefei, Anhui 230601, China; ‡ Department of Chemistry and Biochemistry, University of California Merced, Merced, California 95343, United States

## Abstract

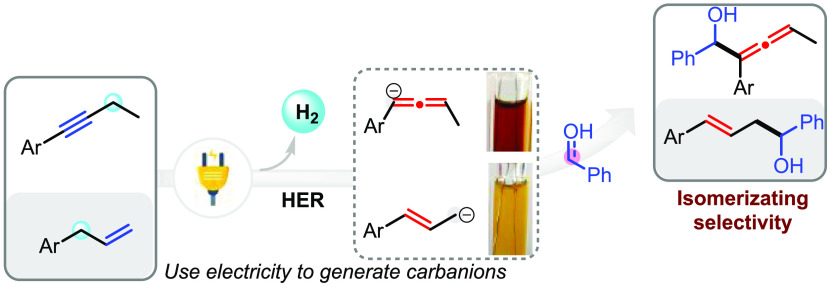

The direct coupling of aldehydes with petrochemical feedstock alkenes and alkynes would represent a practical and streamlined approach for allylation and allenylation chemistry. However, conventional approaches commonly require preactivated substrates or strong bases to generate allylic or propargylic carbanions and only afford branched allylation or propargylation products. Developing a mild and selective approach to access synthetically useful linear allylation and allenylation products is highly desirable, albeit with formidable challenges. We report a strategy using hydrogen evolution reaction (HER) to generate a carbanion from weakly acidic sp^3^ C–H bonds (p*K*_a_ ∼ 35–40) under mild reaction conditions, obviating the use of strong bases, Schlenk techniques, and multistep procedures. The cathodically generated carbanion *reverses* the typical reaction selectivity to afford unconventional isomerizing allylation and allenylation products (125 examples). The generation of carbanions was monitored and identified by *in situ* ultraviolet–visible (UV–vis) spectroelectrochemistry. Furthermore, we extended this protocol to the generation of other carbanions and their application in coupling reactions between alcohols with carbanions. The appealing features of this approach include mild reaction conditions, excellent functional group tolerance, unconventional chemo- and regioselectivity, and the diverse utility of products, which includes offering direct access to diene luminophores and bioactive scaffolds. We also performed cyclic voltammetry, control experiments, and density functional theory (DFT) calculations to rationalize the observed reaction selectivity and mechanism.

## Introduction

**Scheme 1:**
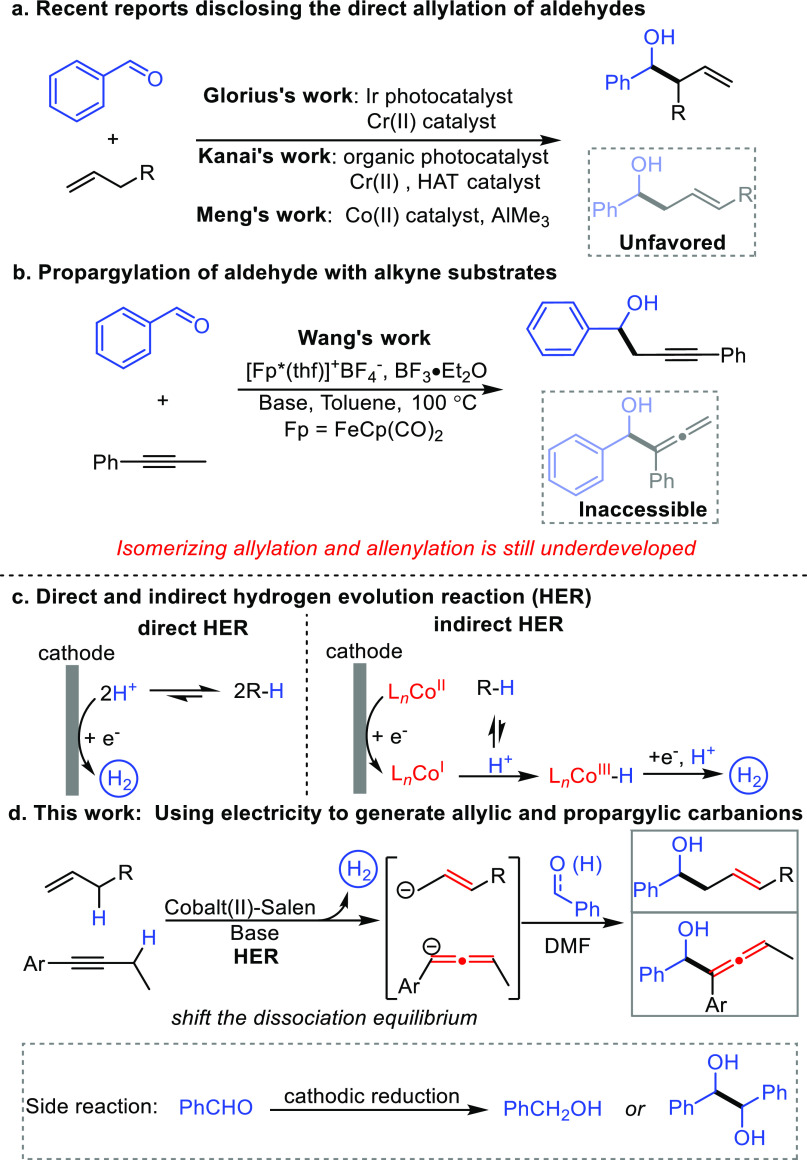
Strategies toward the Allylation and Allenylation of Aldehydes

The direct conversion of readily available bulk feedstock to fine chemicals has long been a fundamental goal of synthetic organic chemistry.^1^ Alkenes, alkynes, and aldehydes are some of the most attractive starting materials since they are widely prevalent in commercially available sources. Consequently, tremendous effort has been devoted to both the carbonyl allylation and allenylation reactions. Conventional approaches (e.g., Barbier-type reaction,^2^ Nozaki–Hiyama–Kishi reaction,^3^ Hosomi–Sakurai reaction,^4^ Roush asymmetric allylation^5^) commonly require preactivation of the substrate alkene or alkyne via installation of halogen,^6^ boron,^7^ silicon atoms,^8^ ester,^9^ or other^10^ groups. Growing concerns related to atom economy have shifted attention to the direct coupling of aldehydes with allylic and propargylic^11^ C–H bonds. For instance, a remarkable breakthrough in the direct allylation of aldehydes has been independently achieved by Glorius,^12^ Kanai,^13^ and Meng^14^ groups (Scheme 1a) by employing photoredox catalysis and cobalt catalysis strategies. In recent work, Wang^15^ and co-workers developed a direct propargylation of aldehydes using a combination of an iron catalyst and Lewis acid (Scheme 1b), in which the acidity of sp^3^ C–H (p*K*_a_ ∼ 35–40) was significantly enhanced via formation of an Fp(η^2^-alkyne)^+^ complex, with concomitant activation of the aldehyde by employing BF_3_. Despite these promising results, isomerization–allylation and allenylation reactions remain underdeveloped. Specifically, alkynes are attractive allenylation reagents^16^ as they would afford facile access to allenols, which are key intermediates^6,17^ in the synthesis of natural products and other bioactive molecules. As demonstrated in the work of Liu,^18^ the formidable challenges associated with isomerization selectivity and reactivity have severely restricted the development of such transformations.

The direct nucleophilic addition between aldehydes and allylic (or propargylic) carbanions could serve as an alternative approach for the allylation and allenylation reactions. However, this chemistry involves the use of highly sensitive bases (e.g., ^*n*^BuLi, ^*t*^BuLi, LDA), Schlenk techniques, and stepwise procedures and suffers from inferior regioselectivity. To address these issues, developing a mild and selective protocol to generate and exhibit “slow release” carbanions is in high demand. Synthetic electrochemistry could offer distinctive and efficient solutions to solve the types of “knotty” problems encountered in traditional transformations.^19^ The Baran group, among others, has been at the forefront of this charge and has demonstrated the advantages of electrochemistry over conventional approaches to the Birch reaction,^20^ Kolbe reaction,^21^ and alkene isomerization.^22^ Inspired by the work, we pondered if electrochemistry could provide a solution to generate a carbanion from allylic or propargylic substrates, and the slowly releasing carbanion might *reverse* the observed selectivity in the reaction with aldehydes.

Hydrogen evolution reaction (HER)^23^ is one of the most important reactions related to energy storage (Scheme 1c), which may proceed in the presence or absence of a catalyst. From the viewpoint of synthetic chemistry, the HER process could serve as an appealing strategy for the deprotonation of acidic substrates via a shift of the dissociation equilibrium. Although cathodic hydrogen evolution of alcohols has been extensively explored as a strategy toward electrochemically generated base (EGB),^24^ electrochemical deprotonation of poorly acidic C–H bonds (p*K*_a_ ∼ 35–40) remains elusive. Additionally, the cathodic reduction of aldehydes could interfere with the HER process. In this context, the key to developing an electrochemical allylation (or allenylation) is that the HER process should outcompete the cathodic reduction of aldehydes. With long-term research interests in synthetic electrochemistry and cobalt catalysis,^25^ we believed that a cobalt-salen^26^ catalyst and alkaline conditions might benefit the HER process of allylic and propargylic substrates thus facilitating the dissociation process (Scheme 1d). Indeed, the electrochemical protocol is amenable to generate allylic and propargylic carbanions, which were monitored and identified by *in situ* ultraviolet–visible (UV–vis) spectroelectrochemistry. More importantly, the slowly releasing carbanions rapidly isomerize and react with aldehydes to give homoallylic alcohols and allenols. This novel protocol was further extended to the generation of other carbanions and the direct allylation and allenylation of benzylic alcohols by virtue of convergent paired electrolysis. Collectively, a general and mild strategy using electricity to generate carbanions is disclosed for the isomerizing allylation and allenylation.

## Results and Discussion

### Feasibility Studies and Reaction Optimization

**Figure 1:**
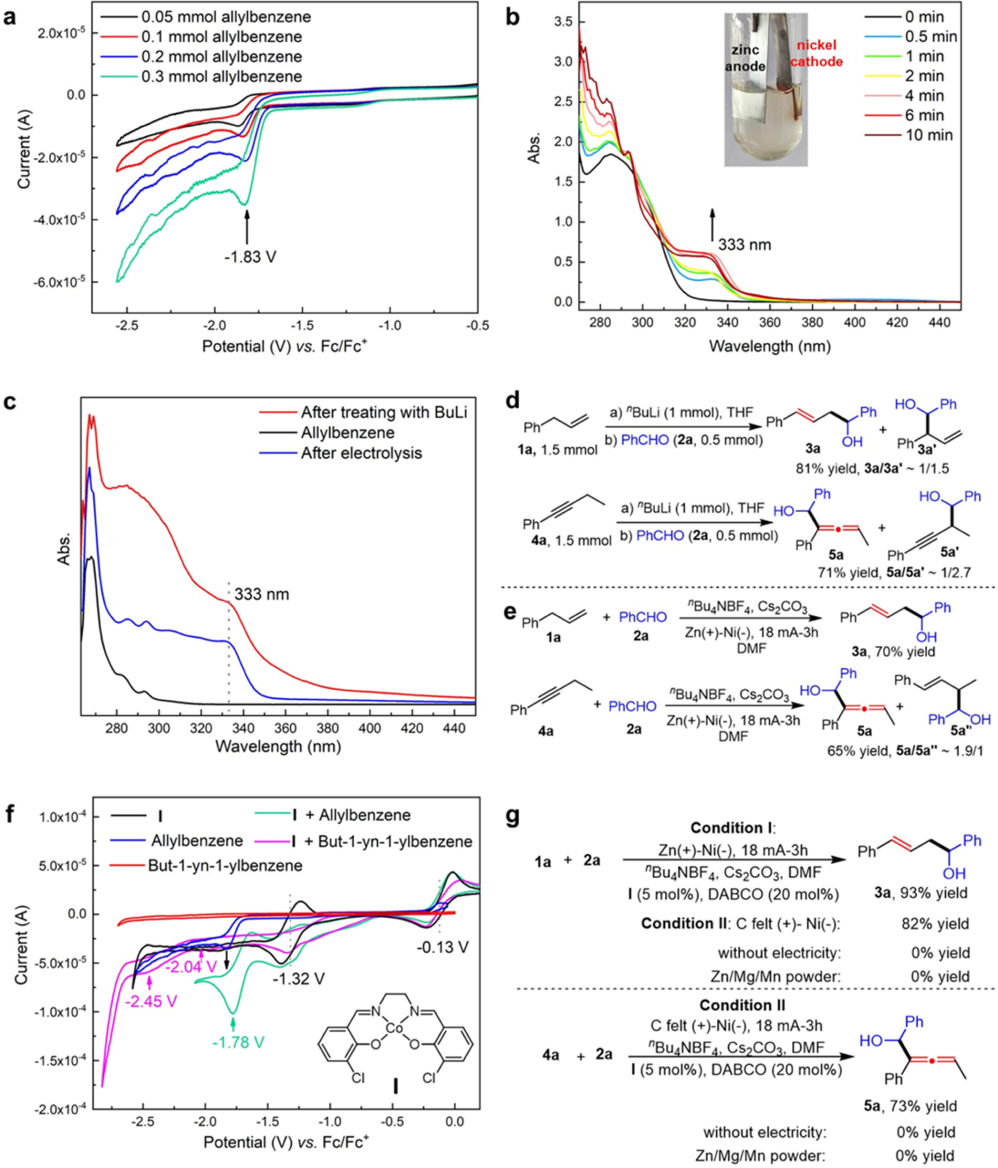
Feasibility studies and reaction optimization. (a) Cyclic voltammetry experiment of allylbenzene (0.05–0.3 mmol) in the presence of Cs_2_CO_3_ (0.05 mmol). (b) *In situ* UV–vis spectrum of electrolysis solution of allylbenzene. (c) Comparison of UV–vis spectra. (d) Testing the reactivity of carbanions using a conventional approach. (e) Preliminary study of electrochemical allylation and allenylation. (f) Cyclic voltammetry experiment to elucidate the role of cobalt (II)-salen. (g) Optimal conditions for electrochemical allylation and allenylation.

We began our study of electrochemical allylation by exploring the possibility of generating carbanions from allylbenzenes. First, the redox properties of allylbenzene were tested with cyclic voltammetry (CV) experiments. An inorganic base, Cs_2_CO_3_, was introduced to promote the HER process of allylbenzene (see Figure S11 for the CV experiment). As expected, allylbenzene showed a clear cathodic peak (−1.83 V) in the presence of Cs_2_CO_3_, and the peak is positively correlated with the concentration of allylbenzene (Figure 1a). This result suggests that the peak should arise from allylbenzene. Further CV study comparing the HER of AcOH (see Figure S13 for details) and detection of hydrogen product (see Figure S19 for details) verifies the HER process of allylbenzene. Direct electrolysis of allylbenzene in an alkaline solution of DMF gave a brown species over the surface of the nickel cathode (Figure 1b). Next, ultraviolet–visible (UV–vis) spectroelectrochemistry was conducted to identify the species electrochemically generated from allylbenzene (Figure 1b,c). As shown in the *in situ* spectra, a new peak at 333 nm was detected, and it increased with the electrolysis time (Figure 1b, also see Supplementary Video 1). We next compared the UV–vis spectra with that of allylic carbanion generated independently using ^*n*^BuLi, and it revealed that the cathodically generated species is likely the proposed allylic carbanion (Figure 1c). Propargylic carbanion arising from cathodic reduction of but-1-yn-1-ylbenzene was also identified by UV–vis spectroelectrochemistry (see Figure S4,S5, Supplementary Video 2 for details). Third, we tested the reactions between allylic (or propargylic) carbanion and benzaldehyde using *^n^*BuLi as a base (Figure 1d). It revealed the presence of the desired isomerizing allylation (**3a**) and allenylation (**5a**) products but alongside substantial amounts of side products (**3a′** and **5a′**). This observation confirmed that carbanions can serve as a reactive intermediate to afford allylation and allenylation products. Encouraged by these results, electrochemical allylation and allenylation were conducted in a solution of Cs_2_CO_3_ in DMF (Figure 1e). To our delight, the cathodically generated allylic carbanions reacted readily with benzaldehyde to exclusively afford an isomerizing homoallylic alcohol product **3a** in 70% yield. In the reaction of but-1-yn-1-ylbenzene, allenol **5a** and reductive product **5a″** were afforded with inferior selectivity (1.9/1).

To further improve the reaction efficiency and selectivity, an HER catalyst, Co^II^-salen (**I**), was introduced to the reaction system. As shown in Figure 1f, two reversible couples were detected for **I**, which is assigned to the process of Co^II^/Co^III^ (−0.13 V) and Co^II^/Co^I^ (−1.32 V). Upon treatment of **I** with allylbenzene, an obvious increase (from 36 to 101 μA) and shift (from −1.83 to −1.78 V) of the cathodic peak of allylbenzene was observed, indicating the catalytic role of cobalt catalyst in HER. A similar result was also detected in the case of but-1-yn-1-ylbenzene and revealed cathodic peaks at −2.04 and −2.45 V, which were weak or even undetectable in the absence of **I**. Finally, the merger of **I** and DABCO was introduced to the reaction system (Figure 1g), and excellent yield (93%) of product **3a** was observed using a sacrificial zinc anode (**condition I**). Additionally, the sacrificial anode could be replaced by the graphite felt anode (**condition II**, 82% yield) through the sacrificial oxidation of an excess substrate. By obviating the generation of zinc anions, allenol product **5a** was generated selectively in 73% yield. Control experiments carried out in the absence of electricity or with reductants (zinc, magnesium, manganese powder) completely shut down the reaction, even with the prolonged reaction time (24 h). This result highlights both the novelty and power of this electrochemical protocol.

### Exploration of Scope

**Scheme 2:**
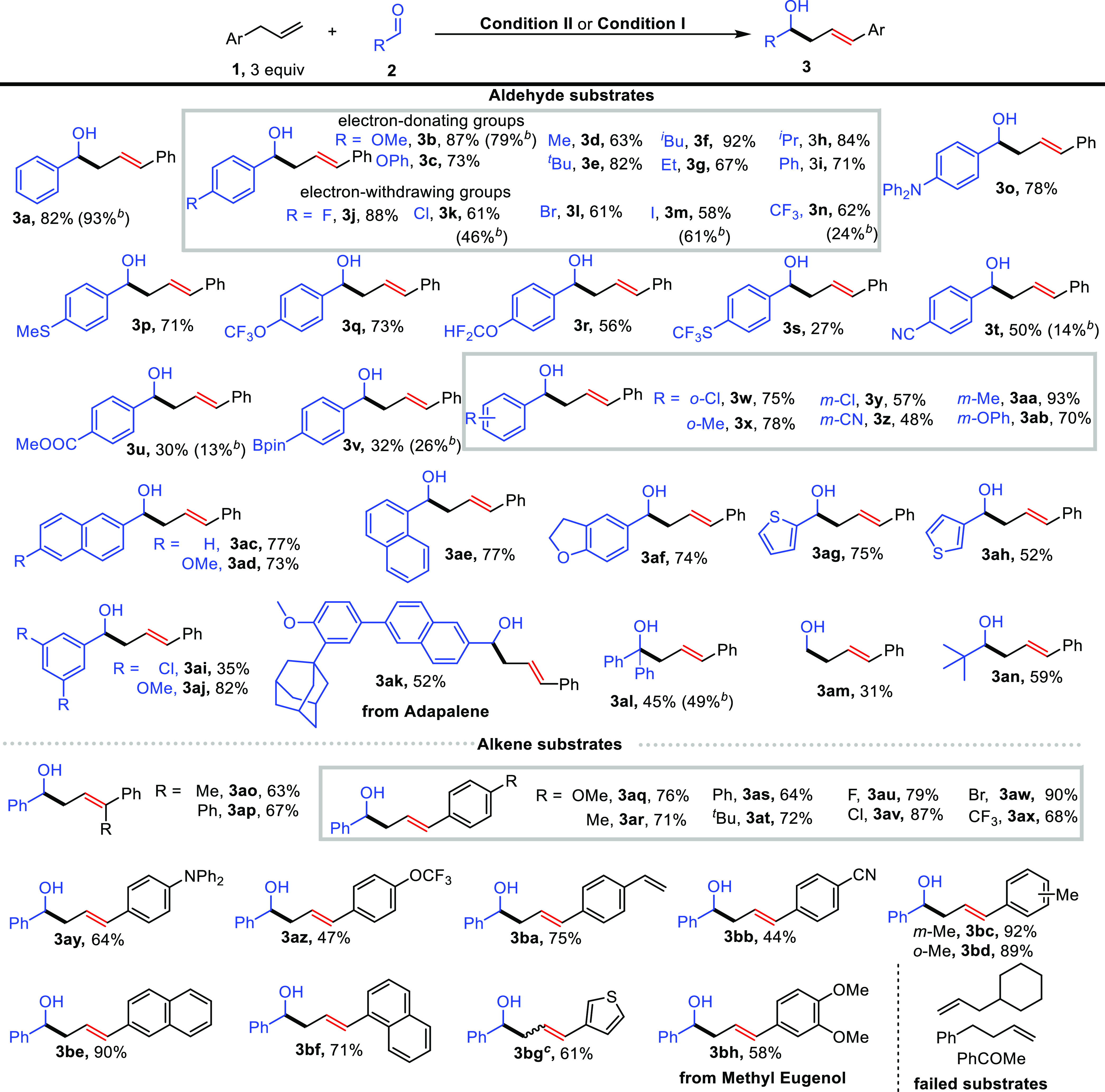
Substrate Scope of the Isomerizing Allylation of Aldehydes,, **Condition II**: aldehyde (0.5 mmol), allylbenzene (1.5 mmol), ^*n*^Bu_4_NBF_4_ (1 mmol), Cs_2_CO_3_ (1 mmol), DABCO (0.1 mmol), **I** (0.025 mmol), graphite felt anode, nickel plate cathode, CCE (18 mA, 3h), 10 °C; isolated yield is given; *l*/*b* > 20/1, *E*/*Z* > 20/1. **Condition I**: Zinc plate anode. *E*/*Z* = 2/1.

With the optimized reaction conditions in hand (see Tables S1–S2 for details of optimization), the scope of the substrate amenable to the isomerizing allylation of aldehydes was evaluated (Scheme 2). A broad range of aldehydes were first examined using allylbenzene (**1a**) as the alkene substrate, and uniformly excellent regioselectivity (*l*/*b* > 20/1) and stereoselectivity (*E*/*Z* > 20/1) were observed by employing reaction **condition II**. In contrast, the use of **condition I** displayed inferior functional group tolerance (**3b, 3k, 3n, 3t–3v**) presumably arising from the anodically generated zinc anions coordinating to the aldehyde and thus facilitating an undesired reduction reaction. An investigation of substrate electronic effects revealed that electron-rich substrates (**3b-3i**) are more efficient than electron-deficient ones (**3j-3n**). This result can be understood by the cathodic reduction of electron-deficient aldehydes that would interfere with the hydrogen evolution reaction of allylbenzene due to their relatively positive cathodic potential. Notably, some synthetically useful but challenging substituents (amine, thioether, fluorinated group, nitrile, ester, borate) were well tolerated and afforded linear allylation products (**3p**–**3v**) in acceptable yields. Changing the position of the substituent had no significant effect on the reaction performance, and the allylation products (**3w–3ab**) were observed in similar yields. Additionally, aldehydes bearing fused rings (**3ac–3af**), heterocycles (**3ag–3ah**), and multiple substituents (**3ai–3aj**) were readily allylated and furnished homoallylic alcohols **3ac–3aj**. To demonstrate the potential synthetic utility of our approach, an adapalene-derived aldehyde was subjected to the standard conditions. Gratifyingly, the desired product (**3ak**) was obtained in synthetically useful yield (52%). The reaction scope was further extended to less reactive benzophenone (**3al**) and aliphatic aldehydes (**3am–3an**) albeit with decreased yields. The generality of an electrochemical allylation was further demonstrated by employing several allylbenzene substrates. A similar functional group tolerance was observed, and the corresponding products (**3ao–3bh**) were obtained with moderate to good yields. Remarkably, halogen atoms (**3au–3aw**) and nitrile (**3bb**) and thiophene (**3bg**)-substituted substrates proceeded smoothly in the reaction to give desired products that are incompatible with conventional ^*n*^BuLi conditions. α-Substituted alkenes also proved to be viable substrates to smoothly deliver trisubstituted alkene products (**3ao–3ap**) with high stereocontrol. Additionally, excellent chemoselectivity was demonstrated in a substrate bearing multiple double bonds delivering a mono-allylation product (**3ba**). Unfortunately, both allylcyclohexane and 4-phenyl-1-butene failed to serve as allylation reagents presumably due to the instability of the corresponding carbanions. Acetophenone, which bears a more acidic α C–H bond than allylbenzene, failed to give the desired allylation product.

**Scheme 3:**
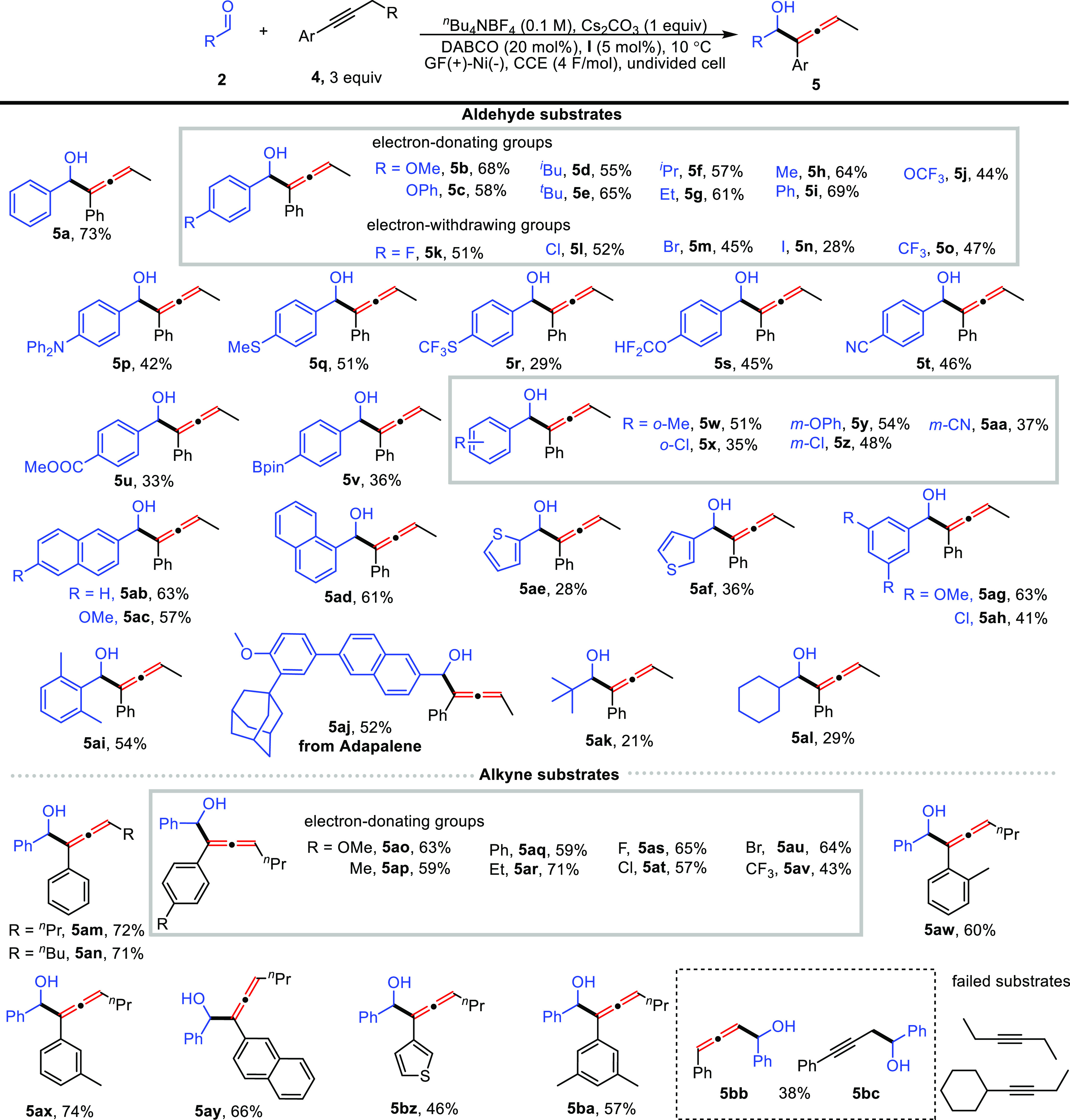
Substrate Scope of the Allenylation of Aldehydes **Condition II**: aldehyde (0.5 mmol), alkyne (1.5 mmol), ^*n*^Bu_4_NBF_4_ (1 mmol), Cs_2_CO_3_ (1 mmol), DABCO (0.1 mmol), **I** (0.025 mmol), graphite felt anode, nickel plate cathode, CCE (18 mA, 3h), 10 °C; isolated yield is given; *dr* ∼ 1/1.

Having established electrochemical isomerizing allylation of aldehydes, we next turned attention to the allenylation of aldehydes (see Table S3 for details of optimization). Several aldehydes were shown to be excellent coupling partners in the reaction with but-1-yn-1-ylbenzene (Scheme 3). Consistent with the results of allylation, aldehydes bearing electron-donating groups (**5b–5j**) delivered better yields than the aldehydes bearing electron-withdrawing groups (**5k–5o**). This excellent functional group tolerance was further highlighted in the cases of **5p–5v** since these substituents are sensitive to conventional conditions, such as strong bases, oxidants, or reductants. To our delight, *ortho-* (**5w–5x**)*, meta*-substitution (**5y–5aa**), other aromatic rings (**5ab–5af**), and multiple substituents (**5ag–5ai**) on substrates were all well tolerated. Specifically, sterically hindered 2,6-dimethylbenzaldehyde could efficiently couple with 1-phenyl-1-butyne, giving product **5ai** in moderate yield. Late stage derivatization of adapalene affords the corresponding allenyl alcohol product **5aj** with reasonable yield. Aliphatic aldehydes (**5ak–5al**) showed lower reactivity in this transformation, presumably due to the presence of acidic α C–H bonds (**5al**) that would compete with the HER of the alkyne. The substrate scope of the alkyne coupling partner (**5am–5bc**) was also examined. The reaction performance is uniformly maintained regardless of the electronic nature or substitution patterns, whereas aliphatic alkynes do not react under optimal conditions. Control experiments with phenylpropyne as an allenylation reagent resulted in a mixture of allenol (**5bb**) and propargyl alcohol (**5bc**), which could be rationalized by the superior nucleophilicity of a primary propargylic carbanion compared to the allenylic resonance. The unusual site selectivity obtained with allenol **5bb** presumably arose from the isomerization of **5bc** via the deprotonation of HER.

### Versatility and Extension of the Electrochemical Protocol

**Scheme 4:**
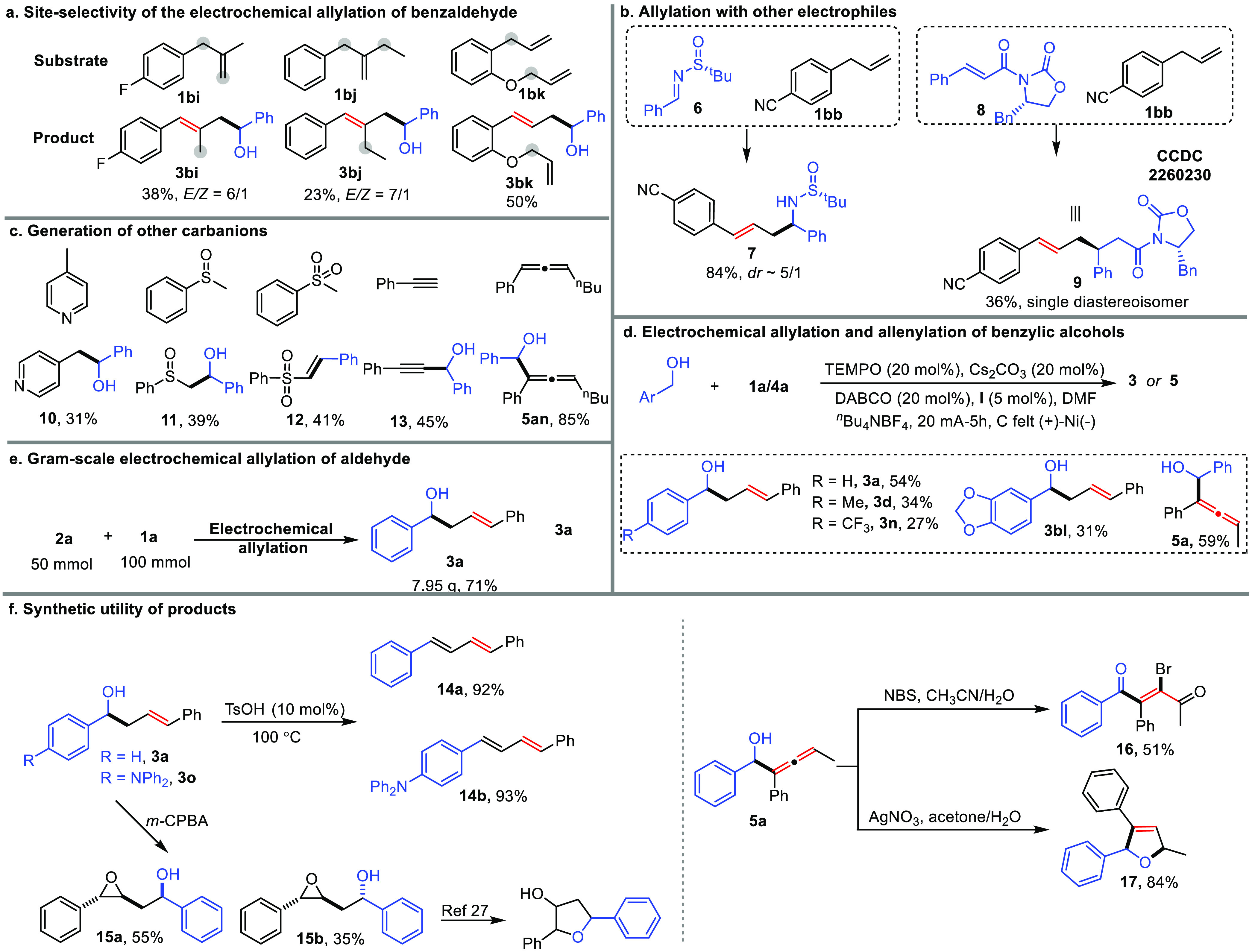
Superiority, Synthetic Utility, and Extension of the Electrochemical Protocol

To demonstrate the versatile and complementary nature of the electrochemical protocol compared with conventional approaches, substrates **1bi–1bk** bearing multiple potentially reactive sites were subjected to the optimal conditions (Scheme 4a). Gratifyingly, corresponding products **3bi–3bk** arising from single site reactivity were produced exclusively and in good stereo- and site selectivity. Next, we extended the electrochemical protocol to other transformations (Scheme 4b–d). For example, imine (**6**) and activated alkene (**8**) substrates containing chiral auxiliaries were used as electrophiles in reaction with allylic anions (Scheme 4b). As anticipated, the desired allylation products **7** and **9** were readily generated under high stereocontrol, and the absolute configuration of **9** could be assigned by single-crystal X-ray diffraction studies (CCDC 2260230). Other substrates with acidic C–H bonds, including 4-methylpyridine, (methylsulfinyl)benzene, (methylsulfonyl)-benzene, phenylacetylene, and allene, were also examined as precursors of carbanions in the electrochemical protocol (Scheme 4c), and the aldol-type products (**10–13, 5an**) were uniformly accessed in acceptable yields under ambient atmosphere. Conventional approaches toward these products involve LDA and *^n^*BuLi. Furthermore, we proposed a transformation, which would integrate the cathodic generation of carbanions with anodic oxidation of benzylic alcohols (Scheme 4d). Under convergent paired electrolysis, the direct allylation and allenylation of alcohols were achieved, albeit with lower yields and prolonged electrolysis time. To the best of our knowledge, this is the first report of the direct allylation (or allenylation) of alcohols.

### Applications in Synthesis: Multigram-Scale Chemistry and Beyond

The synthetic utility of the electrochemical allylation and allenylation reactions was further demonstrated by applying them in both gram-scale reactions and in the application of the derivatization of products (Scheme 4e,f). With simple modification of reaction conditions, the electrochemical isomerizing allylation was readily scaled up to afford the corresponding allylation product and exhibited only slightly diminished yields (Scheme 4e). Elaboration of the linear homoallylic alcohols (**3a, 3o**) was performed with facile acidic dehydration to deliver corresponding diene products (**14a, 14b**), which were accessed in excellent yields and *E*/*Z* selectivity (Scheme 4f). To our delight, these conjugated dienes (**14a**, **14b**) showed excellent photoluminescence properties (Figures S40–S43) in the solid state with emission peaks, which fall in the range of blue light (422, 474 nm) and exhibit high quantum yield (61.2, 54.5%). These promising photophysical properties made them potential candidates as blue OLED materials. Additionally, epoxidation of **3a** was readily achieved with 3-chloroperbenzoic acid (*m-*CPBA) as an oxidant to afford isomers **15a** and **15b**, which could be further transformed into a bioactive tetrahydrofuran scaffold^27^ (Scheme 4f). Allenols are a versatile building block and have been extensively explored in previous work.^6,17^ The synthetic utility of the obtained products was further demonstrated by employing them in a series of transformations (Scheme 4f). In the presence of *N-*bromosuccinimide (NBS), allenol **5a** was readily converted to an interesting 1,4-dione product **16**, which would be completely inaccessible using prior approaches. Moreover, silver (I)-promoted cyclization of **5a** affords direct access to 2,5-dihydrofuran, **17**.

### Examination of Mechanism

**Figure 2:**
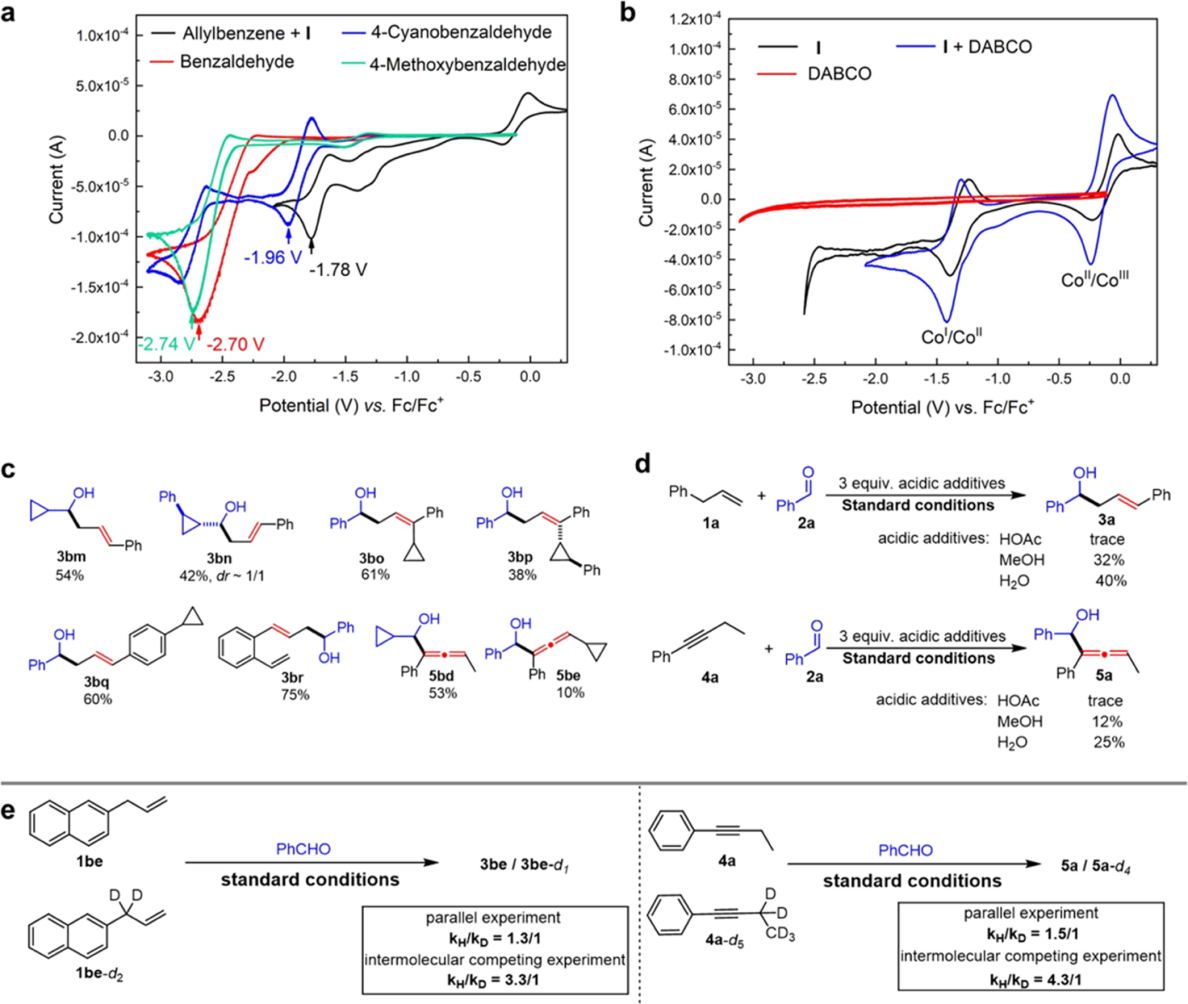
Mechanistic studies. (a) Cyclic voltammetry of substrates. (b) Cyclic voltammetry of **I** and DABCO. (c) Radical clock experiments for electrochemical allylation and allenylation. (d) Effect of acidic additives. (e) Kinetic isotope effect (KIE) study.

To gain insight into the electrochemical behavior of substrates and the additive DABCO, a series of CV experiments were carried out (Figure 2a,b). First, we compared the reduction potential of allylbenzene with aldehyde substrates (Figure 2a). It clearly showed that allylbenzene (*E*_red_ = −1.78 V) is more susceptible to cathodic reduction compared to aldehydes (*E*_red_ = −1.96–2.74 V). A cathodic potential monitoring experiment also showed that the reaction cathodic potential is more positive than that of aldehyde (−2.7 V), only enabling the reduction of allylbenzene (see Figures S35,S36 for details). This result indicates that the electrochemical allylation should be initiated by the HER of the excess allylbenzene rather than the reduction of aldehyde. Second, we explored the role of DABCO with the CV spectra including Co^II^-salen (**I**) (Figure 2b). It revealed that the cathodically inert DABCO could facilitate the redox cycle of Co^II^/Co^III^ and Co^I^/Co^II^ with a significant increase of the peaks. The control experiment removing the DABCO additive also suggests its important role in the reaction (Tables S1–S3). Third, the oxidation of excess allylbenzene is proposed to be the anodic reaction under **condition II** based upon the CV and anode potential monitoring experiments (see Figures S37, S38 for details).

To further probe the reaction mechanism, a series of control experiments were subsequently carried out (Figure 2c–e). We exclude the possibility of a radical mechanism using radical clock experiments by employing a broad range of substrates (Figure 2c). Both aldehydes and carbanion precursors (allylbenzene and alkyne) bearing cyclopropyl groups were subjected to the standard conditions, and the corresponding products (**3bm-3br, 5bd-5be**) were obtained without detection of radical-initiated ring-opening or cyclization (in the case **3br**) products. From these observations, we conclude that the electrochemical allylation and allenylation proceed via an ionic pathway. The pathway involving carbanions was further supported by studying the effects of acidic additives on the reaction. The addition of excess acid, such as acetic acid, methanol, and water, substantially decreased the reaction efficiency, due to the undesired HER of the acidic additives. Finally, we investigated kinetic isotope effects (KIEs) in the electrochemical protocol with parallel and intermolecular competition experiments. Interestingly, the parallel experiment revealed the presence of a secondary KIE (*k*_H_/*k*_D_ = 1.3/1–1.5/1) in the reaction, further supporting the proton transfer in the HER process.^28^ In contrast, the intermolecular competition experiment gives a significantly higher value of *k*_H_/*k*_D_ (3.3/1–4.3/1). The discrepancy in the KIE results can be rationalized by considering the rapid hydrogen/deuterium (H/D) exchange of the carbanions generated in the reaction.

**Figure 3:**
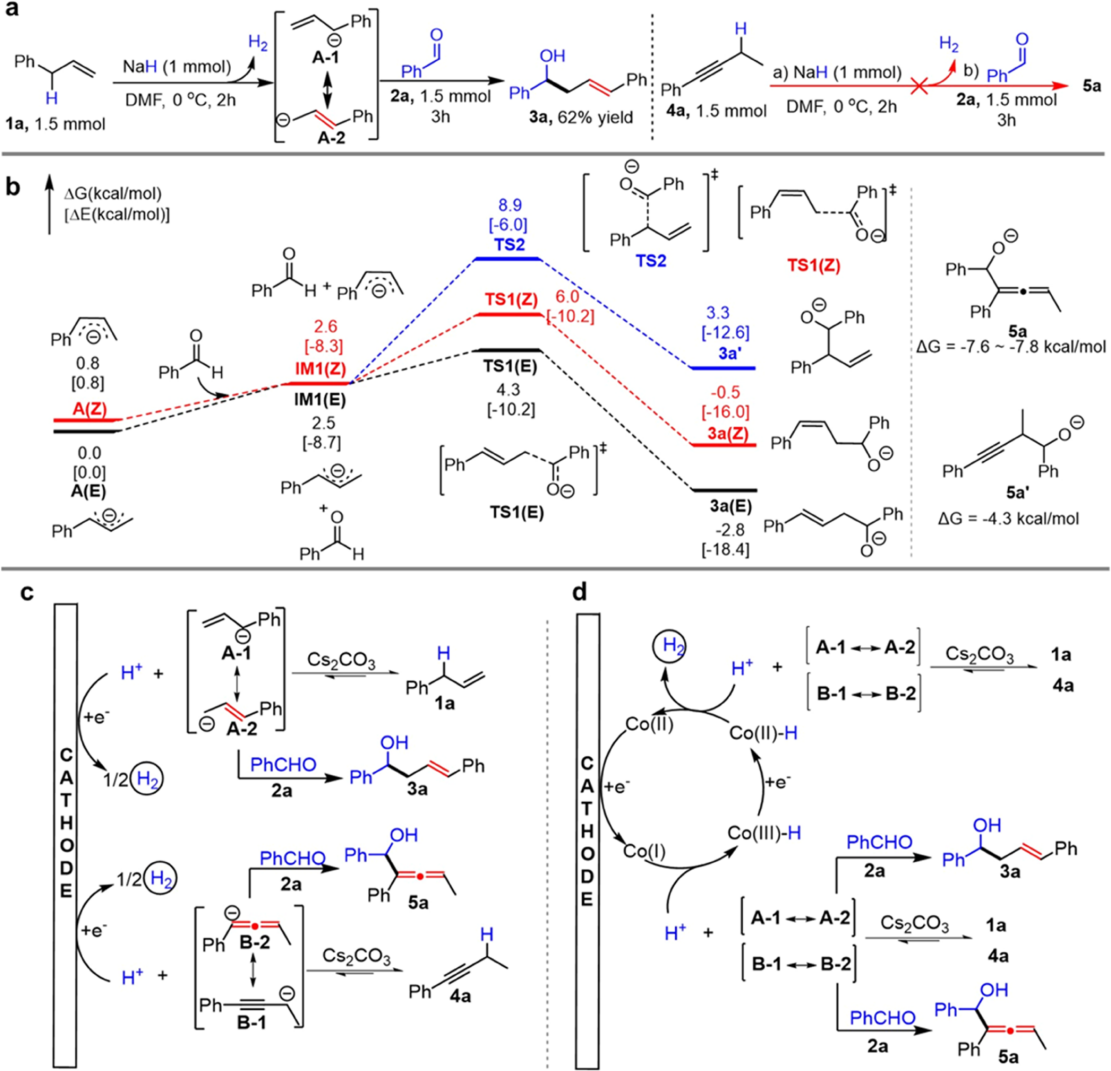
Investigation of the reaction selectivity and proposed reaction mechanism. (a) Using NaH to mimic the HER process. (b) DFT calculation of free energy at the PBE0 + D3(BJ)/ma-def2-TZVPP (SMD, solvent = DMF)//B3LYP + D3(BJ)/Def2-SVP level. (c) Mechanism involving direct HER. (d) Mechanism involving indirect HER.

To help understand the unconventional selectivity in the electrochemical protocol, control experiments and DFT calculations were conducted. We first used NaH to mimic the HER process in a polar aprotic solvent (DMF) (Figure 3a). The isomerizing allylation product **3a** was obtained exclusively in 62% yield, although NaH failed to initiate the reaction of **4a** due to its lower acidity. This result clearly suggests that the allylic carbanion (**A-2**) generated in DMF would react with **2a** to afford the isomerizing product **3a.** This conclusion is also supported by the computational work of Brinck,^29^ in which an allylic carbanion was found to have a larger solvation energy in DMF than in THF. We next sought to rationalize the reaction selectivity using DFT calculations. As shown in Figure 3b, **TS1(E)** is kinetically favored over **TS1(Z)** and **TS2** with lower activation energy (4.3 *vs* 6.0, 8.9 kcal/mol). The desired product **3a(E)** is also controlled by thermodynamics, as the Gibbs free energy change Δ*G* in the reaction is −2.8 kcal/mol, much lower than that of the other two products. The branched product **3a′** proved to be inaccessible due to a positive Δ*G* (3.3 kcal/mol). These results are consistent with the experimental observations. For the electrochemical allenylation, computational results (see Figure S45) support that the reaction selectivity arises from thermodynamical control, and a lower Δ*G* (−7.6 *vs* −4.3 kcal/mol) was calculated in the reaction producing **5a** compared to **5a′**.

Based on our combined experimental observations and DFT calculations, two plausible reaction mechanisms involving direct HER or indirect HER mechanism^23^ are proposed for the electrochemical allylation and allenylation. In the direct HER mechanism (Figure 3c), the proton dissociated from the weakly acidic substrate **1a** or **4a** is directly reduced to hydrogen, and the equilibrium shifts to the right. Corresponding carbanions **A-2** and **B-2** are slowly released and react with benzaldehyde giving products **3a** and **5a**, respectively. In the Co^II^-salen-mediated HER mechanism (Figure 3d), the dissociation of **1a** and **4a** is promoted by the cathodically generated Co^I^ species giving a Co^III^-H intermediate. Further single-electron transfer (SET) reduction of Co^III^-H gives a hydride species (Co^II^-H), which enables an alternative deprotonation approach for an acidic substrate **1a** or **4a.** The *in situ* generated carbanions **A-2** and **B-2** proceed via a similar nucleophilic addition with aldehydes to afford the final products **3a** and **5a**.

## Conclusions

In conclusion, a strategy using hydrogen evolution reaction (HER) to generate a carbanion from allylic and propargylic C(sp^3^)–H bonds has been developed and is revealed to *reverse* the chemoselectivity of allylation and allenylation of aldehydes. With this strategy, unactivated alkenes and alkynes readily couple with aldehydes to afford linear allylation and allenylation products, which are challenging to access using previous approaches. Moreover, we extended this protocol to the generation of other carbanions under ambient conditions and the coupling reaction between alcohols with carbanions. We believe that this strategy can be adapted to facilitate access to a broad range of carbanions to deliver previously challenging or even inaccessible product classes.

## Supporting Information Available

Experimental procedures; characterization of the synthesized compounds; and NMR spectra; SI part I_reaction procedures (PDF)
SI part II_spectra (PDF)
Supplementary video 1_Carbanion generating from allylbenzene (MP4)
Supplementary video 2_Carbanion generating from 1-Phenyl-1-butyne (MP4)
