# Supplementary material for: Electrochemically Generated Carbanions Enable Isomerizing Allylation and Allenylation of Aldehydes with Alkenes and Alkynes
Source: J Am Chem Soc. 2023 Jun 15;145(25):14143–54. doi: 10.1021/jacs.3c04864 (PMC10311535; doi:10.1021/jacs.3c04864)
Supplement: Supplementary file 1 — ja3c04864_si_001.pdf [file ja3c04864_si_001.pdf]

Electrochemically Generated Carbanions Enable Isomerizing Allylation and Allenylation of Aldehydes with Alkene and Alkynes

Sheng Zhang[1]*, Yating Liang[1], Ke Liu[1], Xuan Zhan[1], Weigang Fan[1], Man-Bo Li[1]* and Michael Findlater[2]*

[1] Institute of Physical Science and Information Technology, Key Laboratory of Structure and Functional Regulation of Hybrid Materials of Ministry of Education, Anhui University, Hefei, Anhui 230601, P. R. China. [2]Department of Chemistry and Biochemistry, University of California Merced, Merced CA 95343, USA.

Email: shengzhang@ahu.edu.cn; mbli@ahu.edu.cn; michaelfindlater@ucmerced.edu

Supporting Information

Table of Contents

Part I Experimental Section S2

1. General information S2

2. Ultraviolet-visible (UV–vis) spectroelectrochemistry monitoring S2-S4

3. Cyclic voltammetric experiments S5-S11

4. GC analysis of reaction atmosphere S12

5. Optimization of reaction conditions S13-S17

6. General procedure for the electrochemical allylation and allenylation S18-S19

7. Procedure for gram scale reaction and derivatization of products S20-S21

8. Procedure and details for control experiments S22-S35

9. Photophysical properties of **14a** and **14b** S36-S37

10. Details of DFT calculation S38-S62

11. Experimental data S63-S104

12. References S105

1. General Information

^{1}H NMR and ^{13}C NMR were recorded on a Bruker 400 MHz and 600 MHz spectrometer (^{1}H NMR: 400MHz, ^{13}C NMR: 100MHz, 150MHz). The chemical shifts (δ) and coupling constants (J) were expressed in ppm and Hz respectively. ^{1}H NMR spectra were referenced to the solvent residual peak (TMS, δ 0 ppm) and ^{13}C{1H} NMR spectra were referenced to the solvent residual peak ($CDCl_3$, δ 77.0 ppm). Ultraviolet-visible (UV–vis) spectra were recorded with a SPECORD 210 PLUS spectrophotometer. High Resolution mass spectra were obtained using AB SCIEX X500R TOF mass spectrometer. GC analysis was conducted using CEAULIGHT GC-7920 spectrometer. All solvents were purified and dried according to the standard procedures unless otherwise noted. Commercially substrates were purchased and used directly. α-Substituted allylbenzenes (but-3-en-2-ylbenzene, prop-2-ene-1,1-diyldibenzene, 1-cyclopropylallyl)benzene)[1], **2bj**,[1] **10**,[1] non-substituted allylbenzenes[2], **2bf-*d*$_2$**[2], Adapalene derived aldehyde[3], 1-phenyl-1-hexyne derivatives[4], phenylheptyne[4], **4a-*d*$_5$**[5], **4be**[5], **11**[6], salen-cobalt (**II, VII, VIII, IX, X**)[7], **V**[8] were prepared according to the literature procedures.

2. Ultraviolet-visible (UV–vis) spectroelectrochemistry monitoring

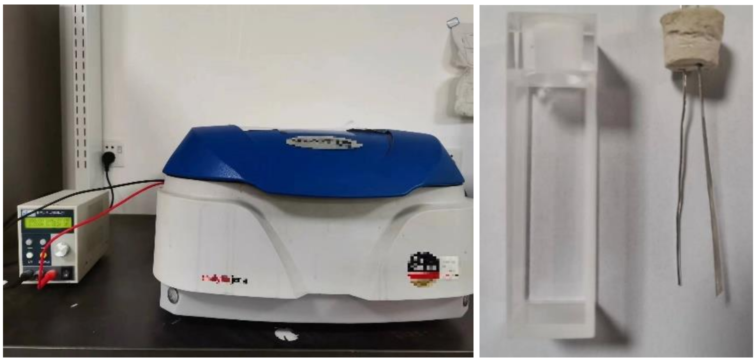

Figure S1. Setup of UV-vis spectroelectrochemistry (zinc wire anode, nickel wire cathode)

We conducted the cathodic electrolysis of allylbenzene (0.5 µL, 3.8*10^{-3} mmol) in the solution of DMF (3 mL) containing $^{n}Bu_4NBF_4$ (0.1 M) and Cs_2CO_3 (0.01 M) under constant current (3 mA). The UV-vis spectra were obtained during electrolysis process (Figure S2).

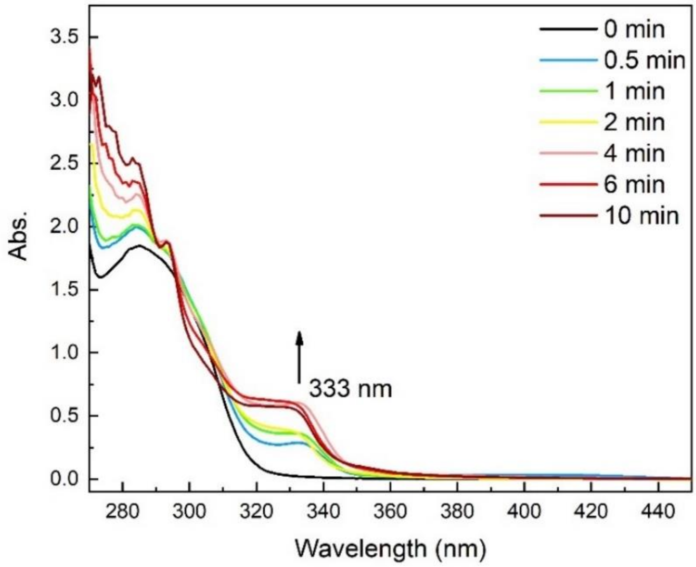

Figure S2. *In-situ* UV–vis spectrum of electrolysis solution of allylbenzene

To identify the species generating in the reaction, the reaction solution of allylbenzene treating with ^{n}BuLi (in THF) was recorded with UV-vis spectrophotometer. The comparison (Figure S3) between the electrolysis solution and the above reaction solution suggests that the intermediate generating over cathode should be allylic carbanion.

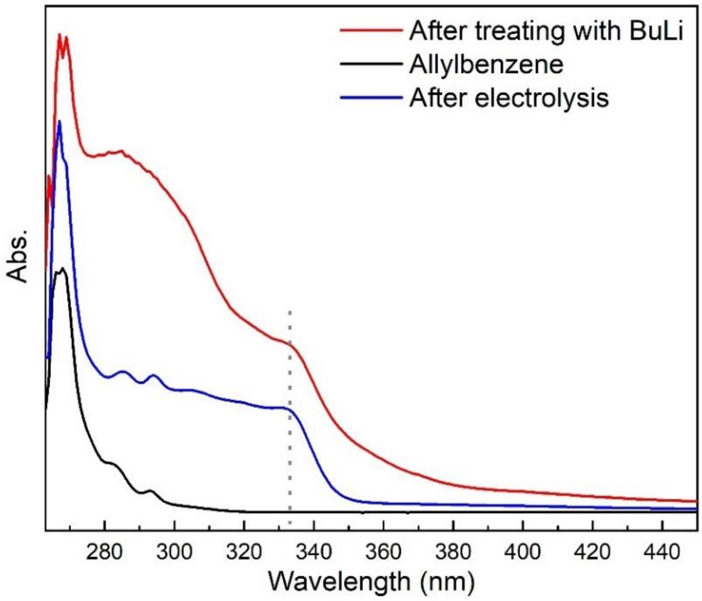

Figure S3. Comparison of UV–vis spectra

We conducted the cathodic electrolysis of but-1-yn-1-ylbenzene (0.5 μL, 3.5*10^{-3} mmol) in the solution of DMF (3 mL) containing $^{n}Bu_4NBF_4$ (0.1 M) and Cs_2CO_3 (0.01 M) under constant current (3 mA). The UV-vis spectra were obtained during electrolysis process (Figure S4). As shown the spectra, a broad peak ranging from 300-400 nm was detected.

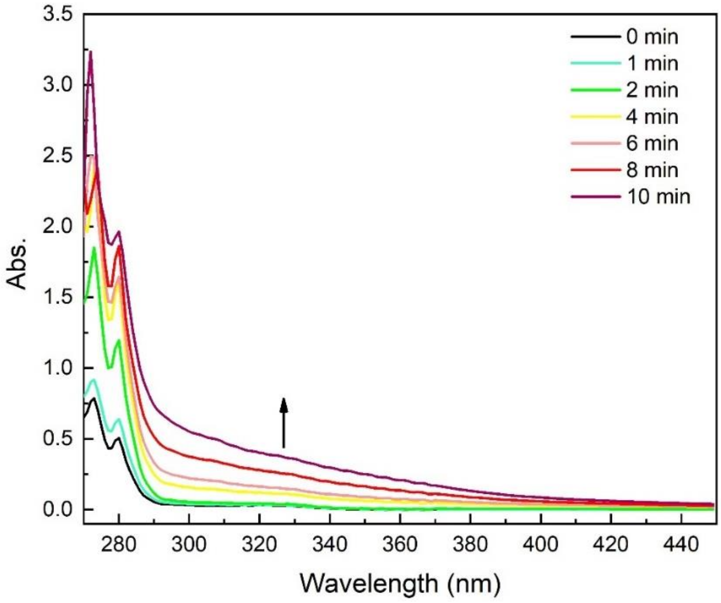

Figure S4. *In-situ* UV–vis spectrum of electrolysis solution of but-1-yn-1-ylbenzene

To identify the species generating in the above reaction, the reaction solution of but-1-yn-1-ylbenzene treating with ^{n}BuLi (in THF) was recorded with UV-vis spectrophotometer. The comparison (Figure S5) between the electrolysis solution and the above reaction solution

suggests that the intermediate generating over cathode should be propargylic carbanion.

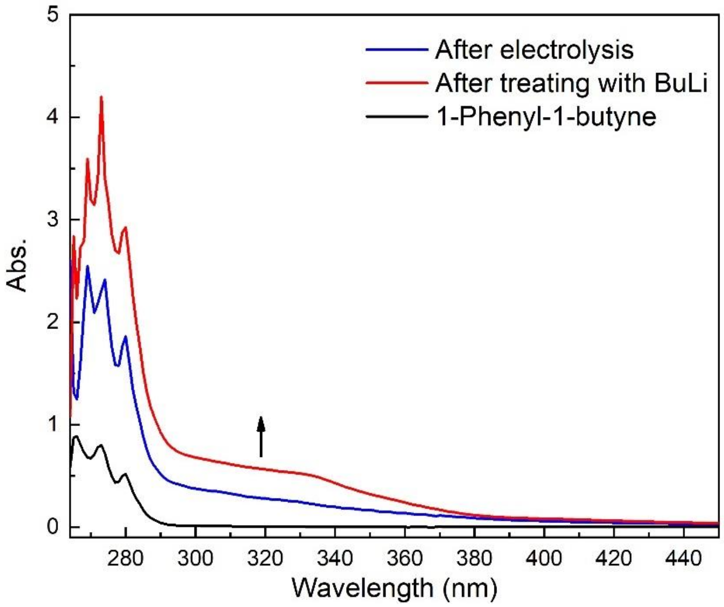

Figure S5. Comparison of UV–vis spectra

3. Cyclic voltammetric experiments

The electrochemical analysis was demonstrated with Ag wire as a reference electrode, which is not a stable reference electrode. CVs can be calibrated using ferrocene as an external reference. (Figure S6) $E_0(Fc/Fc^+)$ = (0.145+0.028)/2 = 0.865V.

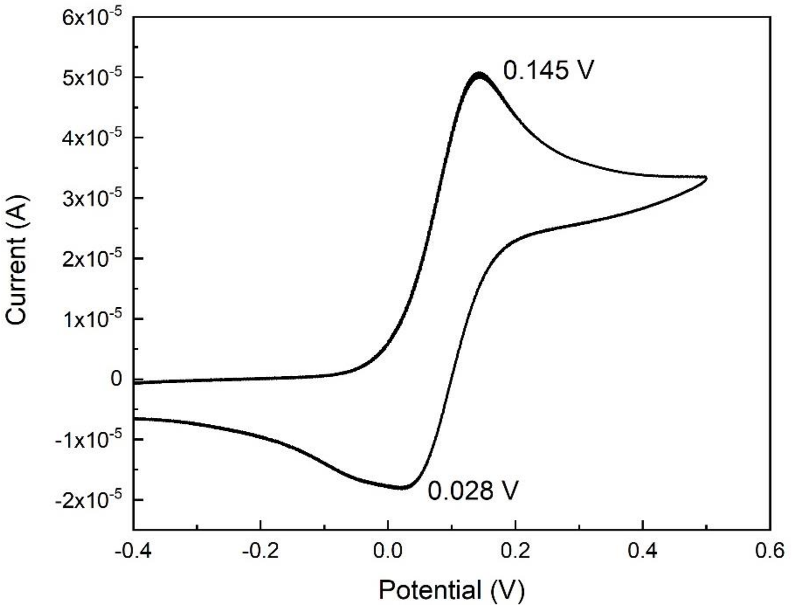

Figure S6 Cyclic voltammograms of ferrocene (0.02 M) in 0.1 M $^nBu_4NBF_4$ (DMF), using a glassy carbon working electrode and Pt wire, Ag/$AgNO_3$ (0.1 M in CH_3CN) as counter and reference electrodes at a 100 mV/s scan rate.

According to the UV–vis pectroelectrochemistry study, allylbenzene can be reduced to afford carbanions. The reductive potential of the allylbenezen was investigated (Figure S7), and a obvious cathodic peak was detected at -1.83V (*vs* Fc/Fc+).

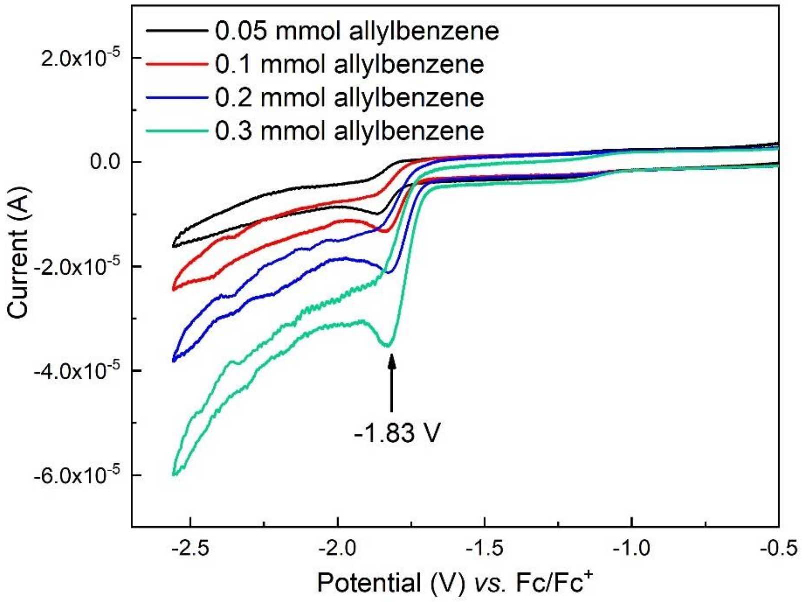

Figure S7 Cyclic voltammograms of allylbenezene (0.05 mmol-0.3 mmol) in 0.1 M $^nBu_4NBF_4$ (DMF 3 mL) containing Cs_2CO_3 (0.05 mmol), using a glassy carbon working electrode and Pt wire, Ag/$AgNO_3$ (0.1 M in CH_3CN) as counter and reference electrodes at a 100 mV/s scan rate.

The reductive potential of the 1-allyl-4-methoxybenzene was also investigated (Figure S8), and a obvious cathodic peak was detected at -1.97 V (*vs* Fc/Fc+).

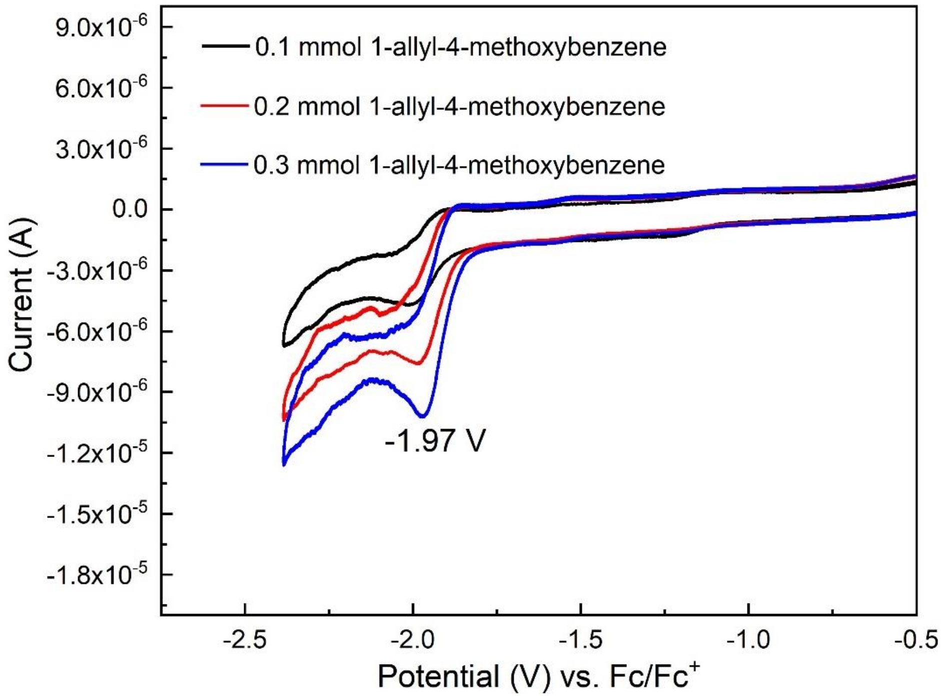

Figure S8 Cyclic voltammograms of 1-allyl-4-methoxybenzene (0.1 mmol-0.3 mmol) in 0.1 M $^{n}Bu_4NBF_4$ (DMF 3 mL) containing Cs_2CO_3 (0.05 mmol), using a glassy carbon working electrode and Pt wire, $Ag/AgNO_3$ (0.1 M in CH_3CN) as counter and reference electrodes at a 100 mV/s scan rate.

The reductive potential of the 4-allylbenzonitrile was also investigated (Figure S9), and a obvious cathodic peak was detected at -2.28 V (*vs* Fc/Fc+).

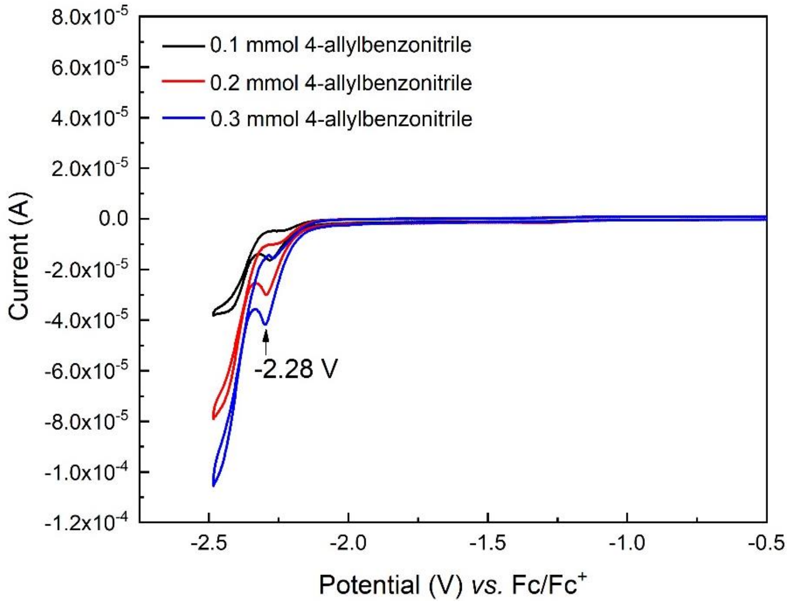

Figure S9 Cyclic voltammograms of 4-allylbenzonitrile (0.1 mmol-0.3 mmol) in 0.1 M $^{n}Bu_4NBF_4$ (DMF 3 mL) containing Cs_2CO_3 (0.05 mmol), using a glassy carbon working electrode and Pt wire, $Ag/AgNO_3$ (0.1 M in CH_3CN) as counter and reference electrodes at a 100 mV/s scan rate.

The reductive potential of the but-1-yn-1-ylbenzene was also investigated (Figure S9), and a slope around -2.10 V (*vs* Fc/Fc+) was detected. This weak slope might attribute to the weak acidicity of but-1-yn-1-ylbenzene.

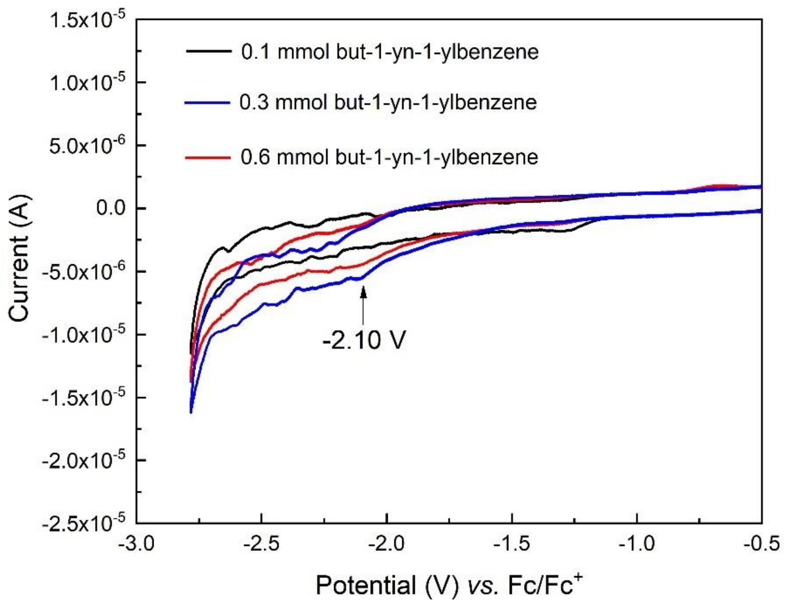

Figure S10 Cyclic voltammograms of but-1-yn-1-ylbenzene (0.1 mmol-0.6 mmol) in 0.1 M $^{n}Bu_4NBF_4$ (DMF 3 mL) containing Cs_2CO_3 (0.1 mmol), using a glassy carbon working electrode and Pt wire, $Ag/AgNO_3$ (0.1 M in CH_3CN) as counter and reference electrodes at a 100 mV/s scan rate.

To demonstrate the role of base Cs_2CO_3, CV spectra of allylbenzene derivatives were recorded in the presence or absence of Cs_2CO_3. As shown in the Figure S11, Cs_2CO_3 significantly enhanced the cathodic peaks of allylbenzene derivatives. In other words, Cs_2CO_3 could promote the hydrogen evolution reaction of these substrates.

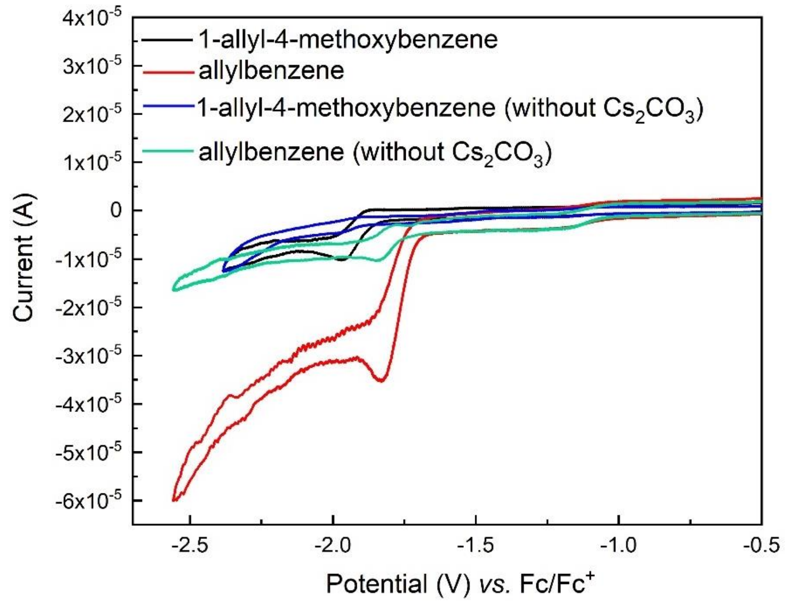

Figure S11 Cyclic voltammograms of allylbenzene variants (0.3 mmol) in 0.1 M $^{n}Bu_4NBF_4$ (DMF 3 mL), using a glassy carbon working electrode and Pt wire, $Ag/AgNO_3$ (0.1 M in CH_3CN) as counter and reference electrodes at a 100 mV/s scan rate.

To elucidate the role of Co^{II}-salen, CV spectra intoducing salen catalyst (**I**) were investigated. As shown in the Figure S12, two reversible couples were detected for **I**, which is assigned to the process of Co^{II}/Co^{III} (-0.13 V) and Co^{II}/Co^{I} (-1.32 V). Upon treating **I** with allylbenzene, an obvious increase (from 36 uA to 101 uA) and shift (from -1.83 to -1.78 V) of cathodic peak of allylbenzene was observed, indicating the catalytic role of cobalt catalyst in HER. A similar result was also detected in the case of but-1-yn-1-ylbenzene, and it showed cathodic peaks at -2.04 V and -2.45 V, that were undetectable or weak in the absence of **I.**

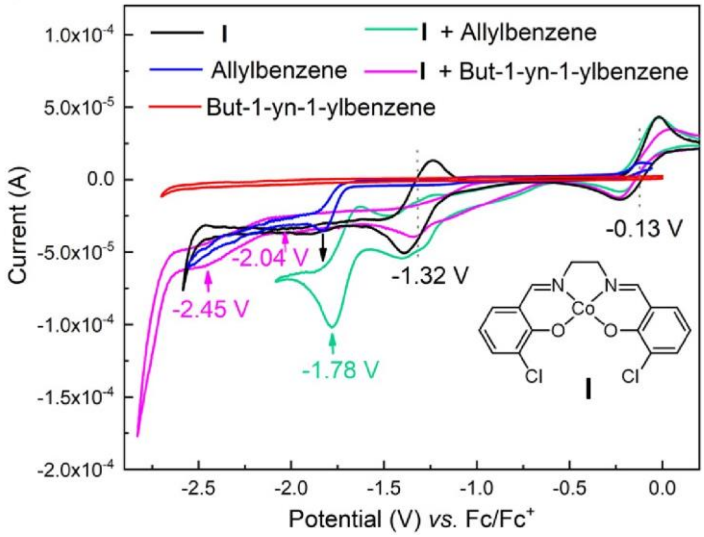

Figure S12 Cyclic voltammograms of substrates (0.3 mmol), Co^{II}-salen (0.025 mmol) in 0.1 M $^{n}Bu_4NBF_4$ (DMF 3 mL), using a glassy carbon working electrode and Pt wire, Ag/$AgNO_3$ (0.1 M in CH_3CN) as counter and reference electrodes at a 100 mV/s scan rate.

To verify the HER peak of allylbenzene, we use acetic acid instead of allylbenzene (Figure 13). Upon treating HOAc with **I**, an similar cathodic peak (green curve) was detected at ~-1.8 V. This result clearly suggests that the cathodic peak at -1.78 V (allylbenezen) should be arising from HER process.

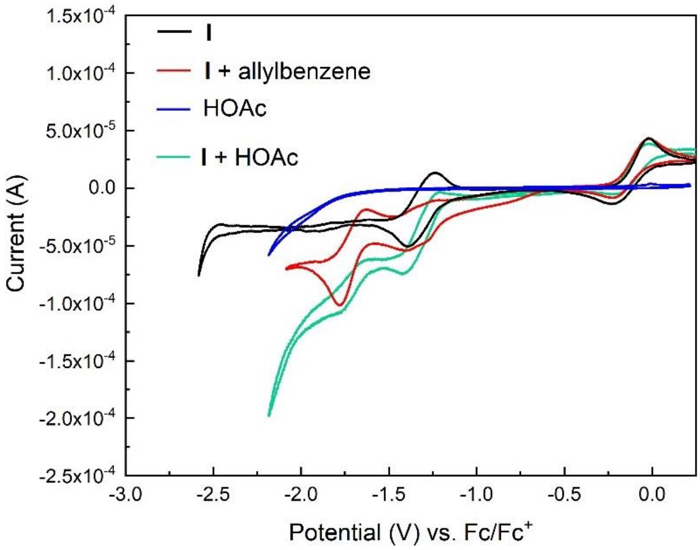

Figure S13 Cyclic voltammograms of substrates (0.3 mmol), Co^{II}-salen (0.025 mmol) in 0.1 M $^{n}Bu_4NBF_4$ (DMF 3 mL), using a glassy carbon working electrode and Pt wire, Ag/$AgNO_3$ (0.1 M in CH_3CN) as counter and reference electrodes at a 100 mV/s scan rate.

We sought to investigate the role of DABCO by mixing it with Co^{II}-salen, and we found that the cathodically inert DABCO could facilitate the redox cycle of Co^{II}/Co^{III} and Co^{I}/Co^{II} with significant increase of the peaks.

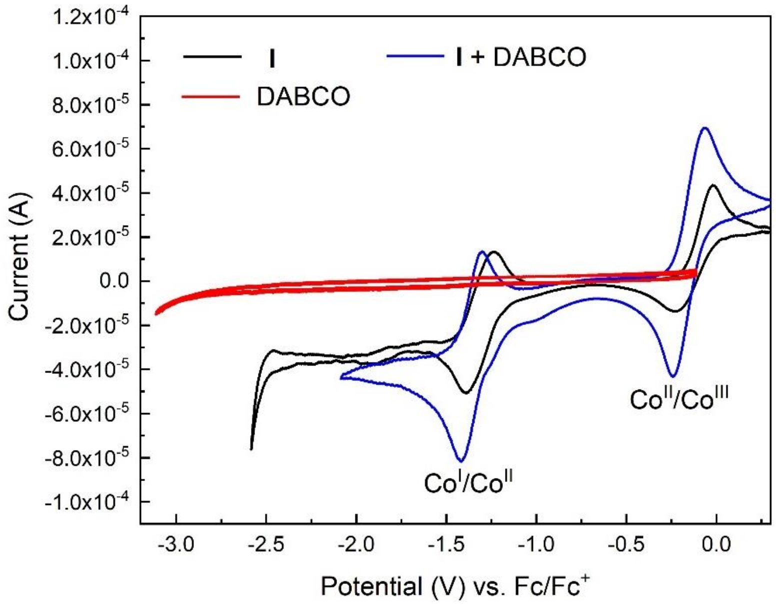

Figure S14 Cyclic voltammograms of DABCO (0.1 mmol), Co^{II}-salen (0.025 mmol) in 0.1 M $^{n}Bu_4NBF_4$ (DMF 3 mL), using a glassy carbon working electrode and Pt wire, $Ag/AgNO_3$ (0.1 M in CH_3CN) as counter and reference electrodes at a 100 mV/s scan rate.

We excluded the hydrogen atom transfer mediator role of DABCO by the titration experiment with allylbenzene (**1a**). As shown in the Figure S15, increasing amount of allylbenzene marginally affected the anodic peak of DABCO.

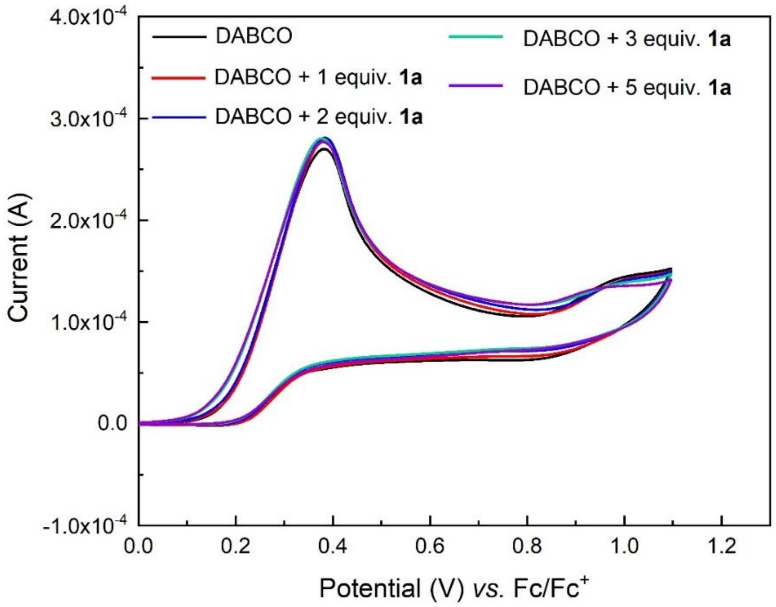

Figure S15 Cyclic voltammograms of DABCO (0.1 mmol), allylbenzene (0.1 mmol-0.5 mmol) in 0.1 M $^{n}Bu_4NBF_4$ (DMF 3 mL), using a glassy carbon working electrode and Pt wire, $Ag/AgNO_3$ (0.1 M in CH_3CN) as counter and reference electrodes at a 100 mV/s scan rate.

We compared the reductive potential of allylbenzene with aldehyde substrates (Figure S16). It clearly showed that allylbenzene (E_{red} = -1.78 V) is more susceptible to cathodic reduction compared to aldehydes (E_{red} = -1.96-2.74 V). This result indicates that the electrochemical allylation should be initiated by the HER of the excessive allylbenzene rather than the reduction of aldehyde.

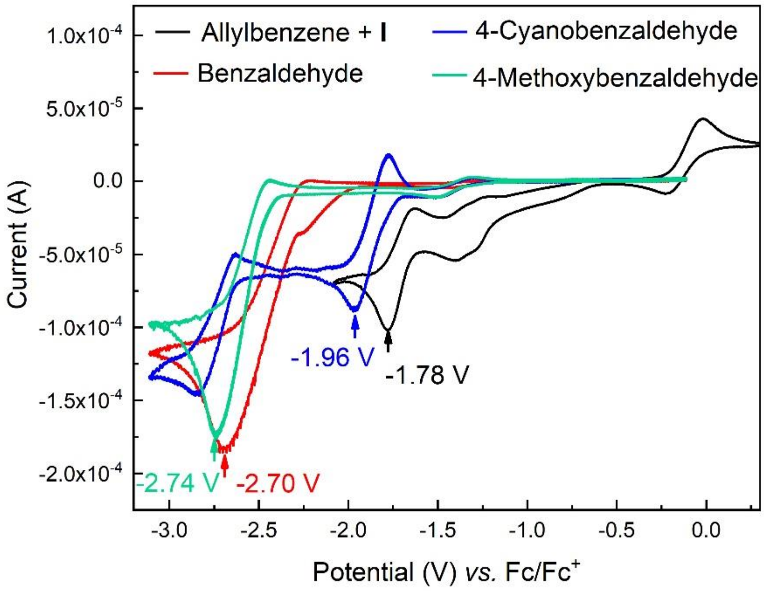

Figure S16 Cyclic voltammograms of substrates (0.3 mmol) in 0.1 M $^{n}Bu_4NBF_4$ (DMF 3 mL), using a glassy carbon working electrode and Pt wire, Ag/$AgNO_3$ (0.1 M in CH_3CN) as counter and reference electrodes at a 100 mV/s scan rate.

The anodic behavior of substrates was also investigated (Figure S17). It shows that DABCO and the substrates allylbenezene is anodically active in the solution of DMF.

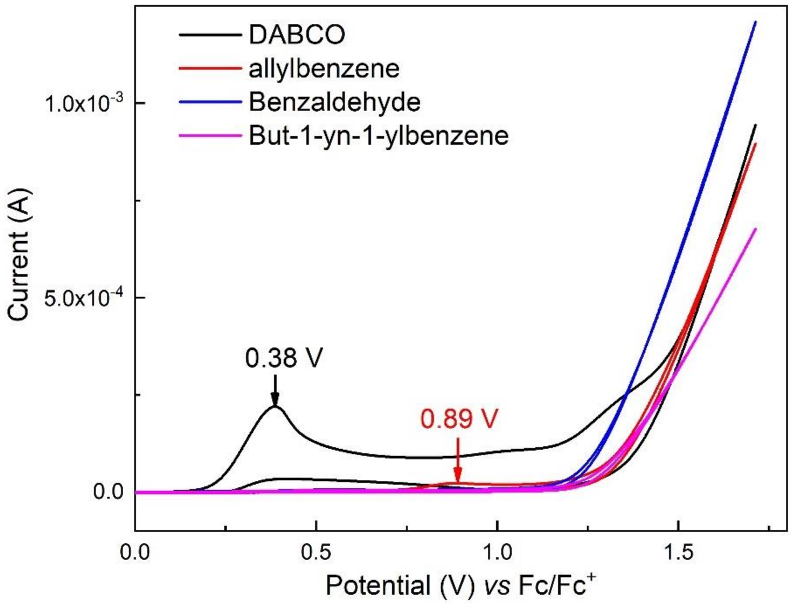

Figure S17 Cyclic voltammograms of substrates (0.1 mmol) in 0.1 M $^{n}Bu_4NBF_4$ (DMF 3 mL), using a glassy carbon working electrode and Pt wire, Ag/$AgNO_3$ (0.1 M in CH_3CN) as counter and reference electrodes at a 100 mV/s scan rate.

We also studied the anodic potential of solvents in the acetonitrile, and DMF showed an anodic peak at 1.95V. This result suggests that the sacrificial oxidation of substrates and DABCO might be preferred over anode surface.

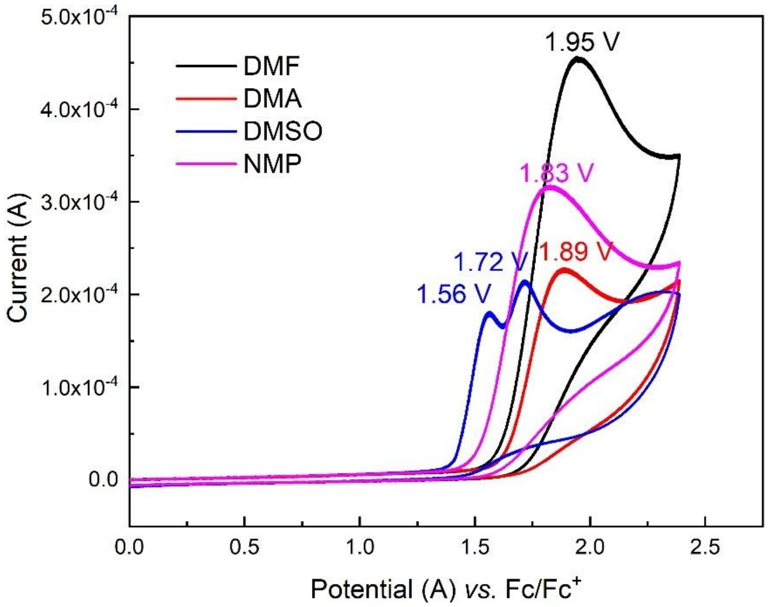

Figure S18 Cyclic voltammograms of solvents (0.02 M) in 0.1 M $LiClO_4$ (CH_3CN), using a glassy carbon working electrode and Pt wire, Ag/$AgNO_3$ (0.1 M in CH_3CN) as counter and reference electrodes at a 100 mV/s scan rate.

4. GC analysis of reaction atmosphere

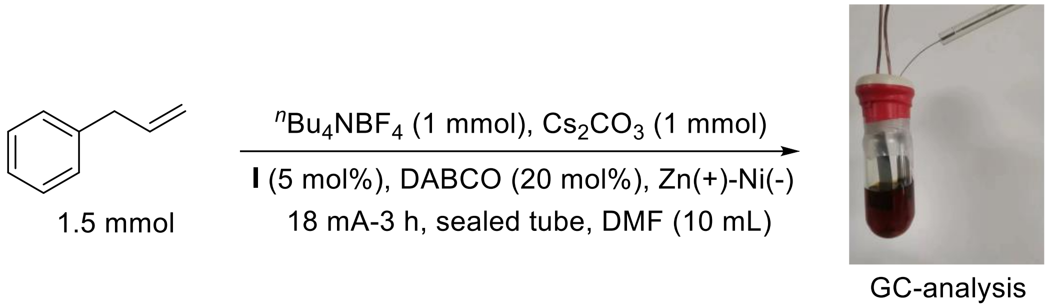

To verify the hydrogen evolution reaction of allylbenzene, we also conducted a GC analysis for the reaction atmosphere. After electrolysis for 3 hours, the headspace atmosphere was subjected to GC analysis. As shown in the GC spectra, substantial amount of hydrogen was detected by comparing with the standard sample containing H_2, O_2, N_2. The result clearly confirmed the HER process of allylbenzene.

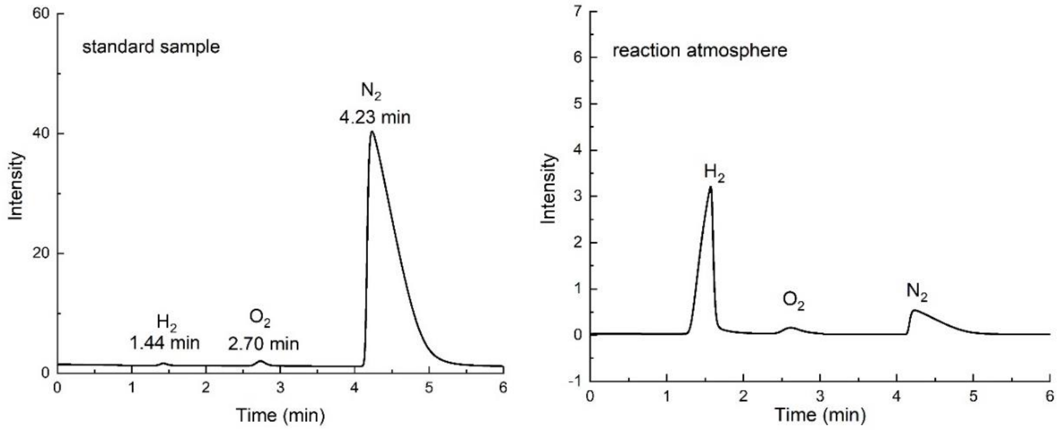

Figure S19 GC spectrum of standard sample (containing H_2, O_2, N_2) and the reaction atmosphere (after reaction)

5. Optimization of reaction conditions

Table S1. Optimization of electrochemical isomerizing allylation of aldehyde (**Condition II**)[a]

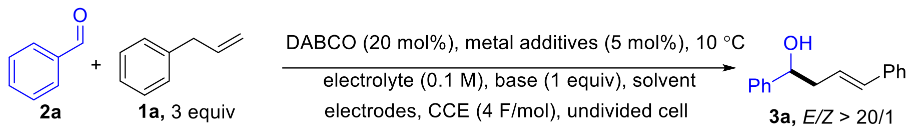

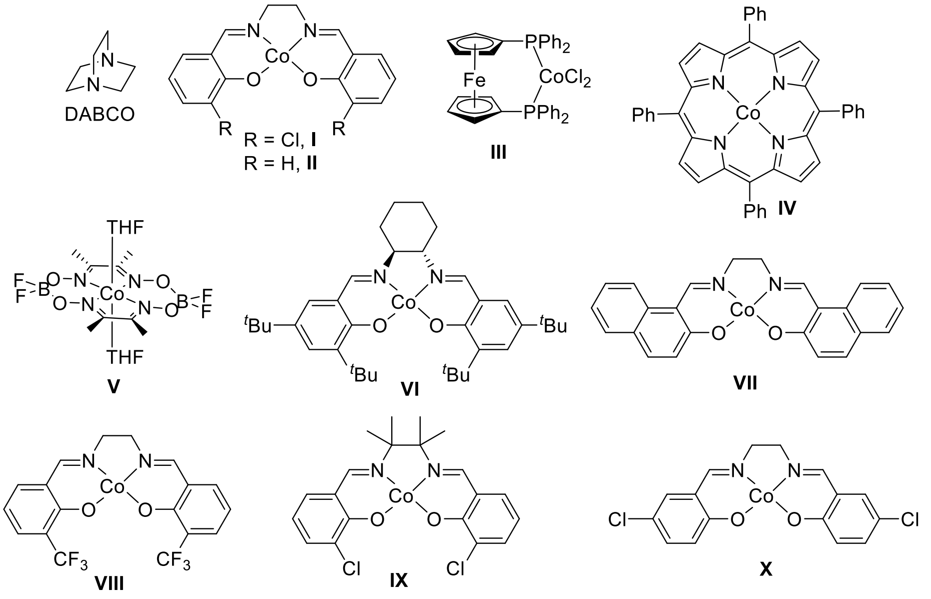

Entry	Electrolyte	Electrodes	Solvent	Base	metal additives	Yield (%)[b]
1	$^nBu_4NBF_4$	C rod (+)-Pt(-)	DMA	none	$CrCl_2$	0
2	$^nBu_4NBF_4$	C rod (+)-Pt(-)	DMA	none	$CoCl_2$	4
3	$^nBu_4NBF_4$	C rod (+)-Pt(-)	DMA	none	$NiCl_2$	0
4	$^nBu_4NBF_4$	C rod (+)-Pt(-)	DMA	none	**II**	13
5	$^nBu_4NBF_4$	C rod (+)-Pt(-)	DMA	Na_2CO_3	**II**	25
6	$^nBu_4NBF_4$	C rod (+)-Ni(-)	DMA	Na_2CO_3	**II**	39
7	$^nBu_4NBF_4$	C rod (+)-Cu(-)	DMA	Na_2CO_3	**II**	29
8	$^nBu_4NBF_4$	C rod (+)-Pb(-)	DMA	Na_2CO_3	**II**	21
9	$^nBu_4NBF_4$	C rod (+)-Zn(-)	DMA	Na_2CO_3	**II**	21
10	$^nBu_4NClO_4$	C rod (+)-Ni(-)	DMA	Na_2CO_3	**II**	33
11	$^nBu_4NOAc$	C rod (+)-Ni(-)	DMA	Na_2CO_3	**II**	trace
12	$^nBu_4NBr$	C rod (+)-Ni(-)	DMA	Na_2CO_3	**II**	21
13	$^nBu_4NBF_4$	C rod (+)-Ni(-)	DMF	Na_2CO_3	**II**	54
14	$^nBu_4NBF_4$	C rod (+)-Ni(-)	DMSO	Na_2CO_3	**II**	40
15	$^nBu_4NBF_4$	C rod (+)-Ni(-)	NMP	Na_2CO_3	**II**	39
16	$^nBu_4NBF_4$	C rod (+)-Ni(-)	DMF	K_2CO_3	**II**	49
17	$^nBu_4NBF_4$	C rod (+)-Ni(-)	DMF	Na_2CO_3	**II**	50
18	$^nBu_4NBF_4$	C rod (+)-Ni(-)	DMF	Cs_2CO_3	**II**	69
19	$^nBu_4NBF_4$	C felt (+)-Ni(-)	DMF	Cs_2CO_3	**II**	72
20	$^nBu_4NBF_4$	C felt (+)-Ni(-)	DMF	Cs_2CO_3	**I**	82
21	$^nBu_4NBF_4$	C felt (+)-Ni(-)	DMF	Cs_2CO_3	**III**	52
22	$^nBu_4NBF_4$	C felt (+)-Ni(-)	DMF	Cs_2CO_3	**IV**	69
23	$^nBu_4NBF_4$	C felt (+)-Ni(-)	DMF	Cs_2CO_3	**V**	71
24	$^nBu_4NBF_4$	C felt (+)-Ni(-)	DMF	Cs_2CO_3	**VI**	73
25	$^nBu_4NBF_4$	C felt (+)-Ni(-)	DMF	Cs_2CO_3	**VII**	78
26	$^nBu_4NBF_4$	C felt (+)-Ni(-)	DMF	Cs_2CO_3	**VIII**	79
27	$^nBu_4NBF_4$	C felt (+)-Ni(-)	DMF	Cs_2CO_3	**IX**	77
28	$^nBu_4NBF_4$	C felt (+)-Ni(-)	DMF	Cs_2CO_3	**X**	75
29[c]	$^nBu_4NBF_4$	C felt (+)-Ni(-)	DMF	Cs_2CO_3	**I**	65
30	$^nBu_4NBF_4$	C felt (+)-Ni(-)	DMF	none	**I**	53
31	$^nBu_4NBF_4$	C felt (+)-Ni(-)	DMF	Cs_2CO_3	none	61
32[c]	$^nBu_4NBF_4$	C felt (+)-Ni(-)	DMF	none	none	32
33	$^nBu_4NBF_4$	C felt (+)-Ni(-)	CH_3CN	Cs_2CO_3	**I**	trace
34	$^nBu_4NBF_4$	C felt (+)-Ni(-)	DMA	Cs_2CO_3	**I**	58
35	$^nBu_4NBF_4$	C felt (+)-Ni(-)	DMSO	Cs_2CO_3	**I**	60
36	$^nBu_4NBF_4$	C felt (+)-Ni(-)	NMP	Cs_2CO_3	**I**	54
37	$^nBu_4NBF_4$	C felt (+)-Cu(-)	DMF	Cs_2CO_3	**I**	61
38	$^nBu_4NBF_4$	C felt (+)-Pt(-)	DMF	Cs_2CO_3	**I**	56
39	$^nBu_4NBF_4$	C felt (+)-Pb(-)	DMF	Cs_2CO_3	**I**	45
40[d]	$^nBu_4NBF_4$	C felt (+)-Ni(-)	DMF	Cs_2CO_3	**I**	65

[a] Reaction conditions: **1a** (1.5 mmol), **2a** (0.5 mmol), electrolyte (1 mmol), base (1 mmol), DABCO (0.1 mmol), metal additive (0.025 mmol), graphite anode, metal cathode, CCE (18 mA, 3h), 10˚C; [b] isolated yield. [c] Removing DABCO. [d] Reaction at 40˚C.

We commenced the electrochemical allylation with DMA as solvent, $^nBu_4NBF_4$ as electrolyte, DABCO as an additive, graphite rod and platinum plate as anode and cathode, respectively. After screening metal additives (entry 1-3), previously reported chromium salt failed to afford any

allylation product, while cobalt(II) chloride afford trace of product (4%). Inspired by the promising result, we replaced the simple cobalt salt with salen-Co complex (**II**), and further increase of yield was observed (entry 4). Upon introducing inorganic base (Na_2CO_3), linear allylation product **3a** was observed in 25% yield (entry 5). Variation of cathodes (entry 6-9) showed that nickel cathode was the optimal one (with 39% yield). Changing $^nBu_4NBF_4$ to other electrolytes (entry 10-12) failed to give better yields. Specifically, $^nBu_4NOAc$ directly shut down the reaction (entry 11). Solvent effect was further investigated (entry 13-15), and DMF showed significant enhancement effect on the reaction efficiency. Additionally, we also investigated other bases, and strong base Cs_2CO_3 gave the desired product **3a** in 69% yield. Graphite felt anode further improved the reaction yield (72%). Finally, a broad range of cobalt complexes were further evaluated (entry 20-29) and complex **I** proved to be the most efficient one (entry 20). Control experiments by varying parameters of the optimal conditions were next performed as shown from entry 30 to 40.

Table S2. Optimization of electrochemical isomerizing allylation of aldehyde (**Condition I**)[a]

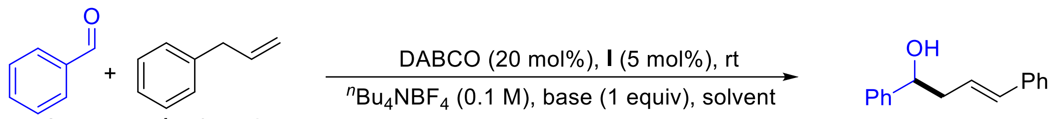

2a **1a,** 3 equiv **3a,** *E/Z* > 20/1

Entry	Electrodes	Solvent	Base	Yield (%)[b]
1	Zn(+)-Ni(-)	DMF	Cs_2CO_3	93
2	Mg (+)-Ni(-)	DMF	Cs_2CO_3	79
3	Al (+)-Ni(-)	DMF	Cs_2CO_3	29
4	Zn(+)-Ni(-)	DMA	Cs_2CO_3	78
5	Zn(+)-Ni(-)	CH_3CN	Cs_2CO_3	0
6	Zn(+)-Ni(-)	THF	Cs_2CO_3	trace
7	Zn(+)-Pt(-)	DMF	Cs_2CO_3	64
8	Zn(+)-C(-)	DMF	Cs_2CO_3	48
9	Zn(+)-Pb(-)	DMF	Cs_2CO_3	30
10	Zn(+)-Ni(-)	DMF	Na_2CO_3	21
11	Zn(+)-Ni(-)	DMF	K_2CO_3	45
12	Zn(+)-Ni(-)	DMF	-	10
13[c]	Zn(+)-Ni(-)	DMF	Cs_2CO_3	70
14[d]	Zn(+)-Ni(-)	DMF	Cs_2CO_3	72
15[e]	Zn(+)-Ni(-)	DMF	Cs_2CO_3	73
16[f]	Zn(+)-Ni(-)	DMF	Cs_2CO_3	0
17[g]	-	DMF	Cs_2CO_3	0

[a] Reaction conditions: **1a** (1.5 mmol), **2a** (0.5 mmol), electrolyte (1 mmol), base (1 mmol), DABCO (0.1 mmol), metal additive (0.025 mmol), CCE (18 mA, 3h), room temperature; [b] isolated yield. [c] Removing DABCO and **I**. [d] Removing **I**. [e] Removing DABCO. [f] No electricity. [g] Using magnesium, zinc or manganese powder to replace electricity.

We also found that graphite felt anode can be replaced with sacrificial electrodes (entry 1-3), and zinc proved to be the optimal. Replacing polar solvent DMF to other solvents led to deteriorated yields (entry 4-6). Specifically, THF failed to give any product, presumbely the low polar solvent would suppress the dissociation of weak acidic substrates **1a**. Acetonitrile with more acidic C-H bond only give an aldol product arising from acetonitrile and benzaldehyde.

Changing nickel cathode to other cathodes failed to give better yields (entry 7-9). Specifically, lead plate led to deminished yield since it has a high overpotential for HER process. Effect of bases revealed that carbonate base played a crucial role for the reaction efficiency (entry 10-12). Control experiments removing DABCO or Co^{II}-salen (**I**) supports that the combination of these two additives is criticial for the high efficiency (entry 13-15). The reaction carried out in the absence of electricity or with reductants (zinc, magnesium, manganese powder) completely shuts down the reaction (entry 16-17), even with prolonged reaction time (24 h). This result highlights both the novelty and power of this electrochemical protocol.

Table S3. Optimization of electrochemical allenylation of aldehyde[a]

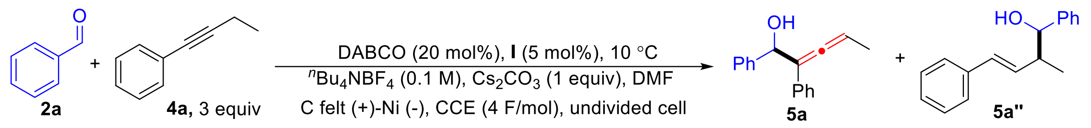

Entry	Deviation from standard conditions	Yield of 5a (%)[b]
1	None	73
2	Removal of Cs_2CO_3	50
3	Removal of DABCO	51
4	Removal of **I**	57
5	Removal of Cs_2CO_3, DABCO and **I**	22
6	DMA or DMSO or NMP as solvent	60, 54, 57
7	CH_3CN as solvent	trace
8	**II** or **III** or **IV** as additives	68, 65, 61
9	Li_2CO_3 or Na_2CO_3 or K_2CO_3 as base	58, 62, 65
10	Pt or Cu or Pb as cathode	60, 68, 41
11[c]	Zn as anode	65 (**5a/5a''** = 1.9/1)
12	Reaction at 40 ˚C	62
13	Using Mg/ Zn/ Mn powder instead of electricity	0
14	No electricity, 24 h	0

[a] Reaction conditions: **1a** (0.5 mmol), **4a** (1.5 mmol), $^nBu_4NBF_4$ (1 mmol), Cs_2CO_3 (1 mmol), DABCO (0.1 mmol), **I** (0.025 mmol), graphite felt anode, nickel plate cathode, CCE (18 mA, 3h), 10 ˚C; [b] isolated yield.[c] The ratio of product was determined by ^1H NMR.

Variations on the optimal conditions of electrochemical allenylation were also performed, and the necessity of Cs_2CO_3, DABCO and cobalt additives **I** was demonstrated by the results (entry 2-5). Investigations of solvent effect showed that redox-active solvents (entry 6) are beneficial to the reaction compared to the inert one acetonitrile (entry 7). Change cobalt additive to other complexes failed to give better yields (entry 8). Replacing Cs_2CO_3 with weaker bases led to slightly

lower yields and some unidentified side products (entry 9). Study on the other cathodes showed that nickel plate enabled the highest efficiency (entry 10). Change graphite felt anode to sacrificial anode (zinc) led to undesired products **5a''** (entry 11). This poor reaction selectivity can be explained by that the coordination effect of zinc ion improve the reactivity (electrophilicity) of benzaldehyde. Improving the reaction temperature (to 40 ˚C) resulted in deteriorated yield, presumably due to the instability of carbanions generating in the reaction (entry 12). Finally, control experiments using reductant to replace electricity or without electricity directly shut down the reaction, suggesting that electricity is necessary for the electrochemical allenylation (entry 13-14).

Ratio of **5a/5a''** (entry 11)

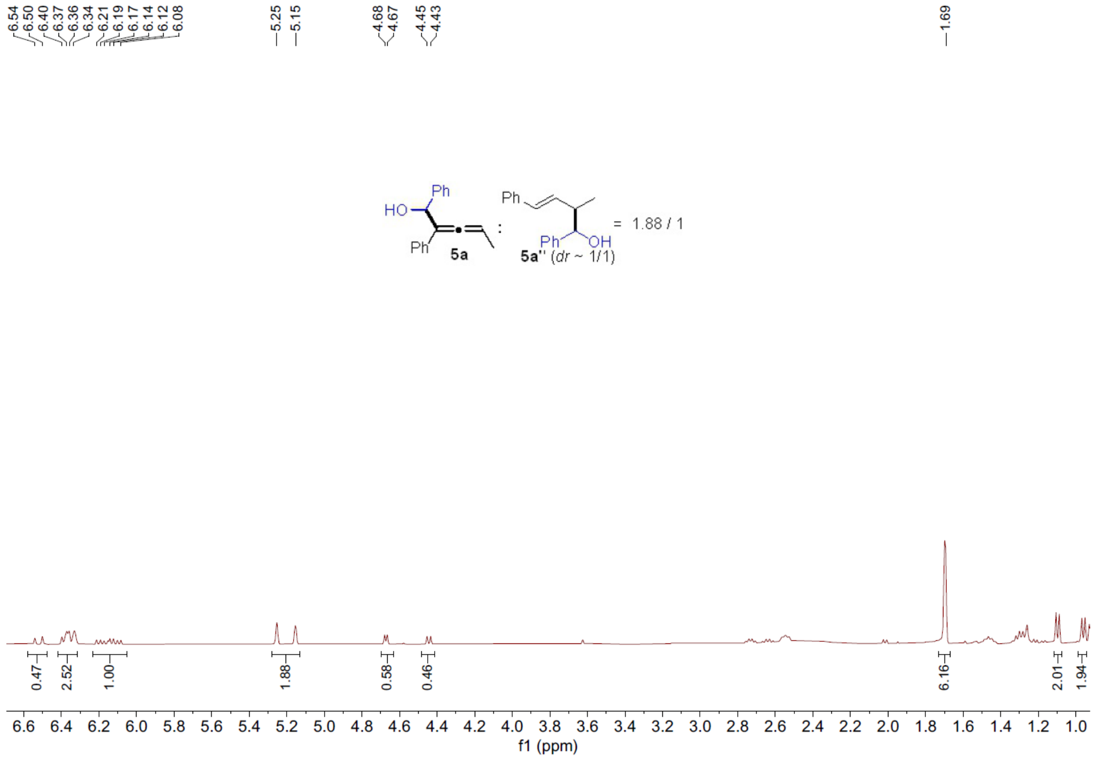

Figure S20 ^{1}H NMR spectra of the mixed product **5a** and **5a''**

6. General procedure for the electrochemical allylation and allenylation

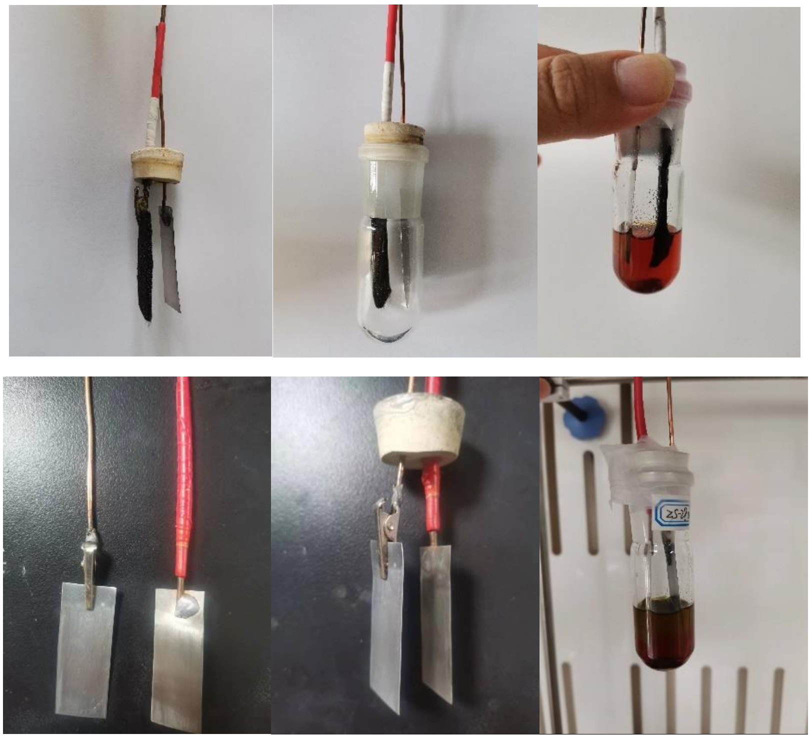

Figure S21 Electrolysis setup (graphite felt, zinc plate/nickel plate: width 1.8 cm, immersion depth 1.5 cm)

3a as example (Condition I)

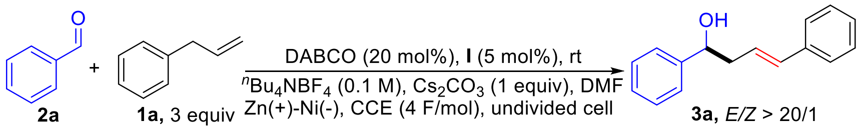

An undivided cell was equipped with a magnet stirrer, nickel plate (1.8 *1.5 cm^2), zinc plate (1.8 *1.5 cm^2), as cathode and anode, respectively (the electrolysis setup is shown in Figure S21). The substrate benzaldehyde (52 µL, 0.5 mmol), allylbenzene **2a** (199 µL, 1.5 mmol), Cs_2CO_3 (163 mg, 0.5 mmol), DABCO (11 mg, 0.1 mmol), **I** (10 mg, 0.025 mmol) and $^nBu_4NBF_4$ (329 mg, 1 mmol) were added to the solvent DMF (10 mL). The resulting mixture was allowed to stir and electrolyze under constant current condition (18 mA, J = 6.7 mA·cm^{-2}) at room temperature for 3 hours. The reaction mixture was subsequently poured into water (100 mL) and extracted with ethyl acetate (40 mL×3). The combined organic phases were washed with saturated brine solution (100 mL). The volatile solvent was then removed with a rotary evaporator, and the residue was purified by column chromatography (PE/ EA= 8/1-5/1, v/v) on silica gel to afford the desired product **3a** (104 mg) in 93 % yield.

3a as example (Condition II)

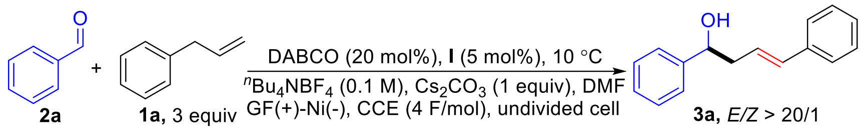

An undivided cell was equipped with a magnet stirrer, nickel plate (1.8 *1.5 cm^2), graphite felt (1.8 *1.5 cm^2), as cathode and anode, respectively (the electrolysis setup is shown in Figure S21). The substrate benzaldehyde (52 μL, 0.5 mmol), allylbenzene **2a** (199 μL, 1.5 mmol), Cs_2CO_3 (163 mg, 0.5 mmol), DABCO (11 mg, 0.1 mmol), **I** (10 mg, 0.025 mmol) and $^{n}Bu_4NBF_4$ (329 mg, 1 mmol) were added to the solvent DMF (10 mL). The resulting mixture was allowed to stir and electrolyze under constant current condition (18 mA, J = 6.7 mA·cm^{-2}) at 10 °C for 3 hours. The reaction mixture was subsequently poured into water (100 mL) and extracted with ethyl acetate (40 mL×3). The combined organic phases were washed with saturated brine solution (100 mL). The volatile solvent was then removed with a rotary evaporator, and the residue was purified by column chromatography (PE/ EA= 8/1-5/1, v/v) on silica gel to afford the desired product **3a** (92 mg) in 82 % yield.

The allylation between **1bb** and electrophiles **6** or **8** was conducted with **Condition II.** The reactions to generate other carbanions (**10-13, 5an**) were also performed with **Condition II**, and the carbanion precursors were used in the place of allylbenzene.

3a as example (Condition III)

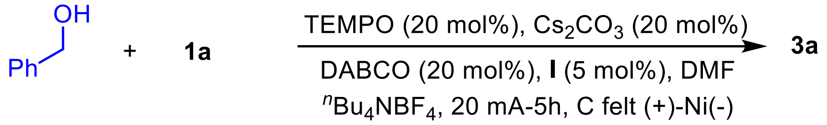

An undivided cell was equipped with a magnet stirrer, nickel plate (1.8 *1.5 cm^2), graphite felt (1.8 *1.5 cm^2), as cathode and anode, respectively (the electrolysis setup is shown in Figure S21). The substrate benzyl alcohol (52 μL, 0.5 mmol), allylbenzene **2a** (199 μL, 1.5 mmol), Cs_2CO_3 (32 mg, 0.1 mmol), DABCO (11 mg, 0.1 mmol), **I** (10 mg, 0.025 mmol), TEMPO (17 mg, 0.1 mmol) and $^{n}Bu_4NBF_4$ (329 mg, 1 mmol) were added to the solvent DMF (10 mL). The resulting mixture was allowed to stir and electrolyze under constant current condition (20 mA, J = 7.4 mA·cm^{-2}) at room temperature for 5 hours. The reaction mixture was subsequently poured into water (100 mL) and extracted with ethyl acetate (40 mL×3). The combined organic phases were washed with saturated brine solution (100 mL). The volatile solvent was then removed with a rotary evaporator, and the residue was purified by column chromatography (PE/ EA= 8/1-5/1, v/v) on silica gel to afford the desired product **3a** (60 mg) in 54 % yield.

7. Procedure for gram scale reaction and derivatization of products

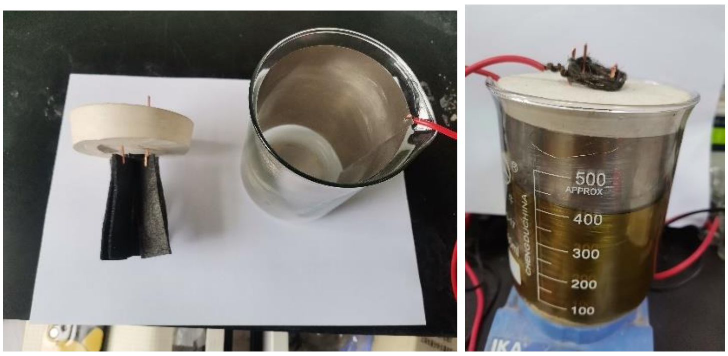

Figure S22 Gram electrolysis device (graphite felt (4 pieces): immersion depth 5.0 cm, width 3.0 cm; nickel plate: immersion depth 5.5 cm, width 25.0 cm)

Gram scale reaction

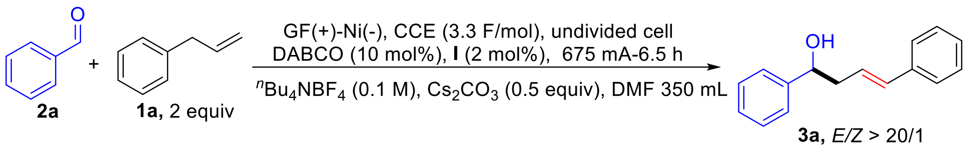

An undivided cell was equipped with a magnet stirrer, nickel plate (25 *5.5 cm^2), 4 pieces of graphite felt (5 *3 cm^2), as cathode and anode, respectively (the electrolysis setup is shown in Figure S22). The substrate benzaldehyde (5.2 mL, 50 mmol), allylbenzene **1a** (13 mL, 100 mmol), Cs_2CO_3 (8.2 g, 50 mmol), DABCO (561 mg, 5 mmol), **I** (400 mg, 1 mmol) and $^nBu_4NBF_4$ (11.5 g, 35 mmol) were added to the solvent DMF (350 mL). The resulting mixture was allowed to stir and electrolyze at constant current condition (675 mA) at room temperature for 6.5 hours. Then the reaction mixture was poured into water (1000 mL) and extracted with ethyl acetate (150 mL×3). Combined organic phase was washed with saturated brine solution (500 mL). The volatile solvent was then removed with a rotary evaporator, and the residue was purified by column chromatography (PE/EA = 8/1-5/1, v/v) on silica gel to afford the desired product **3a** (7.95 g) in 71 % yield.

Dehydration of product 3a and 3o (3a as an example)

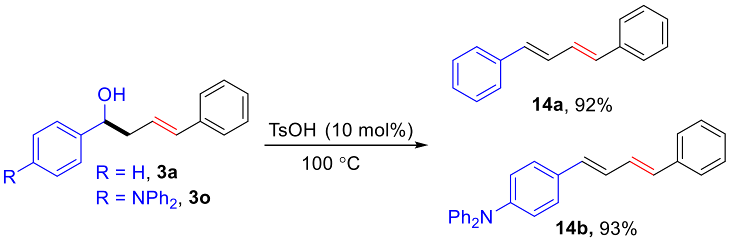

A flask was charged with **3a** (0.5 mmol, 112 mg), *p*-toluenesulfonic acid monohydrate (0.05 mmol, 10 mg) and toluene (3 mL). The resulted mixture was heated to 100 °C. After 10 hours, the volatile solvent was removed with a rotary evaporator. The residue was purified by column chromatography (PE/ EA= 50/1-20/1, v/v) on silica gel to afford the desired product **14a** (95 mg) in 92 % yield.

Epoxidation of product 3a

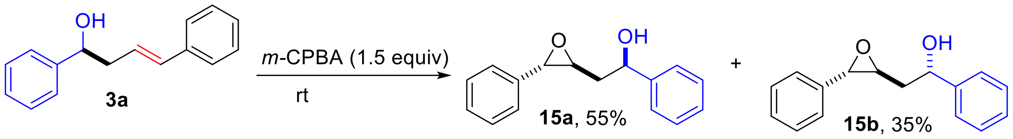

A flask was charged with **3a** (1 mmol, 224 mg), *m*-chloroperoxybenzoic acid (1.5 mmol, 259 mg) and DCM (3 mL). After stirring at room temperature 8 hours, the volatile solvent was removed with a rotary evaporator. The residue was purified by column chromatography (PE/ EA= 5/1-2/1, v/v) on silica gel to afford the desired product isomer I **15a** (133 mg) and isomer II **15b** (83 mg).

Oxidation of 5a with NBS

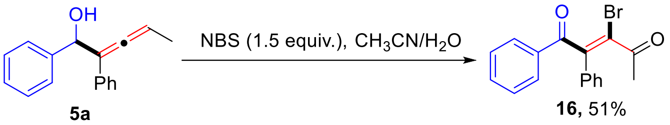

A flask was charged with **5a** (0.5 mmol, 118 mg), *N*-bromosuccinimide (0.75 mmol, 134 mg) and CH_3CN/H_2O (2/0.2 mL). After stirring at room temperature 24 hours, the volatile solvent was removed with a rotary evaporator. The residue was purified by column chromatography (PE/ EA= 30/1-20/1, v/v) on silica gel to afford the desired product **16** (84 mg) in 51 % yield.

Cyclization of 5a

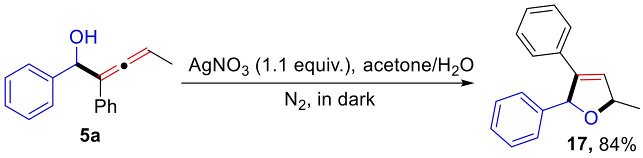

5a (0.5 mmol, 118 mg), $AgNO_3$ (0.55 mmol, 94 mg), acetone/H_2O (3/0.3 mL) and a stir bar were added to a sealed tube under nitrogen. After being stirred at room temperature for 12 h in dark, the mixture was evaporated under vacuum. The desired product was isolated by column chromatography (PE/ EA= 40/1-30/1, v/v) on silica gel to afford the desired product **17** (99 mg) in 84 % yield.

8. Procedure and details for control experiments

Allylation and allenylation of aldehydes using ^{n}BuLi as a base

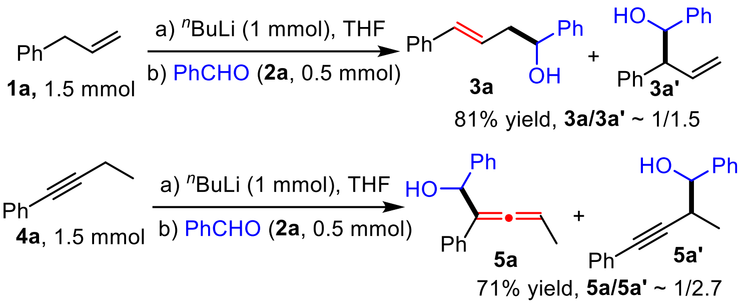

We conducted the above transformations with following procedure (**3a/3a'** as an example): To an oven dried Schlenk tube was added a solution of allylbenzene (199 μL, 1.5 mmol) in anhydrous THF (3 mL) under nitrogen. The resulting solution was cooled to -78 °C, and subsequently treated with ^{n}BuLi (0.4 mL, 1.0 mmol). After stirring at same temperature for 2 hours, benzaldehyde (52 μL, 0.5 mmo) was added to the reddish-brown solution. The reaction mixture was allowed to warm to room temperature in 2 hours, and further stirred for half an hour. Then the reaction was quenched with saturated NH_4Cl solution (3 mL) and poured into water (50 mL). After extraction with ethyl acetate (20 mL×3), the combined organic phases were washed with saturated brine solution (100 mL). The volatile solvent was then removed with a rotary evaporator, and the residue was purified by column chromatography (PE/ EA= 5/1-3/1, v/v) on silica gel to afford the desired product **3a/3a'** (91 mg) in 81% yield. The ratio of product was determined by ^{1}H NMR (as shown below).

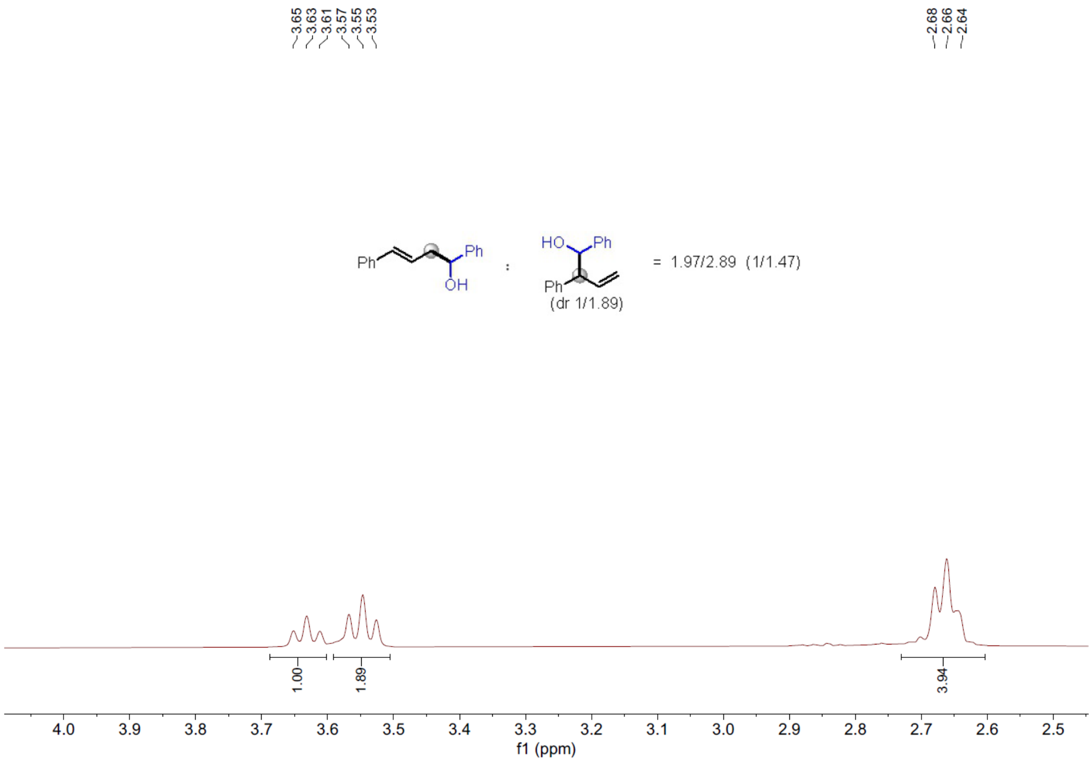

Figure S23 ^{1}H NMR spectrum of mixed product **3a** and **3a'**

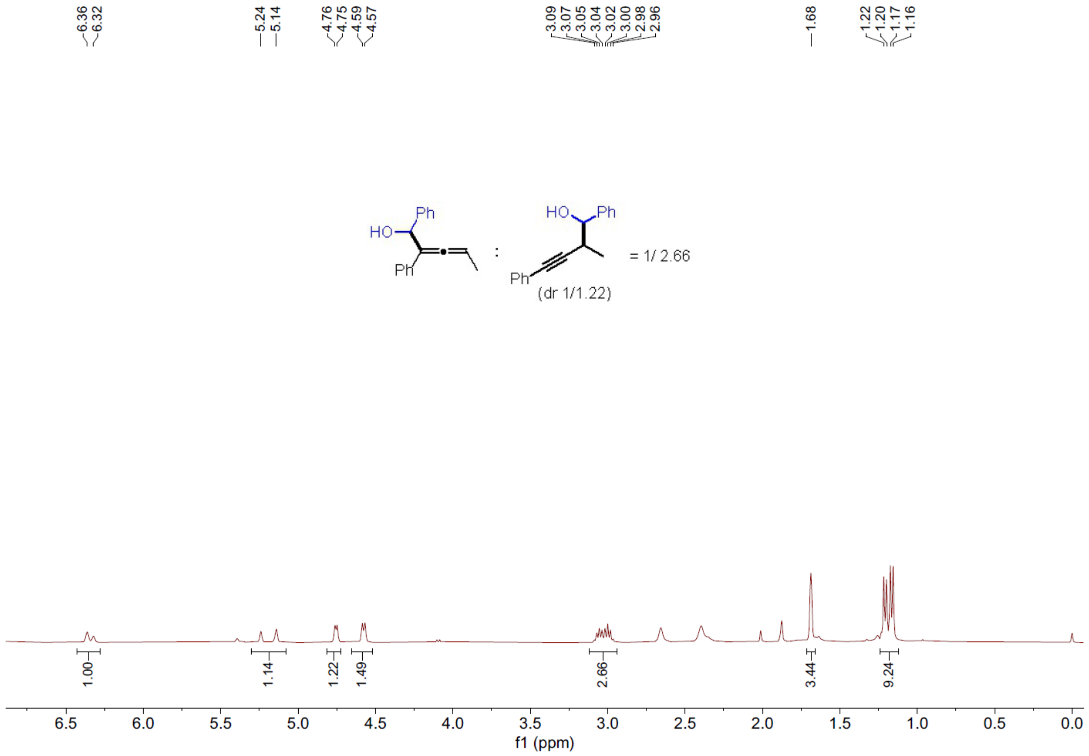

Figure S24 ^{1}H NMR spectrum of mixed product **5a** and **5a'**

Allylation and allenylation of aldehydes using NaH as a base

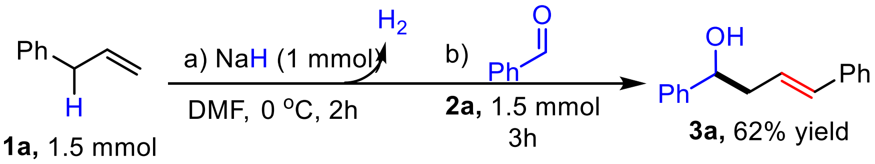

We conducted the above transformation with following procedure: To an oven dried Schlenk tube was added a solution of allylbenzene (199 µL, 1.5 mmol) in anhydrous DMF (5 mL) under nitrogen. The resulting solution was cooled to 0 °C, and subsequently treated with NaH (1.0 mmol, 40 mg, 60% in mineral oil). After stirring at same temperature for 2 hours, benzaldehyde (52 µL, 0.5 mmo) was added to the brown solution. The reaction mixture was allowed to warm to room temperature and further stirred for 3 hours. Then the reaction was quenched with saturated NH_4Cl solution (3 mL) and poured into water (50 mL). After extraction with ethyl acetate (20 mL×3), the combined organic phases were washed with saturated brine solution (100 mL). The volatile solvent was then removed with a rotary evaporator, and the residue was purified by column chromatography (PE/ EA= 5/1-3/1, v/v) on silica gel to afford the desired product **3a** (69 mg) in 62% yield. The ratio of product was determined by ^{1}H NMR (as shown below).

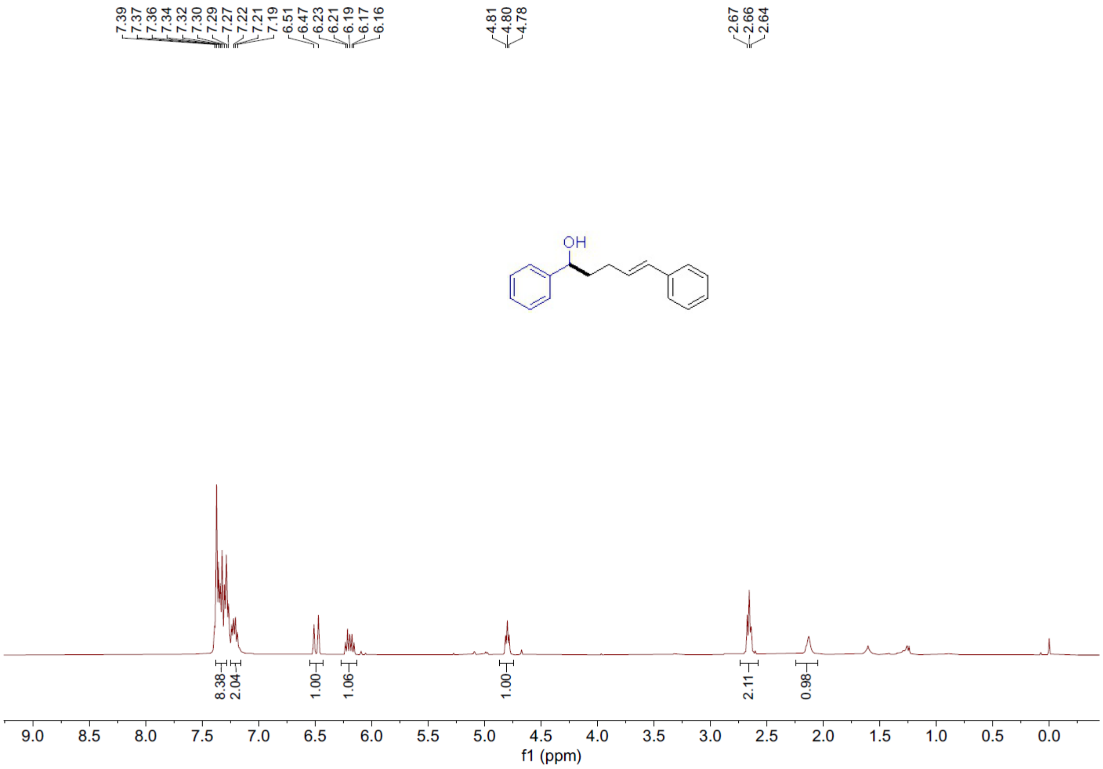

Figure S25 ^{1}H NMR spectrum of product **3a**

Radical clock experiment

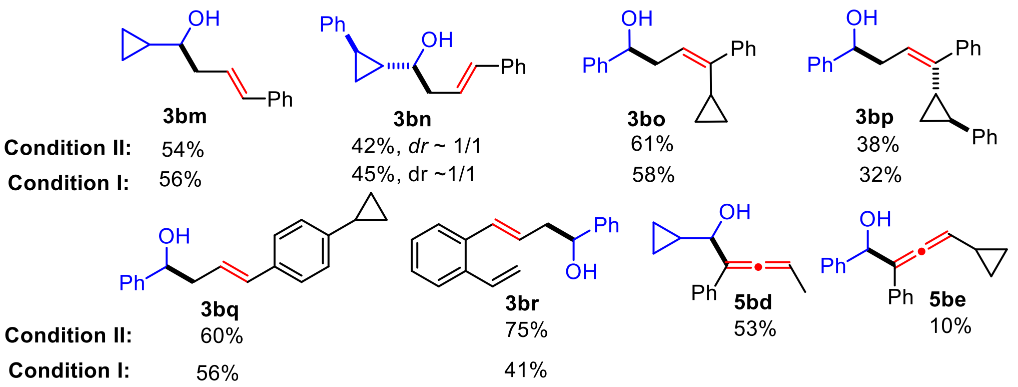

A broad range of substrates bearing cyclopropyl grpoup were subjected to the standard conditions (**Condition I/II**). The desired products were uniformly accessed without detection of any radical-initiated ring-expansion products. These results support that the reaction should proceed via an ionic pathway.

The effect of acidic additives

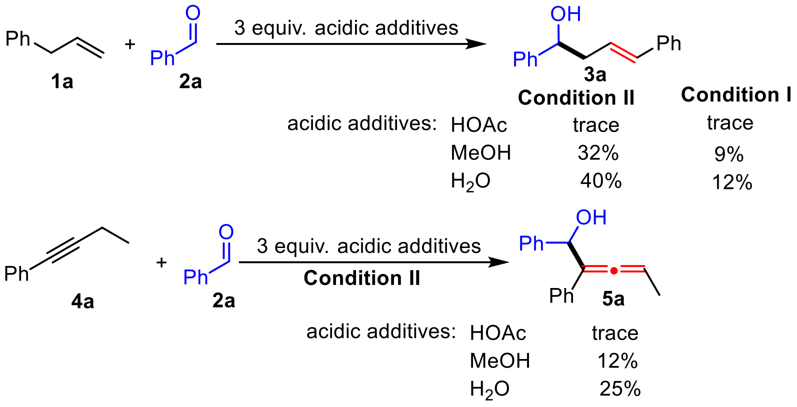

To further verify the carbanion species in the reaction, some acidic additives has been introduced to the reaction system. It revealed that these acidic additives significantly suppress the reaction yields. These results can be explained by that the HER process of acidic additives is favored over the weakly acidic substrates **1a/4a**.

Radical suppression experiment

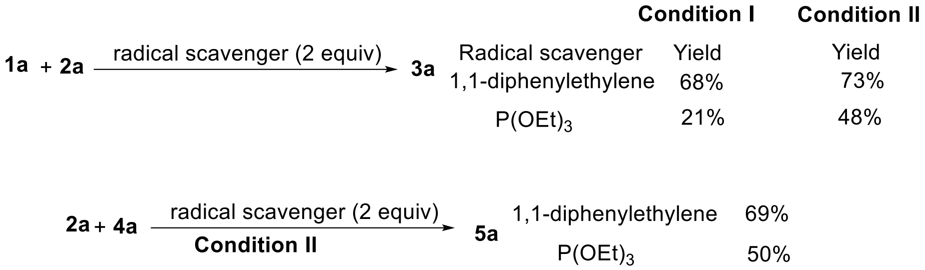

Some radical scavengers, 1,1-diphenylethylene and $P(OEt)_3$ were also tested in the electrochemical allylation and allenylation. 1,1-Diphenylethylene marginally affected the reaction performance, while $P(OEt)_3$ led to an obvious erosion on the reaction yield. We speculate that the carbanions generating in the reaction can be intercepted by $P(OEt)_3$ via nucleophilic addition. The above result suggests that the ionic pathway should be the dominant pathway in the reaction. Additionally, radical-like species Co^{III}-H generating in the reaction might be affected by the radical scavengers.

Deuterium incorporation experiment

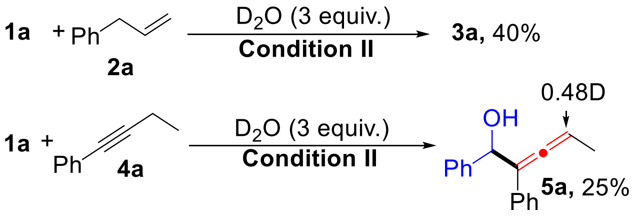

An undivided cell was equipped with a magnet stirrer, nickel plate (1.8 *1.5 cm^2), graphite felt (1.8 *1.5 cm^2), as cathode and anode, respectively (the electrolysis setup is shown in Figure S21). The substrate benzaldehyde (52 μL, 0.5 mmol), allylbenzene **2a** (199 μL, 1.5 mmol), Cs_2CO_3 (163 mg, 0.5 mmol), DABCO (11 mg, 0.1 mmol), **I** (10 mg, 0.025 mmol), $^{n}Bu_4NBF_4$ (329 mg, 1 mmol) and D_2O (27 μL, 1.5 mmol) were added to the solvent DMF (10 mL). The resulting mixture

was allowed to stir and electrolyze at constant current condition (18 mA, J = 6.7 mA·cm^{-2}) at 10 °C for 3 hours. Then the reaction mixture was poured into water (100 mL) and extracted with ethyl acetate (40 mL×3). Combined organic phase was washed with saturated brine solution (100 mL). The volatile solvent was then removed with a rotary evaporator, and the residue was purified by column chromatography (PE/EA = 8/1-5/1, v/v) on silica gel to afford the desired product **3a** in 40% yield. Allenol product **5a** was isolated in 25% yield. ^{1}H NMR spectra of product **3a** and **5a** were listed below.

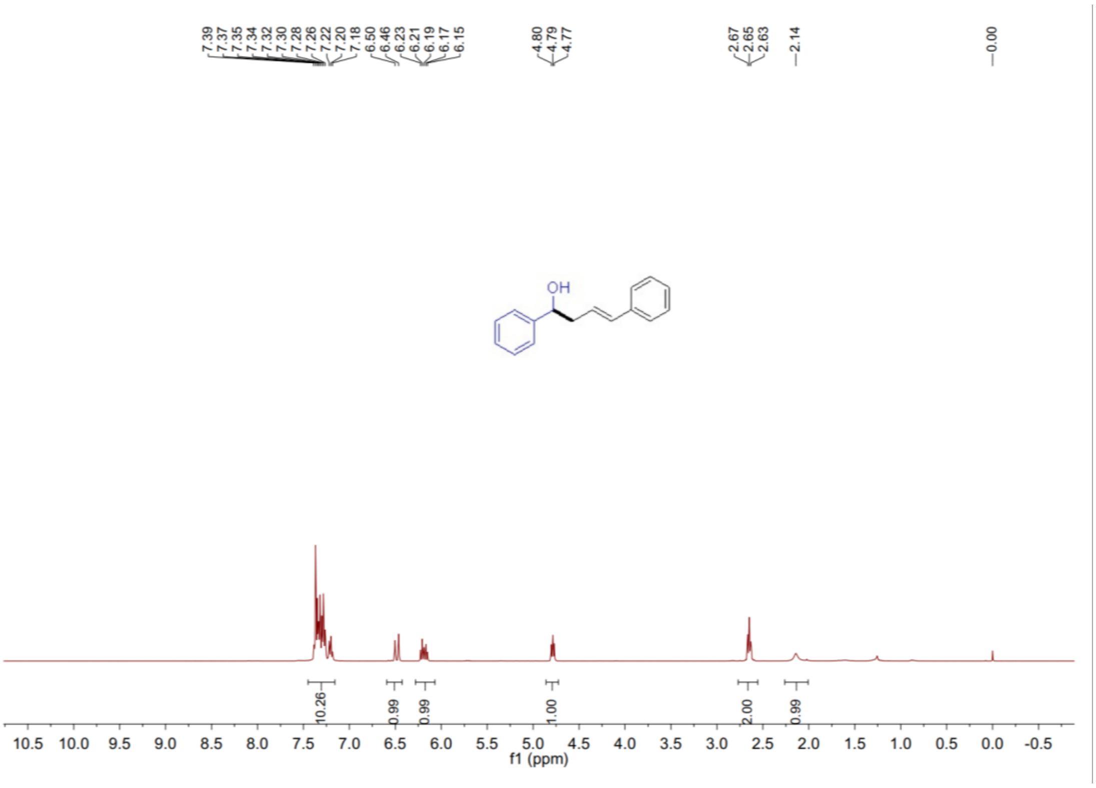

Figure S26 ^{1}H NMR spectrum of **3a**

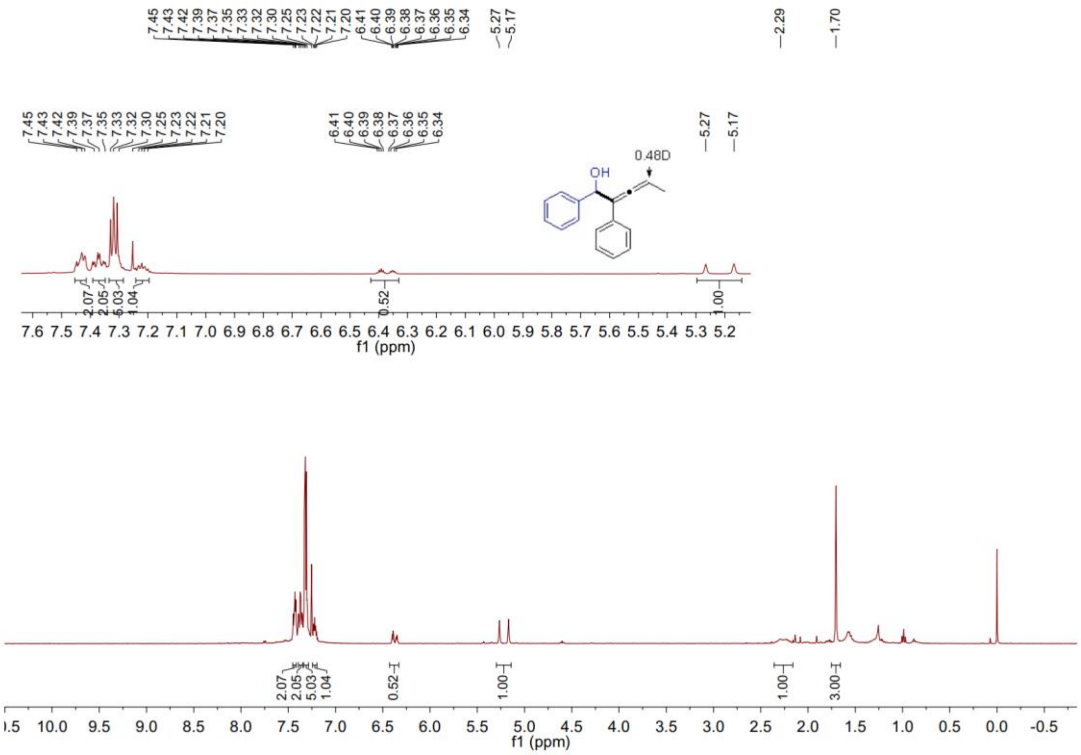

Figure S27 ^{1}H NMR spectrum of **5a**

As shown in the above spectra, substantial amount of deuterium atom was incorporated into the product **5a**, while no deuterium atom was detected in the product **3a**. This result cannot serve as evidence to exclude allylic carbanions, since D_2O is preferential to proceed HER to deliver deuterium gas or transfer deuterium to cathode surface (or Co^{II}-salen) before the HER process of **1a** or **4a**. The allylic carbanions showed high reactivity that would immediately react with benzaldehyde after generating from the cathode. In contrast, propargylic carbanions with lower reactivity might exchange deuterium over cathode surface with a reversible HER process, thus furnishing a deuterium product.

Deuterium labeling experiment with 4a-*d*$_5$

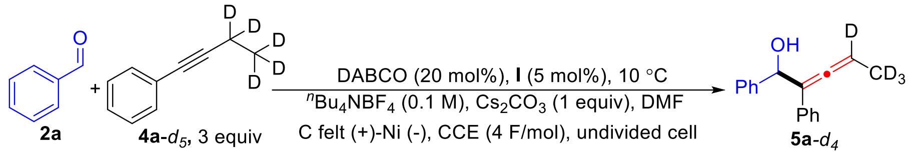

An undivided cell was equipped with a magnet stirrer, nickel plate (1.8 *1.5 cm^2), graphite felt (1.8 *1.5 cm^2), as cathode and anode, respectively (the electrolysis setup is shown in Figure S21). The substrate benzaldehyde (52 µL, 0.5 mmol), **4a-*d*$_5$** (215 µL, 1.5 mmol), Cs_2CO_3 (163 mg, 0.5 mmol), DABCO (11 mg, 0.1 mmol), **I** (10 mg, 0.025 mmol), and ^{n}Bu$_4$NBF$_4$ (329 mg, 1 mmol) were added to the solvent DMF (10 mL). The resulting mixture was allowed to stir and electrolyze at constant current condition (18 mA, J = 6.7 mA·cm^{-2}) at 10 °C for 3 hours. Then the reaction mixture was poured into water (100 mL) and extracted with ethyl acetate (40 mL×3). Combined organic phase was washed with saturated brine solution (100 mL). The volatile solvent was then removed with a rotary evaporator, and the residue was purified by column chromatography (PE/EA = 12/1-8/1, v/v) on silica gel to afford the desired product in 33% yield. ^{1}H NMR spectra of product **5a-*d*$_4$** were listed below. It showed that deuterium at allene carbon is significantly decreased. It also partially supports the carbanions generating in the reaction.

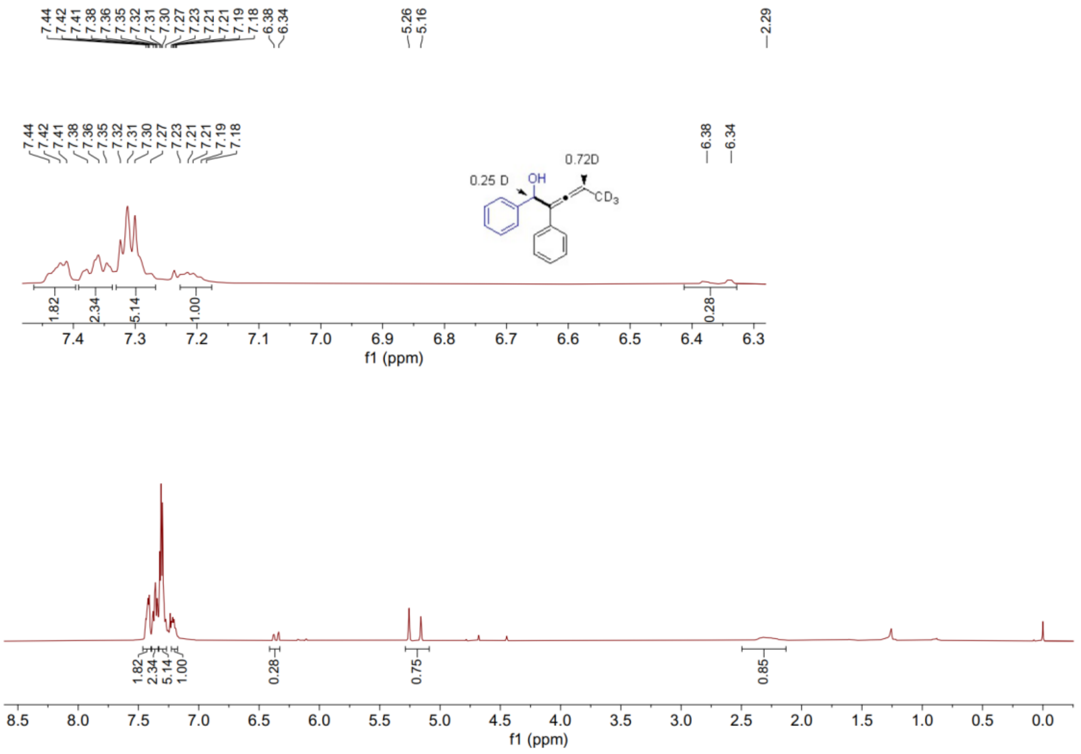

Figure S28 ^{1}H NMR spectrum of **5a**-*d*$_4$

KIE study

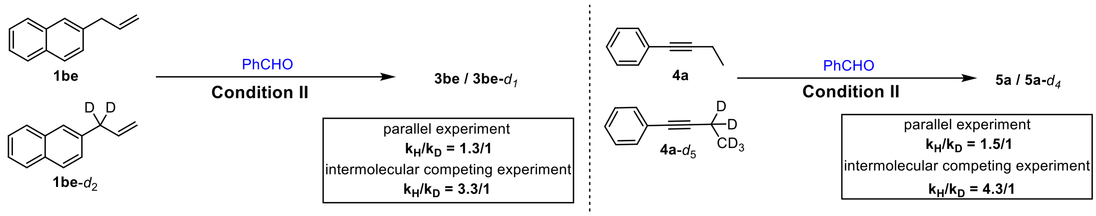

We tested KIE in the electrochemical allylation or allenylation by parallel and intermolecular competing experiment. Interestingly, the parallel experiment revealed a secondary KIE (k_H/k_D = 1.3/1-1.5/1) in the reaction further supporting the proton transfer in the HER process. In contrast, the intermolecular competing experiment gives a significantly higher value of k_H/k_D (3.3/1-4.3/1). The discrepancy of the KIE results can be explained with the rapid hydrogen/deuterium (H/D) exchange of the carbanions generating in the reaction. The results are shown below.

Parallel experiment

Since water has a detrimental effect on the reaction efficiency and rate, we conducted the parallel experiment with anhydrous DMF. The result of the parallel experiment is shown below.

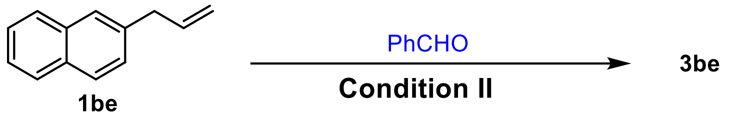

Reaction time	0 min	10 min	20 min	30 min	45 min	60 min
Reaction yield	0%	12%	29%	47%	59%	78%

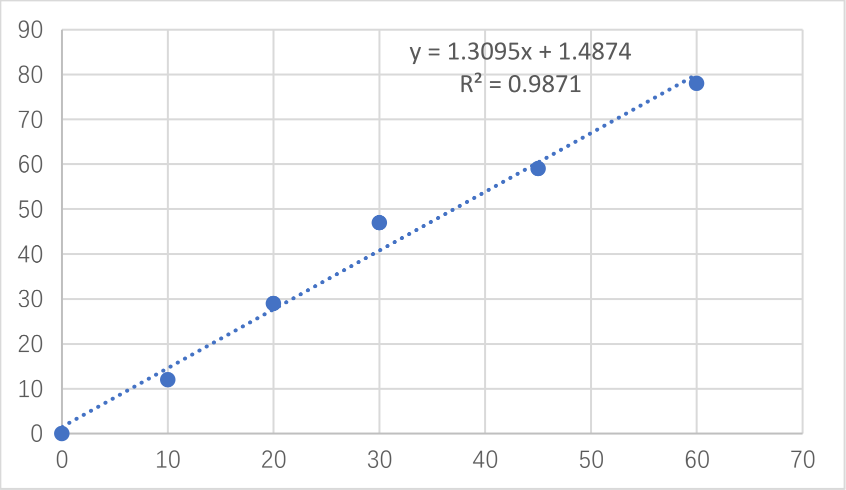

Figure S29 Reaction rate testing for **3be**

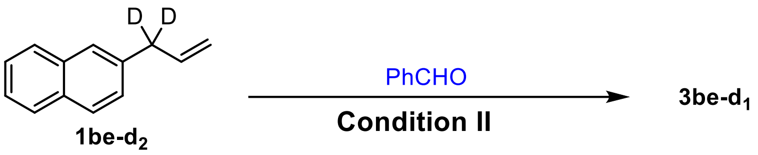

Reaction time	0 min	10 min	20 min	30 min	45 min	60 min
Reaction yield	0%	11%	23%	36%	48%	61%

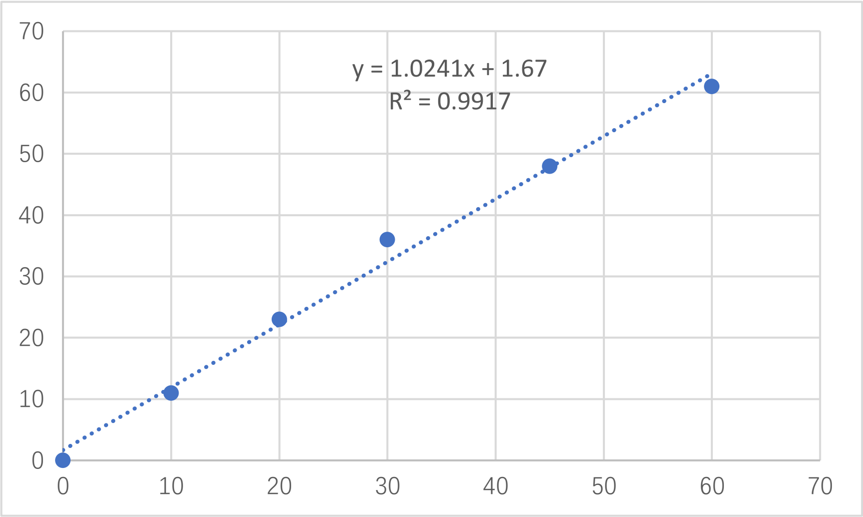

Figure S30 Reaction rate testing for **3be-d$_1$**

The KIE is determined to 1.3/1 (k_H/k_D = 1.3095/1.0241) in the electrochemical allylation.

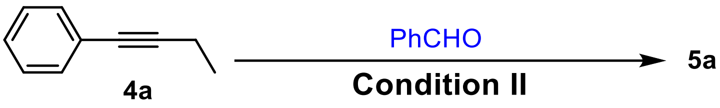

Reaction time	0 min	20 min	40 min	60 min	80 min	100 min
Reaction yield	0%	7%	16%	24%	29%	39%

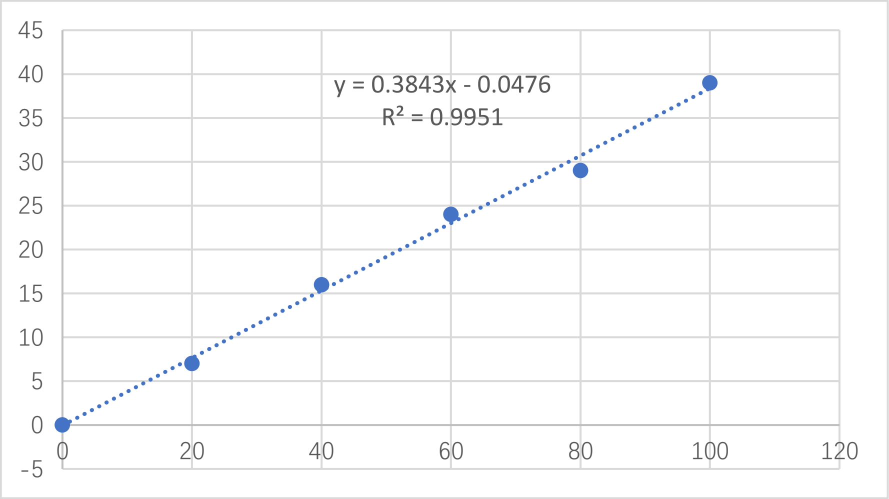

Figure S31 Reaction rate testing for **5a**

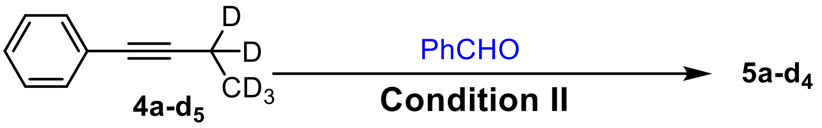

Reaction time	0 min	20 min	40 min	60 min	80 min	100 min
Reaction yield	0%	5%	12%	16%	21%	25%

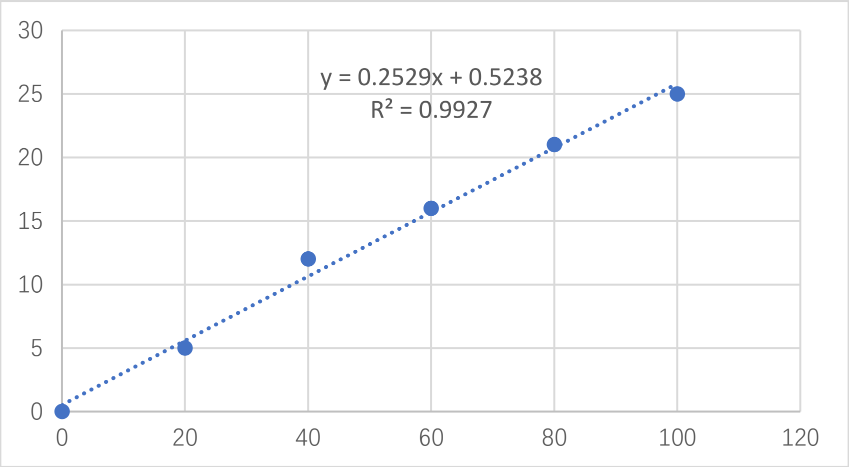

Figure S32 Reaction rate testing for **5a-d₄**

The KIE is determined to 1.5/1 (k_H/k_D = 0.3843/0.2529) in the electrochemical allenylation.

Intermolecular competing experiment

we conducted the intermolecular competing experiment using mixed **1be/1be-d$_1$** and **4a/4a-d$_5$** (1/1) as substrate.

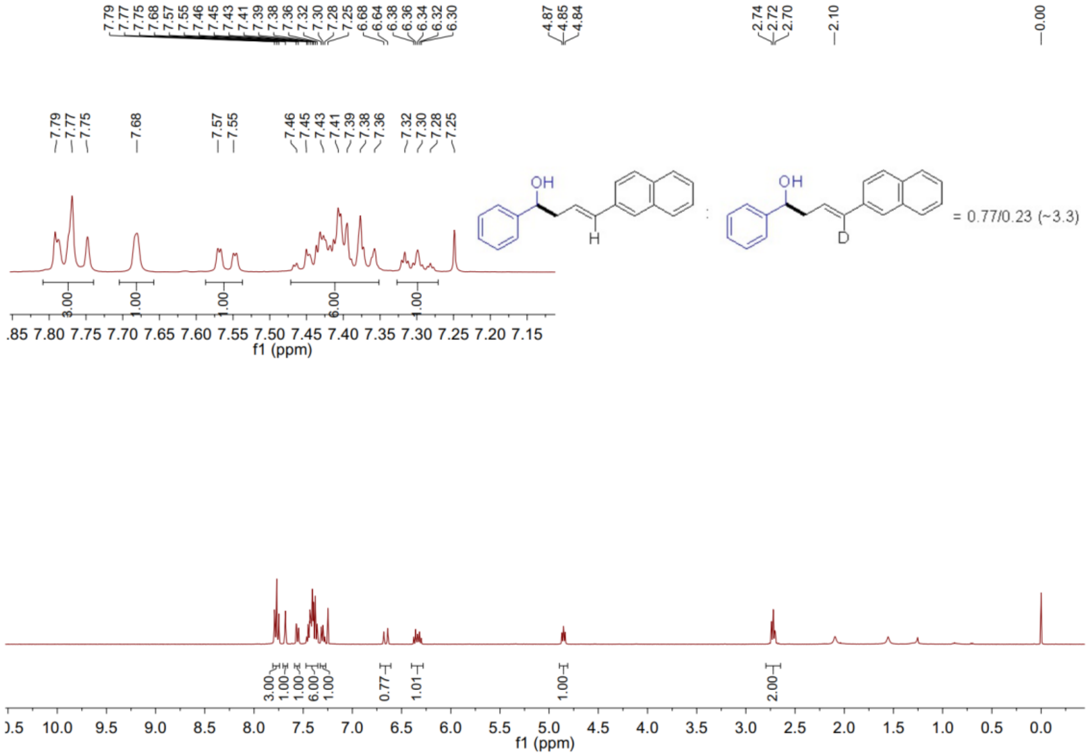

Figure S33 ^{1}H NMR spectrum of mixture of **3be/3be-*d$_1$***

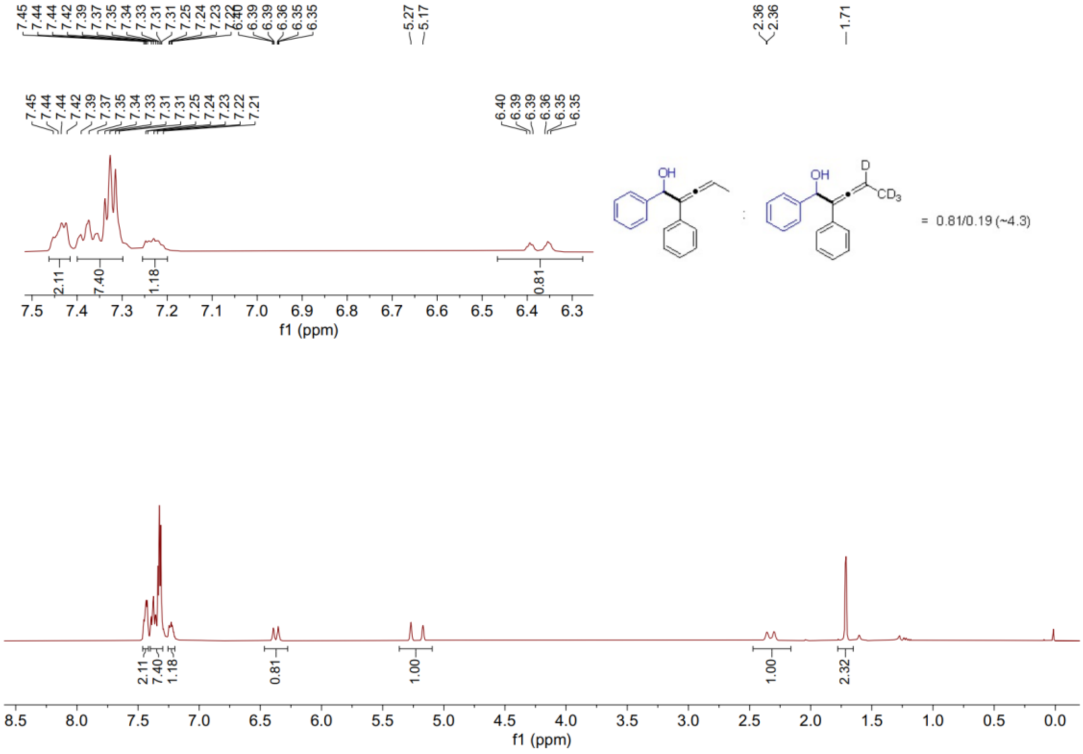

Figure S34 ^{1}H NMR spectrum of mixture of **5a**/**5a**-*d$_4$*

Electrode potential monitoring during the reaction

We monitored the electrode potential with Ag/$AgNO_3$ (0.1 M in CH_3CN) as reference electrode under the method of chronopotentiometry using CHI 760E potentiostat (CCE 18mA).

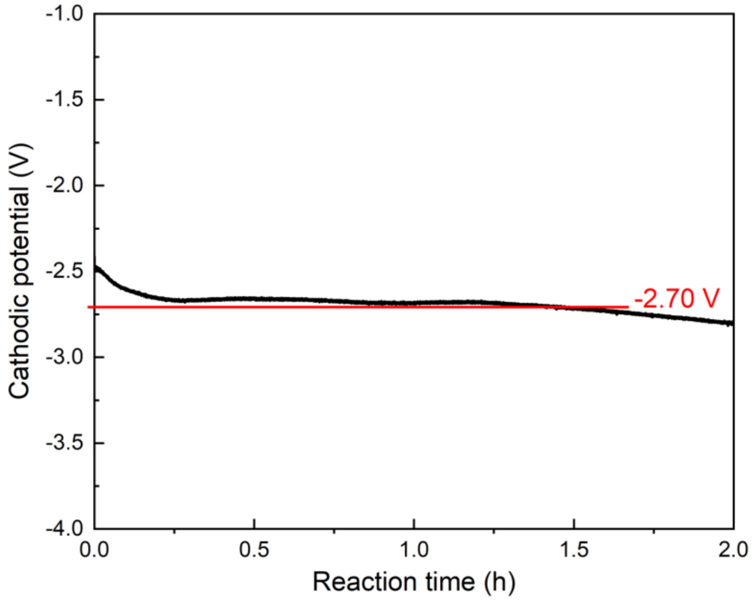

Figure S35 Cathode potential during the reaction between **1a** and **2a** **(Condition II)**

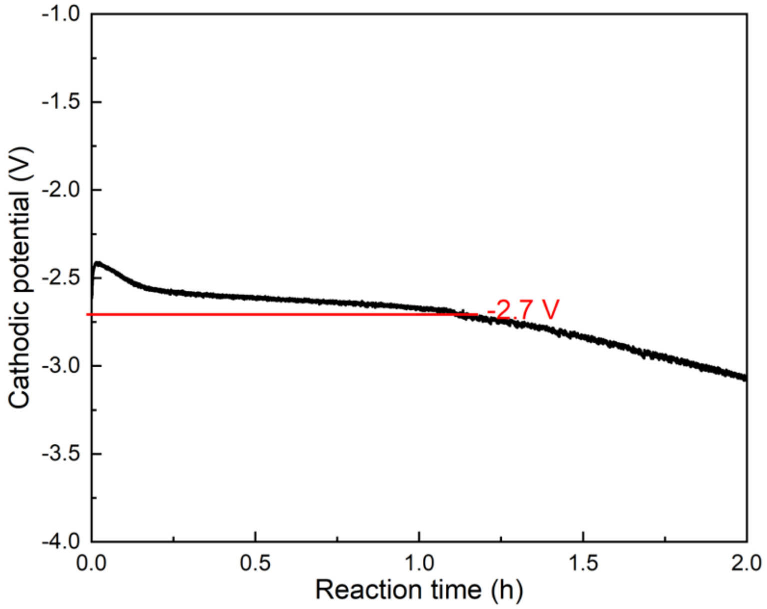

Figure S36 Cathode potential during the reaction between **2a** and **4a** **(Condition II)**

As shown in the above figures, the cathode potential is more positive than that of benzaldehyde (-2.7 V) in both the electrochemical allylation and allenylation. This result indicates that electrochemical allylation should be initiated by the HER of the excessive allylbenzene rather than the reduction of aldehyde.

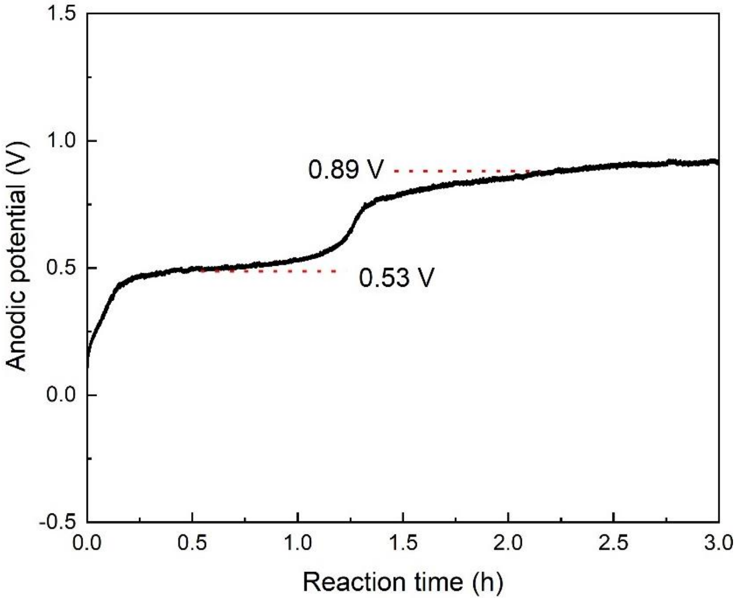

Figure S37 Anode potential during the reaction between **1a** and **2a (Condition II)**

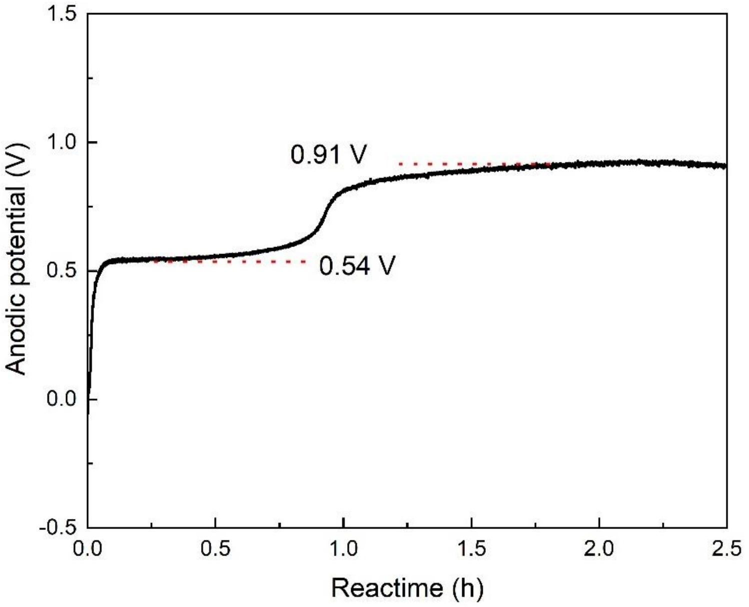

Figure S38 Anode potential during the reaction between**2a** and **4a (Condition II)**

As shown in the above figures, the anode potentials in both reactions are similar. We assigned the anode potentials to the oxidation of DABCO (0.53-0.54 V) and the oxidation of allylic or propargylic carbanions (0.89-0.91 V).

Constant potential electrolysis experiment

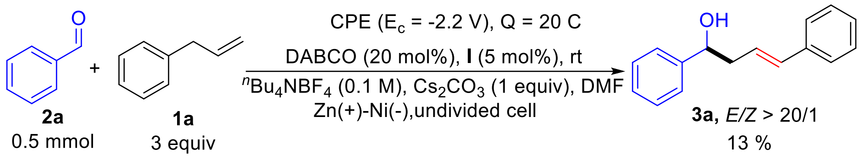

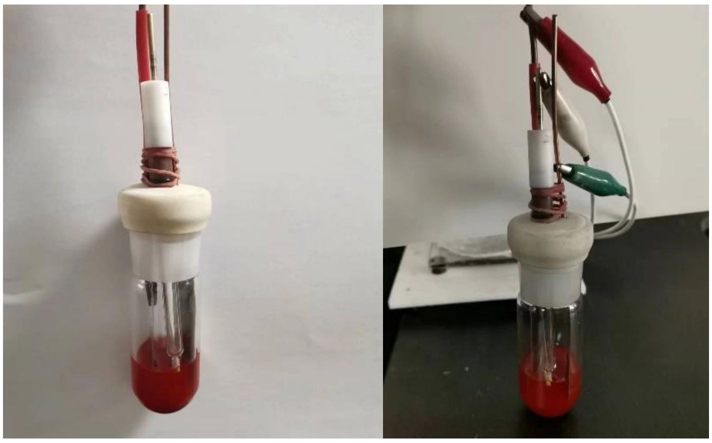

Figure S39 Setup of CPE electrolysis

To further clarify the cathodic reaction in the transformation, constant potential electrolysis experiment was conducted at -2.2 V (vs Ag/Ag^+), that only enables the HER of allylbenzene. As expected, product **3a** was detected in 13% yield under the method of i-t using CHI 760E potentiostat (Q = 20 C). This result further verifies that the reaction is initiated by the HER of allylbenzene.

9. Photophysical properties of 14a and 14b

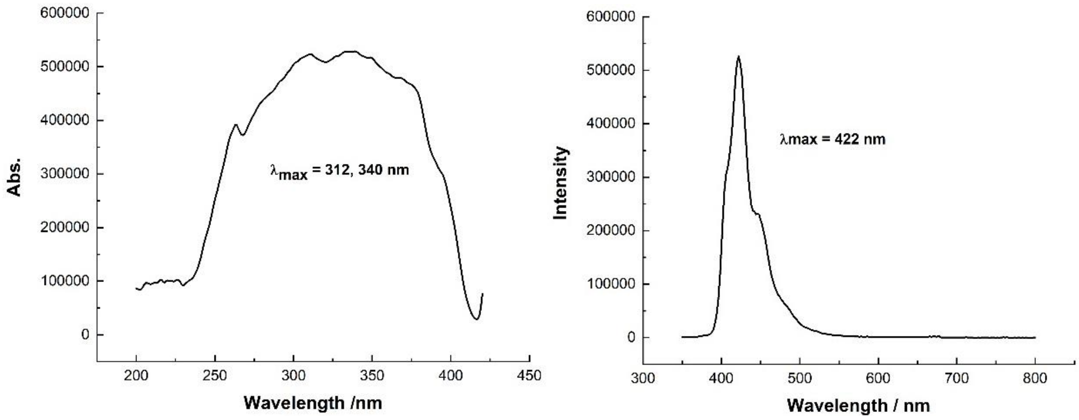

Figure S40 a) Photoluminescence absorption spectrum of **14a** in solid powders b) Photoluminescence emission spectrum of **14a** in solid powders upon excitation at 334 nm

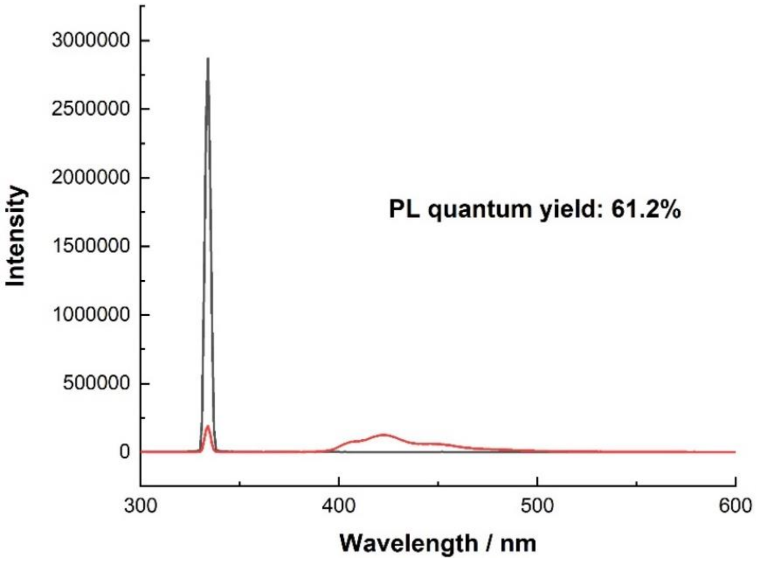

Figure S41 a) Photoluminescence quantum yield of **14a** in solid powders upon excitation at 300-382 nm

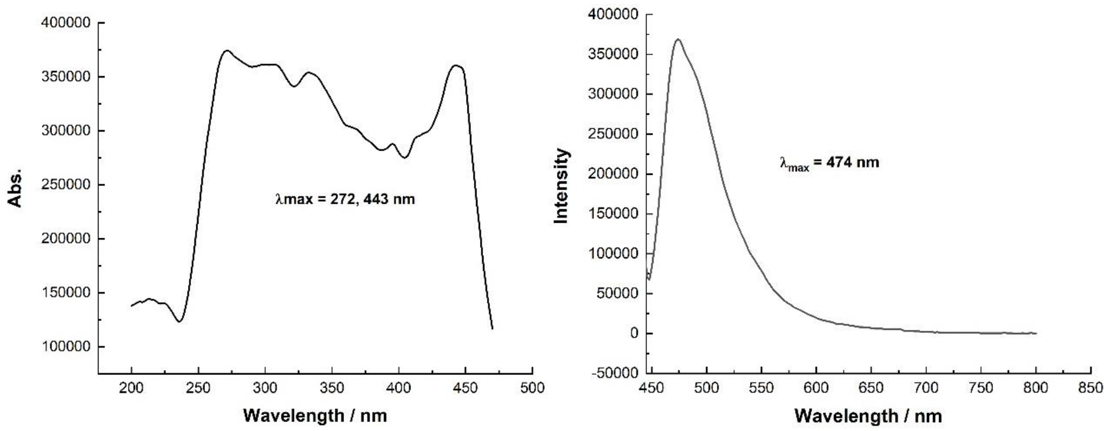

Figure S42 a) Photoluminescence absorption spectrum of **14b** in solid powders b) Photoluminescence emission spectrum of **14b** in solid powders upon excitation at 442 nm

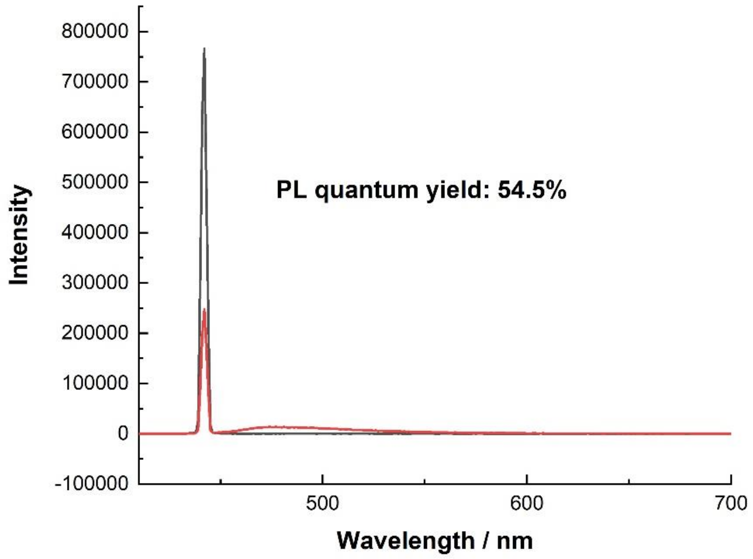

Figure S43 a) Photoluminescence quantum yield of **14b** in solid powders upon excitation at 410-447 nm

10. Details of DFT calculation

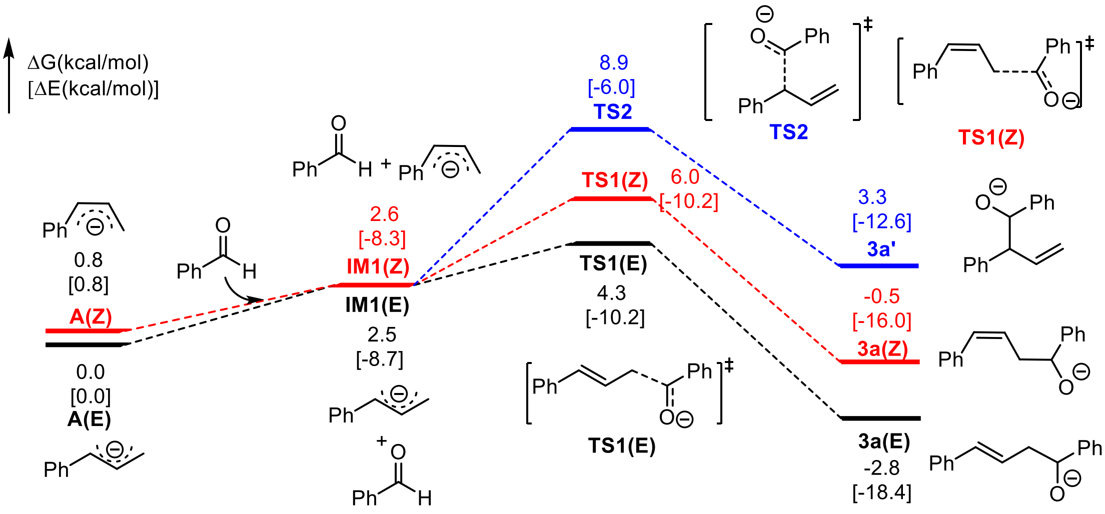

Figure S44 Computed free energy profile (in kcal/mol) for the electrochemical allylation at the PBE0 +D3(BJ)/ma-def2-TZVPP (SMD, solvent = n,n-DiMethylFormamide)//B3LYP + D3(BJ)/Def2-SVP level.

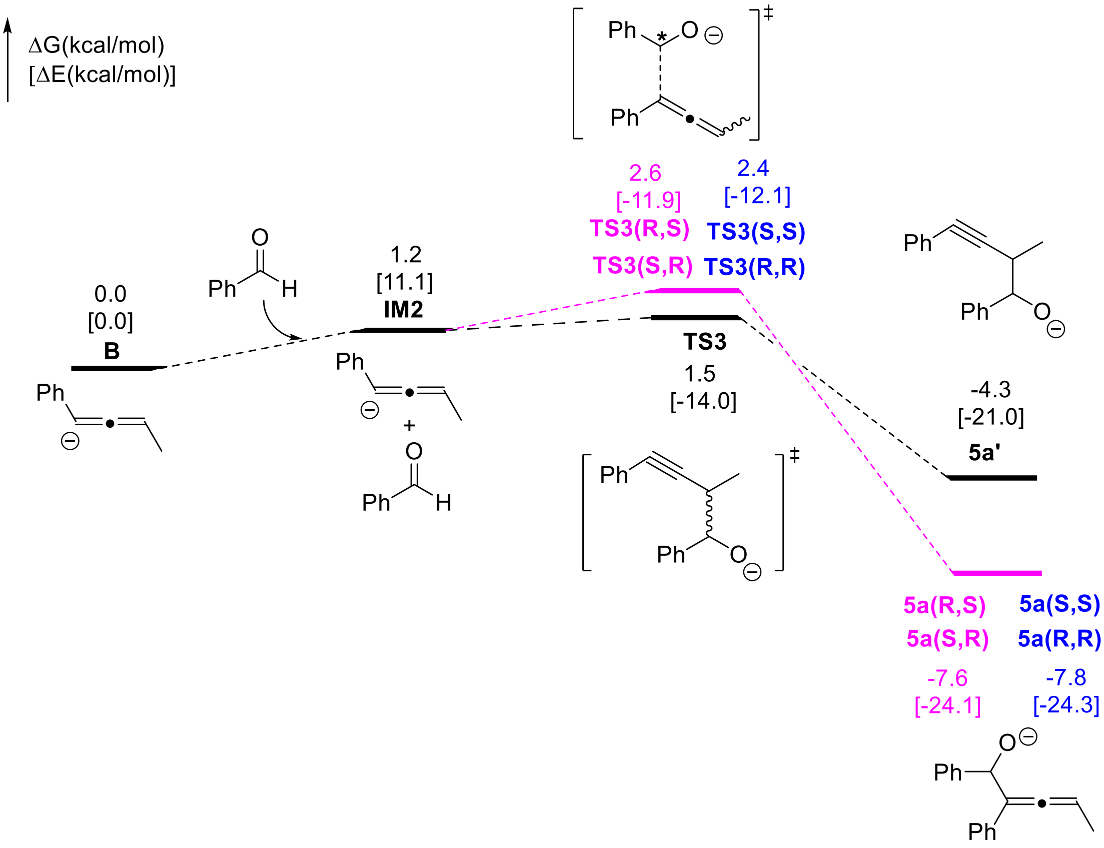

Figure S45 Computed free energy profile (in kcal/mol) for the electrochemical allenylation at the PBE0 +D3(BJ)/ma-def2-TZVPP (SMD, solvent = n,n-DiMethylFormamide)//B3LYP + D3(BJ)/Def2-SVP level.

3a(E)

Cartesian coordinates

ATOM	X	Y	Z
C	-3.21431	-0.39107	-0.14207
C	-4.23530	-1.36616	-0.13693
C	-5.57843	-1.02226	0.02381
C	-5.95035	0.31534	0.18591
C	-4.95170	1.29888	0.18675
C	-3.61170	0.95550	0.02819
H	-3.95628	-2.41646	-0.26414
H	-6.34168	-1.80650	0.02274
H	-7.00102	0.58981	0.31286
H	-5.22373	2.35055	0.31767
H	-2.84784	1.73499	0.04082
C	-1.81886	-0.79650	-0.32271
H	-1.65667	-1.87992	-0.39436
C	-0.75106	0.02448	-0.41761
H	-0.85221	1.11477	-0.34125
C	0.66739	-0.36819	-0.53561
H	0.81239	-1.45796	-0.41929
H	1.05506	-0.06068	-1.52383
C	1.53577	0.53647	0.49224
H	1.27091	0.08038	1.50909
C	3.01537	0.12136	0.27798
C	3.52692	-1.15121	0.56759
C	3.88610	1.09196	-0.23075
C	4.87394	-1.45306	0.34314
H	2.85885	-1.91373	0.98424
C	5.23332	0.79888	-0.45910
H	3.42767	2.06930	-0.41154
C	5.73506	-0.47703	-0.17487
H	5.25879	-2.45210	0.57550
H	5.90428	1.56877	-0.85667
H	6.79083	-0.70927	-0.34792
O	1.29097	1.81048	0.33505

B3LYP+D3(BJ)/Def2-SVP Gibbs free energy:-693.300727 a.u.

B3LYP+D3(BJ)/Def2-SVP enthalpy:-693.241236 a.u.

B3LYP+D3(BJ)/Def2-SVP SCF energy:-693.517216 a.u.

PBE0+D3(BJ)/Def2-TZVPP SOLVENT=(DMF) Gibbs free energy:-693.286821 a.u.

PBE0+D3(BJ)/Def2-TZVPP SOLVENT=(DMF) enthalpy:-693.227330 a.u.

PBE0+D3(BJ)/Def2-TZVPP SOLVENT=(DMF) SCF energy:-693.503310 a.u.

Lowest frequency: 26.8546 cm-1

3a’

Cartesian coordinates

ATOM	X	Y	Z
C	-1.91741	-0.20215	-0.06399
C	-2.73015	-1.27750	-0.45769
C	-4.09462	-1.10186	-0.70882
C	-4.67424	0.16263	-0.57105
C	-3.87097	1.24369	-0.18705
C	-2.51027	1.06841	0.07144
H	-2.28141	-2.26995	-0.56632
H	-4.70681	-1.95643	-1.01451
H	-5.74214	0.30490	-0.76418
H	-4.31411	2.24034	-0.08986
H	-1.82739	1.88575	0.33639
C	-0.44168	-0.40957	0.21405
C	-0.18077	-0.49171	1.68835
H	-0.17017	0.49640	2.16336
C	0.06010	-1.60556	2.38788
H	0.07161	-2.59285	1.91088
H	0.27111	-1.57458	3.46242
H	-0.14445	-1.36128	-0.25891
C	0.42651	0.82726	-0.37226
H	0.10698	0.84639	-1.46699
O	0.24094	1.94394	0.30336
C	1.90268	0.35716	-0.42826
C	2.35218	-0.72582	-1.19686
C	2.84046	1.07814	0.31919
C	3.70161	-1.09138	-1.20697
H	1.63385	-1.28712	-1.80471
C	4.19132	0.71984	0.31544
H	2.43286	1.92536	0.87827
C	4.62976	-0.36957	-0.44596
H	4.03491	-1.94139	-1.81213
H	4.91334	1.29338	0.90731
H	5.68724	-0.65254	-0.45257

B3LYP+D3(BJ)/Def2-SVP Gibbs free energy:-693.297852 a.u.

B3LYP+D3(BJ)/Def2-SVP enthalpy:-693.239066 a.u.

B3LYP+D3(BJ)/Def2-SVP SCF energy:-693.514890 a.u.

PBE0+D3(BJ)/Def2-TZVPP SOLVENT=(DMF) Gibbs free energy:-693.277071 a.u.

PBE0+D3(BJ)/Def2-TZVPP SOLVENT=(DMF) enthalpy:-693.218285 a.u.

PBE0+D3(BJ)/Def2-TZVPP SOLVENT=(DMF) SCF energy:-693.494109 a.u.

Lowest frequency: 34.4607 cm-1

3a(Z)

Cartesian coordinates

ATOM	X	Y	Z
C	2.88874	-0.53408	-0.03793
C	4.25025	-0.50075	-0.41148
C	4.99965	0.67393	-0.34898
C	4.41219	1.86142	0.10104
C	3.07171	1.84487	0.50113
C	2.32336	0.66855	0.44264
H	4.71789	-1.42338	-0.76824
H	6.05033	0.66432	-0.65477
H	4.99576	2.78475	0.15221
H	2.60383	2.75856	0.87928
H	1.29573	0.66178	0.80589
C	2.14475	-1.79772	-0.11806
H	2.76953	-2.69211	0.00071
C	0.82590	-1.99713	-0.35214
H	0.46130	-3.02936	-0.31495
C	-0.26809	-1.03537	-0.60220
H	0.08074	-0.00392	-0.75841
H	-0.82415	-1.35902	-1.49908
C	-1.37721	-1.15978	0.58312
H	-0.82654	-0.68700	1.47375
C	-2.47403	-0.11244	0.23960
C	-2.26354	1.27287	0.20677
C	-3.75476	-0.60057	-0.04262
C	-3.30394	2.15143	-0.11227
H	-1.26971	1.67033	0.44314
C	-4.80050	0.26933	-0.36315
H	-3.85395	-1.68872	0.02516
C	-4.58029	1.65160	-0.40156
H	-3.12322	3.23168	-0.13327
H	-5.79911	-0.12638	-0.58066
H	-5.39759	2.33701	-0.64814
O	-1.80576	-2.37942	0.73425

B3LYP+D3(BJ)/Def2-SVP Gibbs free energy:-693.295122 a.u.

B3LYP+D3(BJ)/Def2-SVP enthalpy:-693.235461 a.u.

B3LYP+D3(BJ)/Def2-SVP SCF energy:-693.511562 a.u.

PBE0+D3(BJ)/Def2-TZVPP SOLVENT=(DMF) Gibbs free energy:-693.283138 a.u.

PBE0+D3(BJ)/Def2-TZVPP SOLVENT=(DMF) enthalpy:-693.223477 a.u.

PBE0+D3(BJ)/Def2-TZVPP SOLVENT=(DMF) SCF energy:-693.499578 a.u.

Lowest frequency: 18.5846 cm-1

A(E)

Cartesian coordinates

ATOM	X	Y	Z
C	0.04864	-0.25893	0.00003
C	-0.95605	-1.28975	-0.00001
C	-2.31619	-1.01515	-0.00003
C	-2.80070	0.30586	-0.00002
C	-1.84597	1.33894	0.00002
C	-0.48185	1.07990	0.00004
H	-0.61847	-2.33229	-0.00002
H	-3.02591	-1.85202	-0.00006
H	-3.87294	0.52048	-0.00003
H	-2.18270	2.38337	0.00003
H	0.21246	1.92433	0.00006
C	1.42663	-0.58640	0.00005
H	1.67025	-1.65741	0.00016
C	2.51636	0.32045	-0.00006
H	2.24503	1.38727	-0.00016
C	3.86312	0.05939	-0.00004
H	4.24811	-0.96757	0.00017
H	4.60020	0.86794	0.00001

B3LYP+D3(BJ)/Def2-SVP Gibbs free energy:-348.031113 a.u.

B3LYP+D3(BJ)/Def2-SVP enthalpy:-347.989604 a.u.

B3LYP+D3(BJ)/Def2-SVP SCF energy:-348.144788 a.u.

PBE0+D3(BJ)/Def2-TZVPP SOLVENT=(DMF) Gibbs free energy:-348.048820 a.u.

PBE0+D3(BJ)/Def2-TZVPP SOLVENT=(DMF) enthalpy:-348.007311 a.u.

PBE0+D3(BJ)/Def2-TZVPP SOLVENT=(DMF) SCF energy:-348.162495 a.u.

Lowest frequency: 96.3595 cm-1

A(Z)

Cartesian coordinates

ATOM	X	Y	Z
C	0.09264	-0.45290	-0.01642
C	-1.06922	-1.30087	0.01712
C	-2.36403	-0.80539	0.03475
C	-2.61731	0.57981	0.01604
C	-1.50653	1.43664	-0.02844
C	-0.20119	0.95431	-0.04371
H	-0.90963	-2.38514	0.03200
H	-3.20423	-1.51038	0.06421
H	-3.63849	0.97130	0.03300
H	-1.66484	2.52211	-0.05465
H	0.62113	1.66446	-0.10007

C 1.39064 -1.02306 -0.03218
H 1.40615 -2.11980 -0.05667
C 2.67503 -0.40930 -0.01880
H 3.48552 -1.15559 -0.08325
C 3.13393 0.88385 0.06196
H 2.48858 1.75720 0.16597
H 4.21202 1.07726 0.05745
B3LYP+D3(BJ)/Def2-SVP Gibbs free energy:-348.027560 a.u.
B3LYP+D3(BJ)/Def2-SVP enthalpy:-347.985651 a.u.
B3LYP+D3(BJ)/Def2-SVP SCF energy:-348.141173 a.u.
PBE0+D3(BJ)/Def2-TZVPP SOLVENT=(DMF) Gibbs free energy:-348.047653 a.u.
PBE0+D3(BJ)/Def2-TZVPP SOLVENT=(DMF) enthalpy:-348.005744 a.u.
PBE0+D3(BJ)/Def2-TZVPP SOLVENT=(DMF) SCF energy:-348.161266 a.u.
Lowest frequency: 44.6386 cm-1

TS1(E)

Cartesian coordinates

ATOM X Y Z
C -2.65900 -0.46045 0.14055
C -2.69059 0.89242 0.58099
C -3.82619 1.68430 0.43418
C -4.99164 1.17907 -0.16159
C -4.98584 -0.14846 -0.60950
C -3.85210 -0.94497 -0.46225
H -1.79890 1.32523 1.04041
H -3.80256 2.72076 0.78756
H -5.87982 1.80664 -0.27633
H -5.88031 -0.56911 -1.08146
H -3.87066 -1.97993 -0.81846
C -1.51207 -1.33520 0.27895
H -1.63527 -2.33961 -0.14587
C -0.31462 -1.05337 0.89742
H -0.20083 -0.05631 1.34439
C 0.84837 -1.88202 0.92609
H 1.55201 -1.70682 1.74718
H 0.69668 -2.94724 0.71674
C 2.12333 -1.63545 -0.56938
H 1.31409 -1.67180 -1.35172
C 2.58480 -0.19770 -0.35902
C 1.81301 0.91567 -0.72940
C 3.85793 0.02419 0.18839
C 2.29099 2.21508 -0.53751
H 0.82529 0.75382 -1.17256

C 4.33779 1.32125 0.38785
H 4.44645 -0.86510 0.43009
C 3.55616 2.42577 0.02720
H 1.67548 3.07122 -0.83305
H 5.33292 1.47661 0.81916
H 3.93158 3.44313 0.17658
O 2.97648 -2.55778 -0.49755
B3LYP+D3(BJ)/Def2-SVP Gibbs free energy:-693.235713 a.u.
B3LYP+D3(BJ)/Def2-SVP enthalpy:-693.176229 a.u.
B3LYP+D3(BJ)/Def2-SVP SCF energy:-693.450538 a.u.
PBE0+D3(BJ)/Def2-TZVPP SOLVENT=(DMF) Gibbs free energy:-693.275440 a.u.
PBE0+D3(BJ)/Def2-TZVPP SOLVENT=(DMF) enthalpy:-693.215956 a.u.
PBE0+D3(BJ)/Def2-TZVPP SOLVENT=(DMF) SCF energy:-693.490265 a.u.
Lowest frequency: -189.8326 cm-1

IM1(E)

Cartesian coordinates
ATOM X Y Z
C -2.59845 -0.55356 0.15449
C -2.72649 0.72858 0.78280
C -3.81839 1.55838 0.55346
C -4.86025 1.18296 -0.31137
C -4.76432 -0.06819 -0.94254
C -3.67749 -0.90590 -0.71971
H -1.94152 1.06889 1.46310
H -3.86133 2.52860 1.06240
H -5.71492 1.84215 -0.48711
H -5.55839 -0.39520 -1.62417
H -3.63382 -1.87572 -1.22706
C -1.50414 -1.45243 0.35233
H -1.56157 -2.40208 -0.19567
C -0.38766 -1.26454 1.18872
H -0.34938 -0.31559 1.74261
C 0.69402 -2.10263 1.37439
H 1.47825 -1.86083 2.09480
H 0.73702 -3.09391 0.91338
C 2.45560 -1.58246 -0.76303
H 1.45404 -1.81337 -1.19165
C 2.66574 -0.14507 -0.43499
C 1.64949 0.80434 -0.64716
C 3.92139 0.28900 0.02940
C 1.88125 2.15537 -0.38806
H 0.66852 0.46625 -0.99281

C	4.15156	1.64019	0.28792
H	4.69829	-0.46559	0.17508
C	3.13298	2.58012	0.08033
H	1.07946	2.88254	-0.54547
H	5.13056	1.96863	0.65153
H	3.31320	3.64029	0.28208
O	3.34657	-2.42001	-0.71287

B3LYP+D3(BJ)/Def2-SVP Gibbs free energy:-693.244836 a.u.

B3LYP+D3(BJ)/Def2-SVP enthalpy:-693.179985 a.u.

B3LYP+D3(BJ)/Def2-SVP SCF energy:-693.454516 a.u.

PBE0+D3(BJ)/Def2-TZVPP SOLVENT=(DMF) Gibbs free energy:-693.278242 a.u.

PBE0+D3(BJ)/Def2-TZVPP SOLVENT=(DMF) enthalpy:-693.213391 a.u.

PBE0+D3(BJ)/Def2-TZVPP SOLVENT=(DMF) SCF energy:-693.487922 a.u.

Lowest frequency: 7.8231 cm-1

TS2

Cartesian coordinates

ATOM	X	Y	Z
C	-1.89659	-0.15140	0.44420
C	-2.48562	-1.43851	0.51062
C	-3.79512	-1.67908	0.09497
C	-4.58012	-0.63543	-0.41168
C	-4.01771	0.64374	-0.49315
C	-2.70506	0.88405	-0.08185
H	-1.88943	-2.26556	0.91045
H	-4.20958	-2.69030	0.17148
H	-5.60862	-0.81683	-0.73856
H	-4.61165	1.47329	-0.89202
H	-2.29753	1.88960	-0.15706
C	-0.48541	0.02306	0.82907
C	0.00377	1.25193	1.47785
H	0.57873	1.08504	2.40109
C	-0.08016	2.52567	1.04051
H	-0.52478	2.77426	0.07662
H	0.38878	3.33770	1.60753
H	-0.13728	-0.86843	1.36957
C	0.41642	-0.04735	-0.90564
H	-0.04957	-1.03826	-1.17482
O	0.14713	0.97510	-1.59593
C	1.86962	-0.27928	-0.49562
C	2.79213	0.76346	-0.67148
C	2.33541	-1.50307	0.01092
C	4.13582	0.59766	-0.32506

H 2.40126 1.68927 -1.10043
C 3.67913 -1.67706 0.35434
H 1.62856 -2.33191 0.13133
C 4.58786 -0.62272 0.19219
H 4.84260 1.42327 -0.46420
H 4.02403 -2.64005 0.74641
H 5.64129 -0.75592 0.45892

B3LYP+D3(BJ)/Def2-SVP Gibbs free energy:-693.222540 a.u.

B3LYP+D3(BJ)/Def2-SVP enthalpy:-693.164083 a.u.

B3LYP+D3(BJ)/Def2-SVP SCF energy:-693.438107 a.u.

PBE0+D3(BJ)/Def2-TZVPP SOLVENT=(DMF) Gibbs free energy:-693.268065 a.u.

PBE0+D3(BJ)/Def2-TZVPP SOLVENT=(DMF) enthalpy:-693.209608 a.u.

PBE0+D3(BJ)/Def2-TZVPP SOLVENT=(DMF) SCF energy:-693.483632 a.u.

Lowest frequency: -234.8355 cm-1

TS1(Z)

Cartesian coordinates

ATOM	X	Y	Z
C	1.97746	-0.81916	0.23643
C	3.32019	-0.65716	0.67192
C	4.17293	0.29248	0.11421
C	3.72190	1.13655	-0.90946
C	2.39663	1.01117	-1.34374
C	1.53884	0.06294	-0.78585
H	3.69131	-1.31099	1.46813
H	5.20182	0.37634	0.48037
H	4.38634	1.88594	-1.34947
H	2.01352	1.67686	-2.12360
H	0.50223	0.02949	-1.11567
C	1.16188	-1.85191	0.85770
H	1.62224	-2.28170	1.75630
C	-0.02184	-2.43944	0.46738
H	-0.34163	-3.24558	1.14636
C	-0.95626	-2.19169	-0.59751
H	-0.61831	-1.59244	-1.44950
H	-1.48826	-3.08888	-0.93109
C	-2.63321	-1.29965	-0.16503
H	-2.81529	-1.87247	0.79083
C	-2.17446	0.11230	0.17934
C	-1.57216	0.44770	1.40219
C	-2.40626	1.13410	-0.75561
C	-1.18812	1.76397	1.67365
H	-1.39826	-0.33773	2.14332

C -2.02004 2.44963 -0.49047
H -2.91142 0.84060 -1.67992
C -1.40435 2.77193 0.72606
H -0.71051 2.00690 2.62845
H -2.20467 3.23530 -1.23154
H -1.09957 3.80180 0.93699
O -3.41355 -1.45825 -1.14465
B3LYP+D3(BJ)/Def2-SVP Gibbs free energy:-693.226358 a.u.
B3LYP+D3(BJ)/Def2-SVP enthalpy:-693.167856 a.u.
B3LYP+D3(BJ)/Def2-SVP SCF energy:-693.442461 a.u.
PBE0+D3(BJ)/Def2-TZVPP SOLVENT=(DMF) Gibbs free energy:-693.272631 a.u.
PBE0+D3(BJ)/Def2-TZVPP SOLVENT=(DMF) enthalpy:-693.214129 a.u.
PBE0+D3(BJ)/Def2-TZVPP SOLVENT=(DMF) SCF energy:-693.488734 a.u.
Lowest frequency: -186.9740 cm-1

IM1(Z)

Cartesian coordinates
ATOM X Y Z
C 2.29238 0.61681 -0.45871
C 2.99200 -0.44234 -1.13486
C 3.67186 -1.44674 -0.45997
C 3.71397 -1.47981 0.94709
C 3.04902 -0.45768 1.63922
C 2.36223 0.55703 0.97470
H 2.97963 -0.44643 -2.23059
H 4.18386 -2.22585 -1.03763
H 4.24733 -2.27238 1.47955
H 3.06667 -0.44876 2.73569
H 1.87802 1.33327 1.56432
C 1.60022 1.60431 -1.21563
H 1.69580 1.49055 -2.30197
C 0.80433 2.70942 -0.80520
H 0.42370 3.28161 -1.66705
C 0.37945 3.20666 0.40779
H 0.65943 2.78984 1.37640
H -0.24437 4.10566 0.43342
C -3.27178 1.23701 0.35565
H -2.51510 2.05556 0.45760
C -2.68668 -0.09770 0.09381
C -1.29486 -0.23229 -0.05319
C -3.51808 -1.23215 0.00689
C -0.73742 -1.49635 -0.27969
H -0.65003 0.64977 -0.00271

C -2.96021 -2.48622 -0.21914
H -4.59605 -1.09375 0.12336
C -1.56746 -2.61728 -0.36198
H 0.34540 -1.58894 -0.39239
H -3.60188 -3.37017 -0.28604
H -1.13059 -3.60444 -0.54077
O -4.46445 1.46105 0.45851
B3LYP+D3(BJ)/Def2-SVP Gibbs free energy:-693.242826 a.u.
B3LYP+D3(BJ)/Def2-SVP enthalpy:-693.177181 a.u.
B3LYP+D3(BJ)/Def2-SVP SCF energy:-693.451943 a.u.
PBE0+D3(BJ)/Def2-TZVPP SOLVENT=(DMF) Gibbs free energy:-693.278174 a.u.
PBE0+D3(BJ)/Def2-TZVPP SOLVENT=(DMF) enthalpy:-693.212529 a.u.
PBE0+D3(BJ)/Def2-TZVPP SOLVENT=(DMF) SCF energy:-693.487291 a.u.
Lowest frequency: 15.2011 cm-1

5a(R,S)

Cartesian coordinates

ATOM	X	Y	Z
C	-1.03093	0.88027	0.04270
C	-0.55236	1.73286	1.05846
C	-0.56416	3.11682	0.87813
C	-1.06105	3.68333	-0.30138
C	-1.54096	2.84417	-1.31283
C	-1.52161	1.45871	-1.14316
H	-0.17694	1.23368	1.95886
H	-0.18359	3.76578	1.67351
H	-1.07272	4.76968	-0.43352
H	-1.92542	3.27201	-2.24416
H	-1.88176	0.80050	-1.93800
C	-0.98957	-0.58991	0.22069
C	-1.89508	-1.38053	-0.30610
C	-2.81159	-2.22314	-0.74095
H	-2.72404	-2.62986	-1.76078
C	-4.00169	-2.69205	0.06356
H	-4.95804	-2.43223	-0.42856
H	-3.99957	-3.79116	0.18955
H	-3.99806	-2.23716	1.06528
C	0.19398	-1.20160	1.11477
H	0.03261	-2.31939	0.99952
C	1.50439	-0.94083	0.31354
C	2.56529	-0.33006	0.98783
C	1.68152	-1.31018	-1.02691
C	3.78092	-0.08719	0.34074

H	2.36923	-0.06950	2.03191
C	2.89278	-1.07036	-1.68107
H	0.85131	-1.78382	-1.56061
C	3.95077	-0.45577	-0.99801
H	4.60415	0.39419	0.88013
H	3.01547	-1.35840	-2.73067
H	4.89962	-0.26476	-1.50939
O	0.19298	-0.74459	2.34336

B3LYP+D3(BJ)/Def2-SVP Gibbs free energy:-731.349009 a.u.
B3LYP+D3(BJ)/Def2-SVP enthalpy:-731.286715 a.u.
B3LYP+D3(BJ)/Def2-SVP SCF energy:-731.568207 a.u.
PBE0+D3(BJ)/Def2-TZVPP SOLVENT=(DMF) Gibbs free energy:-731.325492 a.u.
PBE0+D3(BJ)/Def2-TZVPP SOLVENT=(DMF) enthalpy:-731.263198 a.u.
PBE0+D3(BJ)/Def2-TZVPP SOLVENT=(DMF) SCF energy:-731.544690 a.u.
Lowest frequency: 27.4512 cm-1

5a(S,S)

Cartesian coordinates

ATOM	X	Y	Z
C	1.23321	0.75296	0.17745
C	1.95475	0.93598	-1.01758
C	2.24213	2.21565	-1.49537
C	1.80577	3.34347	-0.79156
C	1.08084	3.17138	0.39319
C	0.79999	1.89453	0.88219
H	2.28002	0.05371	-1.57484
H	2.80290	2.33401	-2.42796
H	2.02741	4.34768	-1.16599
H	0.73233	4.04824	0.94842
H	0.23736	1.69827	1.80191
C	0.90786	-0.60287	0.67818
C	1.71067	-1.62419	0.49306
C	2.48305	-2.68516	0.36629
H	3.18410	-2.93529	1.17816
C	2.48714	-3.61322	-0.82611
H	3.48134	-3.65808	-1.31015
H	2.23044	-4.64961	-0.53669
H	1.75758	-3.28464	-1.58214
C	-0.46274	-0.79197	1.49097
C	-1.59922	-0.57264	0.44730
C	-2.60691	0.34027	0.77033
C	-1.67752	-1.25816	-0.77274
C	-3.67232	0.57156	-0.10540

H -2.49477 0.83918 1.73727
C -2.73853 -1.03275 -1.65364
H -0.88836 -1.97110 -1.03237
C -3.74329 -0.11367 -1.32292
H -4.45467 1.29158 0.15894
H -2.78395 -1.57119 -2.60637
H -4.57399 0.06639 -2.01272
O -0.53535 -0.03549 2.55879
H -0.48426 -1.91231 1.67253
B3LYP+D3(BJ)/Def2-SVP Gibbs free energy:-731.349139 a.u.
B3LYP+D3(BJ)/Def2-SVP enthalpy:-731.286848 a.u.
B3LYP+D3(BJ)/Def2-SVP SCF energy:-731.568281 a.u.
PBE0+D3(BJ)/Def2-TZVPP SOLVENT=(DMF) Gibbs free energy:-731.325790 a.u.
PBE0+D3(BJ)/Def2-TZVPP SOLVENT=(DMF) enthalpy:-731.263499 a.u.
PBE0+D3(BJ)/Def2-TZVPP SOLVENT=(DMF) SCF energy:-731.544932 a.u.
Lowest frequency: 25.7093 cm-1

5a(S,R)

Cartesian coordinates

ATOM	X	Y	Z
C	1.03090	0.88027	0.04264
C	0.55261	1.73288	1.05851
C	0.56436	3.11684	0.87814
C	1.06090	3.68333	-0.30152
C	1.54053	2.84416	-1.31309
C	1.52125	1.45869	-1.14338
H	0.17745	1.23375	1.95903
H	0.18400	3.76582	1.67361
H	1.07252	4.76968	-0.43368
H	1.92474	3.27198	-2.24453
H	1.88119	0.80047	-1.93831
C	0.98960	-0.58989	0.22070
C	1.89505	-1.38056	-0.30614
C	2.81159	-2.22318	-0.74094
C	-0.19394	-1.20155	1.11487
C	-1.50436	-0.94088	0.31366
C	-2.56521	-0.32989	0.98785
C	-1.68155	-1.31049	-1.02671
C	-3.78082	-0.08704	0.34071
H	-2.36915	-0.06915	2.03188
C	-2.89280	-1.07070	-1.68091
H	-0.85138	-1.78430	-1.56032
C	-3.95073	-0.45587	-0.99796

H	-4.60400	0.39451	0.88003
H	-3.01553	-1.35895	-2.73044
H	-4.89957	-0.26488	-1.50936
O	-0.19290	-0.74443	2.34342
H	-0.03254	-2.31935	0.99971
H	2.72408	-2.62994	-1.76076
C	4.00182	-2.69185	0.06352
H	4.95809	-2.43185	-0.42867
H	3.99816	-2.23693	1.06523
H	3.99991	-3.79095	0.18953

B3LYP+D3(BJ)/Def2-SVP Gibbs free energy:-731.349009 a.u.
B3LYP+D3(BJ)/Def2-SVP enthalpy:-731.286715 a.u.
B3LYP+D3(BJ)/Def2-SVP SCF energy:-731.568207 a.u.
PBE0+D3(BJ)/Def2-TZVPP SOLVENT=(DMF) Gibbs free energy:-731.325492 a.u.
PBE0+D3(BJ)/Def2-TZVPP SOLVENT=(DMF) enthalpy:-731.263198 a.u.
PBE0+D3(BJ)/Def2-TZVPP SOLVENT=(DMF) SCF energy:-731.544690 a.u.
Lowest frequency: 27.4582 cm-1

5a(R,R)

Cartesian coordinates

ATOM	X	Y	Z
C	-1.23284	0.75338	0.17742
C	-0.79924	1.89468	0.88236
C	-1.07955	3.17170	0.39352
C	-1.80434	3.34424	-0.79125
C	-2.24108	2.21669	-1.49525
C	-1.95422	0.93685	-1.01763
H	-0.23675	1.69809	1.80208
H	-0.73072	4.04835	0.94888
H	-2.02556	4.34858	-1.16556
H	-2.80174	2.33540	-2.42786
H	-2.27978	0.05479	-1.57504
C	-0.90805	-0.60267	0.67791
C	-1.71140	-1.62356	0.49279
C	-2.48424	-2.68421	0.36625
C	0.46239	-0.79246	1.49070
C	1.59902	-0.57330	0.44717
C	1.67709	-1.25850	-0.77305
C	2.60708	0.33909	0.77052
C	2.73825	-1.03329	-1.65383
H	0.88764	-1.97103	-1.03293
C	3.67263	0.57017	-0.10507
H	2.49510	0.83775	1.73761

C 3.74338 -0.11474 -1.32279
H 2.78349 -1.57147 -2.60671
H 4.45528 1.28977 0.15953
H 4.57419 0.06516 -2.01248
H -3.18525 -2.93400 1.17826
C -2.48911 -3.61222 -0.82618
H -2.23289 -4.64875 -0.53686
H -3.48346 -3.65653 -1.30998
H -1.75956 -3.28398 -1.58238
O 0.53523 -0.03629 2.55873
H 0.48353 -1.91287 1.67201
B3LYP+D3(BJ)/Def2-SVP Gibbs free energy:-731.349141 a.u.
B3LYP+D3(BJ)/Def2-SVP enthalpy:-731.286847 a.u.
B3LYP+D3(BJ)/Def2-SVP SCF energy:-731.568281 a.u.
PBE0+D3(BJ)/Def2-TZVPP SOLVENT=(DMF) Gibbs free energy:-731.325795 a.u.
PBE0+D3(BJ)/Def2-TZVPP SOLVENT=(DMF) enthalpy:-731.263501 a.u.
PBE0+D3(BJ)/Def2-TZVPP SOLVENT=(DMF) SCF energy:-731.544935 a.u.
Lowest frequency: 25.6888 cm-1

5a'
Cartesian coordinates
ATOM X Y Z
C 2.47908 -0.52296 0.10922
C 3.61499 -1.15658 -0.45149
C 4.84430 -0.50309 -0.51275
C 4.98592 0.79907 -0.01836
C 3.87178 1.44137 0.53689
C 2.63827 0.79770 0.60058
H 3.50877 -2.17238 -0.83825
H 5.70523 -1.01597 -0.95207
H 5.95184 1.30860 -0.06822
H 3.96576 2.46154 0.92077
H 1.76580 1.30363 1.01882
C 1.21633 -1.16610 0.17584
C 0.08704 -1.63750 0.23666
C -1.27646 -2.08616 0.23822
H -1.33661 -3.11488 -0.15368
C -1.95693 -2.03042 1.60502
H -3.02247 -2.24617 1.42410
H -1.53643 -2.76178 2.31611
H -1.86758 -1.02633 2.05014
C -2.30466 -1.29143 -0.87462
H -1.70703 -1.41752 -1.83113

C	-2.24537	0.21498	-0.53304
C	-1.15163	1.04349	-0.82591
C	-3.36442	0.78514	0.08716
C	-1.16078	2.39655	-0.47216
H	-0.28381	0.62173	-1.33778
C	-3.38037	2.13549	0.44556
H	-4.20956	0.10982	0.24691
C	-2.27392	2.94876	0.17315
H	-0.29481	3.02510	-0.70514
H	-4.26229	2.56358	0.93476
H	-2.28278	4.00797	0.44946
O	-3.47914	-1.83211	-0.82433

B3LYP+D3(BJ)/Def2-SVP Gibbs free energy:-731.344048 a.u.
B3LYP+D3(BJ)/Def2-SVP enthalpy:-731.281272 a.u.
B3LYP+D3(BJ)/Def2-SVP SCF energy:-731.563404 a.u.
PBE0+D3(BJ)/Def2-TZVPP SOLVENT=(DMF) Gibbs free energy:-731.320300 a.u.
PBE0+D3(BJ)/Def2-TZVPP SOLVENT=(DMF) enthalpy:-731.257524 a.u.
PBE0+D3(BJ)/Def2-TZVPP SOLVENT=(DMF) SCF energy:-731.539656 a.u.
Lowest frequency: 16.7125 cm-1

B

Cartesian coordinates

ATOM	X	Y	Z
C	0.51479	-0.10901	-0.00010
C	1.14745	1.18538	-0.00009
C	2.52788	1.32652	0.00002
C	3.38644	0.21199	0.00013
C	2.79519	-1.06457	0.00011
C	1.41764	-1.23178	-0.00002
H	0.50627	2.07103	-0.00017
H	2.95482	2.33683	0.00004
H	4.47296	0.33343	0.00023
H	3.43471	-1.95566	0.00019
H	0.98805	-2.23724	-0.00003
C	-0.86167	-0.26280	-0.00034
C	-2.11031	-0.40950	-0.00008
C	-3.45838	-0.55068	0.00006
H	-3.88799	-1.56220	0.00011
C	-4.42435	0.60528	0.00016
H	-5.09944	0.62281	-0.88510
H	-5.09942	0.62262	0.88538
H	-3.87795	1.56346	0.00019

B3LYP+D3(BJ)/Def2-SVP Gibbs free energy:-386.069762 a.u.

B3LYP+D3(BJ)/Def2-SVP enthalpy:-386.023549 a.u.
B3LYP+D3(BJ)/Def2-SVP SCF energy:-386.184469 a.u.
PBE0+D3(BJ)/Def2-TZVPP SOLVENT=(DMF) Gibbs free energy:-386.079984 a.u.
PBE0+D3(BJ)/Def2-TZVPP SOLVENT=(DMF) enthalpy:-386.033771 a.u.
PBE0+D3(BJ)/Def2-TZVPP SOLVENT=(DMF) SCF energy:-386.194691 a.u.
Lowest frequency: 55.6701 cm-1

TS3(R,S)

Cartesian coordinates

ATOM	X	Y	Z
C	1.60617	-0.01847	-0.20418
C	2.40096	-0.46123	0.88433
C	3.31689	-1.50246	0.72562
C	3.46678	-2.14964	-0.50706
C	2.67233	-1.74023	-1.58698
C	1.75655	-0.69929	-1.43942
H	2.26551	0.01921	1.85326
H	3.92141	-1.81892	1.58250
H	4.18683	-2.96542	-0.62419
H	2.77104	-2.23812	-2.55779
H	1.13789	-0.38649	-2.28464
C	0.62825	1.02302	-0.02785
C	0.41761	2.20646	-0.47685
C	0.10454	3.45235	-0.84819
H	-0.46466	3.59872	-1.77842
C	0.50959	4.70284	-0.10388
H	1.17072	5.36081	-0.70474
H	-0.36223	5.32502	0.18221
H	1.05135	4.45057	0.82151
C	-0.74082	0.40842	1.58049
H	-0.99210	1.48798	1.71202
C	-1.72322	-0.30606	0.68654
C	-1.74200	-1.71031	0.67241
C	-2.66181	0.38858	-0.09602
C	-2.66315	-2.40301	-0.11706
H	-1.01545	-2.22597	1.30483
C	-3.58605	-0.30197	-0.88168
H	-2.64563	1.48269	-0.09206
C	-3.59146	-1.70400	-0.89787
H	-2.66225	-3.49821	-0.12257
H	-4.30893	0.25335	-1.48844
H	-4.31632	-2.24587	-1.51352
O	-0.09551	-0.21366	2.44200

B3LYP+D3(BJ)/Def2-SVP Gibbs free energy:-731.273925 a.u.
B3LYP+D3(BJ)/Def2-SVP enthalpy:-731.210302 a.u.
B3LYP+D3(BJ)/Def2-SVP SCF energy:-731.489852 a.u.
PBE0+D3(BJ)/Def2-TZVPP SOLVENT=(DMF) Gibbs free energy:-731.309345 a.u.
PBE0+D3(BJ)/Def2-TZVPP SOLVENT=(DMF) enthalpy:-731.245722 a.u.
PBE0+D3(BJ)/Def2-TZVPP SOLVENT=(DMF) SCF energy:-731.525272 a.u.
Lowest frequency: -105.0052 cm-1

IM2(R,S)

Cartesian coordinates

ATOM	X	Y	Z
C	1.63186	0.49789	-0.36900
C	2.58738	0.50871	0.69201
C	3.71282	-0.30988	0.66041
C	3.94399	-1.19019	-0.40702
C	3.00387	-1.23646	-1.44817
C	1.87556	-0.42208	-1.43461
H	2.41337	1.17027	1.54235
H	4.42431	-0.26740	1.49259
H	4.82916	-1.83277	-0.42221
H	3.15697	-1.92169	-2.28964
H	1.15385	-0.47048	-2.25396
C	0.47620	1.30182	-0.33611
C	-0.28220	2.26713	-0.63870
C	-1.16026	3.25950	-0.89404
H	-1.80512	3.17495	-1.78021
C	-1.27219	4.53101	-0.08900
H	-0.98296	5.43771	-0.66333
H	-2.30377	4.72136	0.27296
H	-0.61922	4.49185	0.79818
C	-0.77036	0.13045	1.71638
H	-1.17797	1.16616	1.76099
C	-1.50173	-0.77595	0.78831
C	-1.11706	-2.12529	0.68153
C	-2.61524	-0.32345	0.05667
C	-1.82828	-2.99975	-0.14069
H	-0.24993	-2.45356	1.25898
C	-3.32608	-1.19999	-0.76318
H	-2.89583	0.73196	0.11917
C	-2.93824	-2.54385	-0.86408
H	-1.51775	-4.04639	-0.22179
H	-4.18600	-0.83577	-1.33402
H	-3.49743	-3.23119	-1.50630

O 0.09224 -0.25660 2.49525

B3LYP+D3(BJ)/Def2-SVP Gibbs free energy:-731.278311 a.u.

B3LYP+D3(BJ)/Def2-SVP enthalpy:-731.210580 a.u.

B3LYP+D3(BJ)/Def2-SVP SCF energy:-731.490687 a.u.

PBE0+D3(BJ)/Def2-TZVPP SOLVENT=(DMF) Gibbs free energy:-731.311551 a.u.

PBE0+D3(BJ)/Def2-TZVPP SOLVENT=(DMF) enthalpy:-731.243820 a.u.

PBE0+D3(BJ)/Def2-TZVPP SOLVENT=(DMF) SCF energy:-731.523927 a.u.

Lowest frequency: 16.9989 cm-1

TS3(S,S)

Cartesian coordinates

ATOM	X	Y	Z
C	-1.62009	0.44157	-0.05797
C	-1.84703	0.11027	-1.41842
C	-2.95699	-0.63861	-1.80718
C	-3.87858	-1.09528	-0.85475
C	-3.65922	-0.79538	0.49536
C	-2.55017	-0.04807	0.89507
H	-1.13009	0.45994	-2.16588
H	-3.10804	-0.86797	-2.86768
H	-4.75053	-1.68148	-1.16092
H	-4.36345	-1.15523	1.25327
H	-2.36728	0.15772	1.94967
C	-0.45133	1.17029	0.35996
C	0.01792	2.35779	0.23291
C	0.59872	3.56128	0.19031
H	0.41108	4.26851	1.01215
C	1.47913	4.05589	-0.93306
H	1.05437	4.94139	-1.44926
H	2.48400	4.36271	-0.57858
H	1.62462	3.27169	-1.69304
C	0.66585	-0.08782	1.77478
C	1.50149	-0.79876	0.73958
C	1.19174	-2.12629	0.40194
C	2.61903	-0.19409	0.13912
C	1.96695	-2.82503	-0.52678
H	0.33129	-2.58037	0.89908
C	3.39842	-0.89239	-0.78463
H	2.86181	0.84099	0.39833
C	3.07517	-2.21361	-1.12511
H	1.70944	-3.85762	-0.78541
H	4.26472	-0.40642	-1.24551
H	3.68545	-2.76164	-1.84985

O -0.14541 -0.71633 2.47652
H 1.14989 0.84771 2.14462
B3LYP+D3(BJ)/Def2-SVP Gibbs free energy:-731.273923 a.u.
B3LYP+D3(BJ)/Def2-SVP enthalpy:-731.210173 a.u.
B3LYP+D3(BJ)/Def2-SVP SCF energy:-731.489699 a.u.
PBE0+D3(BJ)/Def2-TZVPP SOLVENT=(DMF) Gibbs free energy:-731.309673 a.u.
PBE0+D3(BJ)/Def2-TZVPP SOLVENT=(DMF) enthalpy:-731.245923 a.u.
PBE0+D3(BJ)/Def2-TZVPP SOLVENT=(DMF) SCF energy:-731.525449 a.u.
Lowest frequency: -113.6074 cm-1

IM2(S,S)

Cartesian coordinates

ATOM	X	Y	Z
C	-1.62225	0.73202	-0.19150
C	-1.85611	0.14570	-1.47307
C	-3.02153	-0.56374	-1.74644
C	-4.01052	-0.73829	-0.76579
C	-3.79118	-0.18995	0.50643
C	-2.62974	0.52045	0.79789
H	-1.09554	0.27163	-2.24791
H	-3.16519	-0.98929	-2.74610
H	-4.92487	-1.29725	-0.98547
H	-4.54185	-0.32614	1.29287
H	-2.46689	0.91938	1.80027
C	-0.42967	1.42434	0.09871
C	0.36889	2.40575	0.09782
C	1.28615	3.39320	0.14863
H	1.29258	4.06253	1.02076
C	2.26995	3.70379	-0.95290
H	2.09609	4.69482	-1.42524
H	3.32156	3.72126	-0.59838
H	2.21073	2.95019	-1.75500
C	0.61341	-0.23952	1.88417
C	1.34536	-0.99031	0.82588
C	0.87575	-2.24934	0.40909
C	2.53992	-0.49141	0.27527
C	1.58121	-2.98699	-0.54230
H	-0.05168	-2.61809	0.85291
C	3.24584	-1.23156	-0.67304
H	2.89248	0.49613	0.58672
C	2.77057	-2.48472	-1.08620
H	1.20366	-3.96231	-0.86580
H	4.17050	-0.83052	-1.09972

H 3.32442 -3.06504 -1.83052
O -0.32101 -0.71397 2.51940
H 1.08569 0.72810 2.16953
B3LYP+D3(BJ)/Def2-SVP Gibbs free energy:-731.277972 a.u.
B3LYP+D3(BJ)/Def2-SVP enthalpy:-731.210359 a.u.
B3LYP+D3(BJ)/Def2-SVP SCF energy:-731.490489 a.u.
PBE0+D3(BJ)/Def2-TZVPP SOLVENT=(DMF) Gibbs free energy:-731.311473 a.u.
PBE0+D3(BJ)/Def2-TZVPP SOLVENT=(DMF) enthalpy:-731.243860 a.u.
PBE0+D3(BJ)/Def2-TZVPP SOLVENT=(DMF) SCF energy:-731.523990 a.u.
Lowest frequency: 15.5556 cm-1

TS3(S,R)

Cartesian coordinates

ATOM	X	Y	Z
C	-1.60642	-0.01770	-0.20429
C	-2.40159	-0.45980	0.88421
C	-3.31814	-1.50049	0.72559
C	-3.46829	-2.14781	-0.50699
C	-2.67347	-1.73907	-1.58691
C	-1.75707	-0.69866	-1.43944
H	-2.26597	0.02074	1.85307
H	-3.92295	-1.81642	1.58245
H	-4.18883	-2.96317	-0.62406
H	-2.77238	-2.23707	-2.55763
H	-1.13815	-0.38638	-2.28464
C	-0.62791	1.02322	-0.02807
C	-0.41642	2.20654	-0.47696
C	-0.10245	3.45224	-0.84819
C	0.74091	0.40773	1.58056
C	1.72310	-0.30697	0.68659
C	1.74129	-1.71123	0.67218
C	2.66208	0.38744	-0.09569
C	2.66228	-2.40414	-0.11728
H	1.01443	-2.22672	1.30438
C	3.58616	-0.30333	-0.88134
H	2.64635	1.48156	-0.09151
C	3.59100	-1.70536	-0.89781
H	2.66094	-3.49934	-0.12301
H	4.30936	0.25182	-1.48788
H	4.31574	-2.24740	-1.51346
O	0.09520	-0.21420	2.44186
H	0.99247	1.48721	1.71221
H	0.46684	3.59828	-1.77842

C -0.50660 4.70296 -0.10378
H -1.16729 5.36144 -0.70456
H -1.04849 4.45101 0.82162
H 0.36566 5.32452 0.18232
B3LYP+D3(BJ)/Def2-SVP Gibbs free energy:-731.273926 a.u.
B3LYP+D3(BJ)/Def2-SVP enthalpy:-731.210302 a.u.
B3LYP+D3(BJ)/Def2-SVP SCF energy:-731.489852 a.u.
PBE0+D3(BJ)/Def2-TZVPP SOLVENT=(DMF) Gibbs free energy:-731.309342 a.u.
PBE0+D3(BJ)/Def2-TZVPP SOLVENT=(DMF) enthalpy:-731.245718 a.u.
PBE0+D3(BJ)/Def2-TZVPP SOLVENT=(DMF) SCF energy:-731.525268 a.u.
Lowest frequency: -104.8562 cm-1

IM2(S,R)

Cartesian coordinates

ATOM	X	Y	Z
C	-1.63147	0.49922	-0.36856
C	-2.58712	0.51026	0.69220
C	-3.71304	-0.30776	0.66016
C	-3.94455	-1.18754	-0.40754
C	-3.00426	-1.23401	-1.44861
C	-1.87556	-0.42029	-1.43460
H	-2.41315	1.17154	1.54276
H	-4.42459	-0.26503	1.49227
H	-4.83005	-1.82966	-0.42314
H	-3.15769	-1.91888	-2.29032
H	-1.15369	-0.46872	-2.25381
C	-0.47521	1.30230	-0.33568
C	0.28408	2.26682	-0.63851
C	1.16302	3.25834	-0.89405
C	0.77021	0.12991	1.71663
C	1.50079	-0.77692	0.78837
C	1.11511	-2.12593	0.68150
C	2.61468	-0.32521	0.05673
C	1.82565	-3.00090	-0.14081
H	0.24774	-2.45362	1.25892
C	3.32481	-1.20221	-0.76317
H	2.89601	0.73000	0.11931
C	2.93592	-2.54581	-0.86416
H	1.51428	-4.04728	-0.22195
H	4.18500	-0.83868	-1.33404
H	3.49464	-3.23348	-1.50645
O	-0.09264	-0.25659	2.49552
H	1.17860	1.16532	1.76150

H	1.80754	3.17314	-1.78041
C	1.27638	4.52983	-0.08920
H	0.98675	5.43665	-0.66316
H	0.62452	4.49097	0.79881
H	2.30849	4.71978	0.27141

B3LYP+D3(BJ)/Def2-SVP Gibbs free energy:-731.278308 a.u.
B3LYP+D3(BJ)/Def2-SVP enthalpy:-731.210580 a.u.
B3LYP+D3(BJ)/Def2-SVP SCF energy:-731.490687 a.u.
PBE0+D3(BJ)/Def2-TZVPP SOLVENT=(DMF) Gibbs free energy:-731.311547 a.u.
PBE0+D3(BJ)/Def2-TZVPP SOLVENT=(DMF) enthalpy:-731.243819 a.u.
PBE0+D3(BJ)/Def2-TZVPP SOLVENT=(DMF) SCF energy:-731.523926 a.u.
Lowest frequency: 17.0138 cm-1

TS3

(R,R)

Cartesian coordinates

ATOM	X	Y	Z
C	1.62009	0.44157	-0.05797
C	2.55017	-0.04807	0.89507
C	3.65922	-0.79538	0.49536
C	3.87858	-1.09528	-0.85475
C	2.95699	-0.63861	-1.80718
C	1.84703	0.11027	-1.41842
H	2.36728	0.15772	1.94967
H	4.36345	-1.15523	1.25327
H	4.75053	-1.68148	-1.16092
H	3.10804	-0.86797	-2.86768
H	1.13009	0.45994	-2.16588
C	0.45133	1.17029	0.35996
C	-0.01792	2.35779	0.23291
C	-0.59873	3.56128	0.19031
C	-0.66585	-0.08782	1.77478
C	-1.50149	-0.79876	0.73958
C	-2.61903	-0.19409	0.13912
C	-1.19174	-2.12629	0.40194
C	-3.39842	-0.89239	-0.78463
H	-2.86181	0.84099	0.39833
C	-1.96695	-2.82503	-0.52678
H	-0.33129	-2.58037	0.89908
C	-3.07517	-2.21361	-1.12511
H	-4.26471	-0.40642	-1.24551
H	-1.70943	-3.85762	-0.78541
H	-3.68545	-2.76164	-1.84985

H	-0.41108	4.26851	1.01215
C	-1.47913	4.05589	-0.93306
H	-2.48400	4.36271	-0.57858
H	-1.05437	4.94139	-1.44926
H	-1.62462	3.27169	-1.69304
O	0.14542	-0.71633	2.47652
H	-1.14989	0.84771	2.14462

B3LYP+D3(BJ)/Def2-SVP Gibbs free energy:-731.273923 a.u.

B3LYP+D3(BJ)/Def2-SVP enthalpy:-731.210173 a.u.

B3LYP+D3(BJ)/Def2-SVP SCF energy:-731.489699 a.u.

PBE0+D3(BJ)/Def2-TZVPP SOLVENT=(DMF) Gibbs free energy:-731.309673 a.u.

PBE0+D3(BJ)/Def2-TZVPP SOLVENT=(DMF) enthalpy:-731.245923 a.u.

PBE0+D3(BJ)/Def2-TZVPP SOLVENT=(DMF) SCF energy:-731.525449 a.u.

Lowest frequency: -113.6077 cm-1

IM2(R,R)

Cartesian coordinates

ATOM	X	Y	Z
C	2.40480	-0.59900	0.12614
C	2.89013	0.35764	1.07502
C	4.15570	0.92107	0.95800
C	5.01453	0.57602	-0.09865
C	4.56106	-0.35946	-1.04240
C	3.29853	-0.93383	-0.94161
H	2.23648	0.64307	1.90295
H	4.48423	1.64949	1.70787
H	6.00813	1.02439	-0.18449
H	5.21065	-0.64672	-1.87695
H	2.96627	-1.66070	-1.68745
C	1.13290	-1.17244	0.23457
C	0.02948	-1.74257	0.36080
C	-1.20987	-2.31142	0.46446
H	-1.45581	-3.12207	-0.23247
C	-2.10398	-2.17718	1.66678
H	-1.83504	-1.29153	2.26564
H	-3.16176	-2.07003	1.35459
H	-2.05983	-3.05572	2.34570
C	-2.85033	-0.91726	-1.14459
H	-2.17398	-1.36383	-1.91122
C	-2.40869	0.40515	-0.63794
C	-3.25394	1.13795	0.21963
C	-1.18292	0.97325	-1.03272
C	-2.87880	2.40076	0.67417

H	-4.20545	0.68316	0.50514
C	-0.80885	2.23779	-0.57556
H	-0.51407	0.40597	-1.68361
C	-1.65377	2.95793	0.27813
H	-3.54198	2.96099	1.34140
H	0.15552	2.65774	-0.87465
H	-1.35732	3.94793	0.63771
O	-3.94649	-1.41107	-0.88944

B3LYP+D3(BJ)/Def2-SVP Gibbs free energy:-731.281852 a.u.

B3LYP+D3(BJ)/Def2-SVP enthalpy:-731.215788 a.u.

B3LYP+D3(BJ)/Def2-SVP SCF energy:-731.496246 a.u.

PBE0+D3(BJ)/Def2-TZVPP SOLVENT=(DMF) Gibbs free energy:-731.311140 a.u.

PBE0+D3(BJ)/Def2-TZVPP SOLVENT=(DMF) enthalpy:-731.245076 a.u.

PBE0+D3(BJ)/Def2-TZVPP SOLVENT=(DMF) SCF energy:-731.525534 a.u.

Lowest frequency: 14.4316 cm-1

11. Experimental data

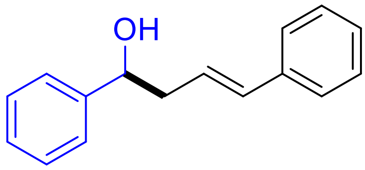

(*E*)-1,4-Diphenylbut-3-en-1-ol (**3a**): **Condition I** 93% yield, 104 mg; **Condition II** 82% yield, 92 mg; **Condition III** 54% yield, 60 mg; white solid, m.p. 102-104˚C; ^{1}H NMR (400 MHz, $CDCl_3$) δ 7.35 (m, 9H), 7.22 (t, *J* = 6.0 Hz, 1H), 6.51 (d, *J* = 12 Hz, 1H), 6.21 (m, 1H), 4.82 (m, 1H), 2.67 (m, 2H), 2.07 (br, 1H); ^{13}C NMR (101 MHz, $CDCl_3$) δ 143.9, 137.2, 133.4, 128.51, 128.47, 127.6, 127.3, 126.2, 125.9, 125.8, 73.7, 43.1; These data are in accordance with the literature. [9]

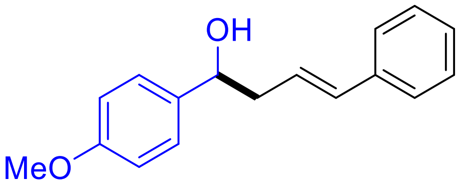

(*E*)-1-(4-Methoxyphenyl)-4-phenylbut-3-en-1-ol (**3b**): **Condition I** 79% yield, 101 mg; **Condition II** 87% yield, 111 mg; white solid, m.p. 89-91˚C; ^{1}H NMR (600 MHz, $CDCl_3$) δ 7.31 (m, 6H), 7.21 (t, *J* = 9.0 Hz, 1H), 6.90 (d, *J* = 8.0 Hz, 2H), 6.49 (d, *J* = 18.0 Hz, 1H), 6.19 (m, 1H), 4.75 (t, *J* = 6.0 Hz, 1H), 3.80 (s, 3H), 2.64 (t, *J* = 6.0 Hz, 2H), 2.04 (br, 1H); ^{13}C NMR (151 MHz, $CDCl_3$) δ 159.1, 137.2, 136.1, 133.2, 128.5, 127.3, 127.1, 126.13, 126.06, 113.8, 73.4, 55.3, 43.0; These data are in accordance with the literature. [9]

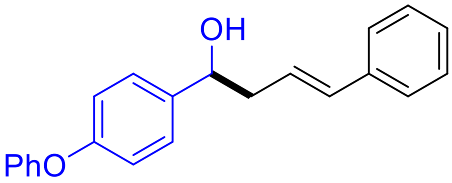

(*E*)-1-(4-phenoxyphenyl)-4-phenylbut-3-en-1-ol (**3c**): **Condition II** 73% yield, 115 mg; colorless oil; ^{1}H NMR (400 MHz, $CDCl_3$) δ 7.32 (m, 8H), 7.22 (t, *J* = 6.0 Hz, 1H), 7.10 (t, *J* = 8.0 Hz, 1H), 7.01 (m, 4H), 6.50 (d, *J* = 8.0 Hz, 1H), 6.21 (m, 1H), 4.80 (t, *J* = 6.0 Hz, 1H), 2.66 (t, *J* = 8.0 Hz, 2H), 2.01 (br, 1H); ^{13}C NMR (101 MHz, $CDCl_3$) δ 157.2, 156.7, 138.7, 137.1, 133.5, 129.7, 128.5, 127.4, 127.3, 126.1, 125.8, 123.3, 118.84, 118.81, 73.3, 43.1; HRMS (ESI): calcd for $C_{22}H_{19}O$ $(M-H_2O+H)^+$ 299.1430, found 299.1428.

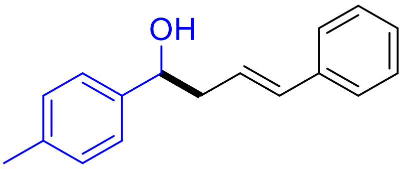

(*E*)-4-Phenyl-1-(*p*-tolyl)but-3-en-1-ol (**3d**): **Condition II** 63% yield, 75 mg; **Condition III** 34% yield, 40 mg; white solid, m.p. 76-78˚C; ^{1}H NMR (400 MHz, $CDCl_3$) δ 7.29 (m, 6H), 7.18 (m, 3H), 6.48 (d, *J* = 16.0 Hz, 1H), 6.19 (m, 1H), 4.75 (t, *J* = 6.0 Hz, 1H), 2.64 (t, *J* = 6.0 Hz, 2H), 2.34 (s, 3H), 2.12 (br,

1H); ^{13}C NMR (101 MHz, $CDCl_3$) δ 140.9, 137.23, 137.20, 133.2, 129.1, 128.5, 127.2, 126.11, 126.06, 125.7, 73.6, 43.0, 21.1; These data are in accordance with the literature.[9]

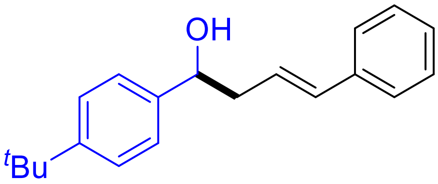

(*E*)-1-(4-(*tert*-Butyl)phenyl)-4-phenylbut-3-en-1-ol (**3e**): **Condition II** 82% yield, 115 mg; white solid, m.p. 77-79 °C; ^{1}H NMR (400 MHz, $CDCl_3$) δ 7.35 (m, 8H), 7.22 (t, *J* = 6.0 Hz, 1H), 6.52 (d, *J* = 16.0 Hz, 1H), 6.23 (m, 1H), 4.79 (t, *J* = 6.0 Hz, 1H), 2.67 (t, *J* = 8.0 Hz, 2H), 2.05 (br, 1H), 1.33 (s, 9H); ^{13}C NMR (101 MHz, $CDCl_3$) δ 150.6, 140.9, 137.3, 133.2, 128.5, 127.3, 126.22, 126.16, 125.6, 125.4, 73.6, 42.9, 34.5, 31.4; HRMS (ESI): calcd for $C_{20}H_{23}$ $(M-H_2O+H)^+$ 263.1794, found 263.1795.

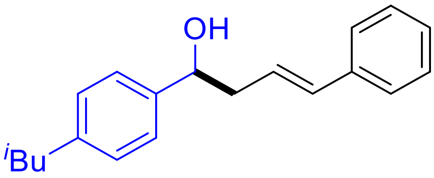

(*E*)-1-(4-Isobutylphenyl)-4-phenylbut-3-en-1-ol (**3f**): **Condition II** 92% yield, 129 mg; white solid, m.p. 73-75 °C; ^{1}H NMR (400 MHz, $CDCl_3$) δ 7.34 (d, *J* = 8.0 Hz, 2H), 7.29 (m, 4H), 7.21 (t, *J* = 6.0 Hz, 1H), 7.14 (d, *J* = 8.0 Hz, 2H), 6.49 (d, *J* = 16.0 Hz, 1H), 6.21 (m, 1H), 4.78 (t, *J* = 8.0 Hz, 1H), 2.66 (t, *J* = 6.0 Hz, 2H), 2.47 (d, *J* = 8.0 Hz, 2H), 2.03 (br, 1H), 1.86 (m, 1H), 0.90 (d, *J* = 8.0 Hz, 6H); ^{13}C NMR (101 MHz, $CDCl_3$) δ 141.2, 141.1, 137.3, 133.3, 129.2, 128.5, 127.3, 126.1, 125.6, 73.7, 45.1, 43.0, 30.2, 22.4; HRMS (ESI): calcd for $C_{20}H_{23}$ $(M-H_2O+H)^+$ 263.1794, found 263.1791.

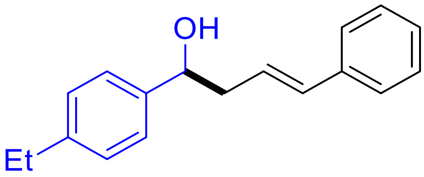

(*E*)-1-(4-Ethylphenyl)-4-phenylbut-3-en-1-ol (**3g**): **Condition II** 67% yield, 85 mg; white solid, m.p. 75-77 °C; ^{1}H NMR (400 MHz, $CDCl_3$) δ 7.33 (m, 6H), 7.21 (t, *J* = 8.0 Hz, 3H), 6.50 (d, *J* = 16.0 Hz, 1H), 6.22 (m, 1H), 4.79 (t, *J* = 6.0 Hz, 1H), 2.66 (q, *J* = 8.0 Hz, 4H), 2.03 (br, 1H), 1.24 (t, *J* = 8.0 Hz, 3H); ^{13}C NMR (101 MHz, $CDCl_3$) δ 143.7, 141.2, 137.3, 133.3, 128.5, 128.0, 127.3, 126.14, 126.11, 125.8, 73.7, 43.0, 28.5, 15.6; HRMS (ESI): calcd for $C_{18}H_{19}$ $(M-H_2O+H)^+$ 235.1481, found 235.1478.

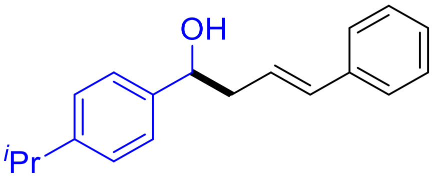

(*E*)-1-(4-Isopropylphenyl)-4-phenylbut-3-en-1-ol (**3h**): **Condition II** 84% yield, 112 mg; white solid, m.p. 58-60 °C; ^{1}H NMR (400 MHz, $CDCl_3$) δ 7.31 (m, 6H), 7.22 (m, 3H), 6.50 (d, *J* = 16.0 Hz, 1H),

6.22 (m, 1H), 4.77 (t, J = 6.0 Hz, 1H), 2.92 (m, 1H), 2.66 (t, J = 6.0 Hz, 2H), 2.05 (br, 1H), 1.25 (d, J = 8.0 Hz, 6H); ^{13}C NMR (101 MHz, $CDCl_3$) δ 148.3, 141.3, 137.3, 133.2, 128.5, 127.3, 126.5, 126.2, 126.1, 125.8, 73.6, 42.9, 33.8, 24.0; HRMS (ESI): calcd for $C_{19}H_{21}$ $(M-H_2O+H)^+$ 249.1638, found 249.1636.

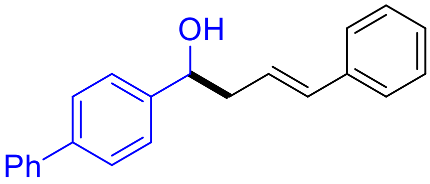

(*E*)-1-([1,1'-Biphenyl]-4-yl)-4-phenylbut-3-en-1-ol (**3i**): **Condition II** 71% yield, 107 mg; white solid, m.p. 164-166 °C; ^{1}H NMR (400 MHz, $CDCl_3$) δ 7.60 (d, J = 8.0 Hz, 4H), 7.45 (m, 4H), 7.32 (m, 5H), 7.22 (t, J = 6.0 Hz, 1H), 6.53 (d, J = 16.0 Hz, 1H), 6.24 (m, 1H), 4.87 (t, J = 6.0 Hz, 1H), 2.71 (m, 2H), 2.12 (br, 1H); ^{13}C NMR (101 MHz, $CDCl_3$) δ 142.9, 140.8, 140.5, 137.2, 133.5, 128.8, 128.5, 127.4, 127.3, 127.2, 127.1, 126.3, 126.2, 125.8, 73.5, 43.1; HRMS (ESI): calcd for $C_{22}H_{19}$ $(M-H_2O+H)^+$ 283.1481, found 283.1485.

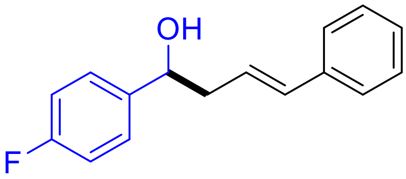

(*E*)-1-(4-Fluorophenyl)-4-phenylbut-3-en-1-ol (**3j**): **Condition II** 88% yield, 107 mg; white solid, m.p. 98-100 °C, ^{1}H NMR (600 MHz, $CDCl_3$) δ 7.34 (m, 4H), 7.30 (t, J = 9.0 Hz, 2H), 7.22 (t, J = 6.0 Hz, 1H), 7.05 (t, J = 9.0 Hz, 2H), 6.49 (d, J = 12.0 Hz, 1H), 6.18 (m, 1H), 4.80 (t, J = 6.0 Hz, 1H), 2.63 (m, 2H), 2.09 (br, 1H); ^{13}C NMR (151 MHz, $CDCl_3$) δ 162.2 (d, $J_{F\text{-}C}$ = 244.6 Hz), 139.6 (d, $J_{F\text{-}C}$ = 3.0 Hz), 137.1, 133.7, 128.6, 127.5, 127.4, 126.2, 125.5, 115.3 (d, $J_{F\text{-}C}$ = 21.1 Hz), 73.1, 43.2; These data are in accordance with the literature.[9]

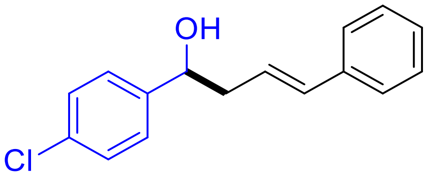

(*E*)-1-(4-Chlorophenyl)-4-phenylbut-3-en-1-ol (**3k**): **Condition I** 46% yield, 60 mg; **Condition II** 61% yield, 79 mg; white solid, m.p. 118-120 °C; ^{1}H NMR (400 MHz, $CDCl_3$) δ 7.32 (m, 8H), 7.22 (t, J = 8.0 Hz, 1H), 6.49 (d, J = 16.0 Hz, 1H), 6.17 (m, 1H), 4.80 (dd, J = 8.0 Hz, J = 4.0 Hz, 1H), 2.63 (m, 2H), 2.04 (br, 1H); ^{13}C NMR (101 MHz, $CDCl_3$) δ 142.3, 137.0, 133.8, 133.2, 128.58, 128.55, 127.5, 127.2, 126.2, 125.3, 73.0, 43.1; These data are in accordance with the literature.[9]

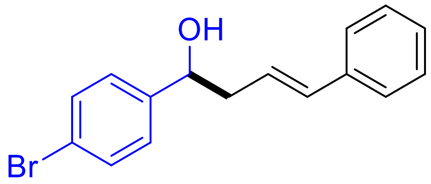

(*E*)-1-(4-Bromophenyl)-4-phenylbut-3-en-1-ol (**3l**): **Condition II** 61% yield, 92 mg; white solid, m.p. 115-117 ˚C; ^{1}H NMR (400 MHz, $CDCl_3$) δ 7.48 (d, *J* = 8.0 Hz, 2H), 7.28 (m, 7H), 6.49 (d, *J* = 16.0 Hz, 1H), 6.16 (m, 1H), 4.78 (t, *J* = 6.0 Hz, 1H), 2.62 (m, 2H), 2.12 (br, 1H); ^{13}C NMR (101 MHz, $CDCl_3$) δ 142.8, 137.0, 133.9, 131.5, 128.6, 127.53, 127.46, 126.2, 125.2, 121.3, 73.0, 43.1; These data are in accordance with the literature. [9]

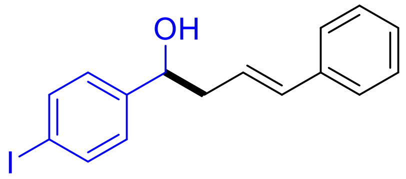

(*E*)-1-(4-Iodophenyl)-4-phenylbut-3-en-1-ol (**3m**): **Condition I** 61% yield, 107 mg; **Condition II** 58% yield, 102 mg; white solid, m.p. 126-128 ˚C; ^{1}H NMR (400 MHz, $CDCl_3$) δ 7.68 (d, *J* = 8.0 Hz, 2H), 7.31 (m, 4H), 7.22 (t, *J* = 8.0 Hz, 1H), 7.13 (d, *J* = 12.0 Hz, 2H), 6.48 (d, *J* = 16.0 Hz, 1H), 6.15 (m, 1H), 4.75 (dd, *J* = 8.0 Hz, *J* = 4.0 Hz, 1H), 2.61 (m, 2H), 2.13 (br, 1H); ^{13}C NMR (101 MHz, $CDCl_3$) δ 143.5, 137.5, 137.0, 133.8, 128.5, 127.8, 127.4, 126.2, 125.2, 92.9, 73.1, 43.0; HRMS (ESI): calcd for $C_{16}H_{14}I$ $(M-H_2O+H)^+$ 333.0135, found 333.0137.

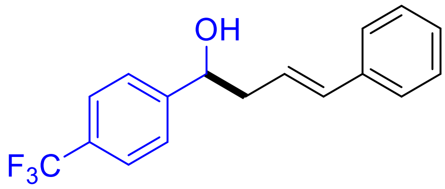

(*E*)-4-Phenyl-1-(4-(trifluoromethyl)phenyl)but-3-en-1-ol (**3n**): **Condition I** 24% yield, 35 mg; **Condition II** 62% yield, 91 mg; **Condition III** 27% yield, 40 mg; white solid, m.p. 111-113 ˚C; ^{1}H NMR (400 MHz, $CDCl_3$) δ 7.62 (d, *J* = 8.0 Hz, 2H), 7.51 (d, *J* = 8.0 Hz, 2H), 7.32 (m, 4H), 7.24 (t, *J* = 6.0 Hz, 1H), 6.51 (d, *J* = 16.0 Hz, 2H), 6.18 (m, 1H), 4.88 (t, *J* = 6.0 Hz, 1H), 2.65 (m, 2H), 2.19 (br, 1H); ^{13}C NMR (101 MHz, $CDCl_3$) δ 147.8, 136.9, 134.2, 129.9, 128.6, 127.6, 126.2, 126.1, 125.4 (q, J_{F-C} = 4.0 Hz), 124.1 (q, J_{F-C} = 271 Hz), 124.9, 73.0, 43.2; HRMS (ESI): calcd for $C_{17}H_{14}F_3$ $(M-H_2O+H)^+$ 275.1042, found 275.1044.

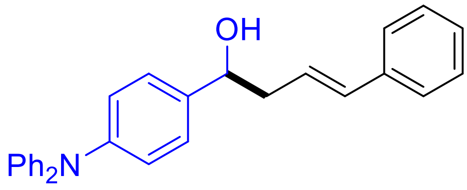

(*E*)-1-(4-(Diphenylamino)phenyl)-4-phenylbut-3-en-1-ol (**3o**): **Condition II** 78% yield, 153 mg; colorless oil; ^{1}H NMR (400 MHz, $CDCl_3$) δ 7.27 (m, 11H), 7.07 (m, 6H), 6.99 (t, *J* = 8.0 Hz, 2H), 6.49

(d, J = 16.0 Hz, 1H), 6.22 (m, 1H), 4.74 (t, J = 6.0 Hz, 1H), 2.65 (t, J = 6.0 Hz, 2H), 2.20 (br, 1H); ^{13}C NMR (101 MHz, $CDCl_3$) δ 147.7, 147.2, 138.0, 137.2, 133.3, 129.2, 128.5, 127.3, 126.8, 126.11, 126.06, 124.2, 123.9, 122.7, 73.4, 42.9; HRMS (ESI): calcd for $C_{28}H_{26}NO$ $(M+H)^+$ 392.2009, found 392.2000.

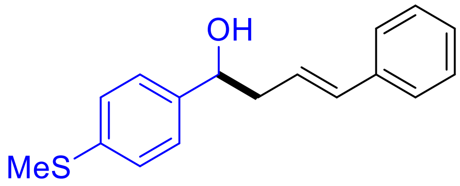

(*E*)-1-(4-(Methylthio)phenyl)-4-phenylbut-3-en-1-ol (**3p**): **Condition II** 71% yield, 96 mg; white solid, m.p. 111-113˚C; ^{1}H NMR (400 MHz, $CDCl_3$) δ 7.32 (m, 6H), 7.23 (m, 3H), 6.49 (d, J = 16.0 Hz, 1H), 6.18 (m, 1H), 4.77 (t, J = 6.0 Hz, 1H), 2.64 (t, J = 8.0 Hz, 2H), 2.48 (s, 3H), 2.08 (br, 1H); ^{13}C NMR (101 MHz, $CDCl_3$) δ 140.8, 137.6, 137.1, 133.5, 128.5, 127.3, 126.7, 126.4, 126.1, 125.7, 73.3, 43.0, 15.9; HRMS (ESI): calcd for $C_{17}H_{17}S$ $(M-H_2O+H)^+$ 253.1045, found 253.1042.

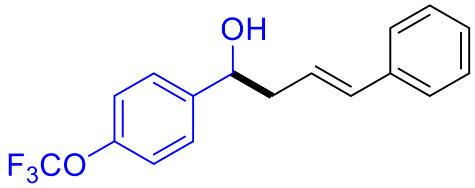

(*E*)-4-Phenyl-1-(4-(trifluoromethoxy)phenyl)but-3-en-1-ol (**3q**): **Condition II** 73% yield, 113 mg; white solid, m.p. 84-86˚C; ^{1}H NMR (400 MHz, $CDCl_3$) δ 7.42 (d, J = 8.0 Hz, 2H), 7.32 (m, 4H), 7.22 (t, J = 8.0 Hz, 3H), 6.50 (d, J = 16.0 Hz, 1H), 6.19 (m, 1H), 4.83 (t, J = 6.0 Hz, 1H), 2.64 (m, 2H), 2.14 (br, 1H); ^{13}C NMR (101 MHz, $CDCl_3$) δ 148.5, 142.5, 137.0, 134.0, 128.6, 127.5, 127.2, 126.2, 125.2, 121.0, 120.5 (q, J_{F-C} = 258.6 Hz), 72.9, 43.2; HRMS (ESI): calcd for $C_{17}H_{14}F_3O$ $(M-H_2O+H)^+$ 291.0091, found 291.0091.

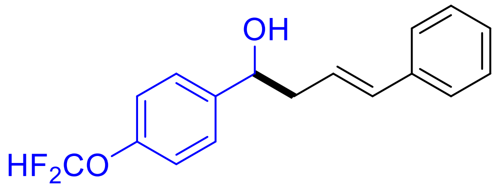

(*E*)-1-(4-(Difluoromethoxy)phenyl)-4-phenylbut-3-en-1-ol (**3r**): **Condition II** 56% yield, 81 mg; white solid, m.p. 75-77˚C; ^{1}H NMR (400 MHz, $CDCl_3$) δ 7.33 (m, 6H), 7.22 (t, J = 8.0 Hz, 1H), 7.10 (d, J = 8.0 Hz, 2H), 6.49 (t, J_{F-H} = 74.0 Hz, 1H), 6.48 (d, J = 16.0 Hz, 1H), 6.17 (m, 1H), 4.79 (dd, J = 4.0 Hz, J = 8.0 Hz, 1H), 2.62 (m, 2H), 2.17 (br, 1H); ^{13}C NMR (101 MHz, $CDCl_3$) δ 150.5 (t, J_{F-C} = 2.5 Hz), 141.1, 137.0, 133.7, 128.5, 127.4, 127.3, 126.1, 125.4, 119.5, 115.9 (t, J_{F-C} = 260.6 Hz), 73.0, 43.1; HRMS (ESI): calcd for $C_{17}H_{15}F_2O$ $(M-H_2O+H)^+$ 273.1085, found 273.1088.

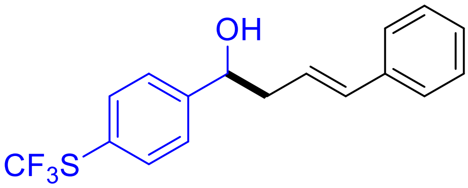

(*E*)-4-Phenyl-1-(4-((trifluoromethyl)thio)phenyl)but-3-en-1-ol (**3s**): **Condition II** 27% yield, 44 mg; white solid, m.p. 65-67˚C; ^{1}H NMR (400 MHz, $CDCl_3$) δ 7.64 (d, *J* = 8.0 Hz, 2H), 7.44 (d, *J* = 8.0 Hz, 2H), 7.32 (m, 4H), 7.22 (t, *J* = 8.0 Hz, 1H), 6.49 (d, *J* = 16 Hz, 1H), 6.18 (m, 1H), 4.85 (dd, *J* = 8.0 Hz, *J* = 4.0 Hz, 1H), 2.64 (m, 2H), 2.45 (br, 1H); ^{13}C NMR (101 MHz, $CDCl_3$) δ 147.0, 136.9, 136.4, 134.1, 129.7 (q, $J_{F\text{-}C}$ = 291.9 Hz), 128.6, 127.5, 126.9, 126.2, 125.1, 123.2 (d, $J_{F\text{-}C}$ = 8.0 Hz), 73.0, 43.1; HRMS (ESI): calcd for $C_{17}H_{14}F_3S$ $(M\text{-}H_2O\text{+}H)^+$ 307.0763, found 307.0764.

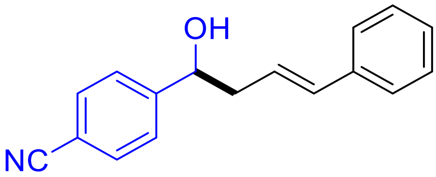

(*E*)-4-(1-Hydroxy-4-phenylbut-3-en-1-yl)benzonitrile (**3t**): **Condition I** 14% yield, 17 mg; **Condition II** 50% yield, 62 mg; white solid, m.p. 124-126˚C; ^{1}H NMR (400 MHz, $CDCl_3$) δ 7.63 (d, *J* = 8.0 Hz, 2H), 7.49 (d, *J* = 8.0 Hz, 2H), 7.31 (m, 4H), 7.23 (t, *J* = 8.0 Hz, 1H), 6.49 (d, *J* = 16.0 Hz, 1H), 6.15 (m, 1H), 4.87 (dd, *J* = 8.0 Hz, *J* = 4.0 Hz, 1H), 2.62 (m, 2H), 2.37 (br, 1H); ^{13}C NMR (101 MHz, $CDCl_3$) δ 149.1, 136.7, 134.3, 132.2, 128.6, 127.6, 126.5, 126.1, 124.5, 118.8, 111.2, 72.8, 43.0; These data are in accordance with the literature. [10]

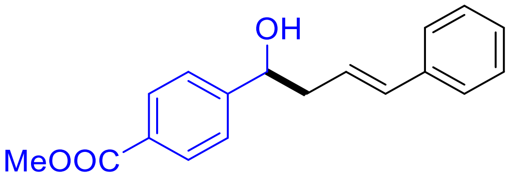

Methyl (*E*)-4-(1-hydroxy-4-phenylbut-3-en-1-yl)benzoate (**3u**): **Condition I** 14% yield, 18 mg; **Condition II** 30% yield, 42 mg; white solid, m.p. 101-103˚C; ^{1}H NMR (400 MHz, $CDCl_3$) δ 8.03 (d, *J* = 12.0 Hz, 2H), 7.45 (d, *J* = 8.0 Hz, 2H), 7.32 (m, 4H), 7.24 (t, *J* = 6.0 Hz, 1H), 6.49 (d, *J* = 16.0 Hz, 1H), 6.17 (m, 1H), 4.87 (dd, *J* = 8.0 Hz, *J* = 4.0 Hz, 1H), 3.91 (s, 3H), 2.65 (m, 2H), 2.29 (br, 1H); ^{13}C NMR (101 MHz, $CDCl_3$) δ 166.9, 149.0, 136.9, 133.9, 129.8, 129.3, 128.5, 127.5, 126.2, 125.7, 125.1, 73.2, 52.1, 43.0; HRMS (ESI): calcd for $C_{18}H_{17}O_2$ $(M\text{-}H_2O\text{+}H)^+$ 265.1223, found 265.1223.

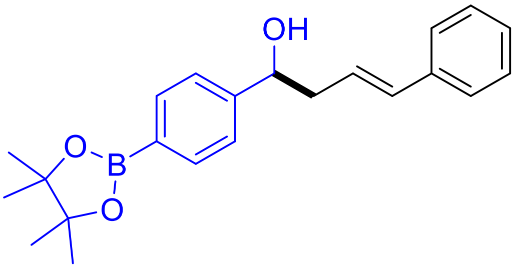

(*E*)-4-Phenyl-1-(4-(4,4,5,5-tetramethyl-1,3,2-dioxaborolan-2-yl)phenyl)but-3-en-1-ol (**3v**):

Condition I 26% yield, 46 mg; **Condition II** 32% yield, 56 mg; colorless oil; ^{1}H NMR (400 MHz, $CDCl_3$) δ 7.81 (d, *J* = 8.0 Hz, 2H), 7.39 (d, *J* = 8.0 Hz, 2H), 7.33 (m, 4H), 7.21 (d, *J* = 8.0 Hz, 1H), 6.50 (d, *J* = 16.0 Hz, 1H), 6.18 (m, 1H), 4.83 (t, *J* = 8.0 Hz, 1H), 2.66 (m, 2H), 1.35 (s, 12H), 1.26 (br, 1H); ^{13}C NMR (101 MHz, $CDCl_3$) δ 147.0, 137.1, 135.0, 133.5, 128.5, 127.3, 126.2, 125.7, 125.1, 83.8, 73.7, 43.0, 24.9; HRMS (ESI): calcd for $C_{22}H_{26}BO_2$ $(M\text{-}H_2O+H)^+$ 333.2020, found 333.2023.

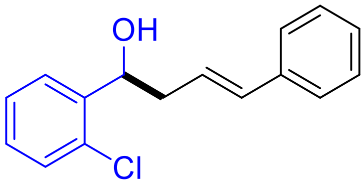

(*E*)-1-(2-Chlorophenyl)-4-phenylbut-3-en-1-ol (**3w**): **Condition II** 75% yield, 97 mg; white solid, m.p. 60-62˚C; ^{1}H NMR (400 MHz, $CDCl_3$) δ 7.60 (dd, *J* = 4.0 Hz, *J* = 8.0 Hz, 1H), 7.33 (m, 6H), 7.21 (m, 2H), 6.51 (d, *J* = 16 Hz, 1H), 6.26 (m, 1H), 5.24 (dd, *J* = 4.0 Hz, *J* = 8.0 Hz, 1H), 2.77 (m, 1H), 2.54 (m, 1H), 2.19 (br, 1H); ^{13}C NMR (101 MHz, $CDCl_3$) δ 141.2, 137.1, 133.6, 131.7, 129.4, 128.52, 128.48, 127.4, 127.1, 127.0, 126.2, 125.6, 70.1, 41.3; HRMS (ESI): calcd for $C_{16}H_{14}Cl$ $(M\text{-}H_2O+H)^+$ 241.0779, found 241.0777.

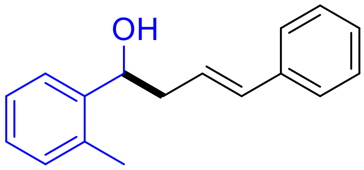

(*E*)-4-Phenyl-1-(*o*-tolyl)but-3-en-1-ol (**3x**): **Condition II** 78% yield; 93 mg; colorless oil; ^{1}H NMR (400 MHz, $CDCl_3$) δ 7.50 (d, *J* = 8.0 Hz, 1H), 7.34 (d, *J* = 8.0 Hz, 2H), 7.28 (t, *J* = 8.0 Hz, 2H), 7.20 (m, 3H), 7.14 (t, *J* = 8.0 Hz, 1H), 6.49 (d, *J* = 16.0 Hz, 1H), 6.22 (m, 1H), 5.02 (dd, *J* = 4.0 Hz, *J* = 8.0 Hz, 1H), 2.60 (m, 2H), 2.34 (s, 3H), 2.09 (br, 1H); ^{13}C NMR (101 MHz, $CDCl_3$) δ 142.0, 137.2, 134.3, 133.2, 130.3, 128.5, 127.26, 127.25, 126.3, 126.2, 126.1, 125.1, 70.1, 41.8, 19.0; HRMS (ESI): calcd for $C_{17}H_{17}$ $(M\text{-}H_2O+H)^+$ 221.1325, found 221.1323.

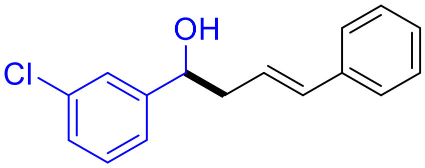

(*E*)-1-(3-Chlorophenyl)-4-phenylbut-3-en-1-ol (**3y**): **Condition II** 57% yield, 74 mg; colorless oil; ^{1}H NMR (400 MHz, $CDCl_3$) δ 7.39 (s, 1H), 7.33 (m, 4H), 7.23 (m, 4H), 6.49 (d, *J* = 8.0 Hz, 1H), 6.16 (m, 1H), 4.77 (dd, *J* = 4.0 Hz, *J* = 8.0 Hz, 1H), 2.62 (m, 2H), 2.14 (br, 1H). ^{13}C NMR (101 MHz, $CDCl_3$) δ 146.0, 137.0, 134.4, 133.9, 129.7, 128.5, 127.6, 127.4, 126.2, 126.0, 125.2, 123.9, 73.0, 43.0; HRMS (ESI): calcd for $C_{16}H_{14}Cl$ $(M\text{-}H_2O+H)^+$ 241.0779, found 241.0778.

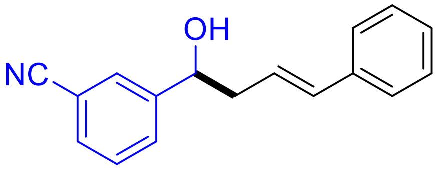

(*E*)-3-(1-Hydroxy-4-phenylbut-3-en-1-yl)benzonitrile (**3z**): **Condition II** 48% yield, 60 mg; colorless oil; ^{1}H NMR (400 MHz, $CDCl_3$) δ 7.70 (s, 1H), 7.61 (d, *J* = 8.0 Hz, 1H), 7.56 (d, *J* = 8.0 Hz, 1H), 7.45 (t, *J* = 8.0 Hz, 1H), 7.32 (m, 4H), 7.23 (m, 1H), 6.50 (d, *J* = 16.0 Hz, 1H), 6.16 (m, 1H), 4.84 (d, *J* = 8.0 Hz, *J* = 4.0 Hz, 1H), 2.62 (m, 2H), 2.39 (br, 1H). ^{13}C NMR (101 MHz, $CDCl_3$) δ 145.3, 136.8, 134.3, 131.1, 130.3, 129.4, 129.2, 128.6, 127.6, 126.2, 124.6, 118.8, 112.4, 72.5, 43.1; HRMS (ESI): calcd for $C_{17}H_{14}N$ $(M-H_2O+H)^+$ 232.1121, found 232.1120.

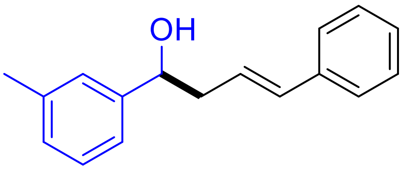

(*E*)-4-Phenyl-1-(*m*-tolyl)but-3-en-1-ol (**3aa**): **Condition II** 93% yield, 111 mg; colorless oil; ^{1}H NMR (400 MHz, $CDCl_3$) δ 7.30 (m, 4H), 7.20 (m, 4H), 7.09 (d, *J* = 8.0 Hz, 1H), 6.49 (d, *J* = 16.0 Hz, 1H), 6.19 (m, 1H), 4.75 (t, *J* = 6.0 Hz, 1H), 2.64 (t, *J* = 6.0 Hz, 2H), 2.36 (s, 3H), 2.07 (br, 1H). ^{13}C NMR (101 MHz, $CDCl_3$) δ 143.9, 138.1, 137.2, 133.2, 128.5, 128.32, 128.30, 127.2, 126.5, 126.12, 126.06, 122.8, 73.7, 43.0, 21.4; HRMS (ESI): calcd for $C_{17}H_{17}$ $(M-H_2O+H)^+$ 221.1325, found 221.1324.

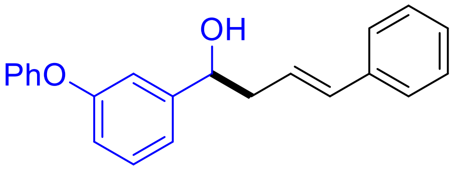

(*E*)-1-(3-Phenoxyphenyl)-4-phenylbut-3-en-1-ol (**3ab**): **Condition II** 70% yield, 111 mg; colorless oil; ^{1}H NMR (400 MHz, $CDCl_3$) δ 7.30 (m, 7H), 7.21 (m, 1H), 7.10 (t, *J* = 8.0 Hz, 2H), 7.05 (d, *J* = 8.0 Hz, 1H), 6.99 (d, *J* = 8.0 Hz, 2H), 6.92 (dd, *J* = 4.0 Hz, *J* = 8.0 Hz, 1H), 6.46 (d, *J* = 16.0 Hz, 1H), 6.16 (m, 1H), 4.76 (dd, *J* = 4.0 Hz, *J* = 8.0 Hz, 1H), 2.63 (m, 2H), 2.14 (br, 1H); ^{13}C NMR (101 MHz, $CDCl_3$) δ 157.3, 157.1, 146.0, 137.1, 133.5, 129.8, 129.7, 128.5, 127.3, 126.1, 125.5, 123.2, 120.6, 118.8, 117.9, 116.3, 73.3, 43.0; HRMS (ESI): calcd for $C_{22}H_{19}O$ $(M-H_2O+H)^+$ 299.1430, found 299.1430.

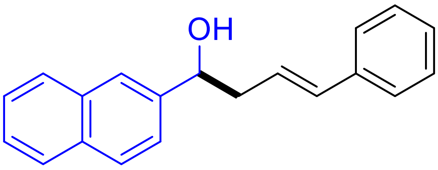

(*E*)-1-(Naphthalen-2-yl)-4-phenylbut-3-en-1-ol (**3ac**): **Condition II** 77% yield, 106 mg; white solid, m.p. 98-100 ˚C; ^{1}H NMR (400 MHz, $CDCl_3$) δ 7.85 (m, 4H), 7.50 (m, 3H), 7.34 (d, *J* = 4.0 Hz, 2H), 7.29 (t, *J* = 6.0 Hz, 2H), 7.21 (t, *J* = 8.0 Hz, 1H), 6.53 (d, *J* = 16.0 Hz, 1H), 6.22 (m, 1H), 4.98 (t, *J* =

6.0 Hz, 1H), 2.74 (m, 2H), 2.20 (br, 1H); ^{13}C NMR (101 MHz, $CDCl_3$) δ 141.3, 137.2, 133.6, 133.3, 133.0, 128.6, 128.3, 128.0, 127.7, 127.4, 126.20, 126.19, 125.9, 125.8, 124.5, 124.0, 73.9, 43.1; These data are in accordance with the literature.[9]

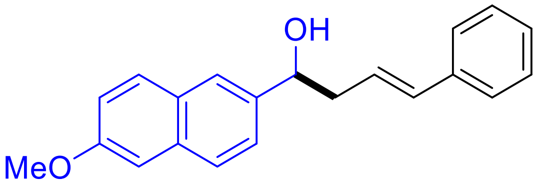

(*E*)-1-(6-methoxynaphthalen-2-yl)-4-phenylbut-3-en-1-ol (**3ad**): **Condition II** 73% yield, 111 mg; white solid, m.p. 129-131˚C; ^{1}H NMR (400 MHz, $CDCl_3$) δ 7.73 (t, *J* = 8.0 Hz, 3H), 7.48 (d, *J* = 12 Hz, 1H), 7.30 (m, 4H), 7.20 (t, *J* = 8.0 Hz, 1H), 7.15 (d, *J* = 8.0 Hz, 2H), 6.52 (d, *J* = 16.0 Hz, 1H), 6.22 (m, 1H), 4.94 (t, *J* = 6.0 Hz, 1H), 3.92 (s, 3H), 2.74 (t, *J* = 8.0 Hz, 2H), 2.17 (br, 1H); ^{13}C NMR (101 MHz, $CDCl_3$) δ 157.7, 139.0, 137.2, 134.1, 133.4, 129.4, 128.7, 128.5, 127.3, 127.1, 126.2, 125.9, 124.6, 124.4, 119.0, 105.7, 73.8, 55.3, 43.0; HRMS (ESI): calcd for $C_{21}H_{19}O$ $(M-H_2O+H)^+$ 287.1430, found 287.1428.

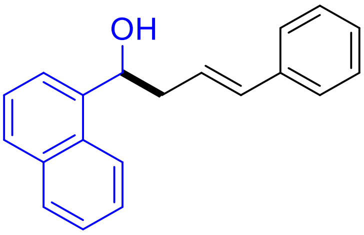

(*E*)-1-(Naphthalen-1-yl)-4-phenylbut-3-en-1-ol (**3ae**): **Condition II** 77% yield, 106 mg; colorless oil; ^{1}H NMR (400 MHz, $CDCl_3$) δ 8.08 (d, *J* = 8.0 Hz, 1H), 7.87 (d, *J* = 8.0 Hz, 1H), 7.77 (d, *J* = 8.0 Hz, 1H), 7.67 (d, *J* = 4.0 Hz, 1H), 7.49 (m, 3H), 7.33 (d, *J* = 8.0 Hz, 2H), 7.28 (t, *J* = 8.0 Hz, 2H), 7.20 (t, *J* = 8.0 Hz, 1H), 6.52 (d, *J* = 16.0 Hz, 1H), 6.29 (m, 1H), 5.56 (dd, *J* = 4.0 Hz, *J* = 8.0 Hz, 1H), 2.87 (m, 1H), 2.73 (m, 1H), 2.31 (br, 1H); ^{13}C NMR (101 MHz, $CDCl_3$) δ 139.4, 137.2, 133.8, 133.2, 130.2, 128.9, 128.5, 128.0, 127.3, 126.3, 126.1, 126.0, 125.5, 125.4, 123.0, 122.8, 70.4, 42.1; HRMS (ESI): calcd for $C_{20}H_{17}$ $(M-H_2O+H)^+$ 257.1325, found 257.1327.

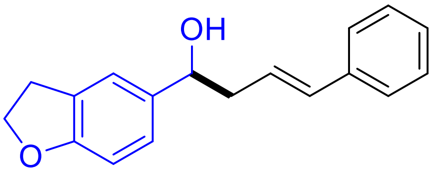

(*E*)-1-(2,3-Dihydrobenzofuran-5-yl)-4-phenylbut-3-en-1-ol (**3af**): **Condition II** 74% yield, 99 mg; white solid, m.p. 82-84˚C; ^{1}H NMR (400 MHz, $CDCl_3$) δ 7.31 (m, 4H), 7.21 (m, 2H), 7.10 (d, *J* = 8.0 Hz, 1H), 6.75 (d, *J* = 8.0 Hz, 1H), 6.49 (d, *J* = 16.0 Hz, 1H), 6.19 (m, 1H), 4.72 (t, *J* = 8.0 Hz, 1H), 4.56 (t, *J* = 8.0 Hz, 2H), 3.20 (t, *J* = 10.0 Hz, 2H), 2.64 (t, *J* = 6.0 Hz, 2H), 2.02 (br, 1H); ^{13}C NMR (101 MHz, $CDCl_3$) δ 159.6, 137.2, 136.1, 133.1, 128.5, 127.2, 126.2, 126.1, 125.8, 122.5, 109.0, 73.7, 71.3, 43.1, 29.7; HRMS (ESI): calcd for $C_{18}H_{17}O$ $(M-H_2O+H)^+$ 249.1274, found 249.1271.

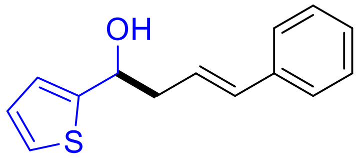

(*E*)-4-Phenyl-1-(thiophen-2-yl)but-3-en-1-ol (**3ag**): **Condition II** 75% yield, 86 mg; colorless oil; ^{1}H NMR (400 MHz, $CDCl_3$) δ 7.33 (d, *J* = 8.0 Hz, 2H), 7.28 (t, *J* = 8.0 Hz, 2H), 7.21 (m, 2H), 6.97 (m, 2H), 6.50 (d, *J* = 16.0 Hz, 2H), 6.21 (m, 1H), 5.03 (t, *J* = 6.0 Hz, 1H), 2.75 (d, *J* = 8.0 Hz, 2H), 2.37 (br, 1H); ^{13}C NMR (101 MHz, $CDCl_3$) δ 147.8, 137.1, 133.7, 128.5, 127.3, 126.6, 126.2, 125.2, 124.6, 123.7, 69.7, 43.0; These data are in accordance with the literature.[9]

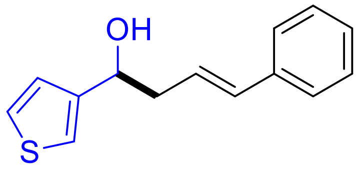

(*E*)-4-Phenyl-1-(thiophen-3-yl)but-3-en-1-ol (**3ah**): **Condition II** 52% yield, 60 mg; white solid, m.p. 83-85 ˚C; ^{1}H NMR (400 MHz, $CDCl_3$) δ 7.31 (m, 5H), 7.22 (m, 2H), 7.10 (dd, *J* = 4.0 Hz, *J* = 8.0 Hz, 1H), 6.49 (d, *J* = 16.0 Hz, 1H), 6.19 (m, 1H), 4.89 (t, *J* = 6.0 Hz, 1H), 2.68 (m, 2H), 2.14 (br, 1H); ^{13}C NMR (101 MHz, $CDCl_3$) δ 145.4, 137.1, 133.5, 128.5, 127.3, 126.13, 126.11, 125.6, 120.8, 70.0, 42.2; HRMS (ESI): calcd for $C_{14}H_{13}S$ $(M-H_2O+H)^+$ 213.0732, found 213.0732.

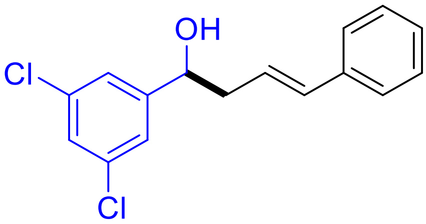

(*E*)-1-(3,5-Dichlorophenyl)-4-phenylbut-3-en-1-ol (**3ai**): **Condition II** 35% yield, 51 mg; colorless oil; ^{1}H NMR (400 MHz, $CDCl_3$) δ 7.32 (m, 7H), 7.23 (t, *J* = 8.0 Hz, 1H), 6.51 (d, *J* = 16.0 Hz, 1H), 6.15 (m, 1H), 4.75 (dd, *J* = 8.0 Hz, *J* = 4.0 Hz, 1H), 2.60 (m, 2H), 2.18 (br, 1H); ^{13}C NMR (101 MHz, $CDCl_3$) δ 147.3, 136.8, 135.0, 134.4, 128.63, 128.59, 127.6, 126.2, 124.6, 124.4, 72.4, 43.0; HRMS (ESI): calcd for $C_{16}H_{13}Cl_2$ $(M-H_2O+H)^+$ 275.0389, found 275.0382.

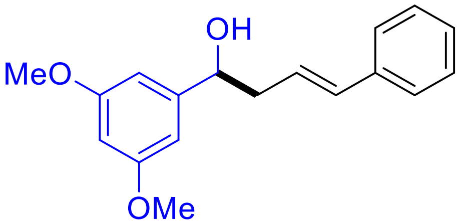

(*E*)-1-(3,5-Dimethoxyphenyl)-4-phenylbut-3-en-1-ol (**3aj**): **Condition II** 82% yield, 117 mg; colorless oil; ^{1}H NMR (400 MHz, $CDCl_3$) δ 7.30 (m, 4H), 7.20 (t, *J* = 8.0 Hz, 1H), 6.54 (d, *J* = 2.2 Hz, 2H), 6.48 (d, *J* = 16.0 Hz, 1H), 6.38 (t, *J* = 2.0 Hz, 1H), 6.20 (m, 1H), 4.72 (t, *J* = 6.0 Hz, 1H), 3.77 (s, 6H), 2.63 (t, *J* = 6.0 Hz, 2H), 2.24 (br, 1H); ^{13}C NMR (101 MHz, $CDCl_3$) δ 160.8, 146.5, 137.2, 133.3, 128.5, 127.3, 126.1, 125.9, 103.7, 99.5, 73.7, 55.3, 42.9; HRMS (ESI): calcd for $C_{18}H_{21}O_3$ $(M+H)^+$

285.1485, found 285.1484.

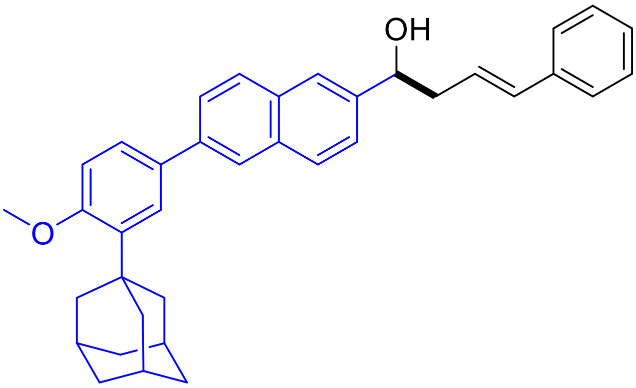

(*E*)-1-(6-(3-(Adamantan-1-yl)-4-methoxyphenyl)naphthalen-2-yl)-4-phenylbut-3-en-1-ol (**3ak**): **Condition II** 52% yield, 134 mg; white solid, m.p. 177-179 °C; ^{1}H NMR (400 MHz, $CDCl_3$) δ 7.97 (s, 1H), 7.88 (t, *J* = 8.0 Hz, 2H), 7.83 (s, 1H), 7.73 (d, *J* = 8.0 Hz, 1H), 7.59 (s, 1H), 7.52 (d, *J* = 8.0 Hz, 2H), 7.34 (d, *J* = 4.0 Hz, 2H), 7.28 (t, *J* = 8.0 Hz, 2H), 7.21 (t, *J* = 6.0 Hz, 1H), 6.98 (d, *J* = 8.0 Hz, 1H), 6.53 (d, *J* = 16.0 Hz, 1H), 6.23 (m, 1H), 4.98 (t, *J* = 6.0 Hz, 1H), 3.89 (s, 3H), 2.76 (t, *J* = 8.0 Hz, 2H), 2.19 (s, 7H), 2.10 (s, 3H), 1.80 (s, 6H); ^{13}C NMR (101 MHz, $CDCl_3$) δ 158.7, 141.1, 139.1, 139.0, 137.3, 133.6, 133.5, 133.23, 132.20, 128.6, 128.5, 128.4, 127.5, 126.3, 126.1, 126.0, 125.9, 125.7, 124.9, 124.44, 124.38, 112.2, 74.0, 55.3, 43.1, 40.7, 37.30, 37.26, 29.2; HRMS (ESI): calcd for $C_{37}H_{37}O$ $(M-H_2O+H)^+$ 497.2839, found 497.2834.

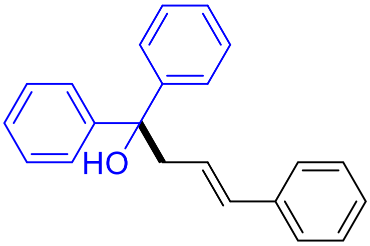

(*E*)-1,1,4-triphenylbut-3-en-1-ol (**3al**): **Condition I** 49% yield, 74 mg; **Condition II** 45% yield, 68 mg; white solid, m.p. 92-93 °C; ^{1}H NMR (400 MHz, $CDCl_3$) δ 7.48 (d, *J* = 8.0 Hz, 4H), 7.33 (t, *J* = 6.0 Hz, 4H), 7.23 (m, 7H), 6.57 (d, *J* = 16.0 Hz, 1H), 6.04 (m, 1H), 3.22 (d, *J* = 8.0 Hz, 2H), 2.57 (s, 1H) ; ^{13}C NMR (101 MHz, $CDCl_3$) δ 146.5, 136.9, 135.4, 128.5, 128.2, 127.5, 126.9, 126.2, 126.0, 124.6, 46.0; These data are in accordance with the literature.[9]

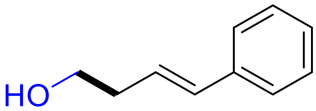

(*E*)-4-Phenylbut-3-en-1-ol (**3am**): **Condition II** 31% yield, 23 mg; colorless oil; ^{1}H NMR (400 MHz, $CDCl_3$) δ 7.36 (d, *J* = 8.0 Hz, 2H), 7.30 (t, *J* = 8.0 Hz, 2H), 7.22 (t, *J* = 8.0 Hz, 1H), 6.50 (d, *J* = 16.0 Hz, 1H), 6.21 (m, 1H), 3.77 (t, *J* = 6.0 Hz, 2H), 2.50 (dd, *J* = 12.0 Hz, *J* = 8.0 Hz, 2H), 1.63 (br, 1H); ^{13}C NMR (101 MHz, $CDCl_3$) δ 137.2, 132.9, 128.5, 127.3, 126.3, 126.1, 62.0, 36.4; These data are in accordance with the literature. [11]

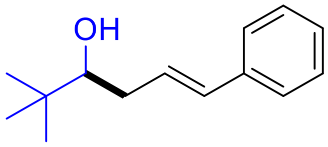

(*E*)-2,2-Dimethyl-6-phenylhex-5-en-3-ol (**3an**): **Condition II** 59% yield, 60 mg; colorless oil; ^{1}H NMR (400 MHz, $CDCl_3$) δ 7.36 (d, *J* = 4.0 Hz, 2H), 7.29 (t, *J* = 8.0 Hz, 2H), 7.20 (t, *J* = 8.0 Hz, 1H), 6.48 (d, *J* = 16.0 Hz, 1H), 6.27 (m, 1H), 3.34 (d, *J* = 12.0 Hz, 1H), 2.50 (m, 1H), 2.15 (m, 1H), 1.67 (br, 1H), 0.95 (s, 9H); ^{13}C NMR (101 MHz, $CDCl_3$) δ 137.3, 132.7, 128.5, 128.1, 127.2, 126.0, 78.7, 35.7, 34.7, 25.8; These data are in accordance with the literature. [12]

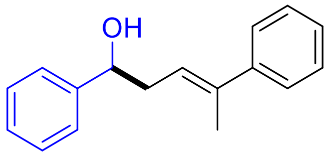

(*E*)-1,4-Diphenylpent-3-en-1-ol (**3ao**): **Condition II** 63% yield, 75 mg; colorless oil; ^{1}H NMR (400 MHz, $CDCl_3$) δ 7.29 (m, 1H), 5.78 (*t*, *J* = 8.0 Hz, 1H), 4.78 (*t*, *J* = 6.0 Hz, 1H), 2.66 (m, 2H), 2.19 (br, 1H), 1.97 (s, 3H); ^{13}C NMR (101 MHz, $CDCl_3$) δ 144.1, 143.5, 137.9, 128.4, 128.1, 127.5, 126.8, 125.8, 125.6, 123.3, 74.1, 38.7, 16.0; These data are in accordance with the literature.[13]

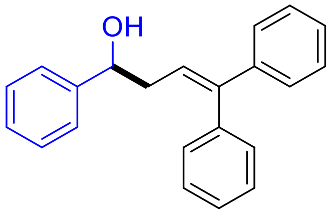

1,4,4-Triphenylbut-3-en-1-ol (**3ap**): **Condition II** 67% yield, 101 mg; colorless oil; ^{1}H NMR (400 MHz, $CDCl_3$) δ 7.29 (m, 8H), 7.20 (m, 5H), 7.06 (d, *J* = 4.0 Hz, 2H), 6.10 (t, *J* = 8.0 Hz, 1H), 4.76 (dd, *J* = 8.0 Hz, *J* = 4.0 Hz, 1H), 2.57 (m, 2H), 2.01 (br, 1H); ^{13}C NMR (101 MHz, $CDCl_3$) δ 144.2, 143.9, 142.4, 139.7, 129.8, 128.4, 128.2, 128.0, 127.5, 127.2, 127.04, 127.00, 125.8, 124.7, 74.3, 39.4; HRMS (ESI): calcd for $C_{22}H_{19}$ $(M-H_2O+H)^+$ 283.1481, found 283.1476.

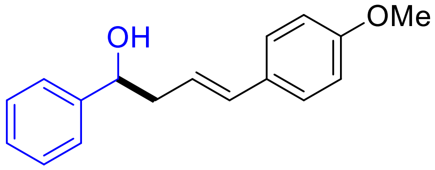

(*E*)-4-(4-Methoxyphenyl)-1-phenylbut-3-en-1-ol (**3aq**): **Condition II** 76% yield, 97 mg; colorless oil; ^{1}H NMR (400 MHz, $CDCl_3$) δ 7.37 (m, 4H), 7.28 (t, *J* = 8.0 Hz, 3H), 6.83 (d, *J* = 8.0 Hz, 2H), 6.45 (d, *J* = 16.0 Hz, 1H), 6.05 (m, 1H), 4.79 (t, *J* = 4.0 Hz, 1H), 3.79 (s, 3H), 2.63 (m, 2H), 2.11 (br, 1H); ^{13}C NMR (101 MHz, $CDCl_3$) δ 159.0, 143.9, 132.9, 130.0, 128.4, 127.5, 127.3, 125.8, 123.5, 113.9, 73.7, 55.3, 43.1; These data are in accordance with the literature.[14]

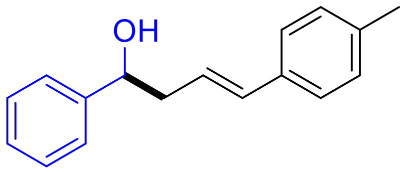

(*E*)-1-Phenyl-4-(*p*-tolyl)but-3-en-1-ol (**3ar**): **Condition II** 71% yield, 85 mg; white solid, m.p. 107-109 ˚C; ^{1}H NMR (400 MHz, $CDCl_3$) δ 7.36 (m, 4H), 7.28 (m, 1H), 7.23 (d, *J* = 8.0 Hz, 2H), 7.10 (d, *J* = 8.0 Hz, 2H), 6.46 (d, *J* = 16.0 Hz, 1H), 6.14 (m, 1H), 4.79 (t, *J* = 6.0 Hz, 1H), 2.64 (m, 2H), 2.32 (s, 3H), 2.06 (br, 1H); ^{13}C NMR (101 MHz, $CDCl_3$) δ 143.9, 137.1, 134.4, 133.3, 129.2, 128.4, 127.5, 126.0, 125.8, 124.7, 73.7, 43.1, 21.1; These data are in accordance with the literature. [15]

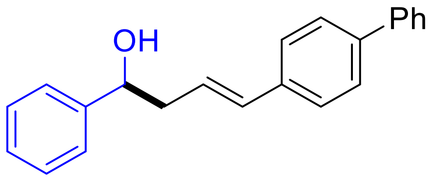

(*E*)-4-([1,1'-Biphenyl]-4-yl)-1-phenylbut-3-en-1-ol (**3as**): **Condition II** 64% yield, 96 mg; white solid, m.p. 157-159 ˚C; ^{1}H NMR (400 MHz, $CDCl_3$) δ 7.59 (d, *J* = 8.0 Hz, 2H), 7.54 (d, *J* = 8.0 Hz, 2H), 7.38 (m, 10H), 6.54 (d, *J* = 16.0 Hz, 1H), 6.26 (m, 1H), 4.84 (t, *J* = 8.0 Hz, 1H), 2.70 (t, *J* = 6.0 Hz, 2H), 2.07 (br, 1H); ^{13}C NMR (101 MHz, $CDCl_3$) δ 143.9, 140.7, 140.1, 136.2, 132.9, 128.8, 128.5, 127.6, 127.24, 127.20, 126.9, 126.6, 126.0, 125.8, 73.8, 43.1; HRMS (ESI): calcd for $C_{22}H_{19}$ $(M-H_2O+H)^+$ 283.1481, found 283.1480.

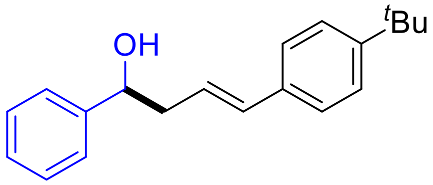

(*E*)-4-(4-(*tert*-Butyl)phenyl)-1-phenylbut-3-en-1-ol (**3at**): **Condition II** 72% yield, 101 mg; colorless oil; ^{1}H NMR (400 MHz, $CDCl_3$) δ 7.35 (m, 5H), 7.29 (m, 4H), 6.47 (d, *J* = 16.0 Hz, 1H), 6.14 (m, 1H), 4.78 (dd, *J* = 8.0 Hz, *J* = 4.0 Hz, 1H), 2.63 (m, 2H), 2.13 (br, 1H), 1.30 (s, 9H); ^{13}C NMR (101 MHz, $CDCl_3$) δ 150.4, 143.9, 134.4, 133.2, 128.4, 127.5, 125.9, 125.8, 125.4, 125.0, 73.7, 43.1, 34.5, 31.3; These data are in accordance with the literature. [16]

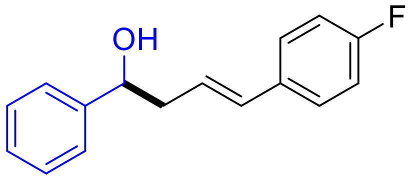

(*E*)-4-(4-Fluorophenyl)-1-phenylbut-3-en-1-ol (**3au**): **Condition II** 79% yield, 96 mg; white solid, m.p. 81-83 ˚C; ^{1}H NMR (400 MHz, $CDCl_3$) δ 7.35 (m, 4H), 7.28 (m, 3H), 6.97 (t, *J* = 8.0 Hz, 2H), 6.44 (d, *J* = 16.0 Hz, 1H), 6.11 (m, 1H), 4.79 (t, *J* = 6.0 Hz, 1H), 2.64 (t, *J* = 8.0 Hz, 2H), 2.14 (br, 1H); ^{13}C NMR (101 MHz, $CDCl_3$) δ 162.1 (d, J_{F-C} = 248.5 Hz), 143.9, 133.4 (d, J_{F-C} = 3.0 Hz), 132.1, 128.5, 127.6, 127.5, 125.8, 125.7 (d, J_{F-C} = 2.0 Hz), 115.4 (d, J_{F-C} = 22.0 Hz), 73.7, 42.9; HRMS (ESI): calcd

for $C_{16}H_{14}F$ (M-H_2O+H)$^+$ 225.1074, found 225.1071.

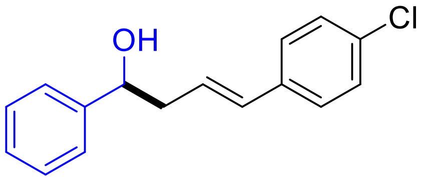

(*E*)-4-(4-Chlorophenyl)-1-phenylbut-3-en-1-ol (**3av**): **Condition II** 87% yield, 113 mg; white solid, m.p. 108-110 °C; ^{1}H NMR (400 MHz, $CDCl_3$) δ 7.36 (m, 4H), 7.30 (m, 1H), 7.25 (s, 4H), 6.44 (d, *J* = 16.0 Hz, 1H), 6.19 (m, 1H), 4.82 (t, *J* = 6.0 Hz, 1H), 2.66 (t, *J* = 6.0 Hz, 2H), 2.03 (br, 1H); ^{13}C NMR (101 MHz, $CDCl_3$) δ 143.8, 135.7, 132.9, 132.1, 128.7, 128.5, 127.7, 127.4, 126.7, 125.8, 73.7, 42.9; These data are in accordance with the literature. [16]

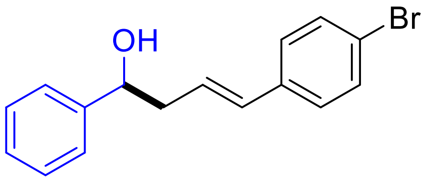

(*E*)-4-(4-Bromophenyl)-1-phenylbut-3-en-1-ol (**3aw**): **Condition II** 90% yield, 136 mg; white solid, m.p. 55-57 °C; ^{1}H NMR (400 MHz, $CDCl_3$) δ 7.40 (m, 6H), 7.30 (m, 1H), 7.19 (d, *J* = 8.0 Hz, 2H), 6.42 (d, *J* = 16.0 Hz, 1H), 6.20 (m, 1H), 4.82 (t, *J* = 6.0 Hz, 1H), 2.66 (t, *J* = 6.0 Hz, 2H), 2.03 (br, 1H); ^{13}C NMR (101 MHz, $CDCl_3$) δ 143.8, 136.2, 132.1, 131.6, 128.5, 127.7, 126.9, 125.8, 121.0, 73.7, 42.9; HRMS (ESI): calcd for $C_{16}H_{14}Br$ (M-H_2O+H)$^+$ 285.0273, found 285.0275.

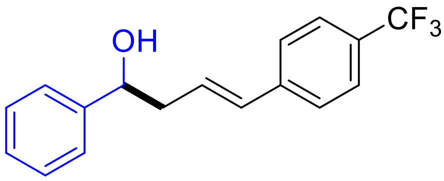

(*E*)-1-Phenyl-4-(4-(trifluoromethyl)phenyl)but-3-en-1-ol (**3ax**): **Condition II** 68% yield, 99 mg; colorless oil; ^{1}H NMR (400 MHz, $CDCl_3$) δ 7.53 (d, *J* = 8.0 Hz, 2H), 7.40 (d, *J* = 8.0 Hz, 2H), 7.32 (m, 5H), 6.49 (d, *J* = 16.0 Hz, 1H), 6.31 (m, 1H), 4.82 (t, *J* = 6.0 Hz, 1H), 2.68 (d, *J* = 8.0 Hz, 2H), 2.14 (br, 1H); ^{13}C NMR (101 MHz, $CDCl_3$) δ 143.8, 140.7, 131.8, 129.0 (q, $J_{F\text{-}C}$ = 32.0 Hz), 128.9, 128.5, 127.8, 126.2, 125.8, 125.4 (q, $J_{F\text{-}C}$ = 4.0 Hz), 124.2 (q, $J_{F\text{-}C}$ = 271.0 Hz), 73.8, 42.9; These data are in accordance with the literature. [17]

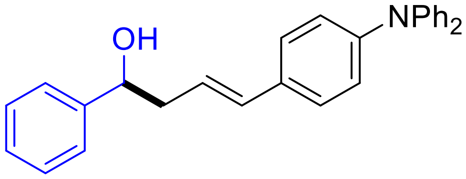

(*E*)-4-(4-(Diphenylamino)phenyl)-1-phenylbut-3-en-1-ol (**3ay**): **Condition II** 64% yield, 125 mg; yellow oil; ^{1}H NMR (400 MHz, $CDCl_3$) δ 7.36 (m, 4H), 7.29 (d, *J* = 4.0 Hz, 1H), 7.23 (m, 6H), 7.07 (d, *J* = 8.0 Hz, 4H), 7.00 (t, *J* = 8.0 Hz, 4H), 6.45 (d, *J* = 16.0 Hz, 1H), 6.09 (m, 1H), 4.79 (dd, *J* = 8.0 Hz, *J*

= 4.0 Hz, 1H), 2.63 (m, 2H), 2.01 (br, 1H); ^{13}C NMR (101 MHz, $CDCl_3$) δ 147.6, 147.1, 143.9, 132.9, 131.5, 129.2, 128.4, 127.6, 127.0, 125.8, 124.3, 124.2, 123.8, 122.8, 73.7, 43.2; HRMS (ESI): calcd for $C_{28}H_{26}NO$ $(M+H)^+$ 392.2009, found 392.2004.

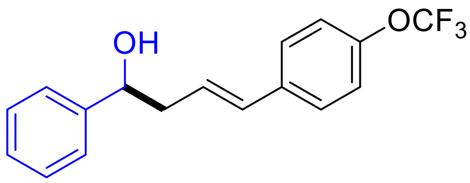

(*E*)-1-Phenyl-4-(4-(trifluoromethoxy)phenyl)but-3-en-1-ol (**3az**): **Condition II** 47% yield, 72 mg; white solid, m.p. 115-117 ˚C; ^{1}H NMR (400 MHz, $CDCl_3$) δ 7.33 (m, 7H), 7.13 (d, *J* = 8.0 Hz, 2H), 6.46 (d, *J* = 16.0 Hz, 1H), 6.19 (m, 1H), 4.82 (t, *J* = 6.0 Hz, 1H), 2.66 (t, *J* = 8.0 Hz, 2H), 2.07 (br, 1H); ^{13}C NMR (101 MHz, $CDCl_3$) δ 148.3, 143.8, 136.0, 131.8, 128.5, 127.7, 127.3, 127.1, 125.8, 121.0, 120.5 (q, $J_{F\text{-}C}$ = 258.6 Hz), 73.8, 42.9; HRMS (ESI): calcd for $C_{17}H_{14}F_3O$ $(M\text{-}H_2O+H)^+$ 291.0991, found 291.0992.

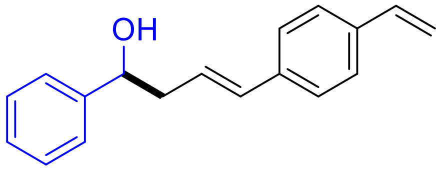

(*E*)-1-Phenyl-4-(4-vinylphenyl)but-3-en-1-ol (**3ba**): **Condition II** 75% yield, 94 mg; white solid, m.p. 102-104 ˚C; ^{1}H NMR (400 MHz, $CDCl_3$) δ 7.36 (m, 6H), 7.29 (m, 3H), 6.68 (dd, *J* = 20.0 Hz, *J* = 12.0 Hz, 1H), 6.47 (d, *J* = 16.0 Hz, 1H), 6.20 (m, 1H), 5.72 (d, *J* = 16.0 Hz, 1H), 5.22 (d, *J* = 12.0 Hz, 1H), 4.80 (t, *J* = 6.0 Hz, 1H), 2.66 (t, *J* = 6.0 Hz, 2H), 2.11 (br, 1H); ^{13}C NMR (101 MHz, $CDCl_3$) δ 143.9, 136.7, 136.6, 136.4, 133.0, 128.5, 127.6, 126.4, 126.3, 125.9, 125.8, 113.5, 73.7, 43.1; HRMS (ESI): calcd for $C_{18}H_{17}$ $(M\text{-}H_2O+H)^+$ 233.1325, found 233.1320.

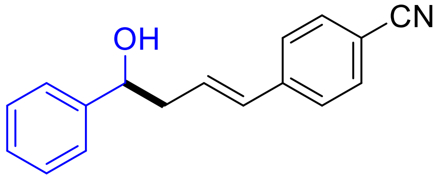

(*E*)-4-(4-Hydroxy-4-phenylbut-1-en-1-yl)benzonitrile (**3bb**): **Condition II** 44% yield, 55 mg; white solid, m.p. 89-91 ˚C; ^{1}H NMR (400 MHz, $CDCl_3$) δ 7.55 (d, *J* = 8.0 Hz, 2H), 7.37 (m, 6H), 7.30 (m, 1H), 6.48 (d, *J* = 16.0 Hz, 1H), 6.36 (m, 1H), 4.84 (t, *J* = 6.0 Hz, 1H), 2.70 (t, *J* = 6.0 Hz, 2H), 2.13 (br, 1H); ^{13}C NMR (101 MHz, $CDCl_3$) δ 143.7, 141.7, 132.3, 131.5, 130.5, 128.6, 127.8, 126.6, 125.7, 119.0, 110.4, 73.7, 42.8; HRMS (ESI): calcd for $C_{17}H_{16}NO$ $(M+H)^+$ 250.1226, found 250.1225.

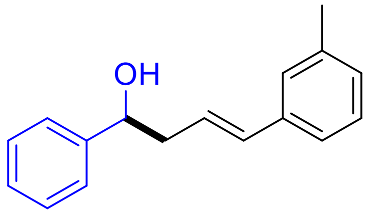

(*E*)-1-Phenyl-4-(*m*-tolyl)but-3-en-1-ol (**3bc**): **Condition II** 92% yield, 110 mg; white solid, m.p. 67-69 ˚C; ^{1}H NMR (400 MHz, $CDCl_3$) δ 7.35 (m, 4H), 7.27 (t, *J* = 8.0 Hz, 1H), 7.17 (m, 3H), 7.02 (d, *J* = 8.0 Hz, 1H), 6.45 (d, *J* = 16.0 Hz, 1H), 6.17 (m, 1H), 4.78 (t, *J* = 6.0 Hz, 1H), 2.63 (t, *J* = 8.0 Hz, 2H), 2.32 (s, 3H), 2.17 (br, 1H); ^{13}C NMR (101 MHz, $CDCl_3$) δ 143.9, 138.0, 137.1, 133.5, 128.41, 128.38, 128.1, 127.5, 126.9, 125.8, 125.6, 123.3, 73.7, 43.1, 21.3; HRMS (ESI): calcd for $C_{17}H_{17}$ $(M-H_2O+H)^+$ 221.1325, found 221.1324.

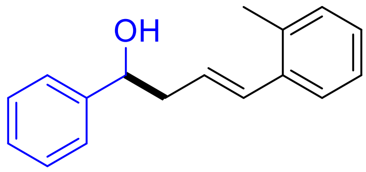

(*E*)-1-Phenyl-4-(*o*-tolyl)but-3-en-1-ol (**3bd**): **Condition II** 89% yield, 106 mg; white solid, m.p. 91-93 ˚C; ^{1}H NMR (400 MHz, $CDCl_3$) δ 7.35 (m, 5H), 7.27 (t, *J* = 6.0 Hz, 1H), 7.12 (m, 3H), 6.64 (d, *J* = 16.0 Hz, 1H), 6.04 (m, 1H), 4.78 (t, *J* = 6.0 Hz, 1H), 2.67 (t, *J* = 8.0 Hz, 2H), 2.28 (s, 3H), 2.19 (br, 1H); ^{13}C NMR (101 MHz, $CDCl_3$) δ 143.8, 136.3, 135.1, 131.3, 130.2, 128.4, 127.5, 127.2, 127.1, 126.0, 125.8, 125.5, 73.7, 43.2, 19.7; These data are in accordance with the literature.[14]

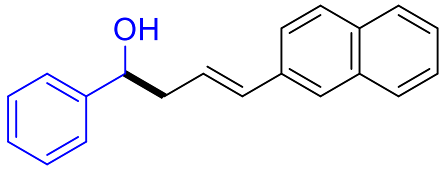

(*E*)-4-(Naphthalen-2-yl)-1-phenylbut-3-en-1-ol (**3be**): **Condition II** 90% yield, 123 mg; white solid, m.p. 120-122 ˚C; ^{1}H NMR (400 MHz, $CDCl_3$) δ 7.77 (t, *J* = 10.0 Hz, 3H), 7.68 (s, 1H), 7.56 (d, *J* = 12.0 Hz, 1H), 7.41 (m, 6H), 7.30 (t, *J* = 6.0 Hz, 1H), 6.65 (d, *J* = 16.0 Hz, 1H), 6.33 (m, 1H), 4.85 (t, *J* = 6.0 Hz, 1H), 2.72 (t, *J* = 6.0 Hz, 2H), 2.11 (br, 1H); ^{13}C NMR (101 MHz, $CDCl_3$) δ 143.9, 134.6, 133.6, 133.5, 132.9, 128.5, 128.1, 127.9, 127.6, 126.3, 126.2, 125.9, 125.8, 125.7, 123.5, 73.8, 43.2; HRMS (ESI): calcd for $C_{20}H_{17}$ $(M-H_2O+H)^+$ 257.1325, found 257.1322.

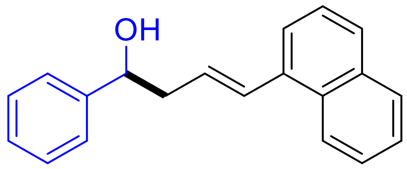

(*E*)-4-(Naphthalen-1-yl)-1-phenylbut-3-en-1-ol (**3bf**): **Condition II** 71% yield, 97 mg; colorless oil; ^{1}H NMR (400 MHz, $CDCl_3$) δ 8.00 (t, *J* = 6.0 Hz, 1H), 7.81 (t, *J* = 4.0 Hz, 1H), 7.73 (d, *J* = 8.0 Hz, 1H), 7.50 (d, *J* = 8.0 Hz, 1H), 7.46 (m, 2H), 7.38 (m, 5H), 7.28 (t, *J* = 8.0 Hz, 1H), 7.16 (d, *J* = 16.0 Hz, 1H),

6.17 (m, 1H), 4.85 (t, *J* = 6.0 Hz, 1H), 2.77 (t, *J* = 6.0 Hz, 2H), 2.15 (br, 1H); ^{13}C NMR (101 MHz, $CDCl_3$) δ 143.9, 135.0, 133.5, 131.0, 130.7, 129.11, 129.09, 128.5, 128.4, 127.63, 127.59, 125.9, 125.7, 125.6, 123.9, 123.7, 73.8, 43.3; These data are in accordance with the literature. [16]

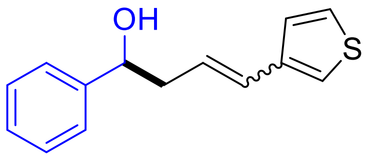

1-Phenyl-4-(thiophen-3-yl)but-3-en-1-ol (**3bg**): **Condition II** 61% yield, 70 mg; E/Z = 2/1; white solid, m.p. 59-61 ˚C; ^{1}H NMR (400 MHz, $CDCl_3$) δ 7.36 (m, 6H), 7.27 (m, 3H), 7.16 (d, *J* = 4.0 Hz, 1.5H), 7.09 (d, *J* = 4.0 Hz, 1.5H), 6.50 (d, *J* = 16.0 Hz, 1.5 H), 6.04 (m, 1H), 5.65 (m, 0.5H), 4.82 (dd, *J* = 4.0 Hz, *J* = 8.0 Hz, 0.5H), 4.77 (t, *J* = 4.0 Hz, *J* = 8.0 Hz, 1.0H), 2.87 (m, 0.5H), 2.74 (m, 0.5H), 2.61 (m, 2H), 2.14 (br, 1.5H); ^{13}C NMR (101 MHz, $CDCl_3$) δ 143.93, 143.87, 139.8, 138.3, 128.5, 128.4, 127.7, 127.59, 127.56, 126.9, 125.9, 125.84, 125.76, 125.7, 125.1, 124.9, 123.1, 121.4, 74.0, 73.7, 42.9, 38.6; HRMS (ESI): calcd for $C_{14}H_{13}S$ $(M-H_2O+H)^+$ 213.0732, found 213.0729.

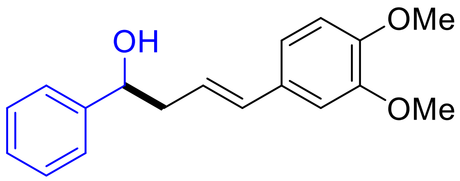

(*E*)-4-(3,4-Dimethoxyphenyl)-1-phenylbut-3-en-1-ol (**3bh**): **Condition II** 58% yield, 82 mg; colorless oil; ^{1}H NMR (400 MHz, $CDCl_3$) δ 7.37 (m, 4H), 7.28 (t, *J* = 8.0 Hz, 1H), 6.88 (m, 2H), 6.79 (d, *J* = 8.0 Hz, 1H), 6.44 (d, *J* = 16.0 Hz, 1H), 6.08 (m, 1H), 4.80 (dd, *J* = 4.0 Hz, *J* = 8.0 Hz, 1H), 3.88 (s, 3H), 3.86 (s, 3H), 2.65 (m, 2H), 2.18 (br, 1H); ^{13}C NMR (101 MHz, $CDCl_3$) δ 149.0, 148.6, 143.9, 133.0, 130.3, 128.4, 127.5, 125.7, 123.9, 119.2, 111.1, 108.7, 73.7, 55.9, 55.8, 43.1; HRMS (ESI): calcd for $C_{18}H_{19}O_2$ $(M-H_2O+H)^+$ 267.1380, found 267.1377.

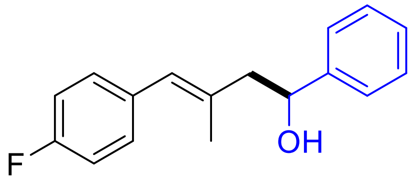

(*E*)-4-(4-Fluorophenyl)-3-methyl-1-phenylbut-3-en-1-ol (**3bi**): **Condition II** 38% yield, 49 mg; colorless oil; E/Z = 6/1; ^{1}H NMR (400 MHz, $CDCl_3$) δ 7.34 (m, 5H), 7.19 (t, *J* = 6.0 Hz, 2H), 6.97 (t, *J* = 8.0 Hz, 2H), 6.43 (s, 1H), 4.93 (dd, *J* = 8.0 Hz, *J* = 12.0 Hz, 1H), 2.86 (dd, *J* = 8.0 Hz, *J* = 12.0 Hz, 1H), 2.42 (dd, *J* = 4.0 Hz, *J* = 12.0 Hz, 1H), 1.96 (s, 3H); ^{13}C NMR (101 MHz, $CDCl_3$) δ 161.4 (d, J_{F-C} = 244.0 Hz), 144.2, 135.2, 133.8 (d, J_{F-C} = 4.0 Hz), 130.3 (d, J_{F-C} = 7.0 Hz), 128.5, 128.1, 127.7, 125.7, 114.9 (d, J_{F-C} = 21.0 Hz), 72.3, 42.2, 24.1; HRMS (ESI): calcd for $C_{17}H_{17}FO$ $(M-H_2O+H)^+$ 239.1231, found 239.1232.

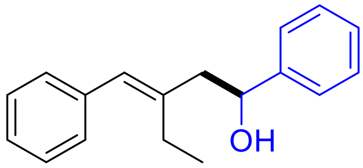

(*E*)-3-Benzylidene-1-phenylpentan-1-ol (**3bj**): **Condition II** 23% yield, 29 mg; colorless oil; E/Z = 7/1; ^{1}H NMR (400 MHz, $CDCl_3$) δ 7.28 (m, 9H), 7.18 (t, *J* = 8.0 Hz, 1H) 6.49 (s, 1H), 4.89 (q, *J* = 4.0 Hz, 1H), 2.91 (dd, *J* = 8.0 Hz, *J* = 12.0 Hz, 1H), 2.51 (dd, *J* = 8.0 Hz, *J* = 12.0 Hz, 1H), 2.27 (q, *J* = 8.0 Hz, 2H), 1.90 (br, 1H), 1.16 (t, *J* = 6.0 Hz, 3H); ^{13}C NMR (101 MHz, $CDCl_3$) δ 144.3, 140.6, 138.0, 128.8, 128.5, 128.4, 128.2, 127.5, 126.2, 125.7, 72.4, 40.6, 30.0, 12.8; HRMS (ESI): calcd for $C_{18}H_{20}O$ $(M-H_2O+H)^+$ 235.1481, found 235.1483.

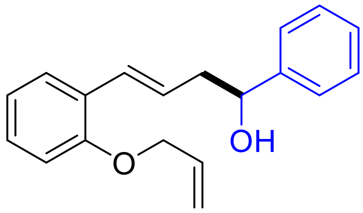

(*E*)-4-(2-(Allyloxy)phenyl)-1-phenylbut-3-en-1-ol (**3bk**): **Condition II** 50% yield, 70 mg; colorless oil; ^{1}H NMR (400 MHz, $CDCl_3$) δ 7.37 (m, 5H), 7.27 (t, *J* = 8.0 Hz, 1H), 7.16 (t, *J* = 8.0 Hz, 1H), 6.87 (m, 3H), 6.20 (m, 1H), 6.05 (m, 1H), 5.40 (d, *J* = 16.0 Hz, 1H), 5.28 (d, *J* = 12.0 Hz, 1H), 4.80 (t, *J* = 6.0 Hz, 1H), 4.54 (d, *J* = 8.0 Hz, 2H), 2.68 (m, 2H), 2.11 (br, 1H); ^{13}C NMR (101 MHz, $CDCl_3$) δ 155.4, 144.0, 133.4, 128.4, 128.2, 127.5, 126.7, 126.6, 126.5, 125.8, 120.8, 117.3, 112.3, 73.7, 69.1, 43.5; HRMS (ESI): calcd for $C_{19}H_{20}O_2$ $(M-H_2O+H)^+$ 263.1430, found 263.1431.

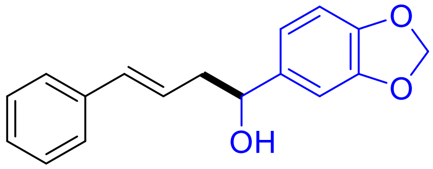

(*E*)-1-(Benzo[*d*][1,3]dioxol-5-yl)-4-phenylbut-3-en-1-ol (**3bl**): **Condition III** 31% yield, 42 mg; white solid, m.p. 66-67 °C; ^{1}H NMR (400 MHz, $CDCl_3$) δ 7.32 (m, 4H), 7.21 (t, *J* = 8.0 Hz, 1H), 6.91 (s, 1H), 6.83 (d, *J* = 8.0 Hz, 1H), 6.78 (d, *J* = 8.0 Hz, 1H), 6.49 (d, *J* = 16.0 Hz, 1H), 6.18 (m, 1H), 5.96 (s, 2H), 4.72 (t, *J* = 8.0 Hz, 1H), 2.63 (t, *J* = 6.0 Hz, 2H), 2.02 (s, 1H); ^{13}C NMR (101 MHz, $CDCl_3$) δ 147.8, 147.0, 138.0, 137.2, 133.4, 128.5, 127.3, 126.1, 125.8, 119.2, 108.1, 106.4, 101.0, 73.6, 43.1; HRMS (ESI): calcd for $C_{17}H_{16}O_3$ $(M-H_2O+H)^+$ 251.1067, found 251.1068.

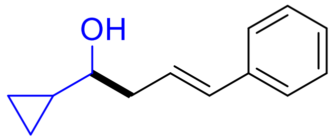

(*E*)-1-Cyclopropyl-4-phenylbut-3-en-1-ol (**3bm**): **Condition I** 56% yield, 53 mg; **Condition II** 54% yield, 51 mg; colorless oil; ^{1}H NMR (400 MHz, $CDCl_3$) δ 7.36 (d, *J* = 8.0 Hz, 2H), 7.29 (t, *J* = 8.0 Hz, 2H), 7.21 (t, *J* = 8.0 Hz, 1H), 6.49 (d, *J* = 16.0 Hz, 1H), 6.30 (m, 1H), 3.02 (m, 1H), 2.58 (m, 1H), 2.48

(m, 1H), 1.75 (br, 1H), 0.98 (m, 1H), 0.56 (m, 2H), 0.34 (m, 1H), 0.25 (m, 1H); ^{13}C NMR (101 MHz, $CDCl_3$) δ 137.4, 132.6, 128.5, 127.1, 126.5, 126.1, 40.9, 17.4, 2.8, 2.6; HRMS (ESI): calcd for $C_{13}H_{15}$ $(M-H_2O+H)^+$ 171.1168, found 171.1168.

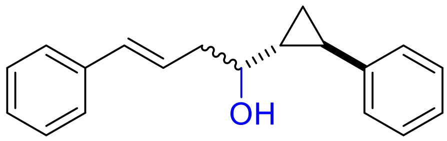

(*E*)-4-Phenyl-1-(2-phenylcyclopropyl)but-3-en-1-ol (**3bn**): **Condition I** 45% yield, 59 mg; **Condition II** 42% yield, 56 mg; colorless oil; Isomers of products can be isolated by column. Isomer I: ^{1}H NMR (400 MHz, $CDCl_3$) δ 7.36 (d, *J* = 8.0 Hz, 2H), 7.31 (d, *J* = 8.0 Hz, 2H), 7.23 (t, *J* = 8.0 Hz, 3H), 7.15 (t, *J* = 6.0 Hz, 1H), 7.08 (d, *J* = 8.0 Hz, 2H), 6.50 (d, *J* = 16.0 Hz, 1H), 6.30 (m, 1H), 3.36 (dd, *J* = 4.0 Hz, *J* = 12.0 Hz, 1H), 2.61 (m, 1H), 2.52 (m, 1H), 1.97 (dd, *J* = 8.0 Hz, *J* = 12.0 Hz, 1H), 1.67 (br, 1H), 1.34 (m, 1H), 0.98 (t, *J* = 6.0 Hz, 2H); ^{13}C NMR (101 MHz, $CDCl_3$) δ 142.5, 137.3, 133.0, 128.5, 128.3, 127.2, 126.1, 126.0, 125.9, 125.6, 74.7, 40.8, 29.0, 21.0, 13.4; Isomer II: ^{1}H NMR (400 MHz, $CDCl_3$) δ 7.31 (t, *J* = 8.0 Hz, 4H), 7.23 (t, *J* = 6.0 Hz, 3H), 7.16 (t, *J* = 6.0 Hz, 1H), 7.05 (d, *J* = 8.0 Hz, 2H), 6.46 (d, *J* = 16.0 Hz, 1H), 6.27 (m, 1H), 3.32 (dd, *J* = 4.0 Hz, *J* = 8.0 Hz, 1H), 2.57 (m, 2H), 1.86 (m, 1H), 1.67 (br, 1H), 1.31 (m, 1H), 1.02 (m, 2H); ^{13}C NMR (101 MHz, $CDCl_3$) δ 142.2, 137.2, 133.1, 128.5, 128.3, 127.2, 126.1, 126.0, 125.9, 125.7, 75.0, 41.1, 29.1, 21.1, 13.5; HRMS (ESI): calcd for $C_{19}H_{20}O$ $(M-H_2O+H)^+$ 247.1481, found 247.1480.

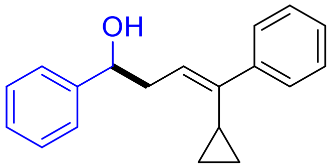

(*E*)-4-Cyclopropyl-1,4-diphenylbut-3-en-1-ol (**3bo**): **Condition I** 58% yield, 77 mg; **Condition II** 61% yield, 81 mg; colorless oil; ^{1}H NMR (400 MHz, $CDCl_3$) δ 7.40 (d, *J* = 8.0 Hz, 2H), 7.35 (t, *J* = 8.0 Hz, 2H), 7.27 (m, 5H), 7.19 (m, 1H), 5.70 (t, *J* = 8.0 Hz, 1H), 4.82 (dd, *J* = 5.5 Hz, *J* = 8.0 Hz, 1H), 2.90 (m, 1H), 2.80 (m, 1H), 2.17 (br, 1H), 1.66 (m, 1H), 0.75 (m, 2H), 0.26 (m, 2H); ^{13}C NMR (101 MHz, $CDCl_3$) δ 144.2, 143.8, 142.1, 128.4, 127.7, 127.5, 127.4, 126.5, 126.4, 125.9, 74.2, 38.4, 11.6, 6.59, 6.56; HRMS (ESI): calcd for $C_{19}H_{21}O$ $(M+H)^+$ 265.1587, found 265.1584.

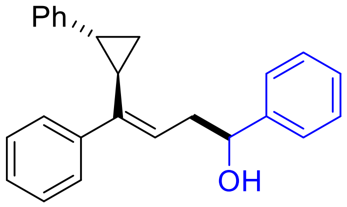

(*E*)-1,4-Diphenyl-4-(2-phenylcyclopropyl)but-3-en-1-ol (**3bp**): **Condition I** 32% yield, 54 mg; **Condition II** 38% yield, 65 mg; colorless oil; ^{1}H NMR (400 MHz, $CDCl_3$) δ 7.29 (m, 13H), 7.07 (t, *J* = 8.0 Hz, 2H), 5.82 (t, *J* = 8.0 Hz, 1H), 4.83 (t, *J* = 6.0 Hz, 1H), 2.83 (m, 2H), 1.95 (m, 2H), 1.78 (m, 1H), 1.27 (m, 1H), 0.96 (m, 1H); ^{13}C NMR (101 MHz, $CDCl_3$) δ 144.1, 142.8, 142.6, 141.84, 141.79, 128.4, 127.9, 127.6, 127.5, 127.4, 127.3, 127.1, 127.0, 126.7, 126.4, 126.1, 126.0, 125.9, 125.6,

125.6, 74.2, 74.1, 38.7, 38.6, 24.9, 24.7, 24.33, 24.30, 17.4; HRMS (ESI): calcd for $C_{25}H_{24}O$ $(M+H)^+$ 323.1794, found 323.1797.

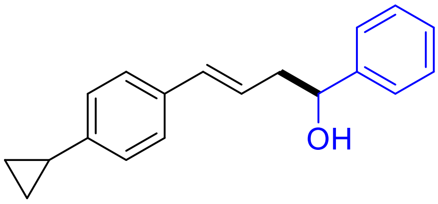

(*E*)-4-(4-Cyclopropylphenyl)-1-phenylbut-3-en-1-ol (**3bq**): **Condition I** 56% yield, 74 mg; **Condition II** 60% yield, 79 mg; white solid, m.p. 93-94 °C; ^{1}H NMR (400 MHz, $CDCl_3$) δ 7.36 (m, 4H), 7.28 (t, *J* = 6.0 Hz, 1H), 7.23 (d, *J* = 8.0 Hz, 2H), 6.99 (d, *J* = 8.0 Hz, 2H), 6.46 (d, *J* = 16.0 Hz, 1H), 6.13 (m, 1H), 4.79 (t, *J* = 6.0 Hz, 1H), 2.63 (m, 2H), 2.07 (s, 1H), 1.87 (m, 1H), 0.95 (dd, *J* = 4.0 Hz, *J* = 16.0 Hz, 2H), 0.67 (dd, *J* = 4.0 Hz, *J* = 8.0 Hz, 2H); ^{13}C NMR (101 MHz, $CDCl_3$) δ 143.9, 143.3, 134.4, 133.3, 128.4, 127.5, 126.1, 125.79, 125.76, 124.7, 73.7, 43.1, 15.2, 9.2; HRMS (ESI): calcd for $C_{19}H_{20}O$ $(M+H)^+$ 247.1481, found 247.1484.

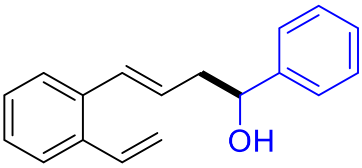

(*E*)-1-Phenyl-4-(2-vinylphenyl)but-3-en-1-ol (**3br**): **Condition I** 41% yield, 51 mg; **Condition II** 75% yield, 94 mg; colorless oil; ^{1}H NMR (400 MHz, $CDCl_3$) δ 7.42 (m, 1H), 7.36 (m, 5H), 7.28 (t, *J* = 6.0 Hz, 1H), 7.20 (m, 2H), 6.92 (dd, *J* = 12.0 Hz, *J* = 16.0 Hz, 1H), 6.71 (d, *J* = 16.0 Hz, 1H), 6.02 (m, 1H), 5.57 (d, *J* = 16.0 Hz, 1H), 5.28 (d, *J* = 12.0 Hz, 1H), 4.80 (t, *J* = 6.0 Hz, 1H), 2.68 (t, *J* = 6.0 Hz, 2H), 2.15 (br, 1H); ^{13}C NMR (101 MHz, $CDCl_3$) δ 143.8, 135.8, 135.6, 134.9, 131.2, 128.43, 128.39, 127.7, 127.6, 127.3, 126.5, 126.2, 125.9, 116.10, 116.07, 73.8, 43.2; HRMS (ESI): calcd for $C_{18}H_{18}O$ $(M+H)^+$ 233.1325, found 233.1327.

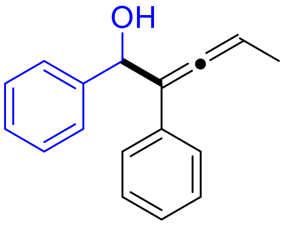

1,2-Diphenylpenta-2,3-dien-1-ol (**5a**): **Condition II** 73% yield, 86 mg; **Condition III** 59% yield, 70 mg; pale yellow oil; *dr* 1/1; ^{1}H NMR (400 MHz, $CDCl_3$) δ 7.43 (m, 2H) , 7.37 (m, 2H), 7.32 (t, *J* = 4.0 Hz, 5H), 7.22 (m, 1H), 6.37 (m, 1H), 5.21 (dd, *J* = 40.0 Hz, *J* = 4.0 Hz, 1H), 2.35 (br, 1H), 1.70 (d, *J* = 4.0 Hz, 3H); ^{13}C NMR (101 MHz, $CDCl_3$) δ 201.3, 200.7, 141.79, 141.76, 134.7, 134.6, 128.6, 128.43, 128.39, 127.9, 127.8, 127.14, 127.09, 126.84, 126.79, 126.6, 126.5, 108.0, 107.6, 97.9, 97.3, 75.2, 75.1, 15.0, 14.5; HRMS (ESI): calcd for $C_{17}H_{17}O$ $(M+H)^+$ 237.1274, found 237.1274.

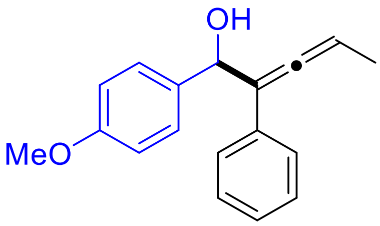

1-(4-Methoxyphenyl)-2-phenylpenta-2,3-dien-1-ol (**5b**): **Condition II** 68% yield, 91 mg; pale yellow oil; *dr* 1/1; ^{1}H NMR (400 MHz, $CDCl_3$) δ 7.33 (m, 6H), 7.20 (m, 1H), 6.89 (dd, *J* = 12.0 Hz, *J* = 4.0 Hz, 2H), 6.36 (m, 1H), 5.15 (dd, *J* = 40.0 Hz, *J* = 4.0 Hz, 1H), 3.80 (s, 3H), 2.25 (br, 1H), 1.69 (t, *J* = 2.0 Hz, 3H); ^{13}C NMR (101 MHz, $CDCl_3$) δ 201.1, 200.5, 159.3, 159.2, 134.8, 134.7, 133.93, 133.90, 128.6, 127.9, 127.8, 127.1, 127.0, 126.80, 126.75, 113.81, 113.76, 108.1, 107.8, 97.9, 97.3, 74.8, 74.7, 55.2, 15.1, 14.7; HRMS (ESI): calcd for $C_{18}H_{19}O_2$ $(M+H)^+$ 267.1380, found 267.1381.

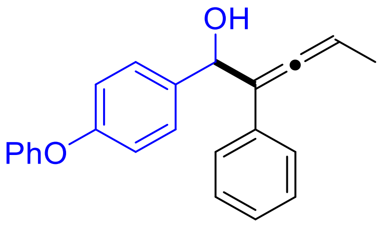

1-(4-Phenoxyphenyl)-2-phenylpenta-2,3-dien-1-ol (**5c**): **Condition II** 58% yield, 95 mg; pale yellow oil; *dr* 1/1; ^{1}H NMR (400 MHz, $CDCl_3$) δ 7.39 (dd, *J* = 8.0 Hz, *J* = 4.0 Hz, 2H), 7.32 (m, 6H), 7.21 (m, 1H), 7.10 (t, *J* = 6.0 Hz, 1H), 7.00 (m, 4H), 6.36 (m, 1H), 5.19 (dd, *J* = 40.0 Hz, *J* = 4.0 Hz, 1H), 2.31 (br, 1H), 1.73 (d, *J* = 4.0 Hz, 3H); ^{13}C NMR (101 MHz, $CDCl_3$) δ 201.2, 200.7, 157.2, 156.92, 156.87, 136.6, 134.61, 134.56, 129.7, 128.7, 128.1, 128.0, 127.2, 127.1, 126.80, 126.77, 123.3, 118.86, 118.85, 118.8, 118.7, 108.0, 107.7, 98.1, 97.5, 74.73, 74.69, 15.2, 14.7; HRMS (ESI): calcd for $C_{23}H_{21}O_2$ $(M+H)^+$ 329.1536, found 329.1536.

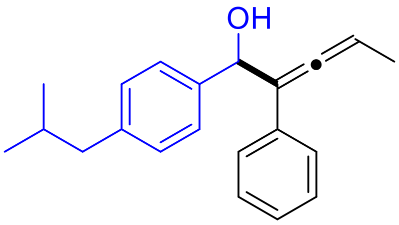

1-(4-Isobutylphenyl)-2-phenylpenta-2,3-dien-1-ol (**5d**): **Condition II** 55% yield, 80 mg; pale yellow oil; *dr* 1/1; ^{1}H NMR (400 MHz, $CDCl_3$) δ 7.32 (m, 6H), 7.21 (m, 1H), 7.14 (dd, *J* = 8.0 Hz, *J* = 4.0 Hz, 2H), 6.36 (m, 1H), 5.17 (d, *J* = 40.0 Hz, 1H), 2.47 (d, *J* = 8.0 Hz, 2H), 2.30 (br, 1H), 1.85 (m, 1H), 1.70 (t, *J* = 2.0 Hz, 3H), 0.89 (d, *J* = 8.0 Hz, 6H); ^{13}C NMR (101 MHz, $CDCl_3$) δ 201.2, 200.7, 141.42, 141.37, 139.0, 134.8, 134.7, 131.5, 129.2, 129.1, 128.6, 127.1, 127.0, 126.83, 126.78, 126.4, 126.3, 108.1, 107.8, 97.9, 97.3, 75.1, 75.0, 45.1, 30.2, 22.3, 15.1, 14.7; HRMS (ESI): calcd for $C_{21}H_{25}O$ $(M+H)^+$ 293.1900, found 293.1901.

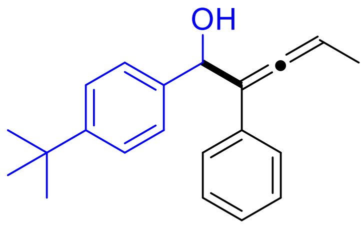

1-(4-(*tert*-Butyl)phenyl)-2-phenylpenta-2,3-dien-1-ol (**5e**): **Condition II** 65% yield; 95 mg; pale yellow oil; *dr* 1/1; ^{1}H NMR (400 MHz, $CDCl_3$) δ 7.35 (m, 8H), 7.21 (m, 1H), 6.36 (m, 1H), 5.18 (dd, *J* = 40.0 Hz, *J* = 4.0 Hz, 1H) , 2.24 (br, 1H), 1.71 (dd, *J* = 4.0 Hz, *J* = 1.8 Hz, 3H), 1.32 (s, 9H); ^{13}C NMR (101 MHz, $CDCl_3$) δ 201.2, 200.7, 150.9, 150.8, 138.8, 134.8, 134.7, 128.6, 127.1, 127.0, 126.9, 126.8, 126.4, 126.2, 125.4, 125.3, 108.0, 107.7, 97.9, 97.3, 75.0, 74.9, 34.5, 31.3, 15.1, 14.7; HRMS (ESI): calcd for $C_{21}H_{25}O$ $(M+H)^+$ 293.1900, found 293.1902.

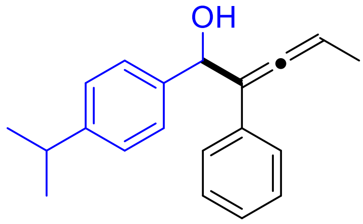

1-(4-Isopropylphenyl)-2-phenylpenta-2,3-dien-1-ol (**5f**): **Condition II** 57% yield, 79 mg; pale yellow oil; *dr* 1/1; ^{1}H NMR (400 MHz, $CDCl_3$) δ 7.33 (m, 6H), 7.22 (m, 3H), 6.37 (m, 1H), 5.17 (d, *J* = 40.0 Hz, 1H), 2.91 (m, 1H), 2.28 (br, 1H), 1.70 (dd, *J* = 4.0 Hz, *J* = 2.0 Hz, 3H), 1.25 (d, *J* = 8.0 Hz, 6H); ^{13}C NMR (101 MHz, $CDCl_3$) δ 201.2, 200.6, 148.61, 148.55, 139.1, 134.8, 134.7, 128.6, 127.1, 127.0, 126.84, 126.79, 126.64, 126.55, 126.51, 126.47, 108.0, 107.7, 97.9, 97.3, 75.1, 75.0, 33.8, 24.0, 15.1, 14.7; HRMS (ESI): calcd for $C_{20}H_{23}O$ $(M+H)^+$ 279.1743, found 279.1741.

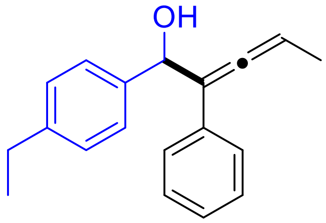

1-(4-Ethylphenyl)-2-phenylpenta-2,3-dien-1-ol (**5g**): **Condition II** 61% yield, 81 mg; pale yellow oil; *dr* 1/1; ^{1}H NMR (400 MHz, $CDCl_3$) δ 7.33 (m, 6H), 7.21 (m, 3H), 6.36 (m, 1H), 5.18 (dd, *J* = 40.0 Hz, *J* = 4.0 Hz, 1H), 2.65 (q, *J* = 8.0 H, 2H), 2.23 (br, 1H), 1.70 (t, *J* = 2.0 Hz, 3H), 1.24 (t, *J* = 8.0 Hz, 3H); ^{13}C NMR (101 MHz, $CDCl_3$) δ 201.2, 200.6, 144.0, 143.9, 139.0, 134.8, 134.7, 128.6, 128.0, 127.9, 127.09, 127.05, 126.9, 126.8, 126.7, 126.5, 108.1, 107.7, 97.9, 97.3, 75.1, 75.0, 28.6, 15.5, 15.1, 14.7; HRMS (ESI): calcd for $C_{19}H_{21}O$ $(M+H)^+$ 265.1587, found 265.1587.

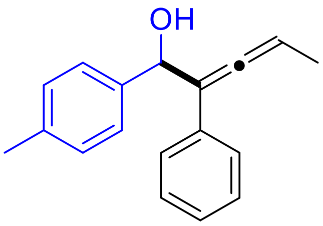

2-Phenyl-1-(*p*-tolyl)penta-2,3-dien-1-ol (**5h**): **Condition II** 64% yield, 80 mg; pale yellow oil; *dr* 1/1; ^{1}H NMR (400 MHz, $CDCl_3$) δ 7.32 (m, 6H), 7.20 (m, 3H), 6.37 (m, 1H), 5.17 (dd, *J* = 40.0 Hz, *J* = 4.0 Hz, 1H), 2.35 (s, 3H), 2.20 (br, 1H), 1.70 (t, *J* = 2.0 Hz, 3H); ^{13}C NMR (101 MHz, $CDCl_3$) δ 201.2, 200.6, 138.8, 137.6, 137.5, 134.8, 134.7, 129.13, 129.09, 128.6, 127.10, 127.05, 126.84, 126.79, 126.6, 126.5, 108.1, 107.7, 97.9, 97.3, 75.1, 75.0, 21.1, 15.1, 14.6; HRMS (ESI): calcd for $C_{18}H_{19}O$ $(M+H)^+$ 251.1430, found 251.1429.

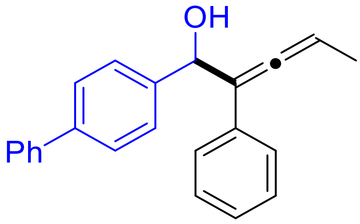

1-([1,1'-Biphenyl]-4-yl)-2-phenylpenta-2,3-dien-1-ol (**5i**): **Condition II** 69% yield, 108 mg; pale yellow oil; *dr* 1/1; ^{1}H NMR (400 MHz, $CDCl_3$) δ 7.59 (m, 4H), 7.49 (dd, *J* = 8.0 Hz, *J* = 4.0 Hz, 2H), 7.43 (t, *J* = 8.0 Hz, 2H), 7.33 (m, 5H), 7.22 (m, 1H), 6.38 (m, 1H), 5.25 (dd, *J* = 40.0 Hz, *J* = 2.0 Hz, 1H), 2.30 (br, 1H), 1.74 (d, *J* = 2.8 Hz, 3H); ^{13}C NMR (101 MHz, $CDCl_3$) δ 201.3, 200.8, 140.83, 140.81, 140.78, 140.7, 134.7, 134.6, 128.74, 128.66, 127.3, 127.2, 127.14, 127.07, 127.04, 126.95, 126.86, 126.81, 107.9, 107.5, 98.0, 97.4, 75.0, 74.9, 15.0, 14.6; HRMS (ESI): calcd for $C_{23}H_{21}O$ $(M+H)^+$ 313.1587, found 313.1588.

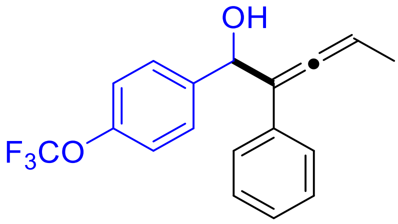

2-Phenyl-1-(4-(trifluoromethoxy)phenyl)penta-2,3-dien-1-ol (**5j**): **Condition II** 44% yield, 70 mg; pale yellow oil; *dr* 1/1; ^{1}H NMR (400 MHz, $CDCl_3$) δ 7.47 (dd, *J* = 8.0 Hz, *J* = 4.0 Hz, 2H), 7.31 (m, 4H), 7.22 (m, 3H), 6.36 (m, 1H), 5.23 (d, *J* = 36.0 Hz, 1H), 2.36 (br, 1H), 1.70 (s, 3H); ^{13}C NMR (101 MHz, $CDCl_3$) δ 201.3, 200.9, 148.7, 140.5, 140.4, 134.38, 134.36, 128.7, 128.0, 127.9, 127.32, 127.28, 126.83, 126.79, 120.9, 120.5 (q, $J_{F\text{-}C}$ = 256.0 Hz), 107.6, 107.3, 98.1, 97.6, 74.5, 74.4, 14.8, 14.4; HRMS (ESI): calcd for $C_{18}H_{16}F_3O_2$ $(M+H)^+$ 321.1097, found 321.1098.

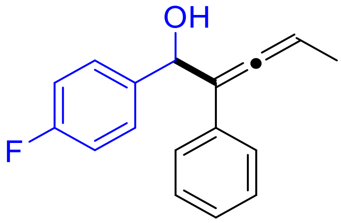

1-(4-Fluorophenyl)-2-phenylpenta-2,3-dien-1-ol (**5k**): **Condition II** 51% yield, 65 mg; pale yellow oil; *dr* 1/1; ^{1}H NMR (400 MHz, $CDCl_3$) δ 7.39 (m, 2H), 7.31 (m, 4H), 7.22 (m, 1H), 7.04 (dt, *J* = 12.0 Hz, *J* = 4.0 Hz, 2H), 6.36 (m, 1H), 5.19 (dd, *J* = 36.0 Hz, *J* = 4.0 Hz, 1H), 2.25 (br, 1H), 1.69 (s, 3H); ^{13}C NMR (101 MHz, $CDCl_3$) δ 201.2, 200.7, 162.4 (d, $J_{F\text{-}C}$ = 245.0 Hz), 137.5, 134.52, 134.47, 128.7, 128.2 (dd, $J_{F\text{-}C}$ = 9.0 Hz), 127.24, 127.20, 126.81, 126.77, 115.3 (dd, $J_{F\text{-}C}$ = 21.0 Hz), 107.9, 107.6, 98.1, 97.5, 74.6, 74.5, 14.9, 14.5; HRMS (ESI): calcd for $C_{17}H_{16}FO$ $(M+H)^+$ 255.1180, found 255.1176.

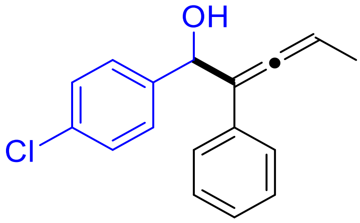

1-(4-Chlorophenyl)-2-phenylpenta-2,3-dien-1-ol (**5l**): **Condition II** 52% yield, 70 mg; pale yellow oil; *dr* 1/1; ^{1}H NMR (400 MHz, $CDCl_3$) δ 7.33 (m, 8H), 7.22 (m, 1H), 6.35 (m, 1H), 5.18 (d, *J* = 40.0 Hz, 1H), 2.34 (br, 1H), 1.69 (d, *J* = 4.0 Hz, 3H); ^{13}C NMR (101 MHz, $CDCl_3$) δ 201.3, 200.8, 140.30, 140.25, 134.43, 134.40, 133.54, 133.50, 128.7, 128.6, 128.53, 127.94, 127.88, 127.3, 127.2, 126.82, 126.78, 107.7, 107.3, 98.1, 97.5, 74.6, 74.5, 14.8, 14.4; HRMS (ESI): calcd for $C_{17}H_{14}Cl$ $(M-H_2O+H)^+$ 253.0779, found 253.0778.

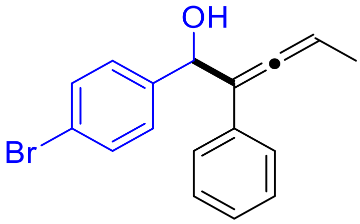

1-(4-Bromophenyl)-2-phenylpenta-2,3-dien-1-ol (**5m**): **Condition II** 45% yield, 71 mg; pale yellow oil; *dr* 1/1; ^{1}H NMR (400 MHz, $CDCl_3$) δ 7.46 (m, 2H), 7.33 (m, 6H), 7.22 (m, 1H), 6.36 (m, 1H), 5.20 (m, 1H), 2.28 (br, 1H), 1.69 (d, *J* = 3.2 Hz, 3H); ^{13}C NMR (101 MHz, $CDCl_3$) δ 201.3, 200.8, 140.84, 140.79, 134.42, 134.39, 131.51, 131.49, 128.7, 128.3, 128.2, 127.30, 127.25, 126.83, 126.79, 121.69, 121.66, 107.6, 107.2, 98.1, 97.5, 74.7, 74.5, 14.8, 14.4; HRMS (ESI): calcd for $C_{17}H_{14}Br$ $(M-H_2O+H)^+$ 297.0273, found 297.0275.

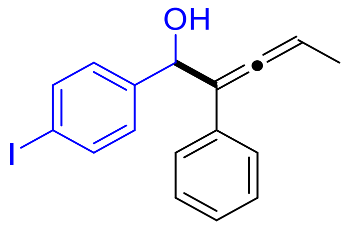

1-(4-Iodophenyl)-2-phenylpenta-2,3-dien-1-ol (**5n**): **Condition II** 28% yield, 51 mg; pale yellow oil; *dr* 1/1; ^{1}H NMR (400 MHz, $CDCl_3$) δ 7.69 (dd, *J* = 8.0 Hz, *J* = 2.0 Hz, 2H), 7.31 (m, 4H), 7.23 (m, 1H), 7.18 (dd, *J* = 8.0 Hz, *J* = 4.0 Hz, 2H), 6.36 (m, 1H), 5.16 (dd, *J* = 36.0 Hz, *J* = 4.0 Hz, 1H), 2.27 (br, 1H), 1.69 (d, *J* = 4.0 Hz, 3H); ^{13}C NMR (101 MHz, $CDCl_3$) δ 201.3, 200.8, 141.54, 141.48, 137.48, 137.45, 134.42, 134.39, 128.7, 128.6, 128.5, 127.30, 127.25, 126.84, 126.79, 107.6, 107.2, 98.1, 97.5, 93.34, 93.30, 74.8, 74.6, 14.8, 14.3; HRMS (ESI): calcd for $C_{17}H_{14}I$ $(M-H_2O+H)^+$ 345.0135, found 345.0137.

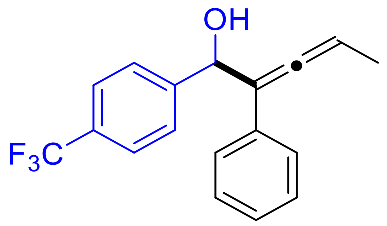

2-Phenyl-1-(4-(trifluoromethyl)phenyl)penta-2,3-dien-1-ol (**5o**): **Condition II** 47% yield, 72 mg; pale yellow oil; *dr* 1/1; ^{1}H NMR (400 MHz, $CDCl_3$) δ 7.62 (d, *J* = 8.0 Hz, 2H), 7.53 (d, *J* = 8.0 Hz, 2H), 7.31 (m, 4H), 7.23 (t, *J* = 6.0 Hz, 1H), 6.37 (m, 1H), 5.28 (d, *J* = Hz, 1H), 2.40 (br, 1H), 1.70 (d, *J* = 4.0 Hz, 3H); ^{13}C NMR (101 MHz, $CDCl_3$) δ 201.5, 201.0, 145.8, 145.7, 134.3, 130.0 (q, $J_{F\text{-}C}$ = 33.0 Hz), 128.7, 127.4, 127.3, 126.83, 126.80, 126.76, 125.4 (q, $J_{F\text{-}C}$ = 3.0 Hz), 124.1 (q, $J_{F\text{-}C}$ = 271.0 Hz), 107.4, 107.0, 98.1, 97.5, 74.8, 74.6, 14.6, 14.2; HRMS (ESI): calcd for $C_{18}H_{16}F_3O$ $(M+H)^+$ 305.1148, found 305.1154.

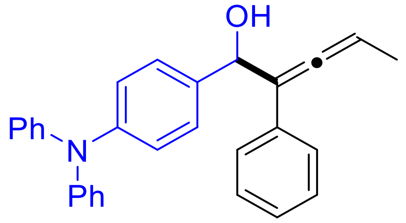

1-(4-(Diphenylamino)phenyl)-2-phenylpenta-2,3-dien-1-ol (**5p**): **Condition II** 42% yield; 85 mg; pale yellow oil; *dr* 1/1; ^{1}H NMR (400 MHz, $CDCl_3$) δ 7.29 (m, 6H), 7.22 (m, 4H), 7.07 (m, 6H), 7.00 (t, *J* = 8.0 Hz, 3H), 6.35 (m, 1H), 5.16 (dd, *J* = 36.0 Hz, *J* = 2.0 Hz, 1H), 2.27 (br, 1H), 1.76 (d, *J* = 4.0 Hz, 3H); ^{13}C NMR (101 MHz, $CDCl_3$) δ 201.2, 200.8, 147.7, 147.6, 147.5, 135.8, 135.7, 134.7, 134.6, 129.2, 128.6, 127.6, 127.5, 127.12, 127.10, 126.80, 126.77, 124.3, 123.72, 123.70, 122.8, 108.1, 107.8, 98.0, 97.5, 74.9, 74.8, 15.4, 14.9; HRMS (ESI): calcd for $C_{29}H_{26}NO$ $(M+H)^+$ 404.2009, found 404.2008.

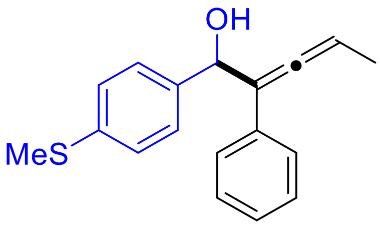

1-(4-(Methylthio)phenyl)-2-phenylpenta-2,3-dien-1-ol (**5q**): **Condition II** 51% yield, 72 mg; pale yellow oil; *dr* 1/1; ^{1}H NMR (400 MHz, $CDCl_3$) δ 7.33 (m, 6H), 7.23 (m, 3H), 6.36 (m, 1H), 5.16 (d, *J* = 40.0 Hz, 1H), 2.48 (s, 3H), 2.33 (br, 1H), 1.69 (dd, *J* = 4.0 Hz, *J* = 1.5 Hz, 3H); ^{13}C NMR (101 MHz, $CDCl_3$) δ 201.3, 200.6, 138.7, 138.0, 137.9, 134.62, 134.56, 128.6, 127.2, 127.13, 127.11, 127.0, 126.82, 126.77, 126.6, 126.5, 107.9, 107.5, 98.0, 97.4, 74.8, 74.7, 15.8, 15.0, 14.5; HRMS (ESI): calcd for $C_{18}H_{19}OS$ $(M+H)^+$ 283.1151, found 283.1148.

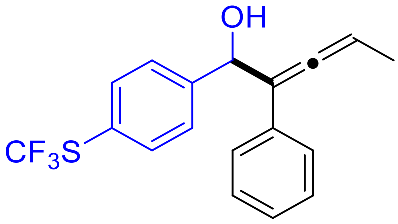

2-Phenyl-1-(4-((trifluoromethyl)thio)phenyl)penta-2,3-dien-1-ol (**5r**): **Condition II** 29% yield, 49 mg; pale yellow oil; *dr* 1/1; ^{1}H NMR (400 MHz, $CDCl_3$) δ 7.64 (d, *J* = 8.0 Hz, 2H), 7.48 (dd, *J* = 8.0 Hz, *J* = 2.4 Hz, 1H), 7.31 (m, 4H), 7.22 (t, *J* = 4.0 Hz, 1H), 6.36 (m, 1H), 5.26 (d, *J* = 32.0 Hz, 1H), 2.36 (br, 1H), 1.72 (t, *J* = 2.0 Hz, 3H); ^{13}C NMR (101 MHz, $CDCl_3$) δ 201.5, 201.1, 145.0, 144.9, 136.3, 134.3, 129.8 (q, $J_{F\text{-}C}$ = 254.0 Hz), 128.7, 127.59, 127.55, 127.4, 127.3, 126.84, 126.81, 123.6, 107.4, 107.1, 98.1, 97.6, 74.7, 74.6, 14.7, 14.3; HRMS (ESI): calcd for $C_{18}H_{16}F_3OS$ $(M+H)^+$ 337.0868, found 337.0863.

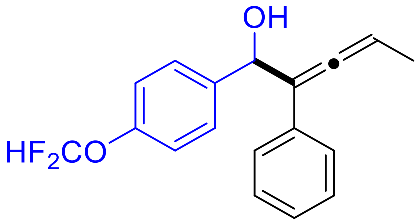

1-(4-(Difluoromethoxy)phenyl)-2-phenylpenta-2,3-dien-1-ol (**5s**): **Condition II** 45% yield, 68 mg; pale yellow oil; *dr* 1/1; ^{1}H NMR (400 MHz, $CDCl_3$) δ 7.42 (dd, *J* = 8.0 Hz, *J* = 4.0 Hz, 2H), 7.31 (m, 4H), 7.22 (m, 1H), 7.11 (dd, *J* = 8.0 Hz, *J* = 4.0 Hz, 2H), 6.50 (t, $J_{F\text{-}H}$ = 74.0 Hz, 1H), 6.36 (m, 1H), 5.21 (dd, *J* = 36.0 Hz, *J* = 2.0 Hz, 1H), 2.33 (br, 1H), 1.70 (d, *J* = 4.0 Hz, 3H); ^{13}C NMR (101 MHz, $CDCl_3$) δ 201.2, 200.7, 150.8, 138.99, 138.95, 134.5, 134.4, 128.7, 128.1, 128.0, 127.3, 127.2, 126.82, 126.78, 119.44, 119.41, 115.9 (t, $J_{F\text{-}C}$ = 258.0 Hz), 107.8, 107.4, 98.1, 97.6, 74.6, 74.5, 14.9, 14.5; HRMS (ESI): calcd for $C_{18}H_{17}F_2O_2$ $(M+H)^+$ 303.1191, found 303.1197.

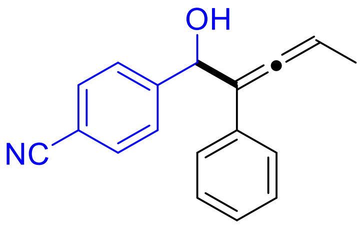

4-(1-Hydroxy-2-phenylpenta-2,3-dien-1-yl)benzonitrile (**5t**): **Condition II** 46% yield, 60 mg; pale yellow oil; *dr* 1/1; ^{1}H NMR (400 MHz, $CDCl_3$) δ 7.64 (d, *J* = 8.0 Hz, 2H), 7.54 (dd, *J* = 8.0 Hz, *J* = 4.0 Hz, 2H), 7.28 (m, 5H), 6.34 (m, 1H), 5.30 (dd, *J* = 28.0 Hz, *J* = 1.6 Hz, 1H), 2.39 (br, 1H), 1.71 (dd, *J* = 4.0 Hz, *J* = 2.0 Hz, 3H); ^{13}C NMR (101 MHz, $CDCl_3$) δ 201.8, 201.5, 147.3, 147.2, 134.08, 134.05, 132.2, 128.73, 128.70, 127.40, 127.37, 127.2, 127.1, 126.80, 126.76, 118.8, 111.4, 107.0, 106.6, 97.9, 97.4, 74.7, 74.5, 14.5, 14.1; HRMS (ESI): calcd for $C_{18}H_{16}NO$ $(M+H)^+$ 262.1226, found 262.1218.

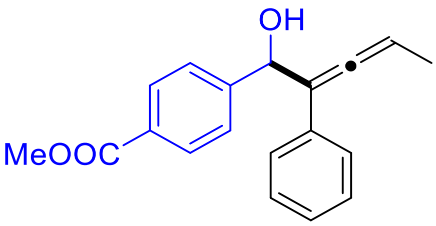

Methyl 4-(1-hydroxy-2-phenylpenta-2,3-dien-1-yl)benzoate (**5u**): **Condition II** 33% yield, 49 mg; pale yellow oil; *dr* 1/1; ^{1}H NMR (400 MHz, $CDCl_3$) δ 8.04 (d, *J* = 8.0 Hz, 2H), 7.51 (dd, *J* = 8.0 Hz, *J* = 4.0 Hz, 2H), 7.31 (m, 4H), 7.23 (t, *J* = 6.0 Hz, 1H), 6.36 (m, 1H), 5.30 (d, *J* = 32.0 Hz, 1H), 3.92 (s, 3H), 2.40 (br, 1H), 1.71 (d, *J* = 4.0 Hz, 3H); ^{13}C NMR (101 MHz, $CDCl_3$) δ 201.5, 201.0, 166.9, 147.0, 146.9, 134.4, 129.74, 129.71, 129.62, 129.58, 128.7, 127.32, 127.27, 126.84, 126.80, 126.5, 126.4, 107.5, 107.1, 98.1, 97.4, 75.0, 74.8, 52.1, 14.7, 14.3; HRMS (ESI): calcd for $C_{19}H_{19}O_3$ $(M+H)^+$ 295.1329, found 295.1323.

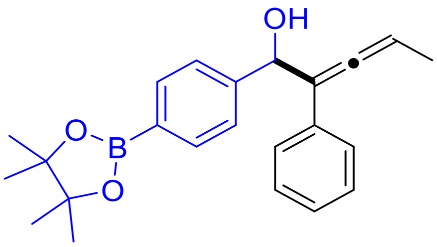

2-Phenyl-1-(4-(4,4,5,5-tetramethyl-1,3,2-dioxaborolan-2-yl)phenyl)penta-2,3-dien-1-ol (**5v**): **Condition II** 36% yield, 65 mg; pale yellow oil; *dr* 1/1; ^{1}H NMR (400 MHz, $CDCl_3$) δ 7.82 (dd, *J* = 2.4 Hz, *J* = 8.0 Hz, 2H), 7.44 (dd, *J* = 4.0 Hz, *J* = 8.0 Hz, 2H), 7.32 (m, 4H), 7.21 (m, 1H), 6.37 (m, 1H), 5.23 (d, *J* = 48.0 Hz, 1H), 2.26 (br, 1H), 1.69 (d, *J* = 4.0 Hz, 3H), 1.35 (s, 12H); ^{13}C NMR (101 MHz, $CDCl_3$) δ 201.5, 200.7, 144.93, 144.87, 134.94, 134.90, 134.7, 134.6, 128.7, 127.2, 127.1, 126.9, 126.8, 125.9, 125.8, 107.9, 107.3, 98.1, 97.2, 83.8, 75.3, 75.1, 24.87, 24.85, 14.9, 14.3; HRMS (ESI): calcd for $C_{23}H_{28}BO_3$ $(M+H)^+$ 363.2126, found 363.2131.

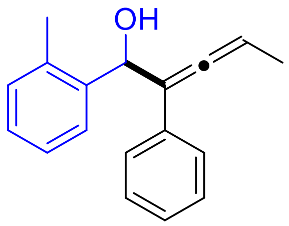

2-Phenyl-1-(*o*-tolyl)penta-2,3-dien-1-ol (**5w**): **Condition II** 51% yield, 64 mg; pale yellow oil; *dr* 1/1; ^{1}H NMR (400 MHz, CDCl$_3$) δ 7.50 (m, 1H), 7.30 (m, 4H), 7.20 (m, 4H), 6.32 (m, 1H), 5.46 (d, *J* = 8.0 Hz, 1H), 2.38 (d, *J* = 12.0 Hz, 3H), 2.16 (br, 1H), 1.72 (dd, *J* = 8.0 Hz, *J* = 4.0 Hz, 3H); ^{13}C NMR (101 MHz, CDCl$_3$) δ 201.7, 201.6, 139.5, 139.4, 135.9, 135.7, 134.7, 134.6, 130.6, 130.5, 128.62, 128.58, 127.7, 127.6, 127.1, 126.87, 126.85, 126.5, 126.3, 126.10, 126.05, 107.0, 106.9, 97.34, 97.27, 71.9, 71.8, 19.3, 19.1, 15.1, 14.8; HRMS (ESI): calcd for $C_{18}H_{19}O$ (M+H)$^+$ 251.1430, found 251.1430.

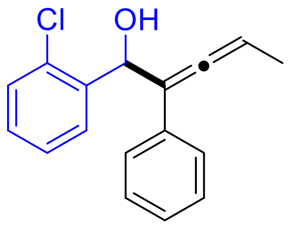

1-(2-Chlorophenyl)-2-phenylpenta-2,3-dien-1-ol (**5x**): **Condition II** 35% yield, 47 mg; pale yellow oil; *dr* 1/1; ^{1}H NMR (400 MHz, CDCl$_3$) δ 7.58 (t, *J* = 8.0 Hz, 1H), 7.32 (m, 6H), 7.20 (m, 2H), 6.31 (m, 1H), 5.68 (d, *J* = 12.0 Hz, 1H), 2.34 (t, *J* = 6.0 Hz, 1H), 1.78 (dd, *J* = 8.0 Hz, *J* = 4.0 Hz, 3H); ^{13}C NMR (101 MHz, CDCl$_3$) δ 201.8, 139.3, 139.1, 134.5, 134.3, 132.9, 132.8, 129.5, 128.91, 128.87, 128.7, 128.57, 128.55, 128.2, 128.1, 127.1, 127.00, 126.97, 126.9, 107.1, 106.7, 97.9, 97.6, 71.6, 15.3, 15.2; HRMS (ESI): calcd for $C_{17}H_{15}ClO$ (M+H)$^+$ 271.0884, found 271.0885.

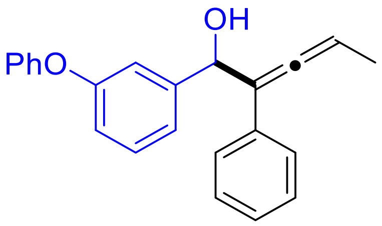

1-(3-Phenoxyphenyl)-2-phenylpenta-2,3-dien-1-ol (**5y**): **Condition II** 54% yield, 89 mg; pale yellow oil; *dr* 1/1; ^{1}H NMR (400 MHz, CDCl$_3$) δ 7.29 (m, 7H), 7.18 (m, 2H), 7.10 (m, 2H), 6.99 (d, *J* = 4.0 Hz, 2H), 6.94 (m, 1H), 6.32 (m, 1H), 5.18 (d, *J* = 40.0 Hz, 1H), 2.32 (br, 1H), 1.72 (d, *J* = 4.0 Hz, 3H); ^{13}C NMR (101 MHz, CDCl$_3$) δ 201.4, 200.9, 157.28, 157.26, 157.2, 144.0, 134.5, 129.72, 129.69, 128.7, 127.2, 127.1, 126.80, 126.77, 123.2, 121.5, 121.4, 118.8, 118.4, 118.3, 117.2, 117.1, 107.7, 107.4, 98.0, 97.4, 74.9, 74.8, 15.0, 14.5; HRMS (ESI): calcd for $C_{23}H_{21}O_2$ (M+H)$^+$ 329.1536, found 329.1533.

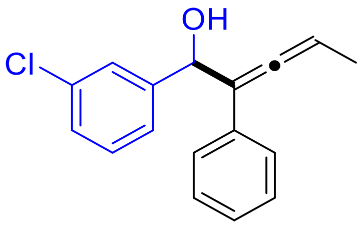

1-(3-Chlorophenyl)-2-phenylpenta-2,3-dien-1-ol (**5z**): **Condition II** 48% yield, 65 mg; pale yellow oil; *dr* 1/1; ^{1}H NMR (400 MHz, $CDCl_3$) δ 7.43 (m, 1H), 7.28 (m, 8H), 6.37 (m, 1H), 5.19 (d, *J* = 32.0 Hz, 1H), 2.35 (br, 1H), 1.70 (dd, *J* = 2.8 Hz, *J* = 1.6 Hz, 1H); ^{13}C NMR (101 MHz, $CDCl_3$) δ 201.3, 201.0, 143.9, 143.8, 134.4, 129.7, 128.7, 128.0, 127.30, 127.26, 126.85, 126.81, 126.76, 126.68, 124.69, 107.5, 107.2, 98.1, 97.6, 74.7, 74.6, 14.7, 14.4; HRMS (ESI): calcd for $C_{17}H_{16}ClO$ $(M+H)^+$ 271.0884, found 271.0879.

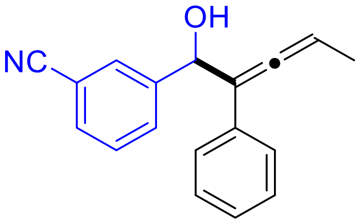

3-(1-Hydroxy-2-phenylpenta-2,3-dien-1-yl)benzonitrile (**5aa**): **Condition II** 37% yield, 48 mg; pale yellow oil; *dr* 1/1; ^{1}H NMR (400 MHz, $CDCl_3$) δ 7.74 (s, 1H), 7.66 (t, *J* = 8.0 Hz, 1H), 7.59 (m, 1H), 7.46 (t, *J* = 8.0 Hz, 1H), 7.31 (m, 4H), 7.23 (m, 1H), 6.37 (m, 1H), 5.28 (d, *J* = 24.0 Hz, 1H), 2.40 (br, 1H), 1.71 (t, *J* = 2.0 Hz, 3H); ^{13}C NMR (101 MHz, $CDCl_3$) δ 201.5, 201.4, 143.4, 143.3, 134.1, 131.4, 131.0, 130.9, 130.2, 129.2, 128.8, 127.5, 126.82, 126.79, 118.8, 112.5, 107.1, 106.9, 98.1, 97.8, 74.4, 74.3, 14.5, 14.3; HRMS (ESI): calcd for $C_{18}H_{14}N$ $(M-H_2O+H)^+$ 244.1121, found 244.1120.

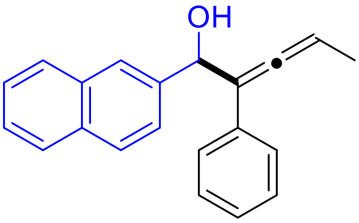

1-(Naphthalen-2-yl)-2-phenylpenta-2,3-dien-1-ol (**5ab**): **Condition II** 63% yield, 90 mg; pale yellow oil; *dr* 1/1; ^{1}H NMR (400 MHz, $CDCl_3$) δ 7.84 (m, 4H), 7.50 (m, 3H), 7.33 (m, 4H), 7.23 (m, 1H), 6.39 (m, 1H), 5.36 (dd, *J* = 44 Hz, *J* = 2.0 Hz, 1H), 2.45 (br, 1H), 1.71 (d, *J* = 4.0 Hz, 3H); ^{13}C NMR (101 MHz, $CDCl_3$) δ 201.4, 200.8, 139.1, 134.7, 134.6, 133.2, 133.1, 131.7, 131.6, 128.7, 128.33, 128.26, 128.0, 127.7, 127.2, 127.1, 126.9, 126.8, 126.2, 126.1, 126.0, 125.9, 125.6, 125.4, 124.4, 107.9, 107.5, 98.1, 97.4, 75.4, 75.3, 15.0, 14.5; HRMS (ESI): calcd for $C_{21}H_{19}O$ $(M+H)^+$ 287.1430, found 287.1428.

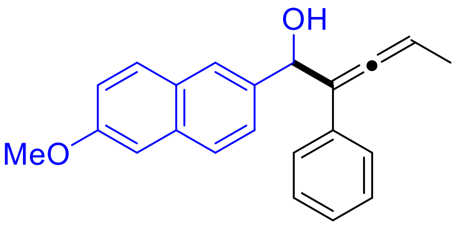

1-(6-Methoxynaphthalen-2-yl)-2-phenylpenta-2,3-dien-1-ol (**5ac**): **Condition II** 57% yield, 90 mg; pale yellow oil; *dr* 1/1; ^{1}H NMR (400 MHz, $CDCl_3$) δ 7.79 (s, 1H), 7.73 (m, 2H), 7.50 (m, 1H), 7.33 (m, 4H), 7.23 (m, 1H), 7.15 (m, 2H), 6.40 (m, 1H), 5.33 (d, *J* = 44 Hz, 1H), 3.91 (s, 3H), 2.40 (br, 1H), 1.72 (dd, *J* = 4.0 Hz, *J* = 1.2 Hz, 3H); ^{13}C NMR (101 MHz, $CDCl_3$) δ 201.3, 200.7, 157.8, 136.9, 134.8, 134.7, 134.31, 134.28, 131.6, 129.5, 128.7, 127.20, 127.16, 127.1, 126.9, 126.8, 125.6, 125.4, 125.01, 124.99, 119.0, 118.9, 108.1, 107.6, 105.7, 98.1, 97.4, 75.4, 75.3, 55.3, 15.1, 14.6; HRMS (ESI): calcd for $C_{22}H_{21}O_2$ $(M+H)^+$ 317.1536 , found 317.1531.

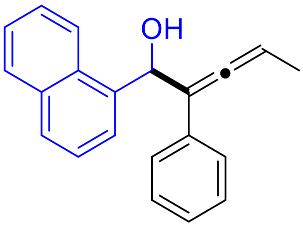

1-(Naphthalen-1-yl)-2-phenylpenta-2,3-dien-1-ol (**5ad**): **Condition II** 61% yield, 87 mg; pale yellow oil; *dr* 1/1; ^{1}H NMR (400 MHz, $CDCl_3$) δ 8.17 (m, 1H), 7.85 (m, 1H), 7.79 (m, 1H), 7.70 (dd, *J* = 12.0 Hz, *J* = 8.0 Hz, 1H), 7.41 (m, 3H), 7.27 (m, 4H), 7.20 (m, 1H), 6.35 (m, 1H), 5.95 (m, 1H), 2.42 (br, 1H), 1.69 (t, *J* = 4.0 Hz, 3H); ^{13}C NMR (101 MHz, $CDCl_3$) δ 202.4, 201.9, 136.7, 136.5, 134.5, 134.4, 133.9, 133.8, 130.9, 130.7, 128.72, 128.69, 128.6, 128.5, 128.4, 127.1, 127.0, 126.1, 126.0, 125.58, 125.56, 125.19, 125.15, 124.7, 124.2, 124.1, 123.8, 107.3, 106.7, 97.5, 96.8, 73.1, 72.7, 15.1, 14.5; HRMS (ESI): calcd for $C_{21}H_{19}O$ $(M+H)^+$ 287.1430, found 287.1430.

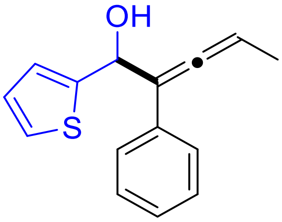

2-Phenyl-1-(thiophen-2-yl)penta-2,3-dien-1-ol (**5ae**): **Condition II** 28% yield, 34 mg; pale yellow oil; *dr* 1/1; ^{1}H NMR (400 MHz, $CDCl_3$) δ 7.31(m, 6H), 7.22 (m, 1H), 7.11 (m, 1H), 6.34 (m, 1H), 5.32 (dd, *J* = 32.0 Hz, *J* = 4.0 Hz, 1H), 2.23 (br, 1H), 1.76 (d, *J* = 4.0 Hz, 3H); ^{13}C NMR (101 MHz, $CDCl_3$) δ 201.4, 200.9, 143.3, 134.62, 134.58, 128.7, 127.2, 127.1, 126.83, 126.79, 126.2, 126.09, 126.07, 126.0, 122.0, 121.8, 107.5, 107.1, 97.8, 97.2, 71.8, 71.4, 14.9, 14.5; HRMS (ESI): calcd for $C_{15}H_{15}OS$ $(M+H)^+$ 243.0838, found 243.0835.

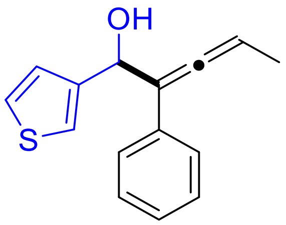

2-Phenyl-1-(thiophen-3-yl)penta-2,3-dien-1-ol (**5af**): **Condition II** 36% yield, 44 mg; pale yellow oil; *dr* 1/1; ^{1}H NMR (400 MHz, $CDCl_3$) δ 7.32 (m, 5H), 7.22 (m, 1H), 7.08 (t, *J* = 4.0 Hz, 1H), 6.99 (t, *J* = 6.0 Hz, 1H), 6.40 (m, 1H), 5.45 (d, *J* = 40.0 Hz, 1H), 2.40 (br, 1H), 1.81 (dd, *J* = 4.0 Hz, *J* = 8.0 Hz, 3H); ^{13}C NMR (101 MHz, $CDCl_3$) δ 201.2, 200.3, 146.1, 134.4, 134.3, 128.7, 127.3, 127.2, 127.0, 126.9, 126.63, 126.60, 125.43, 125.38, 125.2, 124.9, 107.8, 107.4, 98.8, 97.9, 71.4, 70.9, 15.3, 14.7; HRMS (ESI): calcd for $C_{15}H_{15}OS$ $(M+H)^+$ 243.0838, found 243.0836.

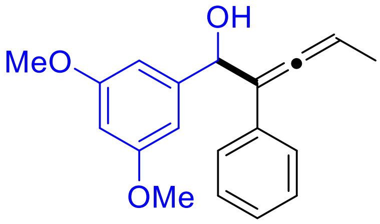

1-(3,5-Dimethoxyphenyl)-2-phenylpenta-2,3-dien-1-ol (**5ag**): **Condition II** 63% yield, 93 mg; pale yellow oil; *dr* 1/1; ^{1}H NMR (400 MHz, $CDCl_3$) δ 7.31 (m, 4H), 7.20 (m, 1H), 6.59 (m, 2H), 6.35 (m, 2H), 5.14 (d, *J* = 60.0 Hz, 1H), 3.76 (s, 6H), 2.37 (br, 1H), 1.73 (dd, *J* = 4.0 Hz, *J* = 1.2 Hz, 3H); ^{13}C NMR (101 MHz, $CDCl_3$) δ 201.7, 200.7, 160.81, 160.78, 144.3, 134.73, 134.66, 128.6, 127.14, 127.07, 126.84, 126.78, 107.9, 107.2, 104.5, 104.3, 100.1, 100.0, 98.0, 96.9, 75.3, 75.1, 55.3, 15.0, 14.3; HRMS (ESI): calcd for $C_{19}H_{21}O_3$ $(M+H)^+$ 297.1485, found 297.1486.

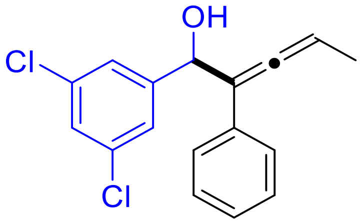

1-(3,5-Dichlorophenyl)-2-phenylpenta-2,3-dien-1-ol (**5ah**): **Condition II** 41% yield, 63 mg; pale yellow oil; *dr* 1/1; ^{1}H NMR (400 MHz, $CDCl_3$) δ 7.28 (m, 8H), 6.38 (m, 1H), 5.18 (dd, *J* = 28.0 Hz, *J* = 1.2 Hz, 1H), 2.34 (br, 1H), 1.71 (d, *J* = 8.0 Hz, 3H); ^{13}C NMR (101 MHz, $CDCl_3$) δ 201.5, 201.3, 145.3, 145.2, 135.0, 134.1, 128.78, 128.76, 127.9, 127.44, 127.42, 126.9, 126.8, 125.1, 107.0, 106.7, 98.1, 97.8, 74.3, 74.2, 14.5, 14.2; HRMS (ESI): calcd for $C_{17}H_{13}Cl_2$ $(M-H_2O+H)^+$ 287.0389, found 287.0388.

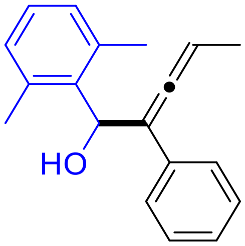

1-(2,6-Dimethylphenyl)-2-phenylpenta-2,3-dien-1-ol (**5ai**): **Condition II** 54% yield, 71 mg; pale yellow oil; *dr* 1/1; ^{1}H NMR (400 MHz, $CDCl_3$) δ 7.31 (m, 4H), 7.22 (m, 1H), 7.08 (m, 2H), 7.00 (d, *J* = 4.0 Hz, 2H), 6.40 (m, 1H), 5.78 (s, 1H), 2.45 (s, 6H), 2.21 (br, 1H), 1.67 (d, *J* = 4.0 Hz, 3H); ^{13}C NMR (101 MHz, $CDCl_3$) δ 199.9, 137.4, 136.2, 134.7, 129.3, 128.6, 127.6, 127.2, 126.9, 107.5, 99.1, 70.1, 20.6, 15.9; HRMS (ESI): calcd for $C_{19}H_{21}O$ (M +H)$^{+}$ 265.1587, found 265.1585.

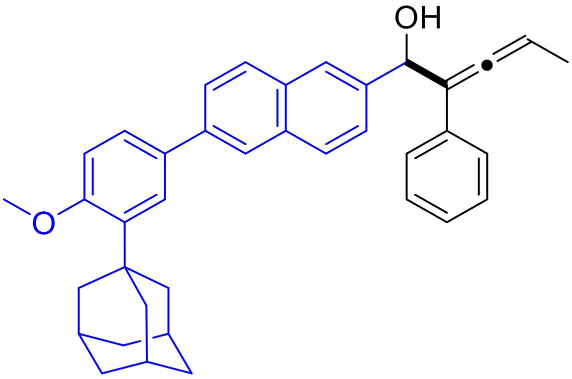

1-(6-(3-(Adamantan-1-yl)-4-methoxyphenyl)naphthalen-2-yl)-2-phenylpenta-2,3-dien-1-ol (**5aj**): **Condition II** 52% yield, 137 mg; pale yellow oil; *dr* 1/1; ^{1}H NMR (400 MHz, $CDCl_3$) δ 7.98 (s, 1H), 7.88 (m, 3H), 7.74 (d, *J* = 8.0 Hz, 1H), 7.59 (s, 1H), 7.54 (t, J = 8.0 Hz, 2H), 7.34 (m, 4H), 7.23 (m, 1H), 6.98 (d, *J* = 8.0 Hz, 1H), 6.42 (m, 1H), 5.38 (d, *J* = 44.0 Hz, 1H), 3.89 (s, 3H), 2.43 (br, 1H), 2.19 (s, 6H), 2.10 (s, 3H), 1.80 (s, 6H), 1.73 (d, *J* = 2.4 Hz, 3H); ^{13}C NMR (101 MHz, $CDCl_3$) δ 201.4, 200.8, 158.6, 139.18, 139.15, 138.9, 138.8, 134.71, 134.65, 133.5, 133.1, 132.0, 128.7, 128.5, 128.4, 128.3, 127.20, 127.15, 126.92, 126.86, 126.0, 125.9, 125.6, 125.4, 125.2, 124.8, 124.7, 112.1, 108.0, 107.5, 98.1, 97.5, 75.4, 75.3, 55.1, 40.6, 37.2 37.1, 29.1, 15.1, 14.6; HRMS (ESI): calcd for $C_{38}H_{37}O$ (M-H_2O+H)$^{+}$ 509.2839, found 509.2836.

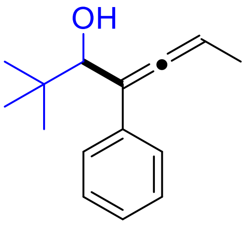

2,2-Dimethyl-4-phenylhepta-4,5-dien-3-ol (**5ak**): **Condition II** 21% yield, 23 mg; pale yellow oil; *dr* 1/1; ^{1}H NMR (400 MHz, $CDCl_3$) δ 7.29 (m, 4H), 7.19 (m, 1H), 6.21 (m, 1H), 3.81 (t, *J* = 4.0 Hz, 1H), 1.91 (d, *J* = 4.0 Hz, 3H), 0.99 (d, *J* = 4.0 Hz, 9H); ^{13}C NMR (101 MHz, $CDCl_3$) δ 203.1, 202.8, 134.9, 134.8, 128.63, 128.56, 126.9, 126.7, 106.2, 105.9, 96.3, 96.1, 81.2, 80.8, 36.5, 26.2, 18.0, 17.8; HRMS (ESI): calcd for $C_{15}H_{21}O$ (M+H)$^{+}$ 217.1587, found 217.1587.

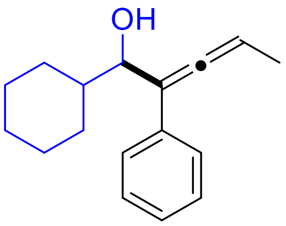

1-Cyclohexyl-2-phenylpenta-2,3-dien-1-ol (**5al**): **Condition II** 29% yield, 35 mg; pale yellow oil; *dr* 1/1; ^{1}H NMR (400 MHz, $CDCl_3$) δ 7.31 (m, 4H), 7.19 (m, 1H), 6.21 (m, 1H), 3.91 (t, *J* = 8.0 Hz, 1H), 1.93 (br, 1H), 1.81 (dd, *J* = 4.0 Hz, *J* = 2.0 Hz, 3H), 1.65 (m, 7H), 1.12 (m, 3H), 0.87 (m, 1H); ^{13}C NMR (101 MHz, $CDCl_3$) δ 202.2, 201.7, 135.0, 128.6, 126.87, 126.85, 126.8, 126.7, 106.2, 105.7, 96.3, 95.7, 77.9, 41.2, 40.9, 29.8, 29.7, 27.9, 27.5, 26.4, 25.9, 14.6, 14.1; HRMS (ESI): calcd for $C_{17}H_{23}O$ $(M+H)^+$ 243.1743, found 243.1740.

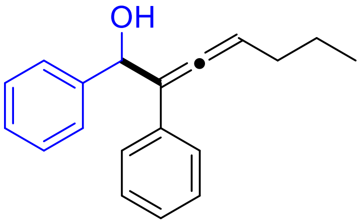

1,2-Diphenylhepta-2,3-dien-1-ol (**5am**): **Condition II** 72% yield, 95 mg; pale yellow oil; *dr* 1/1; ^{1}H NMR (400 MHz, $CDCl_3$) δ ^{1}H NMR (400 MHz, $CDCl_3$) δ 7.35 (m, 9H), 7.22 (m, 1H), 6.45 (m, 1H), 5.20 (dd, *J* = 40.0 Hz, *J* = 2.0 Hz, 1H), 2.32 (br, 1H), 1.94 (m, 2H), 1.45 (m, 2H), 0.86 (dt, *J* = 8.0 Hz, *J* = 4.0 Hz, 3H); ^{13}C NMR (101 MHz, $CDCl_3$) δ 200.6, 200.2, 142.12, 142.06, 134.8, 134.7, 128.7, 128.41, 128.38, 127.9, 127.8, 127.13, 127.10, 126.71, 126.65, 113.6, 113.3, 99.9, 99.4, 74.8, 74.7, 31.0, 30.7, 20.9, 20.8, 13.93, 13.90; HRMS (ESI): calcd for $C_{19}H_{21}O$ $(M+H)^+$ 265.1587, found 265.1589.

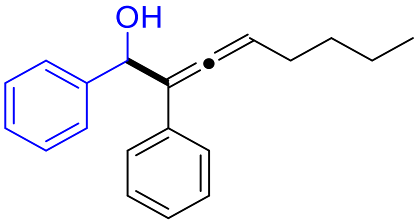

1,2-Diphenylocta-2,3-dien-1-ol (**5an**): **Condition II** 72% yield, 100 mg; **Condition II** (starting from allene substrate) 85% yield, 118 mg; pale yellow oil; *dr* 1/1; ^{1}H NMR (400 MHz, $CDCl_3$) δ 7.41 (t, *J* = 6.0 Hz, 2H), 7.31 (m, 7H), 7.21 (m, 1H), 6.45 (m, 1H), 5.19 (d, *J* = 40.0 Hz, 1H), 2.35 (br, 1H), 1.96 (m, 2H), 1.40 (m, 2H), 1.27 (m, 2H), 0.81 (t, *J* = 6.0 Hz, 3H); ^{13}C NMR (101 MHz, $CDCl_3$) δ 200.6, 200.2, 142.12, 142.06, 134.8, 134.7, 128.6, 128.39, 128.35, 127.84, 127.80, 127.10, 127.07, 126.7, 126.64, 126.62, 113.8, 113.4, 99.9, 99.3, 74.8, 74.7, 29.8, 29.7, 28.5, 28.2, 22.43, 22.40, 13.8; HRMS (ESI): calcd for $C_{20}H_{23}O$ $(M+H)^+$ 279.1743, found 279.1743.

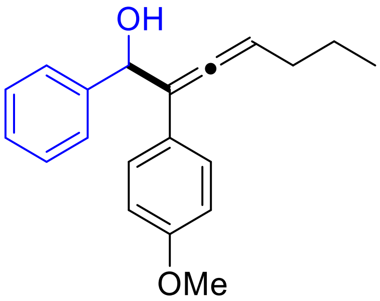

2-(4-Methoxyphenyl)-1-phenylhepta-2,3-dien-1-ol (**5ao**): **Condition II** 63% yield, 73 mg; pale yellow oil; *dr* 1/1; ^{1}H NMR (400 MHz, $CDCl_3$) δ 7.42 (m, 2H), 7.37, 7.35 (m, 2H), 7.27 (m, 3H), 6.86 (d, *J* = 8.0 Hz, 2H), 6.44 (m, 1H), 5.17 (d, *J* = 40.0 Hz, 1H), 3.80 (s, 3H), 2.36 (br, 1H), 1.92 (m, 2H), 1.43 (m, 2H), 0.86 (dt, *J* = 8.0 Hz, *J* = 4.0 Hz, 3H); ^{13}C NMR (101 MHz, $CDCl_3$) δ 199.7, 199.2, 158.9, 142.3, 142.2, 128.39, 128.36, 127.82, 127.77, 127.04, 126.96, 126.73, 126.67, 114.19, 114.17, 113.6, 113.2, 99.6, 99.0, 74.8, 74.7, 55.3, 31.1, 30.8, 20.93, 20.86, 13.93, 13.89; HRMS (ESI): calcd for $C_{20}H_{23}O_2$ $(M+H)^+$ 295.1693, found 295.1696.

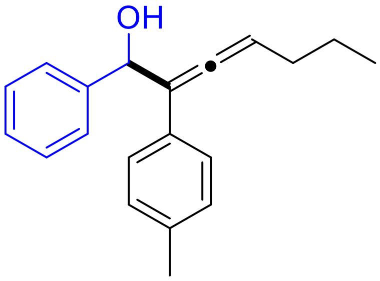

1-Phenyl-2-(*p*-tolyl)hepta-2,3-dien-1-ol (**5ap**): **Condition II** 59% yield, 82 mg; pale yellow oil; *dr* 1/1; ^{1}H NMR (400 MHz, $CDCl_3$) δ 7.35 (m, 5H), 7.22 (d, *J* = 8.0 Hz, 2H), 7.12 (d, *J* = 8.0 Hz, 2H), 6.44 (m, 1H), 5.18 (d, *J* = 44.0 Hz, 1H), 2.37 (br, 1H), 2.33 (s, 3H), 1.93 (m, 2H), 1.43 (m, 2H), 0.85 (dt, *J* = 8.0 Hz, *J* = 4.0 Hz, 3H); ^{13}C NMR (101 MHz, $CDCl_3$) δ 200.2, 199.7, 142.2, 142.1, 136.96, 136.94, 131.8, 131.7, 129.4, 128.38, 128.35, 127.81, 127.78, 126.73, 126.66, 126.62, 126.56, 113.5, 113.2, 99.9, 99.3, 74.8, 74.7, 31.0, 30.7, 21.2, 20.9, 20.8, 13.92, 13.89; HRMS (ESI): calcd for $C_{20}H_{23}O$ $(M+H)^+$ 279.1743, found 279.1745.

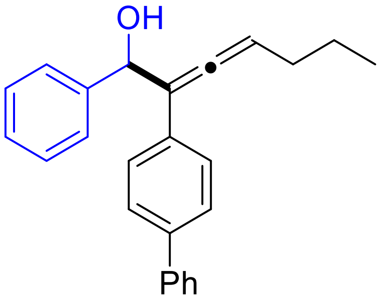

2-([1,1'-Biphenyl]-4-yl)-1-phenylhepta-2,3-dien-1-ol (**5aq**): **Condition II** 59% yield, 100 mg; white solid, m.p. 137-139 ˚C; *dr* 1/1; ^{1}H NMR (400 MHz, $CDCl_3$) δ 7.58 (m, 4H), 7.38 (m, 10H), 6.49 (q, *J* = 4.0 Hz, 1H), 5.28 (s, 1H), 2.27 (br, 1H), 1.98 (m, 2H), 1.48 (m, 2H), 0.89 (t, *J* = 6.0 Hz, 3H); ^{13}C NMR (101 MHz, $CDCl_3$) δ 200.9, 142.1, 140.8, 140.0, 133.8, 128.8, 128.4, 127.9, 127.4, 127.2, 127.1 126.9, 126.7, 113.4, 99.0, 74.8, 30.7, 20.9, 14.0; HRMS (ESI): calcd for $C_{25}H_{25}O$ $(M+H)^+$ 341.1900,

found 341.1909.

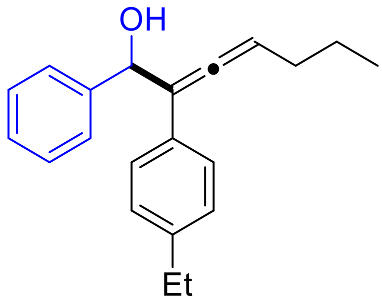

2-(4-Ethylphenyl)-1-phenylhepta-2,3-dien-1-ol (**5ar**): **Condition II** 71% yield, 104 mg; pale yellow oil; *dr* 1/1; ^{1}H NMR (400 MHz, $CDCl_3$) δ 7.42 (m, 2H), 7.36 (m, 2H), 7.30 (m, 1H), 7.25 (d, *J* = 8.0 Hz, 2H), 7.16 (d, *J* = 4.0 Hz, 2H), 6.46 (m, 1H), 5.11 (d, *J* = 44 Hz, 1H), 2.64 (q, *J* = 8.0 Hz, 2H), 2.31 (br, 1H), 1.93 (m, 2H), 1.44 (m, 2H), 1.24 (dt, *J* = 8.0 Hz, *J* = 4.0 Hz, 3H), 0.86 (dt, *J* = 8.0 Hz, *J* = 4.0 Hz, 3H); ^{13}C NMR (101 MHz, $CDCl_3$) δ 200.2, 199.6, 143.5, 143.4, 142.2, 142.1, 132.0, 131.9, 128.40, 128.37, 128.2, 127.84, 127.80, 126.77, 126.71, 126.68, 126.66, 113.6, 113.2, 99.9, 99.4, 74.8, 74.7, 31.0, 30.8, 28.6, 20.93, 20.86, 15.6, 13.94, 13.90; HRMS (ESI): calcd for $C_{21}H_{25}O$ $(M+H)^+$ 293.1900, found 293.1902.

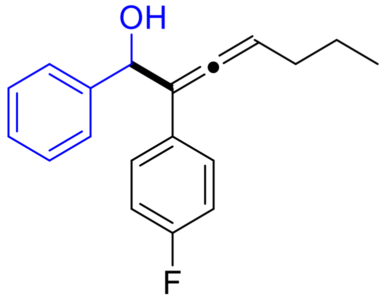

2-(4-Fluorophenyl)-1-phenylhepta-2,3-dien-1-ol (**5as**): **Condition II** 65% yield, 92 mg; pale yellow oil; *dr* 1/1; ^{1}H NMR (400 MHz, $CDCl_3$) δ 7.38 (m, 4H), 7.28 (m, 3H), 7.00 (d, *J* = 8.0 Hz, 2H), 6.42 (m, 1H), 5.20 (dd, *J* = 32.0 Hz, *J* = 4.0 Hz, 1H), 2.26 (br, 1H), 1.95 (m, 2H), 1.44 (m, 2H), 0.86 (dt, *J* = 8.0 Hz, *J* = 4.0 Hz, 3H); ^{13}C NMR (101 MHz, $CDCl_3$) δ 200.5, 200.1, 162.0 (d, $J_{F\text{-}C}$ = 245.0 Hz), 142.1, 130.73 (d, $J_{F\text{-}C}$ = 8.0 Hz), 130.70 (d, $J_{F\text{-}C}$ = 8.0 Hz), 128.5, 128.4, 128.10 (d, $J_{F\text{-}C}$ = 8.0 Hz), 128.06 (d, $J_{F\text{-}C}$ = 8.0 Hz), 127.9, 126.6, 115.7, 115.5, 113.9, 113.5, 98.9, 98.4, 74.8, 74.7, 31.0, 30.8, 20.91, 20.85, 13.92, 13.89; HRMS (ESI): calcd for $C_{19}H_{20}FO$ $(M+H)^+$ 283.1493, found 283.1491.

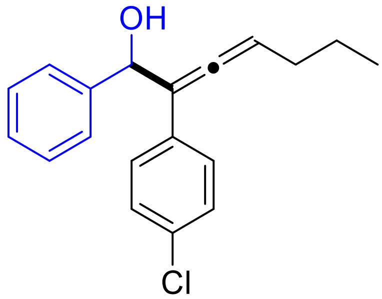

2-(4-Chlorophenyl)-1-phenylhepta-2,3-dien-1-ol (**5at**): **Condition II** 57% yield, 85 mg; pale yellow

oil; *dr* 1/1; ^{1}H NMR (400 MHz, $CDCl_3$) δ 7.34 (m, 7H), 7.23 (m, 2H), 6.39 (m, 1H), 5.21 (dd, *J* = 32.0 Hz, *J* = 4.0 Hz, 1H), 2.21 (br, 1H), 1.95 (m, 2H), 1.44 (m, 2H), 0.86 (dt, *J* = 8.0 Hz, *J* = 2.0 Hz, 3H); ^{13}C NMR (101 MHz, $CDCl_3$) δ 201.0, 200.6, 142.01, 141.98, 133.4, 133.3, 132.6, 128.8, 128.5, 128.4, 128.0, 127.83, 127.80, 126.6, 114.1, 113.7, 98.8, 98.3, 74.8, 74.7, 31.0, 30.7, 20.9, 20.8, 13.91, 13.88; HRMS (ESI): calcd for $C_{19}H_{20}ClO$ $(M+H)^+$ 299.1197, found 299.1187.

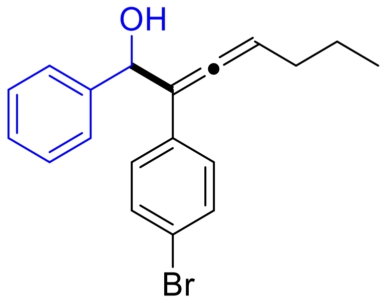

2-(4-Bromophenyl)-1-phenylhepta-2,3-dien-1-ol (**5au**): **Condition II** 64% yield, 110 mg; pale yellow oil; *dr* 1/1; ^{1}H NMR (400 MHz, $CDCl_3$) δ 7.42 (d, *J* = 8.0 Hz, 2H), 7.31 (m, 5H), 7.16 (dd, *J* = 8.0 Hz, *J* = 5.6 Hz, 2H), 6.37 (m, 1H), 5.20 (dd, *J* = 32.0 Hz, *J* = 4.0 Hz, 1H), 2.24 (br, 1H), 1.95 (m, 2H), 1.43 (m, 2H), 0.86 (dt, *J* = 8.0 Hz, *J* = 4.0 Hz, 3H); ^{13}C NMR (101 MHz, $CDCl_3$) δ 201.2, 200.8, 142.12, 142.09, 134.0, 133.9, 131.9, 128.58, 128.56, 128.29, 128.26, 128.09, 128.07, 126.7, 120.8, 114.2, 113.8, 98.9, 98.4, 74.9, 74.8, 31.1, 30.7, 21.01, 20.95, 14.04, 14.01; HRMS (ESI): calcd for $C_{19}H_{18}Br$ $(M-H_2O+H)^+$ 325.0586, found 325.0587.

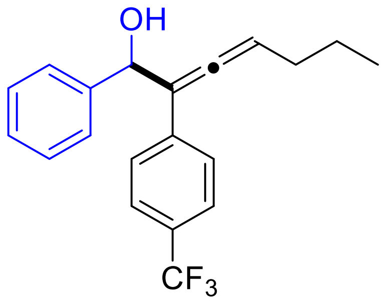

1-Phenyl-2-(4-(trifluoromethyl)phenyl)hepta-2,3-dien-1-ol (**5av**): **Condition II** 43% yield, 71 mg; pale yellow oil; *dr* 1/1; ^{1}H NMR (400 MHz, $CDCl_3$) δ 7.55 (d, *J* = 8.0 Hz, 2H), 7.34 (m, 7H), 6.45 (m, 1H), 5.24 (d, *J* = 36.0 Hz, 1H), 2.26 (br, 1H), 1.98 (m, 2H), 1.46 (m, 2H), 0.87 (t, *J* = 8.0 Hz, 3H); ^{13}C NMR (101 MHz, $CDCl_3$) δ 202.2, 201.8, 141.9, 138.8, 128.9 (q, J_{F-C} = 33.0 Hz), 128.51, 128.49, 128.1, 126.74, 126.71, 126.6, 125.58 (q, J_{F-C} = 4.0 Hz), 124.2 (q, J_{F-C} = 260.0 Hz), 114.2, 113.8, 98.6, 98.1, 74.8, 74.7, 30.9, 30.6, 20.9, 20.8, 13.91, 13.88; HRMS (ESI): calcd for $C_{20}H_{20}F_3O$ $(M+H)^+$ 333.1461, found 333.1457.

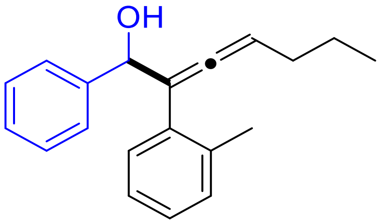

1-Phenyl-2-(*o*-tolyl)hepta-2,3-dien-1-ol (**5aw**): **Condition II** 60% yield, 84 mg; pale yellow oil; *dr* 1/1; ^{1}H NMR (400 MHz, $CDCl_3$) δ 7.40 (d, *J* = 8.0 Hz, 3H), 7.34 (t, *J* = 8.0 Hz, 2H), 7.28 (t, *J* = 8.0 Hz, 1H), 7.13 (m, 3H), 6.64 (m, 1H), 5.19 (d, *J* = 24.0 Hz, 1H), 2.37 (s, 3H), 2.34 (br, 1H), 1.93 (m, 2H), 1.45 (m, 2H), 0.86 (t, *J* = 8.0 Hz, 3H); ^{13}C NMR (101 MHz, $CDCl_3$) δ 201.3, 201.0, 142.10, 142.07, 134.94, 134.92, 132.89, 132.8, 130.5, 128.4, 127.8, 126.99, 126.96, 126.9, 126.71, 126.67, 126.13, 126.09, 112.4, 112.2, 96.9, 96.5, 74.73, 74.65, 30.9, 30.7, 20.92, 20.88, 19.8, 13.94, 13.91; HRMS (ESI): calcd for $C_{20}H_{23}O$ $(M+H)^+$ 279.1743, found 279.1749.

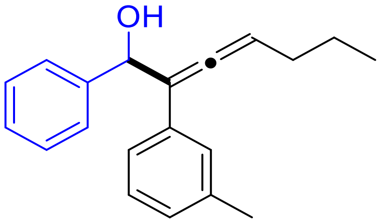

1-Phenyl-2-(*m*-tolyl)hepta-2,3-dien-1-ol (**5ax**): **Condition II** 74% yield, 103 mg; pale yellow oil; *dr* 1/1; ^{1}H NMR (400 MHz, $CDCl_3$) δ 7.42 (t, *J* = 8.0 Hz, 2H), 7.35 (t, *J* = 8.0 Hz, 2H), 7.28 (m, 1H), 7.20 (t, *J* = 8.0 Hz, 1H), 7.13 (s, 2H), 7.03 (d, *J* = 4.0 Hz, 1H), 6.43 (m, 1H), 5.19 (d, *J* = 44.0 Hz, 1H), 2.33 (s, 4H), 1.93 (m, 2H), 1.44 (m, 2H), 0.86 (dt, *J* = 8.0 Hz, *J* = 4.0 Hz, 3H); ^{13}C NMR (101 MHz, $CDCl_3$) δ 200.5, 200.1, 142.2, 142.1, 138.24, 138.20, 134.7, 134.6, 128.6, 128.5, 128.4, 128.3, 127.9, 127.82, 127.79, 127.4, 127.3, 126.7, 126.6, 123.9, 123.8, 113.5, 113.1, 100.0, 99.4, 74.8, 74.7, 31.0, 30.6, 21.3, 20.9, 20.8, 13.91, 13.88; HRMS (ESI): calcd for $C_{20}H_{23}O$ $(M+H)^+$ 279.1743, found 279.1745.

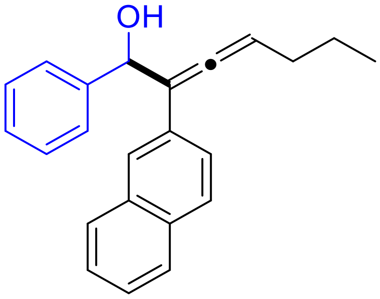

2-(Naphthalen-2-yl)-1-phenylhepta-2,3-dien-1-ol (**5ay**): **Condition II** 66% yield, 104 mg; pale yellow oil; *dr* 1/1; ^{1}H NMR (400 MHz, $CDCl_3$) δ 7.78 (t, *J* = 6.0 Hz, 3H), 7.67 (s, 1H), 7.39 (m, 8H), 6.62 (m, 1H), 5.24 (d, *J* = 44.0 Hz, 1H), 2.37 (br, 1H), 1.99 (m, 2H), 1.47 (m, 2H), 0.88 (dt, *J* = 8.0 Hz, *J* = 4.0 Hz, 3H); ^{13}C NMR (101 MHz, $CDCl_3$) δ 201.3, 200.9, 142.2, 142.1, 133.7, 132.7, 132.3, 132.2, 128.42, 128.40, 128.31, 128.27, 127.89, 127.87, 127.7, 126.68, 126.65, 126.3, 125.7, 125.64, 125.56, 124.51, 124.48, 113.9, 113.5, 100.2, 99.6, 74.9, 74.7, 31.1, 30.7, 20.93, 20.87, 13.94, 13.91; HRMS (ESI): calcd for $C_{23}H_{23}O$ $(M+H)^+$ 315.1743, found 315.1744.

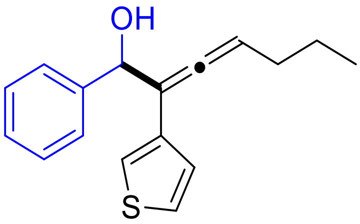

1-Phenyl-2-(thiophen-3-yl)hepta-2,3-dien-1-ol (**5az**): **Condition II** 46% yield, 62 mg; pale yellow oil; *dr* 1/1; ^{1}H NMR (400 MHz, $CDCl_3$) δ 7.35 (m, 6H), 7.08 (m, 2H), 6.53 (m, 1H), 5.17 (d, *J* = 44.0 Hz, 1H), 2.33 (br, 1H), 1.92 (m, 2H), 1.43 (m, 2H), 0.86 (dt, *J* = 8.0 Hz, *J* = 4.0 Hz, 3H); ^{13}C NMR (101 MHz, $CDCl_3$) δ 200.8, 200.1, 142.2, 142.1, 136.21, 136.15, 128.41, 128.37, 127.9, 127.8, 126.7, 126.6, 126.1, 126.0, 121.0, 120.9, 112.8, 112.3, 94.5, 93.8, 74.7, 74.6, 31.1, 30.7, 30.6, 20.9, 20.8, 13.89, 13.86; HRMS (ESI): calcd for $C_{17}H_{19}OS$ $(M+H)^+$ 271.1151, found 271.1148.

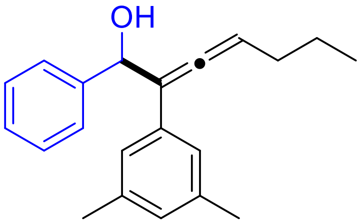

2-(3,5-Dimethylphenyl)-1-phenylhepta-2,3-dien-1-ol (**5ba**): **Condition II** 57% yield, 83 mg; pale yellow oil; *dr* 1/1; ^{1}H NMR (400 MHz, $CDCl_3$) δ 7.43 (t, *J* = 6.0 Hz, 2H), 7.35 (t, *J* = 8.0 Hz, 2H), 7.29 (m, 1H), 6.94 (s, 2H), 6.86 (s, 1H), 6.41 (m, 1H), 5.19 (dd, *J* = 48.0 Hz, *J* = 4.0 Hz, 1H), 2.29 (s, 6H), 1.94 (m, 2H), 1.45 (m, 2H), 0.86 (t, *J* = 8.0 Hz, 3H); ^{13}C NMR (101 MHz, $CDCl_3$) δ 200.4, 200.0, 142.3, 142.2, 138.2, 138.1, 134.6, 134.5, 128.9, 128.3, 127.82, 127.78, 126.73, 126.66, 124.6, 124.5, 113.5, 113.0, 100.1, 99.5, 74.73, 74.66, 31.0, 30.6, 21.2, 20.9, 20.8, 13.91, 13.88; HRMS (ESI): calcd for $C_{21}H_{25}O$ $(M+H)^+$ 293.1900, found 293.1901.

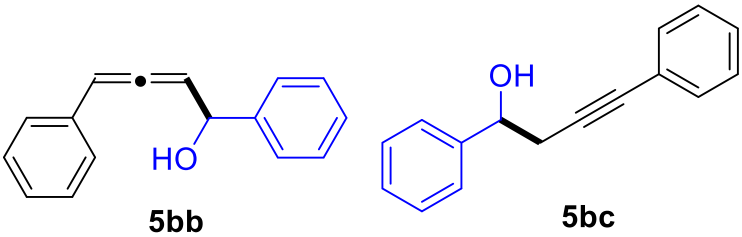

5bb **5bc**

1,4-Diphenylbuta-2,3-dien-1-ol (**5bb**), 1,4-diphenylbut-3-yn-1-ol (**5bc**): mixture of 5bc and 5bd was obtained in 38% yield, 42 mg; pale yellow oil; ^{1}H NMR (400 MHz, $CDCl_3$) δ 7.44 (t, *J* = 6.0 Hz, 5H), 7.38 (t, *J* = 6.0 Hz, 8H), 7.29 (m, 11H), 7.21 (m, 1H), 6.38 (dd, *J* = 8.0 Hz, *J* = 4.0 Hz, 1H) (**5bb**), 5.87 (m, 1H) (**5bb**), 5.37 (dd, *J* = 8.0 Hz, *J* = 4.0 Hz, 1H) (**5bb**), 4.95 (t, *J* = 6.0 Hz, 1.3H) (**5bc**), 2.86 (d, *J* = 8.0 Hz, 2.7H) (**5bc**), 2.47 (br, 1.2H), 2.25 (br, 1H); ^{13}C NMR (101 MHz, $CDCl_3$) δ 203.8 (**5bb**), 203.5 (**5bb**), 142.7, 133.6, 131.6, 128.7, 128.6, 128.4, 128.2, 128.0, 127.9, 127.4, 127.3, 126.90, 126.87, 126.1, 126.0, 125.8, 123.2, 100.1 (**5bb**), 100.0 (**5bb**), 98.2 (**5bb**), 97.9 (**5bb**), 85.9 (**5bc**), 83.2 (**5bc**), 72.6, 72.4, 72.2, 30.6 (**5bc**); These data are in accordance with the literature.[18,19]

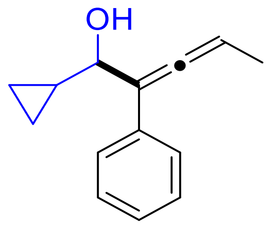

1-Cyclopropyl-2-phenylpenta-2,3-dien-1-ol (**5bd**): **Condition II** 53% yield, 53 mg; pale yellow oil; *dr* 1/1; ^{1}H NMR (400 MHz, $CDCl_3$) δ 7.29 (m, 4H), 7.19 (m, H), 6.24 (m, 1H), 3.47 (t, *J* = 8.0 Hz, 1H), 1.92 (d, *J* = 2.8 Hz, 3H), 1.84 (br, 1H), 1.11 (m, 1H), 0.58 (m, 2H), 0.35 (m, 2H); ^{13}C NMR (101 MHz, $CDCl_3$) δ 201.5, 201.3, 135.0, 134.9, 128.58, 128.56, 126.9, 126.8, 126.69, 126.65, 107.5, 107.0, 96.6, 95.9, 77.6, 16.7, 16.5, 15.4, 14.6, 3.2, 3.00, 2.96, 2.7; HRMS (ESI): calcd for $C_{14}H_{17}O$ $(M+H)^+$ 201.1274, found 201.1276.

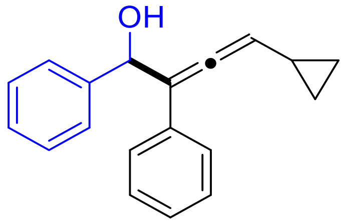

4-Cyclopropyl-1,2-diphenylbuta-2,3-dien-1-ol (**5be**): **Condition II** 10% yield, mg; pale yellow oil; *dr* 1/1; ^{1}H NMR (400 MHz, $CDCl_3$) δ 7.49 (m, 2H), 7.37 (m, 2H), 7.30 (m, 5H), 7.22 (m, 1H), 6.46 (m, 1H), 5.36 (d, *J* = 32.0 Hz, 1H), 2.33 (br, 1H), 1.11 (m, 1H), 0.65 (m, 2H), 0.41 (m, 2H); ^{13}C NMR (101 MHz, $CDCl_3$) δ 199.4, 199.0, 142.3, 142.2, 134.5, 134.4, 128.7, 128.38, 128.35, 127.8, 127.3, 126.80, 126.75, 126.7, 126.6, 117.3, 117.0, 100.8, 100.4, 75.2, 75.1, 9.5, 9.3, 7.7, 7.6, 7.0; HRMS (ESI): calcd for $C_{19}H_{19}O$ $(M+H)^+$ 263.1430, found 263.1429.

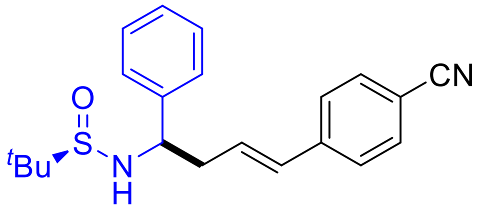

(*R*)-*N*-((*E*)-4-(4-Cyanophenyl)-1-phenylbut-3-en-1-yl)-2-methylpropane-2-sulfinamide (**7**): **Condition II** 84% yield, 148 mg; pale yellow oil; *dr* 5/1; ^{1}H NMR (400 MHz, $CDCl_3$) δ 7.54 (d, *J* = 8.0 Hz, 2H), 7.33 (m, 7H), 6.38 (d, *J* = 16.0 Hz, 1H), 6.15 (m, 1H), 4.56 (t, *J* = 8.0 Hz, 1H), 3.55 (s, 1H), 2.93 (m, 1H), 2.76 (m, 1H), 1.23 (s, 9H); ^{13}C NMR (101 MHz, $CDCl_3$) δ 141.6, 141.5, 132.3, 131.7, 129.8, 128.8, 128.1, 127.0, 126.4, 118.9, 110.4, 58.3, 55.9, 40.2, 22.6; HRMS (ESI): calcd for $C_{21}H_{24}N_2OS$ $(M+H)^+$ 353.1682, found 353.1682.

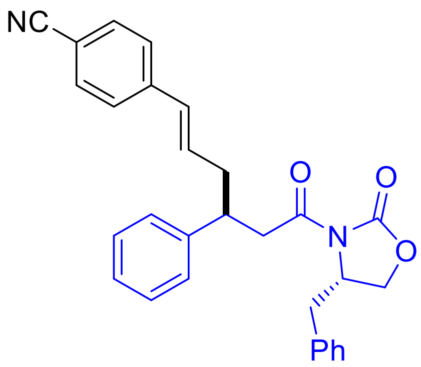

4-((*S,E*)-6-((*S*)-4-Benzyl-2-oxooxazolidin-3-yl)-6-oxo-4-phenylhex-1-en-1-yl)benzonitrile (**9**): **Condition II** 36% yield, 81 mg; white solid, m.p. 118-119 °C; ^{1}H NMR (400 MHz, $CDCl_3$) δ 7.51 (d, *J* = 8.0 Hz, 2H), 7.29 (m, 6H), 7.21 (m, 4H), 6.98 (d, *J* = 8.0 Hz, 2H), 6.37 (d, *J* = 16.0 Hz, 1H), 6.21 (m, 1H), 4.53 (t, *J* = 8.0 Hz, 1H), 4.03 (m, 2H), 3.47 (m, 2H), 3.23 (dd, *J* = 8.0 Hz, *J* = 20.0 Hz, 1H), 2.97 (d, *J* = 16.0 Hz, 1H), 2.61 (d, *J* = 8.0 Hz, 2H), 2.52 (dd, *J* = 8.0 Hz, *J* = 12.0 Hz, 1H); ^{13}C NMR (101 MHz, $CDCl_3$) δ 171.8, 153.3, 143.2, 141.8, 134.9, 132.4, 132.3, 130.5, 129.3, 128.9, 128.6, 127.6, 127.3, 126.8, 126.5, 119.0, 110.3, 65.9, 54.9, 41.6, 40.8, 40.2, 37.4; HRMS (ESI): calcd for $C_{29}H_{26}N_2O_3$ $(M+H)^+$ 451.2016, found 451.2017.

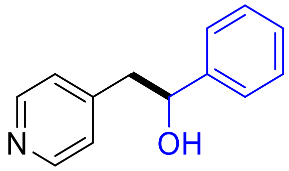

1-Phenyl-2-(pyridin-4-yl)ethan-1-ol (**10**): **Condition II** 31% yield, 31 mg; white solid, m.p. 107-108 °C; ^{1}H NMR (400 MHz, $CDCl_3$) δ 8.39 (d, *J* = 4.0 Hz, 2H), 7.31 (m, 5H), 7.08 (d, *J* = 4.0 Hz, 2H), 4.92 (t, *J* = 8.0 Hz, 1H), 3.01 (m, 3H); ^{13}C NMR (101 MHz, $CDCl_3$) δ 149.4, 147.5, 143.5, 128.5, 127.9, 125.8, 124.9, 74.4, 45.1, 29.7 ; These data are in accordance with the literature.[20]

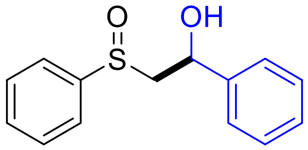

1-Phenyl-2-(phenylsulfinyl)ethan-1-ol (**11**): **Condition II** 31% yield, 48 mg; colorless oil; *dr* 1/1 ^{1}H NMR (400 MHz, $CDCl_3$) δ 7.65 (d, *J* = 8.0 Hz, 2H), 7.54 (m, 3H), 7.31 (m, 5H), 5.31 (dd, *J* = 8.0 Hz, *J* = 28.0 Hz, 1H), 4.41 (d, *J* = 36.0 Hz, 1H), 3.23 (dd, *J* = 12.0 Hz, *J* = 20.0 Hz, 1H), 2.92 (dd, *J* = 16.0 Hz, *J* = 36.0 Hz, 1H); ^{13}C NMR (101 MHz, $CDCl_3$) δ 143.5, 142.8, 142.0, 141.9, 131.5, 131.1, 129.5, 129.4, 128.7, 128.6, 128.1, 127.9, 125.7, 125.6, 123.9, 123.8, 71.1, 68.6, 64.3, 64.0; These data are in accordance with the literature.[21]

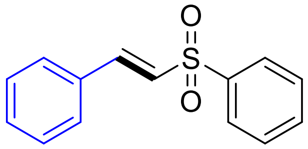

(*E*)-(2-(Phenylsulfonyl)vinyl)benzene (**12**): **Condition II** 41% yield, 50 mg; colorless oil; ^{1}H NMR

(400 MHz, $CDCl_3$) δ 7.96 (d, *J* = 12.0 Hz, 2H), 7.69 (d, *J* = 16.0 Hz, 1H), 7.62 (t, *J* = 8.0 Hz, 1H), 7.55 (t, *J* = 8.0 Hz, 2H), 7.49 (d, *J* = 4.0 Hz, 2H), 7.39 (m, 3H), 6.87 (d, *J* = 16.0 Hz, 1H); ^{13}C NMR (101 MHz, $CDCl_3$) δ 142.5, 140.7, 133.4, 132.3, 131.2, 129.3, 129.1, 128.6, 127.6, 127.3; These data are in accordance with the literature.[22]

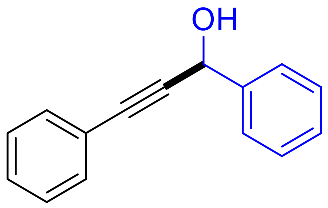

1,3-Diphenylprop-2-yn-1-ol (**13**): **Condition II** 45% yield, 47 mg; colorless oil; ^{1}H NMR (400 MHz, $CDCl_3$) δ 7.60 (d, *J* = 8.0 Hz, 2H), 7.46 (m, 2H), 7.39 (t, *J* = 8.0 Hz, 2H), 7.32 (m, 4H), 5.68 (s, 1H); ^{13}C NMR (101 MHz, $CDCl_3$) δ 140.6, 131.7, 128.7, 128.6, 128.4, 128.3, 126.7, 122.4, 88.7, 86.7, 65.1; These data are in accordance with the literature.[23]

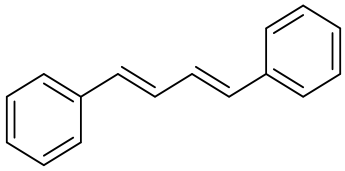

(1*E*,3*E*)-1,4-Diphenylbuta-1,3-diene (**14a**): 92% yield, 95 mg; white solid, m.p. 148-150˚C; ^{1}H NMR (400 MHz, $CDCl_3$) δ 7.43 (d, *J* = 8.0 Hz, 4H), 7.32 (t, *J* = 8.0 Hz, 4H), 7.22 (t, *J* = 8.0 Hz, 2H), 6.95 (m, 2H), 6.66 (m, 2H); ^{13}C NMR (101 MHz, $CDCl_3$) δ 137.3, 132.8, 129.2, 128.6, 127.5, 126.4; These data are in accordance with the literature.[24]

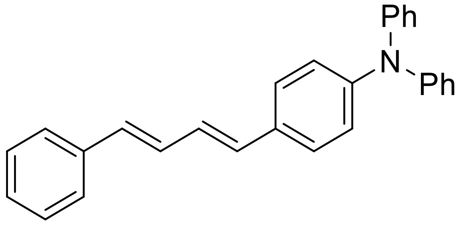

N,N-Diphenyl-4-((1*E*,3*E*)-4-phenylbuta-1,3-dien-1-yl)aniline (**14b**): 93% yield, 174 mg; pale yellow solid, m.p. 154-156˚C; ^{1}H NMR (400 MHz, $CDCl_3$) δ 7.42 (d, *J* = 8.0 Hz, 2H), 7.32 (t, *J* = 10.0 Hz, 4H), 7.26 (d, *J* = 8.0 Hz, 3H), 7.19 (t, *J* = 10.0 Hz, 2H), 7.10 (d, *J* = 8.0 Hz, 4H), 7.03 (t, *J* = 6.0 Hz, 4H), 6.88 (m, 2H), 6.62 (dd, *J* = 16.0 Hz, *J* = 4.0 Hz,2H); ^{13}C NMR (101 MHz, $CDCl_3$) δ 147.5, 147.3, 137.5, 132.4, 131.9, 131.5, 129.5, 129.3, 128.6, 127.7, 127.3, 127.2, 126.3, 124.5, 123.5, 123.1; HRMS (ESI): calcd for $C_{28}H_{24}N$ $(M+H)^+$ 374.1903, found 374.1895.

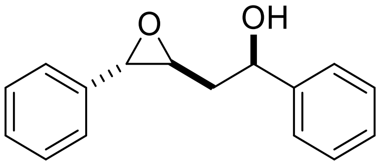

1-Phenyl-2-(*trans*-3-phenyloxiran-2-yl)ethan-1-ol (Isomer I, **15a**): 55% yield, 133 mg; white solid, m.p. 108-110 ˚C; ^{1}H NMR (400 MHz, $CDCl_3$) δ 7.38 (t, *J* = 8.0 Hz, 4H), 7.32 (m, 4H), 7.25 (t, *J* = 6.0 Hz, 2H), 5.02 (m, 1H), 3.69 (s, 1H), 3.24 (t, *J* = 6.0 Hz, 1H), 2.33 (d, *J* = 4.0 Hz, 1H), 2.23 (m, 1H), 2.02 (m, 1H); ^{13}C NMR (101 MHz, $CDCl_3$) δ 144.0, 137.2, 128.6, 128.5, 128.4, 128.2, 127.7, 125.61, 125.56, 71.7, 60.3, 58.6, 41.2; These data are in accordance with the literature.[25]

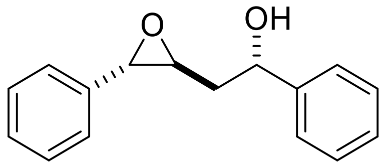

1-Phenyl-2-(*trans*-3-phenyloxiran-2-yl)ethan-1-ol (Isomer II, **15b**): 35% yield, 83 mg; colorless oil; ^{1}H NMR (400 MHz, $CDCl_3$) δ 7.37 (t, *J* = 6.0 Hz, 4H), 7.29 (m, 4H), 7.16 (d, *J* = 12.0 Hz, 2H), 4.96 (t, *J* = 6.0 Hz, 1H), 3.55 (s, 1H), 3.02 (t, *J* = 6.0 Hz, 1H), 2.50 (s, 1H), 2.13 (m, 2H); ^{13}C NMR (101 MHz, $CDCl_3$) δ 143.7, 140.7, 137.1, 128.6, 128.4, 128.1, 127.9, 125.8, 125.5, 72.6, 60.6, 58.3, 41.6; These data are in accordance with the literature.[25]

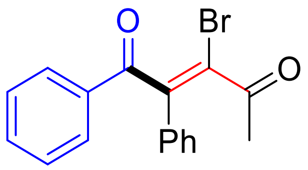

(*Z*)-3-Bromo-1,2-diphenylpent-2-ene-1,4-dione (**16**): 51% yield, 84 mg; pale yellow solid, m.p. 68-70 ˚C; ^{1}H NMR (400 MHz, $CDCl_3$) δ 7.75 (d, *J* = 8.0 Hz, 2H), 7.70 (d, *J* = 8.0 Hz, 2H), 7.52 (t, *J* = 8.0 Hz, 2H), 7.38 (t, *J* = 6.0 Hz, 4H), 2.33 (s, 3H); ^{13}C NMR (101 MHz, $CDCl_3$) δ 195.3, 190.6, 144.6, 135.5, 134.9, 133.80, 133.76, 130.0, 129.5, 128.7, 128.5, 124.2, 21.6; HRMS (ESI): calcd for $C_{17}H_{14}BrO_2$ $(M+H)^+$ 329.0172, found 329.0172.

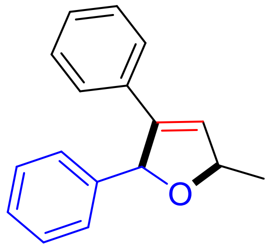

5-Methyl-2,3-diphenyl-2,5-dihydrofuran (**17**): 84% yield, 99 mg; colorless oil; *dr* 1/1; ^{1}H NMR (400 MHz, $CDCl_3$) δ 7.34 (m, 10H), 5.93 (d, *J* = 64.0 Hz, 1H), 5.74 (d, *J* = 4.0 Hz, 1H), 5.65 (d, *J* = 32.0 Hz, 1H), 1.62 (d, *J* = 8.0 Hz, 3H); ^{13}C NMR (101 MHz, $CDCl_3$) δ 142.4, 141.9, 141.1, 140.5, 139.6, 139.2, 128.53, 128.47, 128.42, 128.40, 128.0, 127.72, 127.70, 127.66, 126.9, 126.3, 124.8, 124.7, 91.0, 90.6, 87.9, 86.9, 12.7, 12.5; HRMS (ESI): calcd for $C_{17}H_{17}O$ $(M+H)^+$ 237.1274, found 237.1270.

12. References

1. Zhang, S.; Bedi, D.; Cheng, L.; Unruh, D. K.; Li, G.; Findlater, M. *J. Am. Chem. Soc.* **2020**, *142*, 8910–8917.
2. Thiel, N. O.; Kaewmee, B.; Ngoc, T. T.; Teichert, J. F. *Chem. Eur. J.* **2020**, *26*, 1597–1603.
3. Zhang, Z.; Liu, X.; Ji, L.; Zhang, T.; Jia, Z.; Loh, T.-P. *ACS Catal.* **2022**, *12*, 2052−2057.
4. Lu, B.; Li, C.; Zhang, L. *J. Am. Chem. Soc.* **2010**, *132*, 14070-14072.
5. Phadke, N.; Findlater, M. *Molecules* **2015**, *20*, 20195–20205.
6. Xie, H.; Breit, B. *ACS Catal.* **2022**, *12*, 3249−3255.
7. Ye, K.-Y.; McCallum, T.; Lin, S. *J. Am. Chem. Soc.* **2019**, *141*, 9548−9554.
8. Li, G.; Han, A.; Pulling, M. E.; Estes, D. P.; Norton, J. R. *J. Am. Chem. Soc.* **2012**, *134*, 14662–14665.
9. Zhao, L.-M.; Gao, H.-S.; Li, D.-F.; Dong, J.; Sang, L.-L.; Ji, J. *Org. Biomol. Chem.* **2017**, *15*, 4359–4366.
10. Kunishima, M.; Hioki, K.; Nakata, D.; Nogawa, S.; Tani, S. *Chem. Lett.* **1999**, 683-684.
11. Spivey, A. C.; Laraia, L; Bayly, A. R.; Rzepa, H. S.; White, A. J. P. *Org. Lett*. **2010**, *12*, 900-903.
12. Takeda, M.; Shintani, R.; Hayashi, T. *J. Org. Chem.* **2013**, *78*, 5007−5017.
13. Huang, R.-Z.; Lau, K. K.; Li, Z.; Liu, T.-L.; Zhao, Y. *J. Am. Chem. Soc.* **2018**, *140*, 14647−14654.
14. Li, L.; Navasero, N. *Org. Lett*. **2004**, *6*, 3091-3094.
15. Chaudhari, T. Y.; Hossian, A.; Manna, M. K.; Jana, R. *Org. Biomol. Chem.* **2015**, *13*, 4841–4845.
16. Babu, M. H.; Kumar, G. R.; Kant, R.; Reddy, M. S. *Chem. Commun.* **2017**, *53*, 3894–3897.
17. Mokar, B. D.; Liu, R.-S. *Chem. Commun.* **2014**, *50*, 8966–8969.
18. Redon, S.; Berkaoui, A.-L. B.; Pannecouke, X.; Outurquin, F. *Tetrahedron* **2007**, *63*, 3707–3717.
19. Wang, T.; Jiang, Y.; Wang, Y.; Yan, R. *Org. Biomol. Chem.* **2018**, *16*, 5232–5235.
20. Barrios-Rivera, J.; Xu, Y.; Clarkson, G. J.; Wills, M. *Tetrahedron* **2022**, *103*, 132562.
21. Zhao, J.-J.; Tang, M.; Zhang, H.-H.; Xu, M.-M.; Shi, F. *Chem. Commun.* **2016**, 52, 5953-5956.
22. Tathe, A. G.; Patil, N. T. *Org. Lett.* **2022**, *24*, 4459–4463.
23. Baek, J.; Si, T.; Kim, H. Y.; Oh, K. *Org. Lett.* **2022,** *24*, 4982–4986.
24. Huang, F.; Huang, Z.; Liu, G.; Huang, Z. *Org. Lett.* **2022**, *24*, 5486−5490.
25. Bhadra, S.; Akakura, M.; Yamamoto, H. *J. Am. Chem. Soc.* **2015**, *137*, 15612−15615.
