# Supplementary material for: Electrochemically Generated Carbanions Enable Isomerizing Allylation and Allenylation of Aldehydes with Alkenes and Alkynes
Source: J Am Chem Soc. 2023 Jun 15;145(25):14143–54. doi: 10.1021/jacs.3c04864 (PMC10311535; doi:10.1021/jacs.3c04864)

Part II NMR spectra

3a ^{1}H NMR

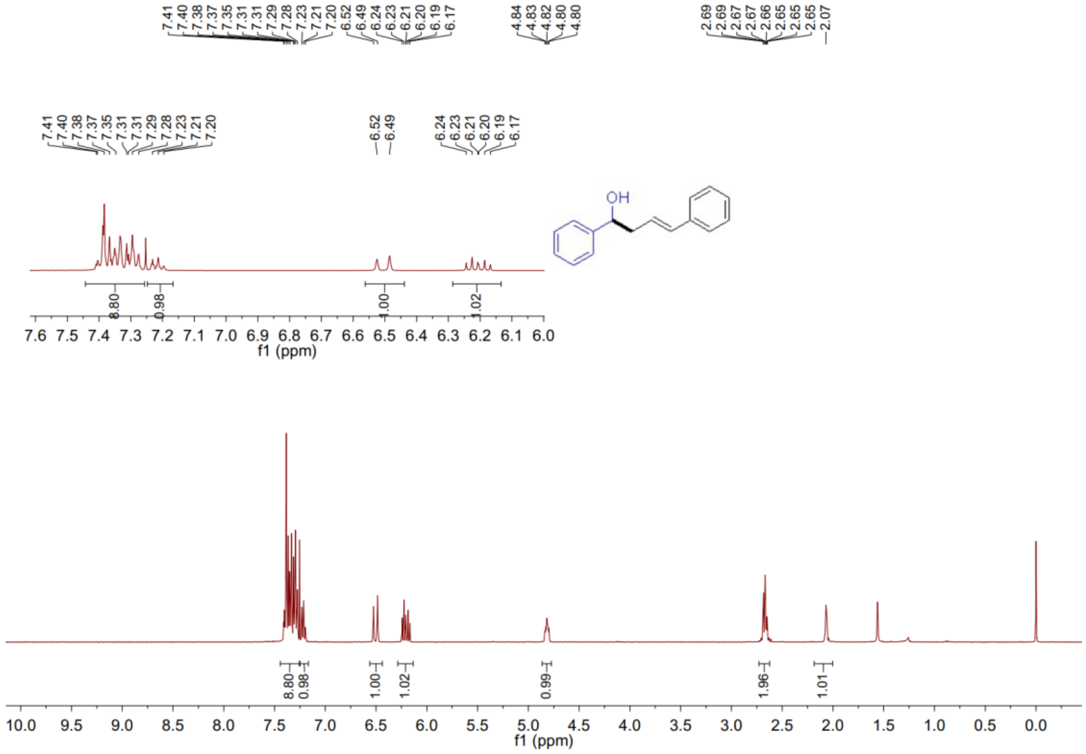

3a ^{13}C NMR

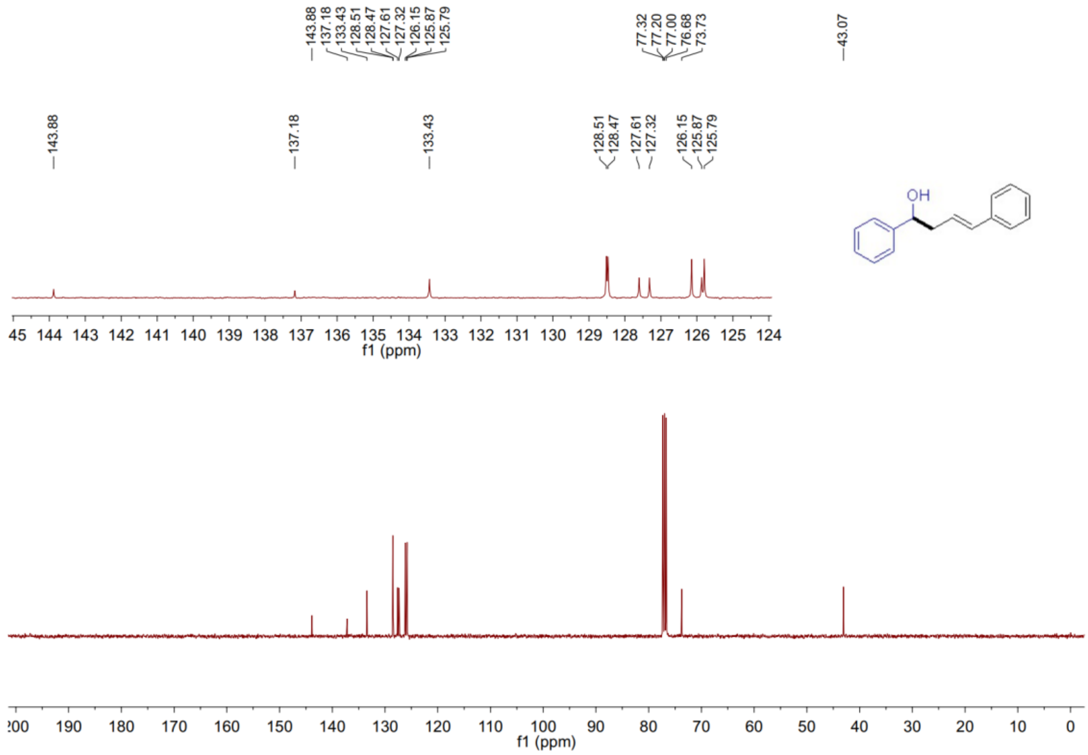

3b ^{1}H NMR

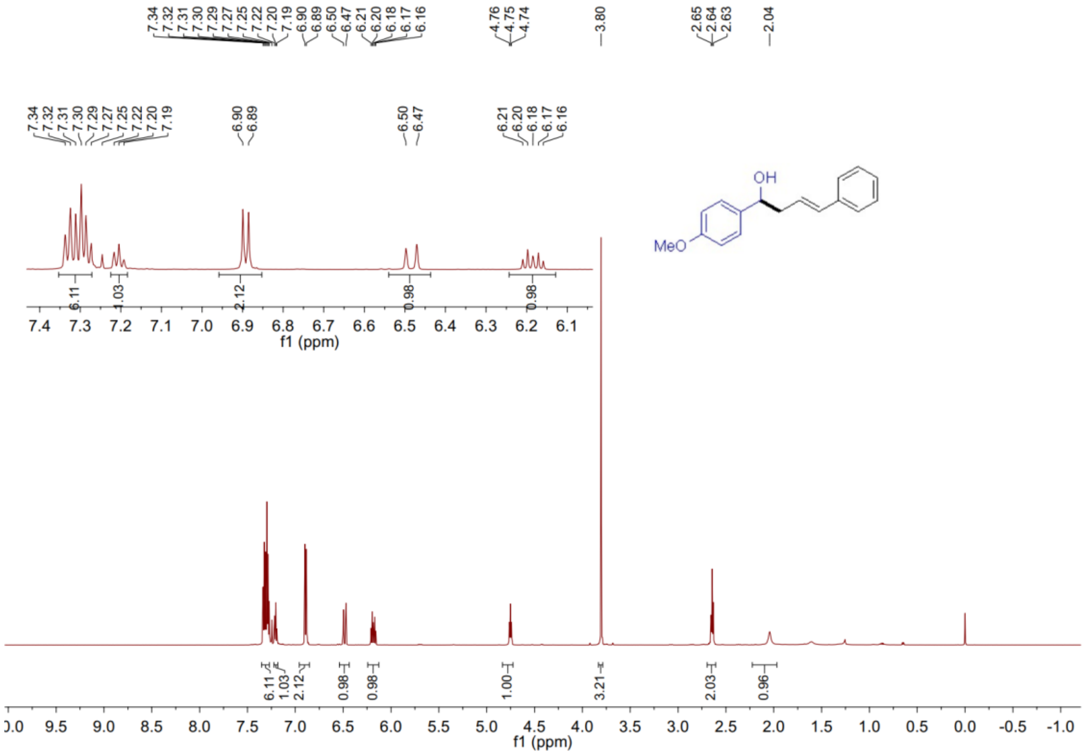

3b ^{13}C NMR

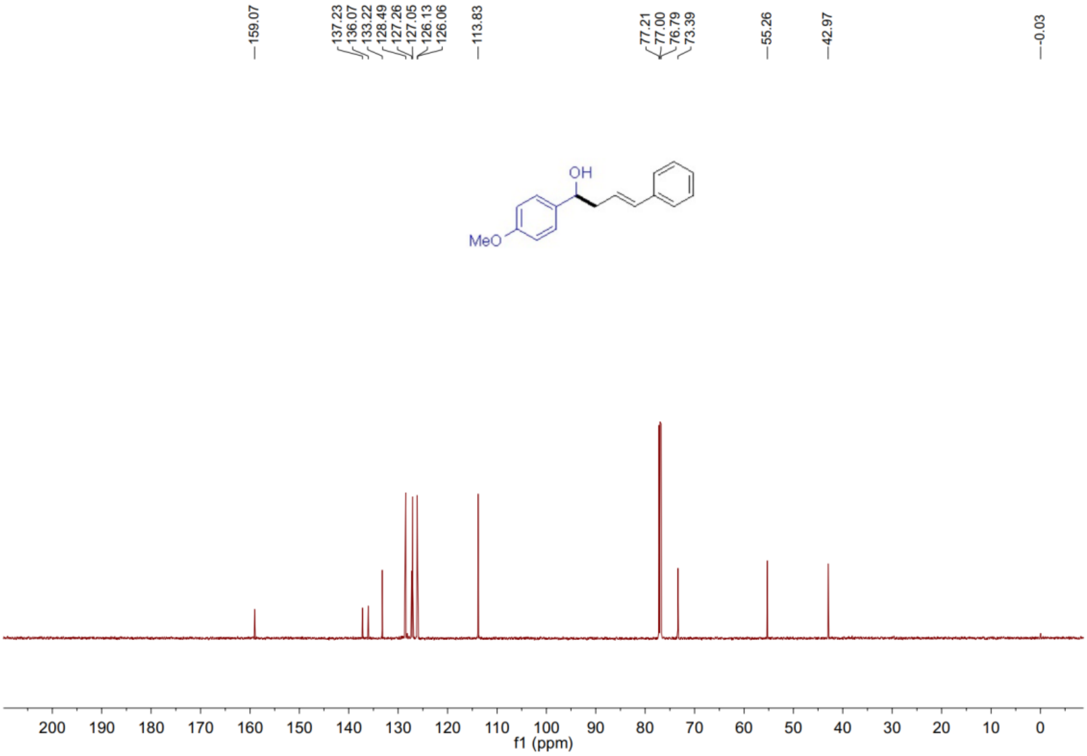

3c ^{1}H NMR

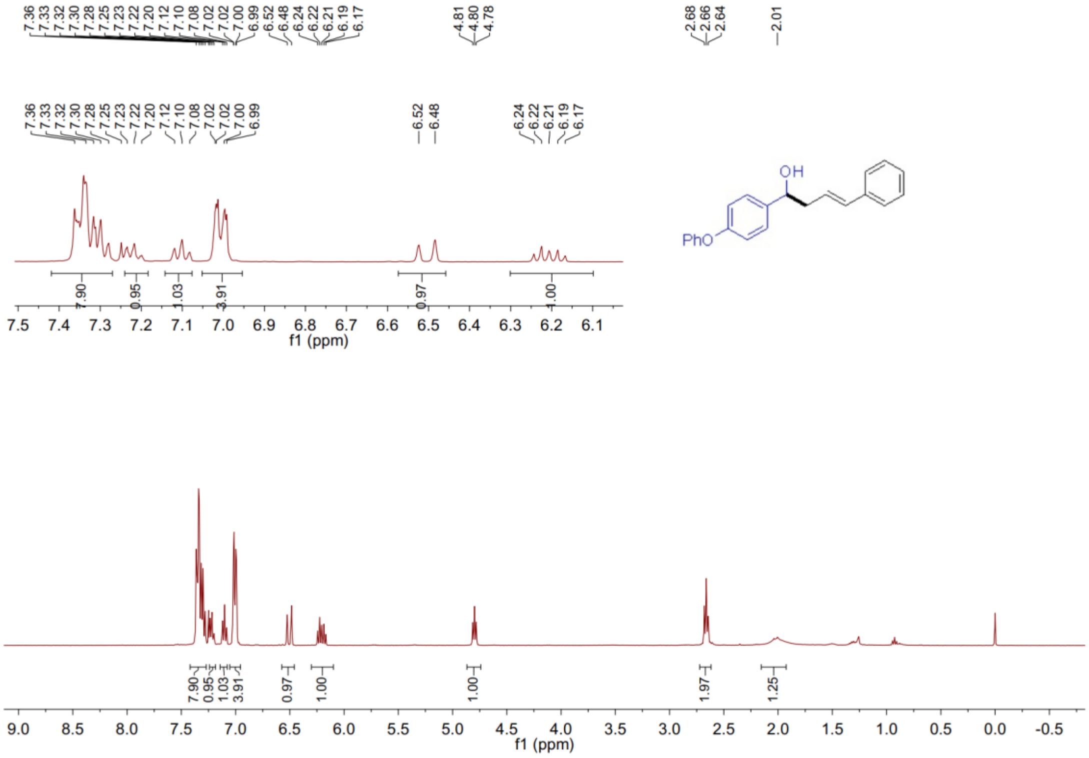

3c ^{13}C NMR

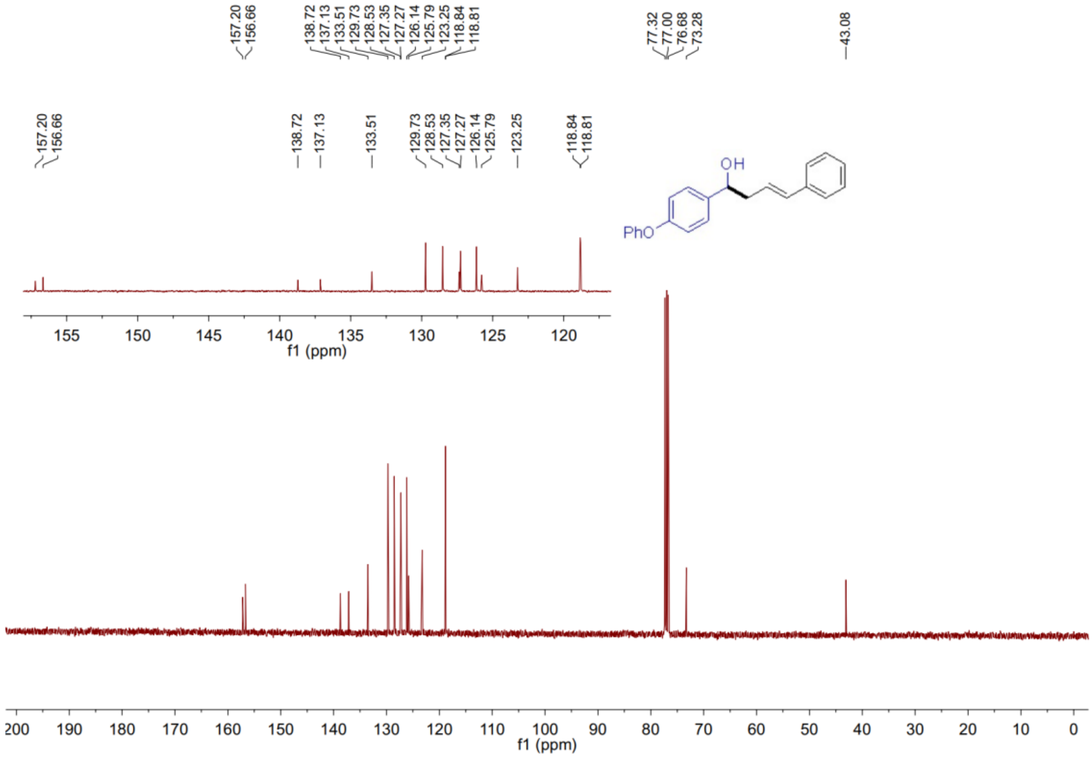

3d ^{1}H NMR

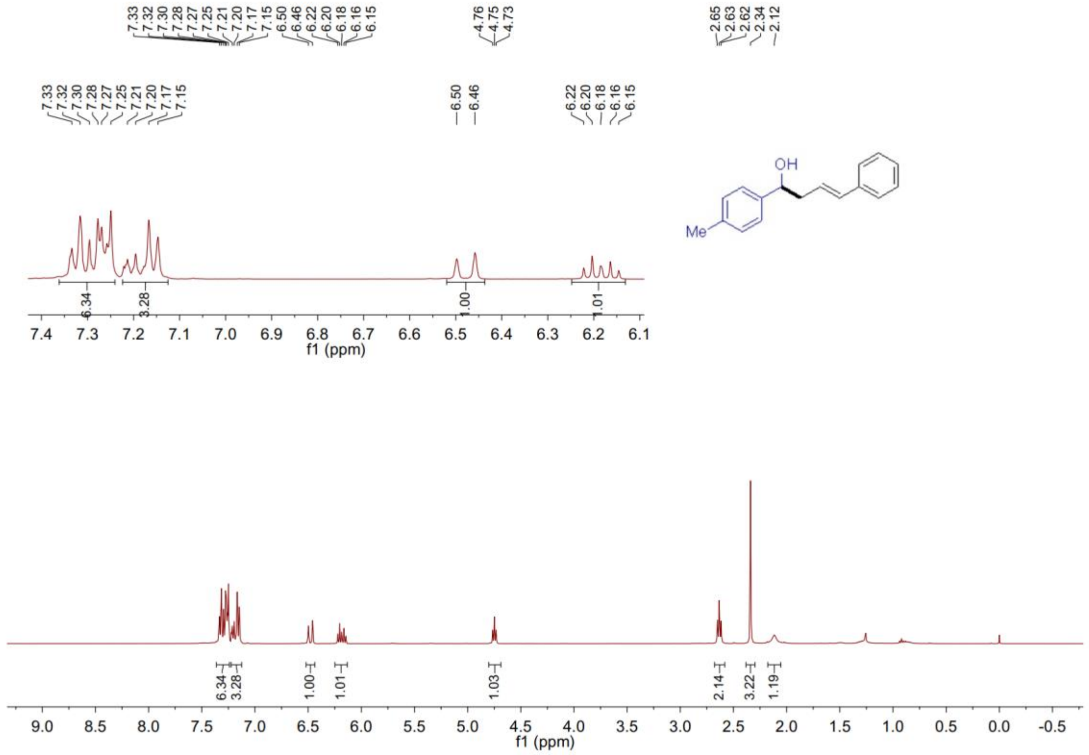

3d ^{13}C NMR

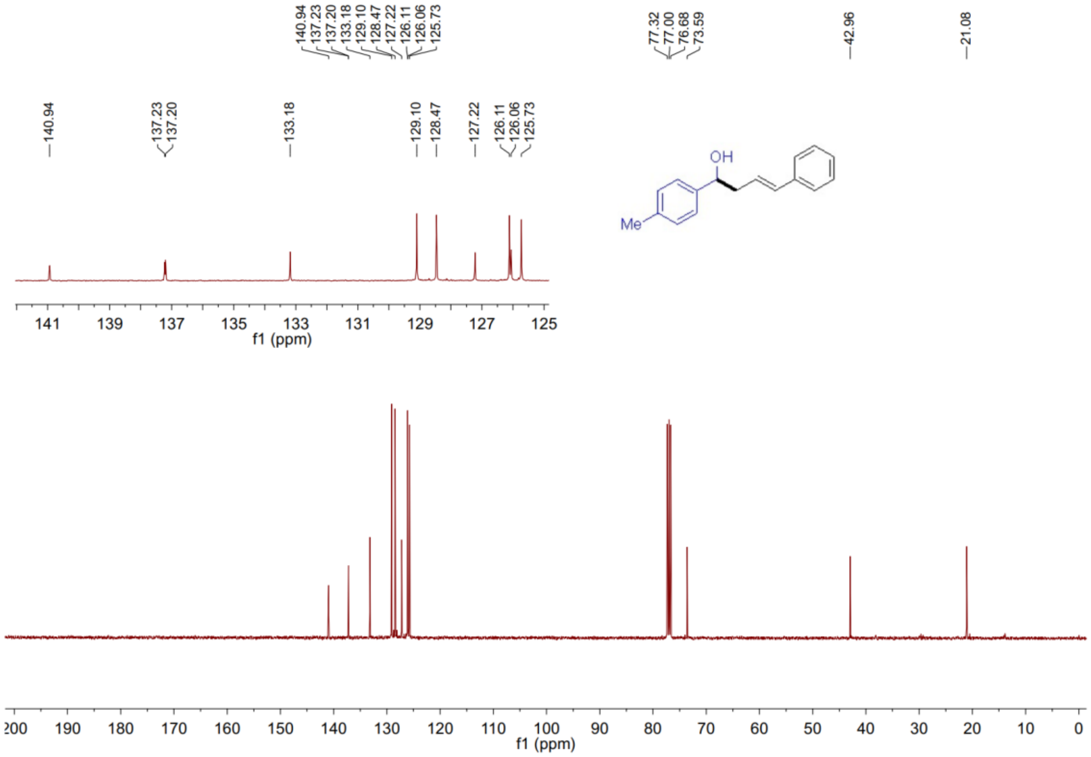

3e ^{1}H NMR

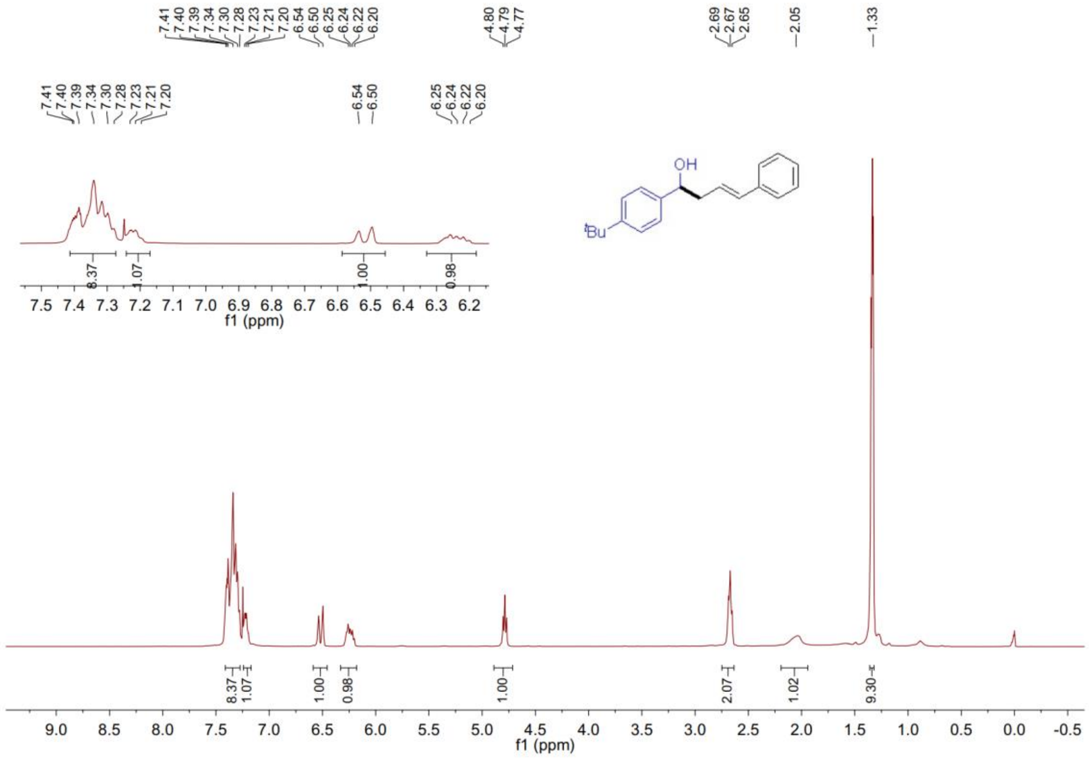

3e ^{13}C NMR

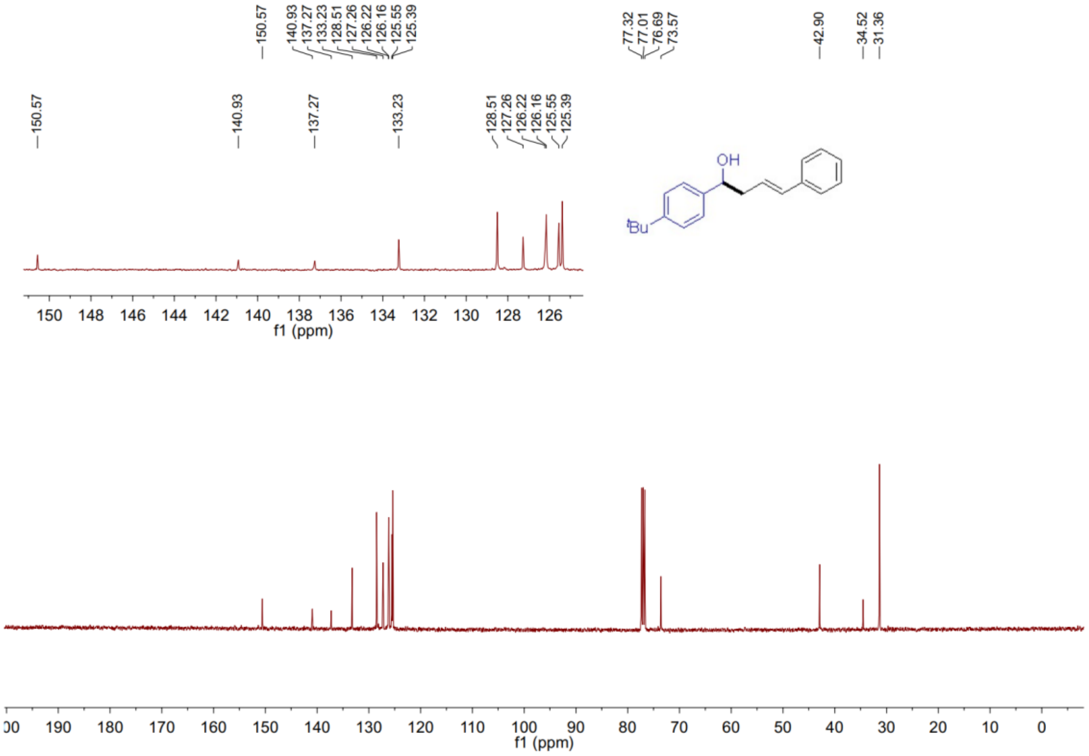

3f ^{1}H NMR

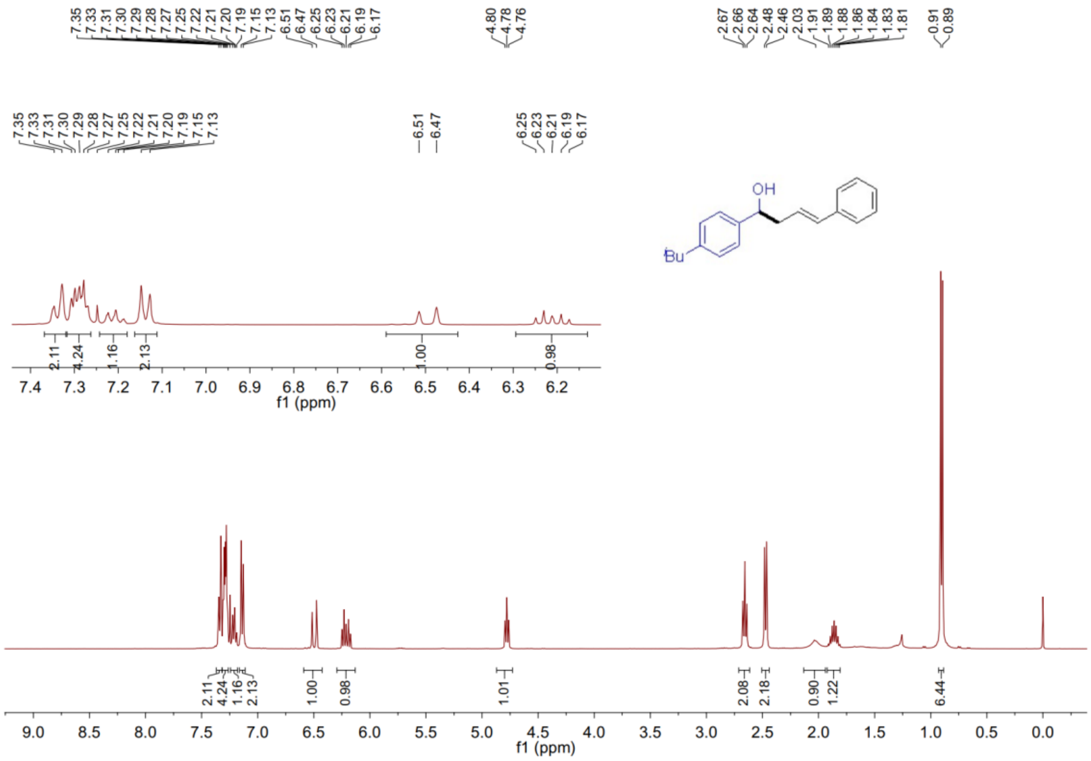

3f ^{13}C NMR

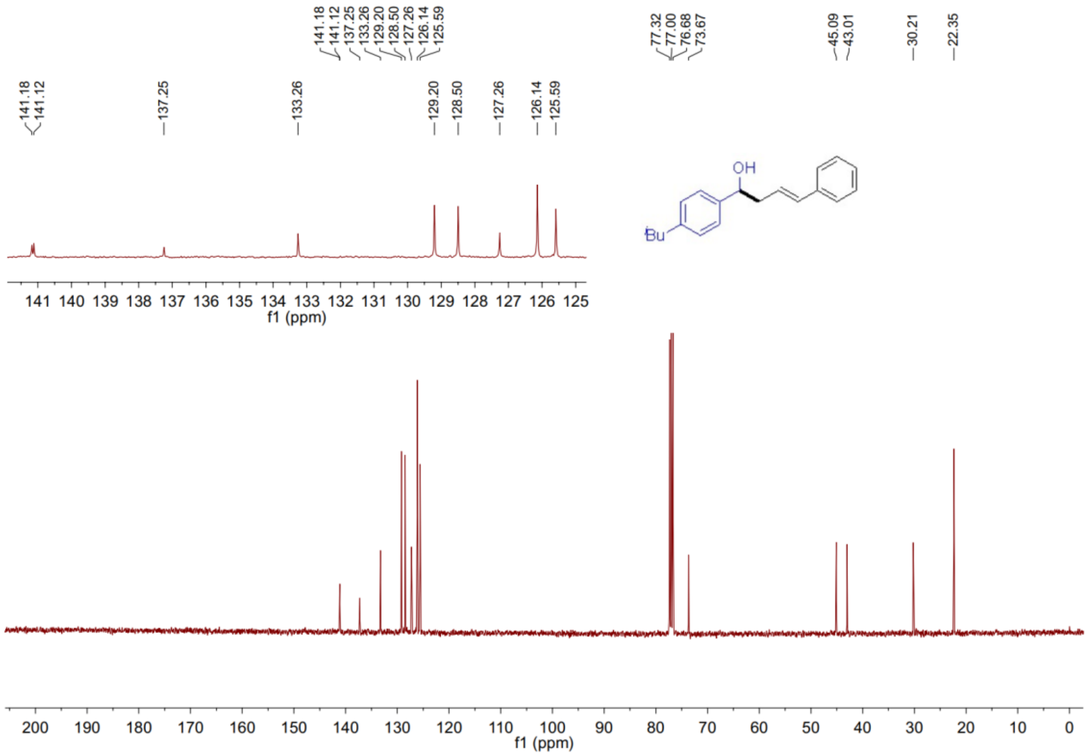

3g ¹H NMR

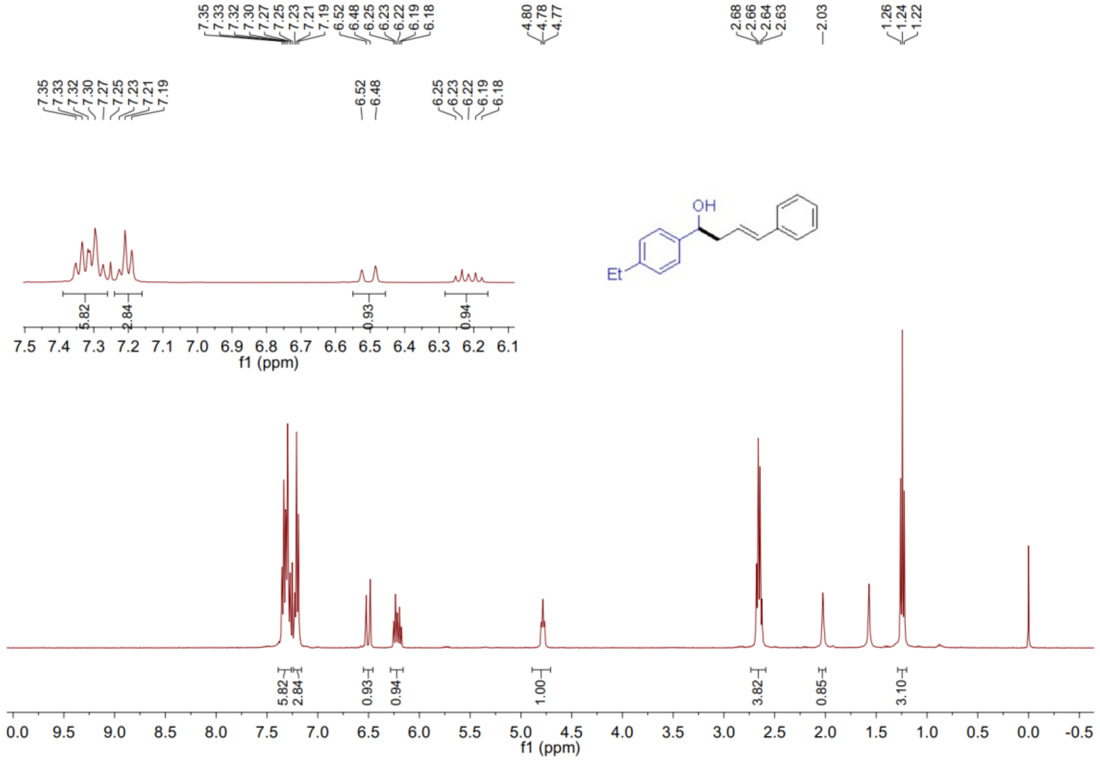

3g ¹³C NMR

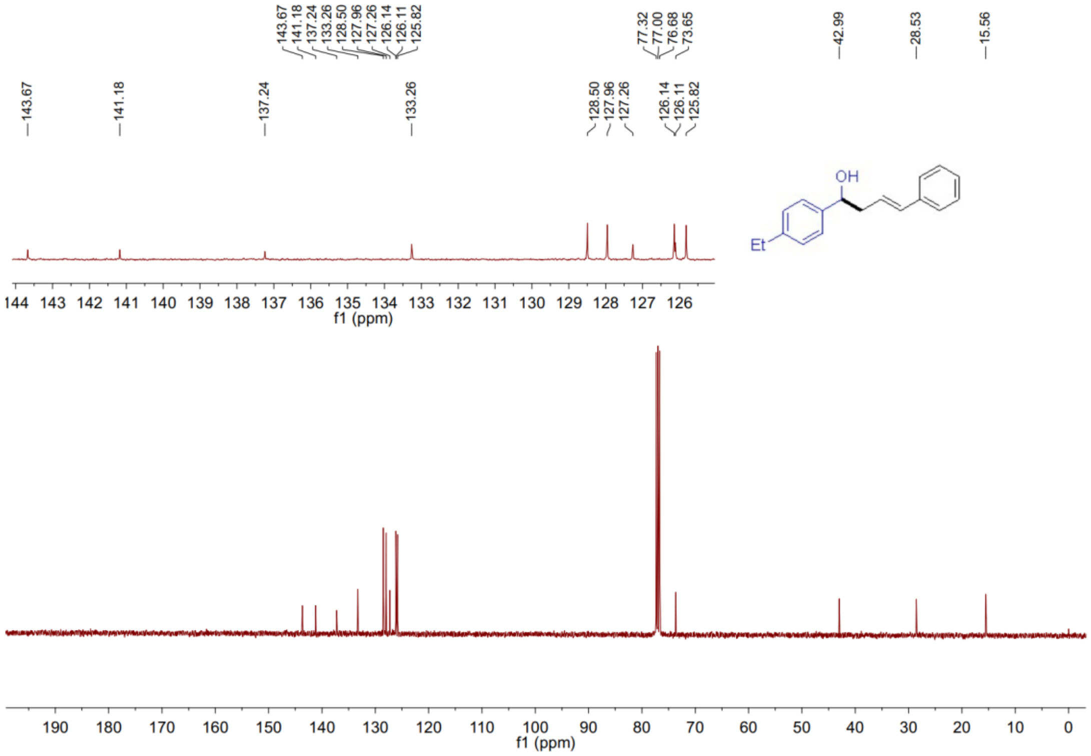

3h ¹H NMR

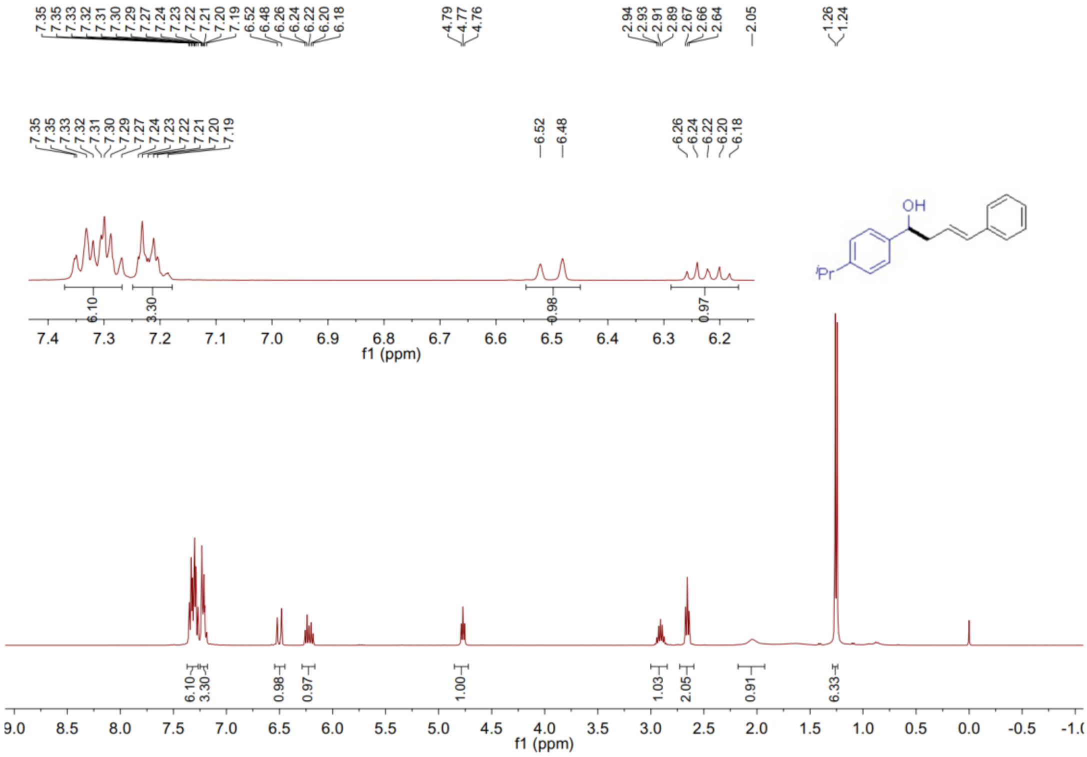

3h ¹³C NMR

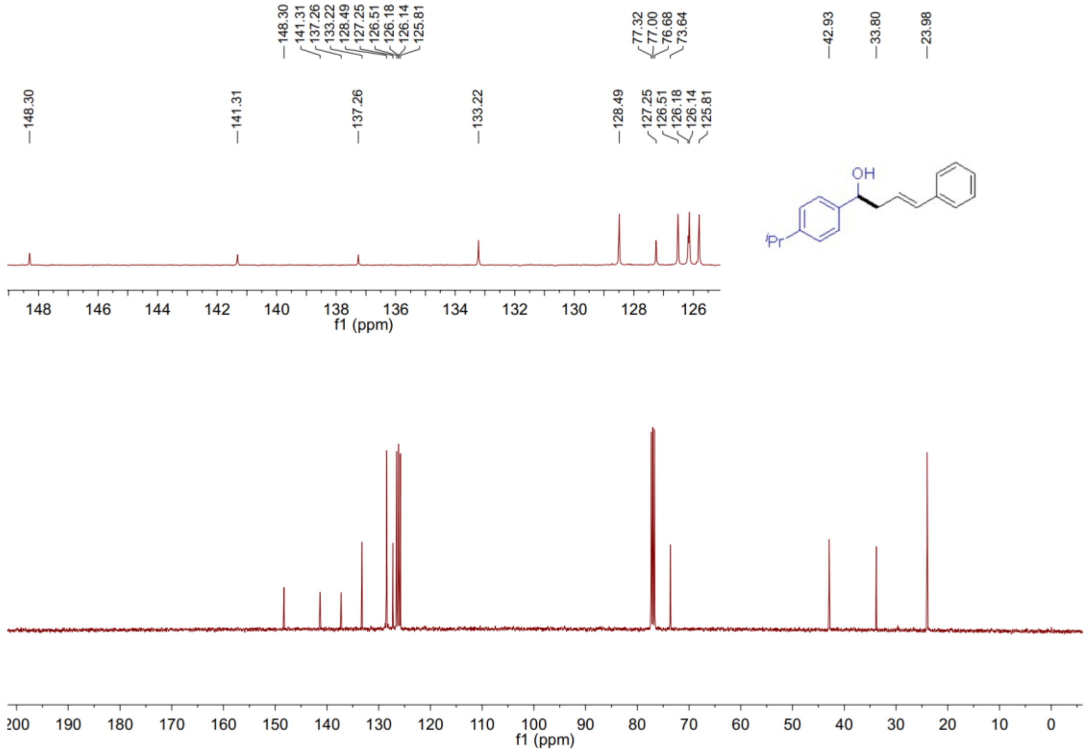

3i ^{1}H NMR

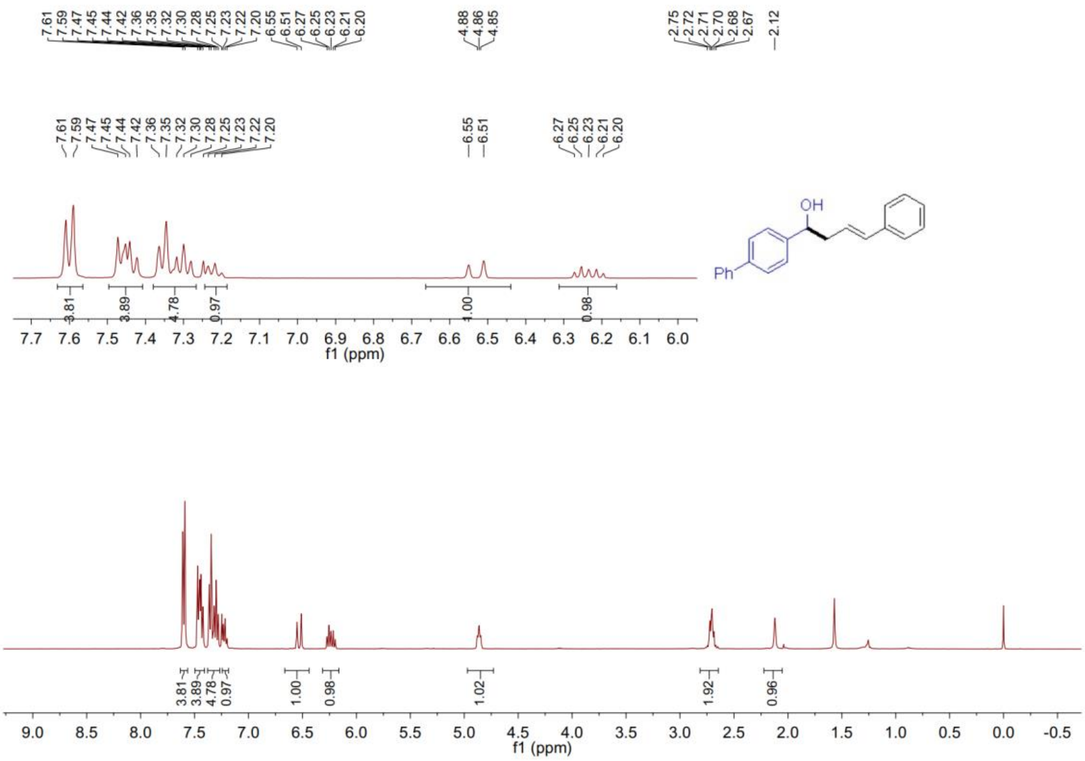

3i ^{13}C NMR

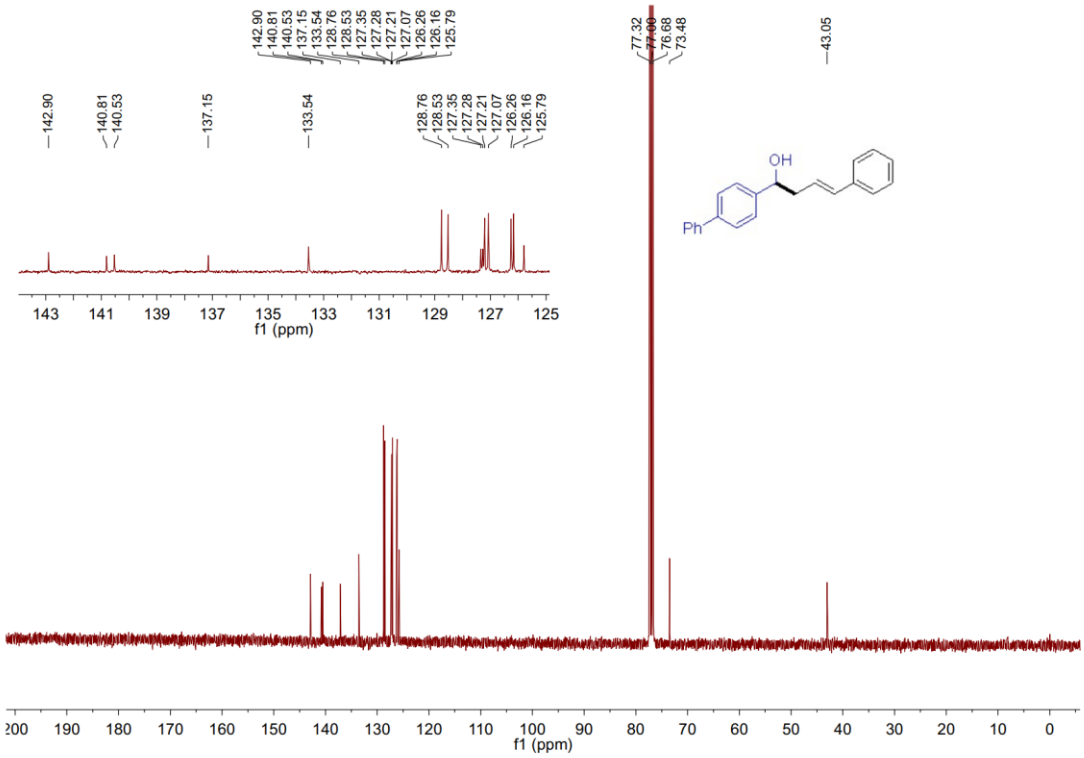

3j ^{1}H NMR

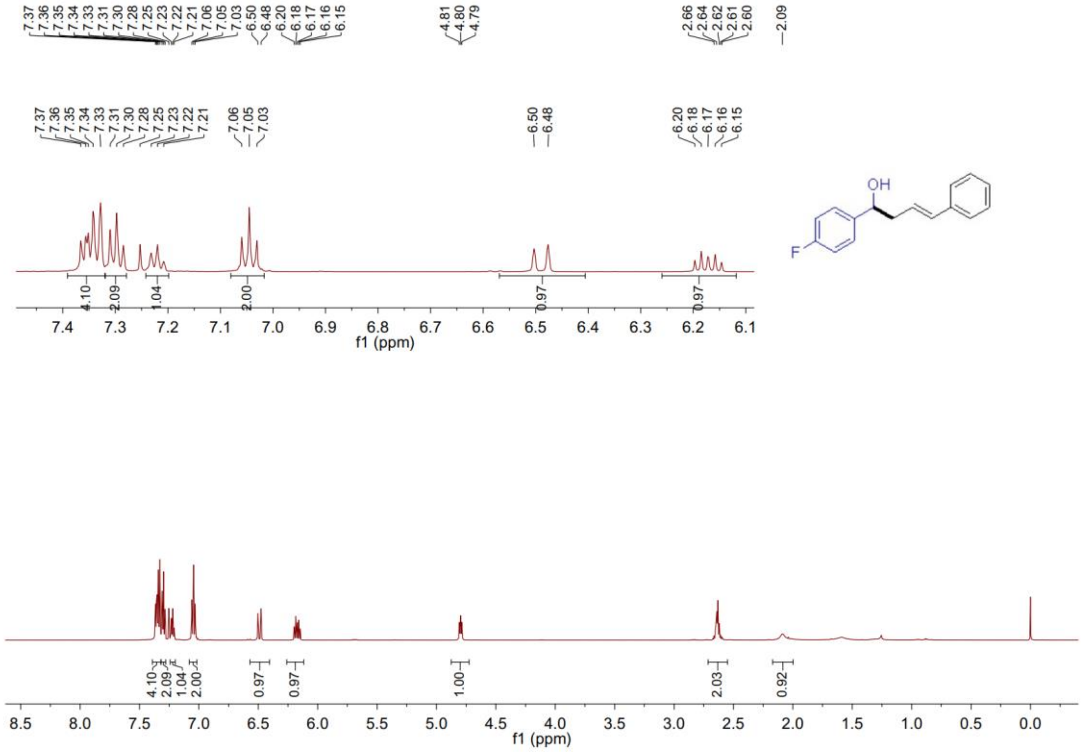

3j ^{13}C NMR

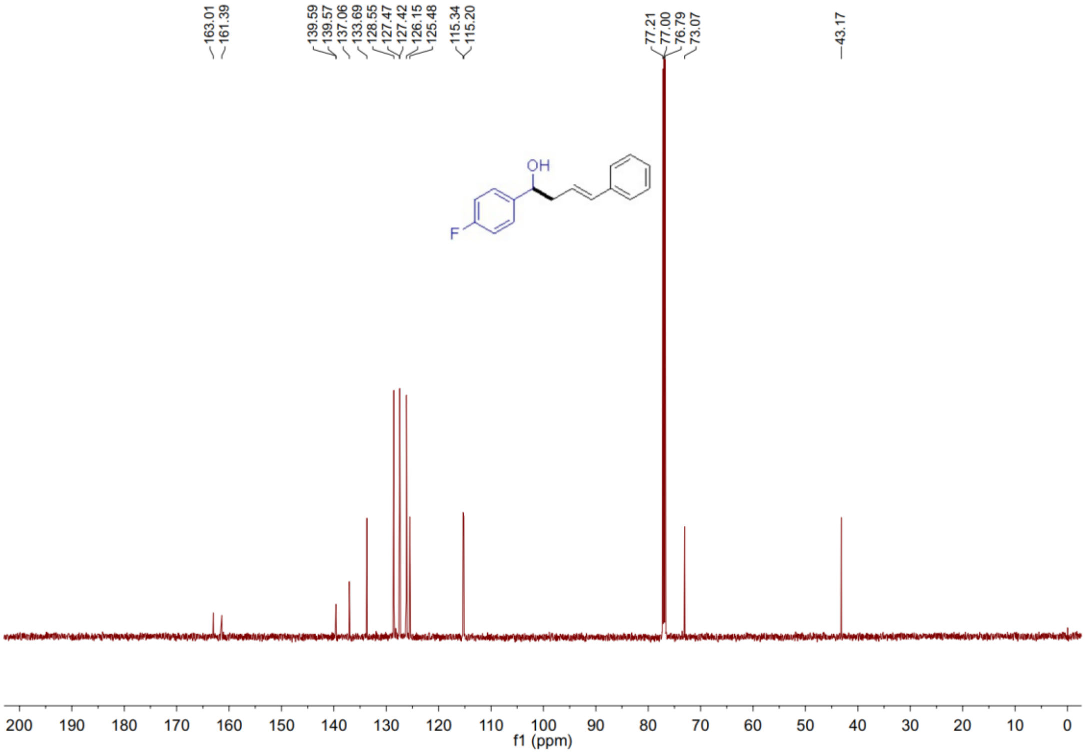

3k ^{1}H NMR

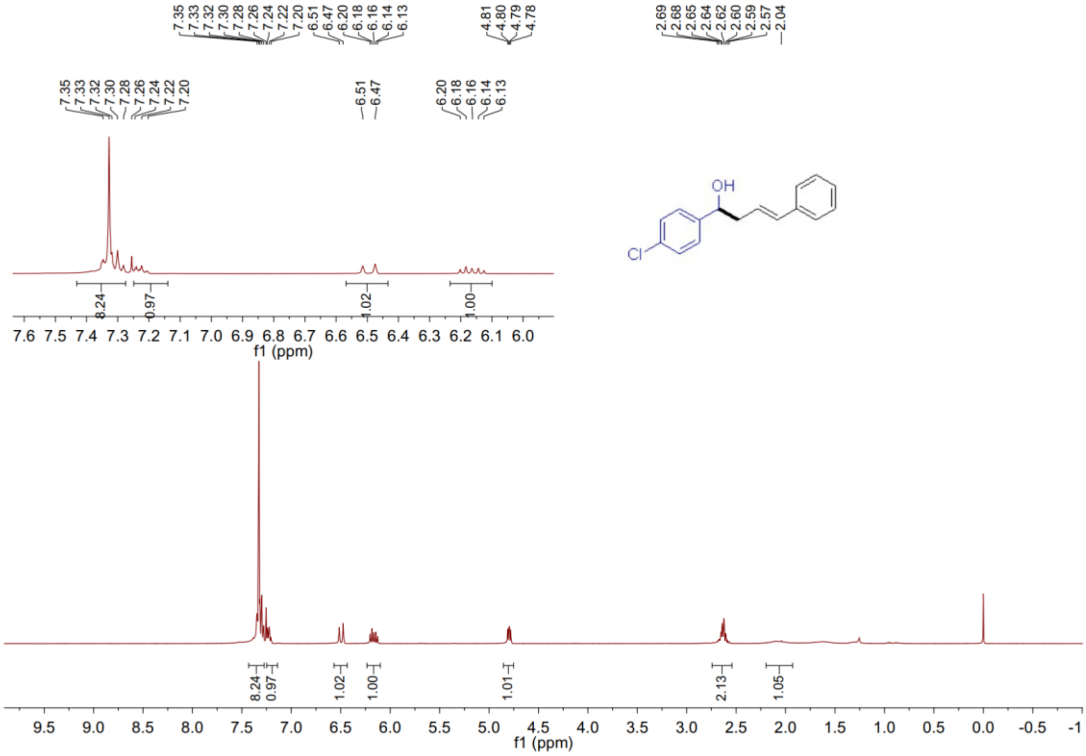

3k ^{13}C NMR

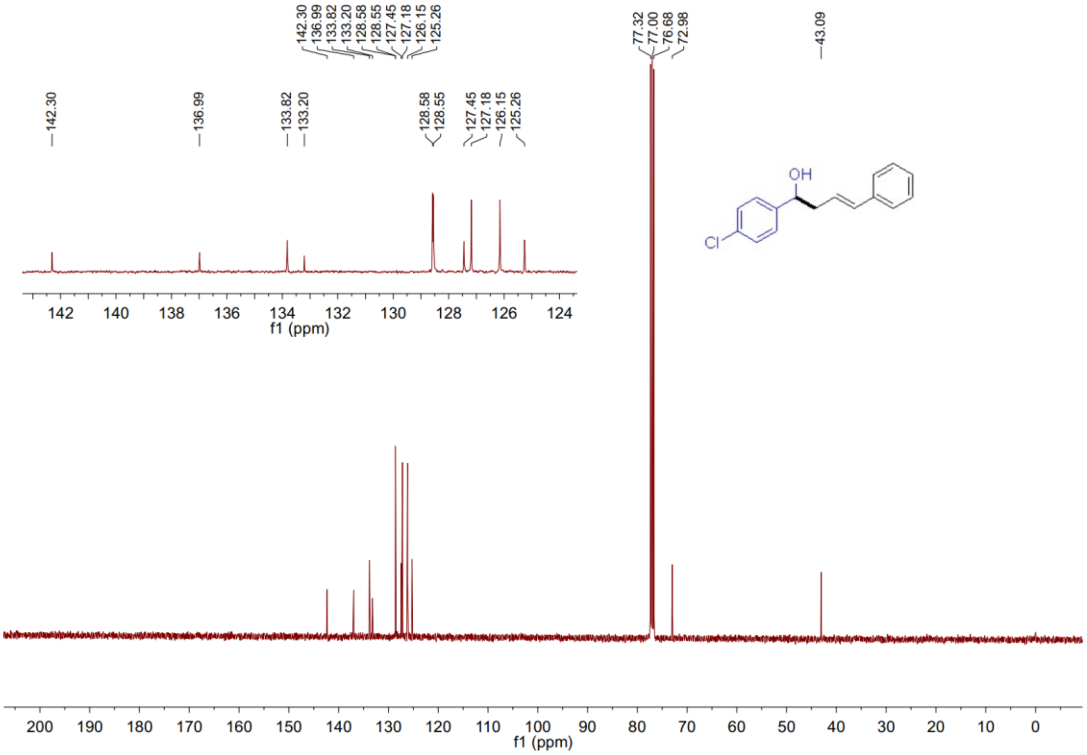

3l ^{1}H NMR

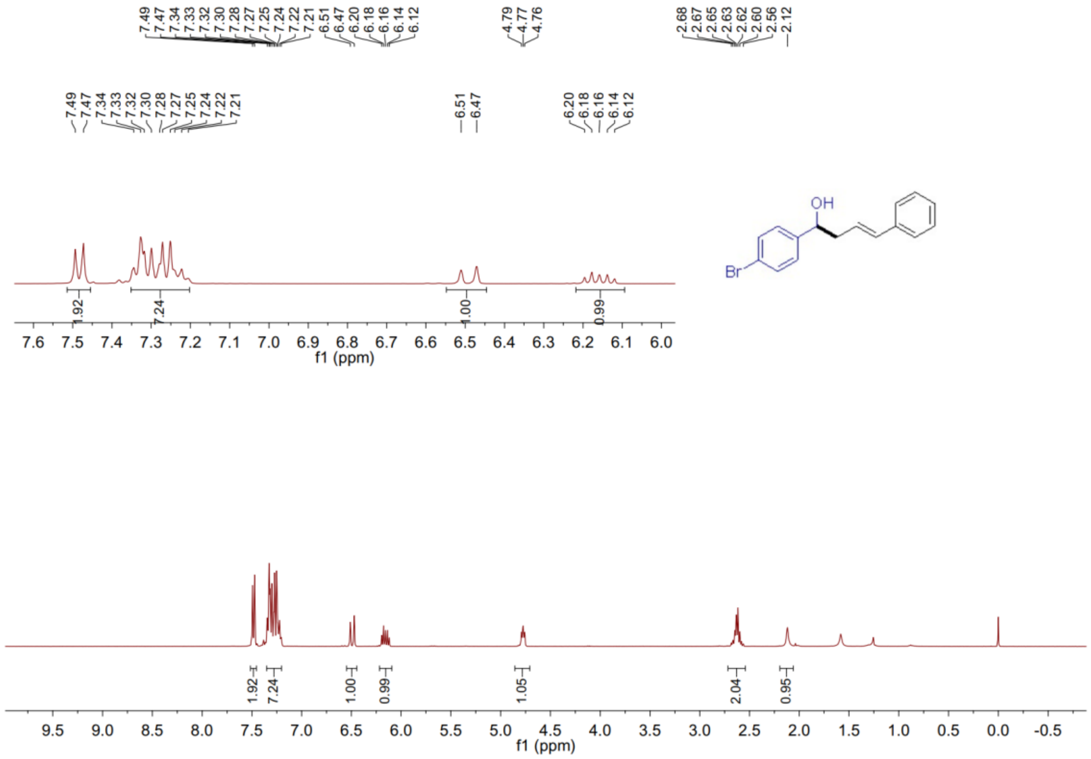

3l ^{13}C NMR

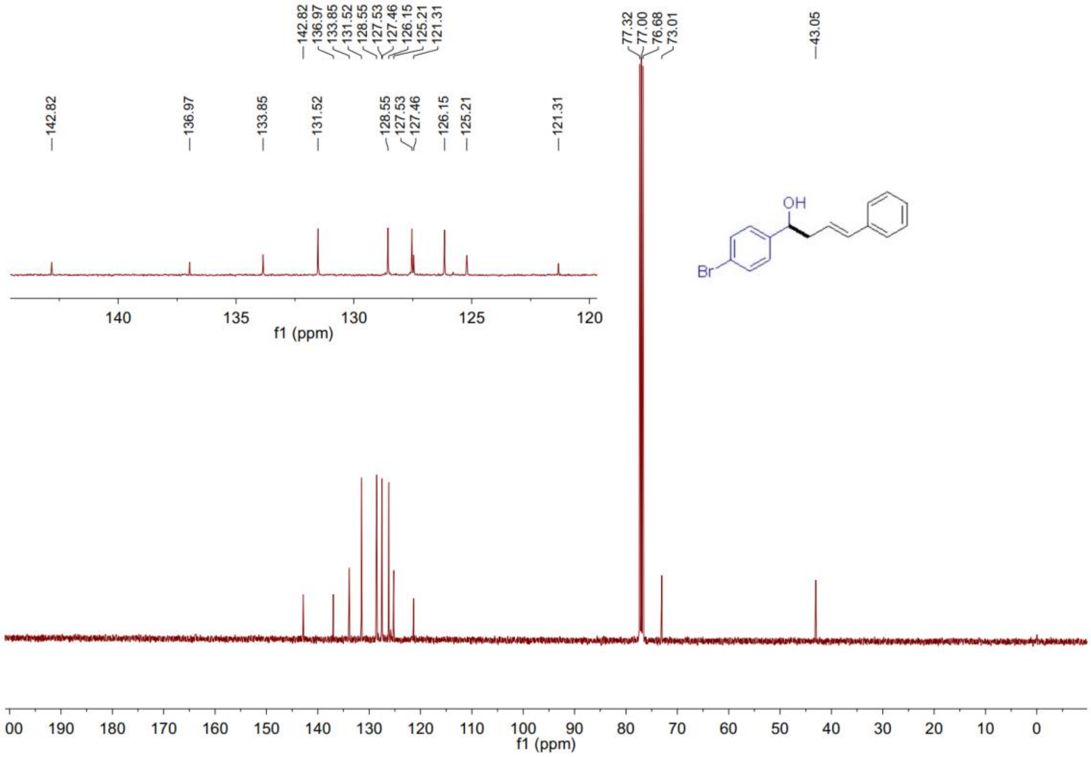

3m ^{1}H NMR

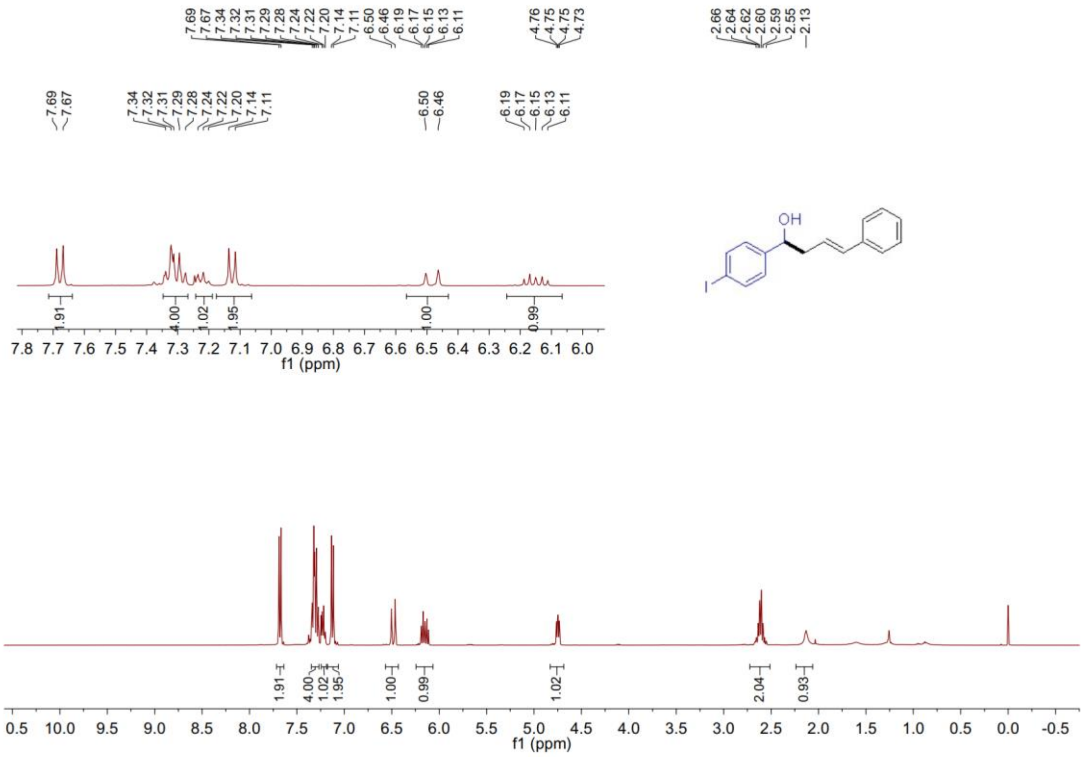

3m ^{13}C NMR

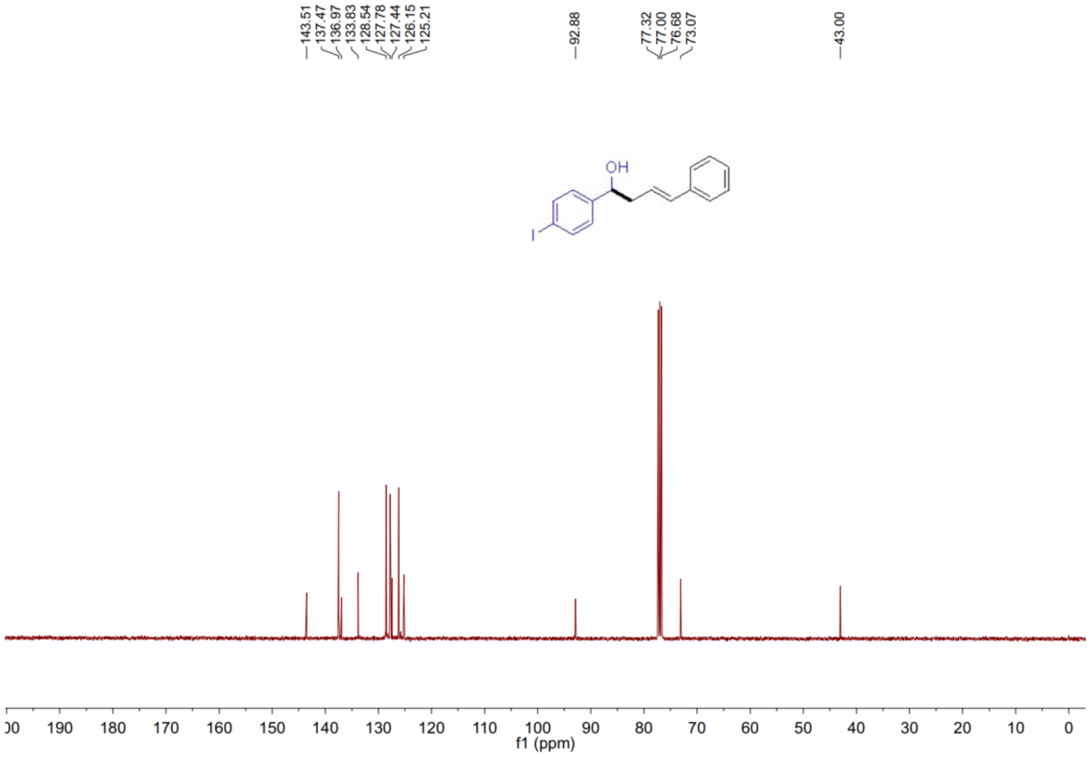

3n ^1H NMR

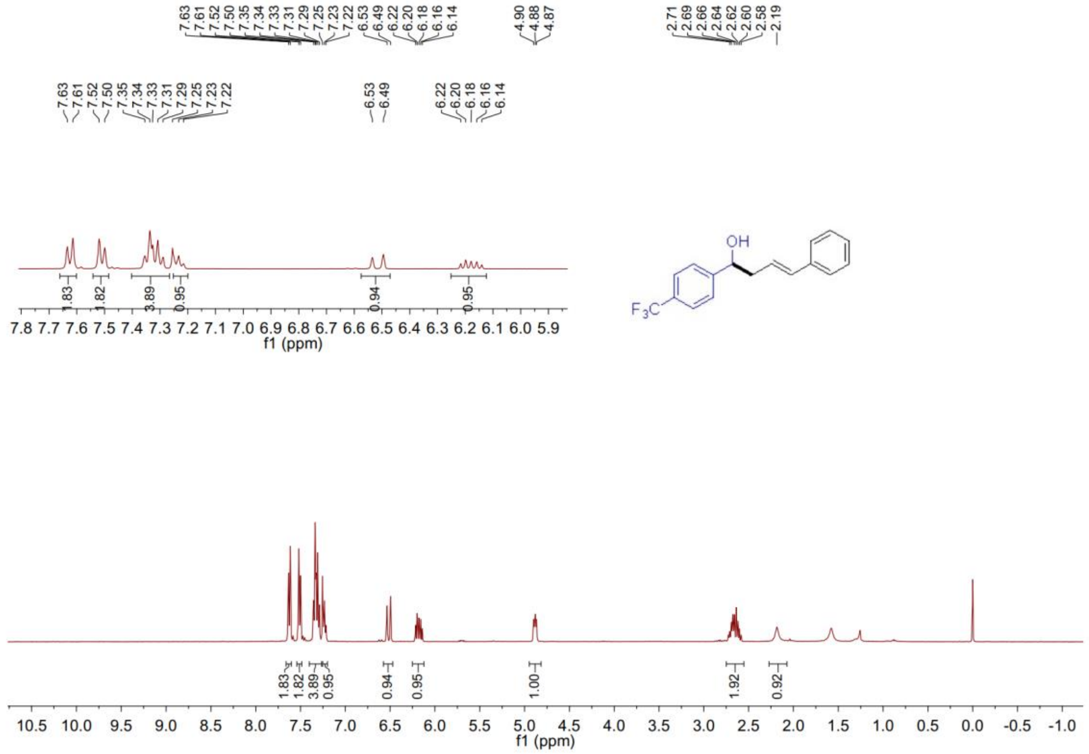

3n ^{13}C NMR

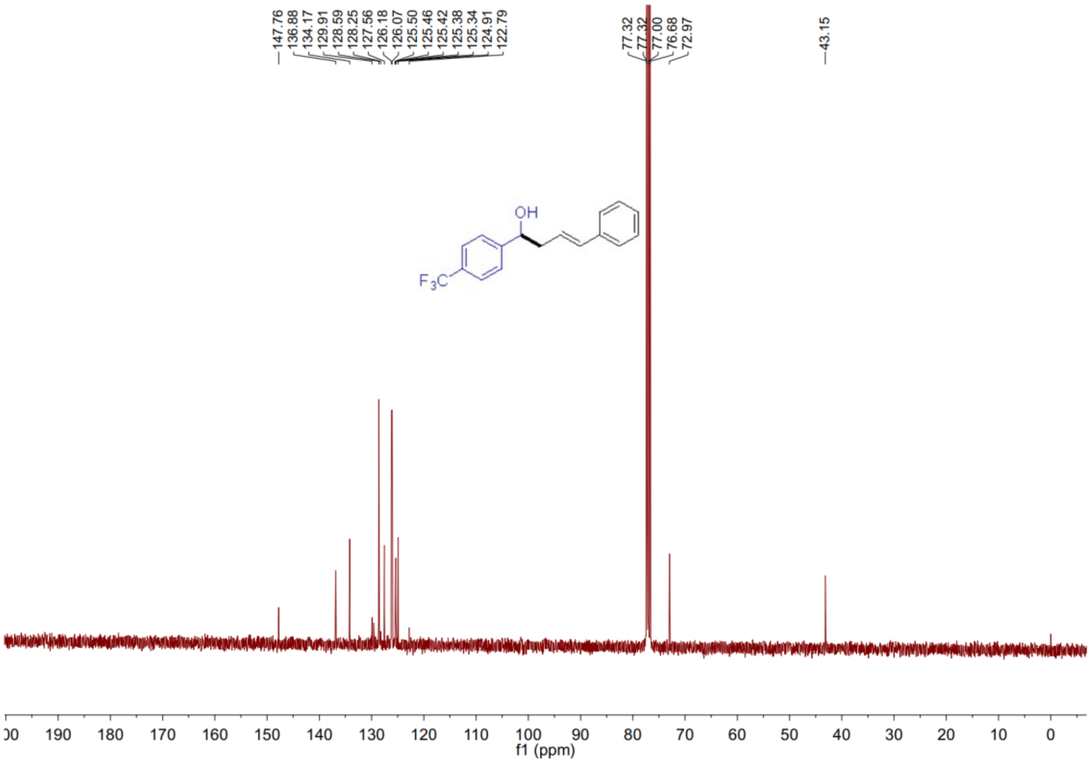

3o ^{1}H NMR

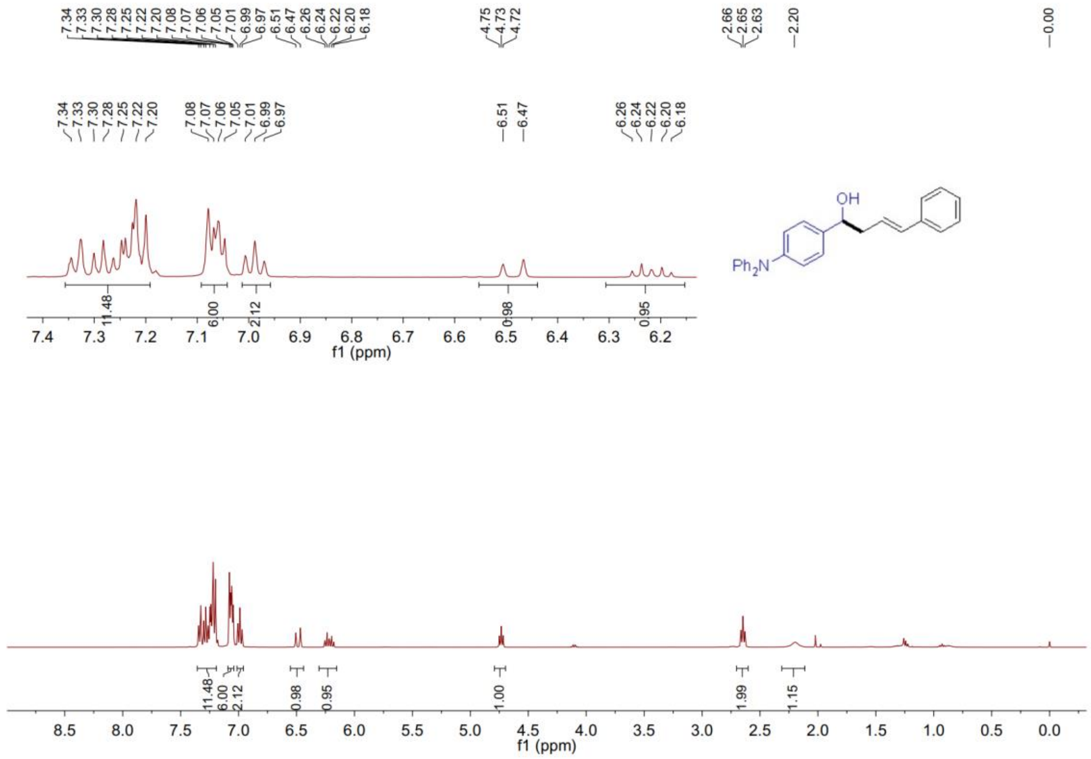

3o ^{13}C NMR

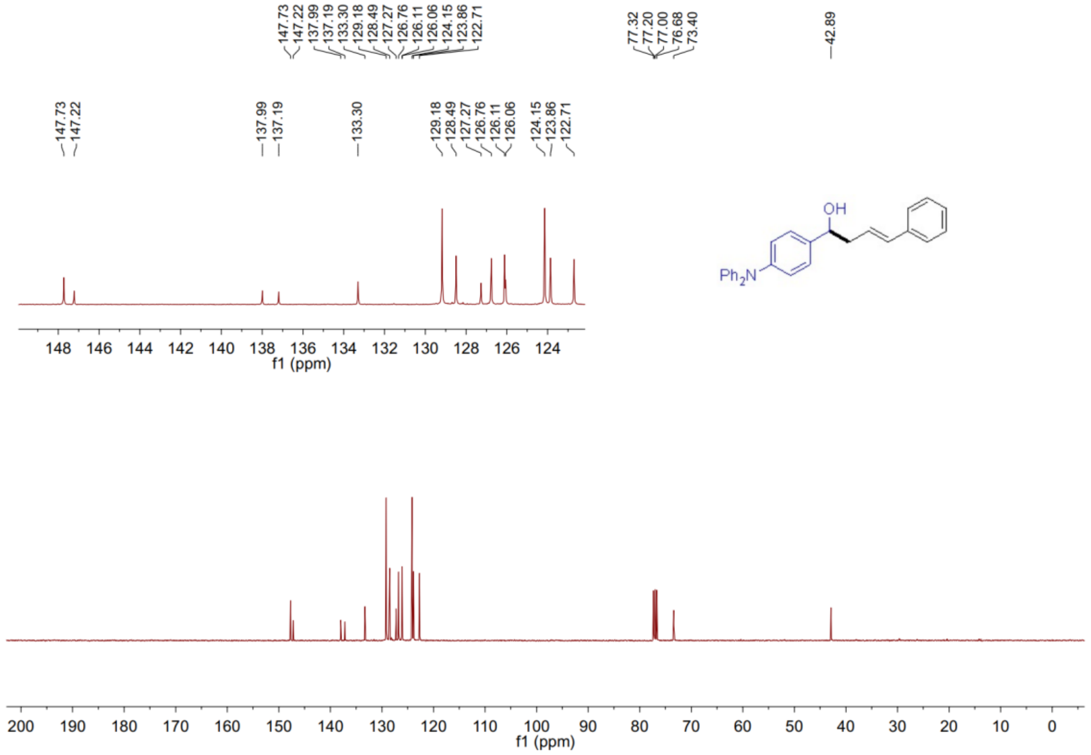

3p ^{1}H NMR

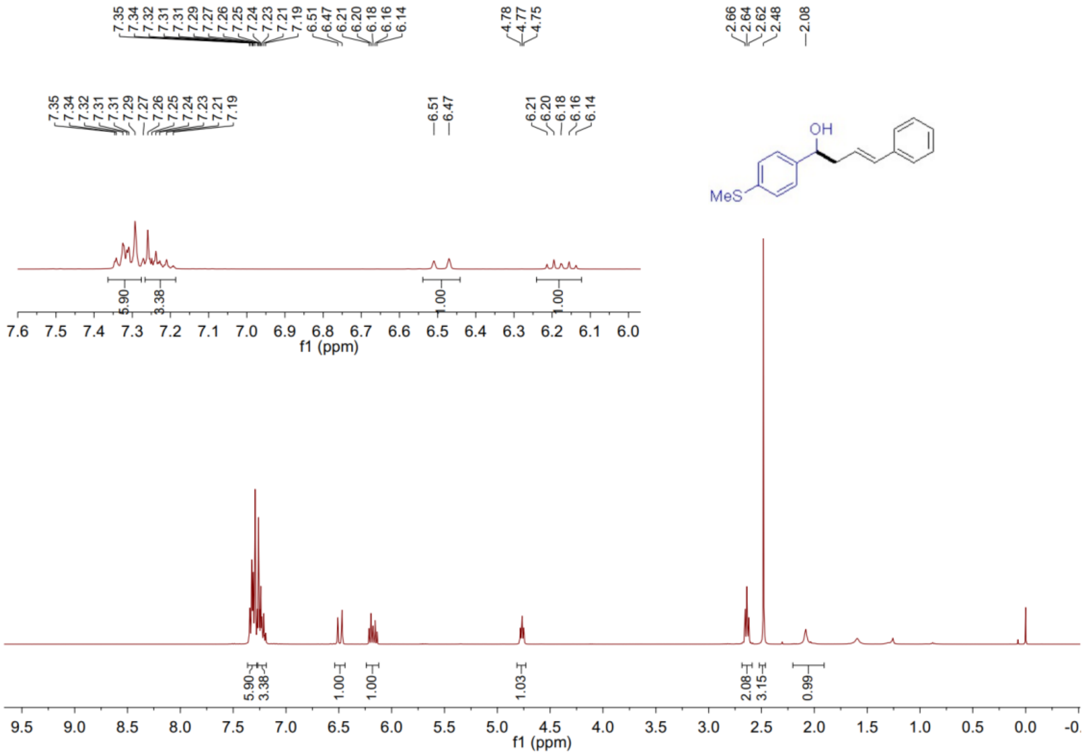

3p ^{13}C NMR

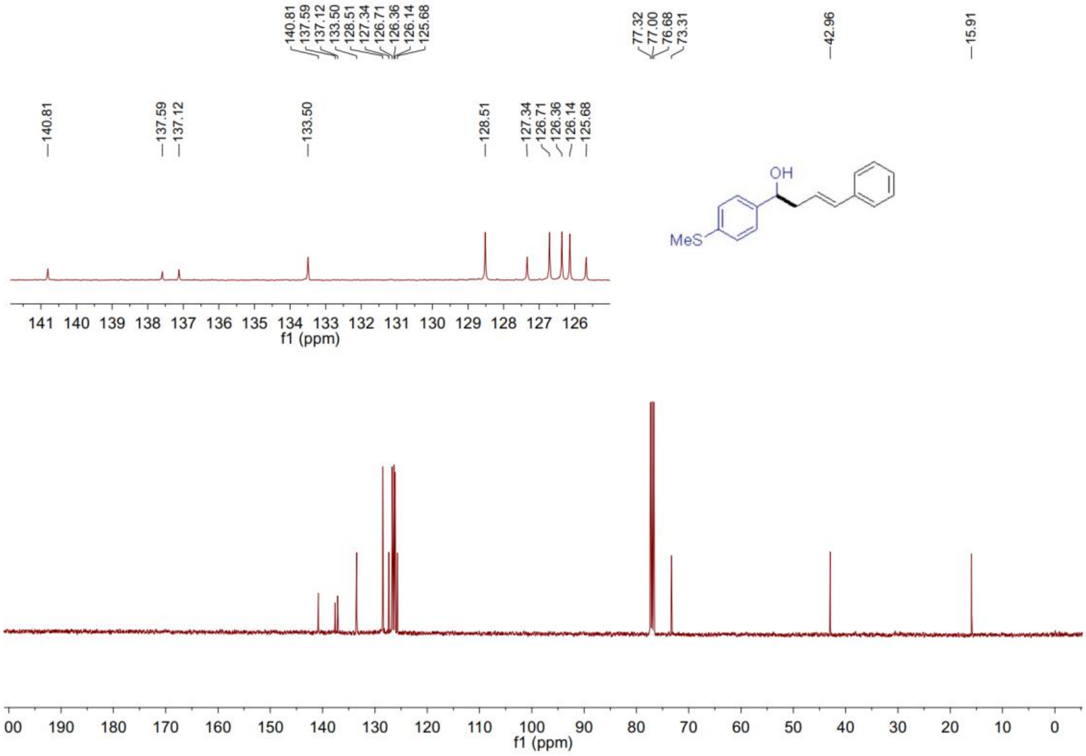

3q ^{1}H NMR

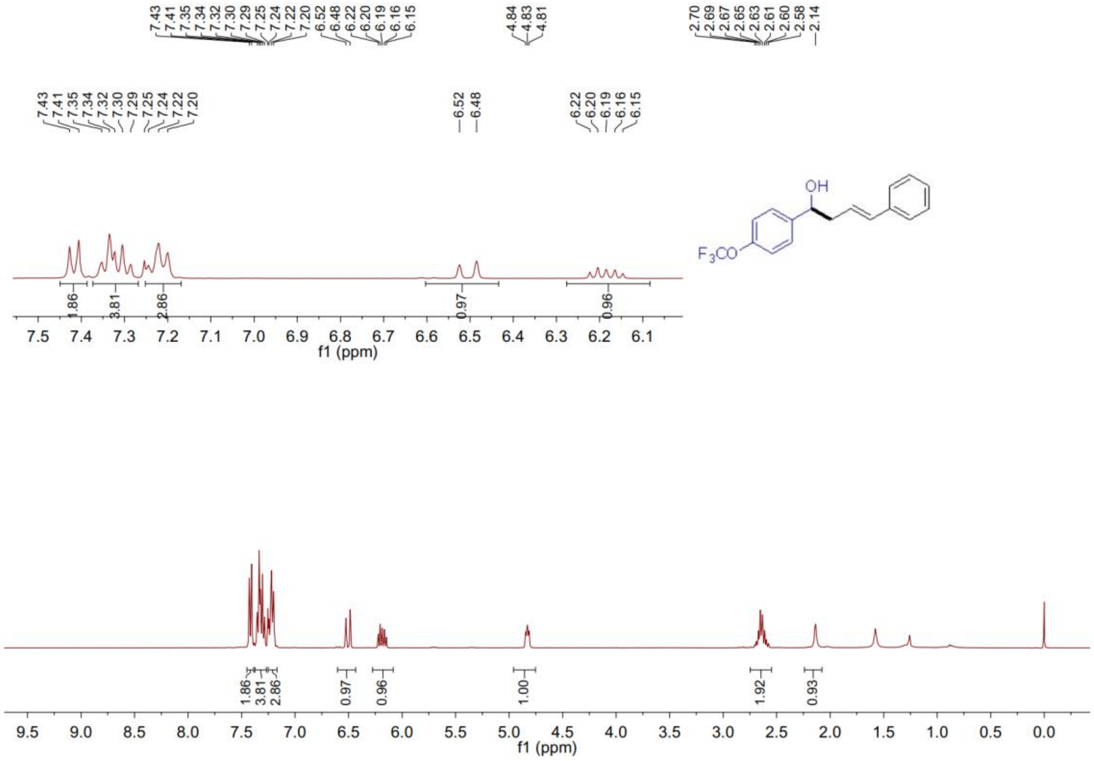

3q ^{13}C NMR

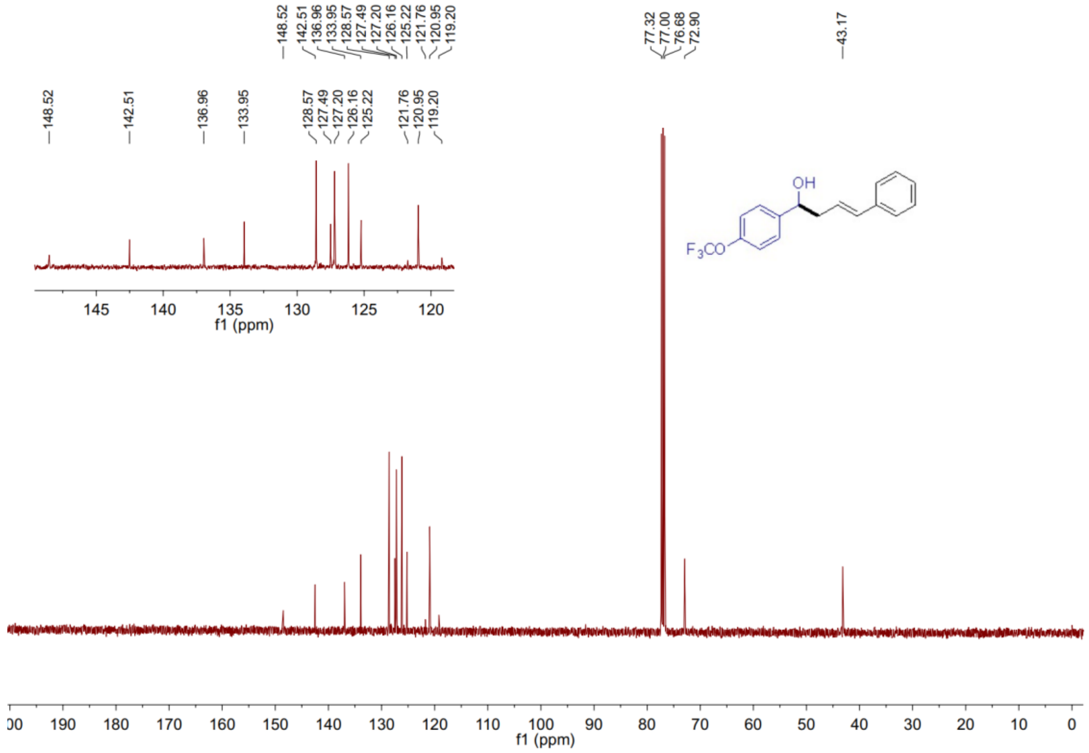

3r ^{1}H NMR

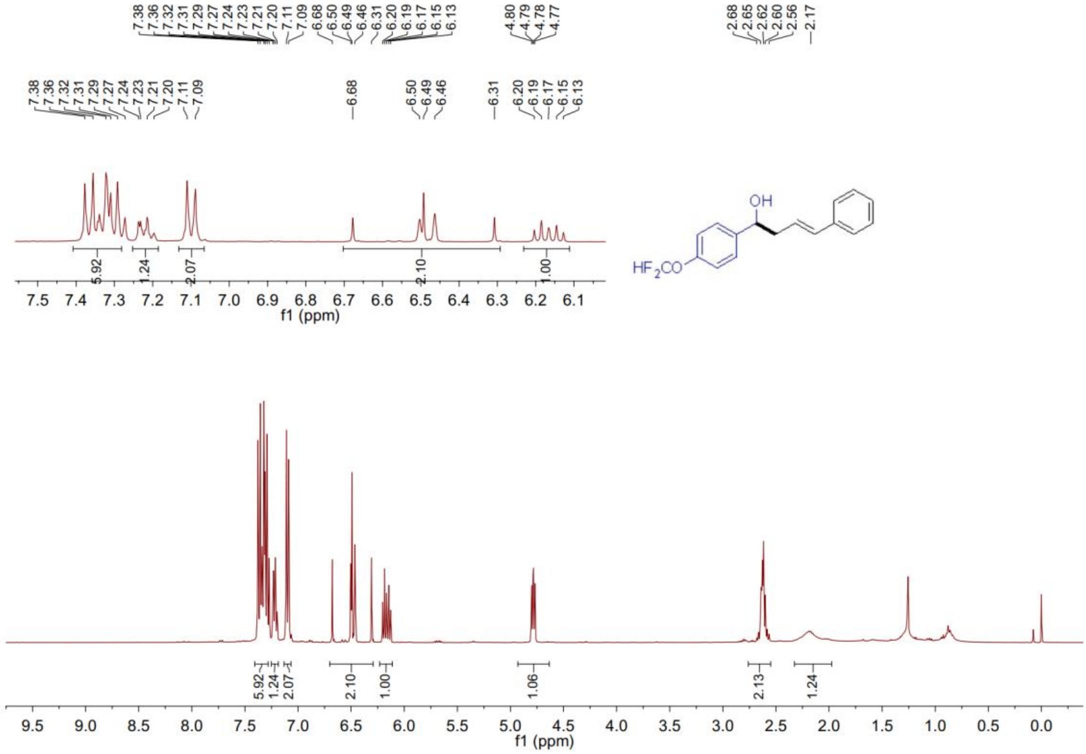

3r ^{13}C NMR

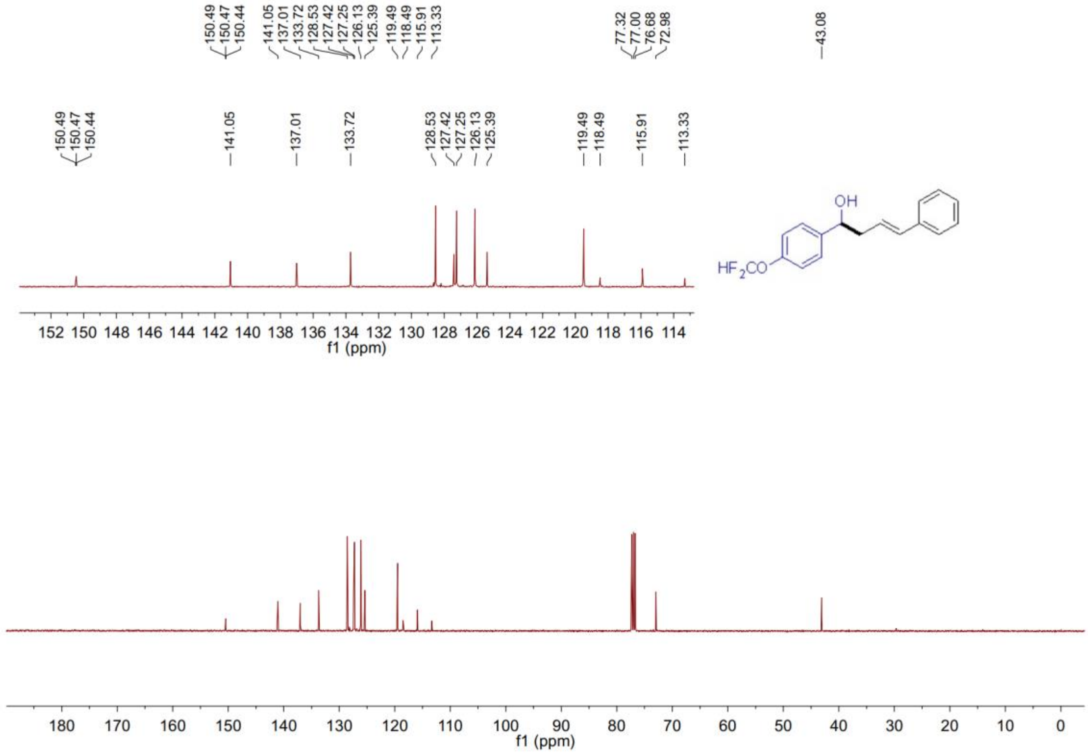

3s ^{1}H NMR

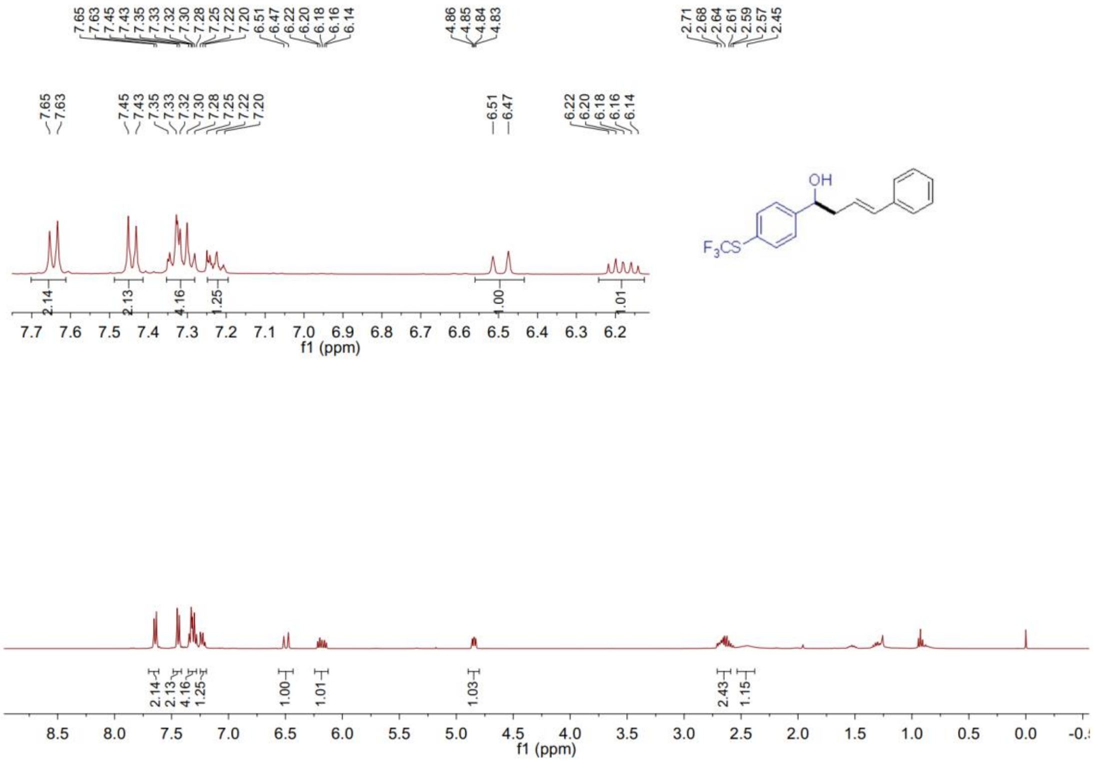

3s ^{13}C NMR

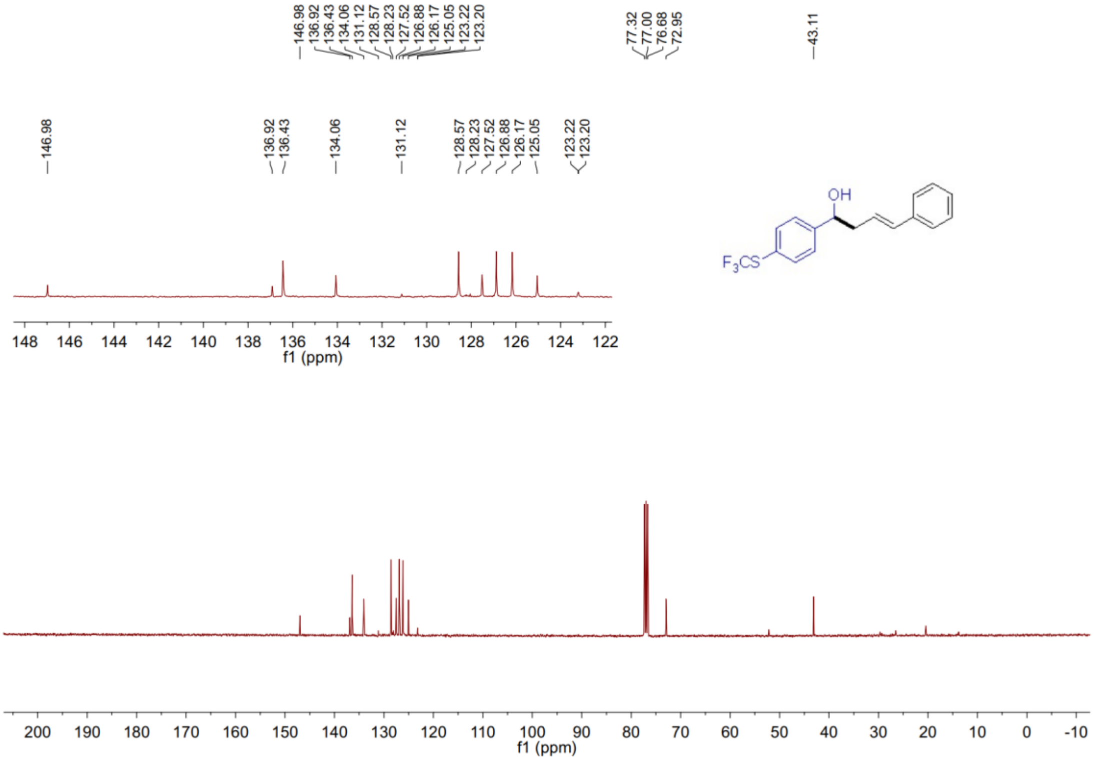

3t ^{1}H NMR

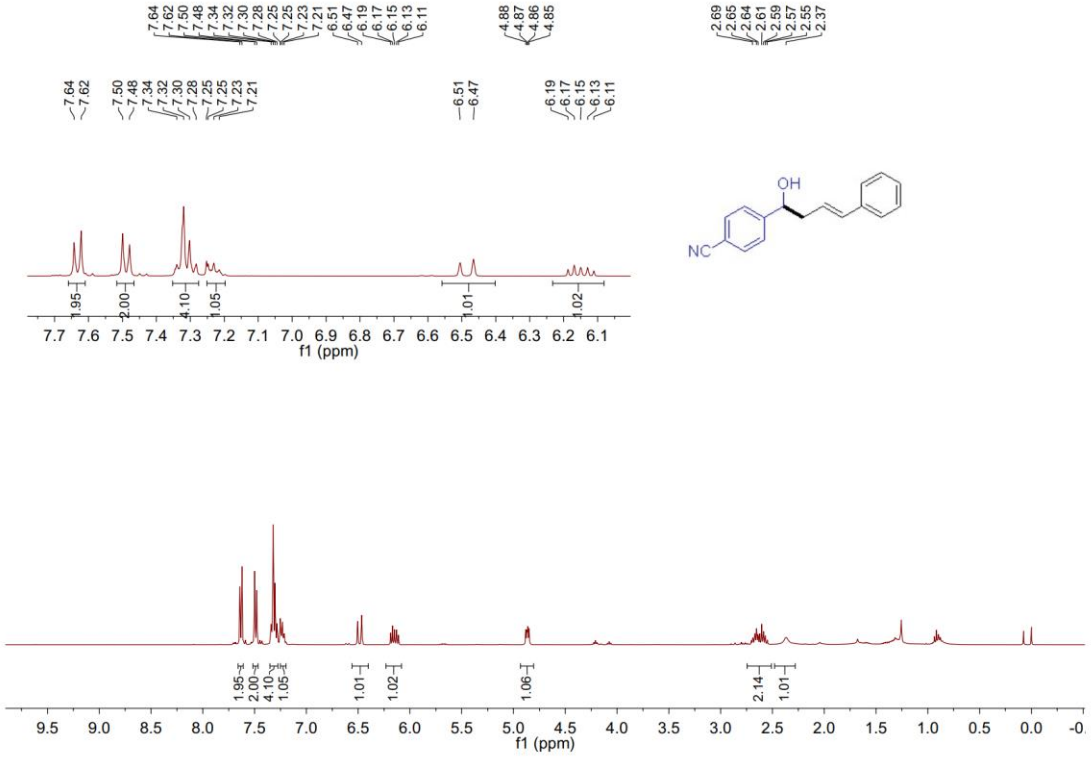

3t ^{13}C NMR

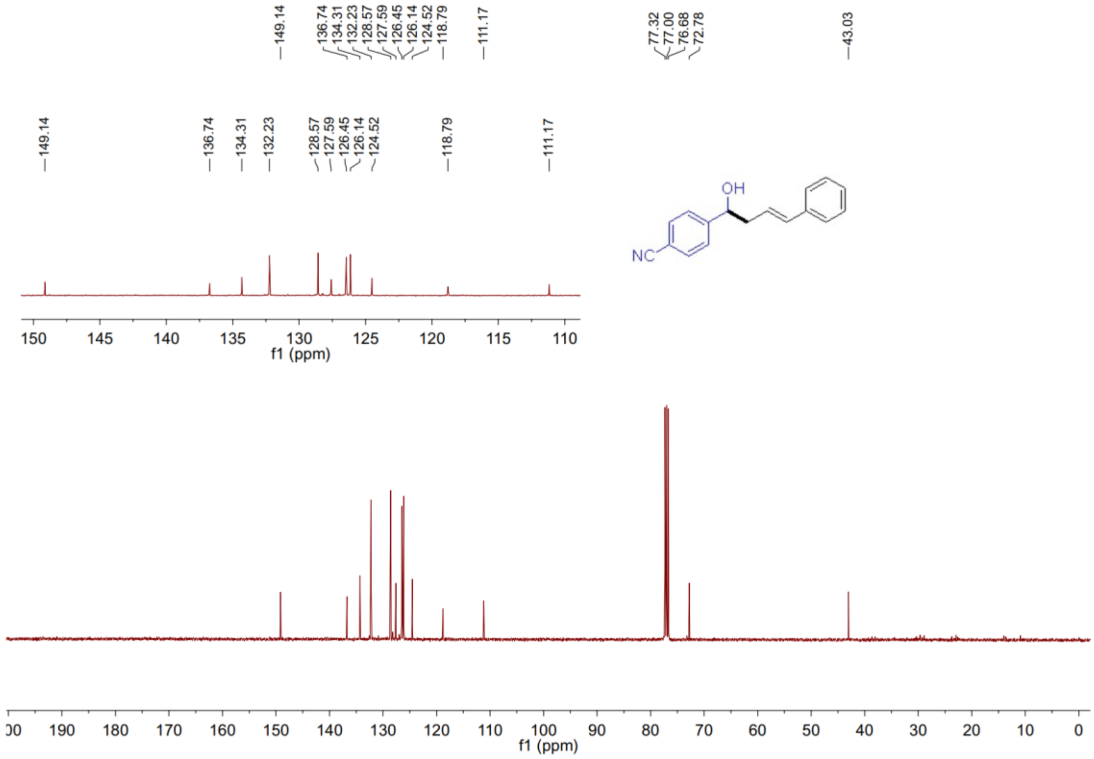

3u 1H NMR

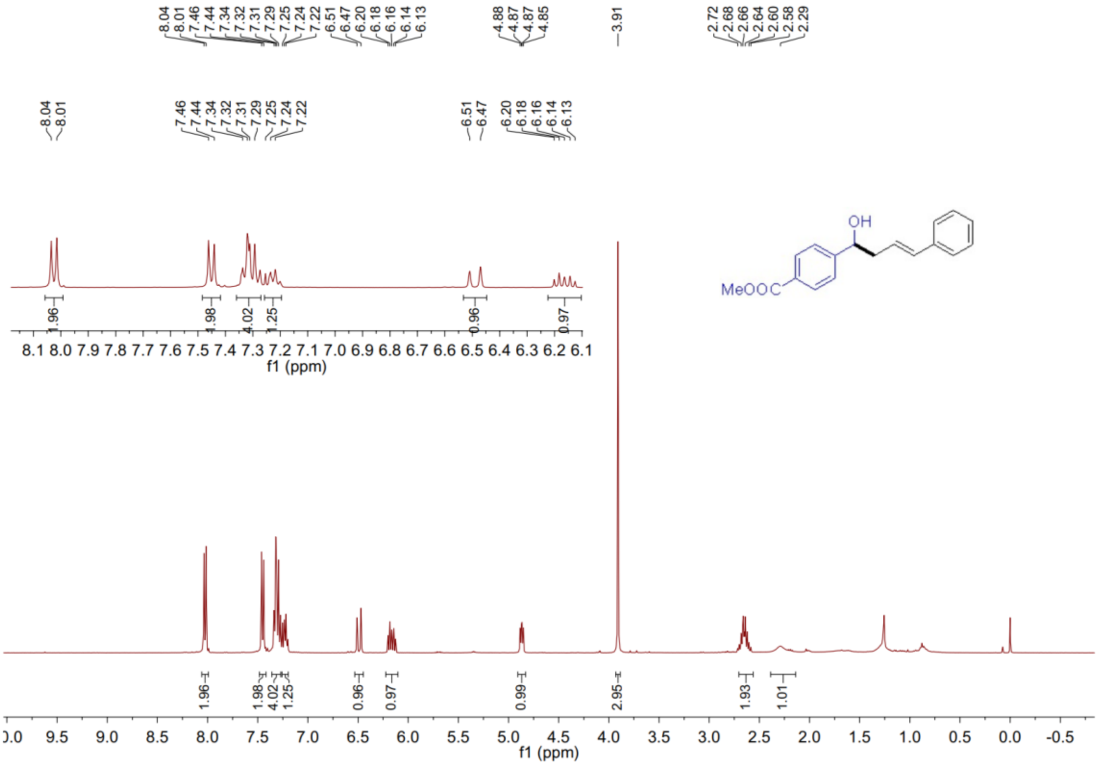

3u 13C NMR

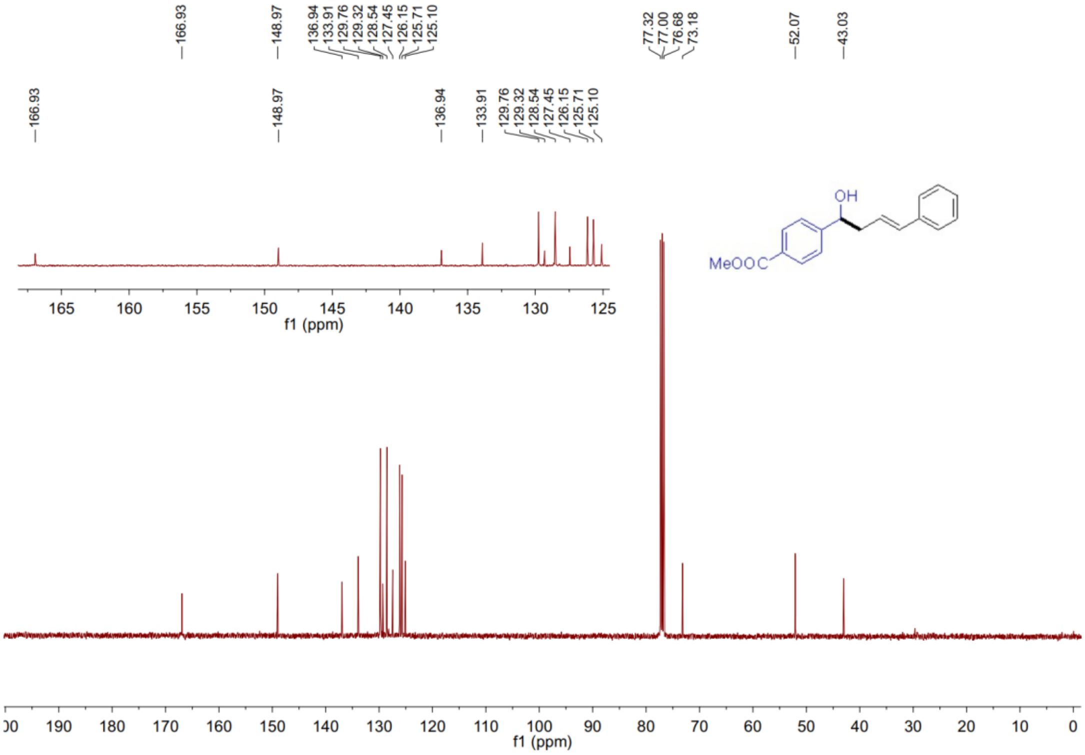

3v ^{1}H NMR

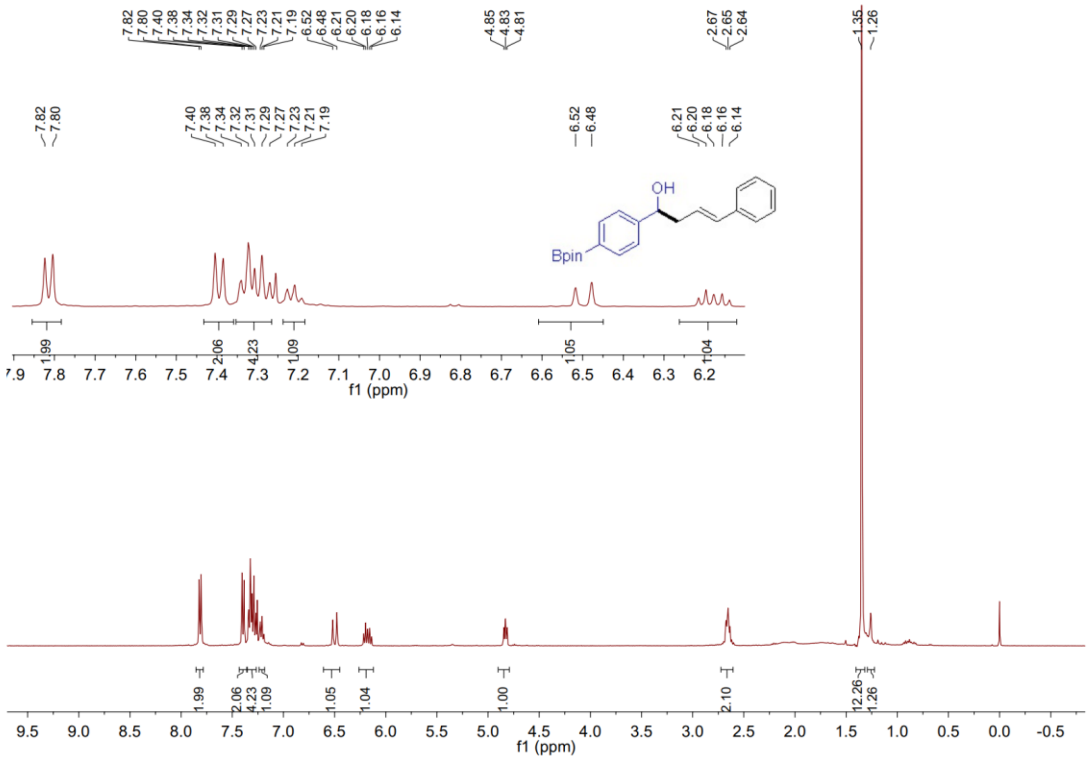

3v ^{13}C NMR

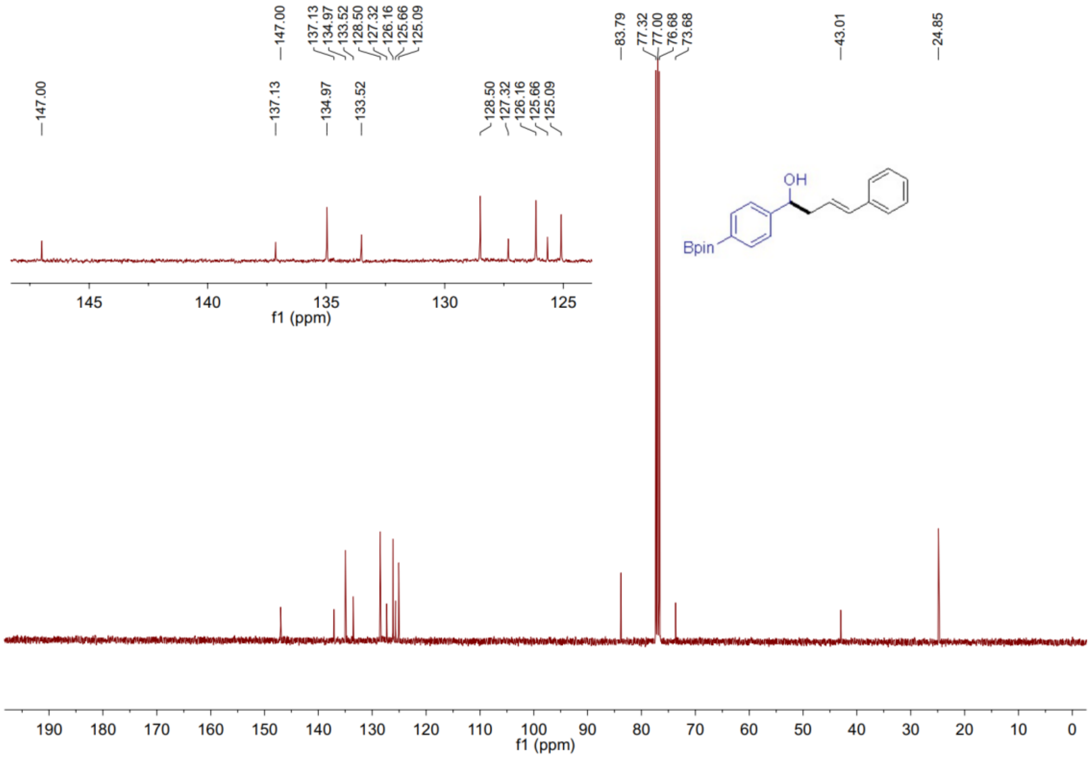

3w ^{1}H NMR

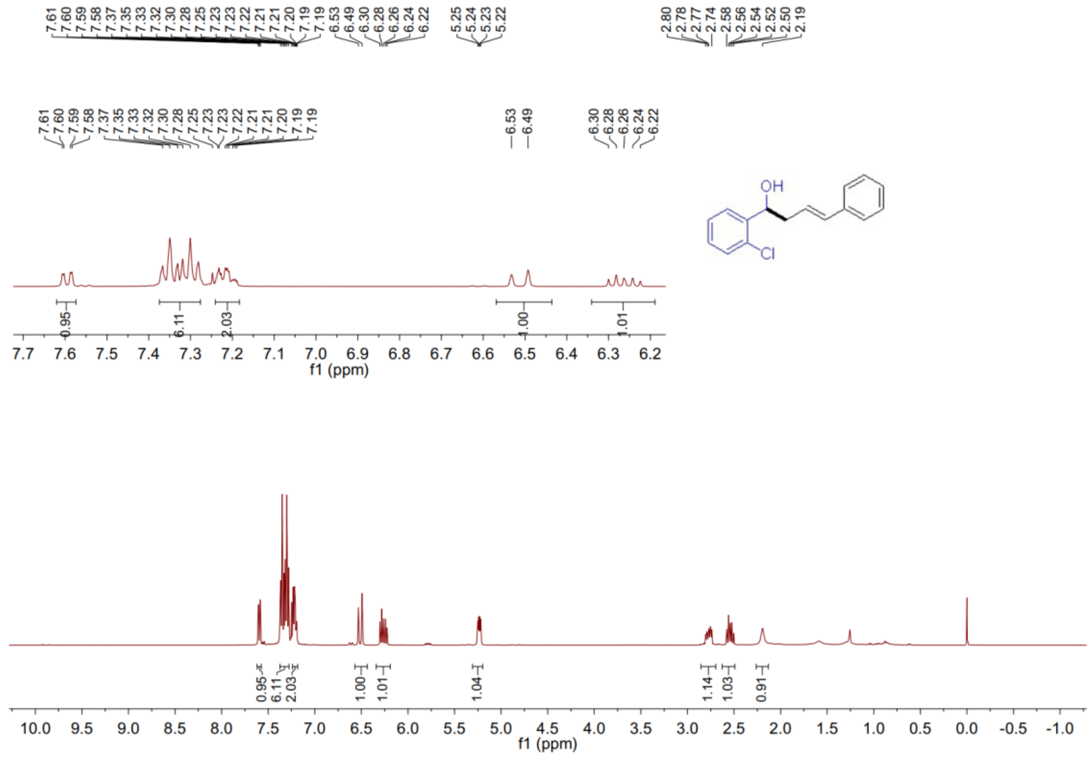

3w ^{13}C NMR

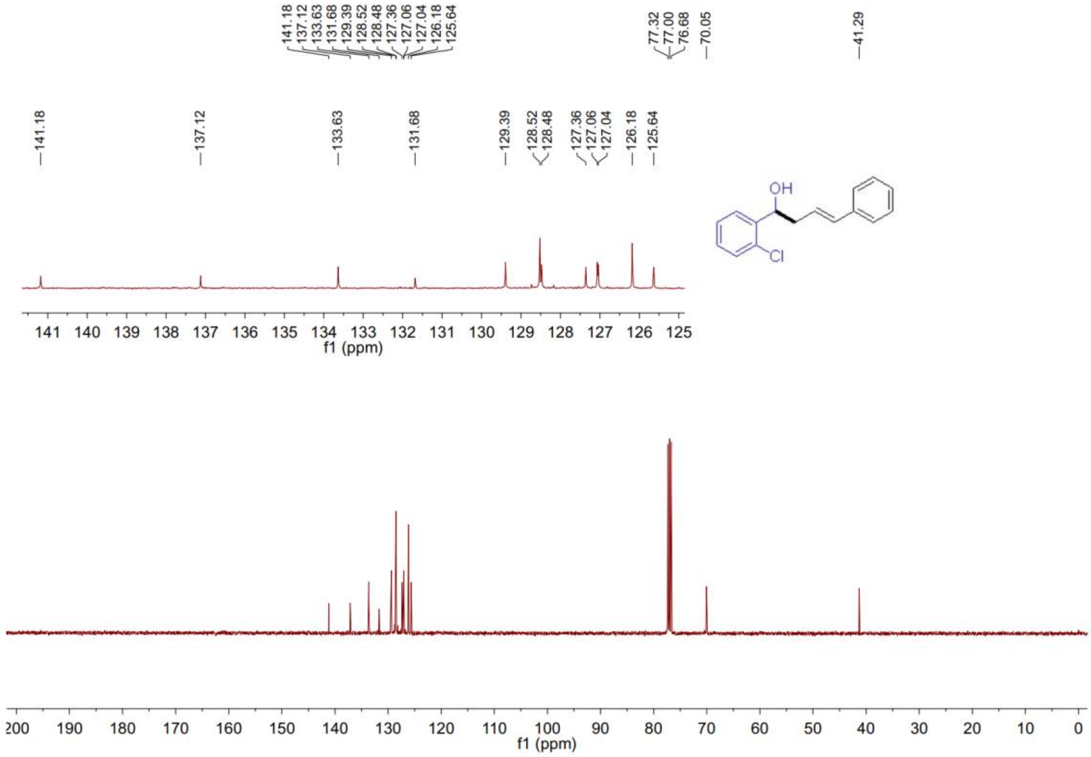

3x ^{1}H NMR

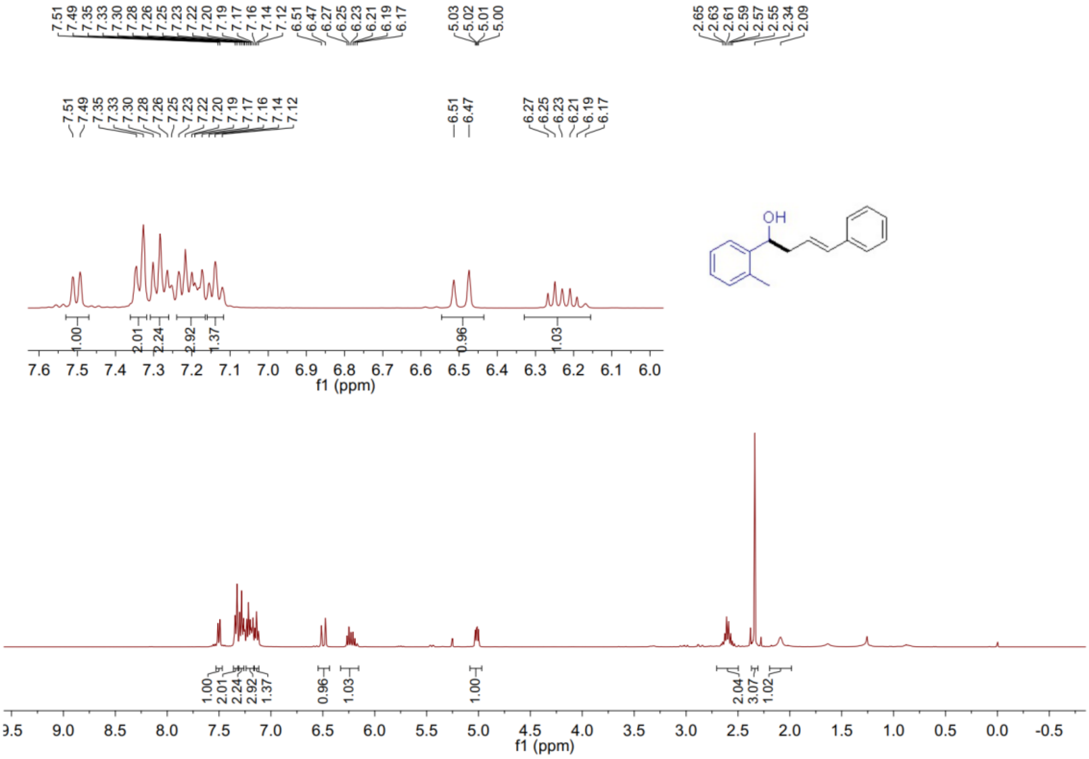

3x ^{13}C NMR

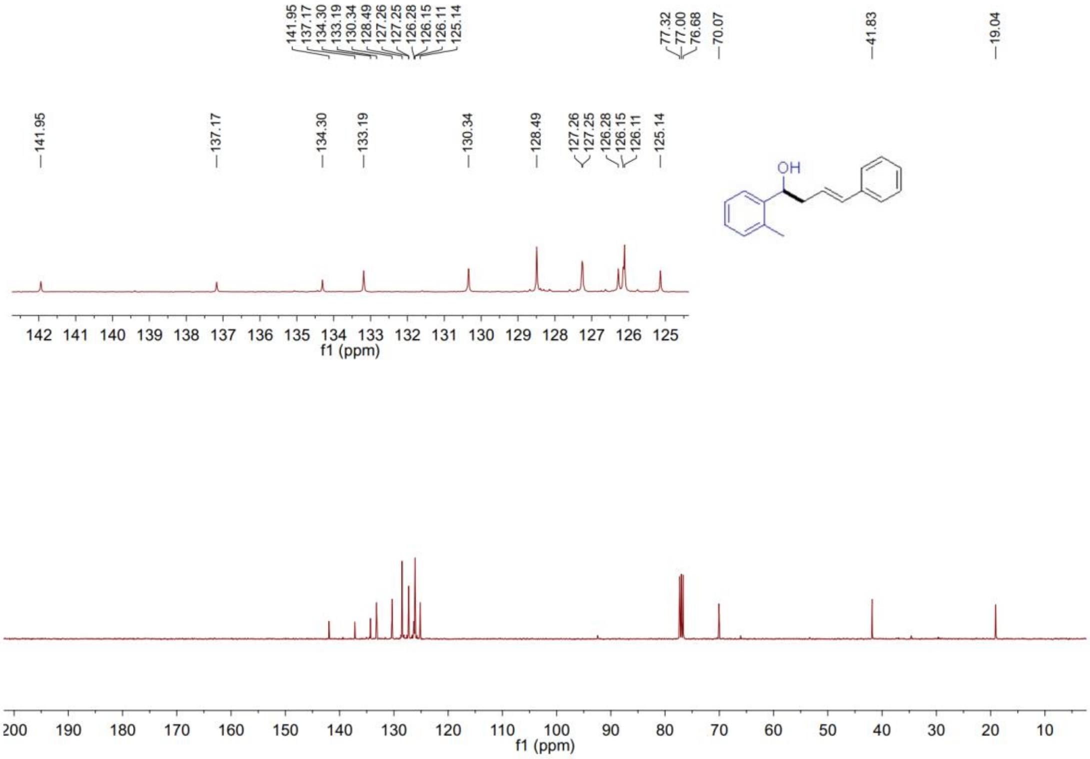

3y ^{1}H NMR

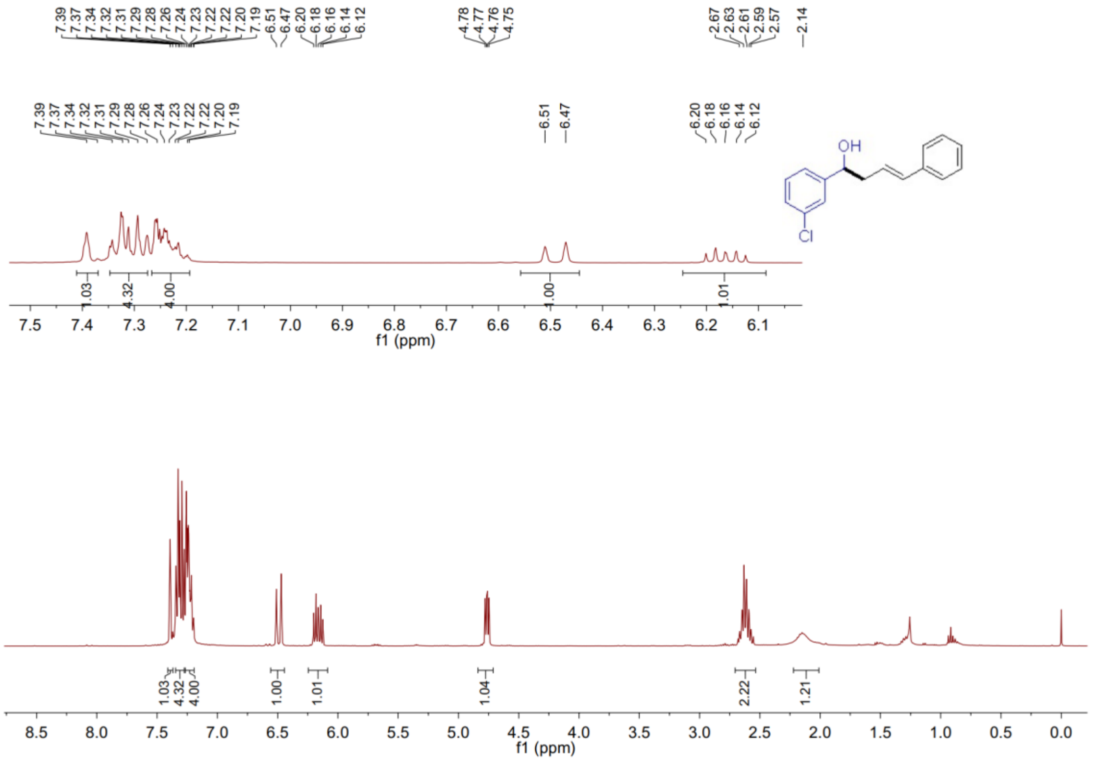

3y ^{13}C NMR

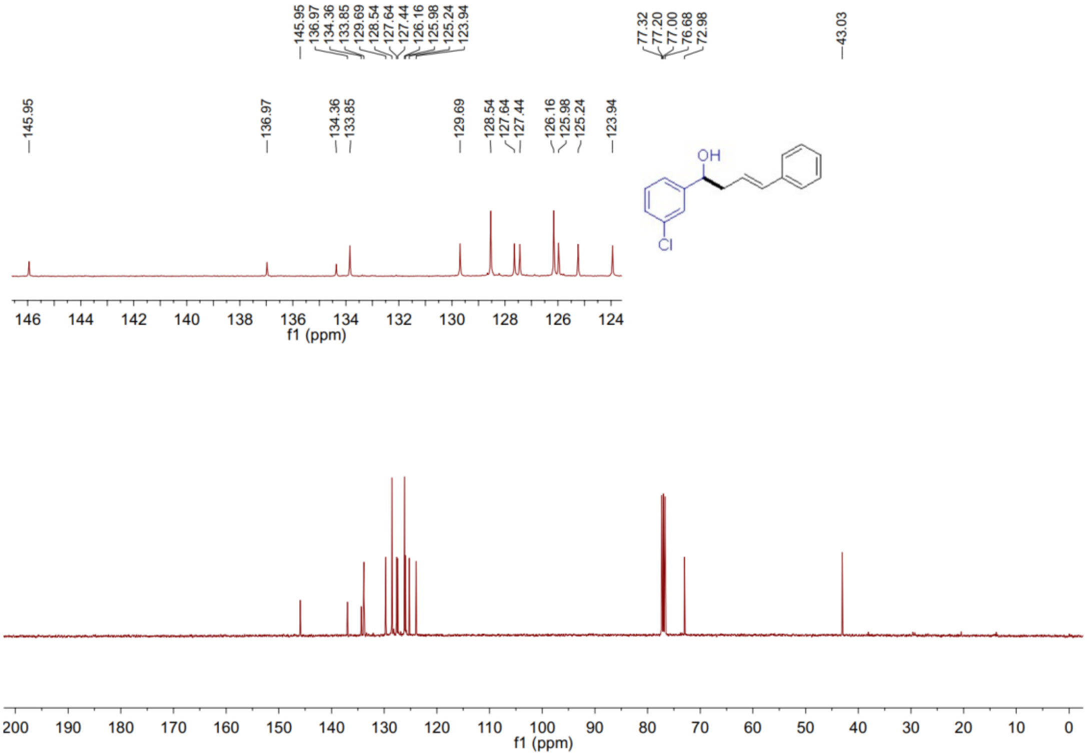

3z ^{1}H NMR

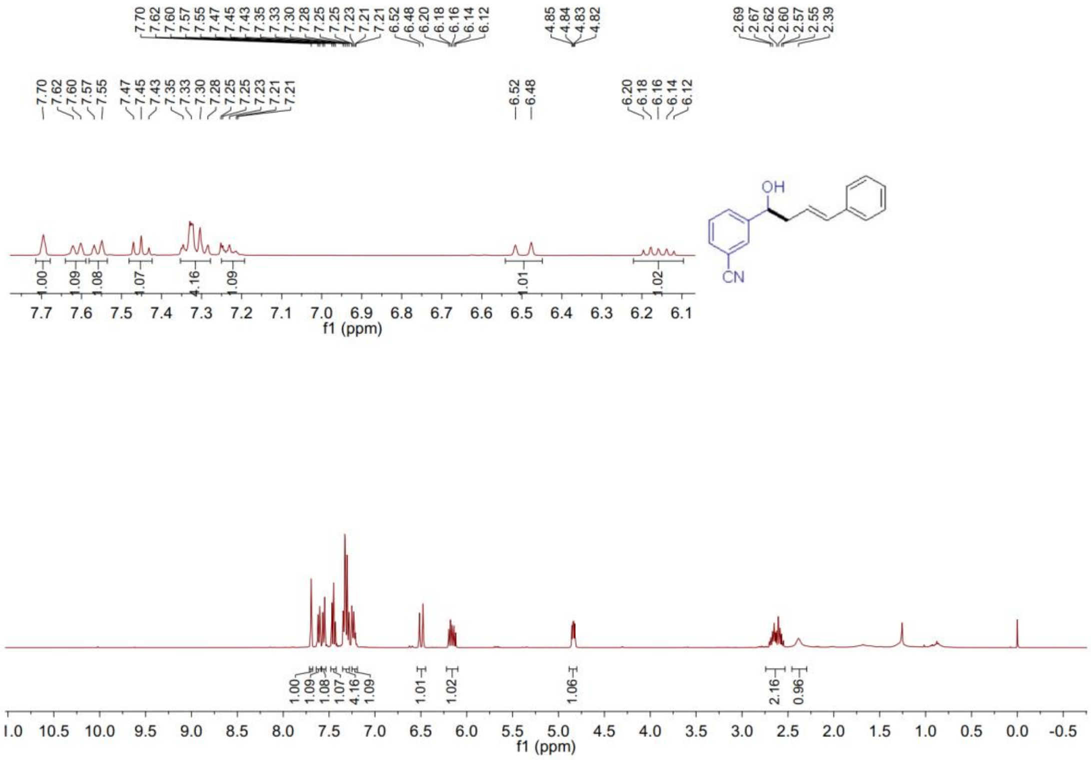

3z ^{13}C NMR

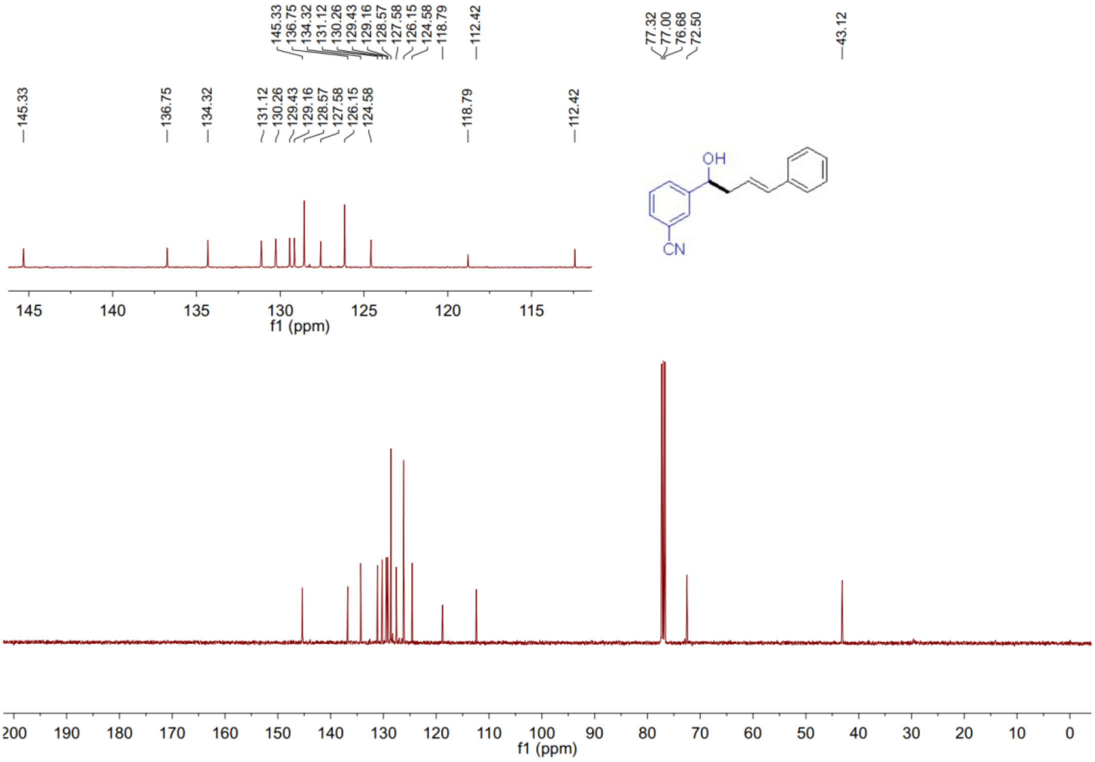

3aa ^{1}H NMR

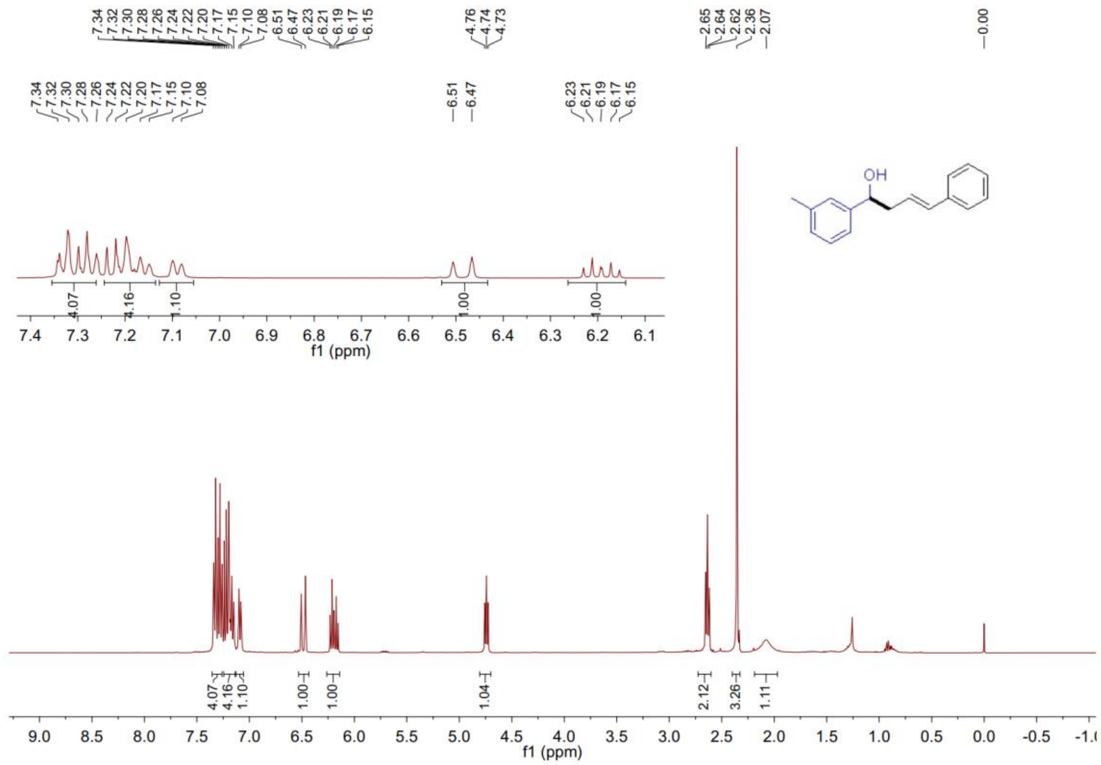

3aa ^{13}C NMR

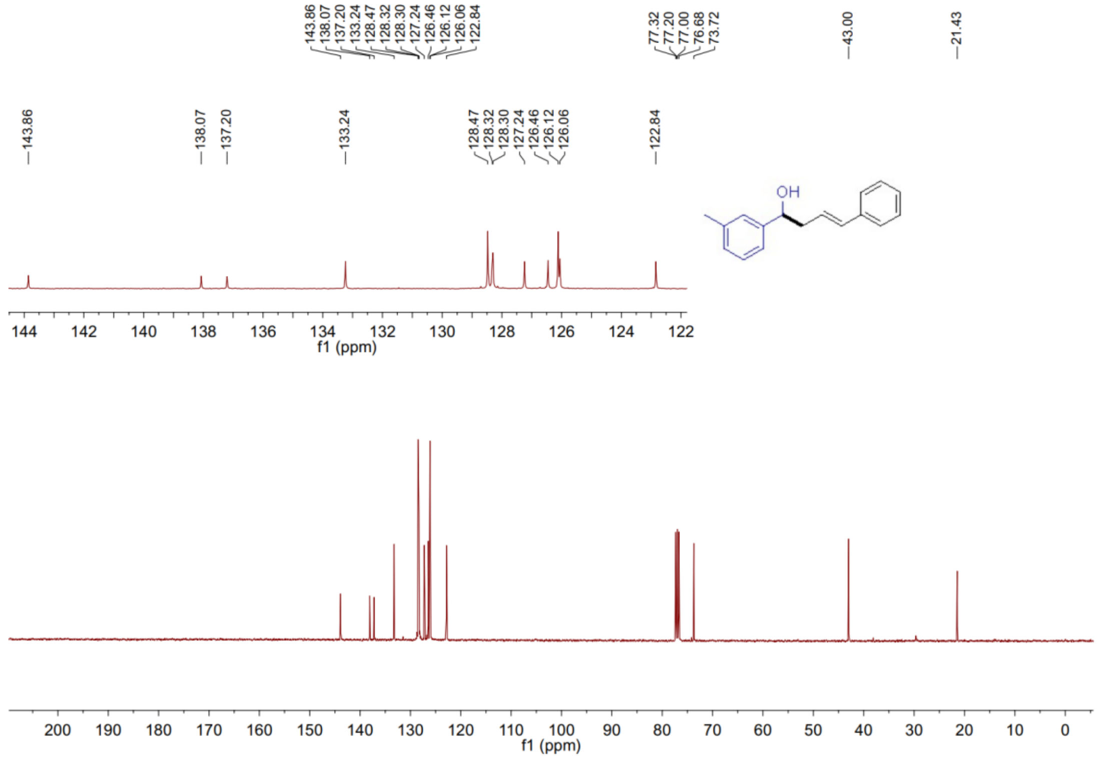

3ab ¹H NMR

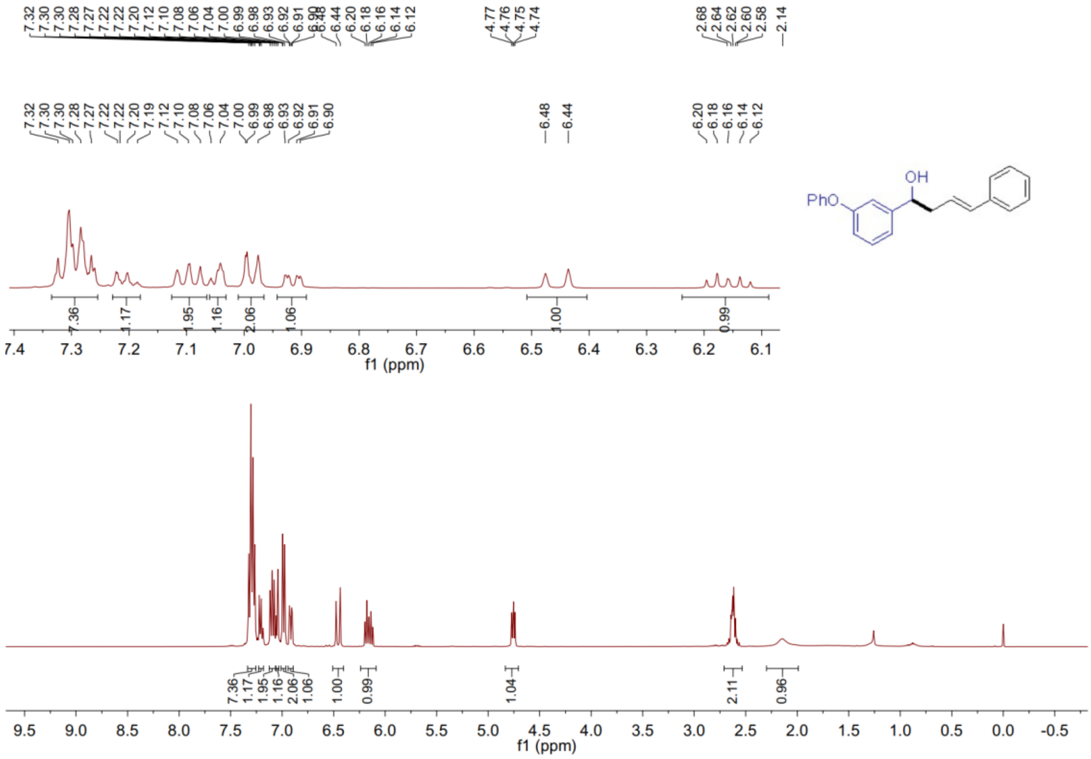

3ab ¹³C NMR

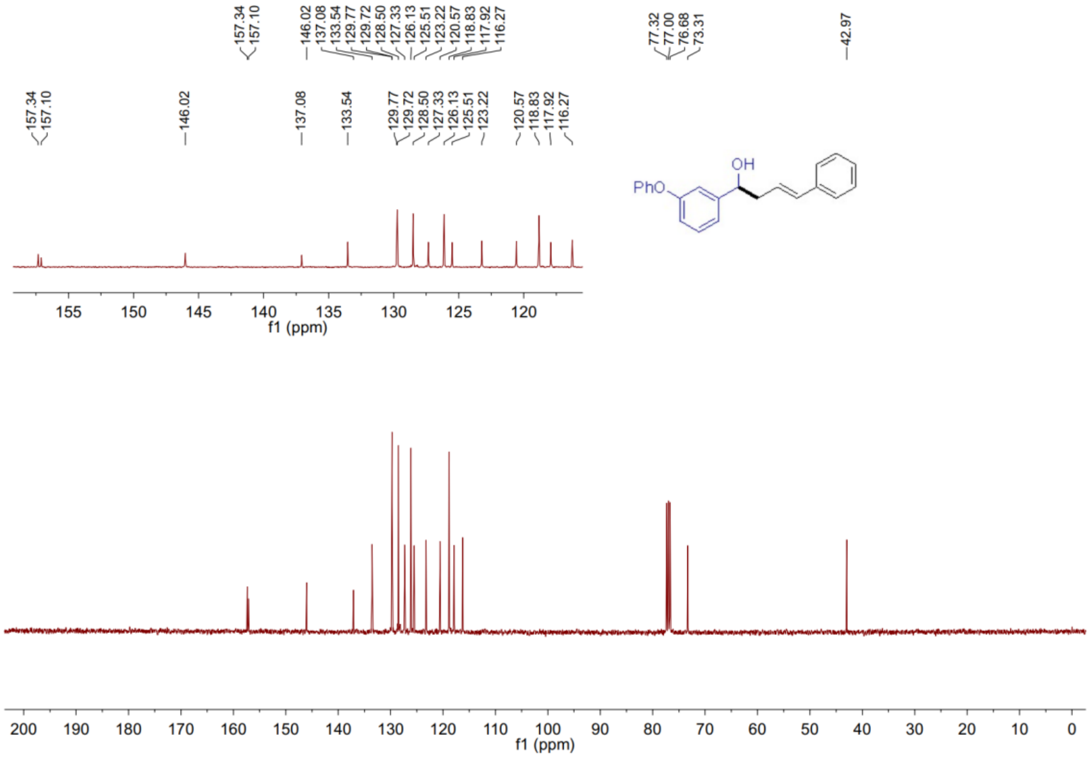

3ac ^{1}H NMR

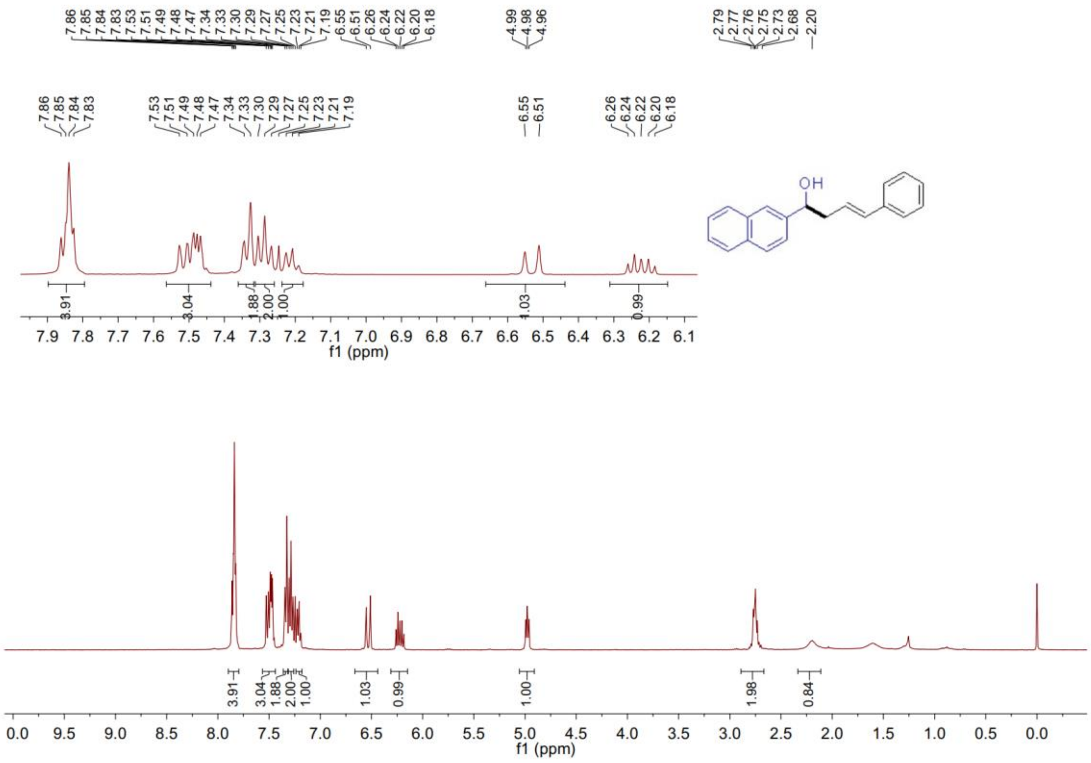

3ac ^{13}C NMR

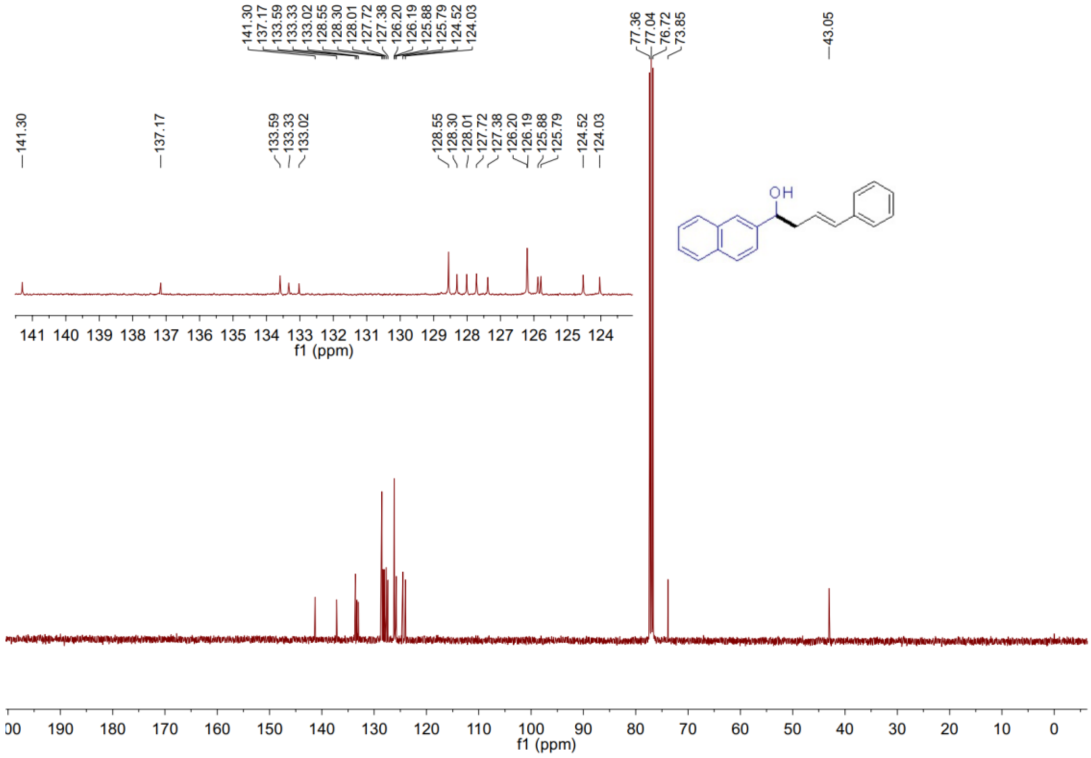

3ad ^{1}H NMR

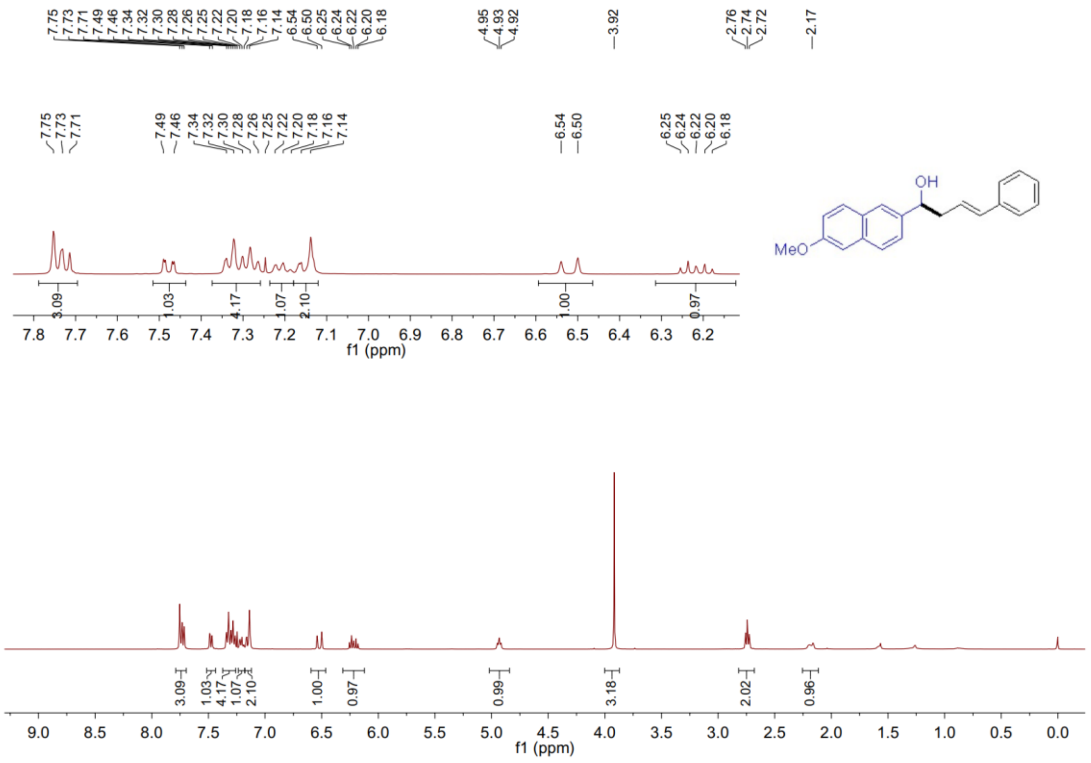

3ad ^{13}C NMR

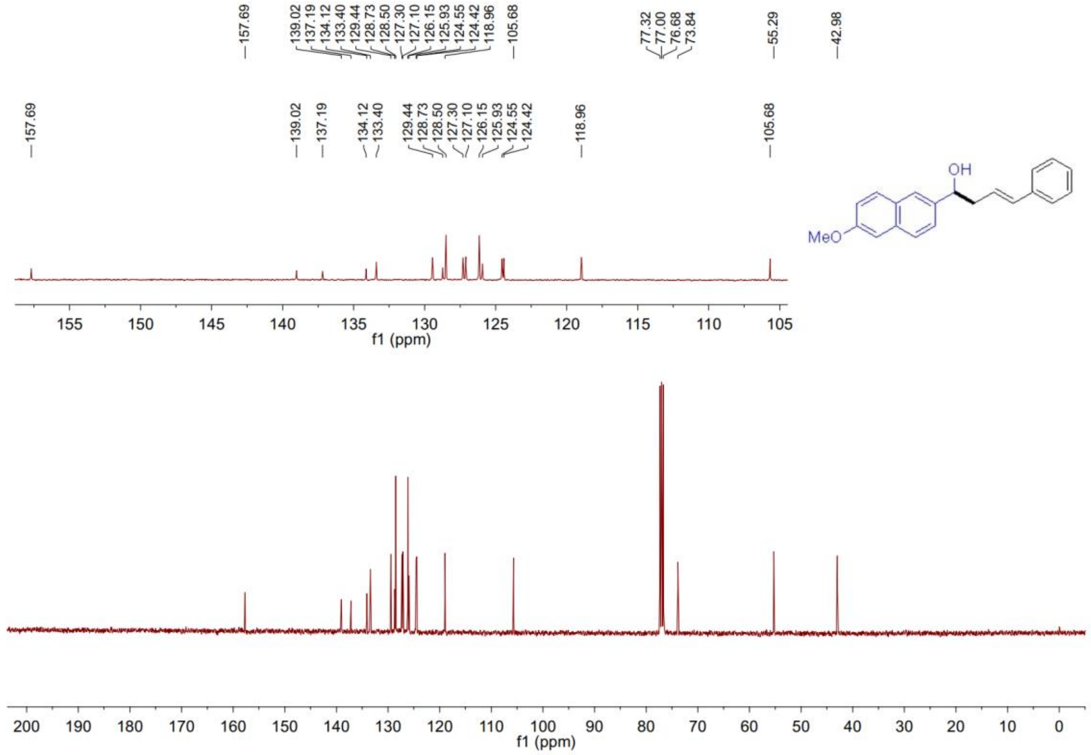

3ae ^{1}H NMR

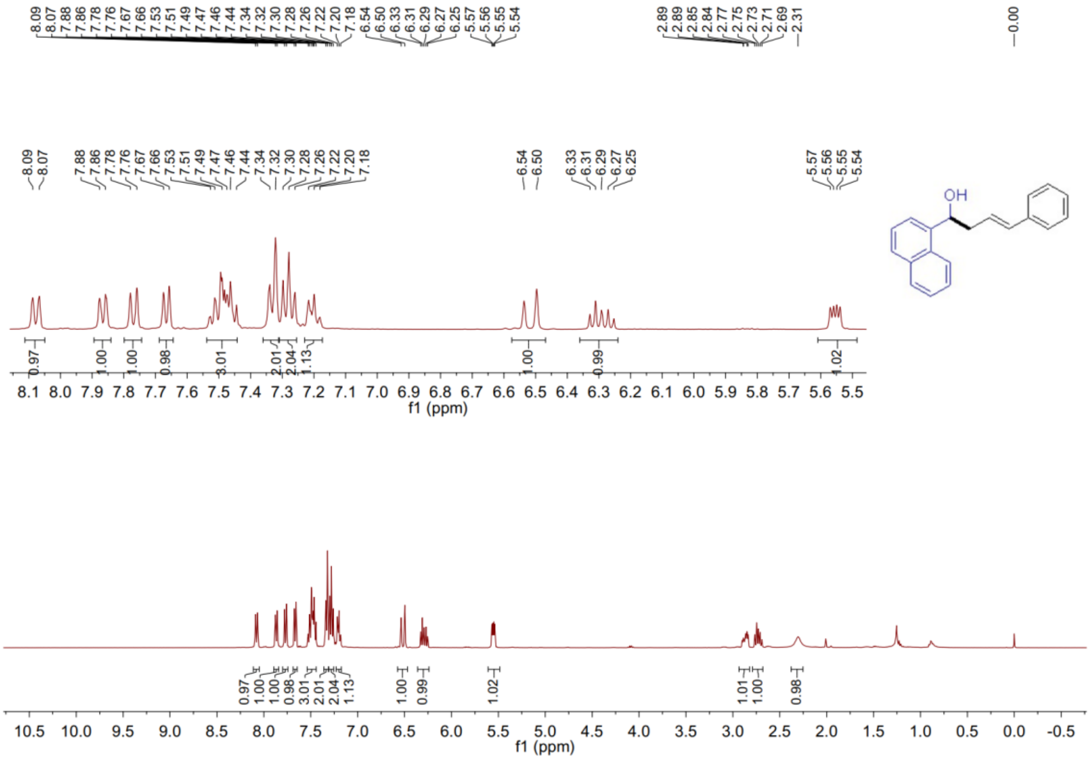

3ae ^{13}C NMR

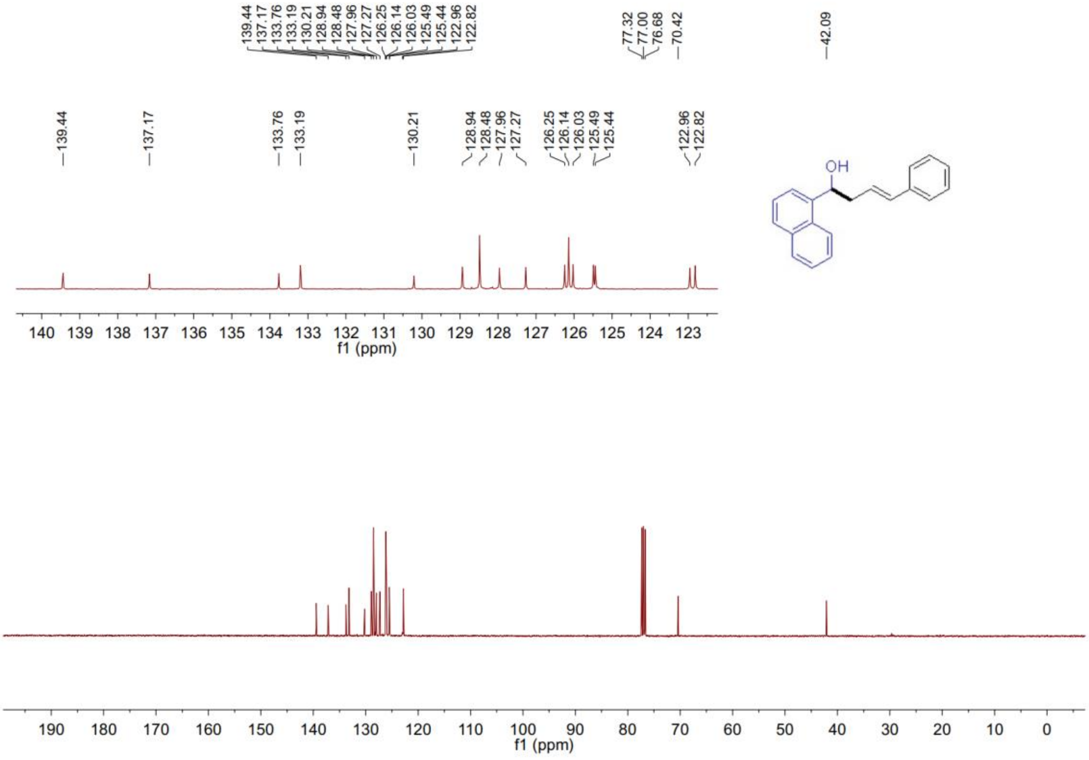

3af ^{1}H NMR

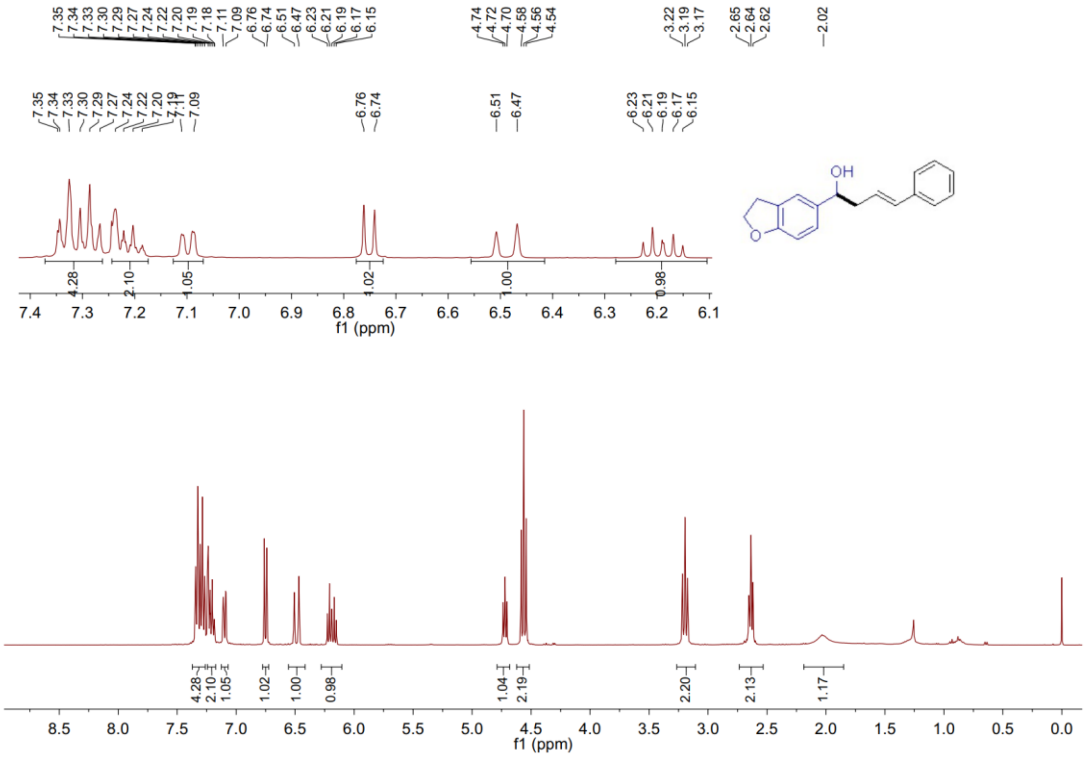

3af ^{13}C NMR

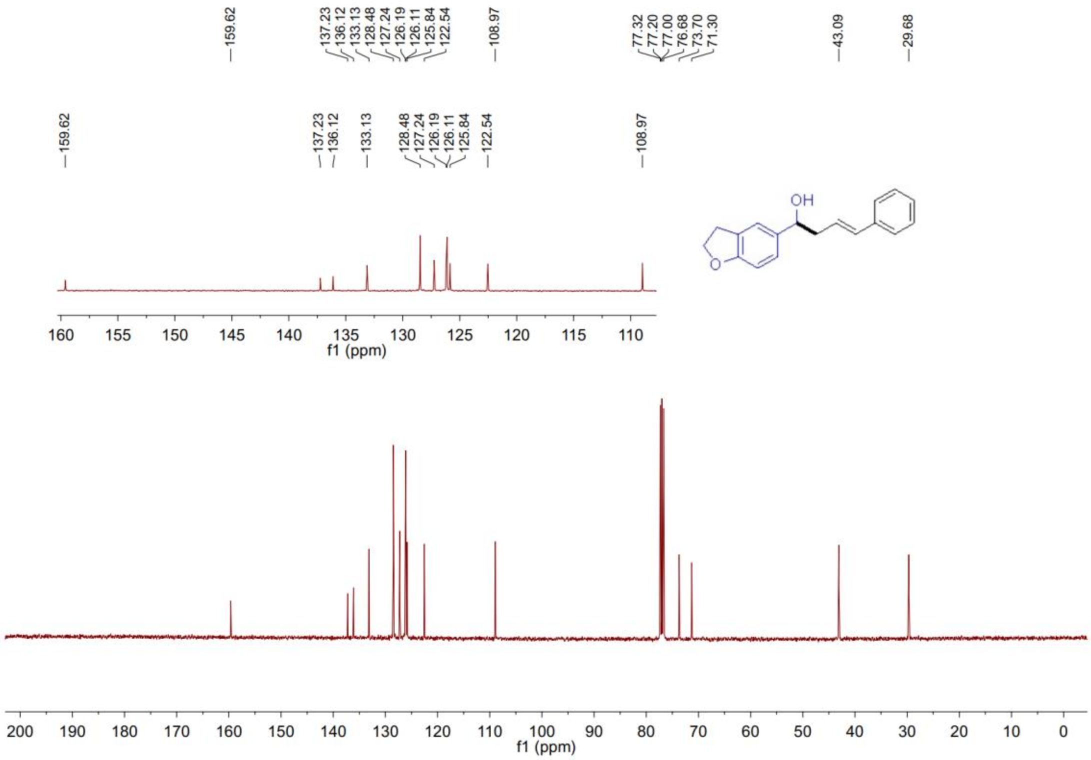

3ag ^{1}H NMR

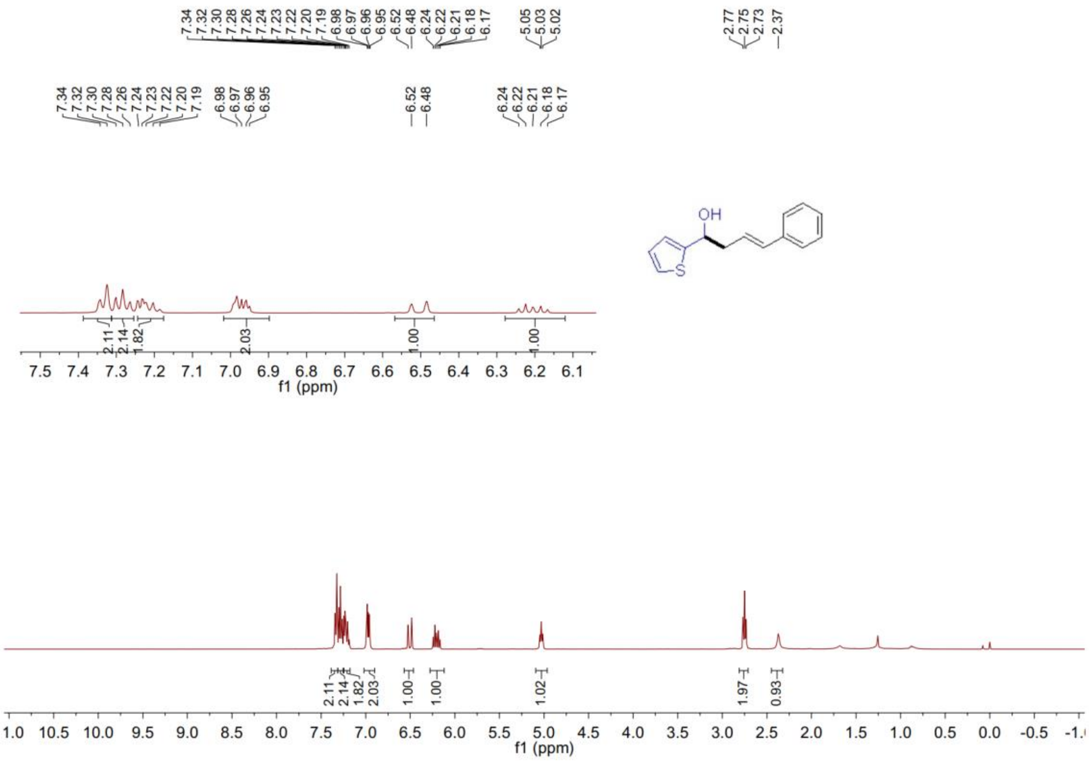

3ag ^{13}C NMR

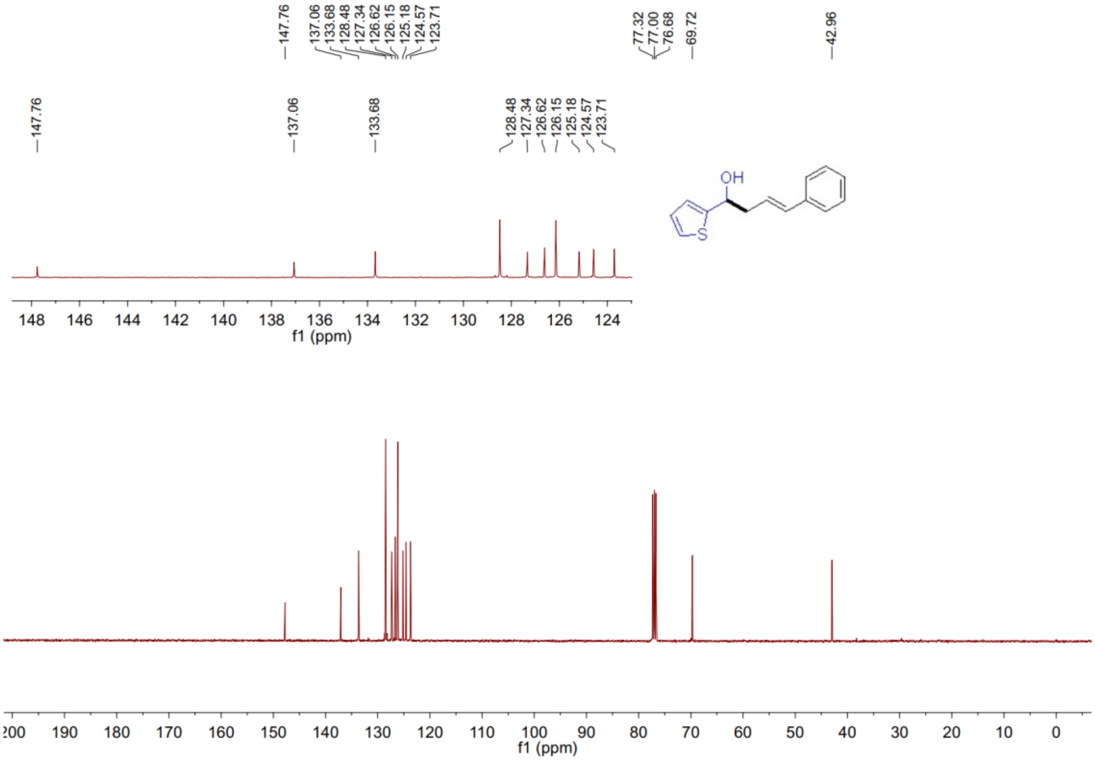

3ah ^{1}H NMR

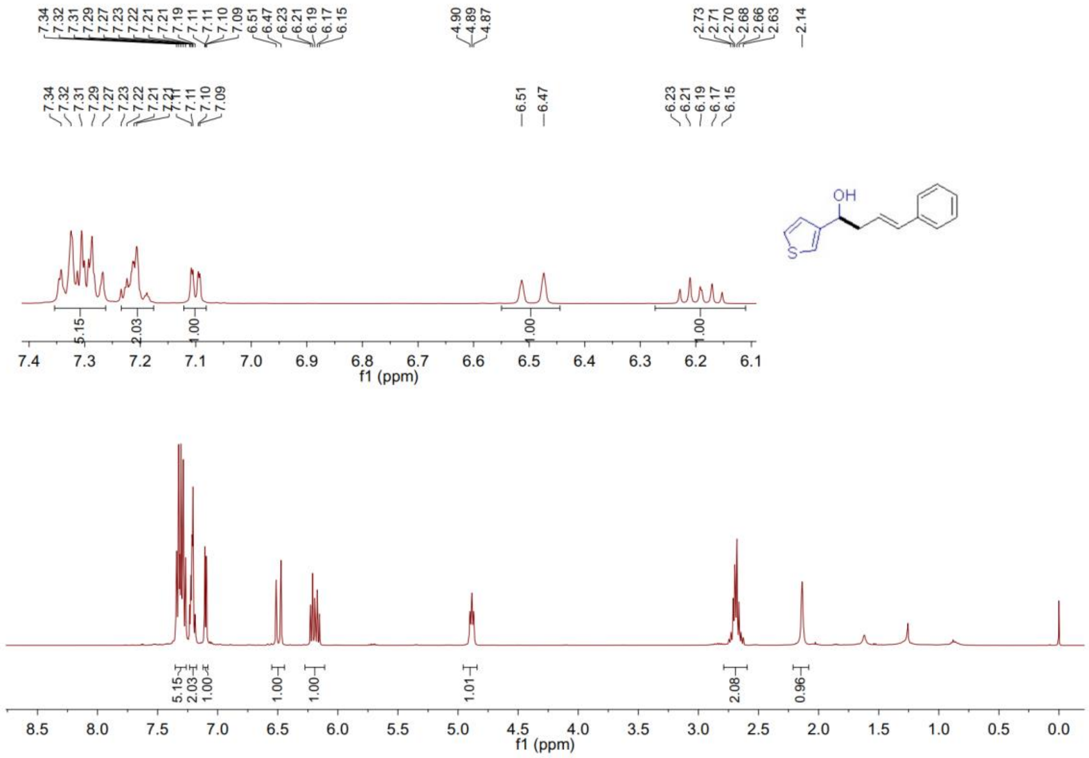

3ah ^{13}C NMR

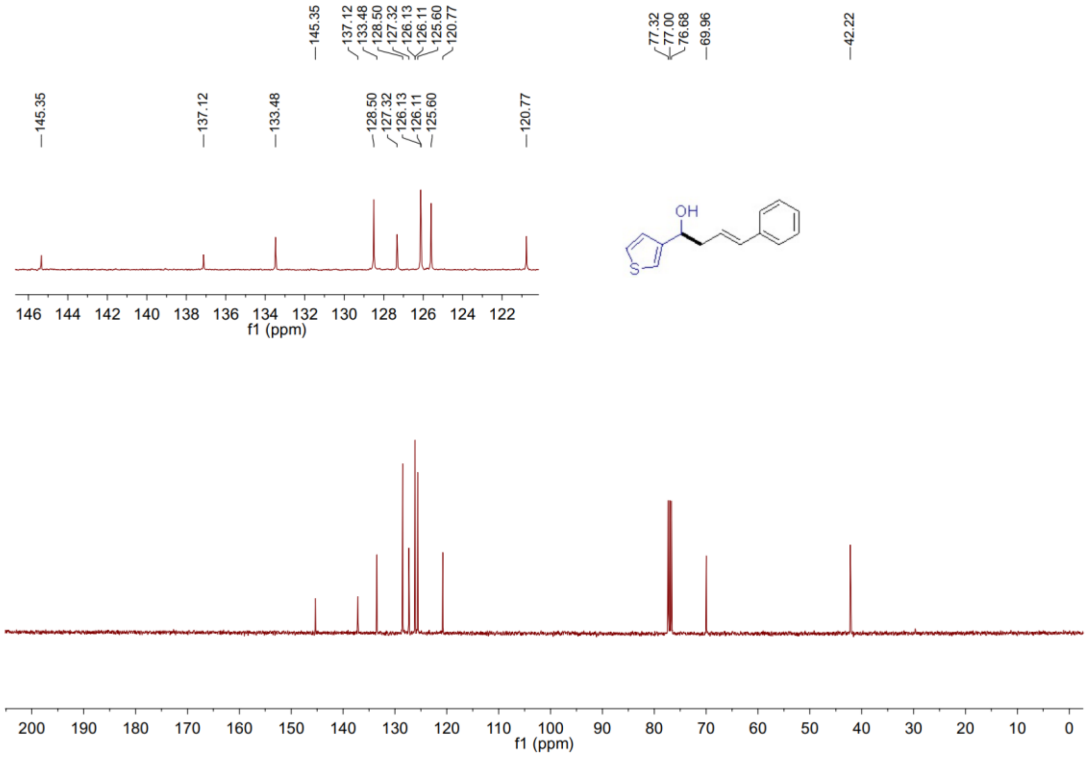

3ai ^{1}H NMR

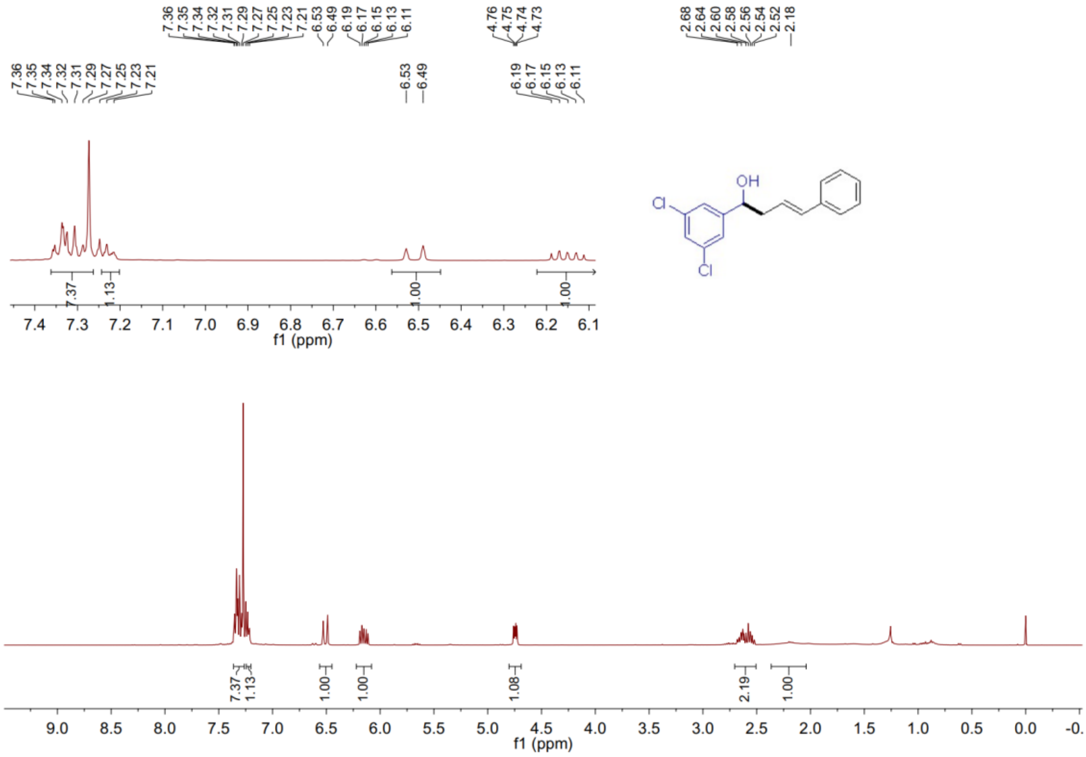

3ai ^{13}C NMR

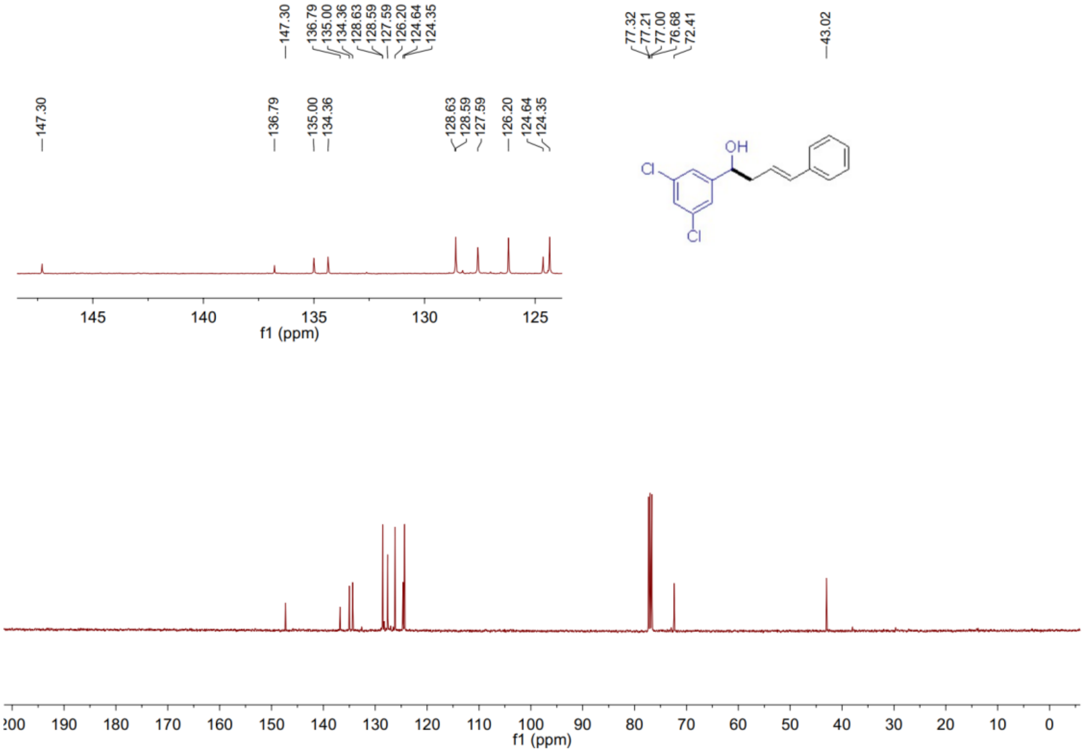

3aj ^{1}H NMR

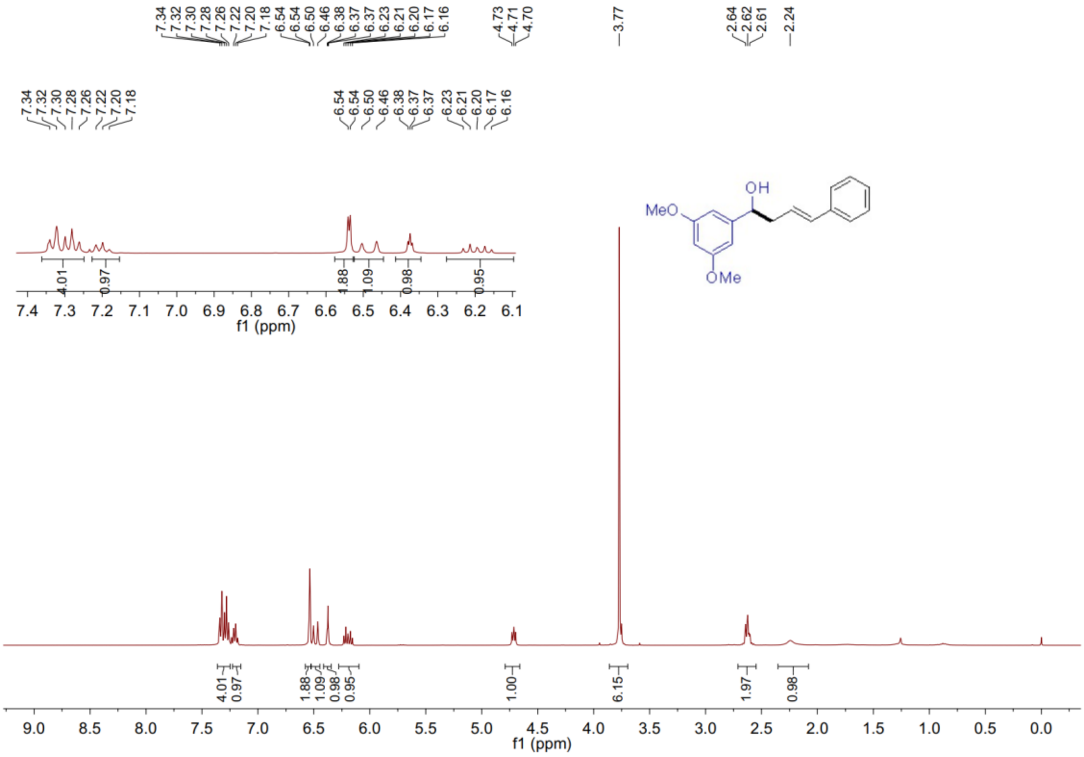

3aj ^{13}C NMR

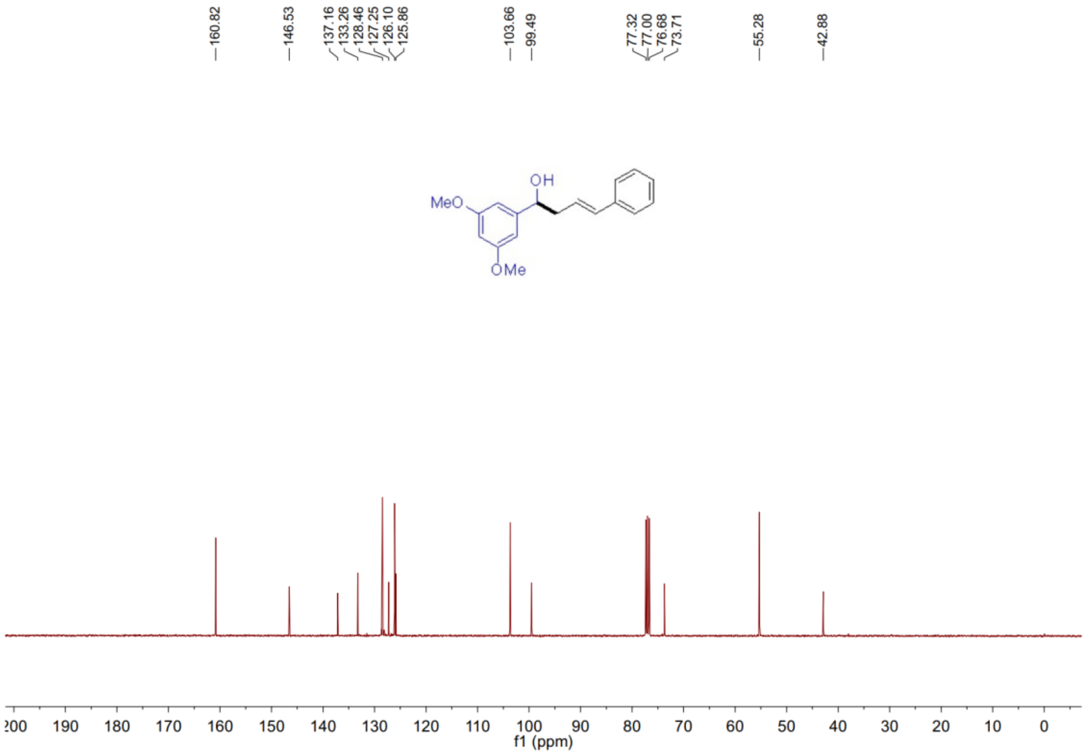

3ak ¹H NMR

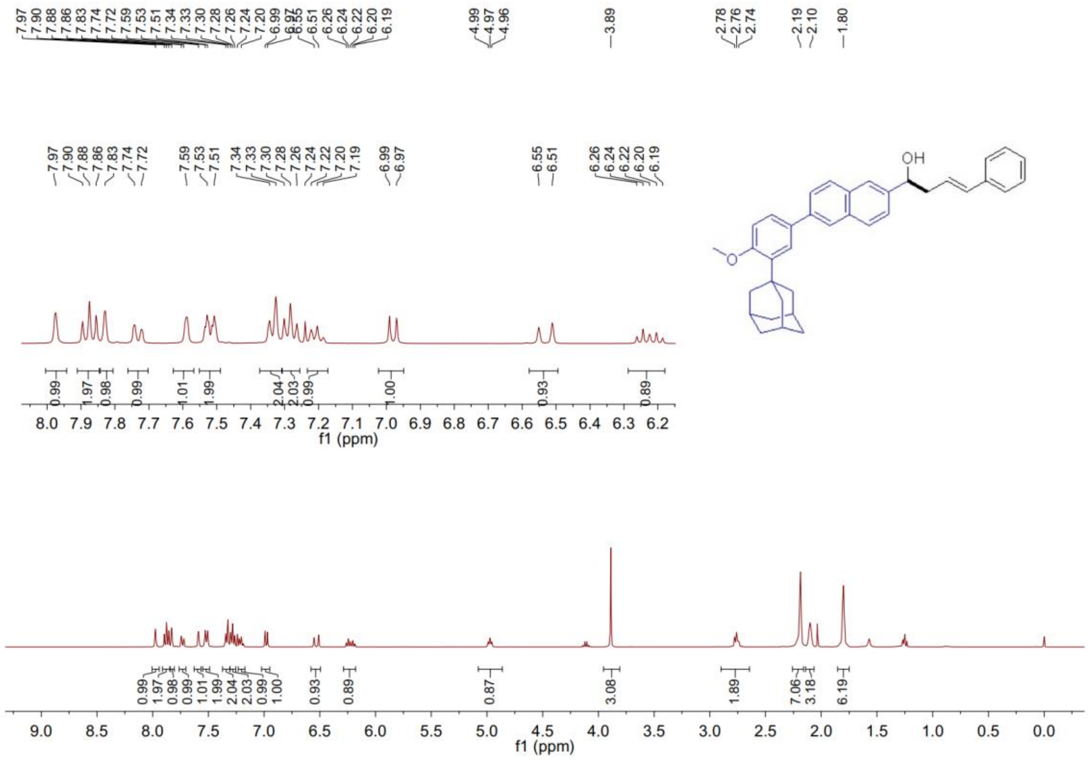

3ak ¹³C NMR

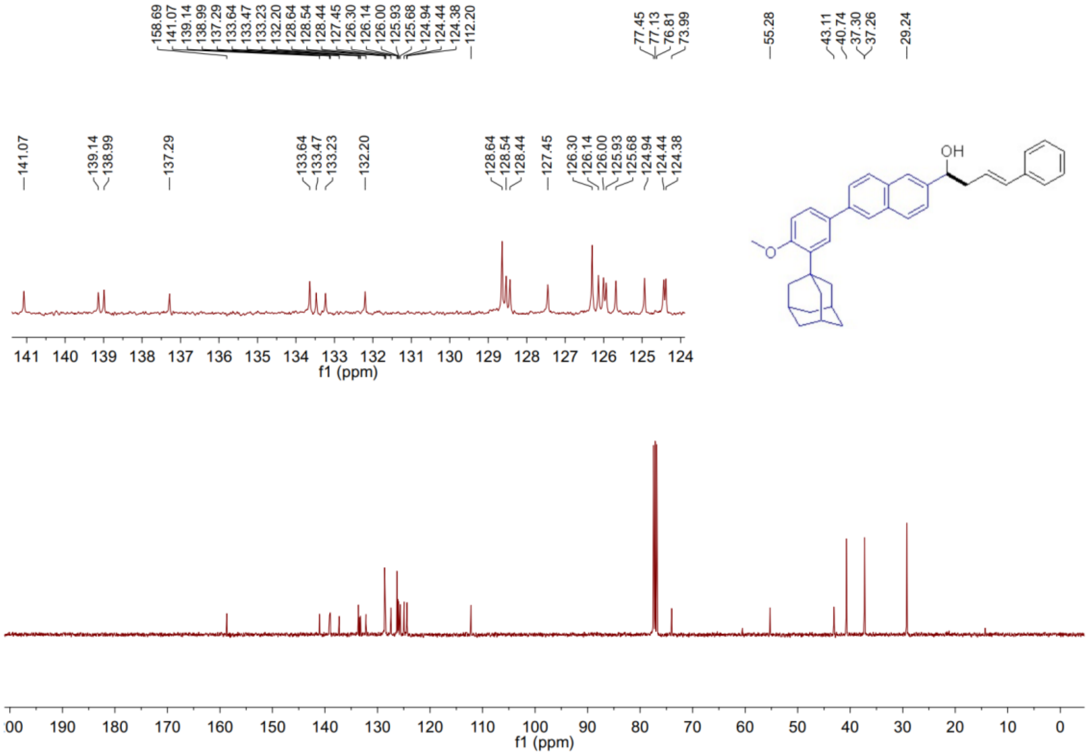

3al ^{1}H NMR

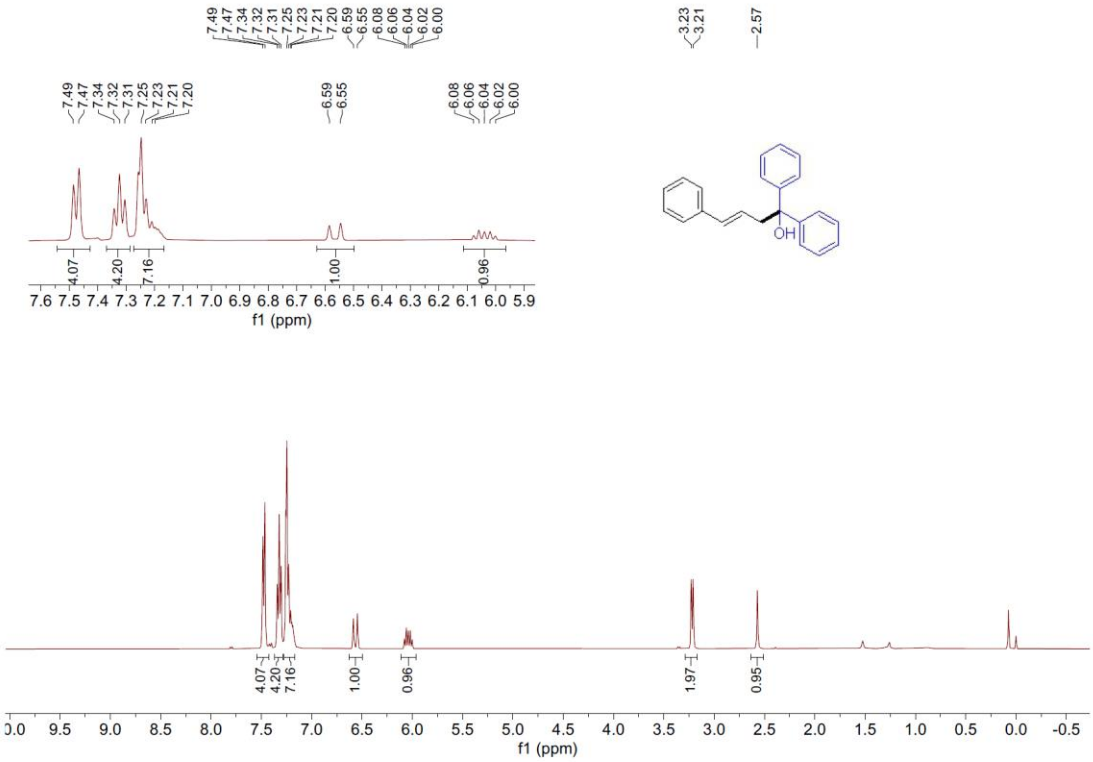

3al ^{13}C NMR

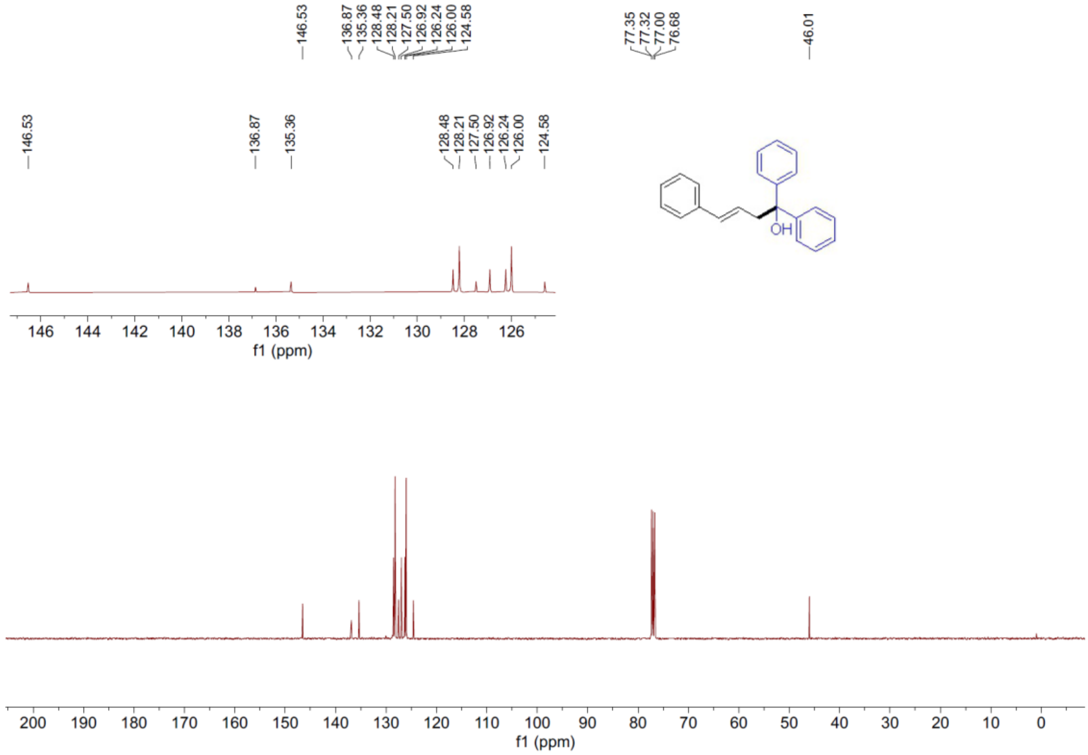

3am ^{1}H NMR

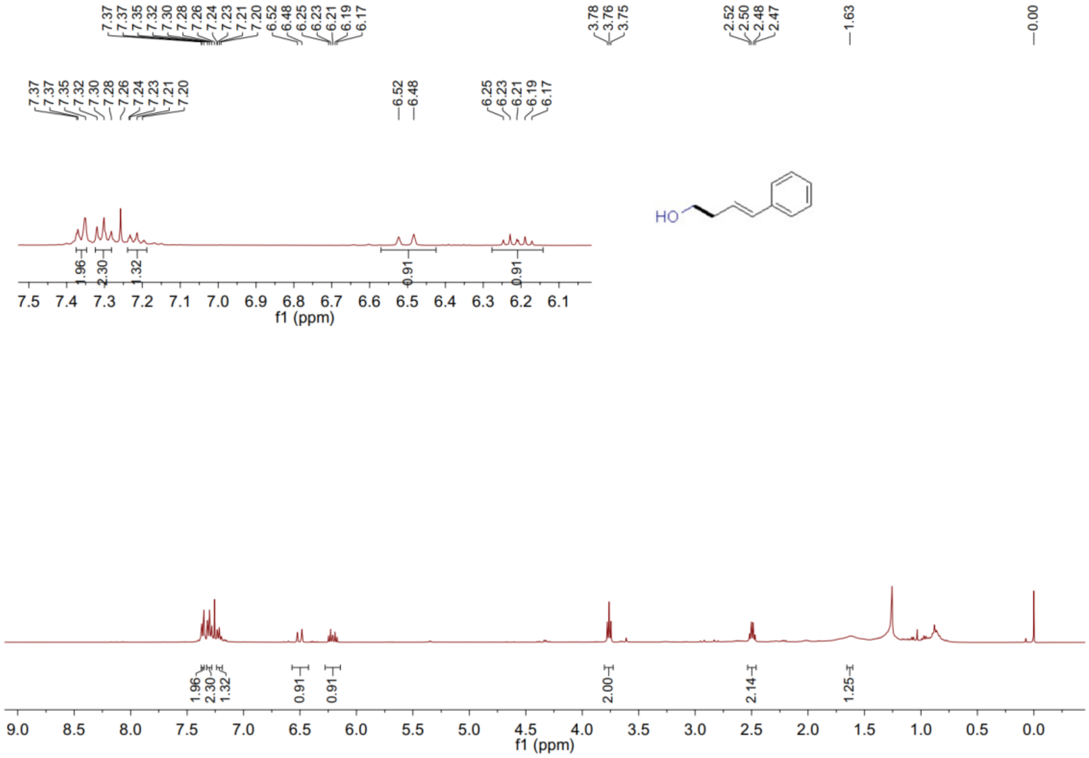

3am ^{13}C NMR

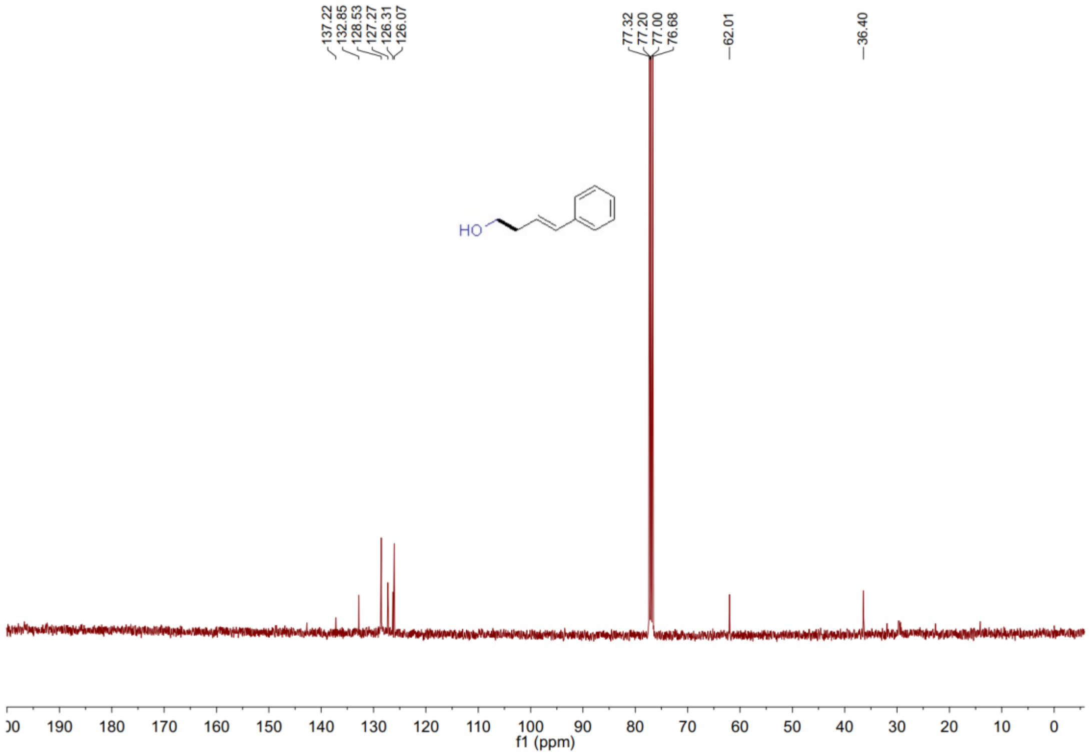

3an ^{1}H NMR

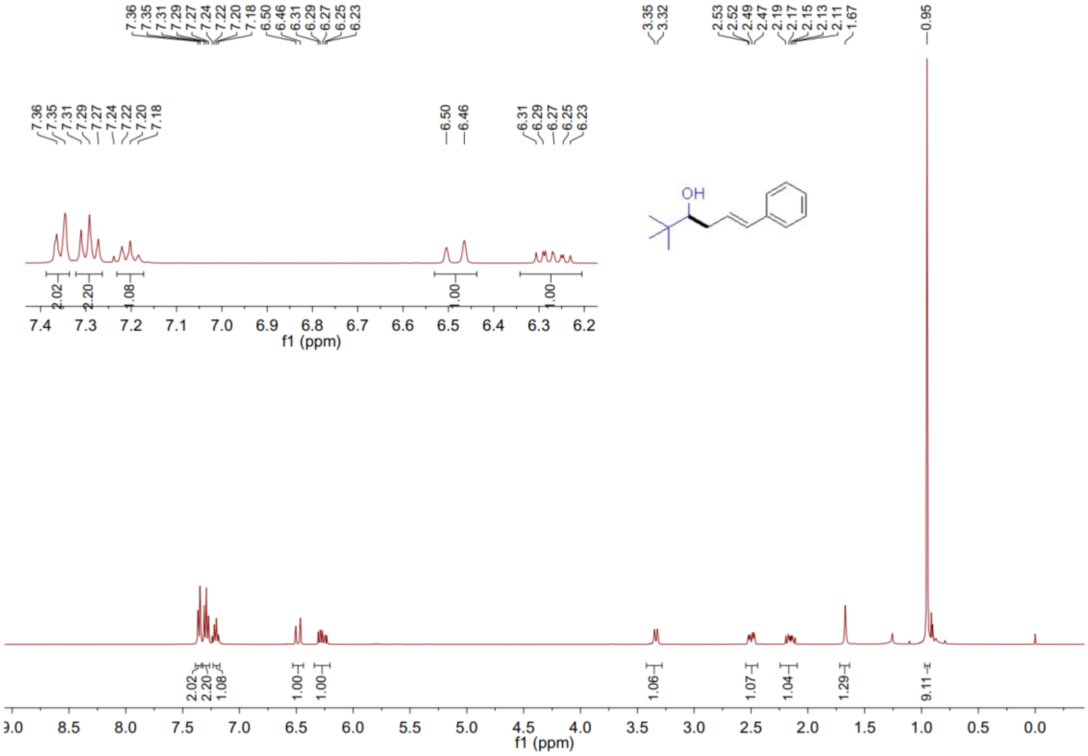

3an ^{13}C NMR

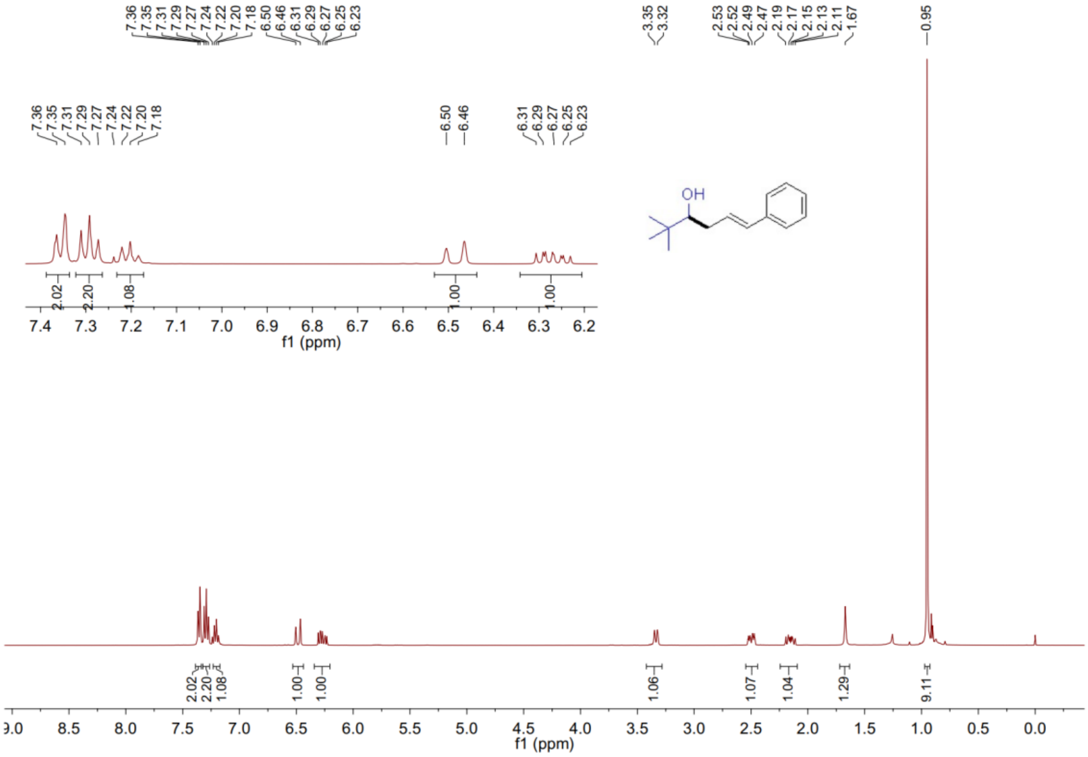

3ao ^{1}H NMR

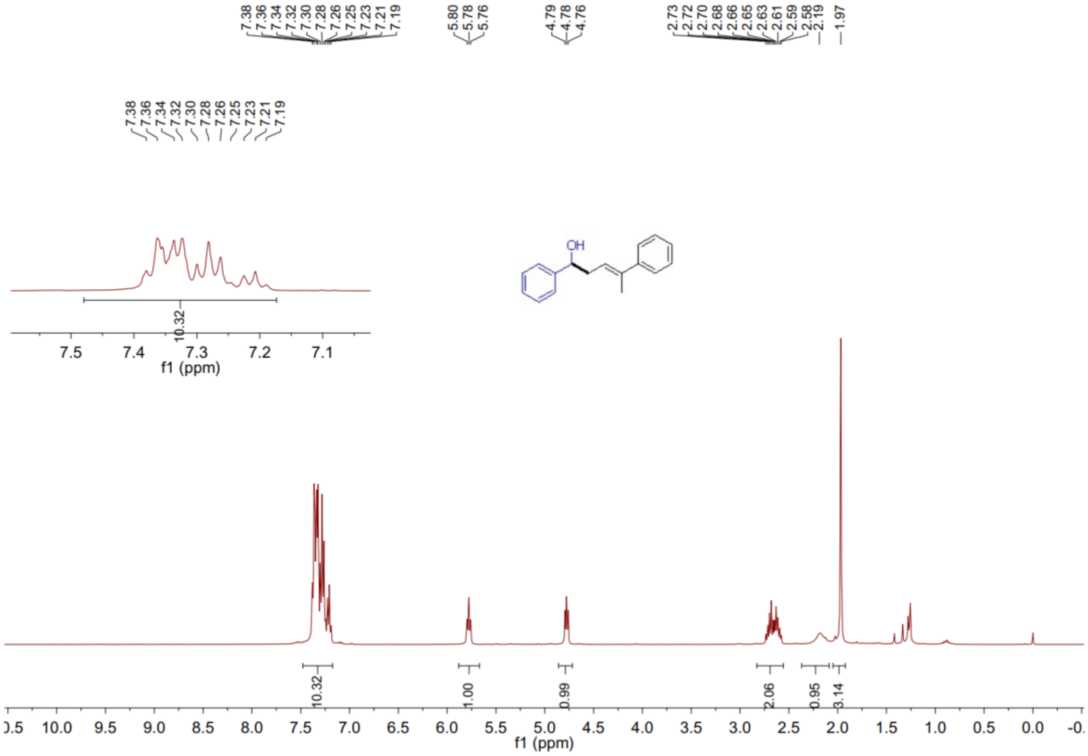

3ao ^{13}C NMR

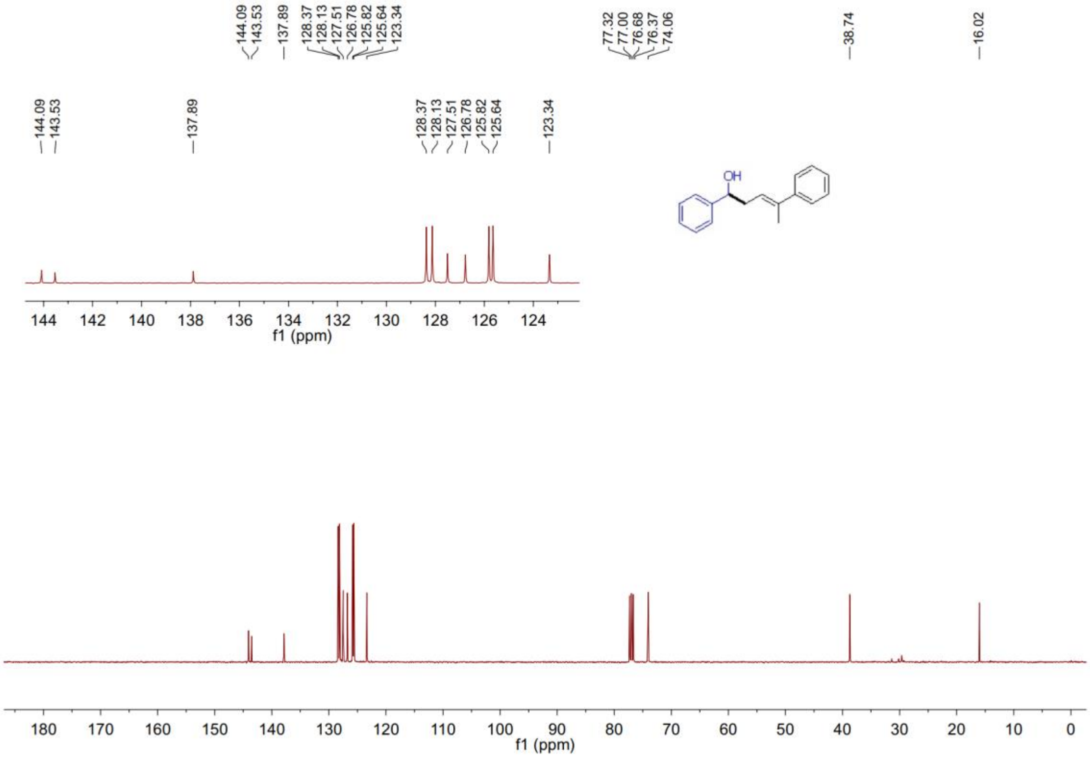

3ap ^{1}H NMR

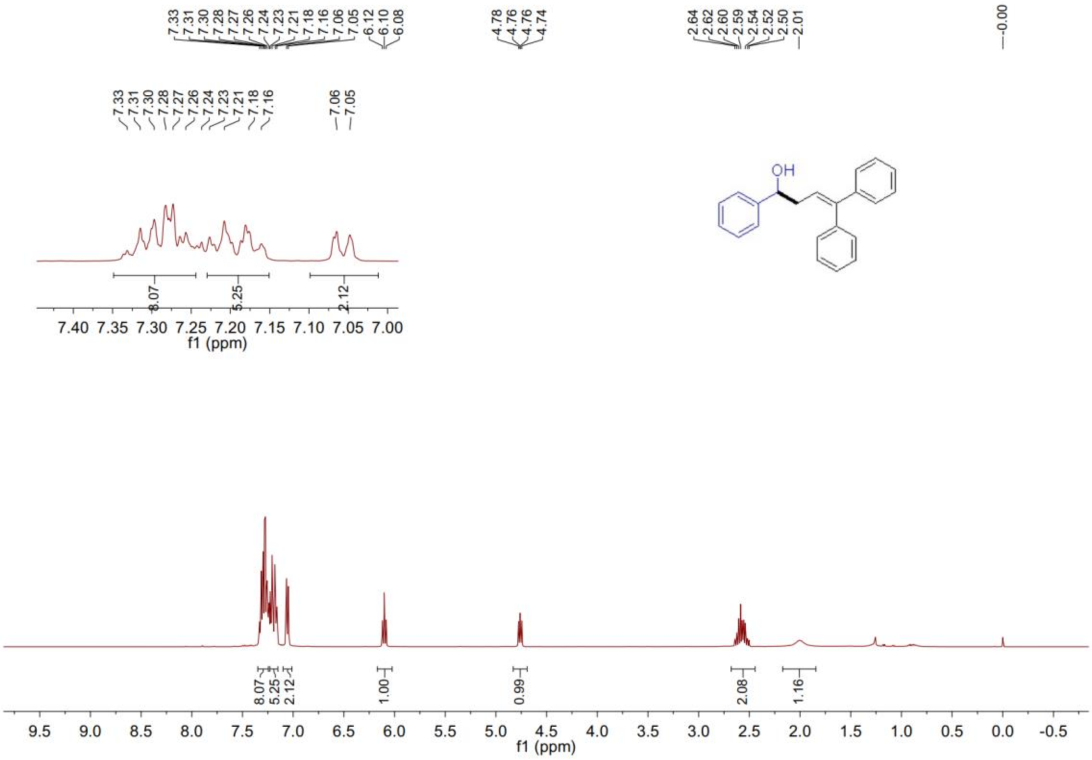

3ap ^{13}C NMR

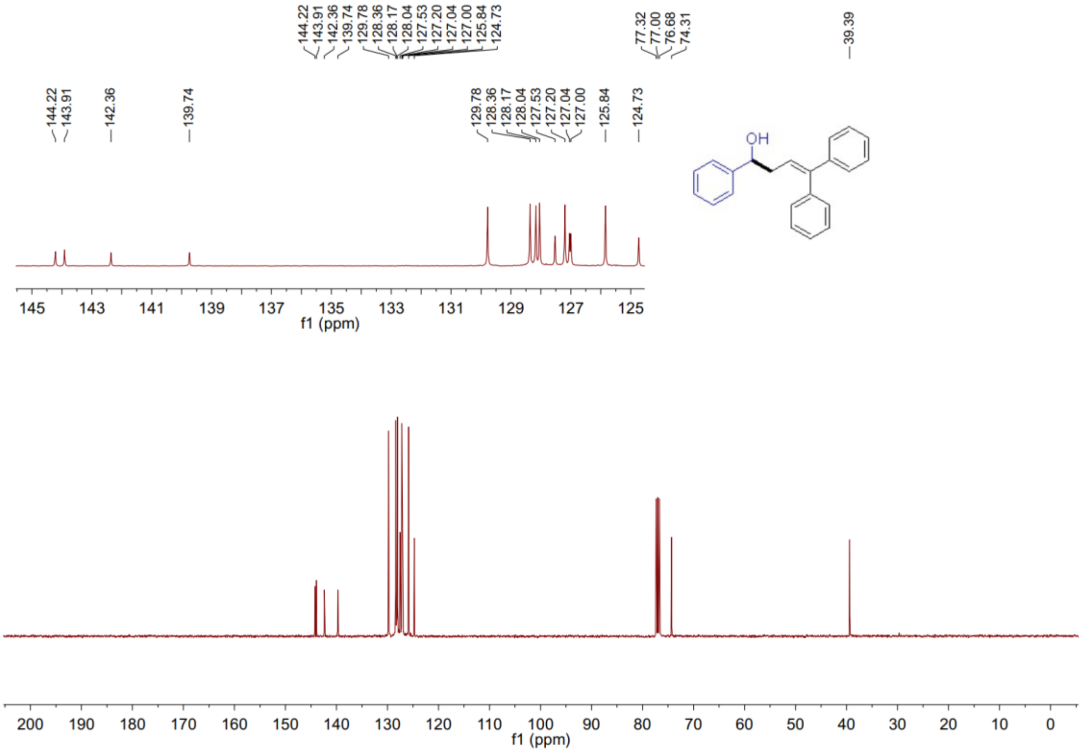

3aq ^{1}H NMR

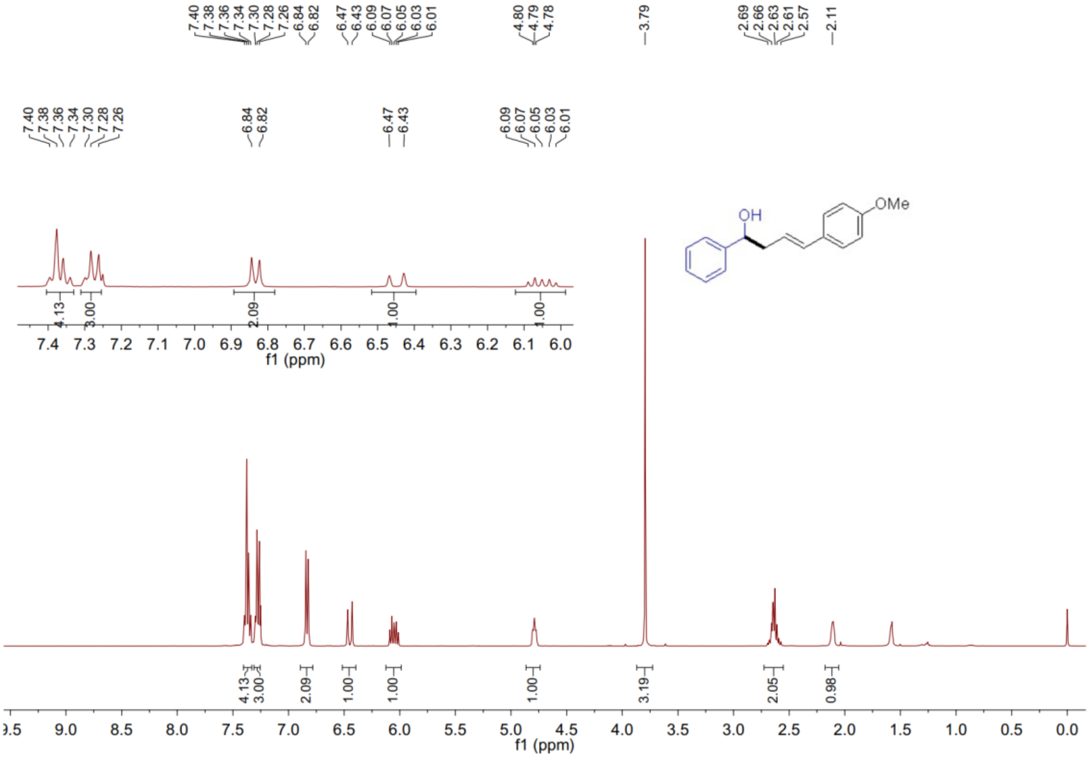

3aq ^{13}C NMR

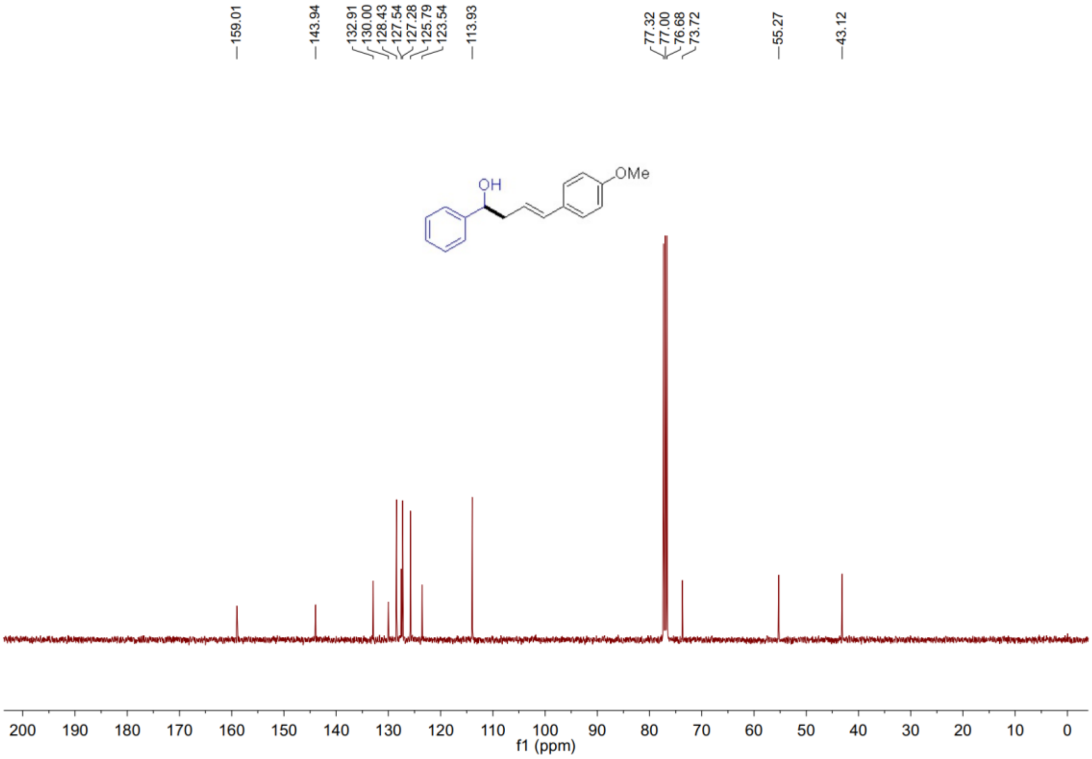

3ar ^{1}H NMR

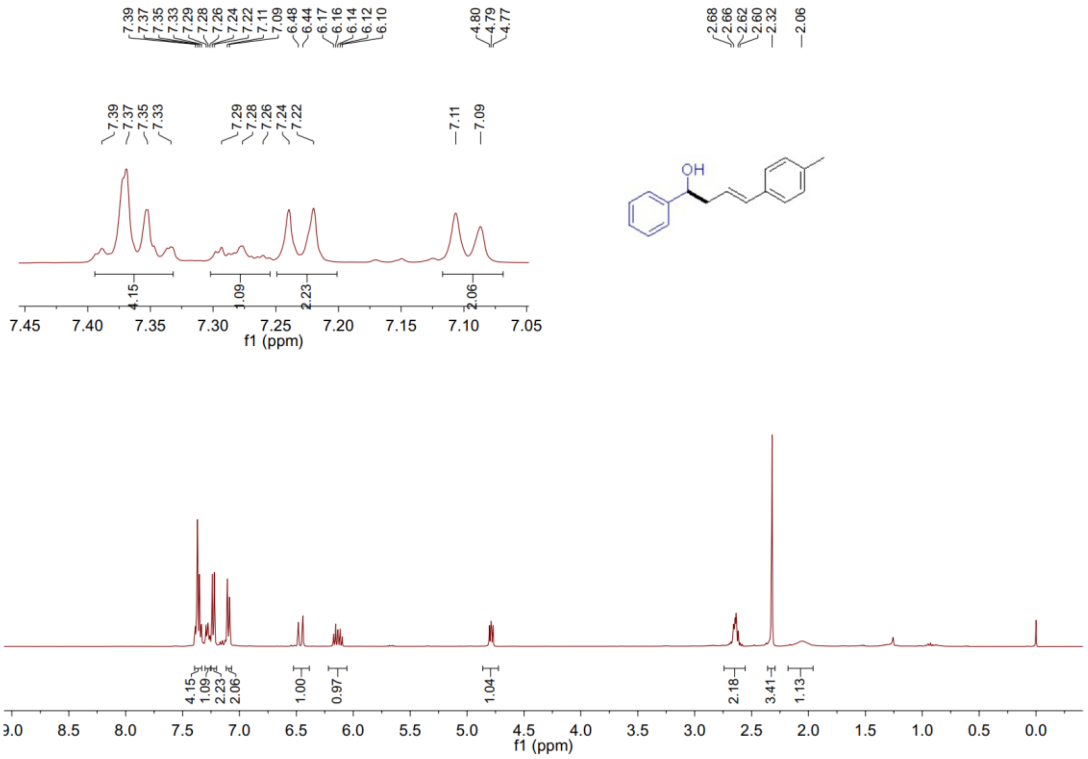

3ar ^{13}C NMR

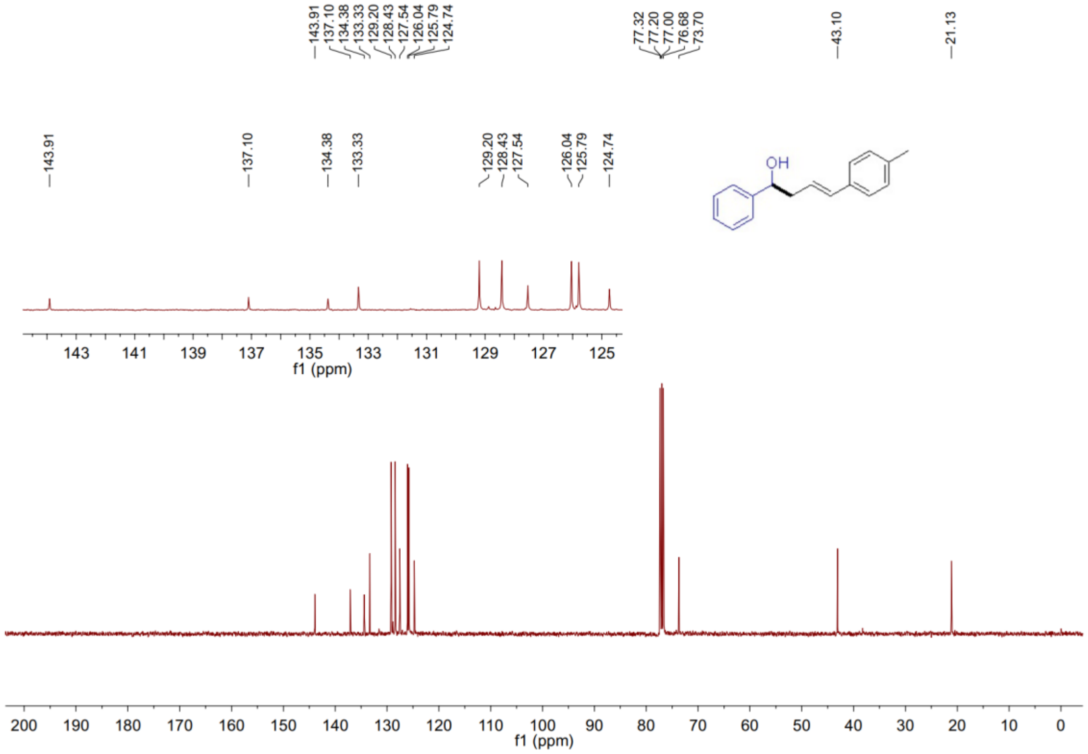

3as ^{1}H NMR

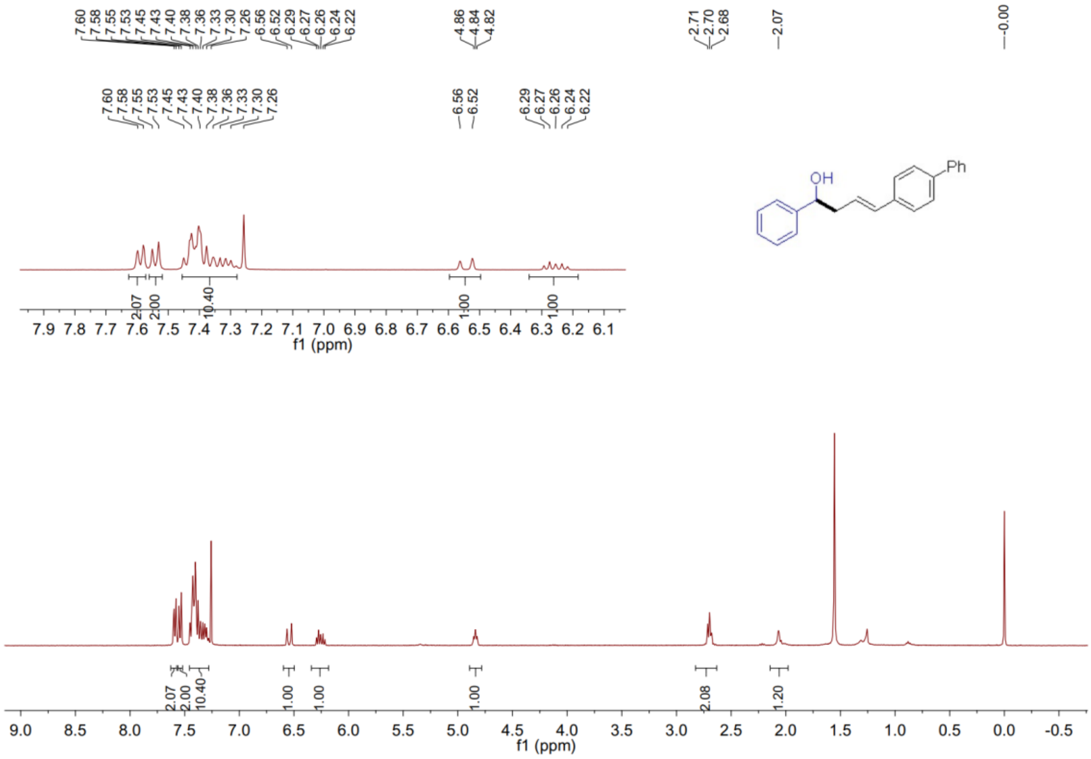

3as ^{13}C NMR

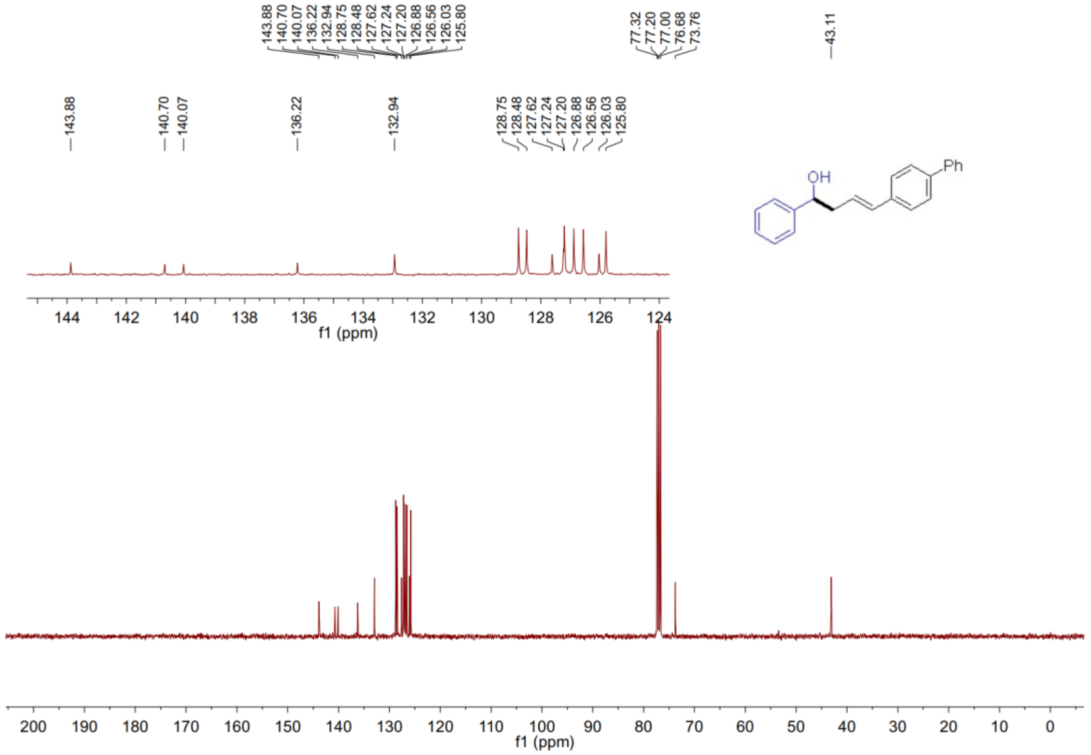

3at ^{1}H NMR

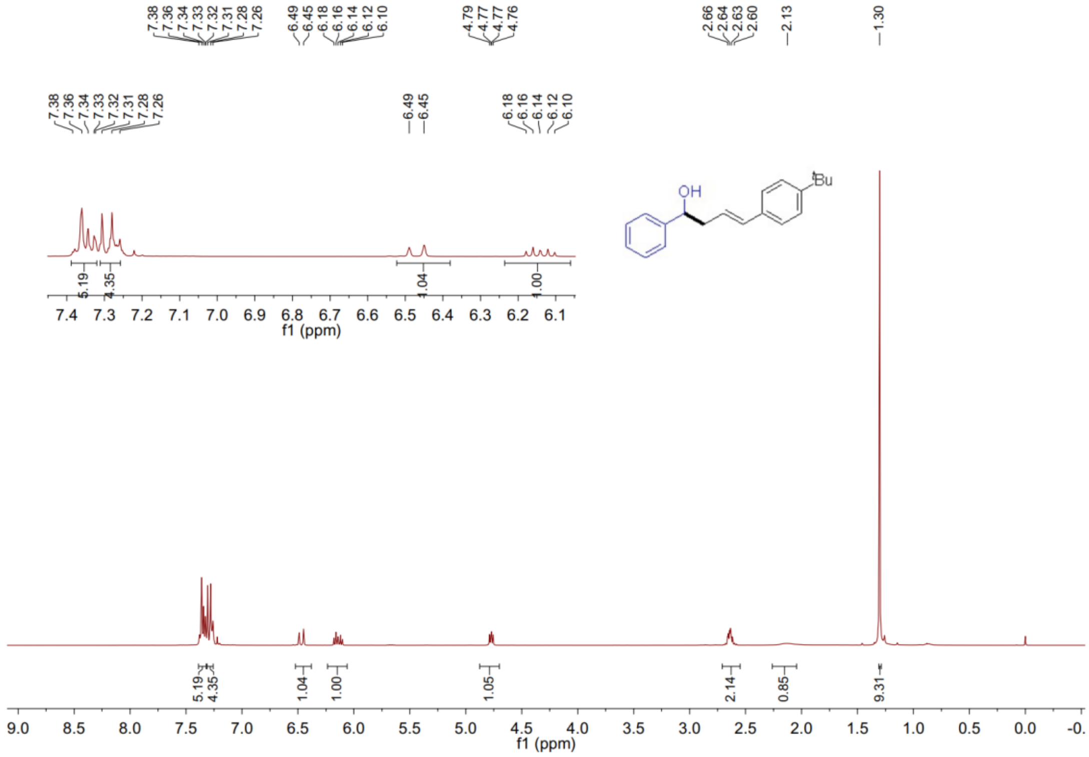

3at ^{13}C NMR

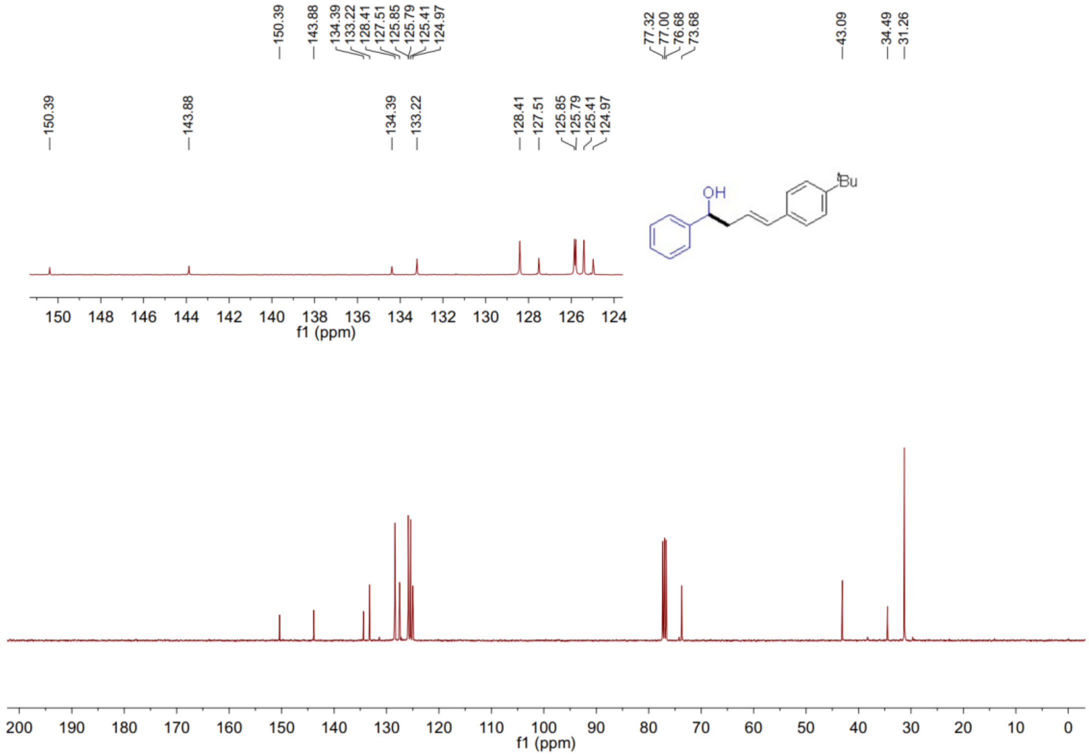

3au ^{1}H NMR

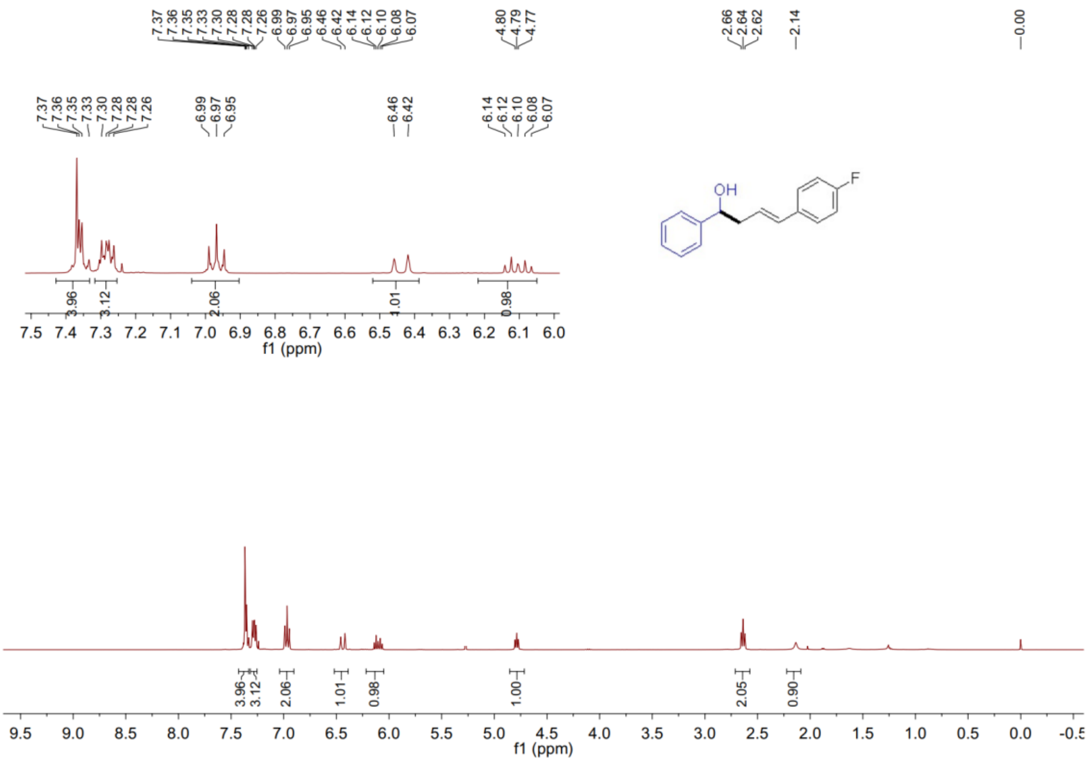

3au ^{13}C NMR

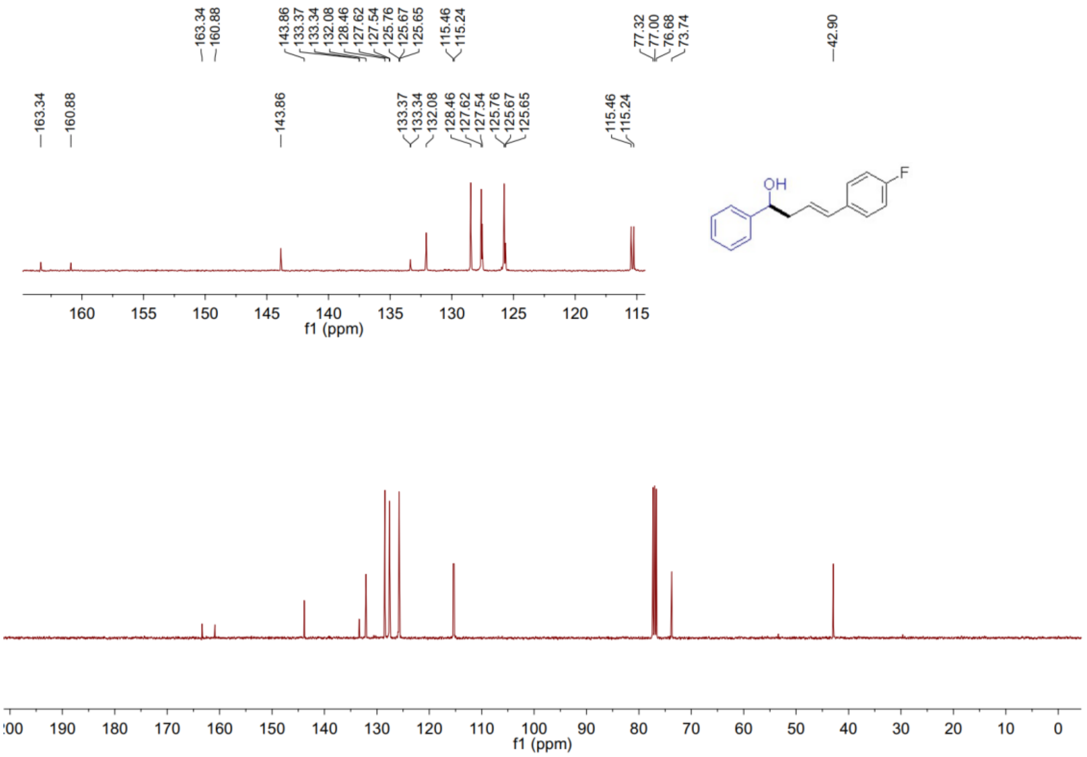

3av ^{1}H NMR

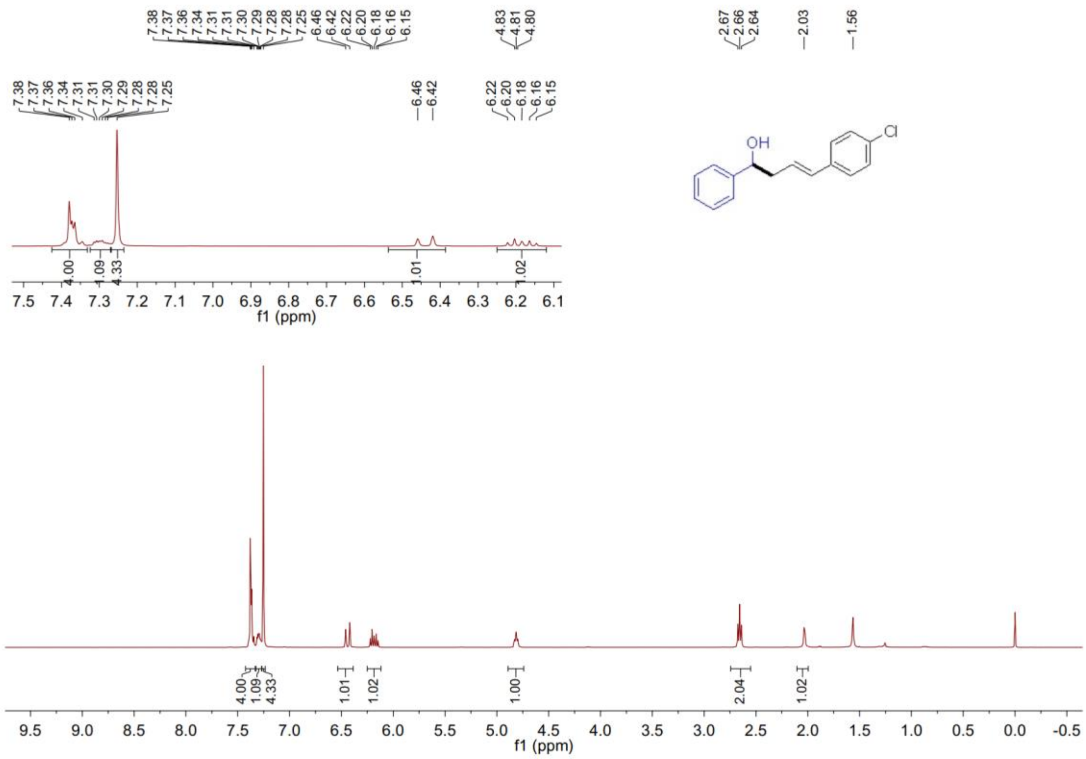

3av ^{13}C NMR

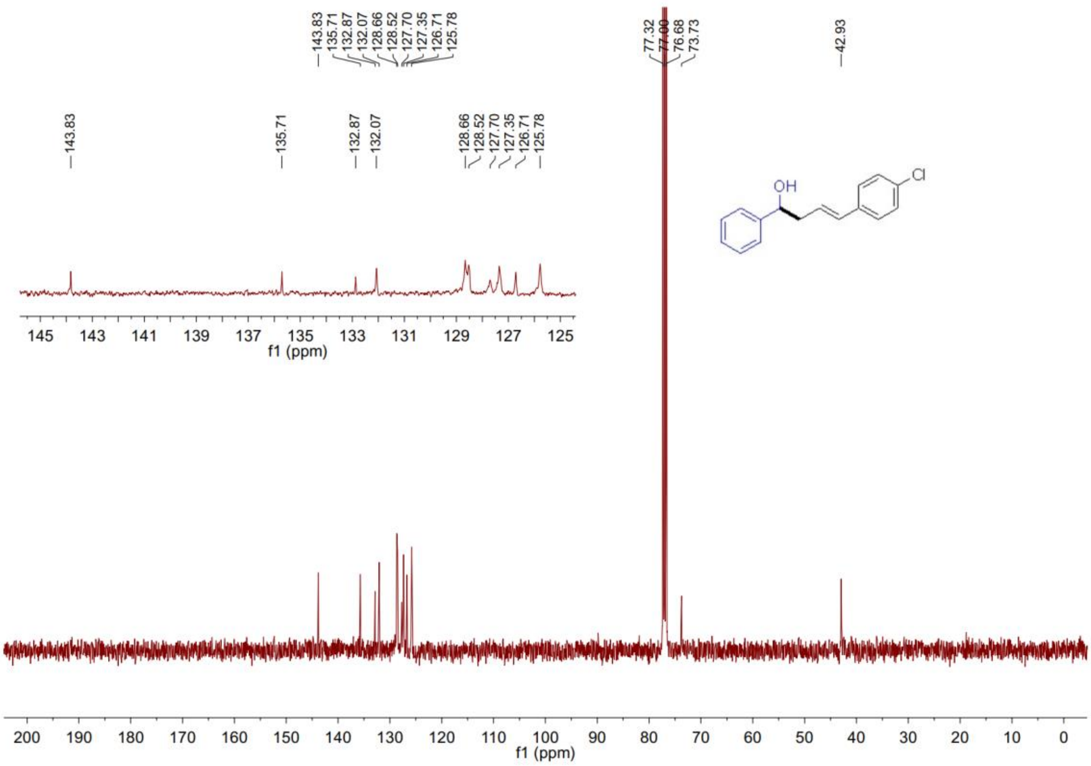

3aw ^{1}H NMR

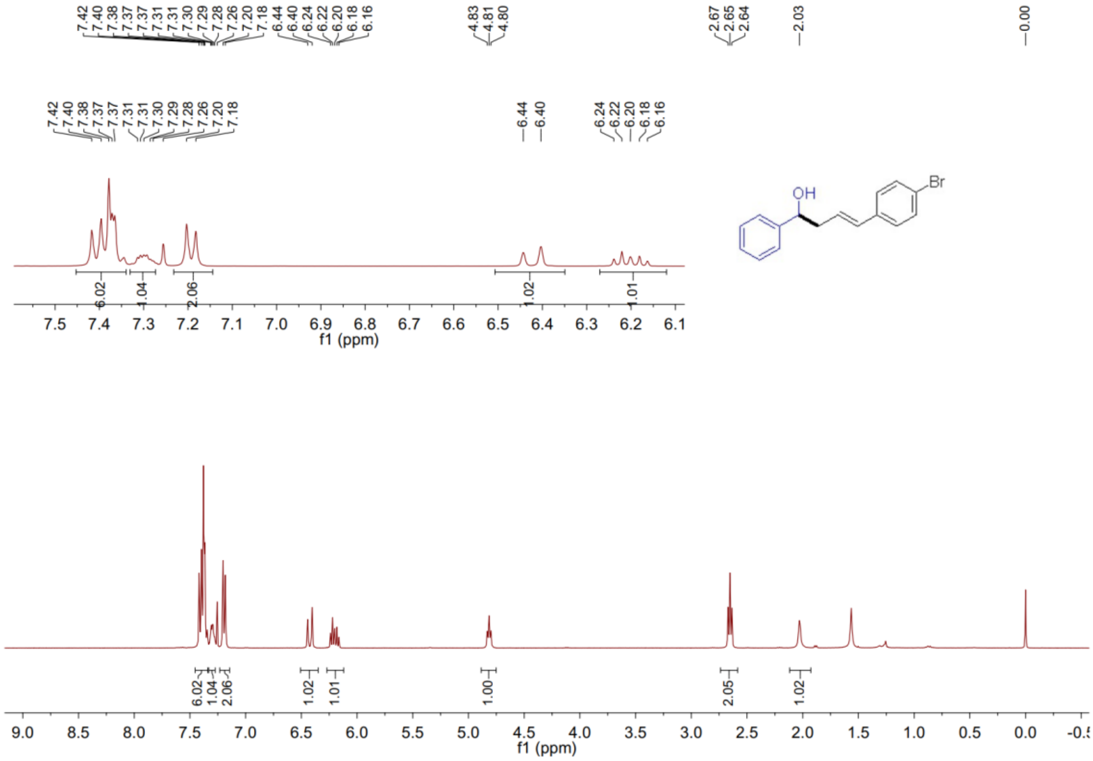

3aw ^{13}C NMR

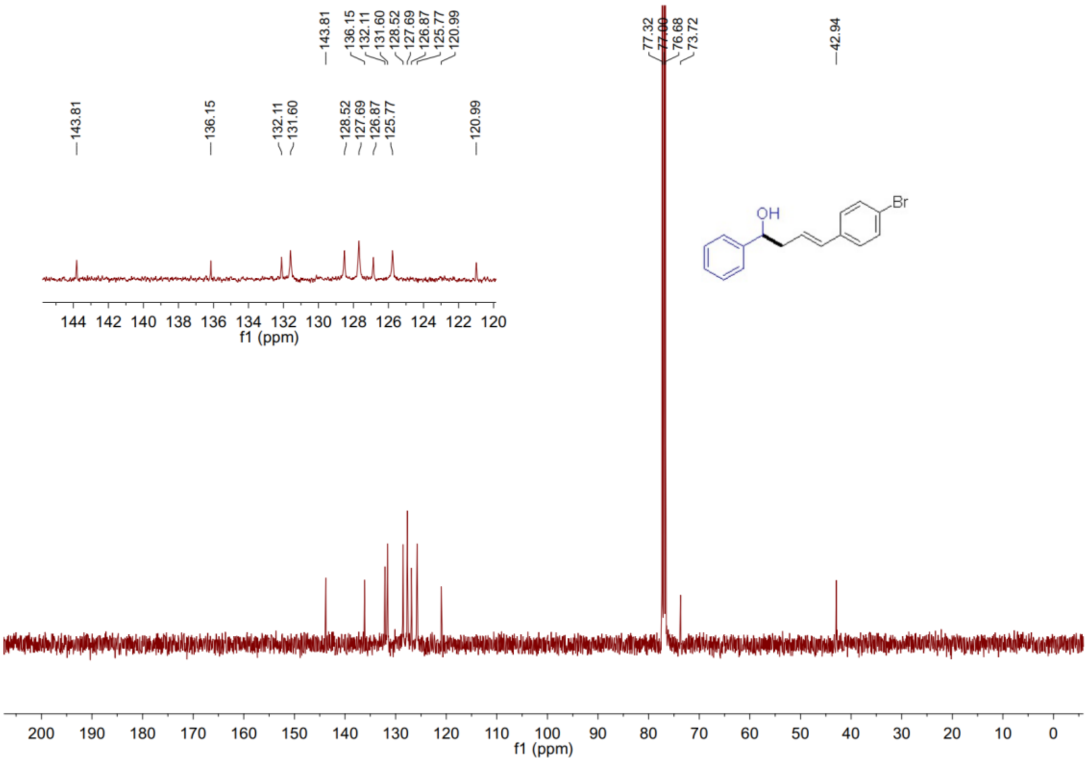

3ax ^{1}H NMR

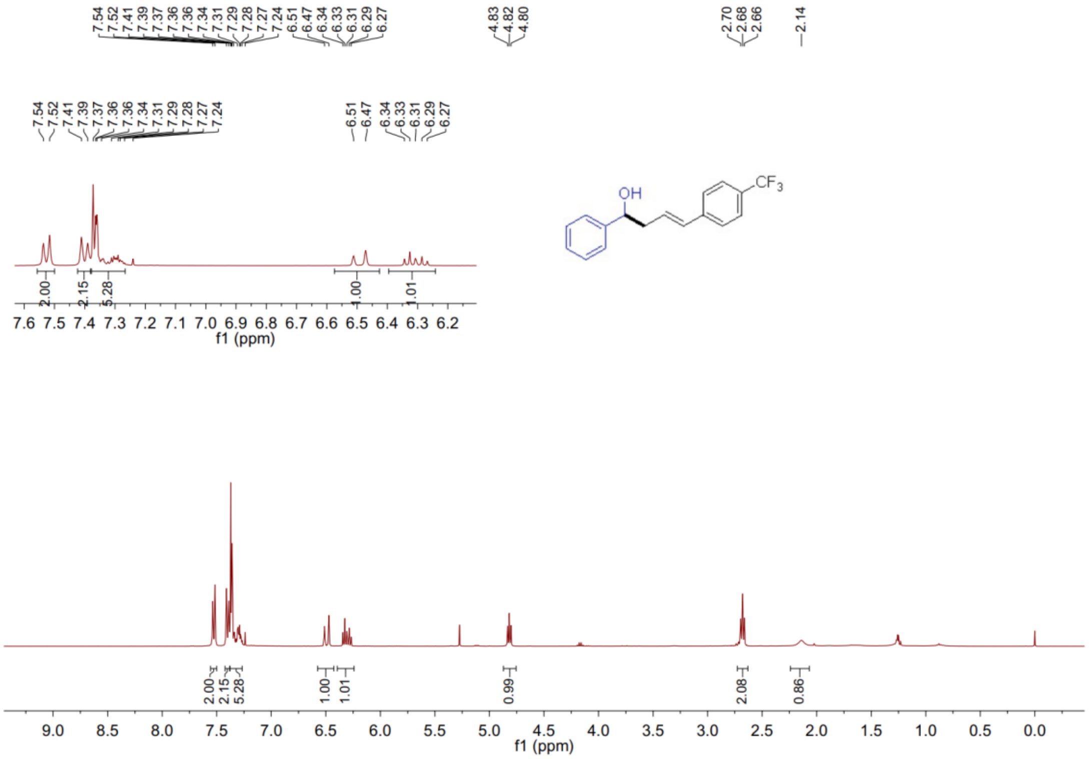

3ax ^{13}C NMR

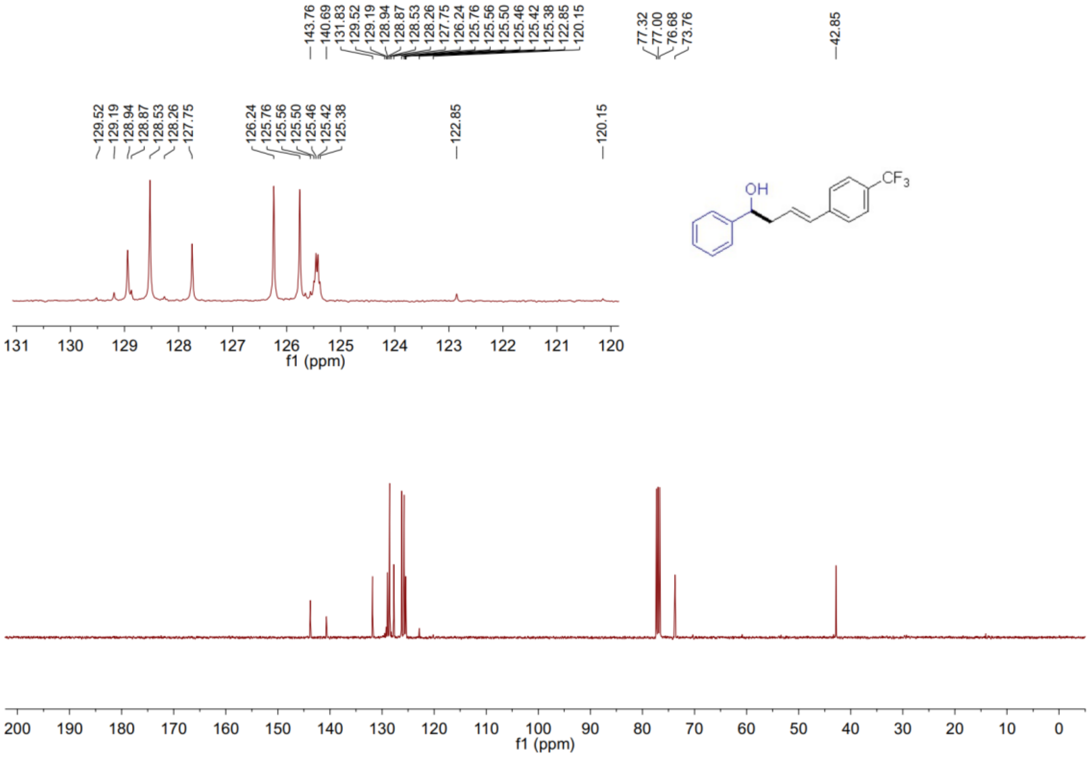

3ay ^{1}H NMR

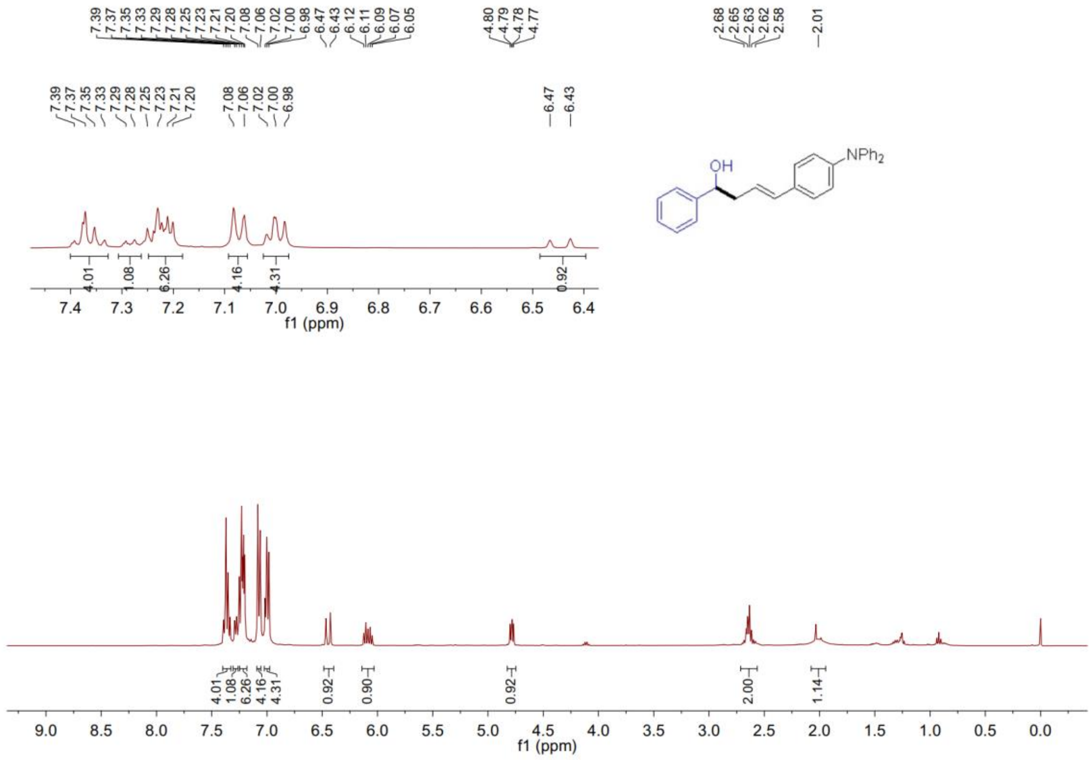

3ay ^{13}C NMR

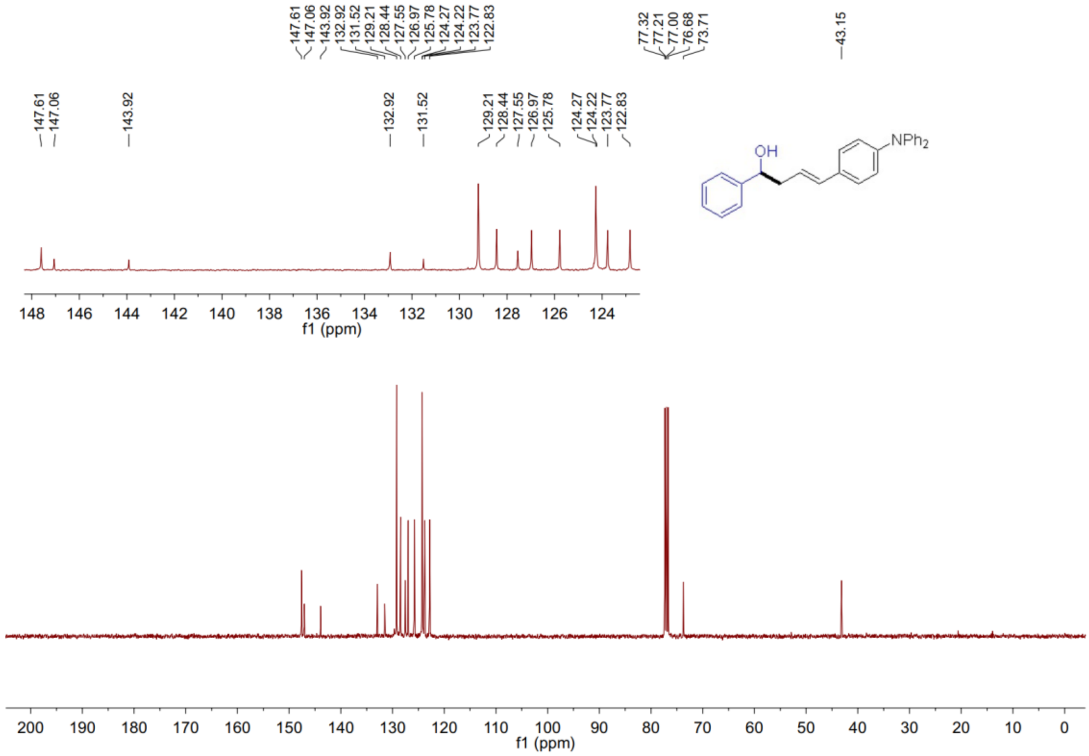

3az ^{1}H NMR

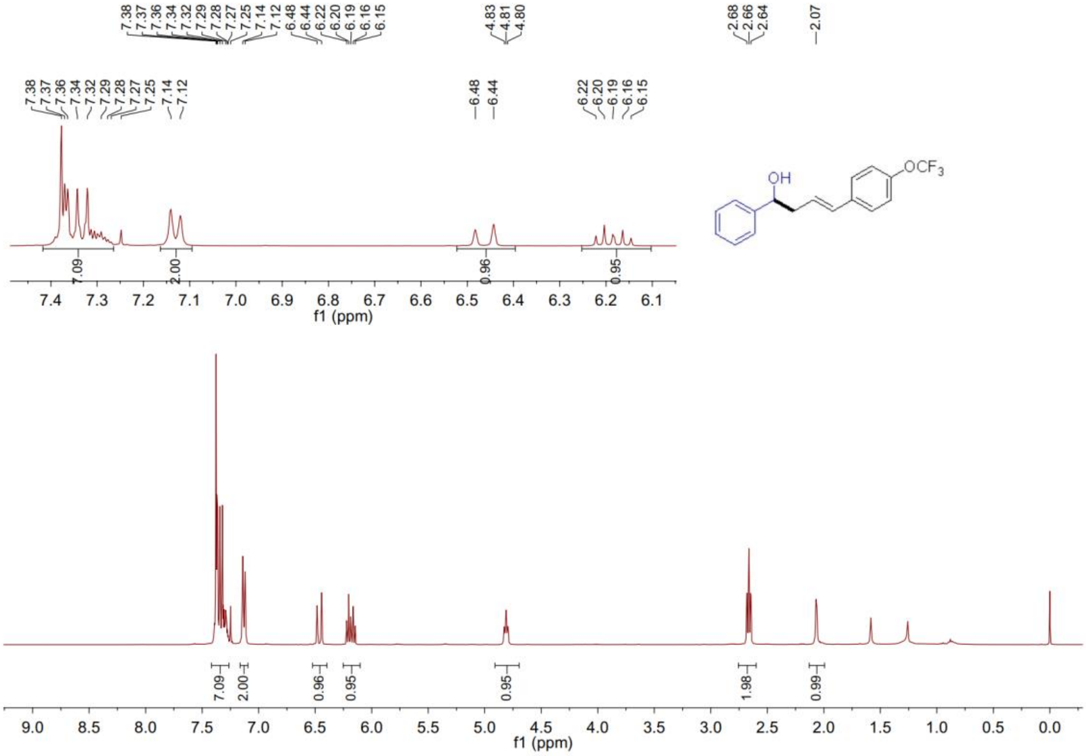

3az ^{13}C NMR

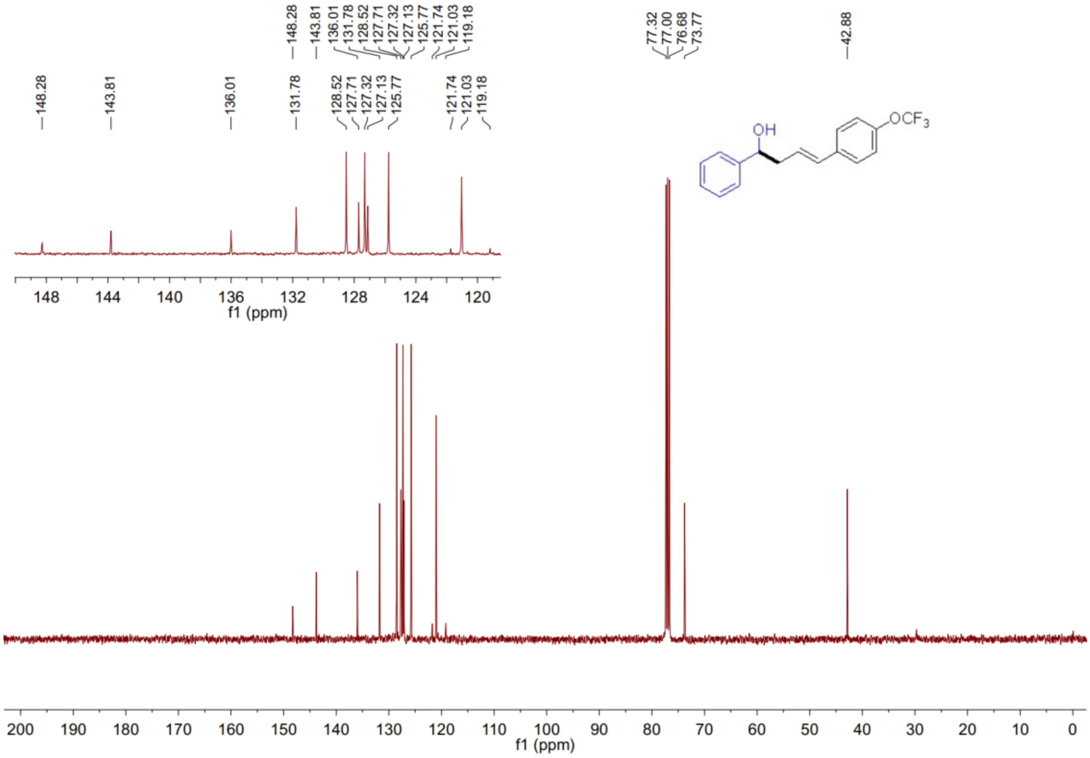

3ba ^{1}H NMR

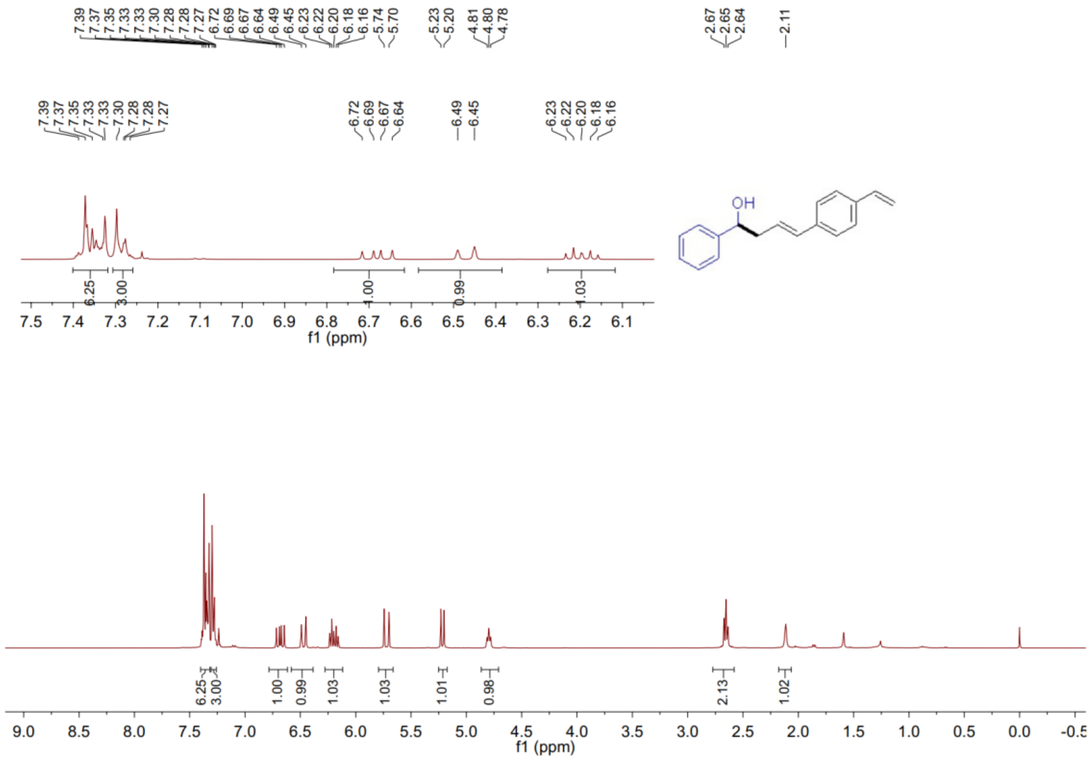

3ba ^{13}C NMR

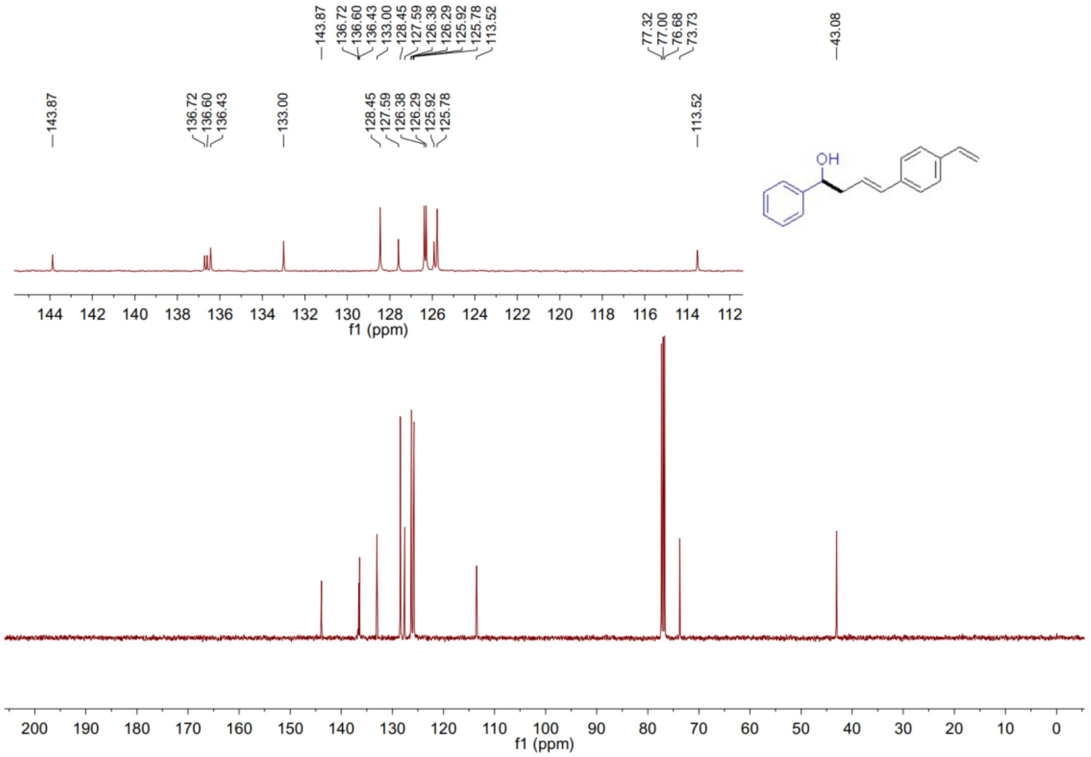

3bb ^{1}H NMR

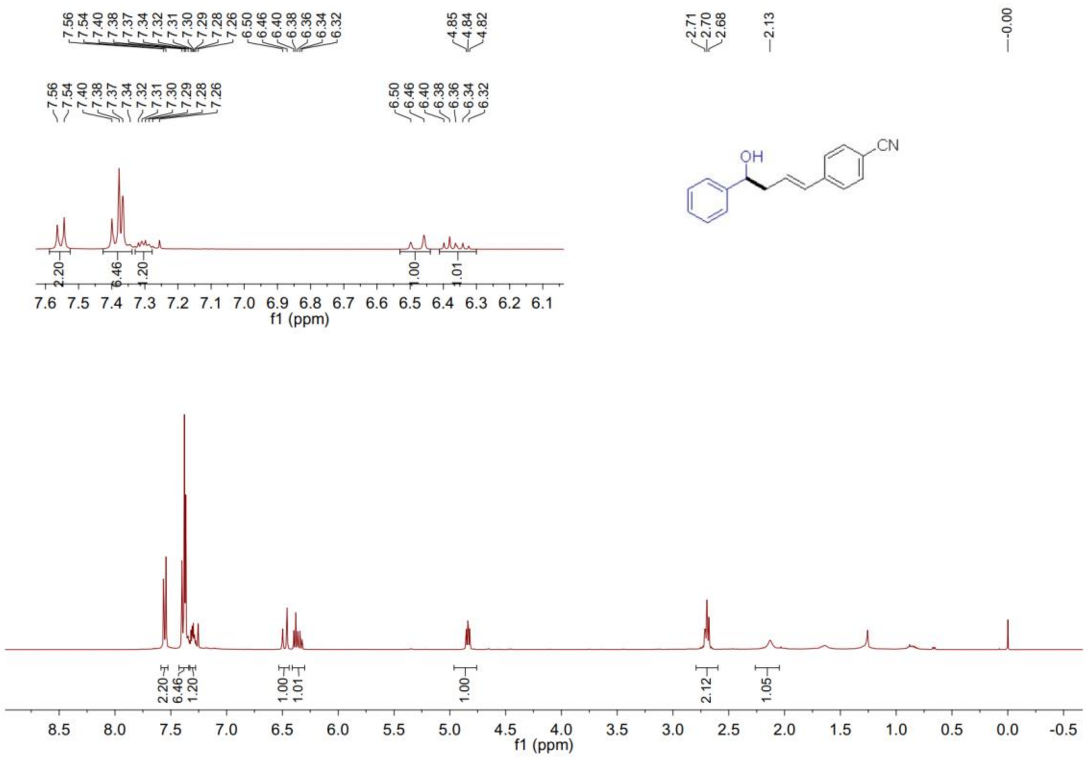

3bb ^{13}C NMR

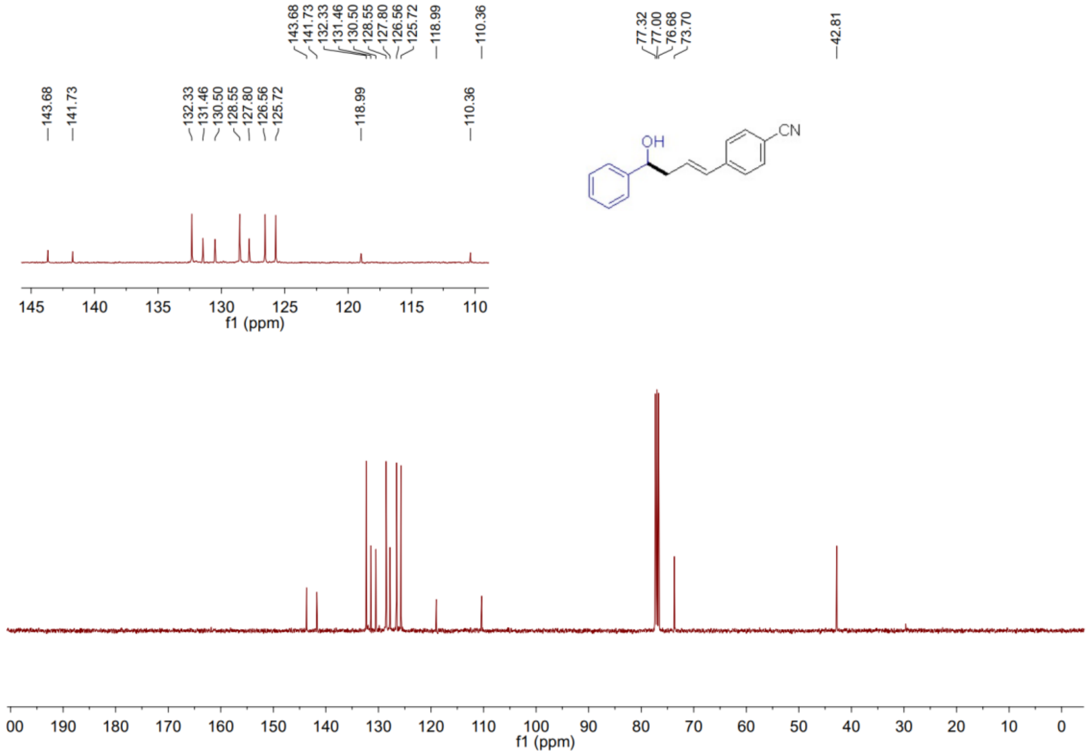

3bc ^{1}H NMR

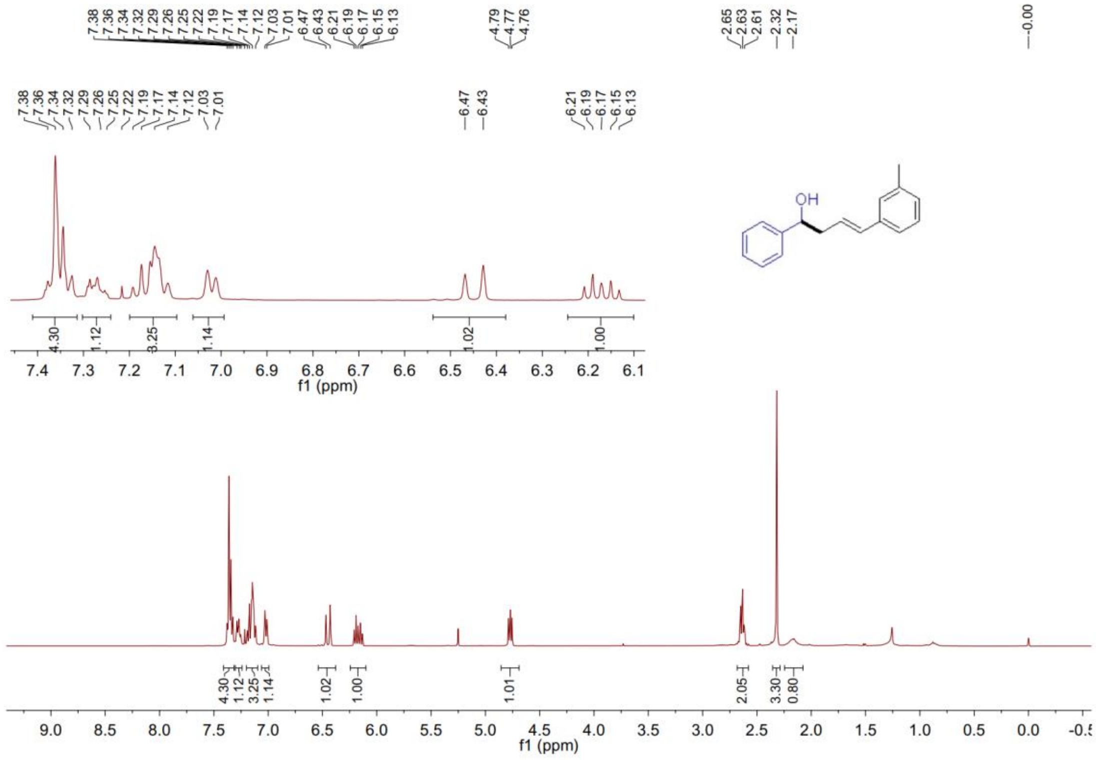

3bc ^{13}C NMR

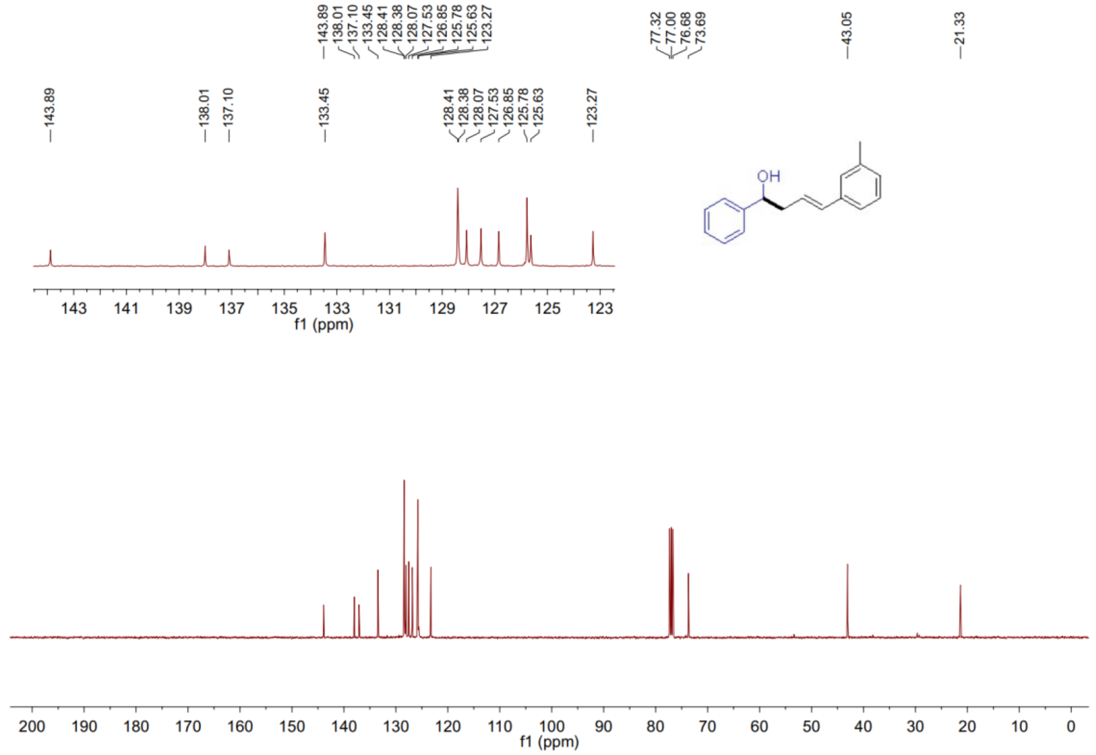

3bd ^{1}H NMR

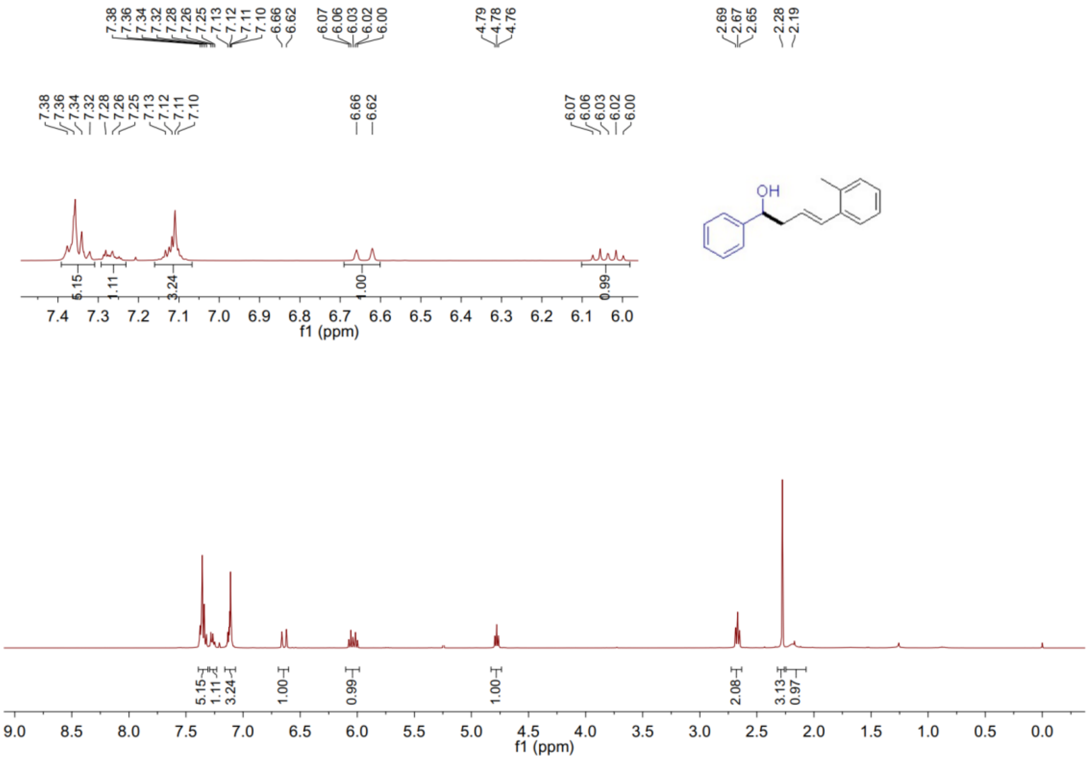

3bd ^{13}C NMR

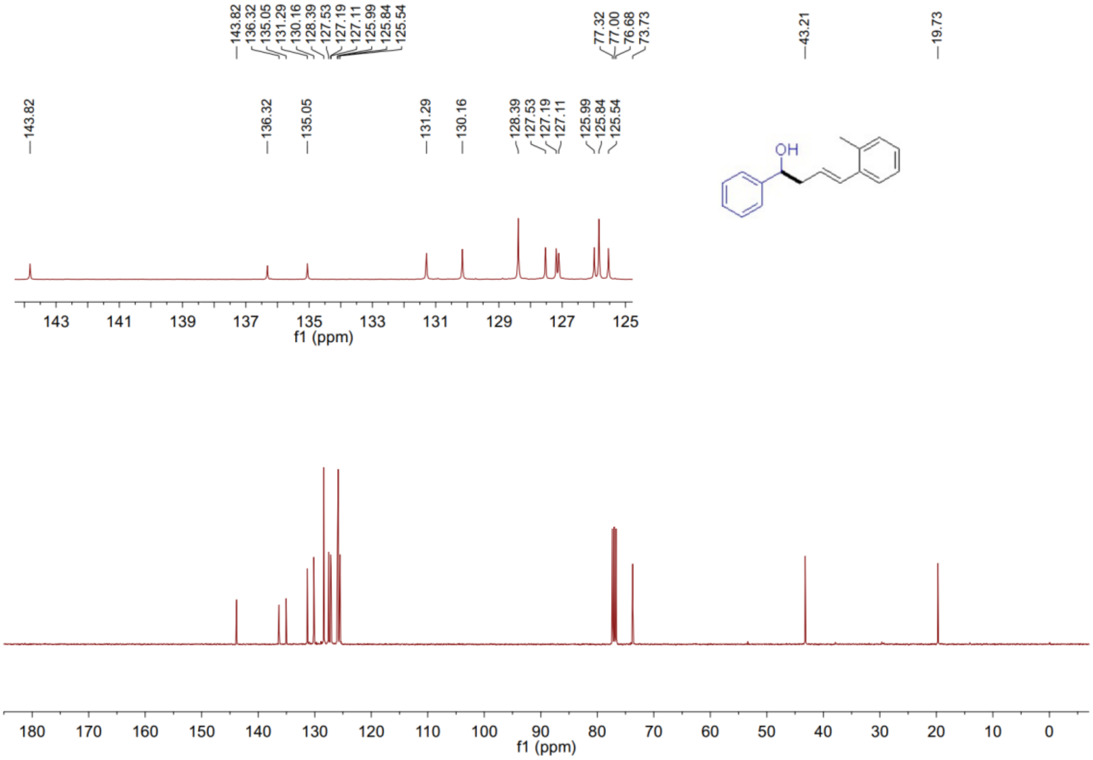

3be ^{1}H NMR

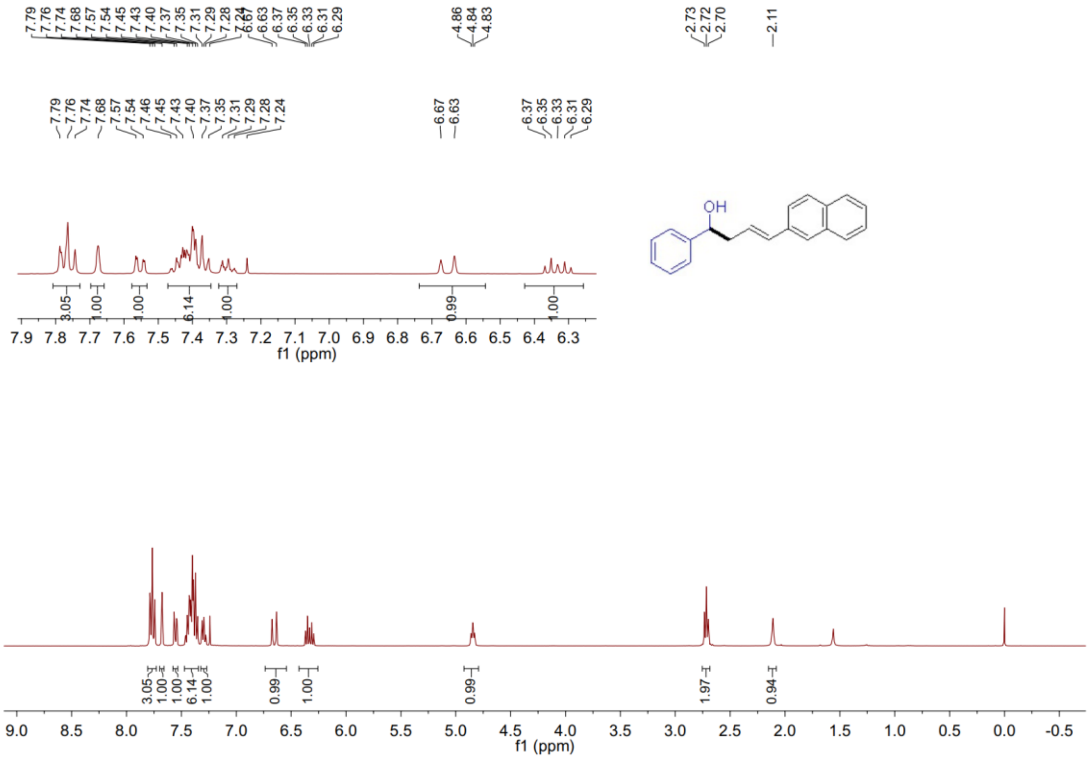

3be ^{13}C NMR

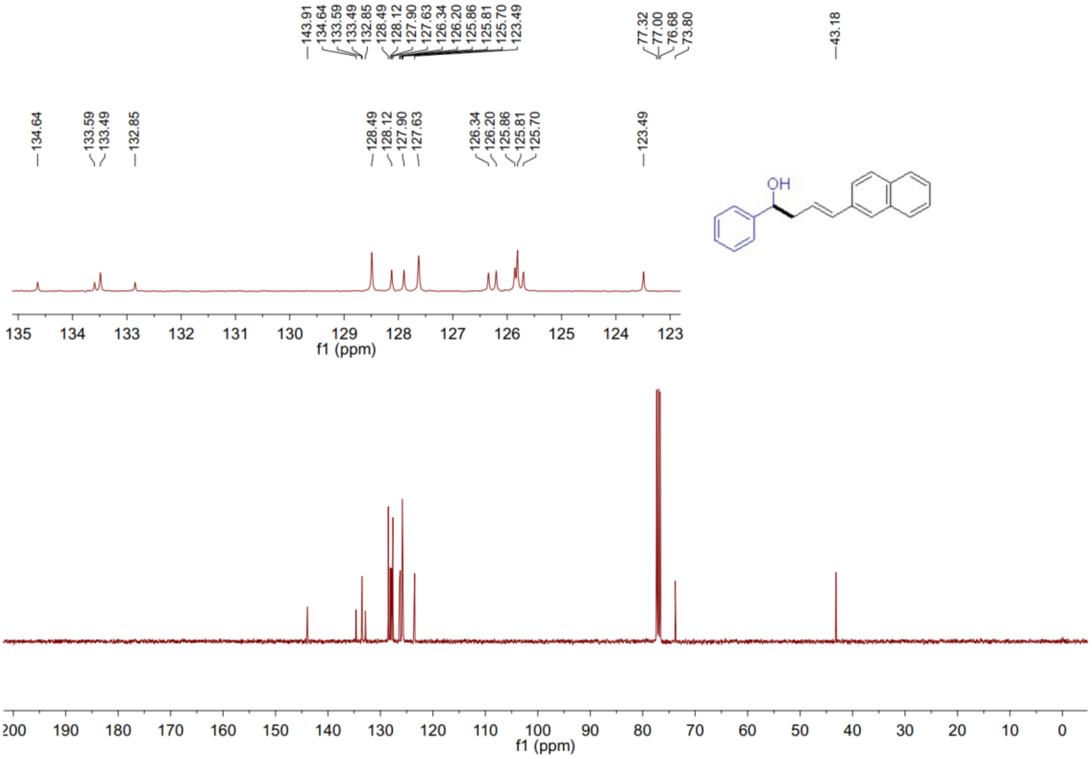

3bf ^{1}H NMR

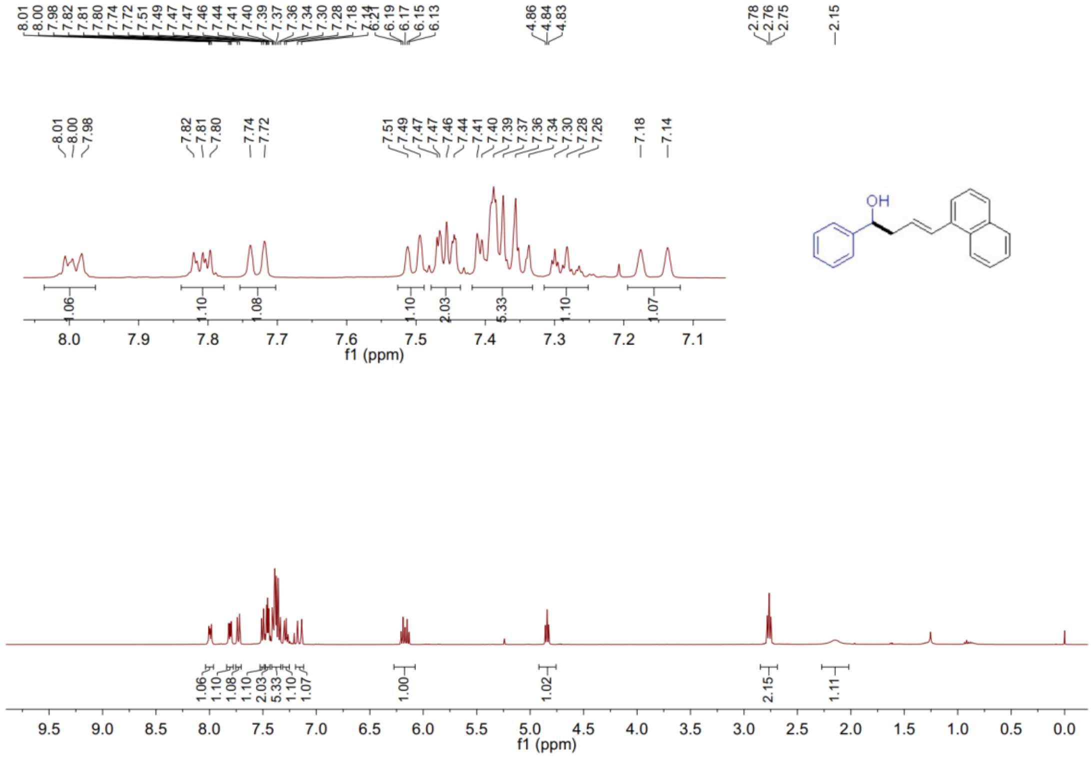

3bf ^{13}C NMR

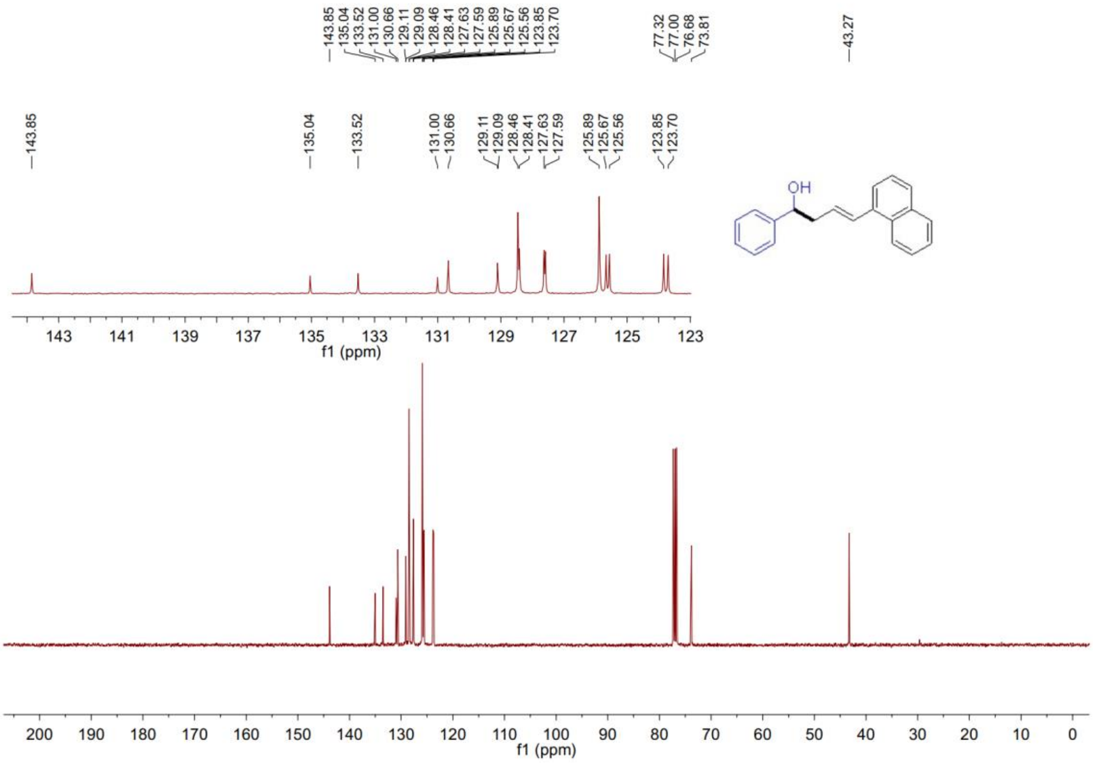

3bg ^{1}H NMR

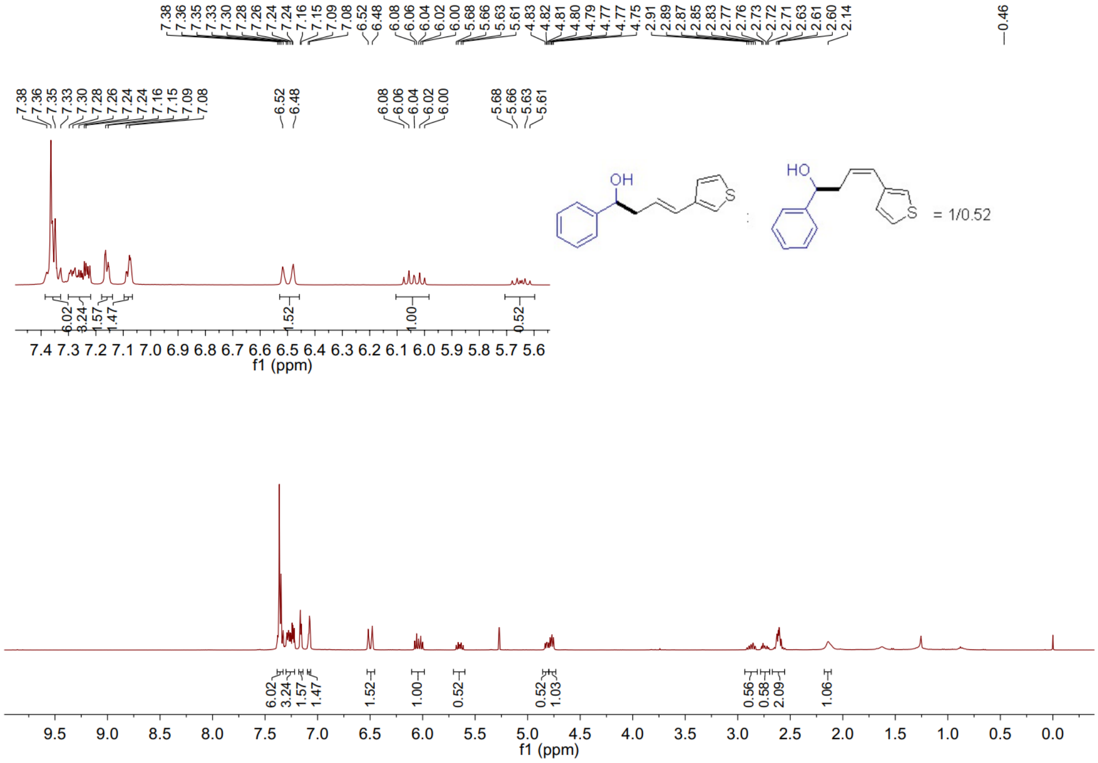

3bg ^{13}C NMR

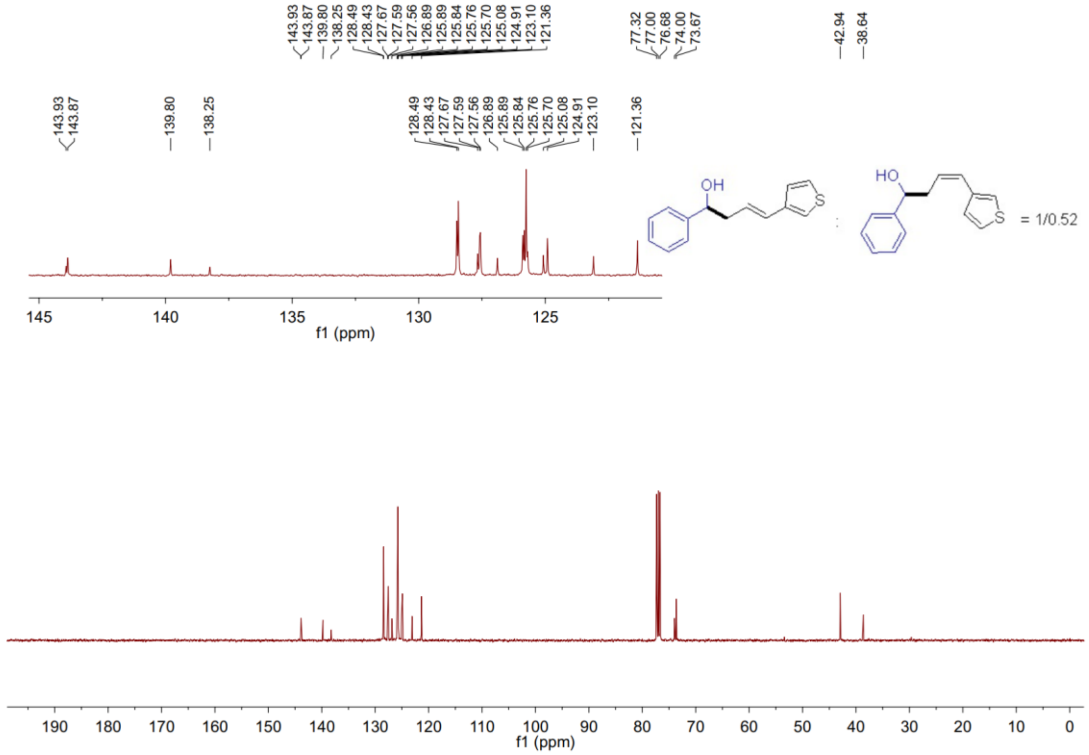

3bh ^{1}H NMR

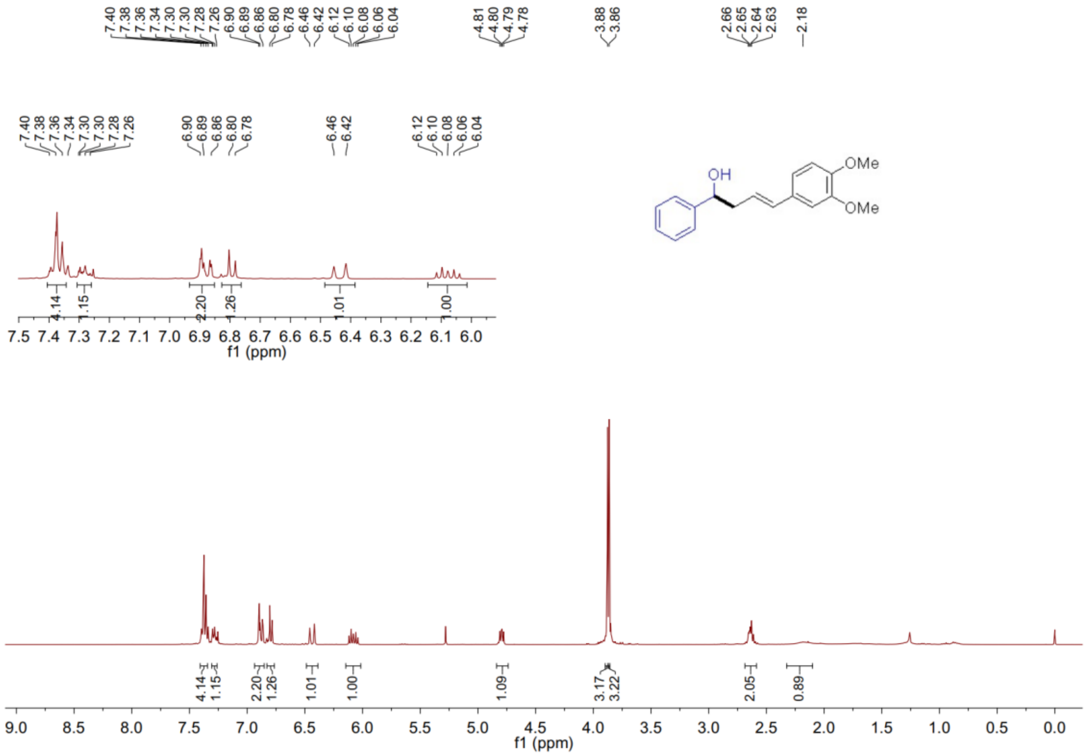

3bh^{13}C NMR

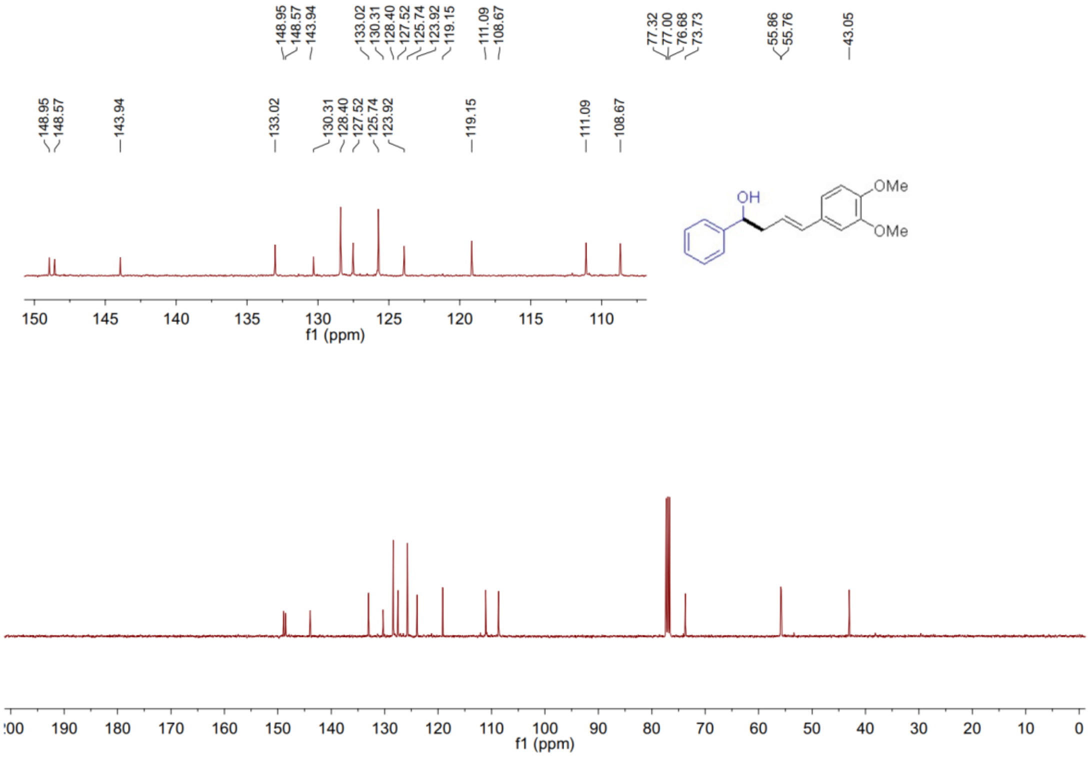

3bi ¹H NMR

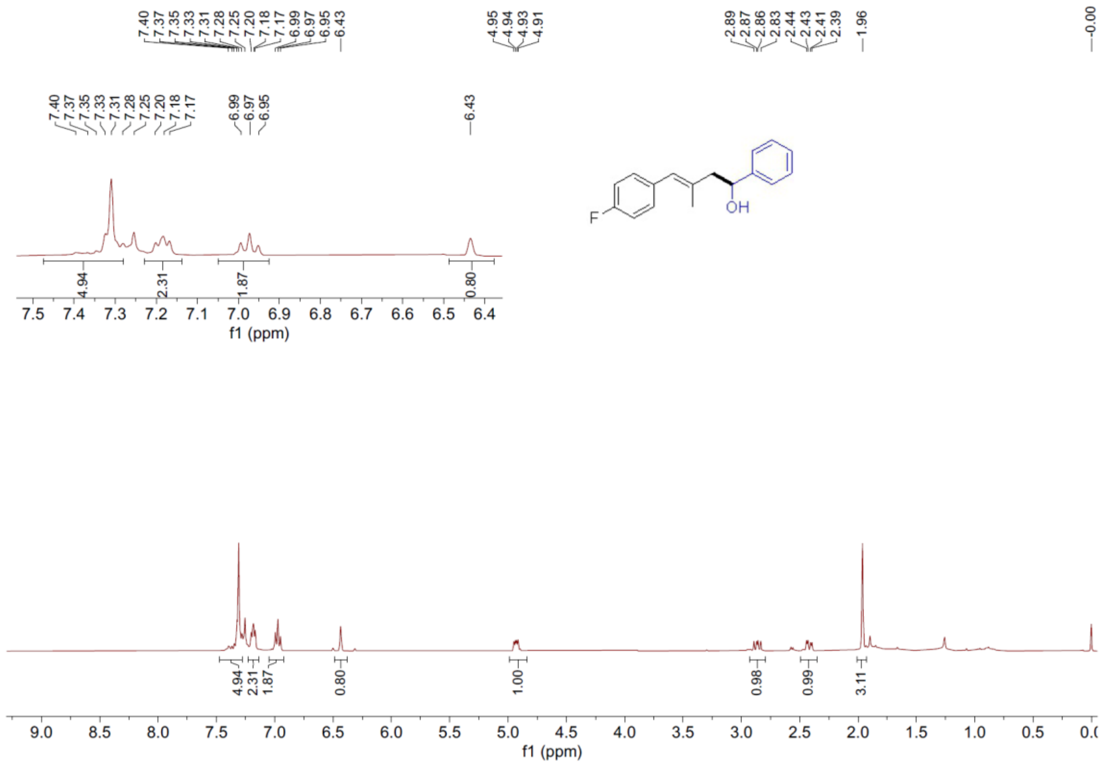

3bi *E/Z*

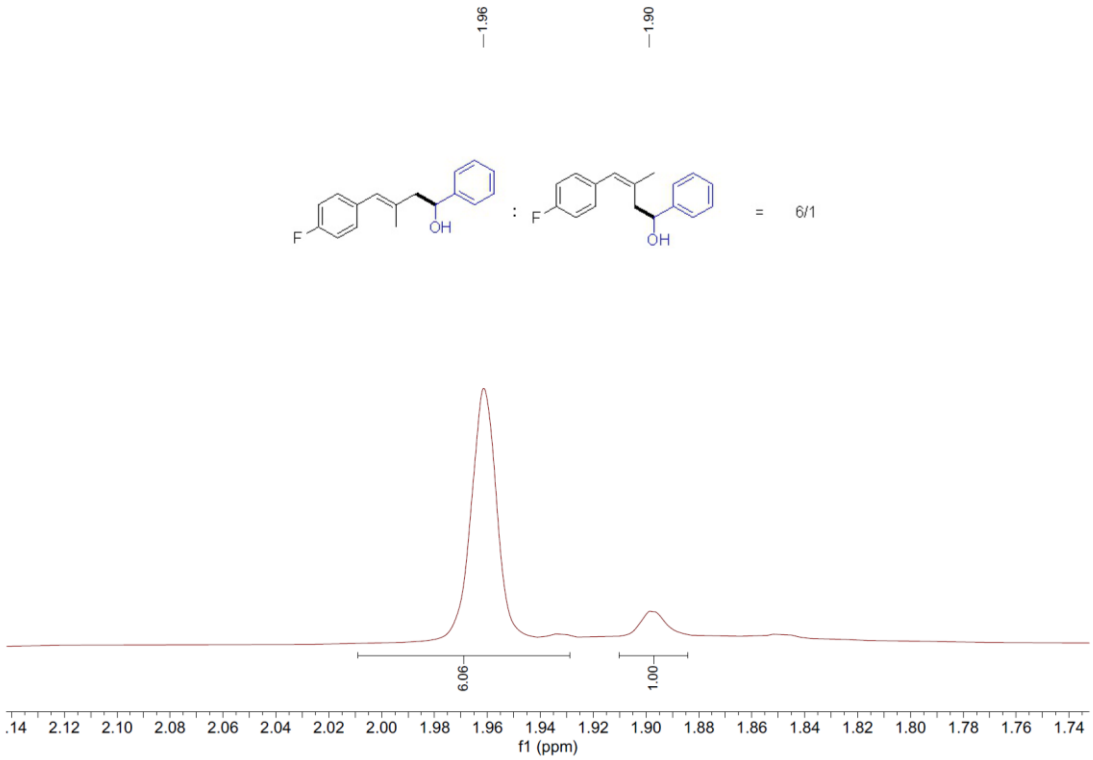

3bi ^{13}C NMR

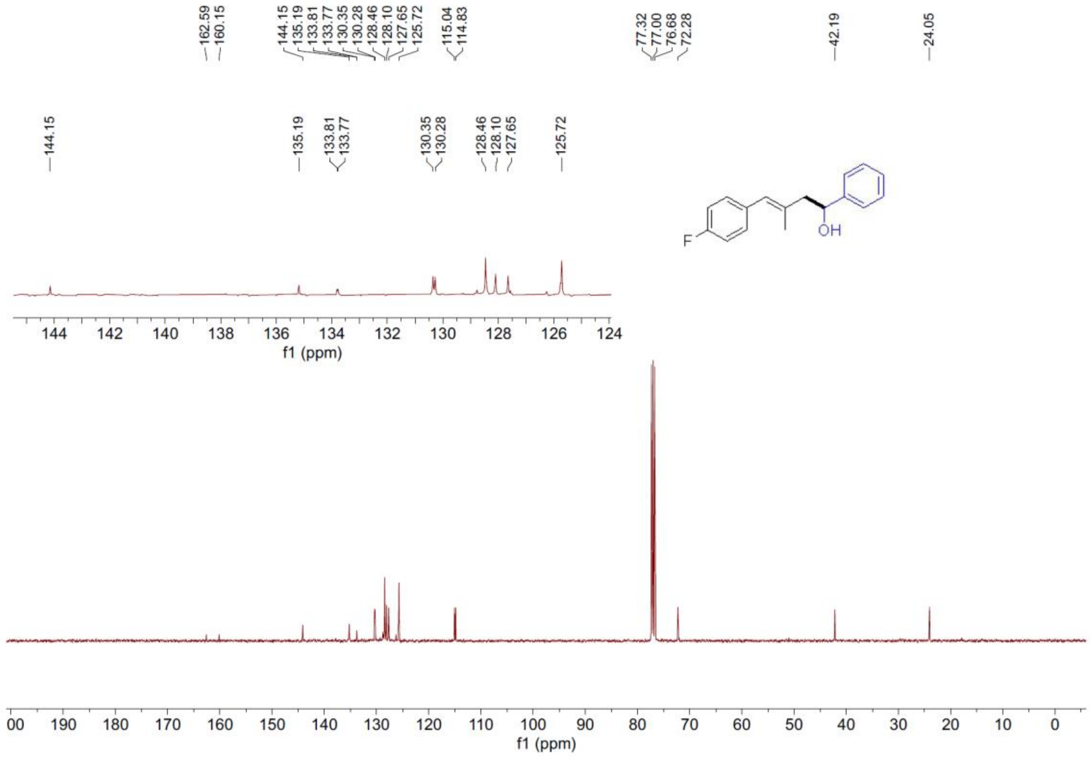

3bj^{1}H NMR

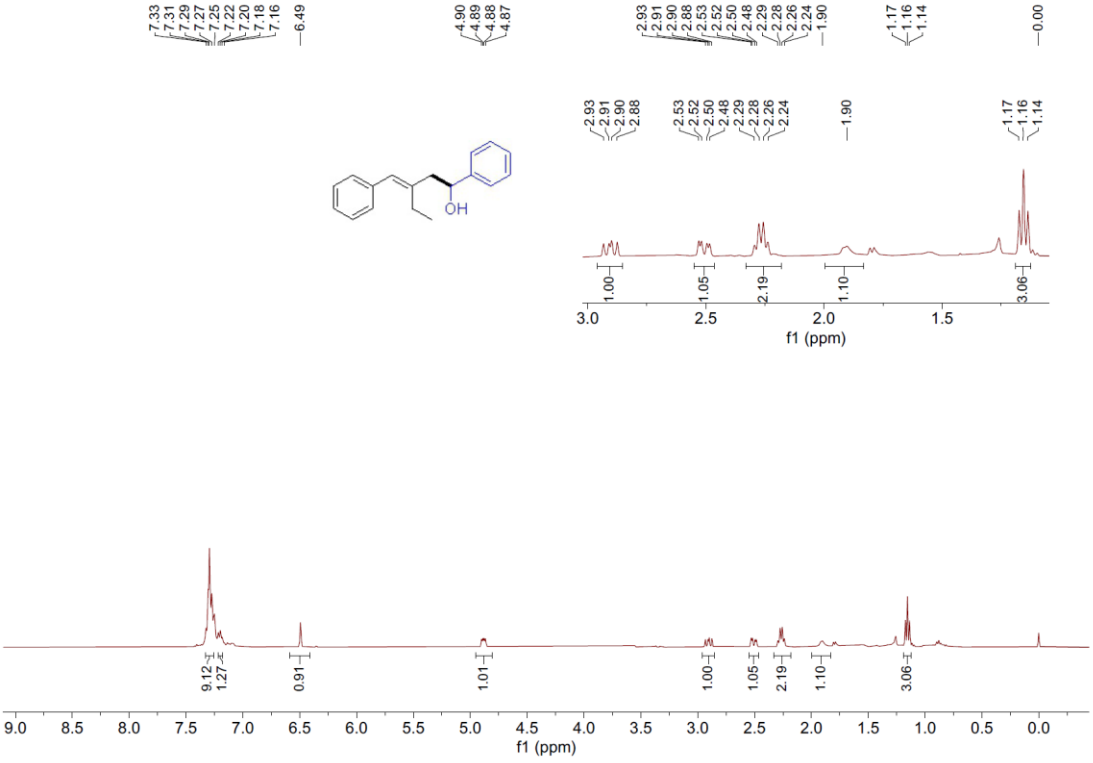

3bj *E/Z*

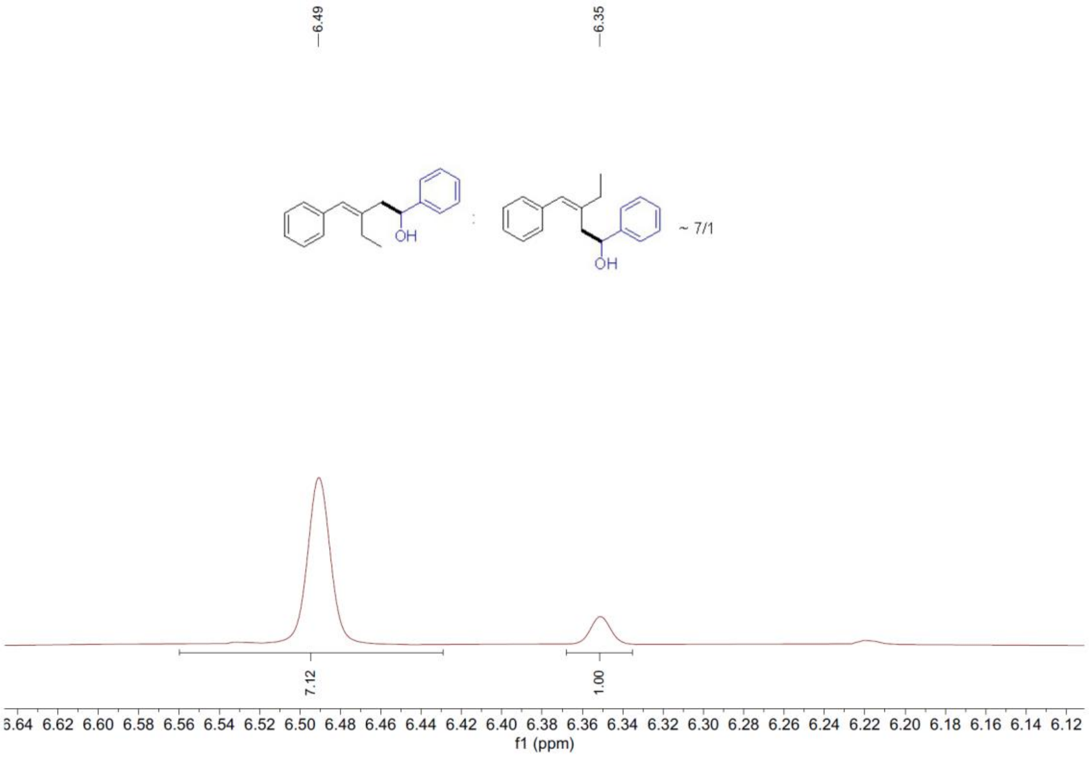

3bj ^{13}C NMR

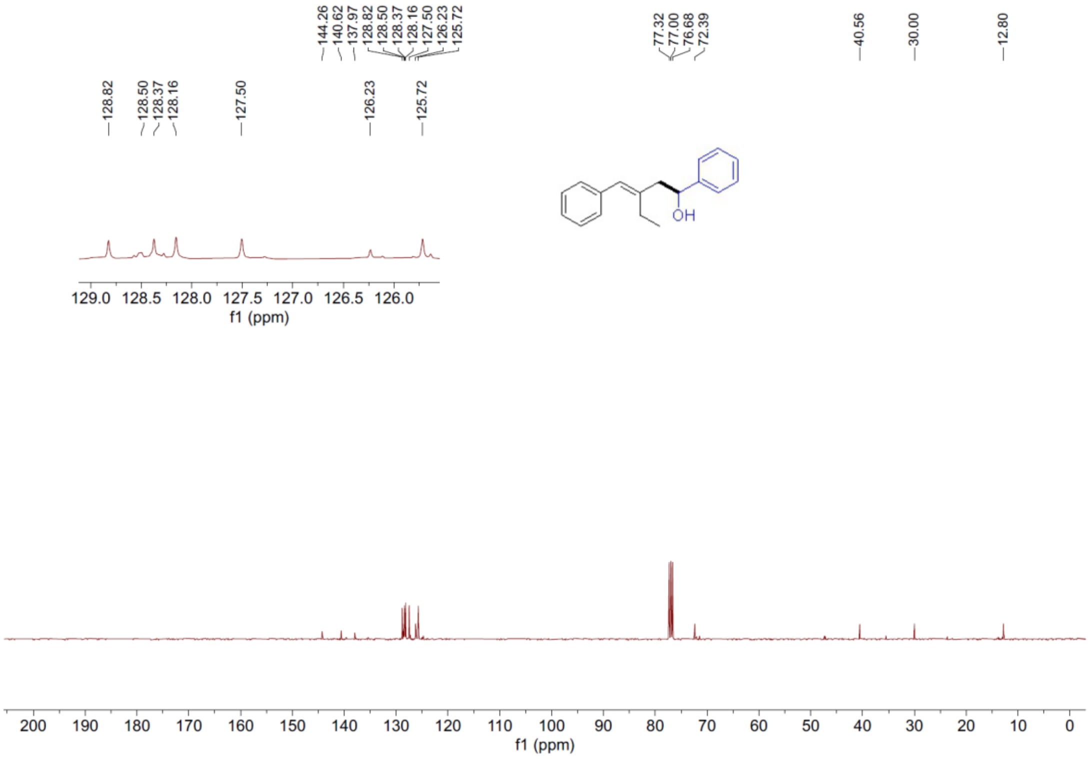

3bk^{1}H NMR

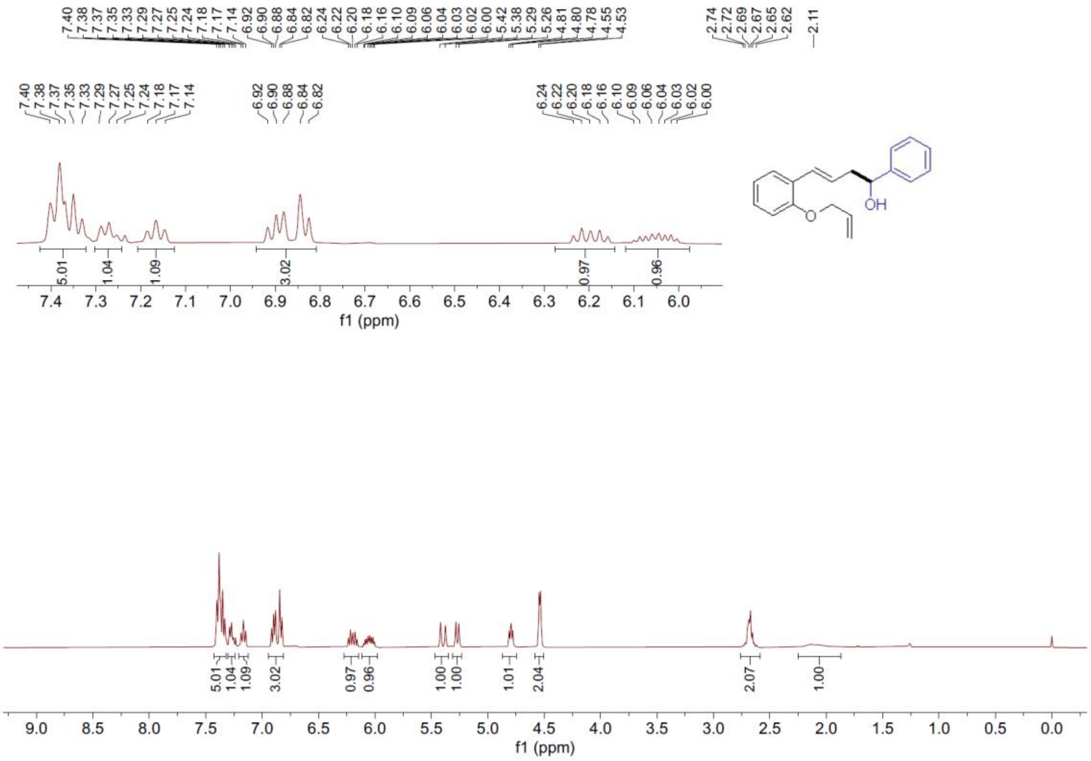

3bk ^{13}C NMR

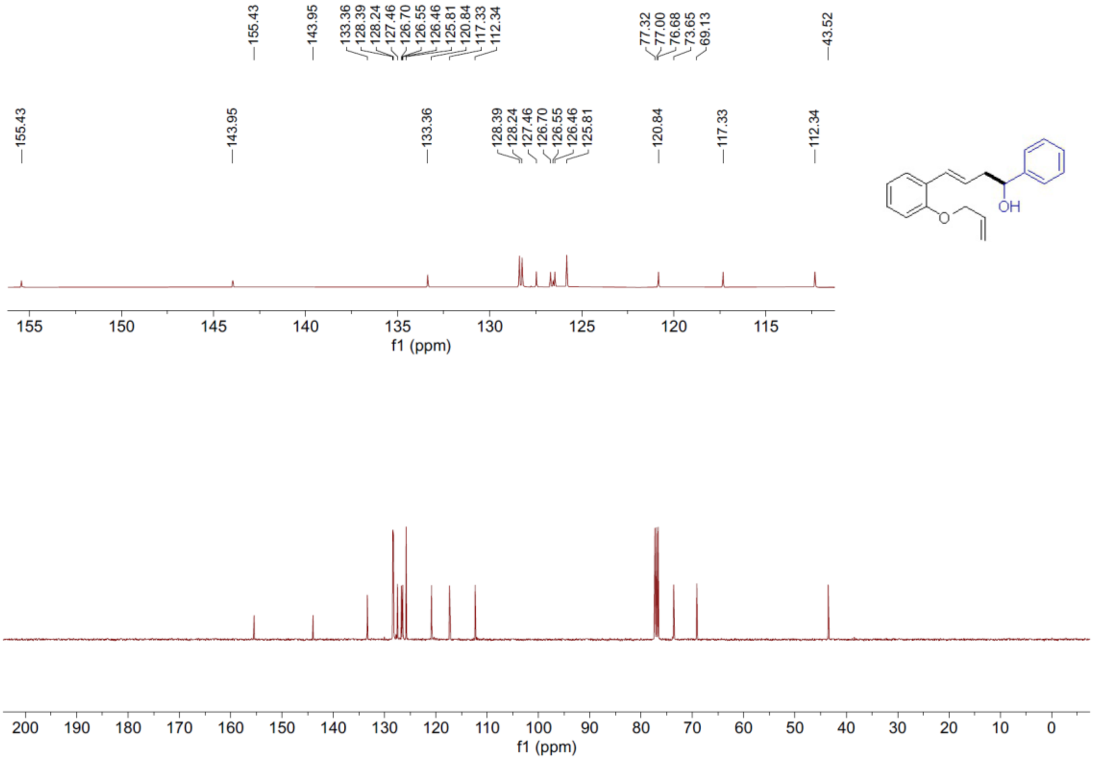

3bl ^{1}H NMR

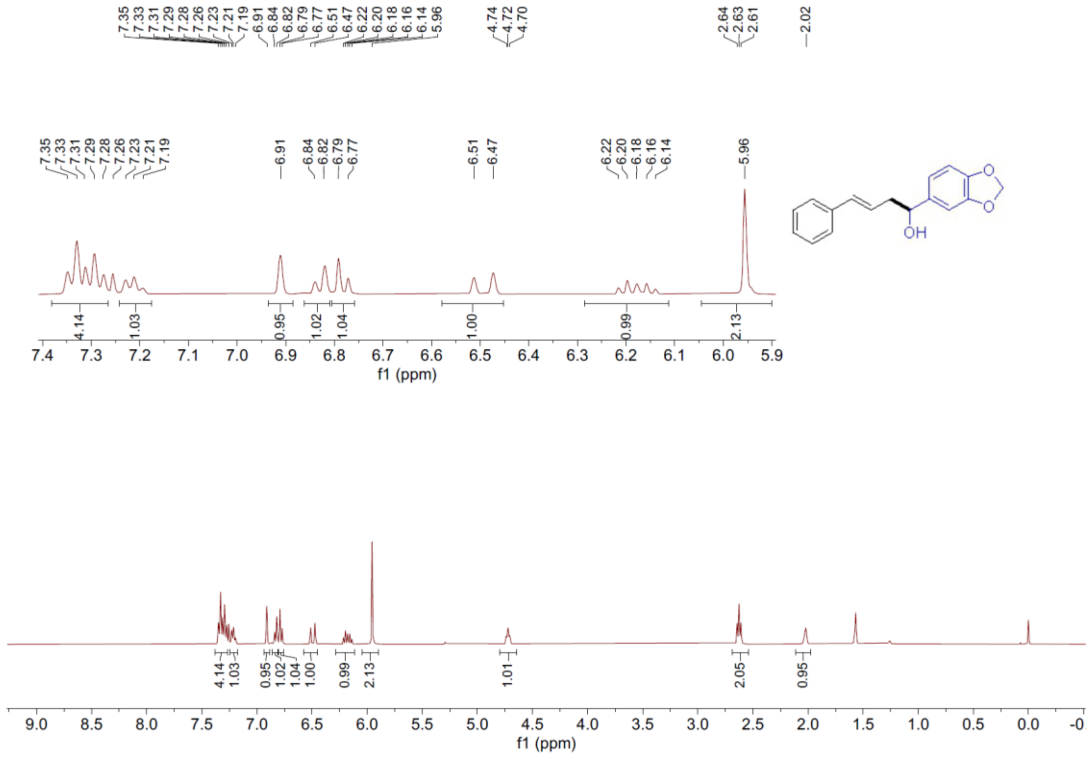

3bl ^{13}C NMR

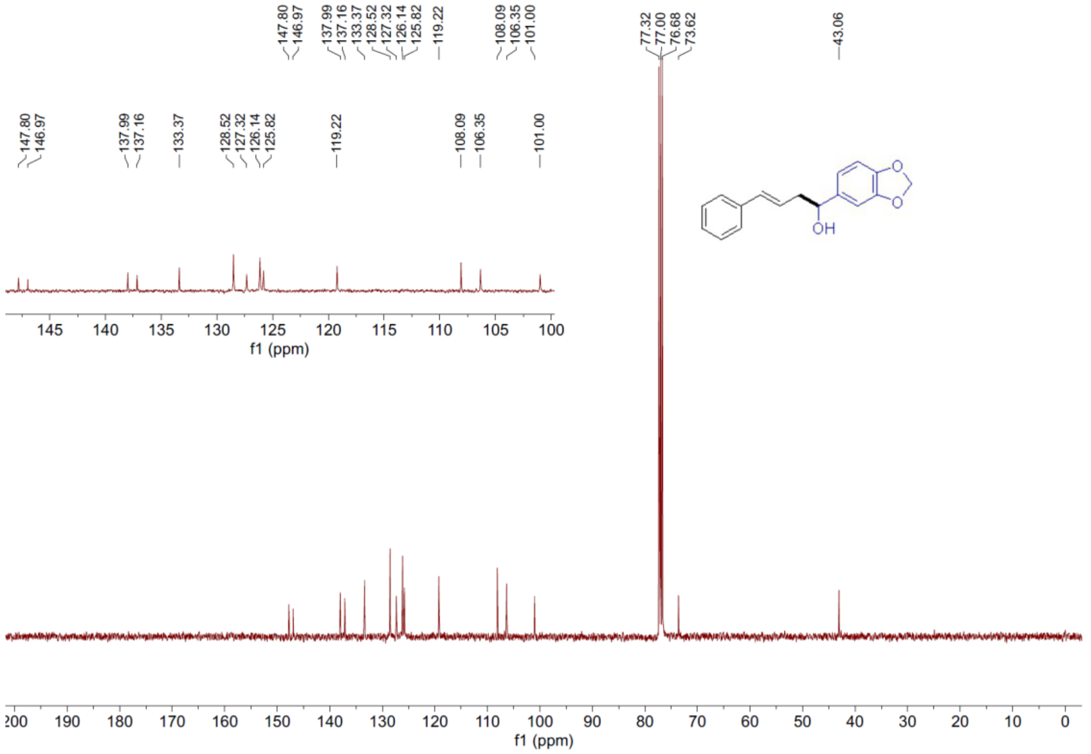

3bm ^{1}H NMR

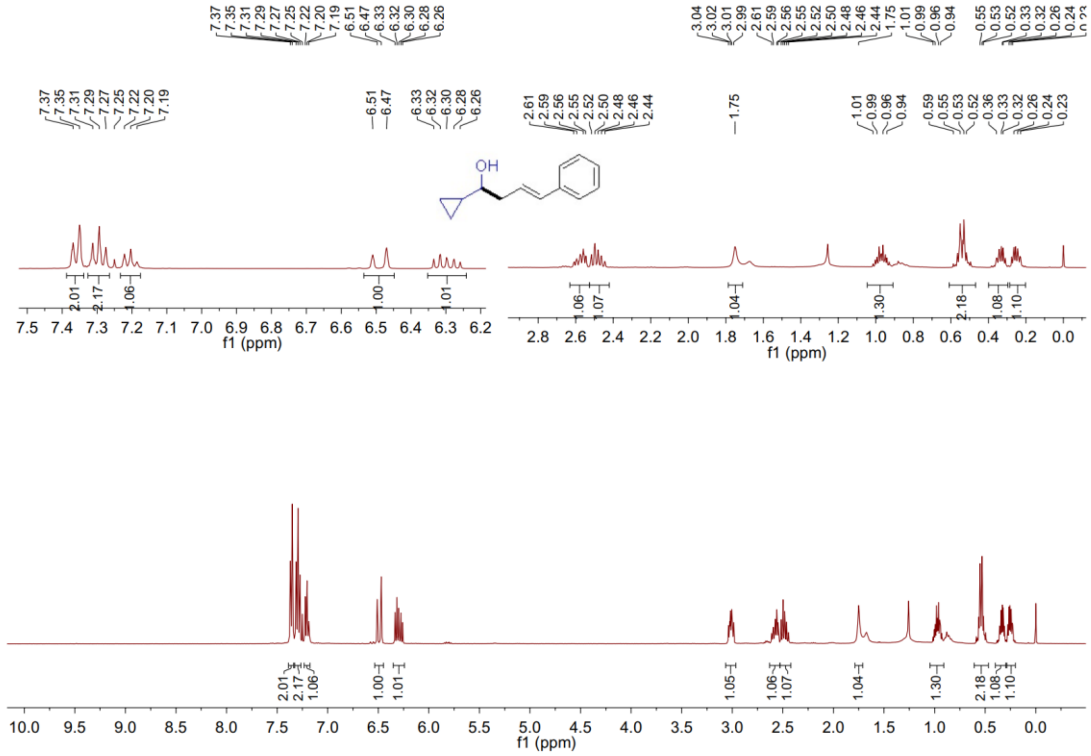

3bm ^{13}C NMR

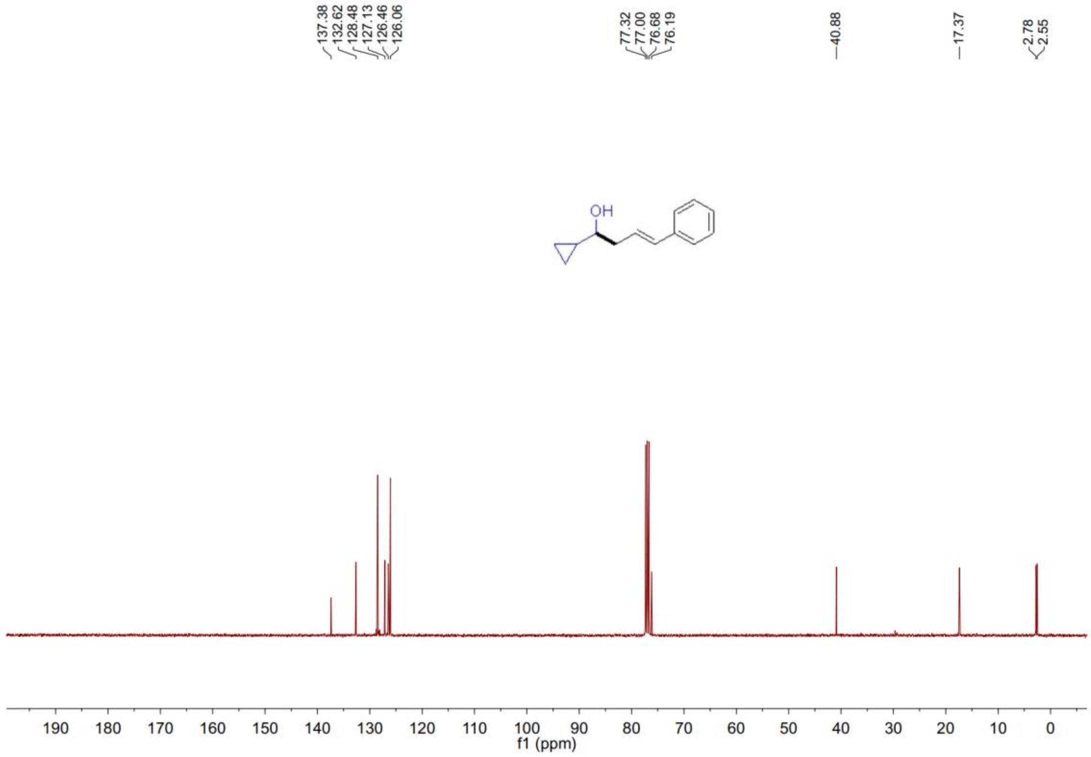

3bn isomer I ^{1}H NMR

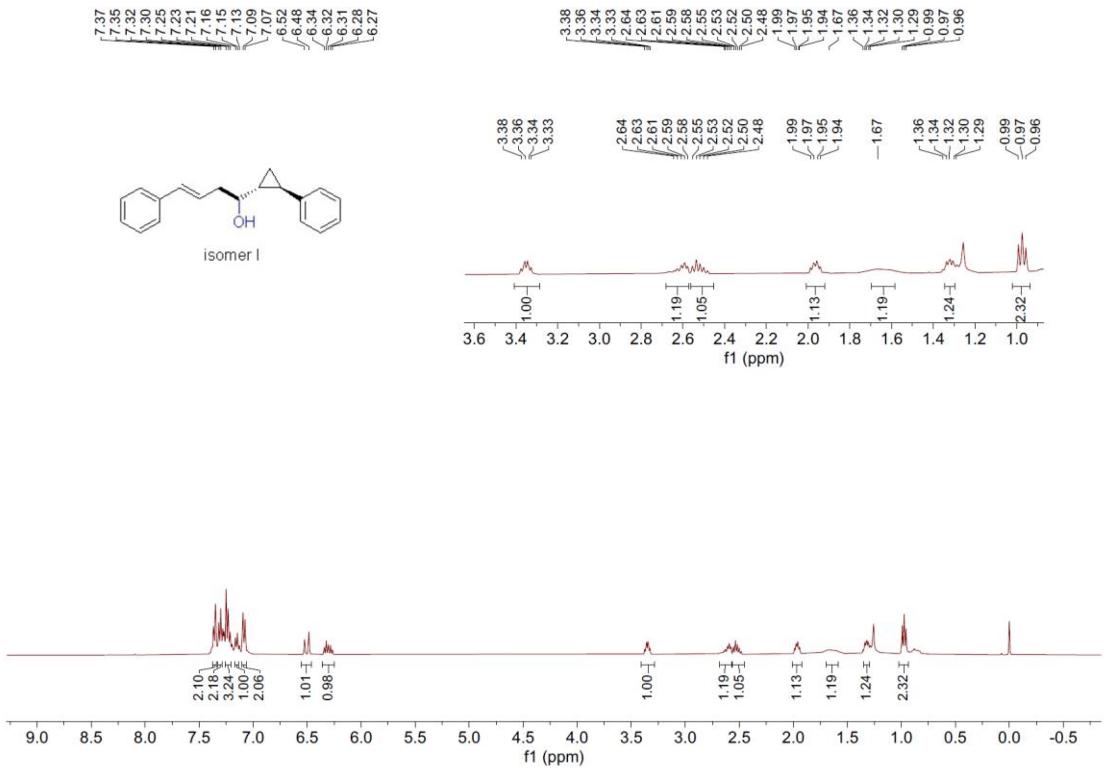

3bn isomer I ^{13}C NMR

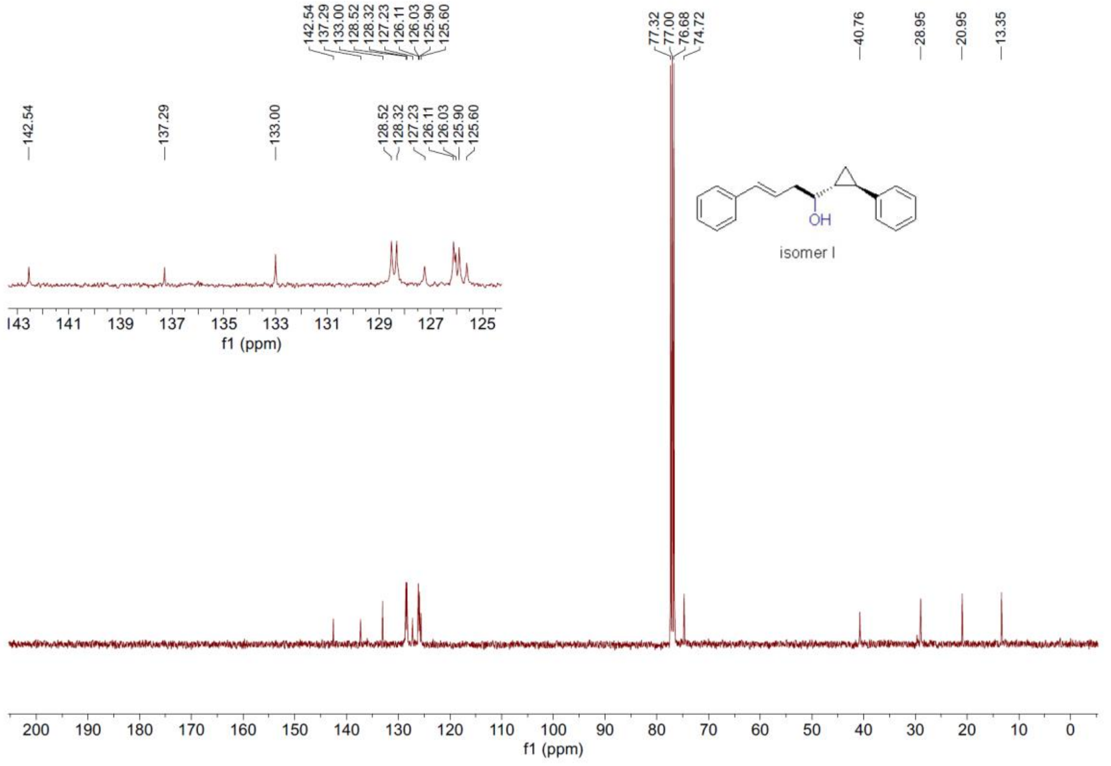

3bn isomer II ^{1}H NMR

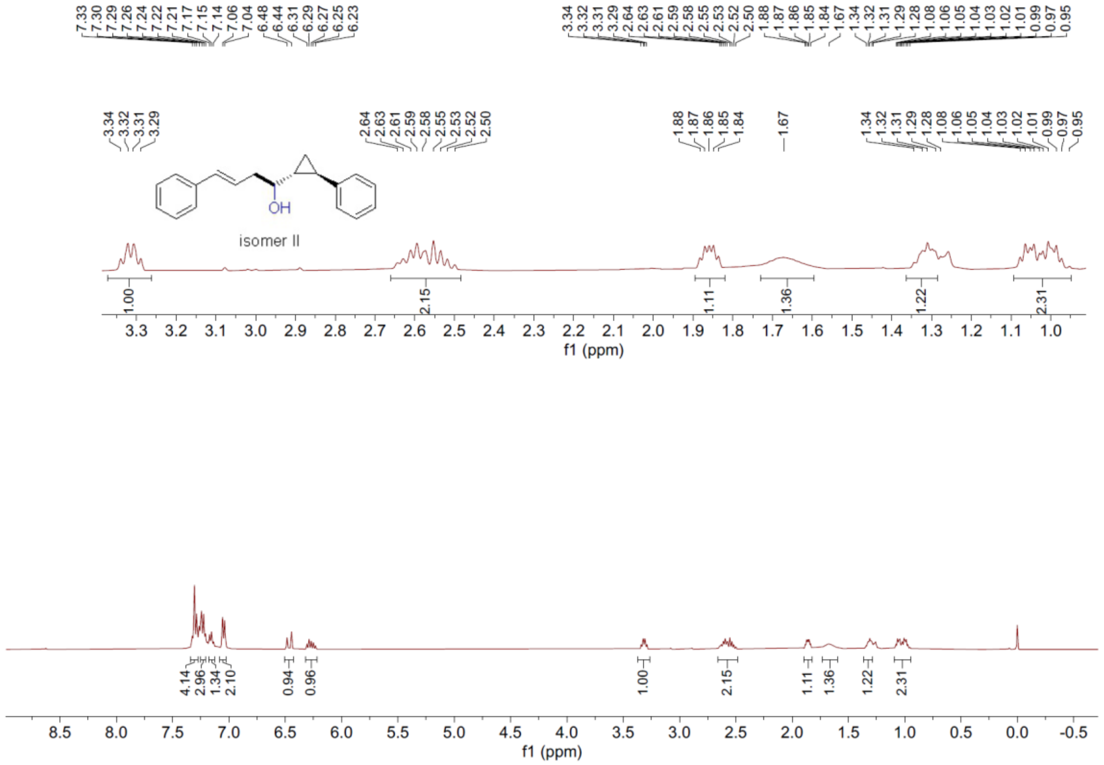

3bn isomer II ^{13}C NMR

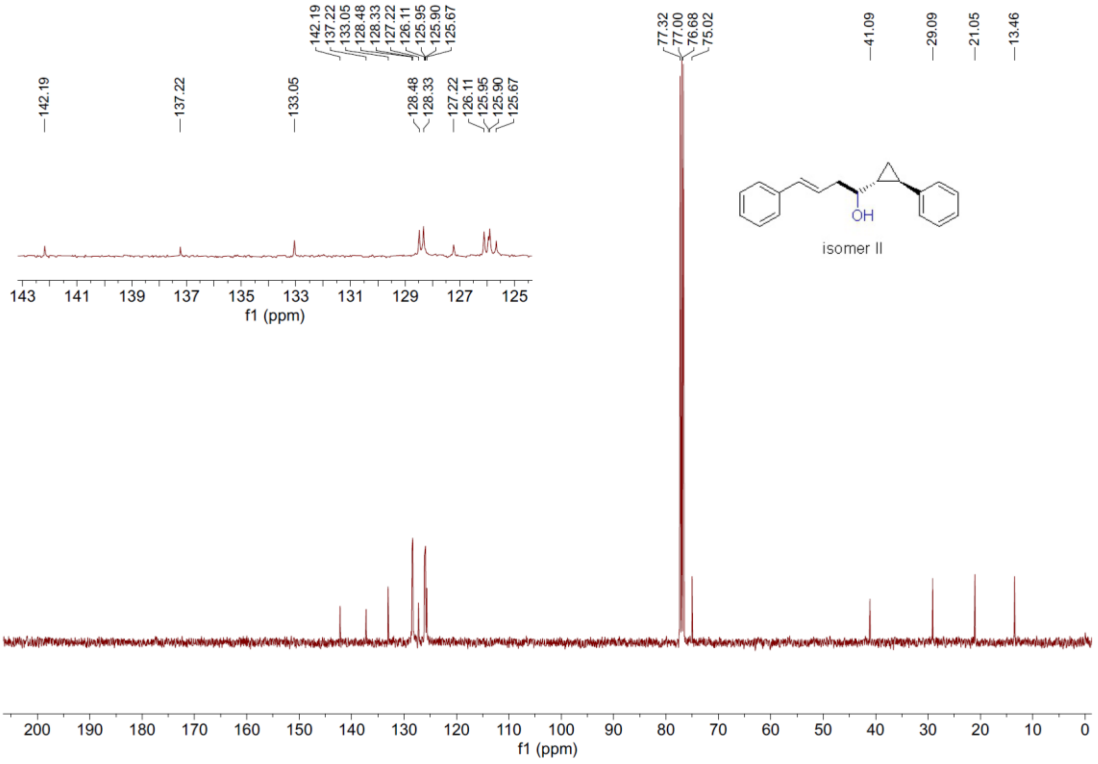

3bo ^{1}H NMR

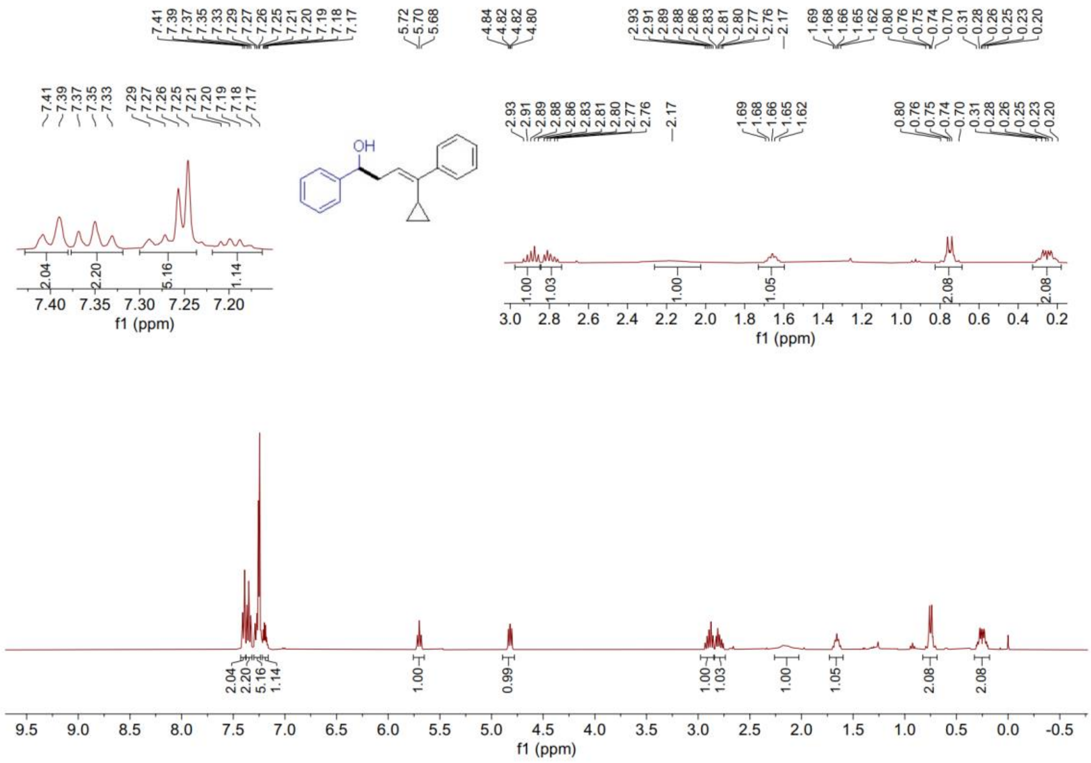

3bo ^{13}C NMR

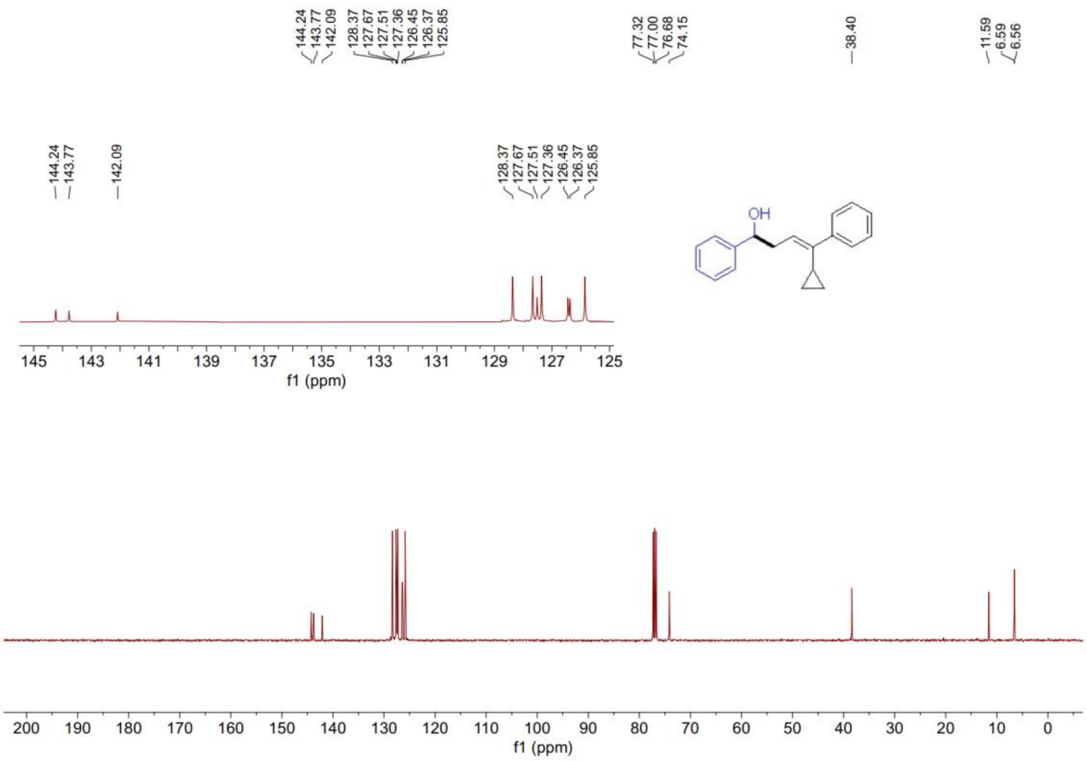

3bp ^{1}H NMR

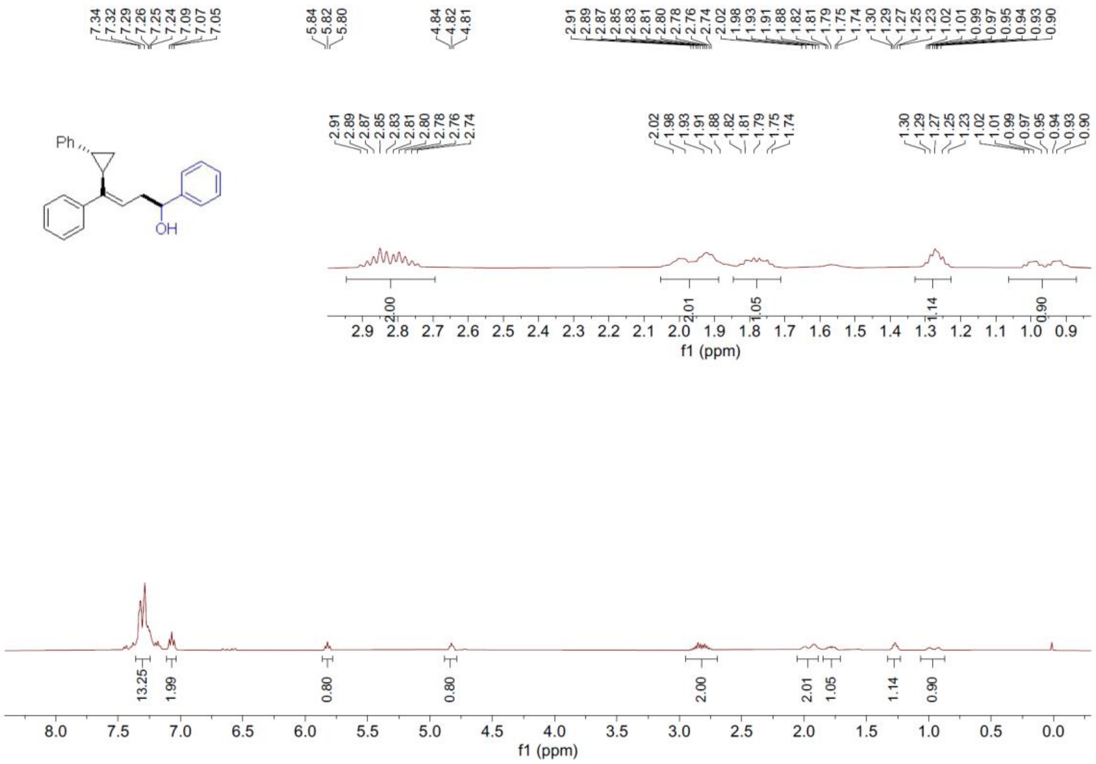

3bp ^{13}C NMR

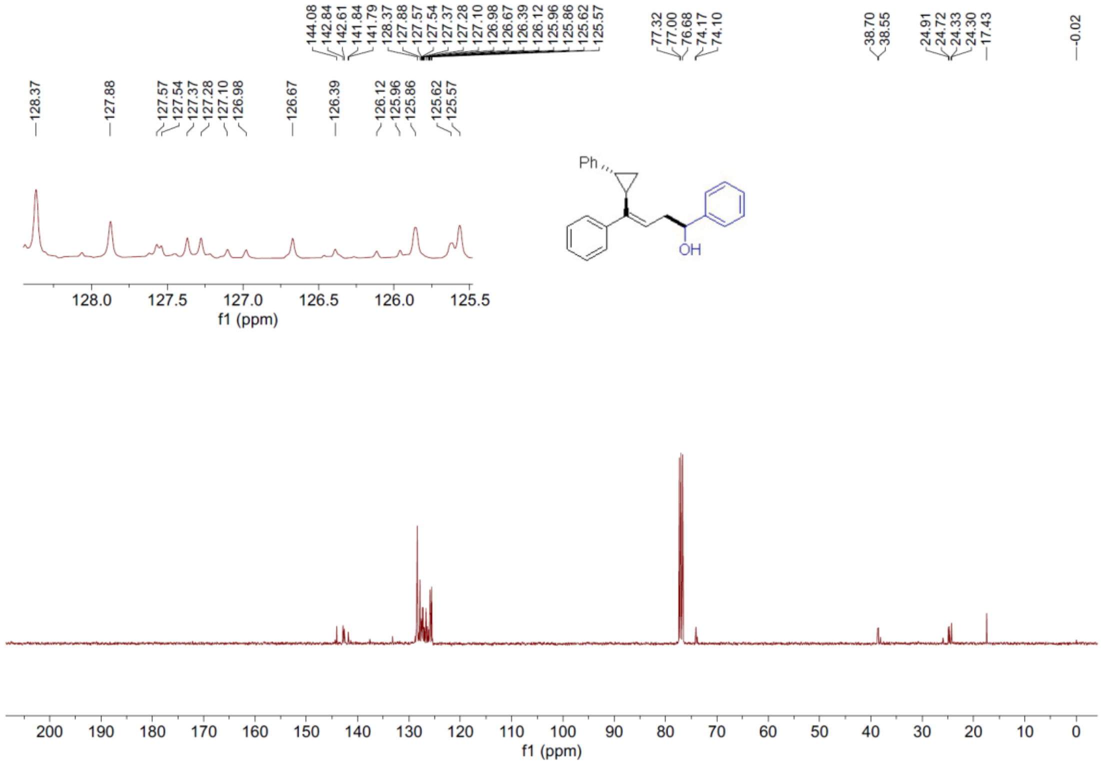

3bq ^{1}H NMR

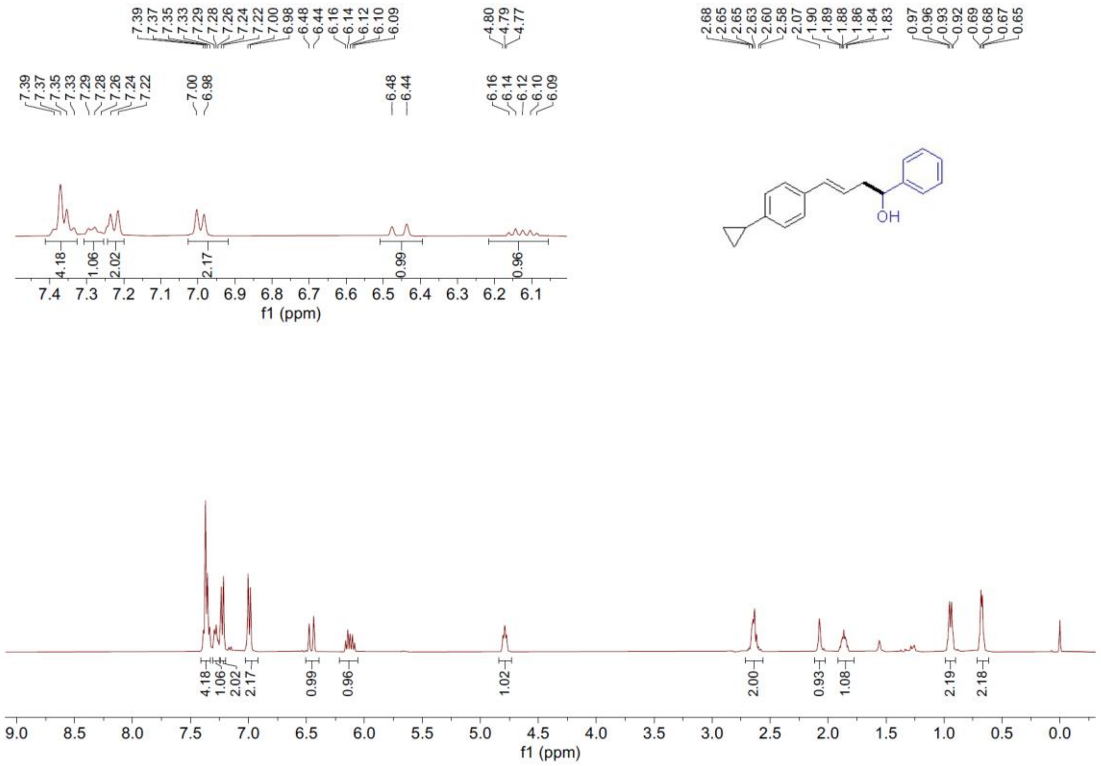

3bq ^{13}C NMR

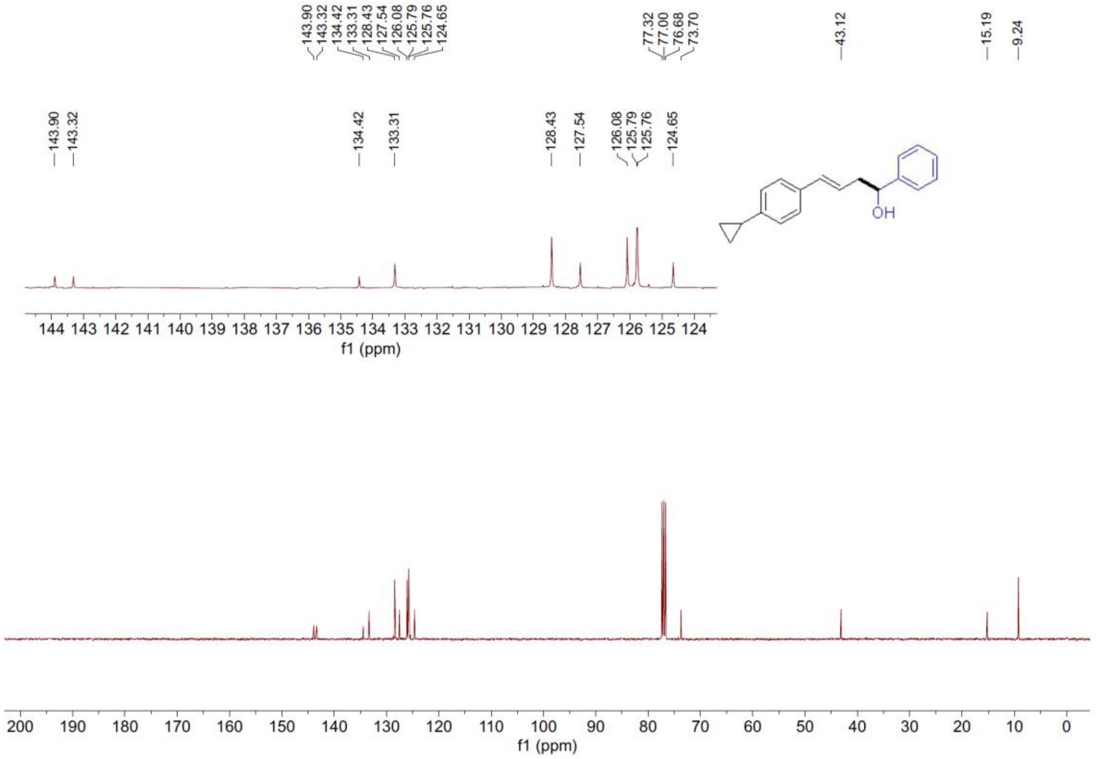

3br ^{1}H NMR

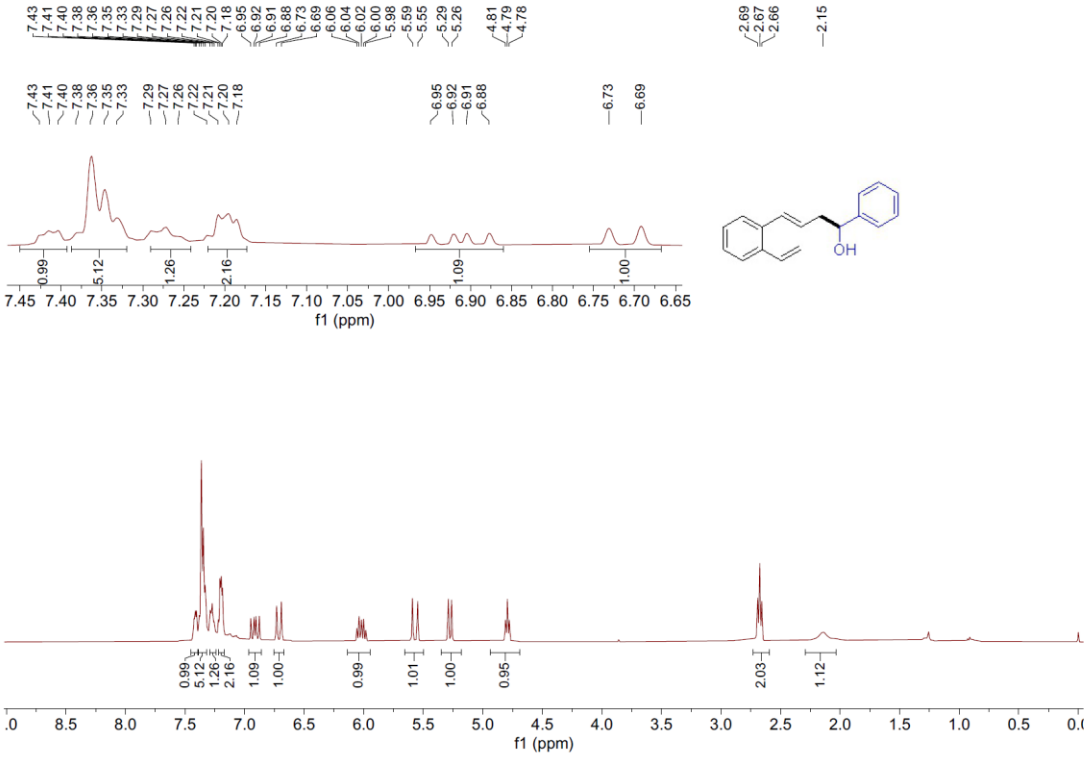

3br ^{13}C NMR

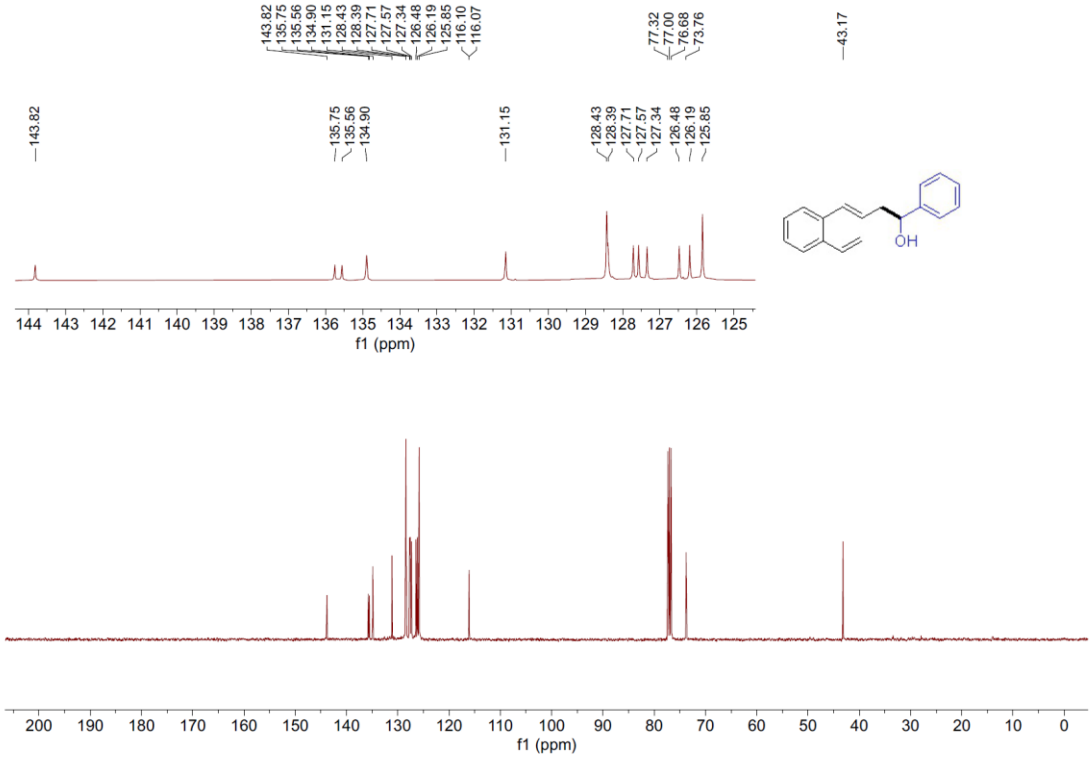

5a ^{1}H NMR

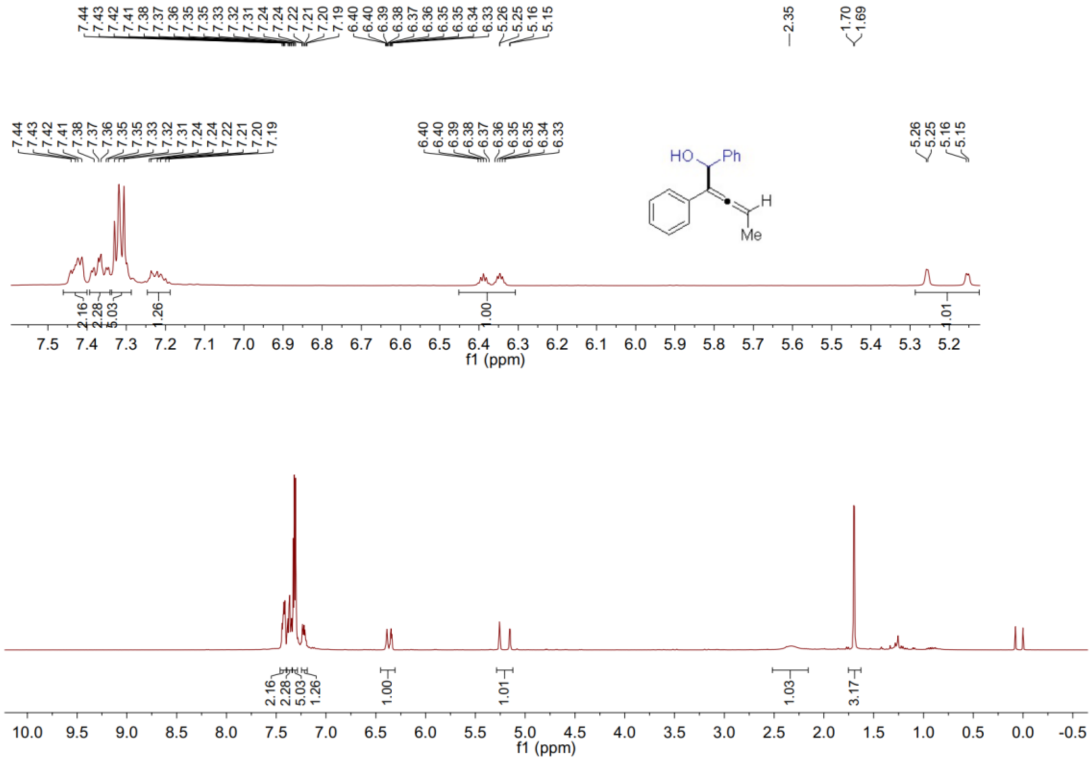

5a ^{13}C NMR

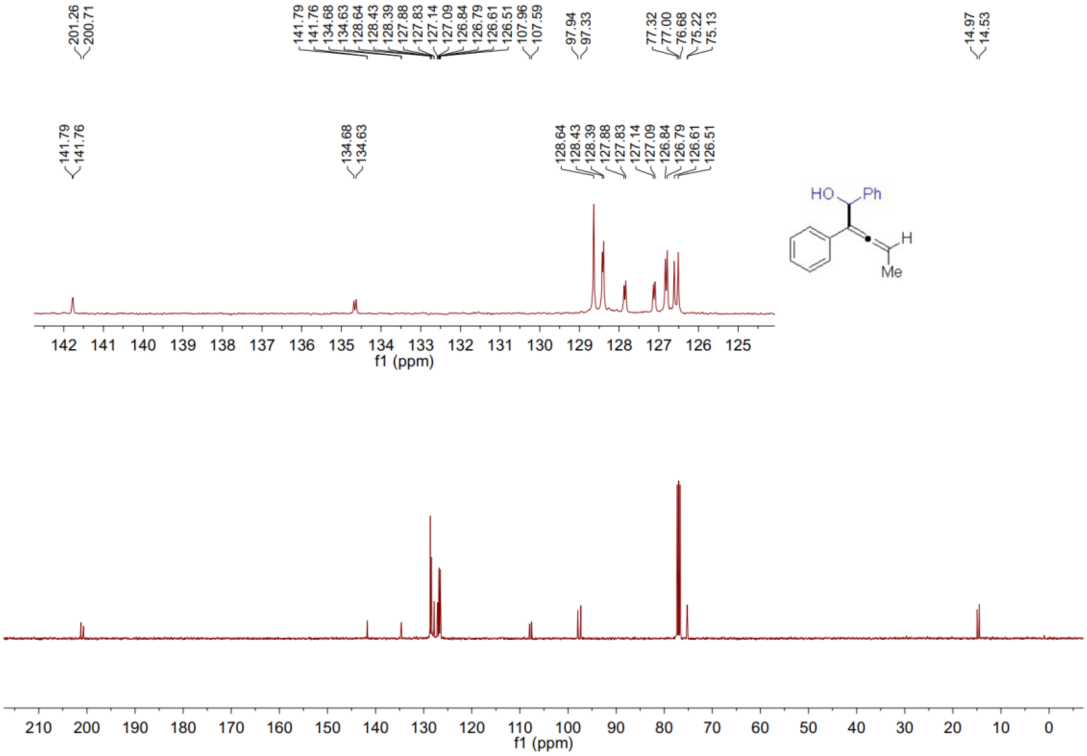

5b ^{1}H NMR

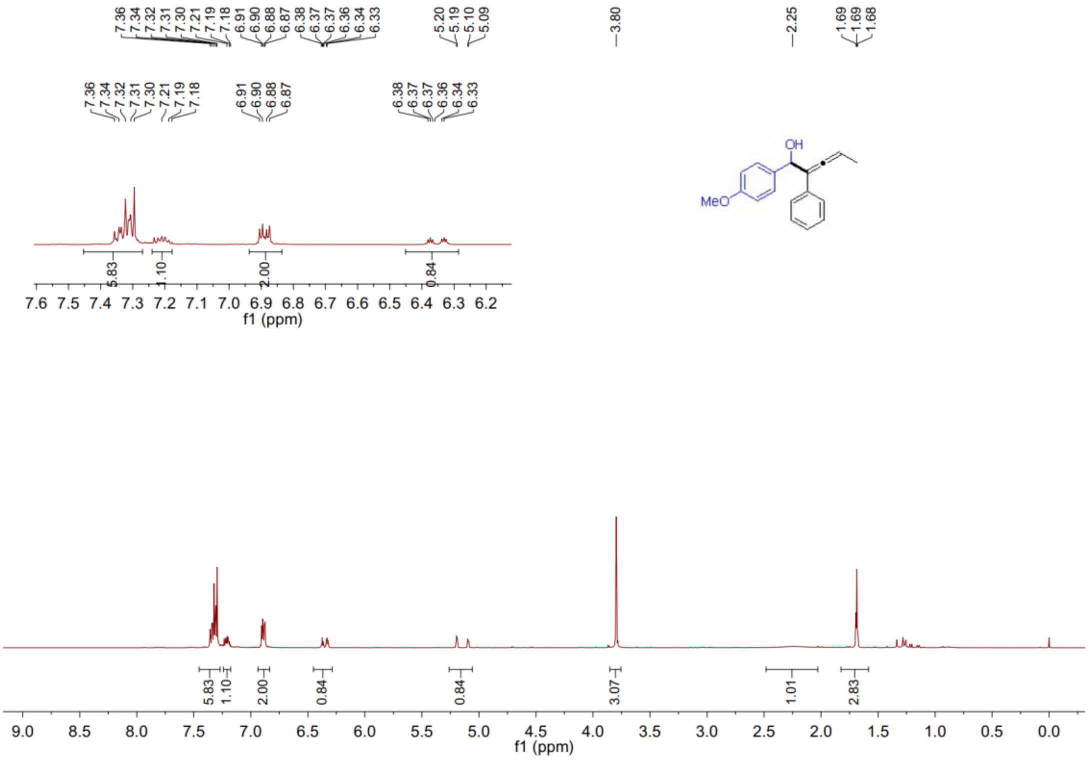

5b ^{13}C NMR

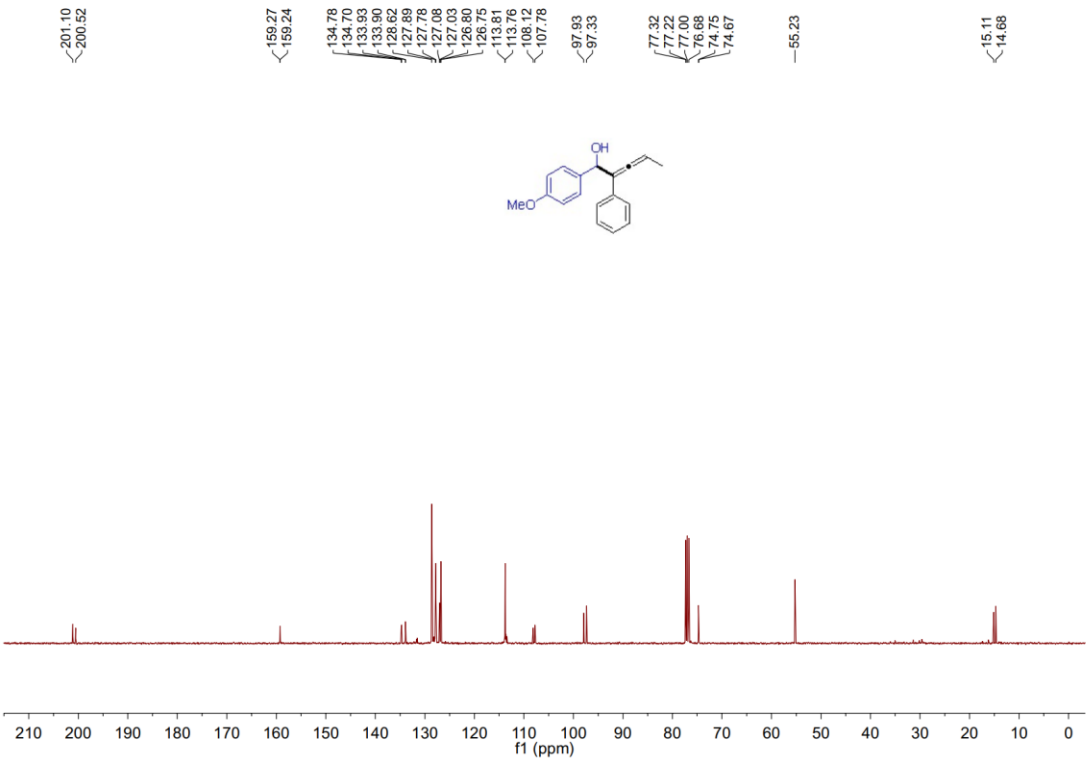

5c ^{1}H NMR

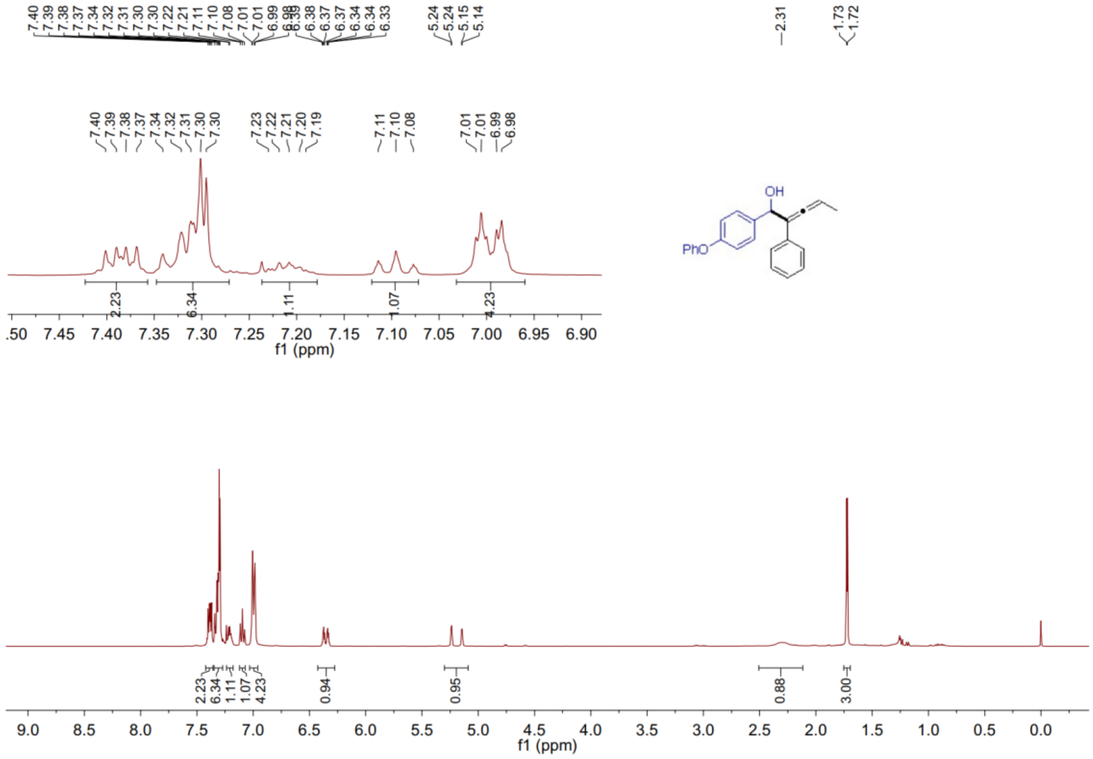

5c ^{13}C NMR

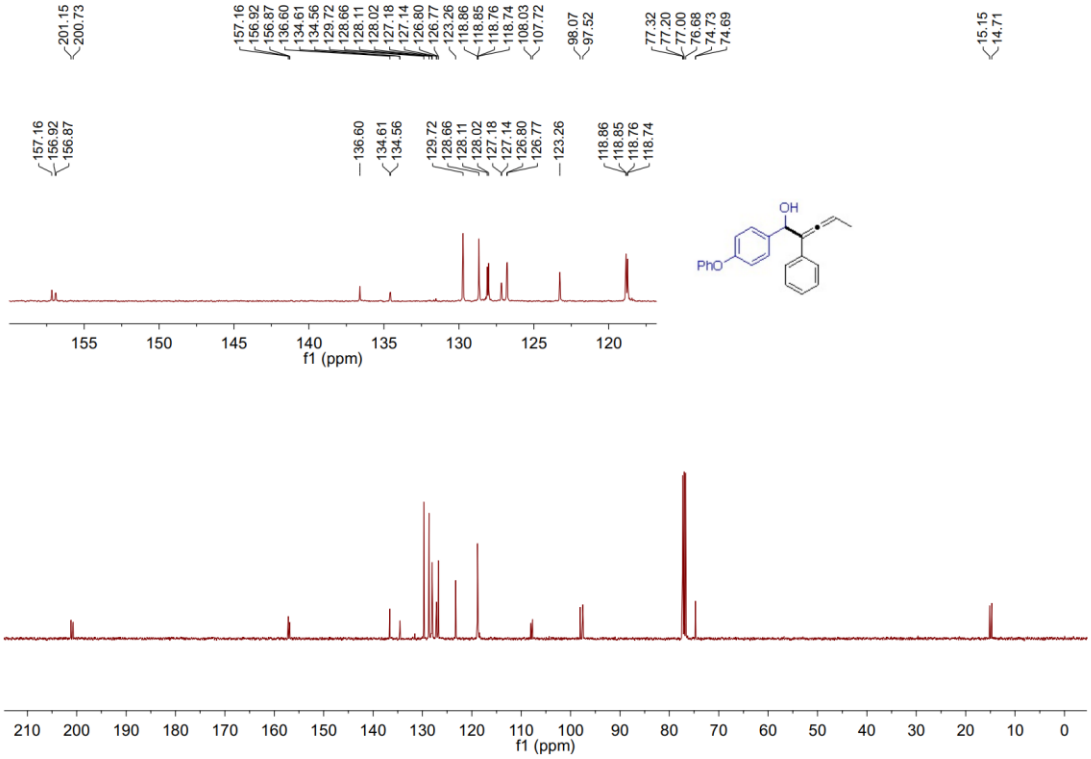

5d ^{1}H NMR

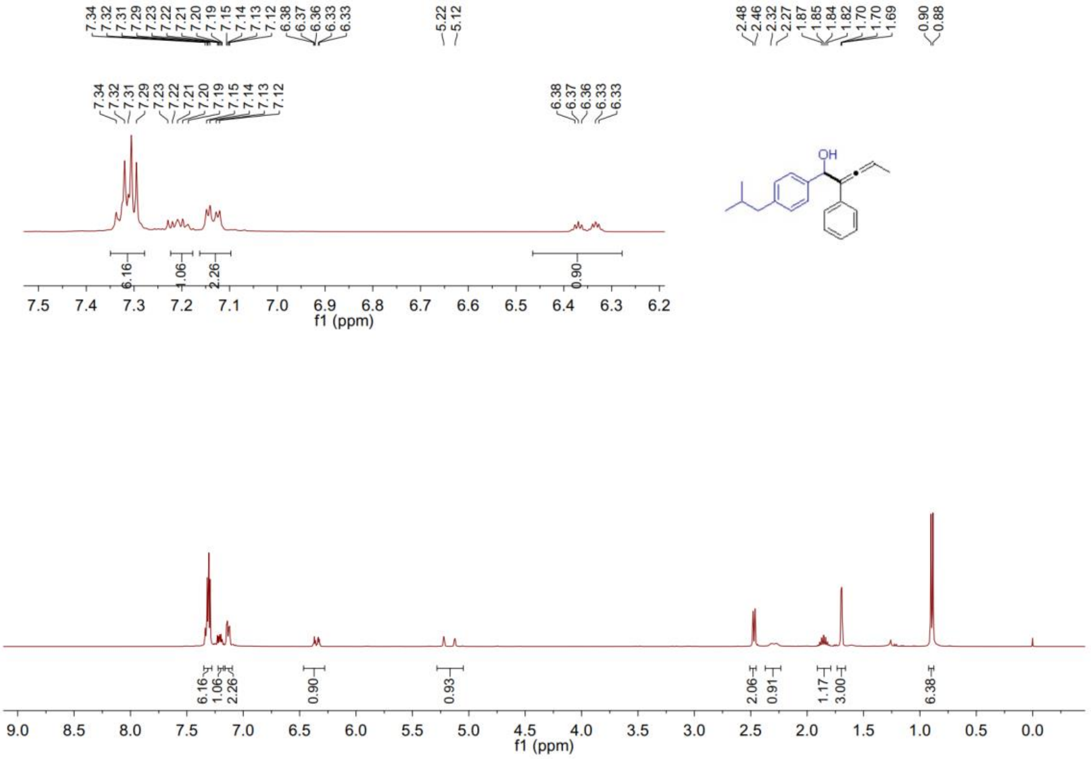

5d ^{13}C NMR

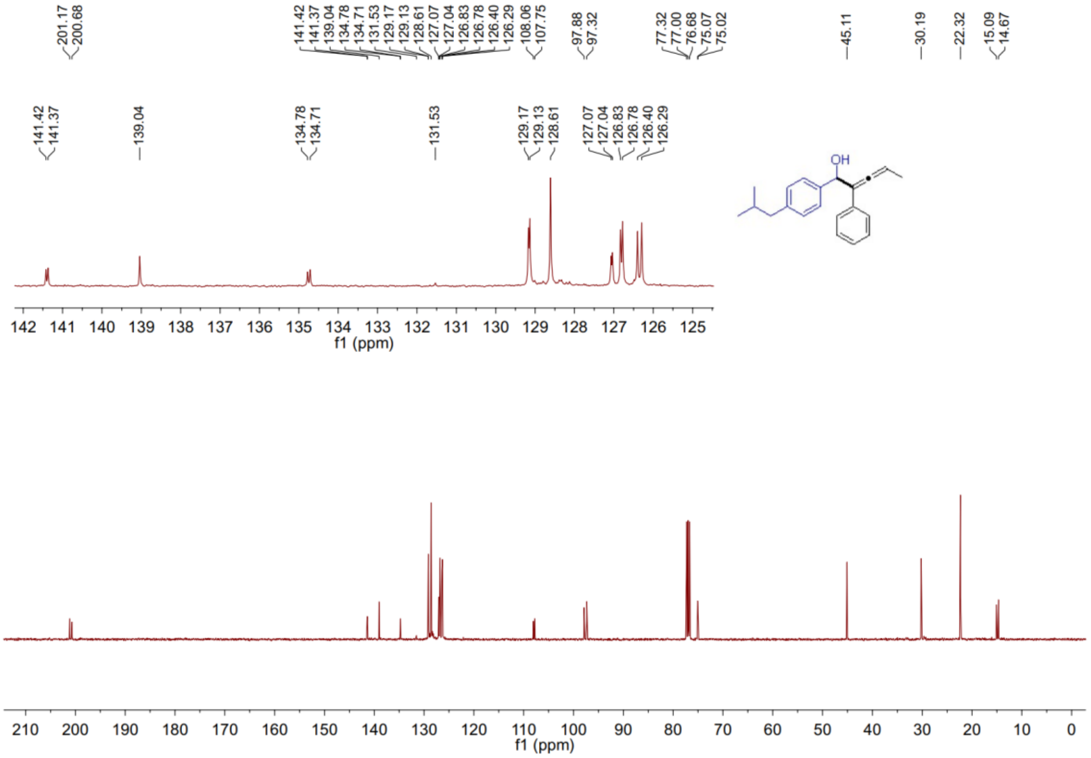

5e ^{1}H NMR

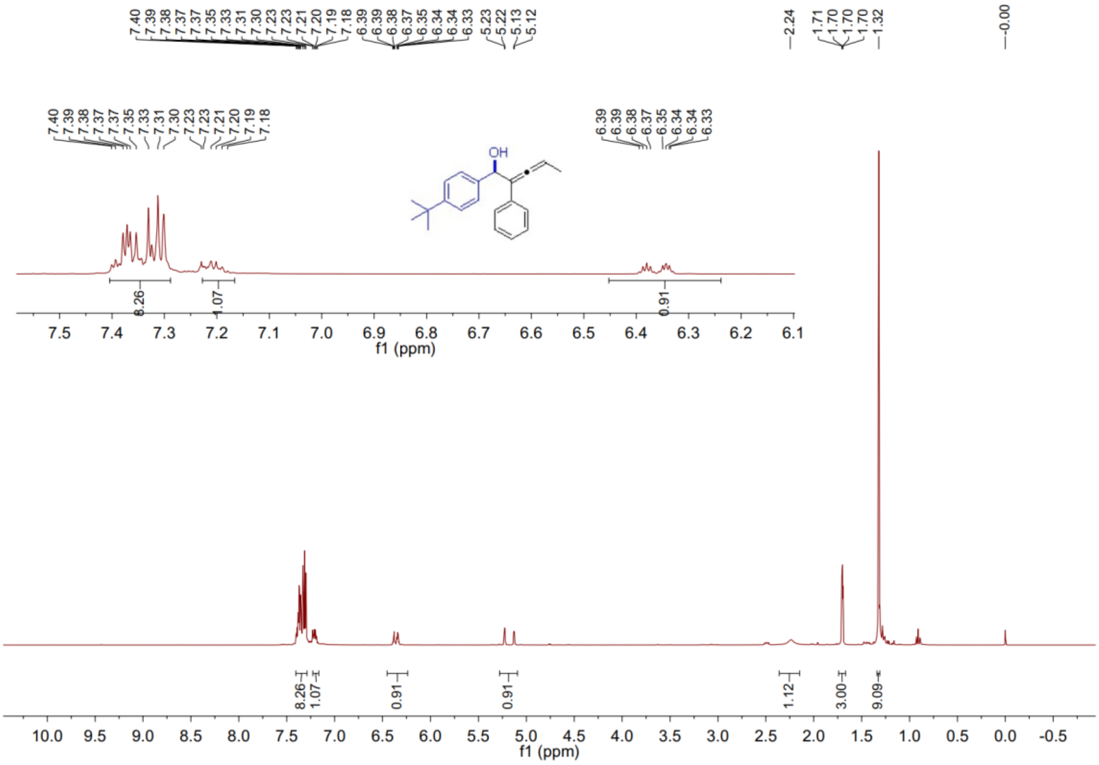

5e ^{13}C NMR

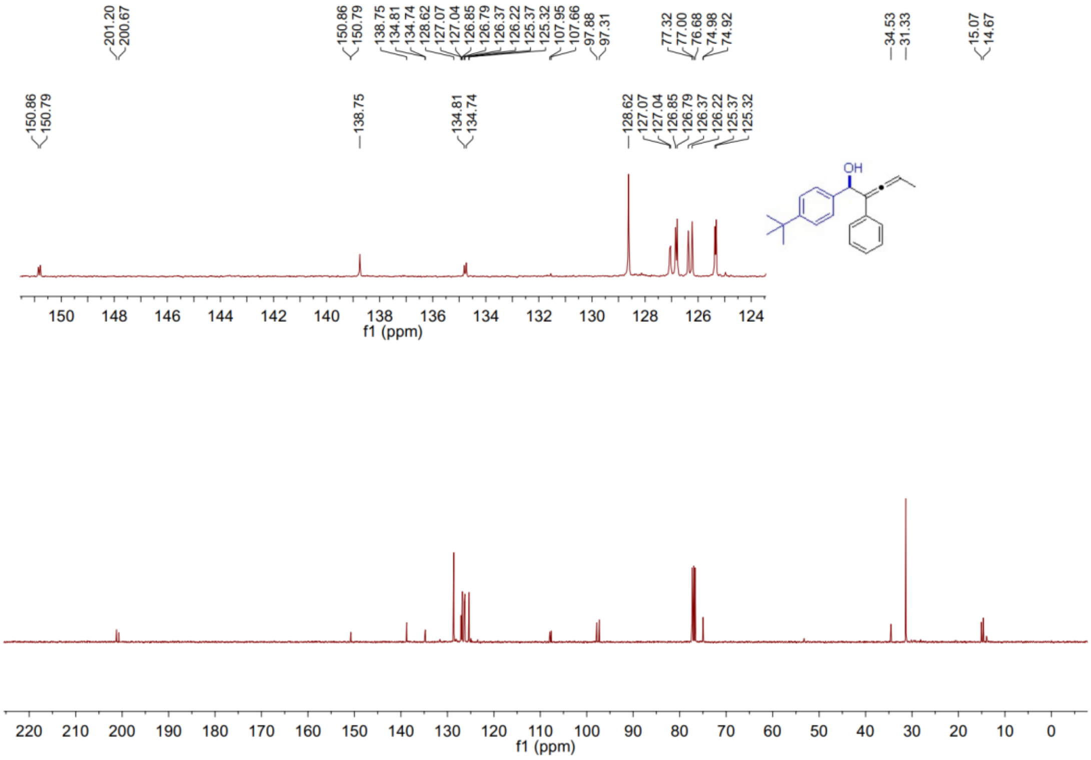

5f ^{1}H NMR

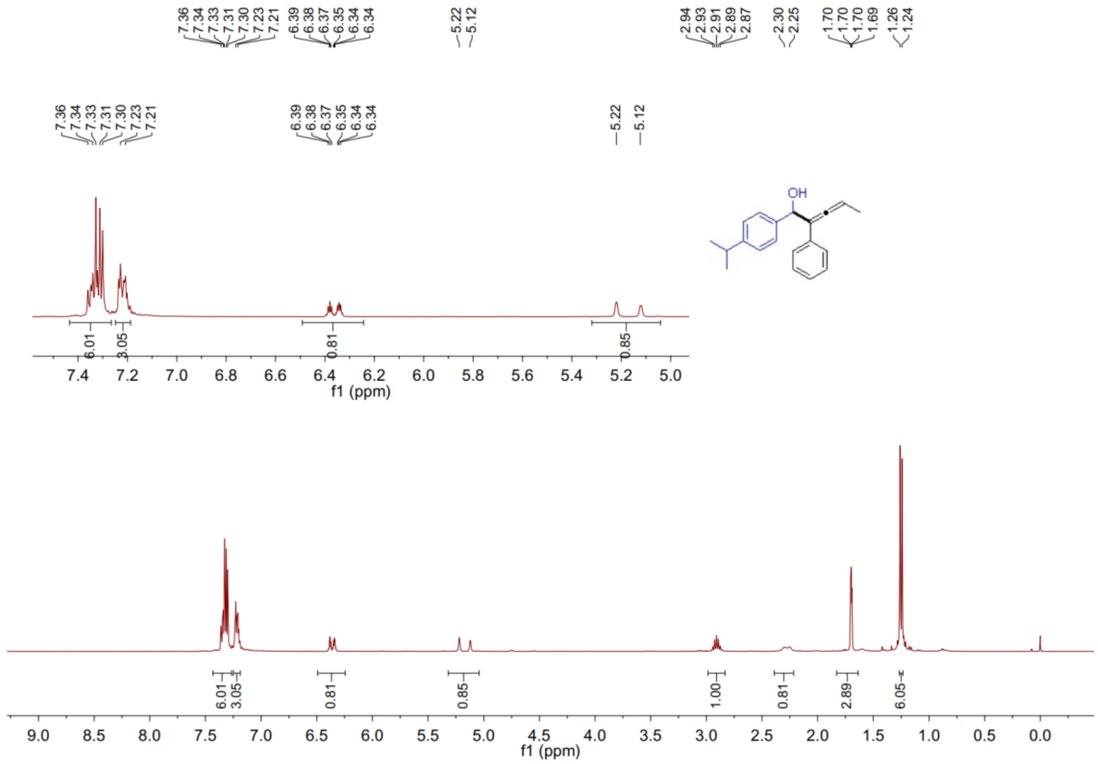

5f ^{13}C NMR

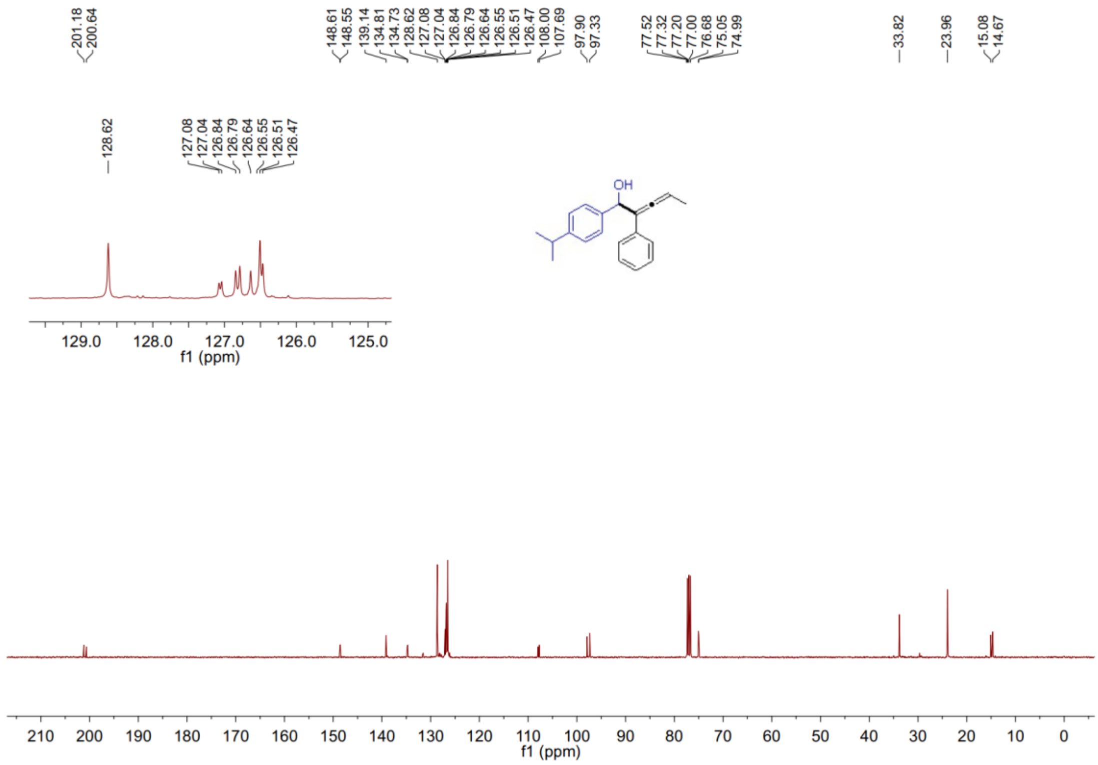

5g ^{1}H NMR

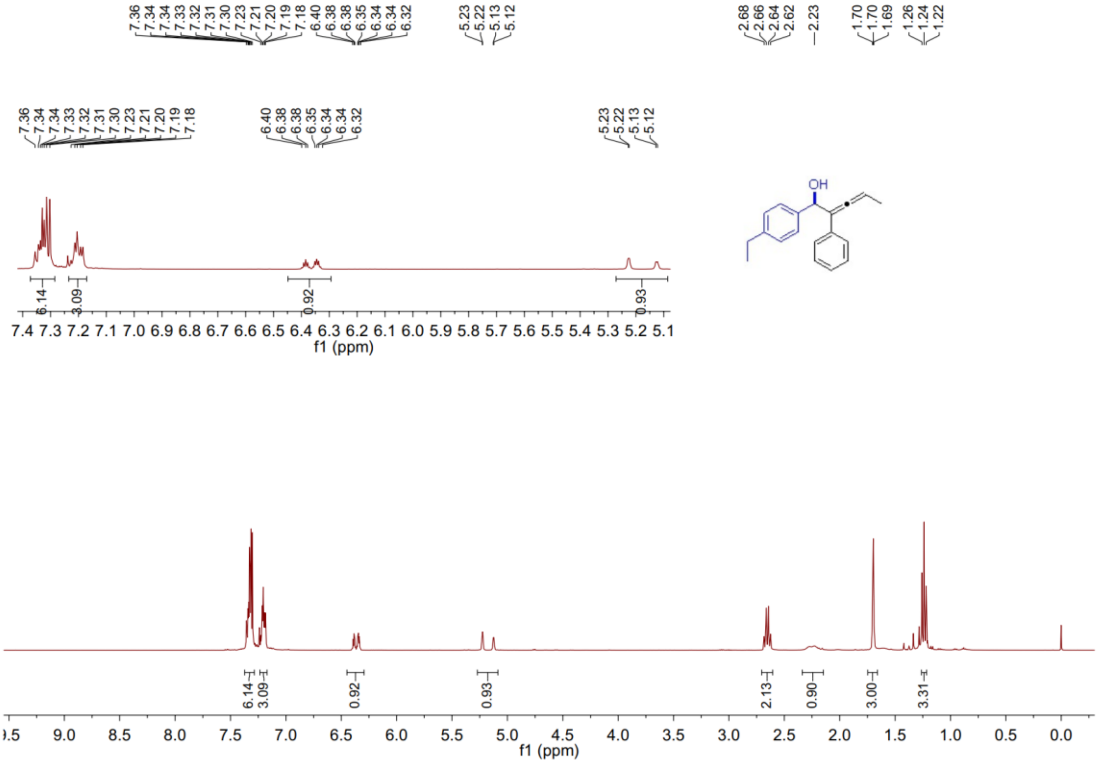

5g ^{13}C NMR

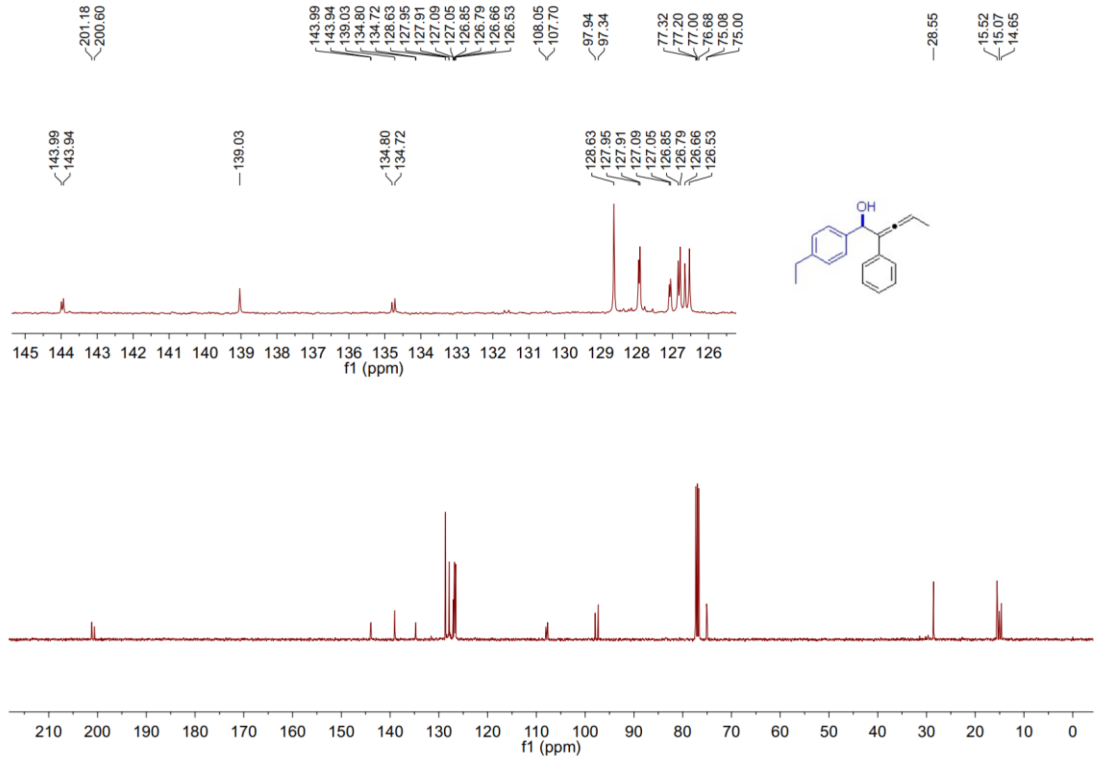

5h ^{1}H NMR

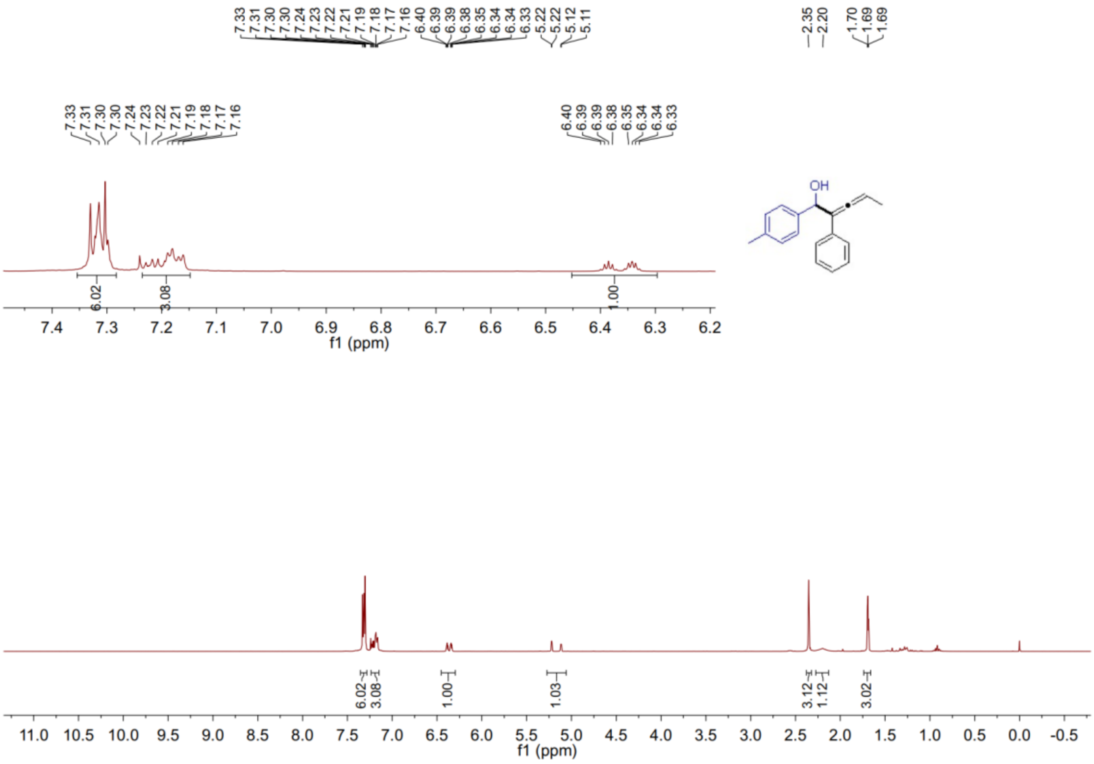

5h ^{13}C NMR

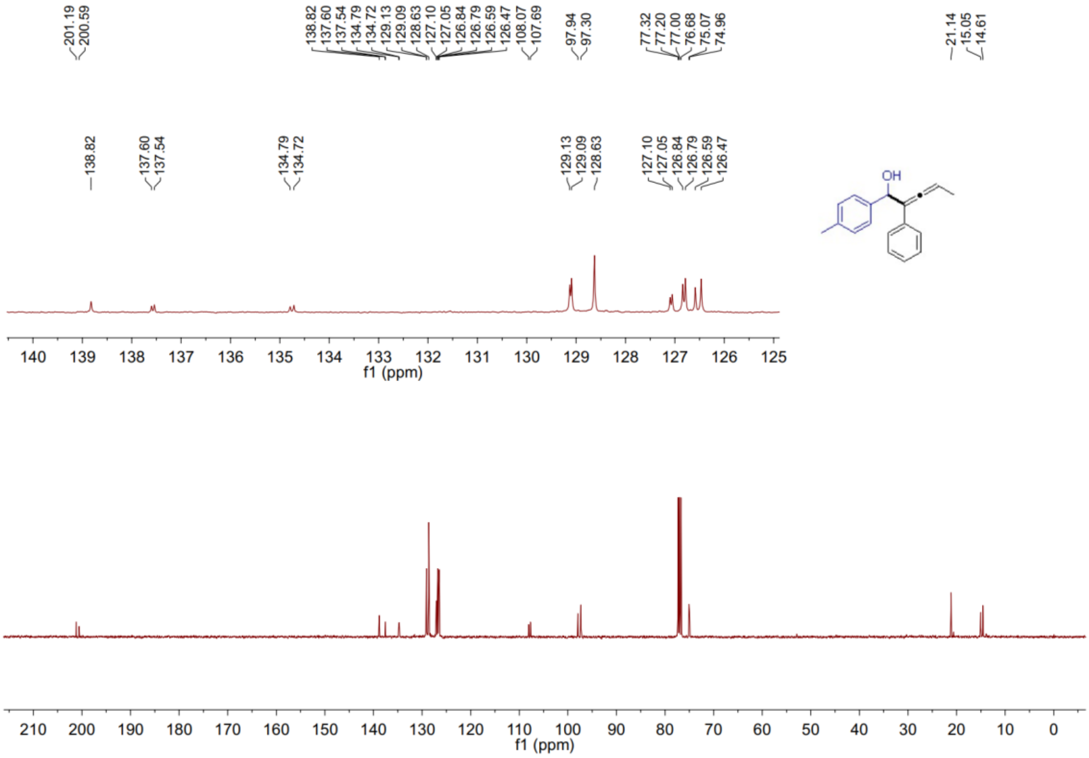

5i ^{1}H NMR

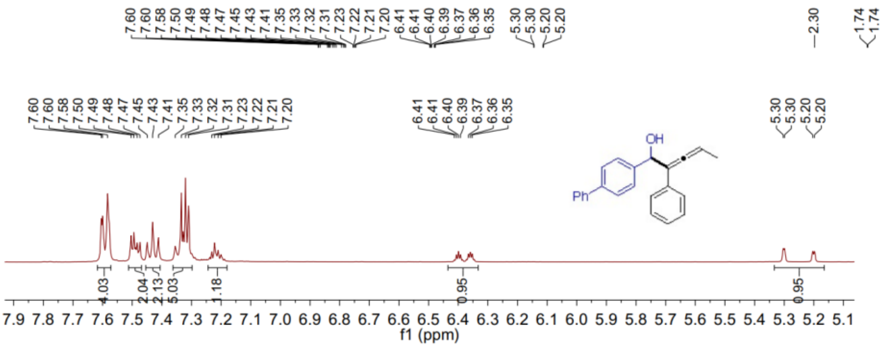

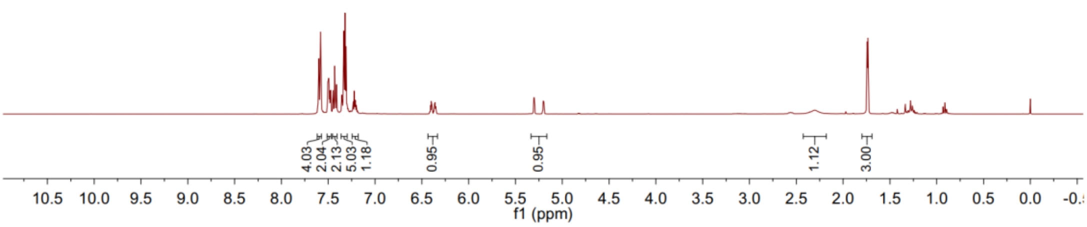

5i ^{13}C NMR

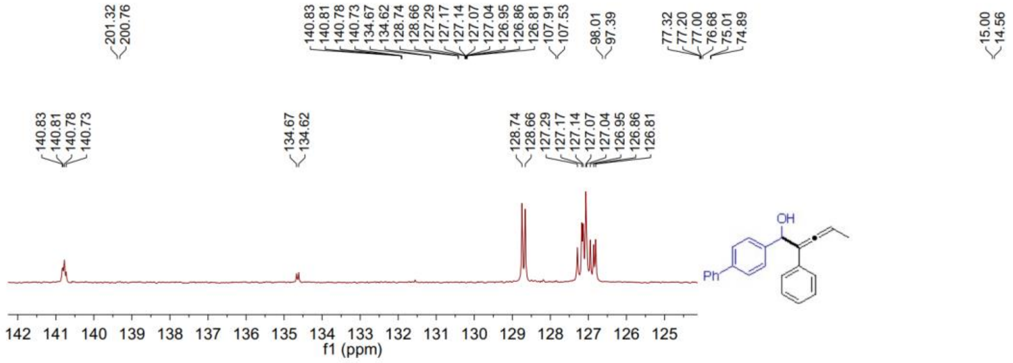

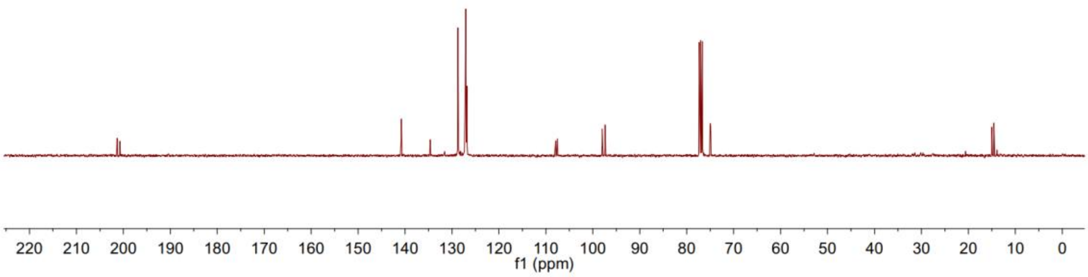

5j ^{1}H NMR

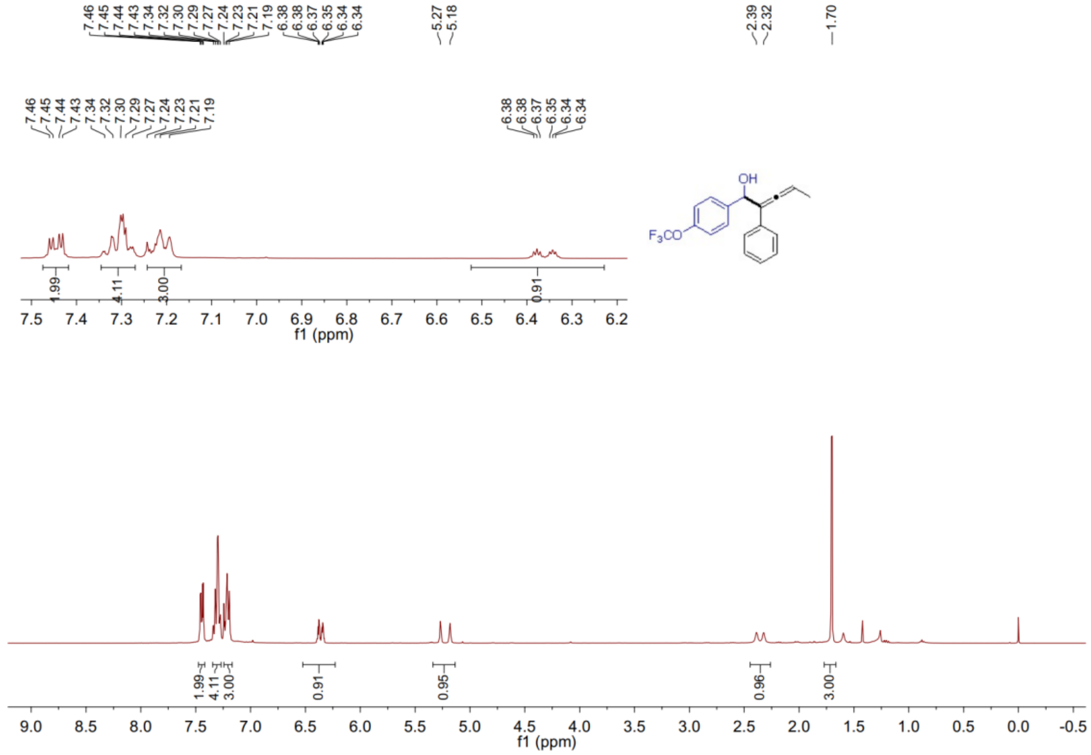

5j ^{13}C NMR

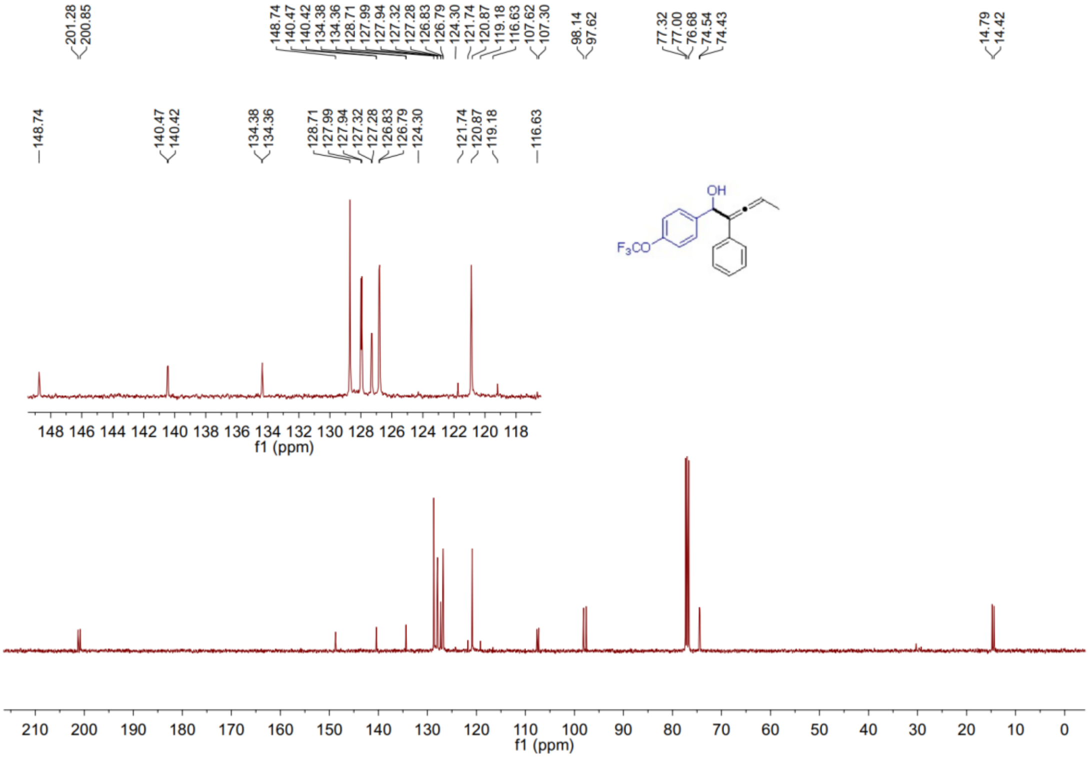

5k ¹H NMR

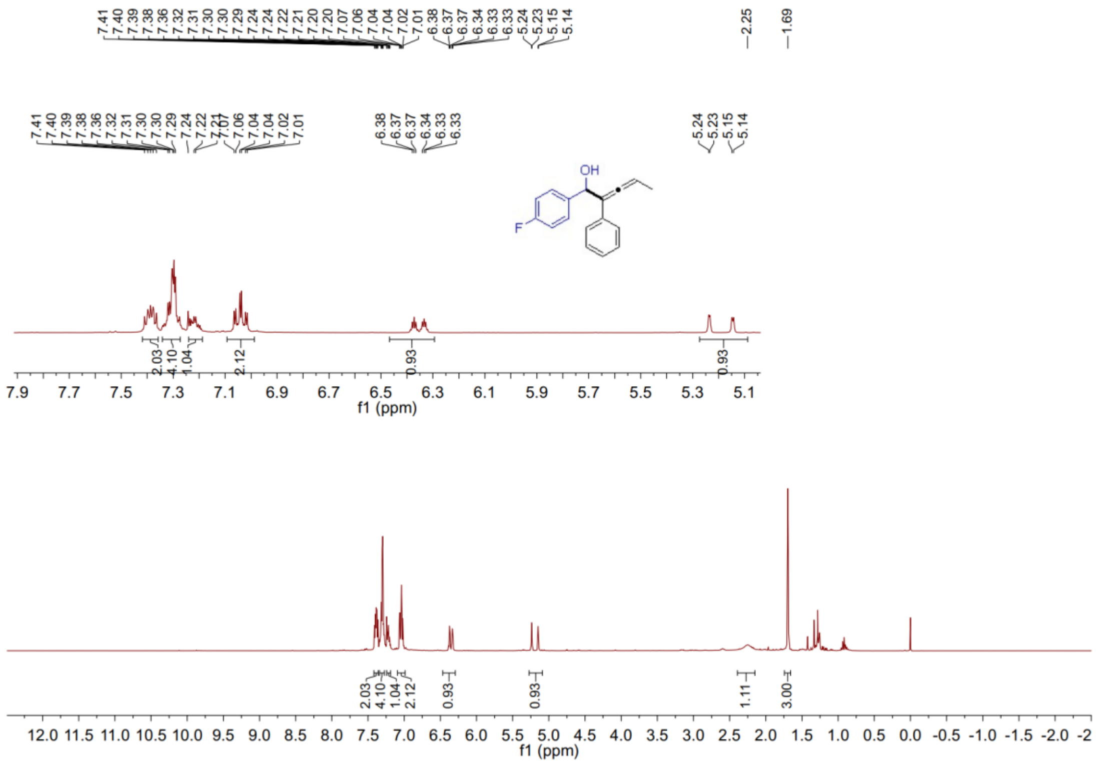

5k ¹³C NMR

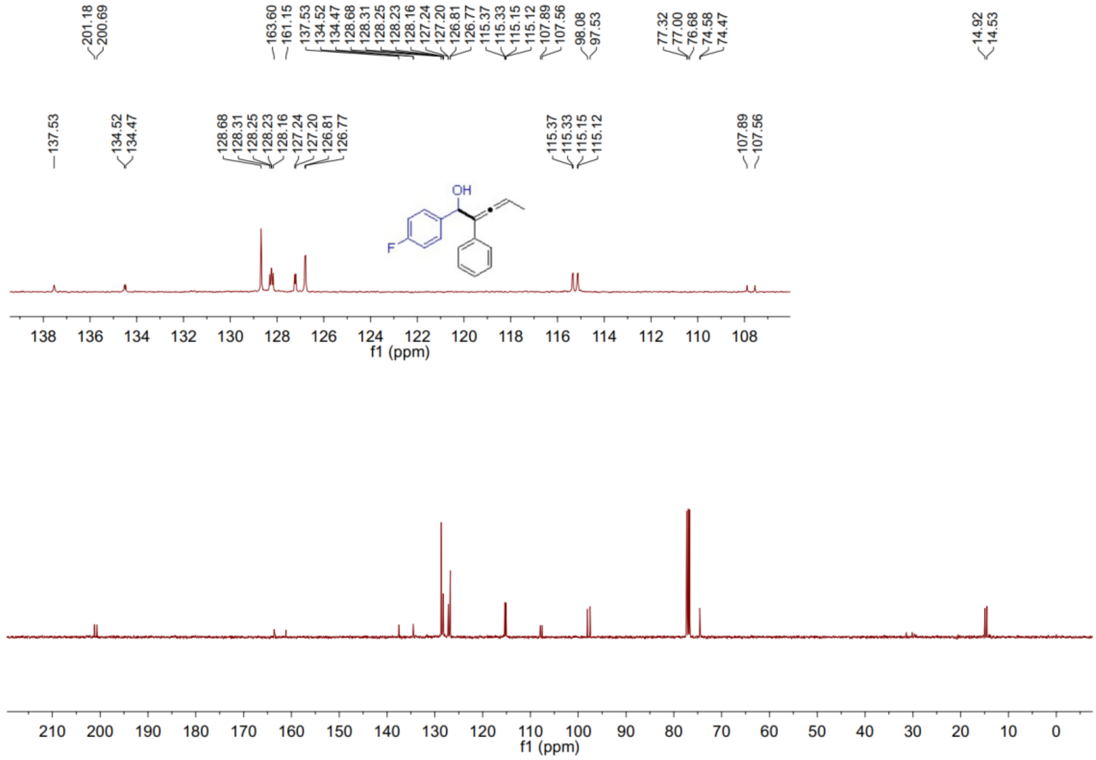

5l ^{1}H NMR

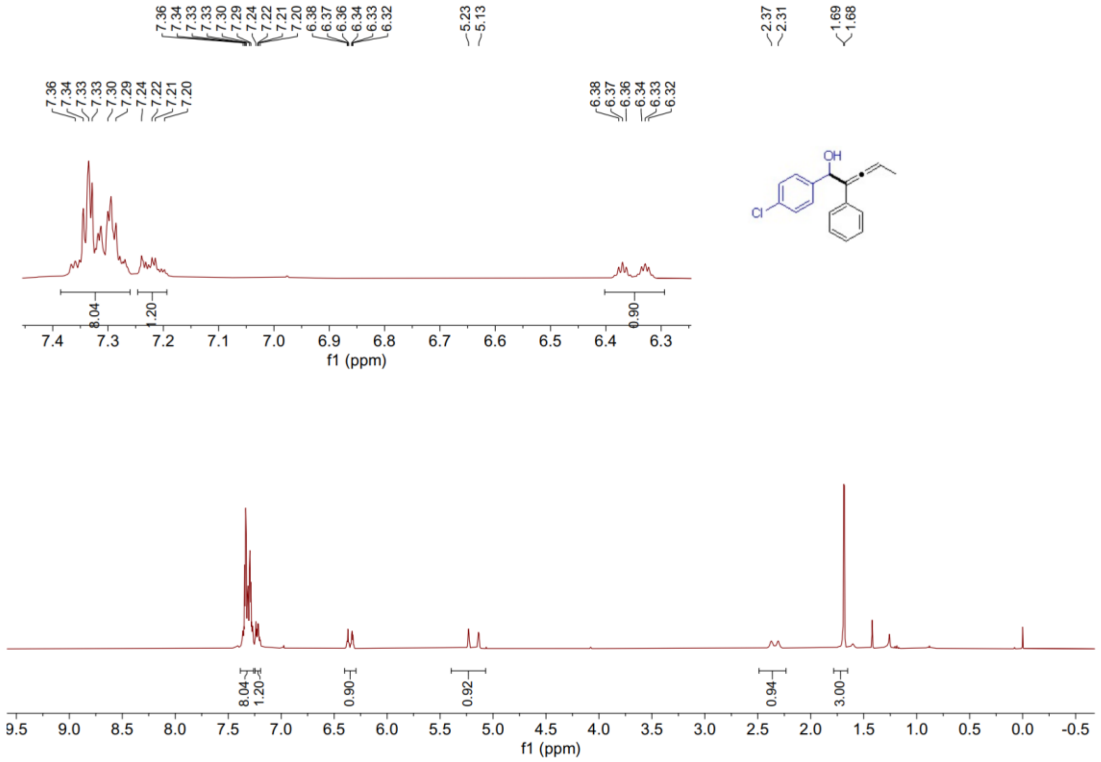

5l ^{13}C NMR

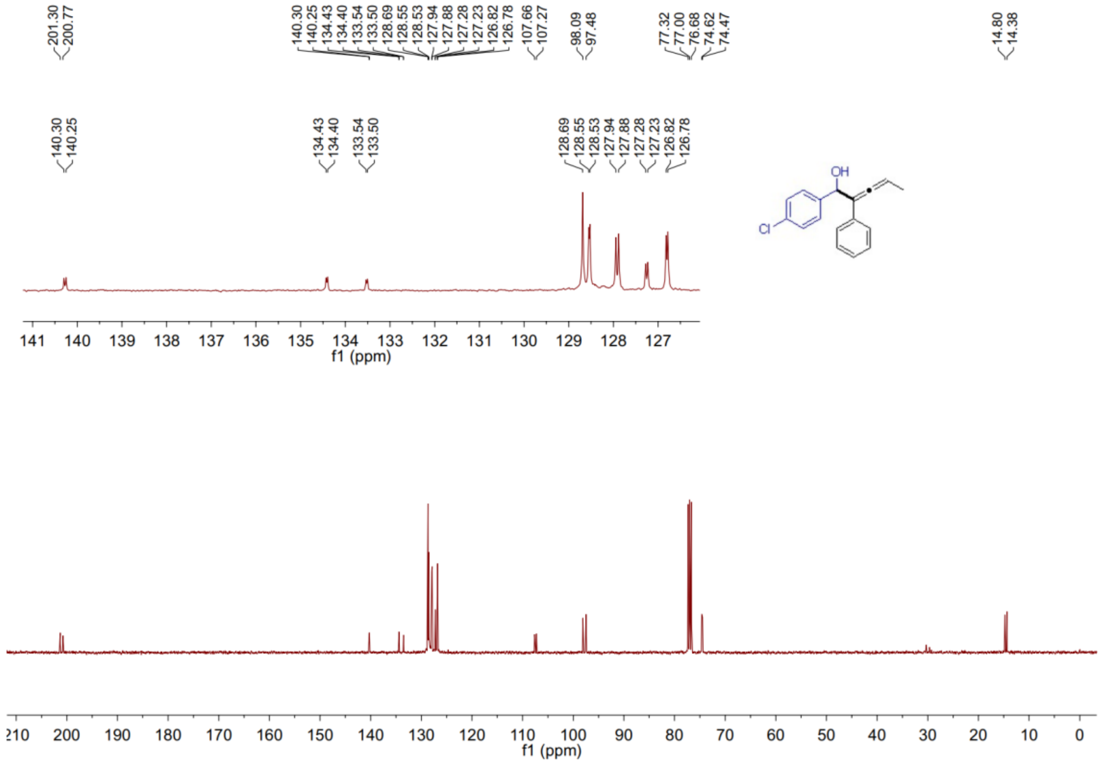

5m ^{1}H NMR

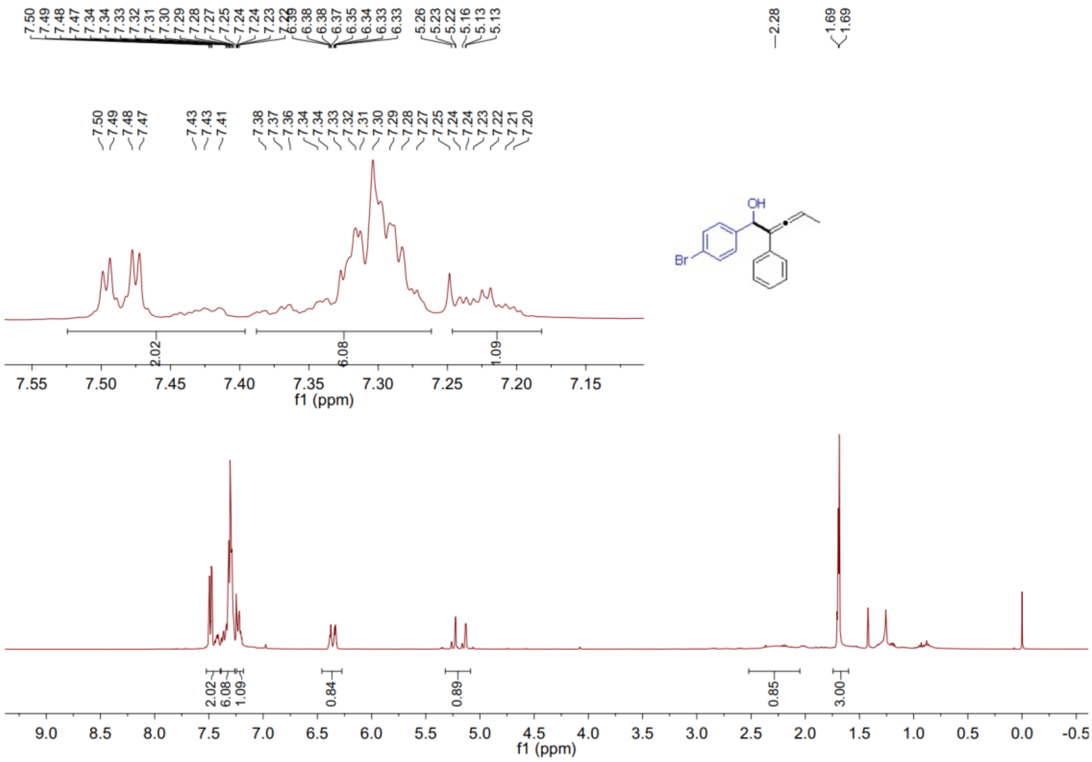

5m ^{13}C NMR

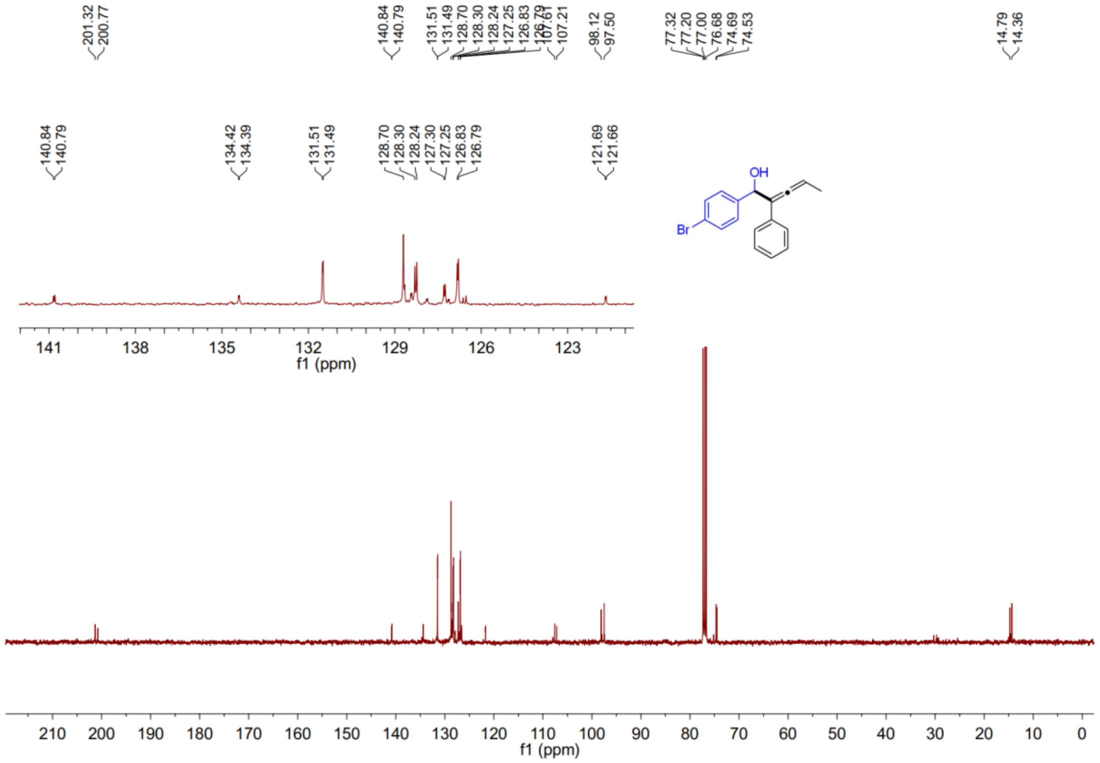

5n ^{1}H NMR

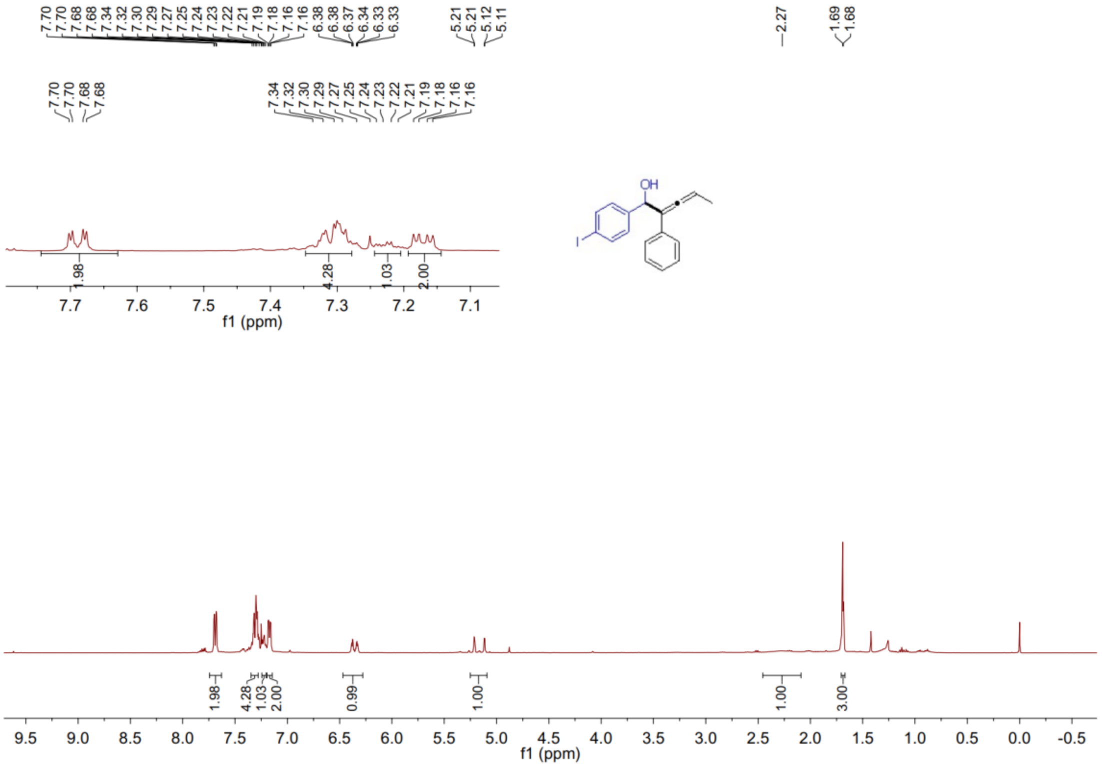

5n ^{13}C NMR

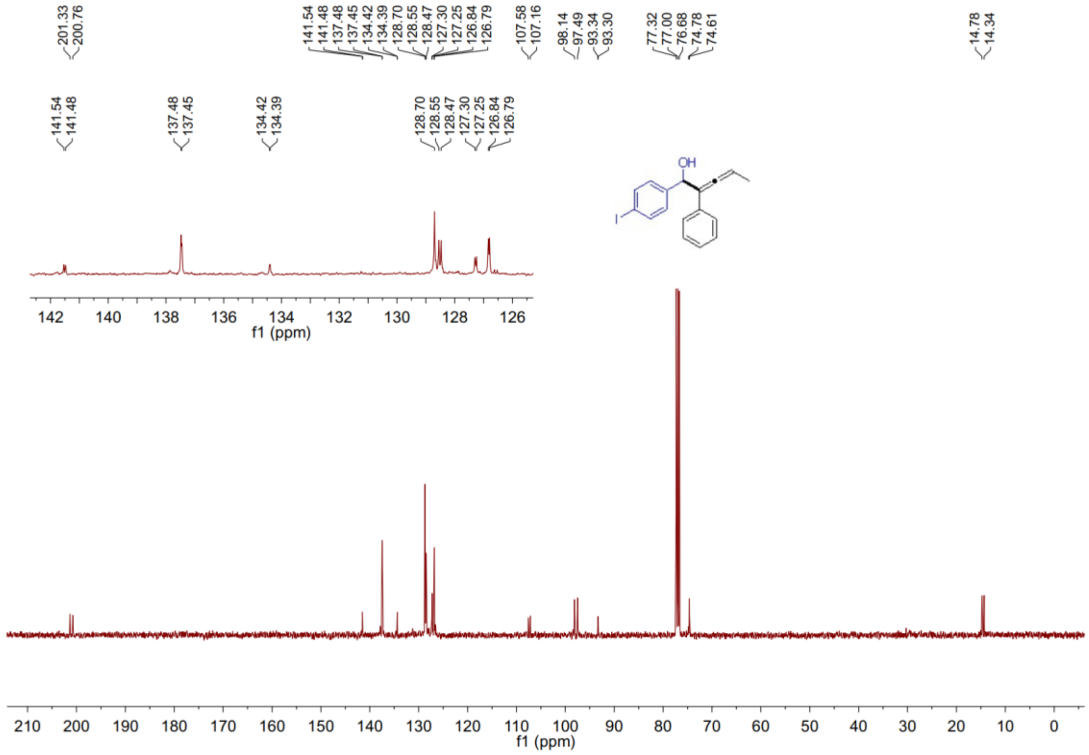

5o ^{1}H NMR

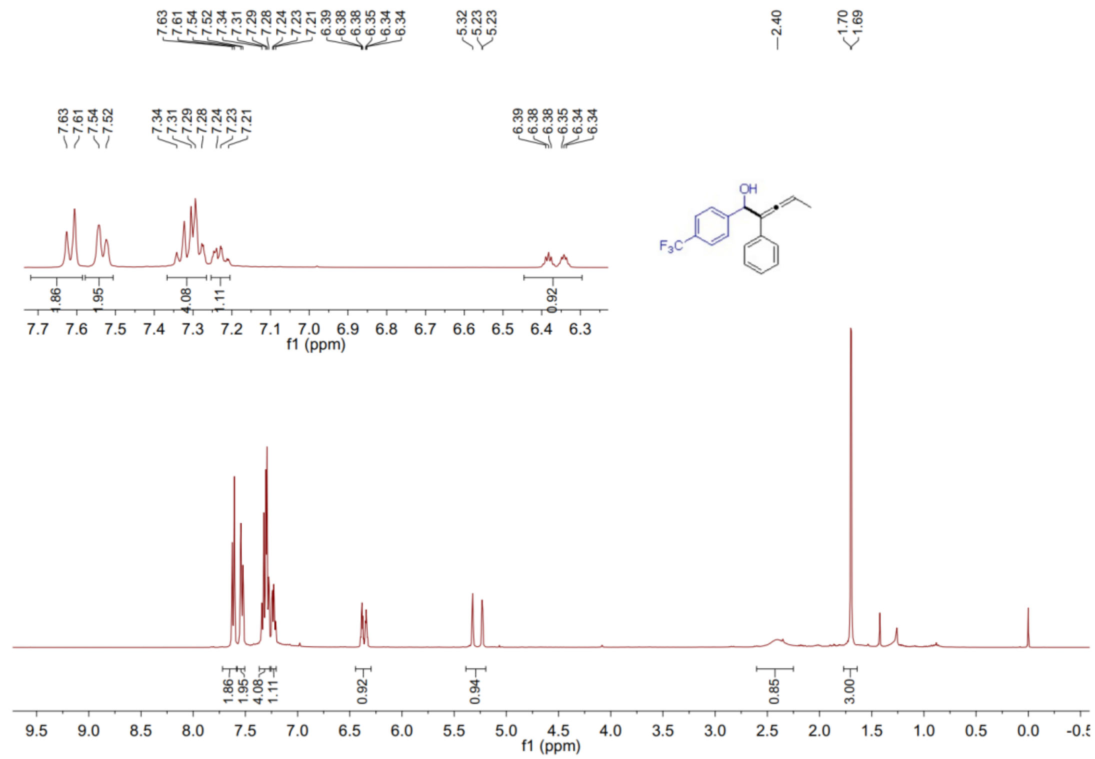

5o ^{13}C NMR

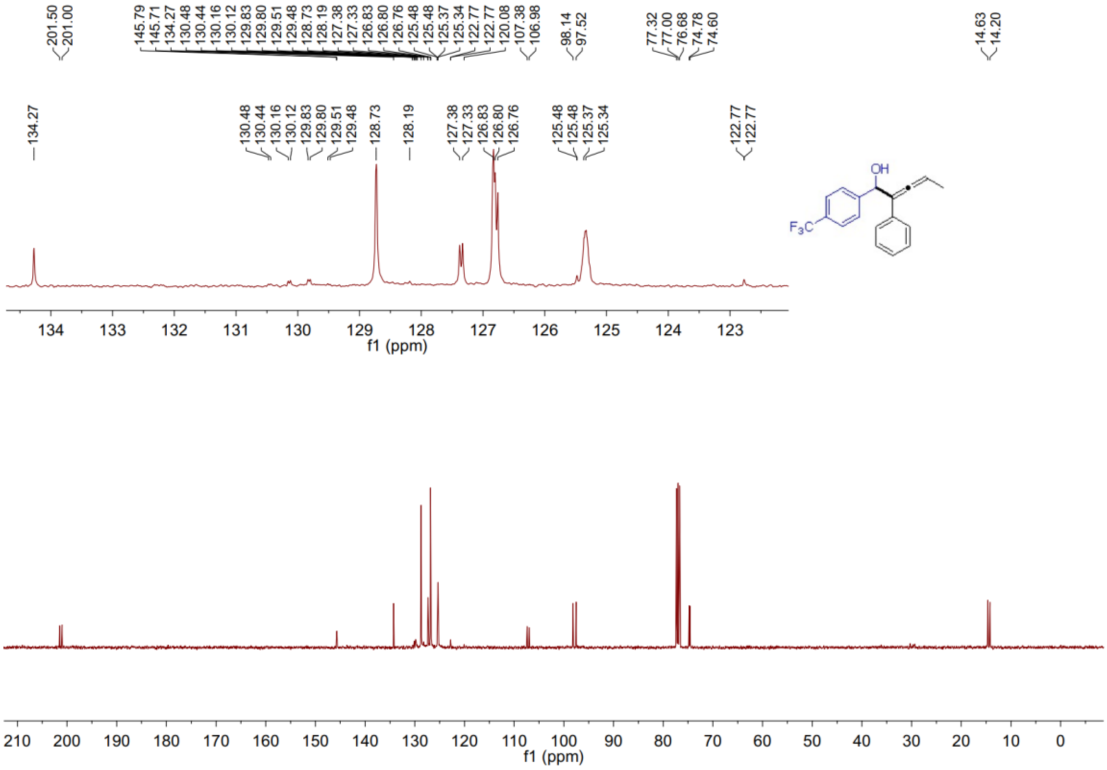

5p ^{1}H NMR

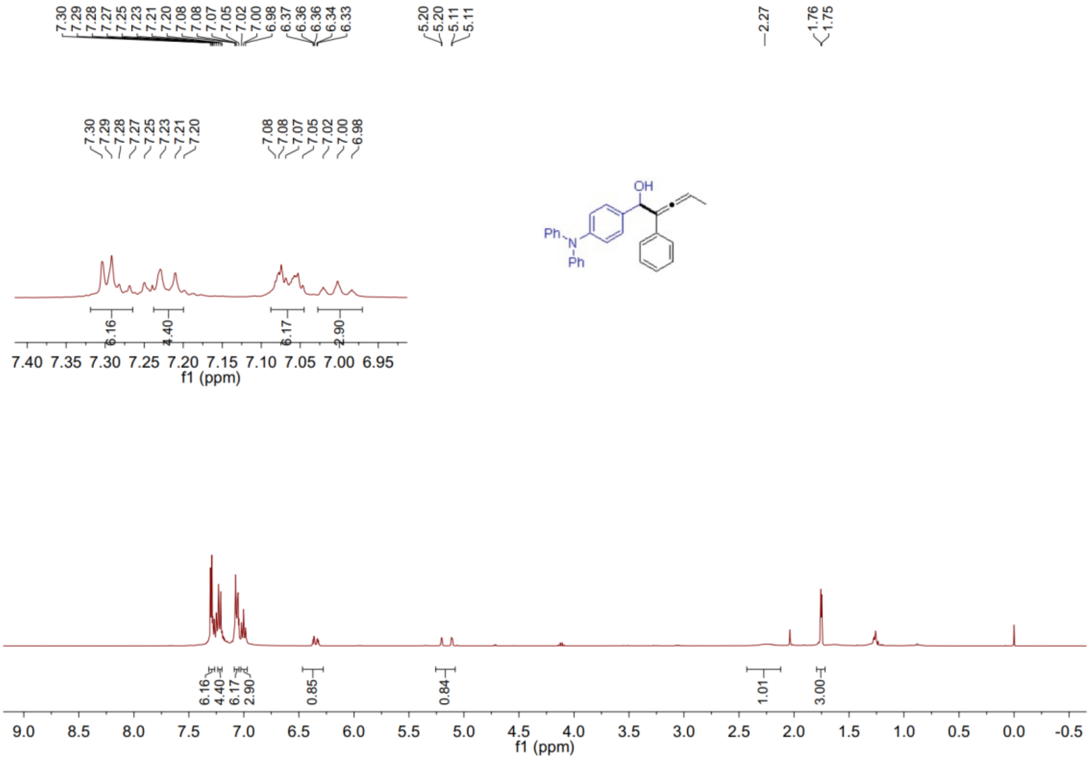

5p ^{13}C NMR

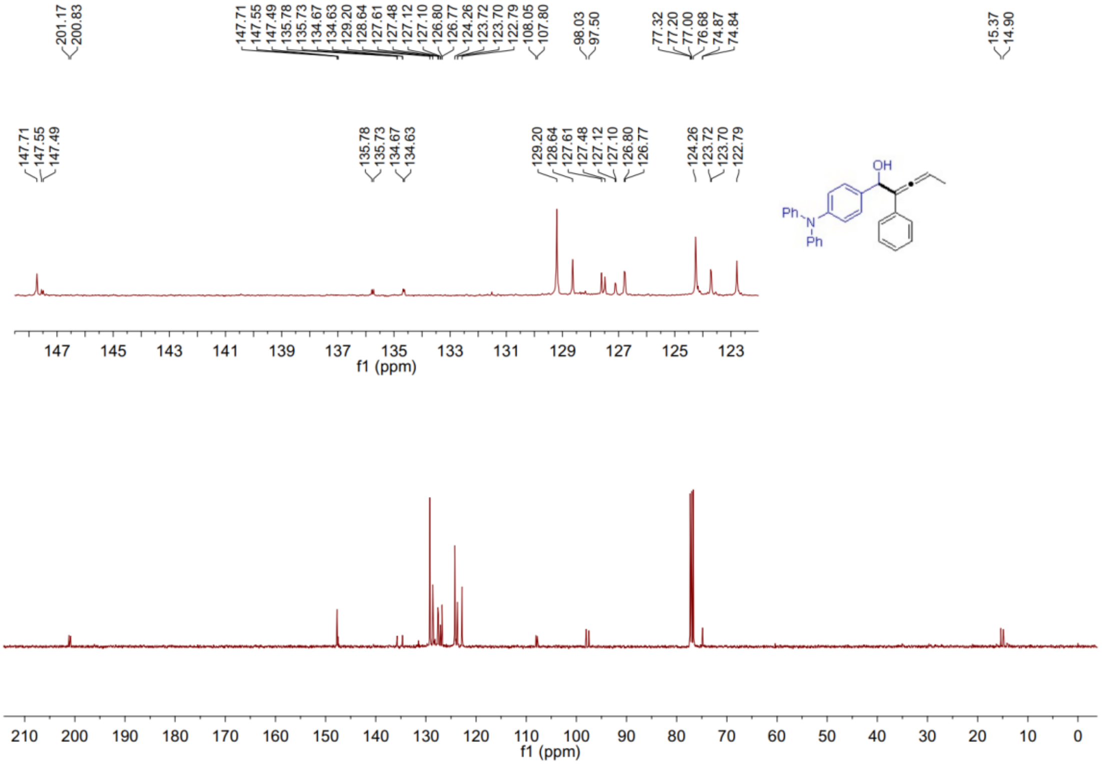

5q ^{1}H NMR

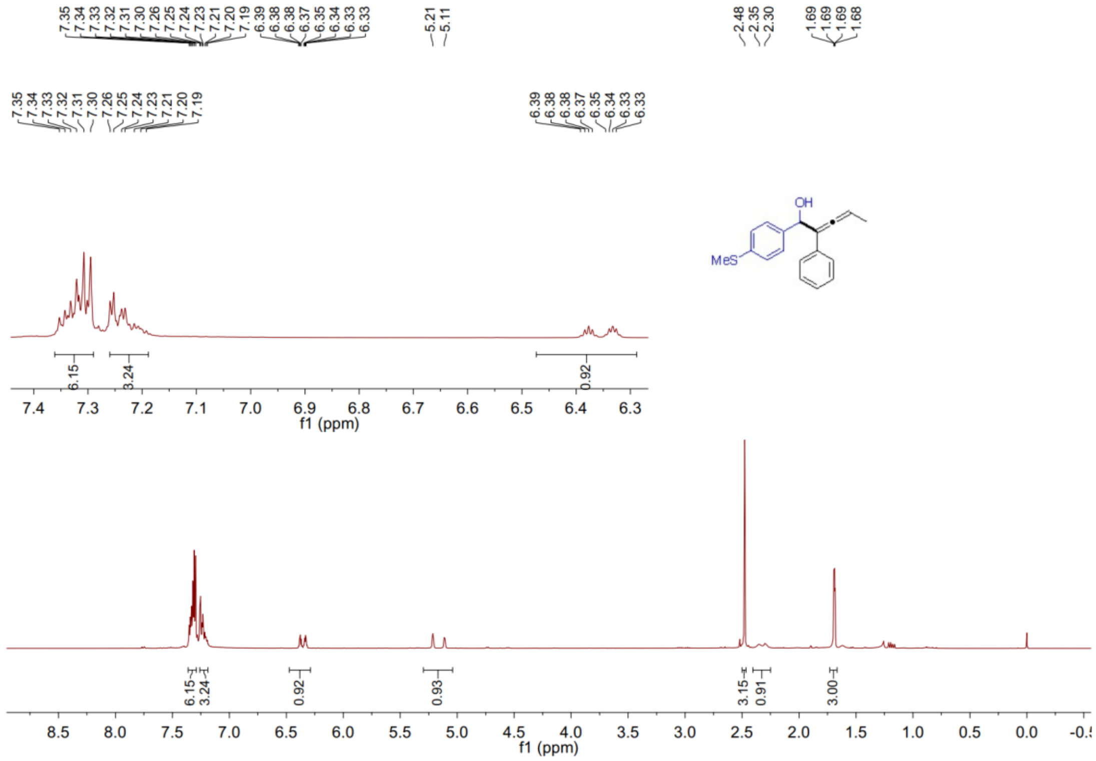

5q ^{13}C NMR

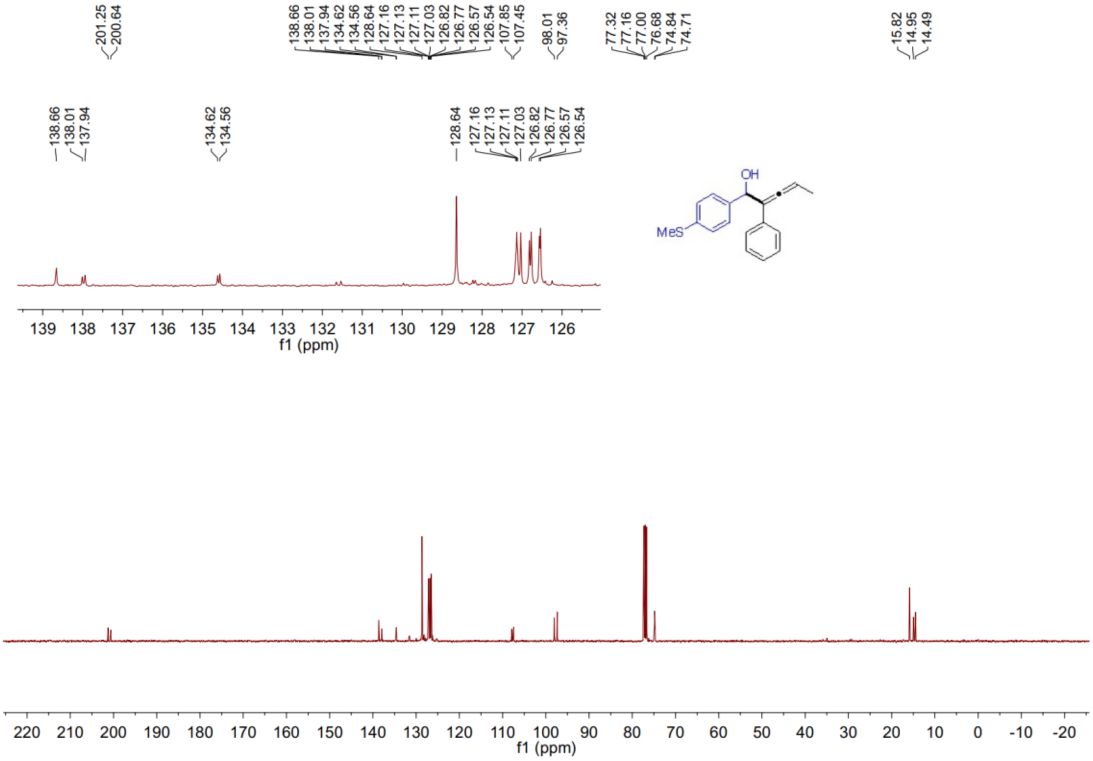

5r ^{1}H NMR

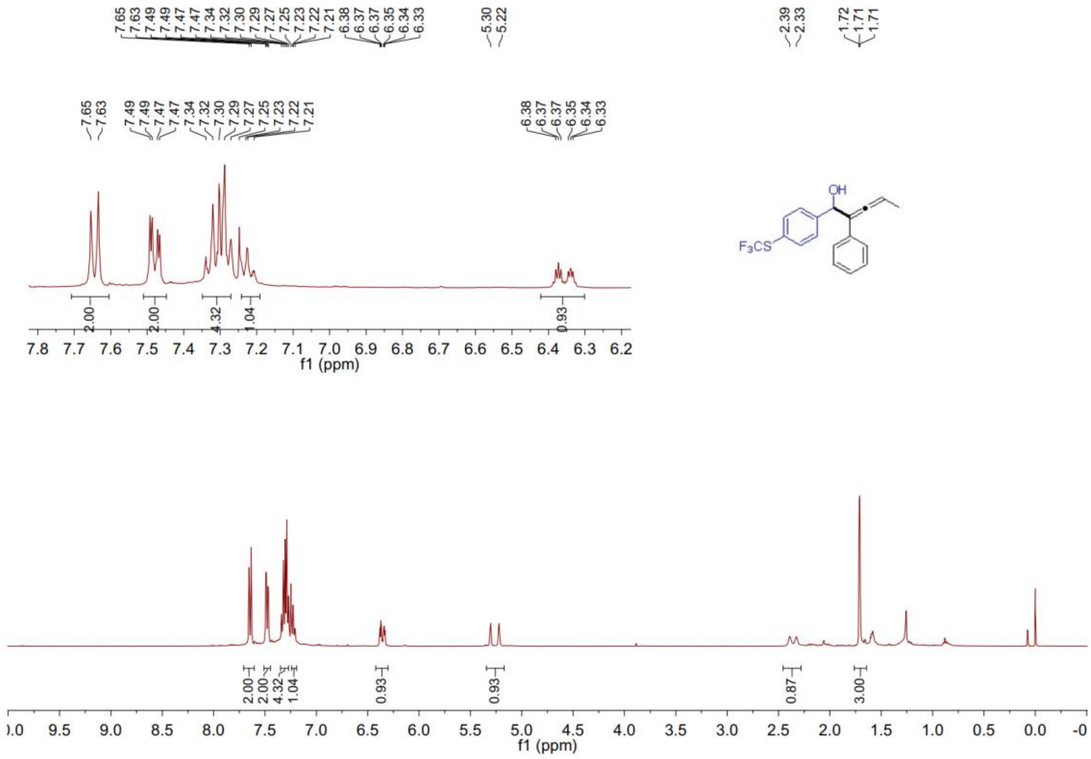

5r ^{13}C NMR

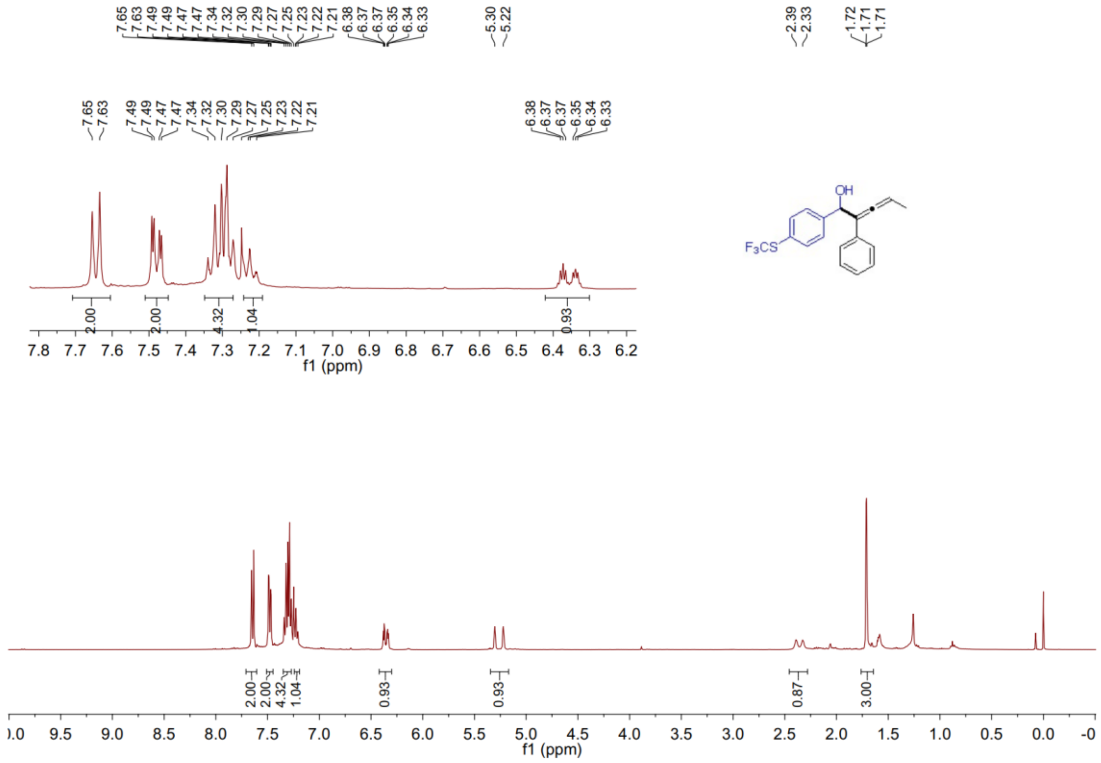

5s ^{1}H NMR

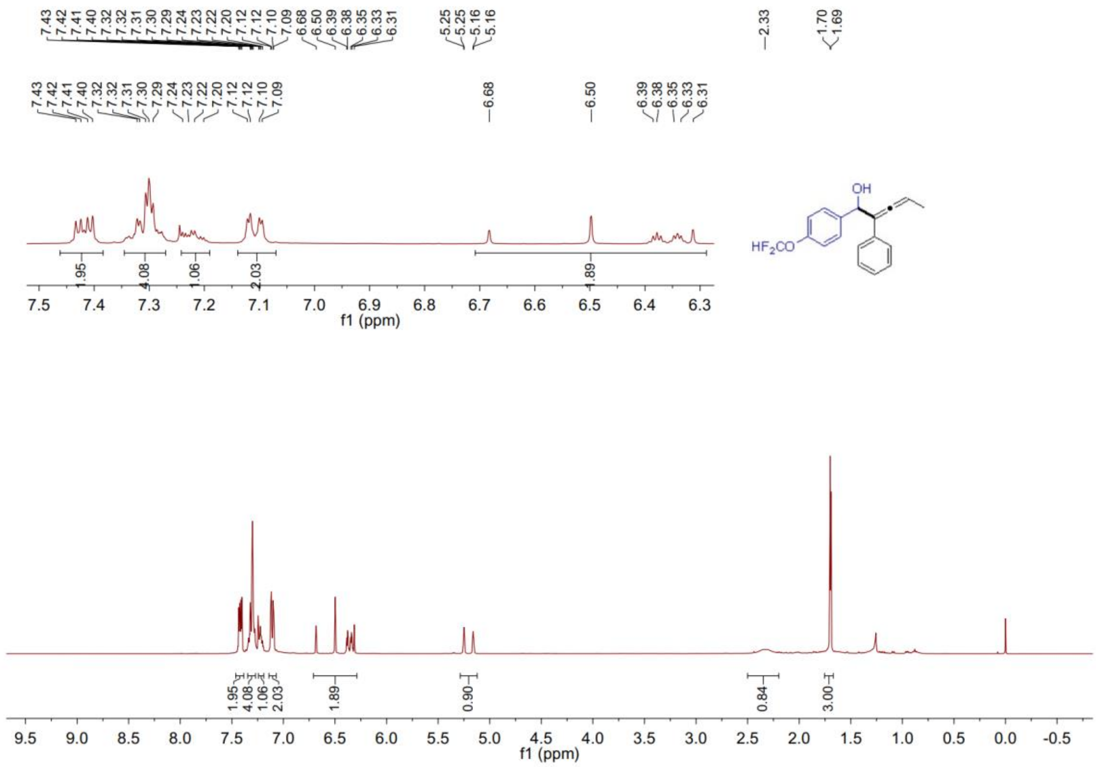

5s ^{13}C NMR

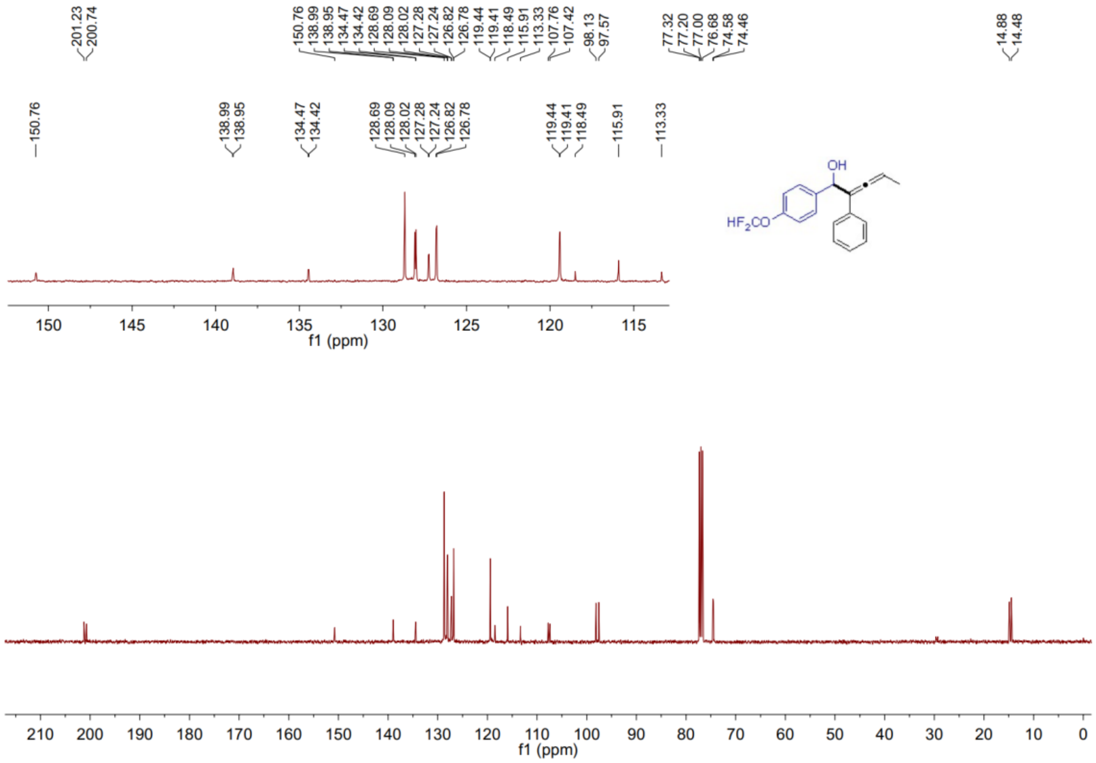

5t ^{1}H NMR

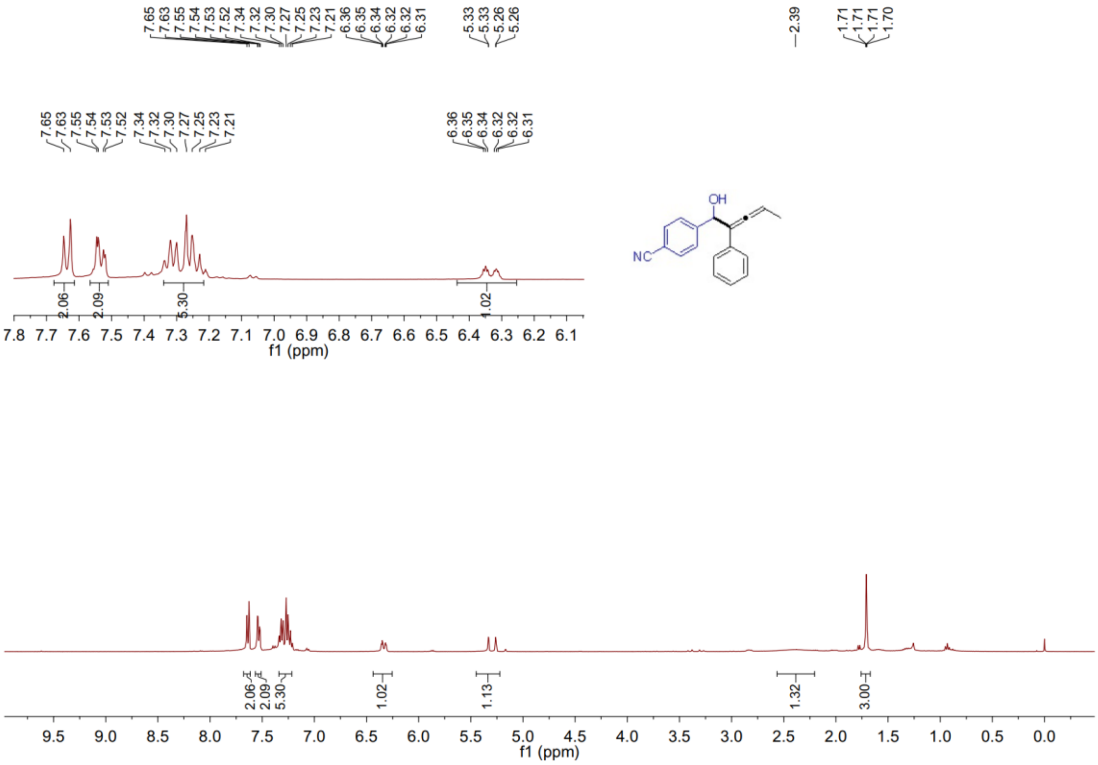

5t ^{13}C NMR

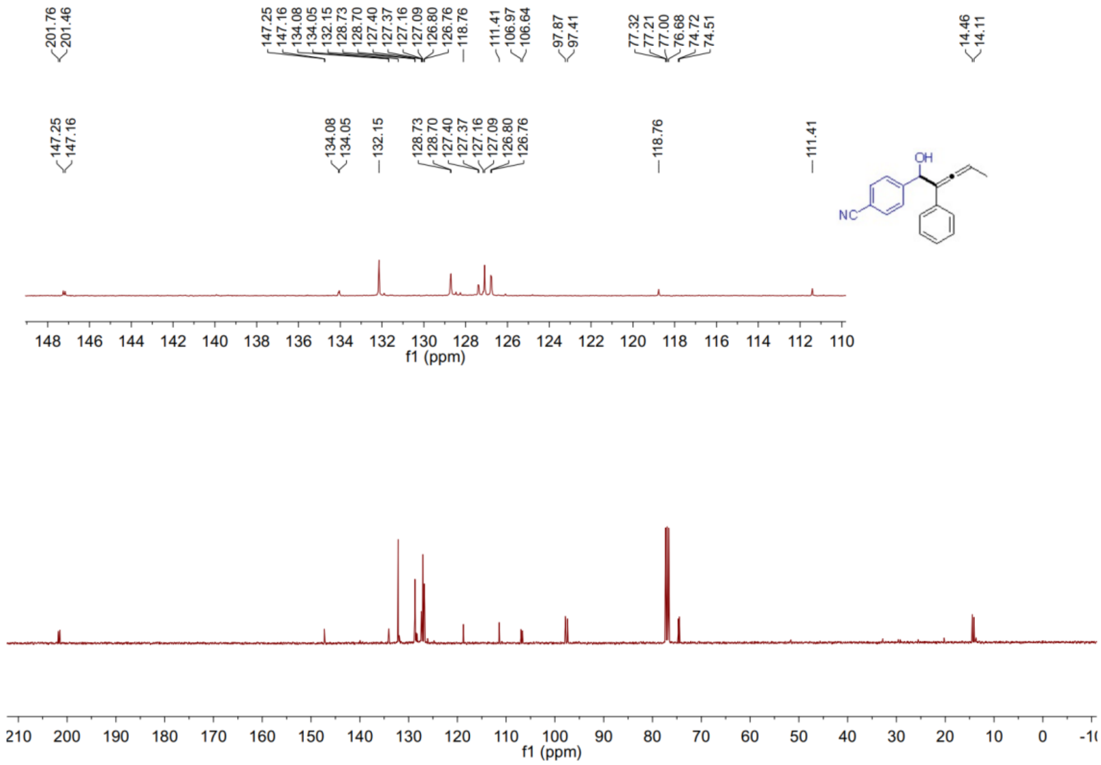

5u ^{1}H NMR

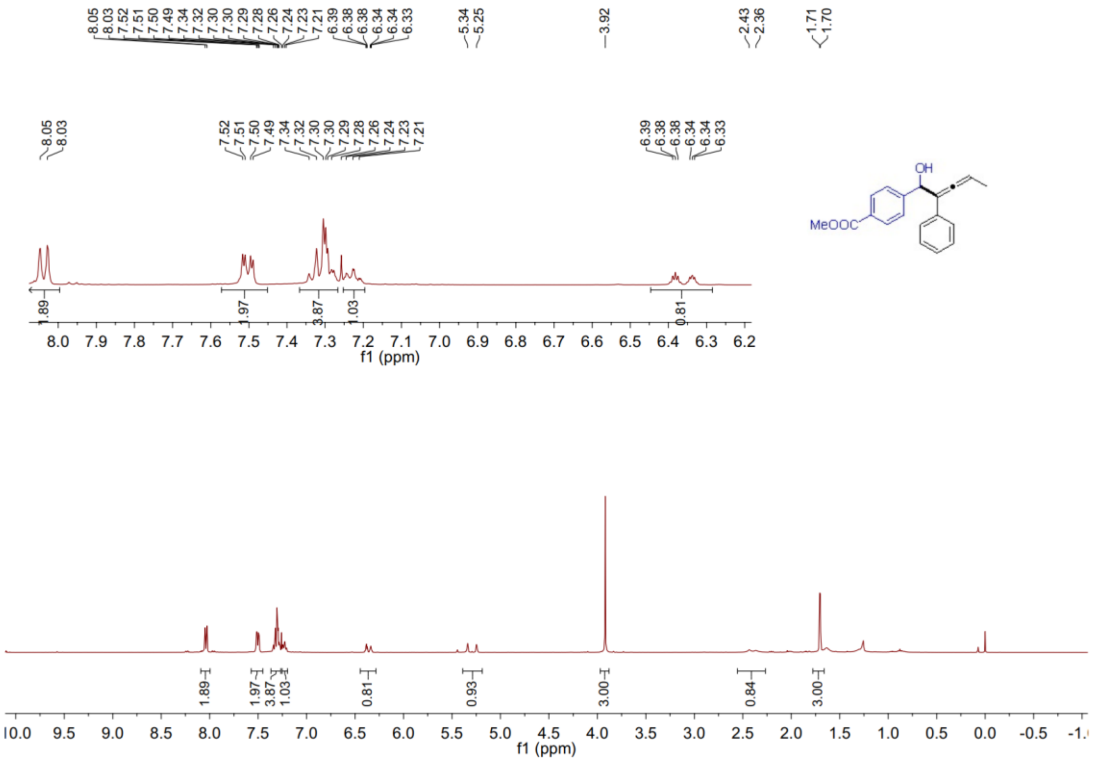

5u ^{13}C NMR

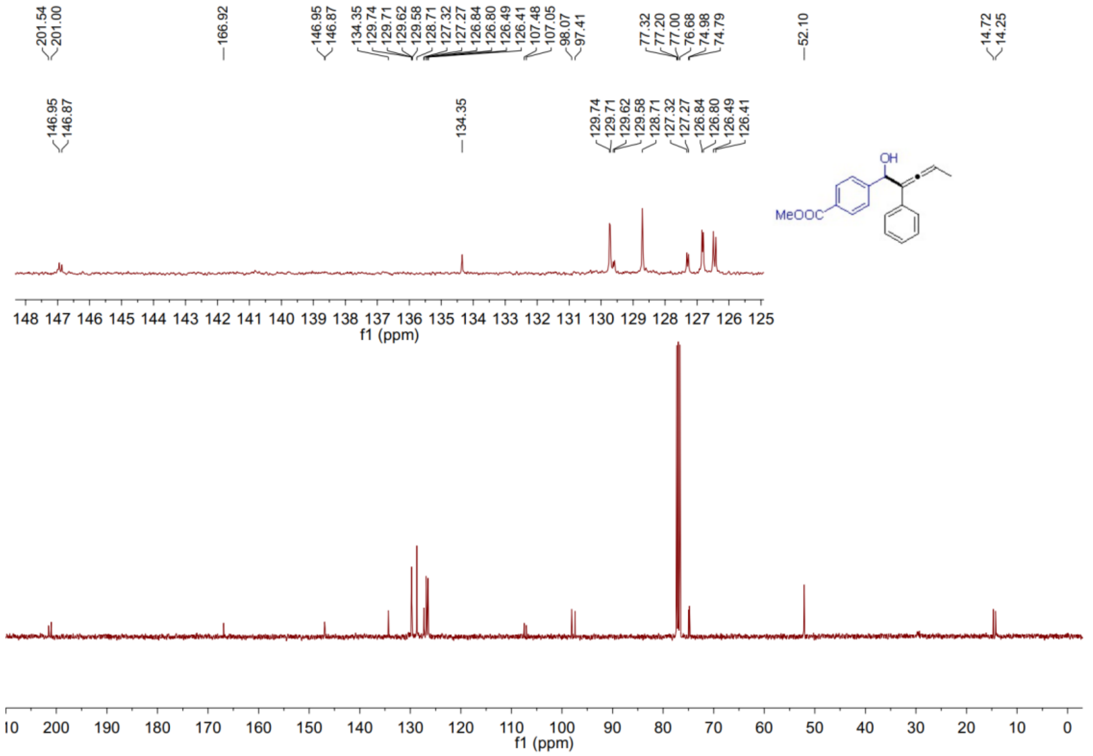

5v ^{1}H NMR

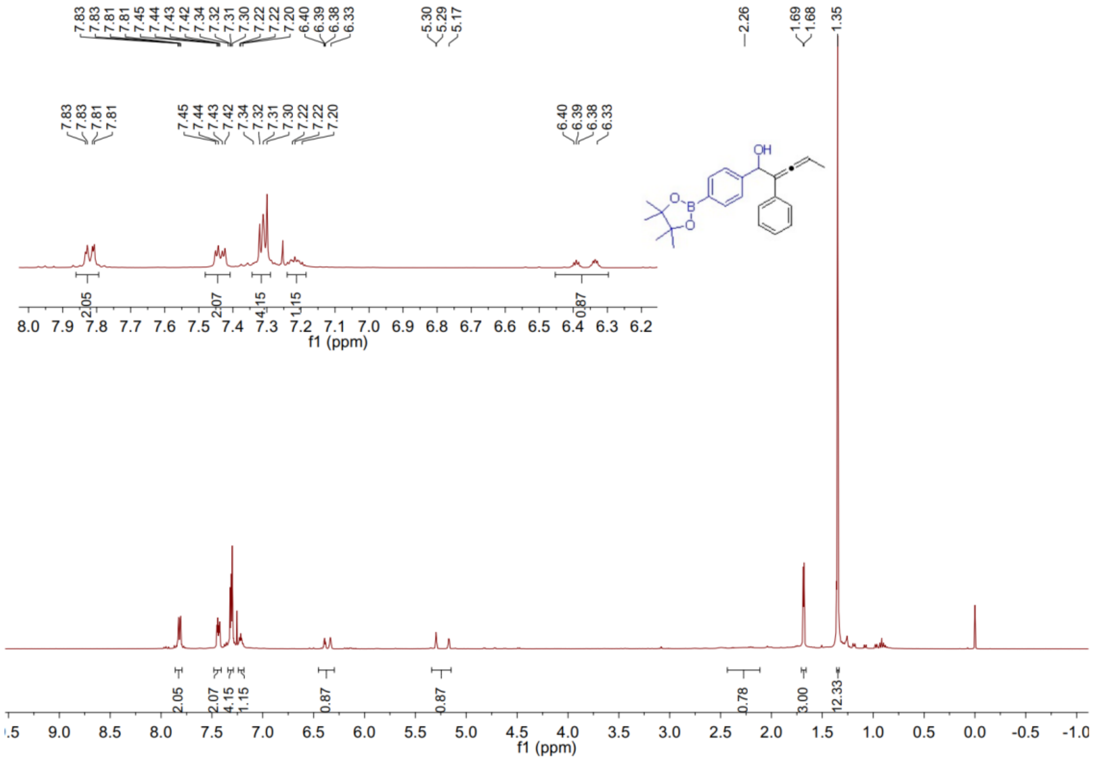

5v ^{13}C NMR

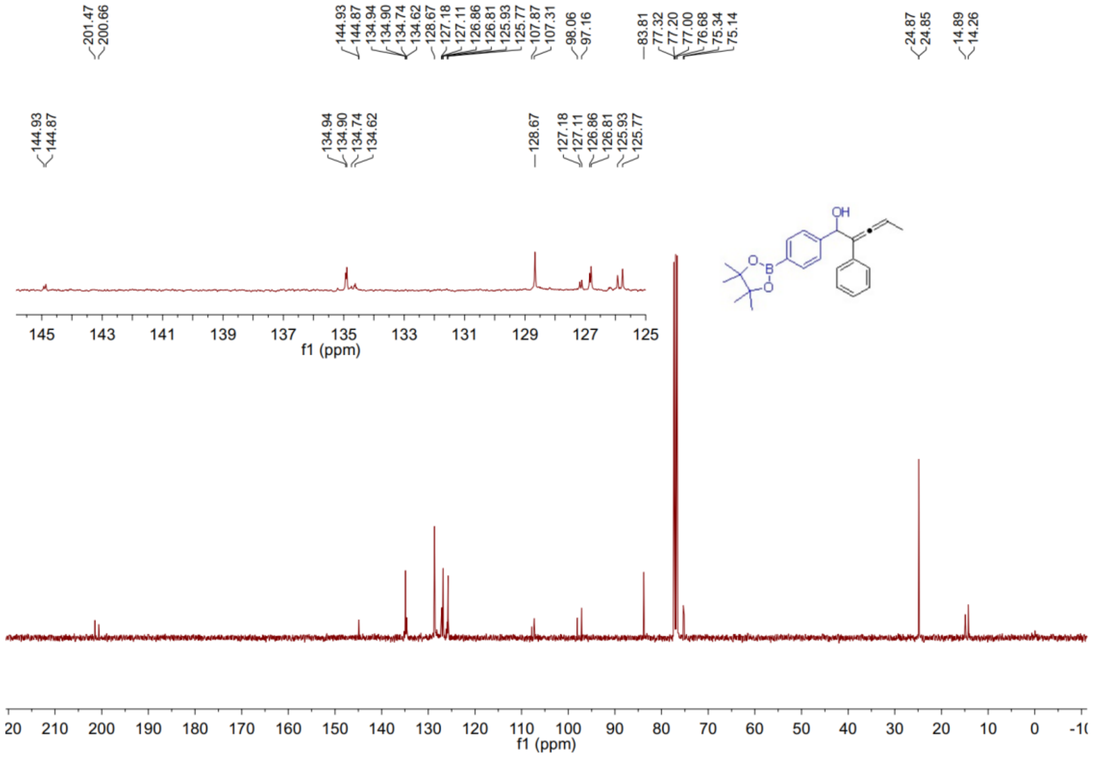

5w ^{1}H NMR

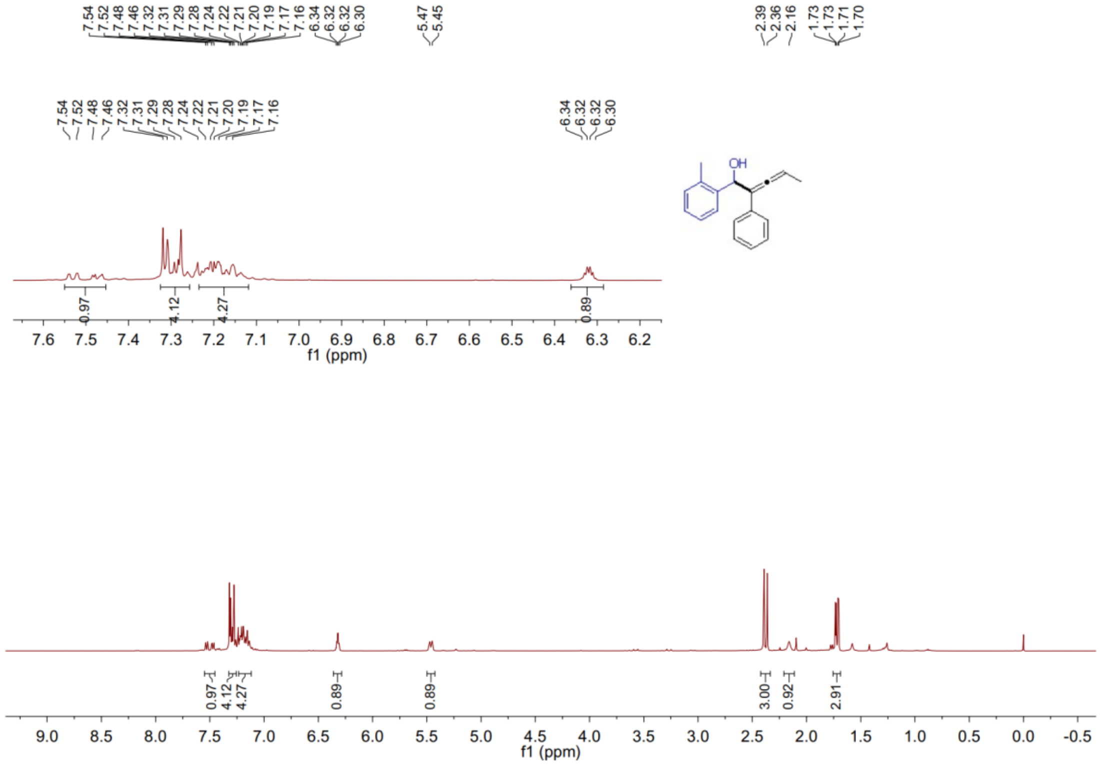

5w ^{13}C NMR

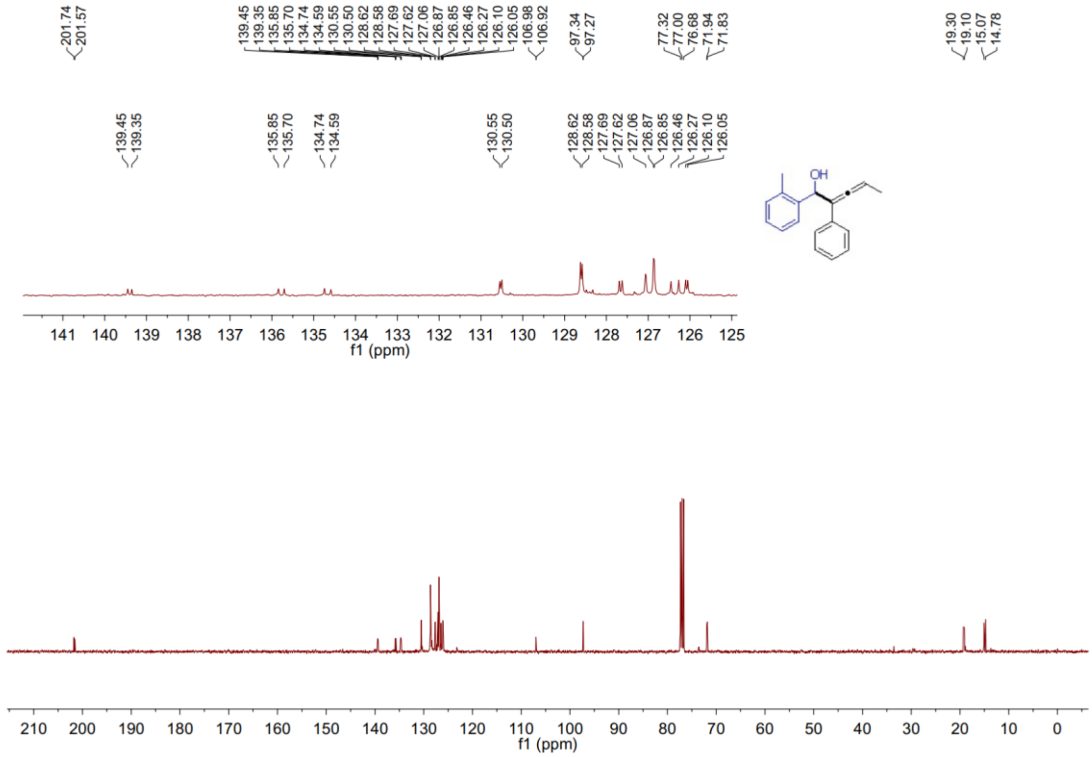

5x ^{1}H NMR

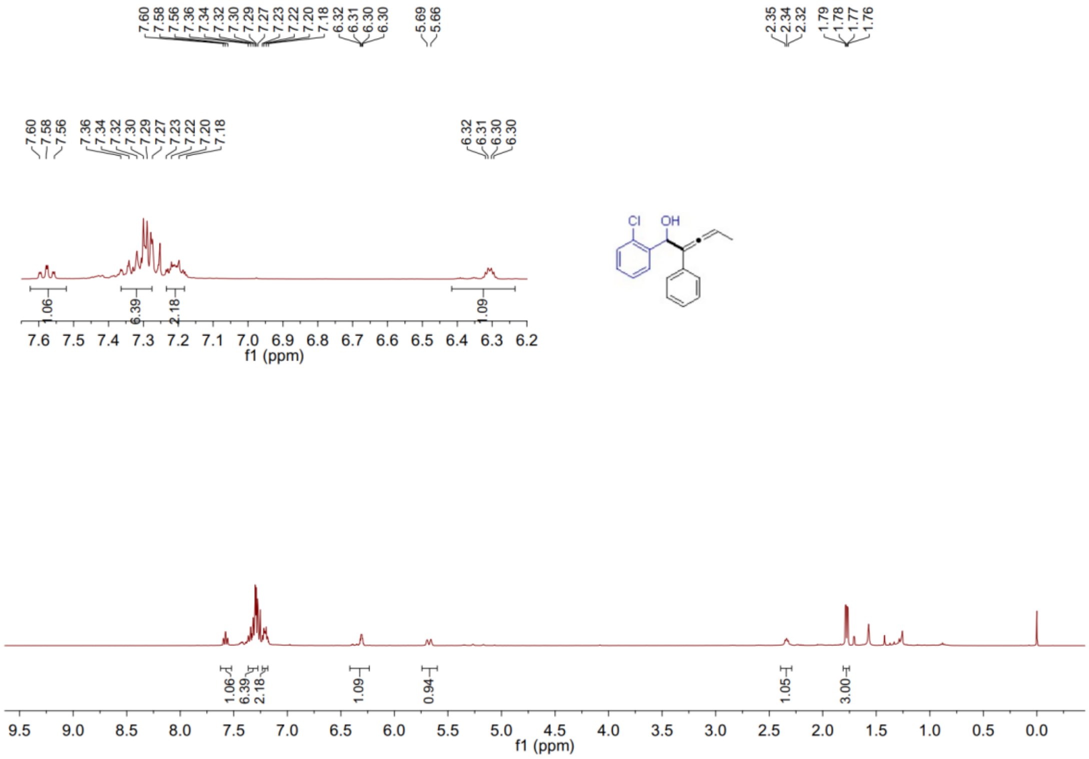

5x ^{13}C NMR

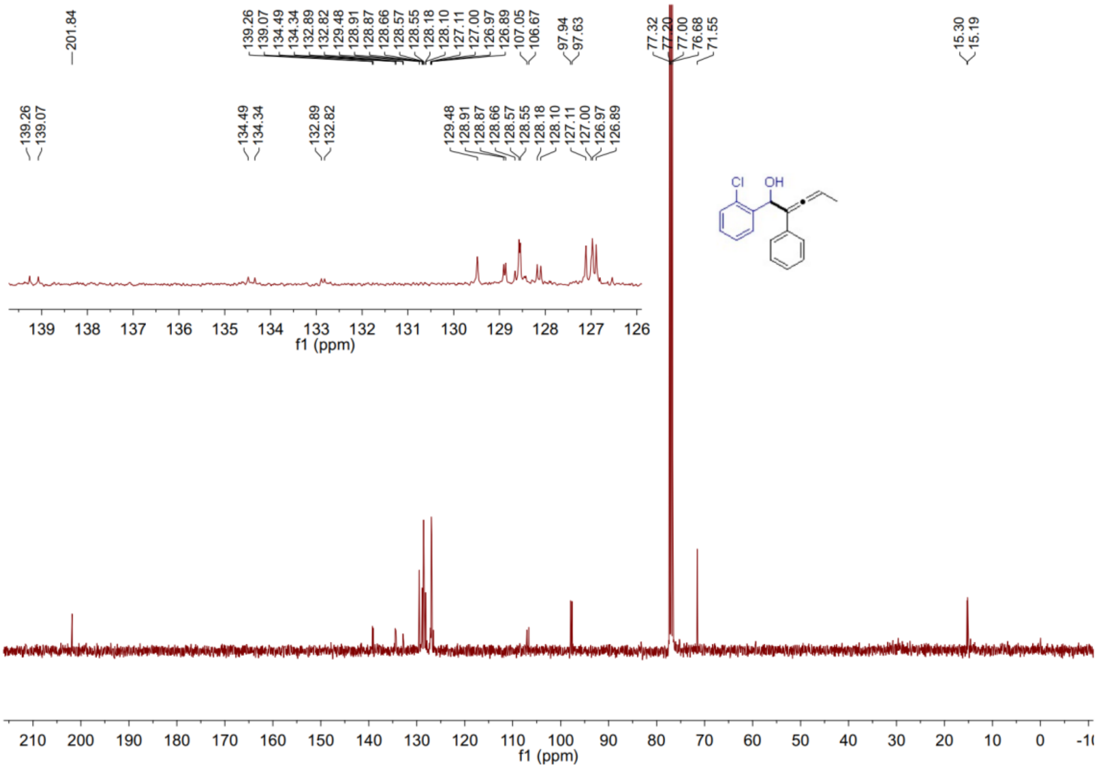

5y ^{1}H NMR

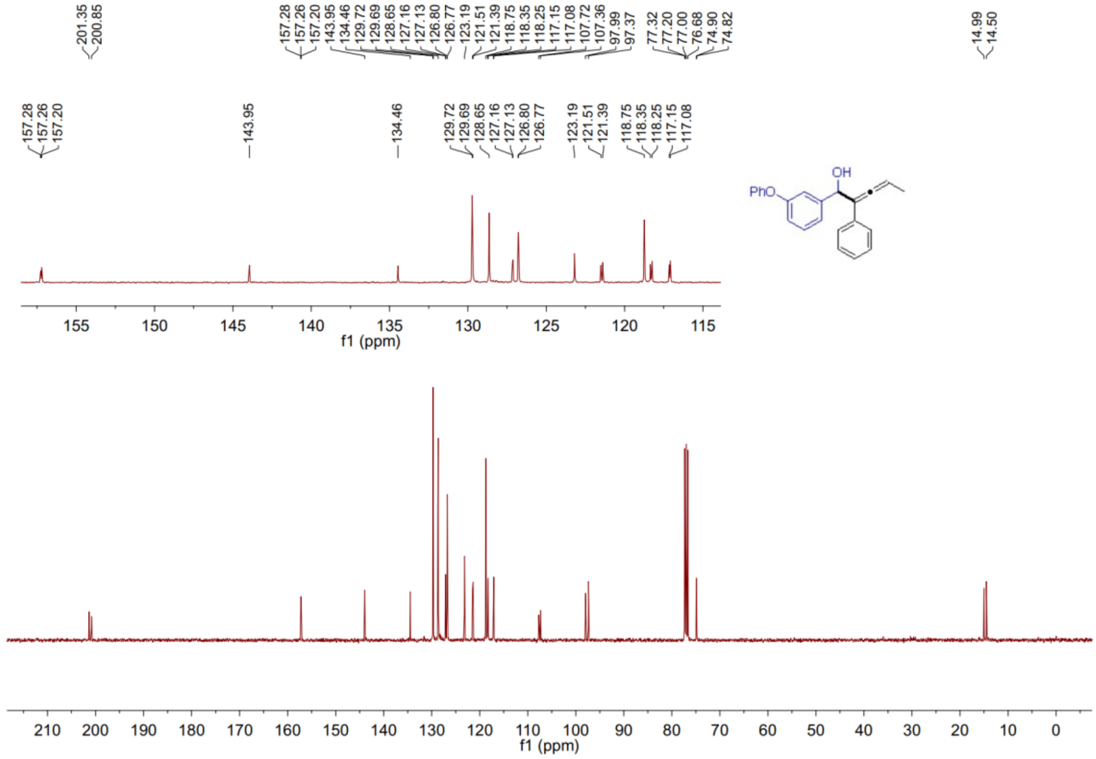

5y ^{13}C NMR

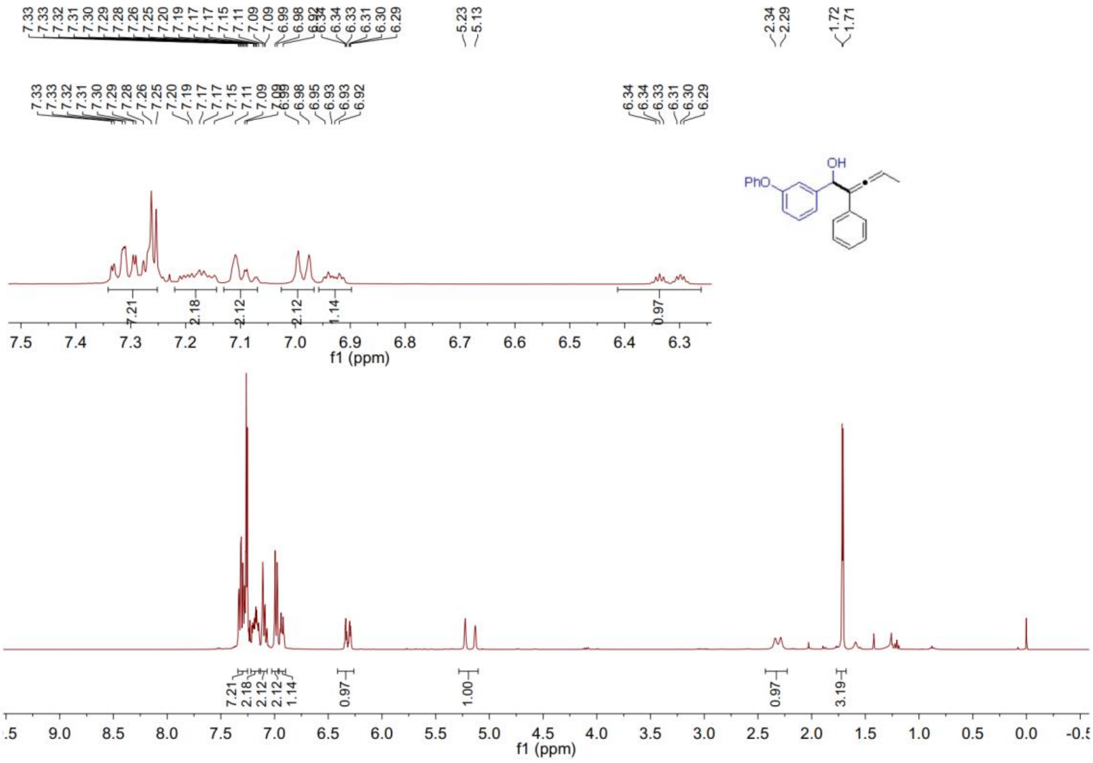

5z ¹H NMR

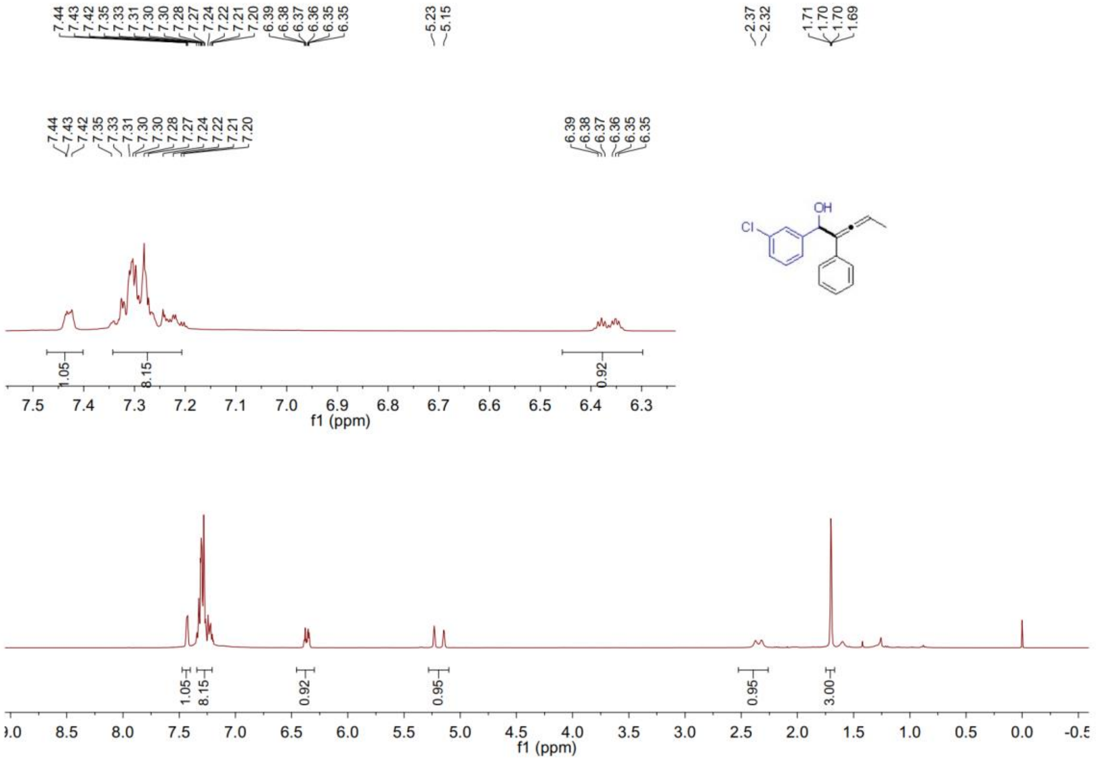

5z ¹³C NMR

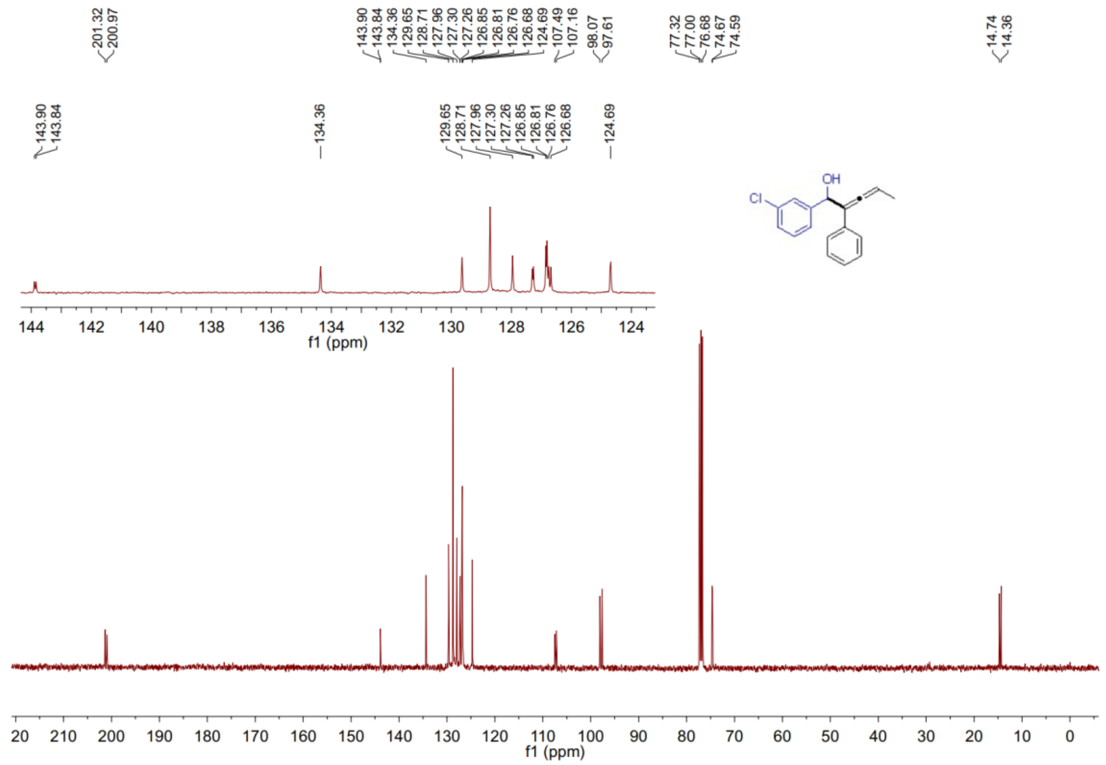

5aa ^{1}H NMR

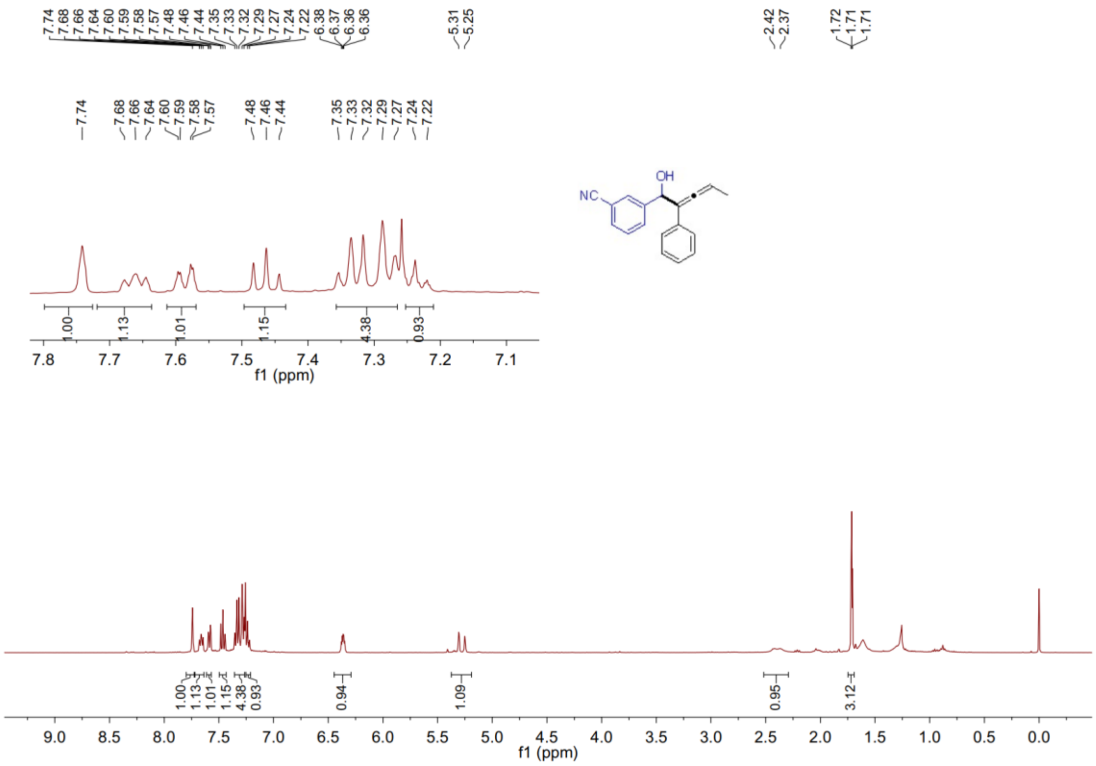

5aa ^{13}C NMR

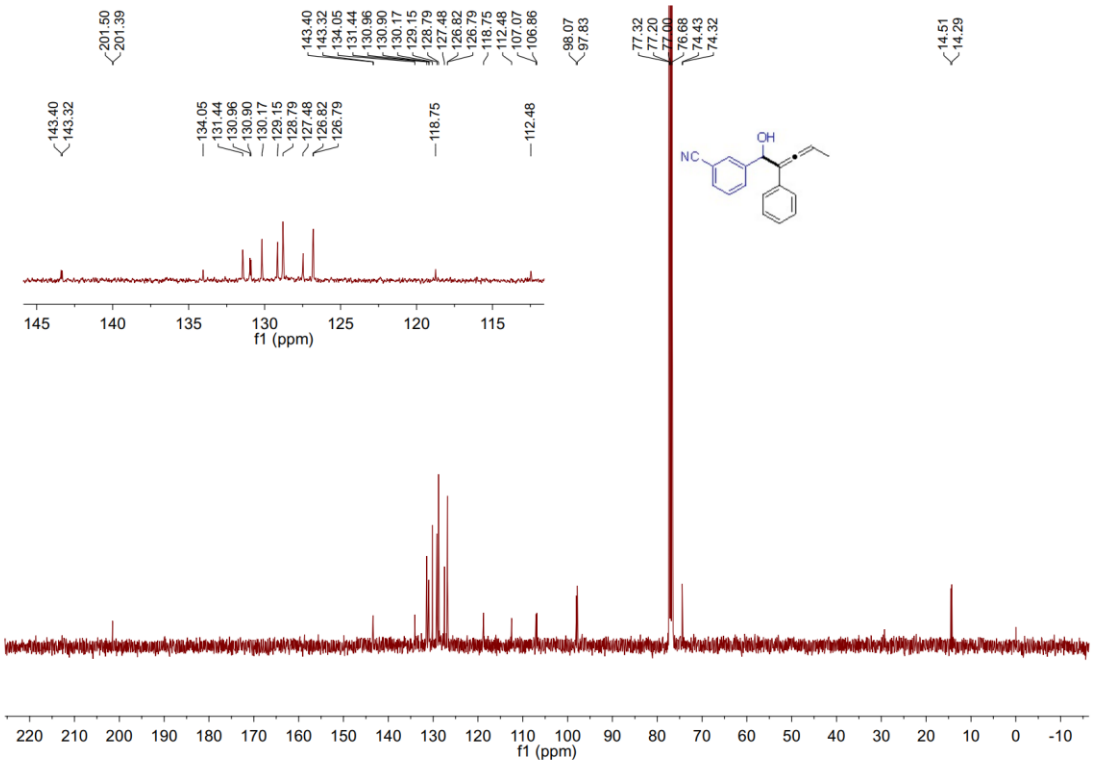

5ab ^{1}H NMR

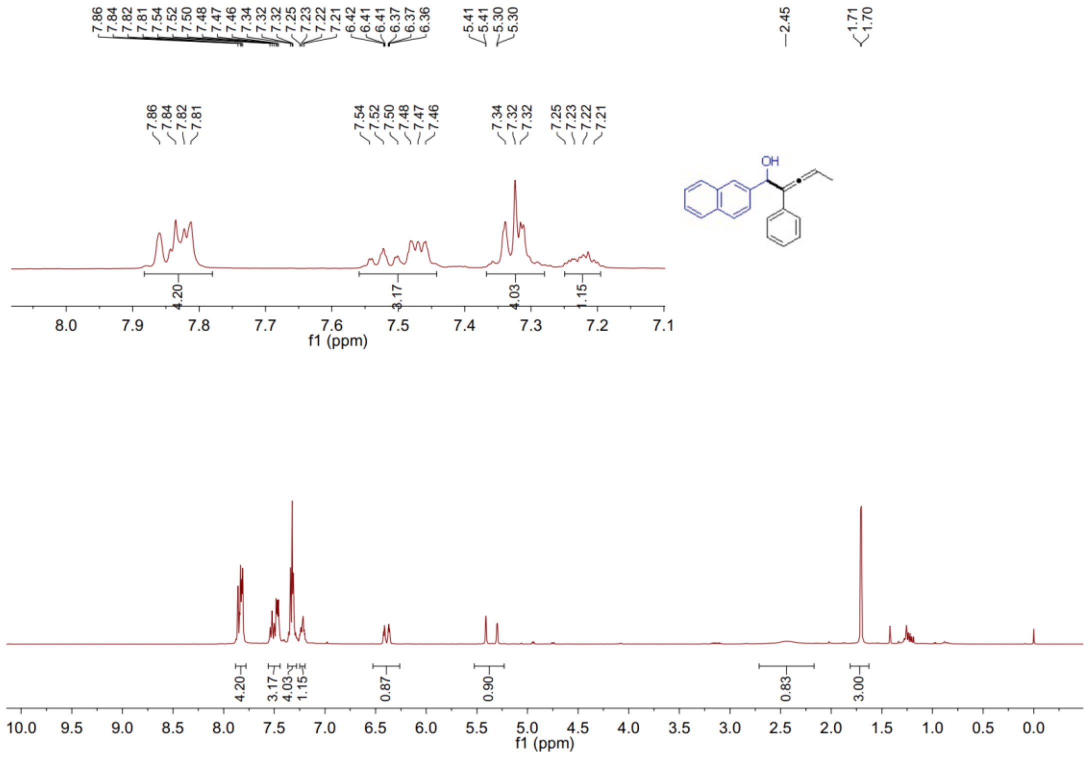

5ab ^{13}C NMR

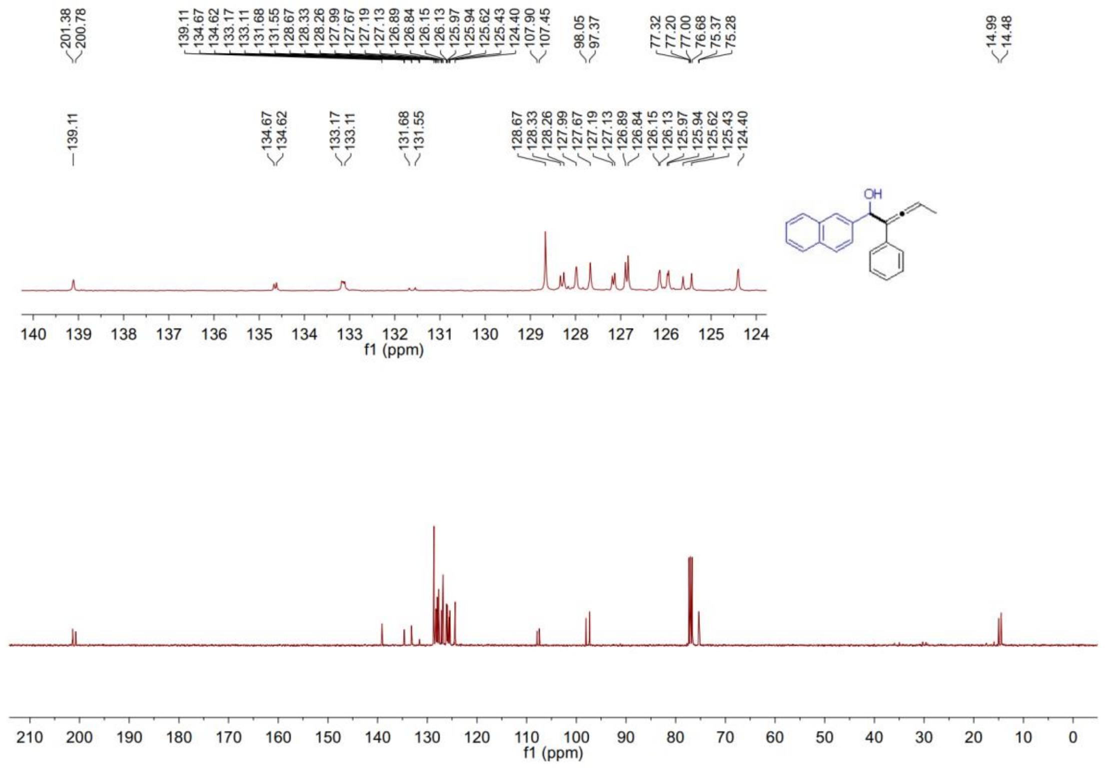

5ac ^{1}H NMR

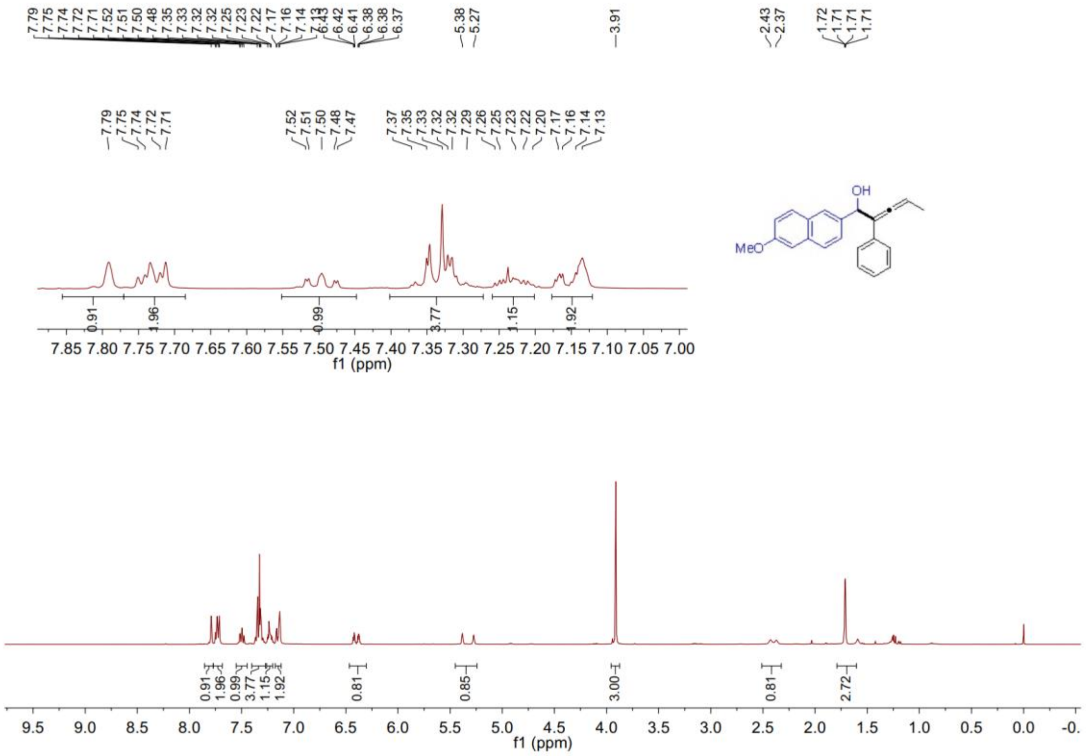

5ac ^{13}C NMR

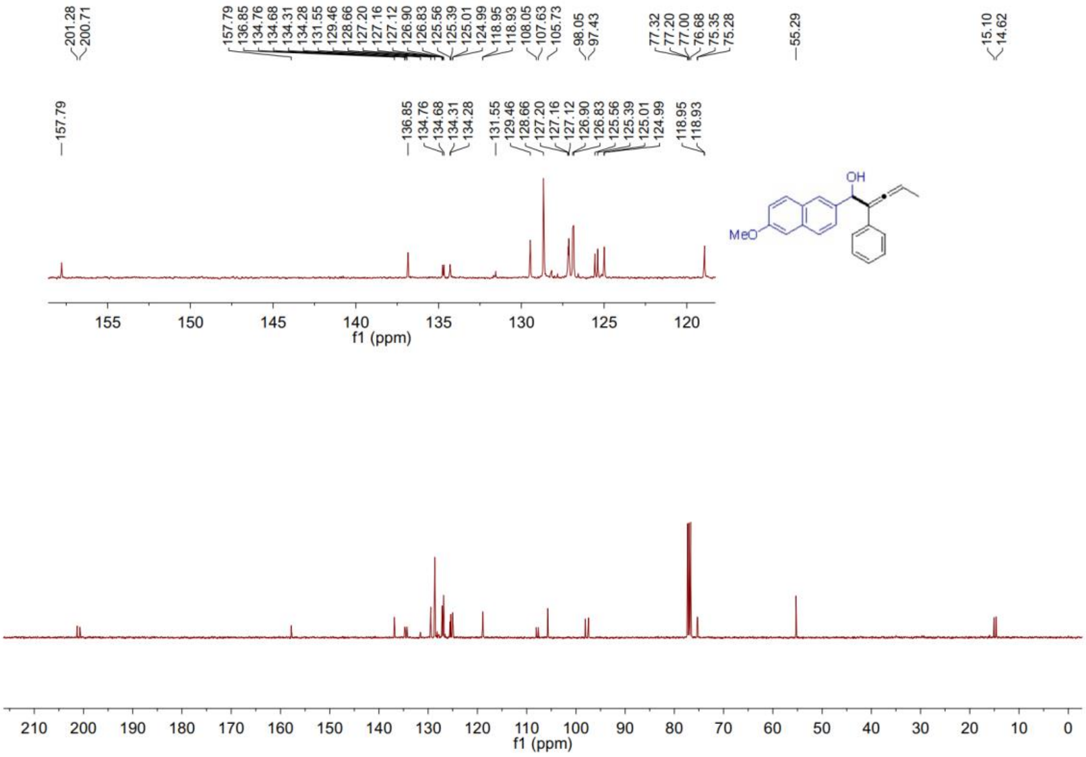

5ad ^{1}H NMR

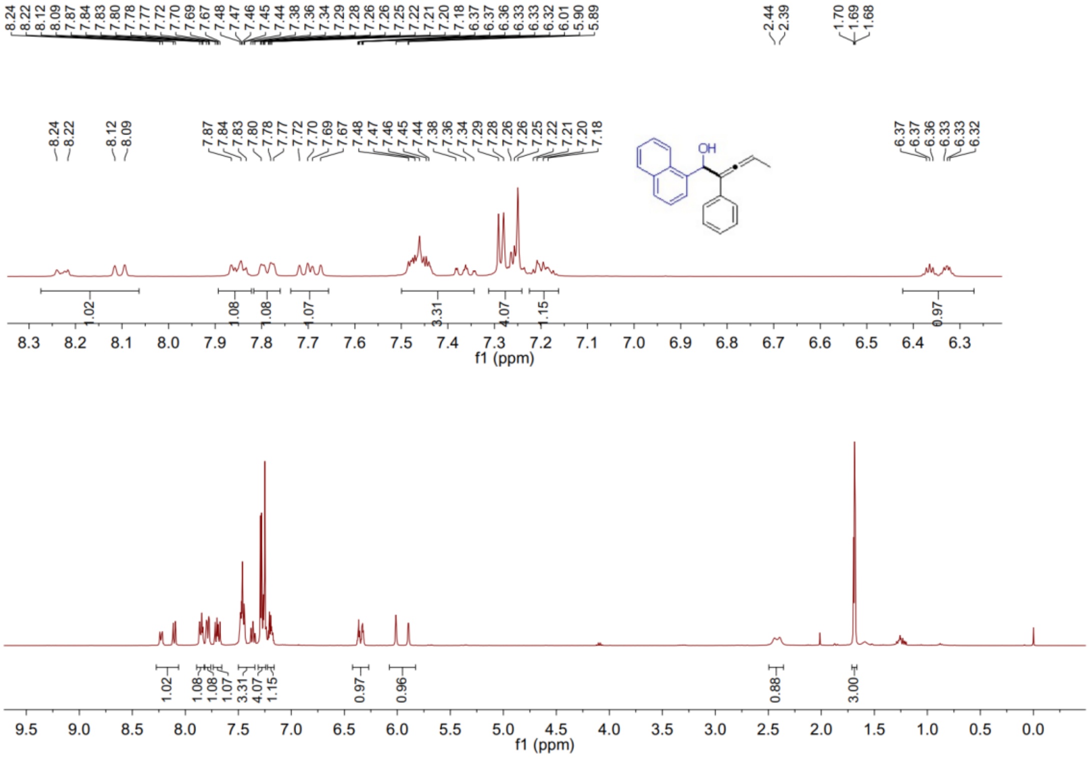

5ad ^{13}C NMR

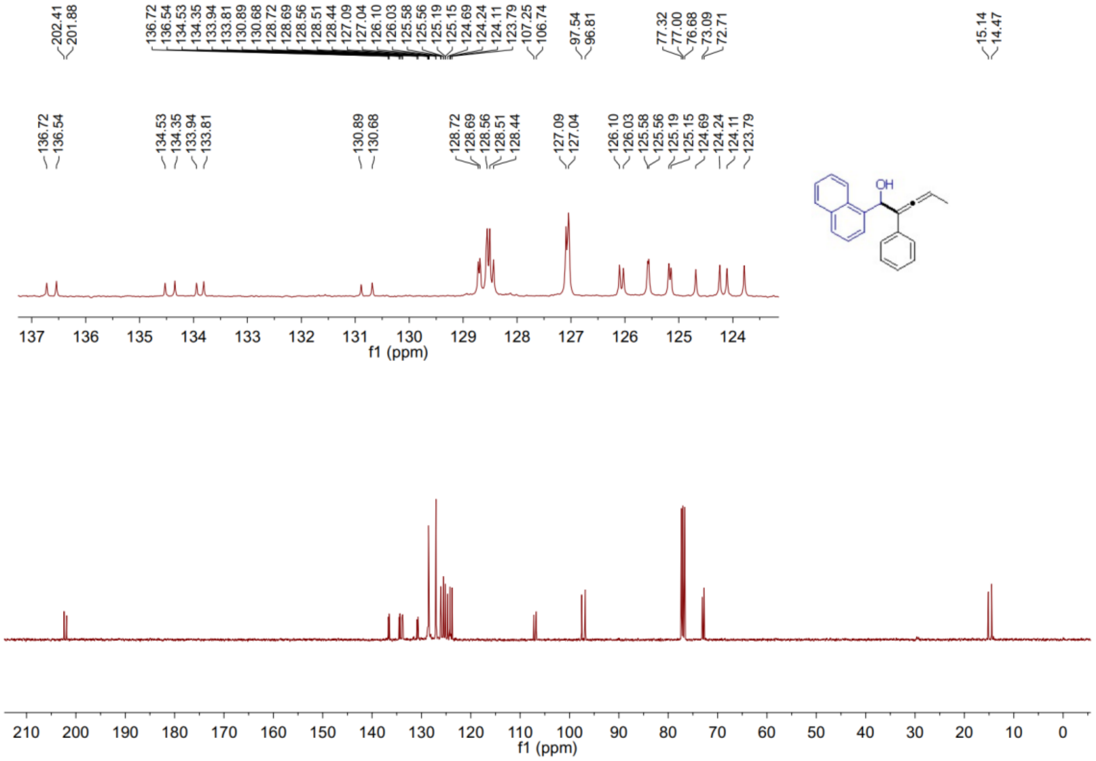

5ae ^{1}H NMR

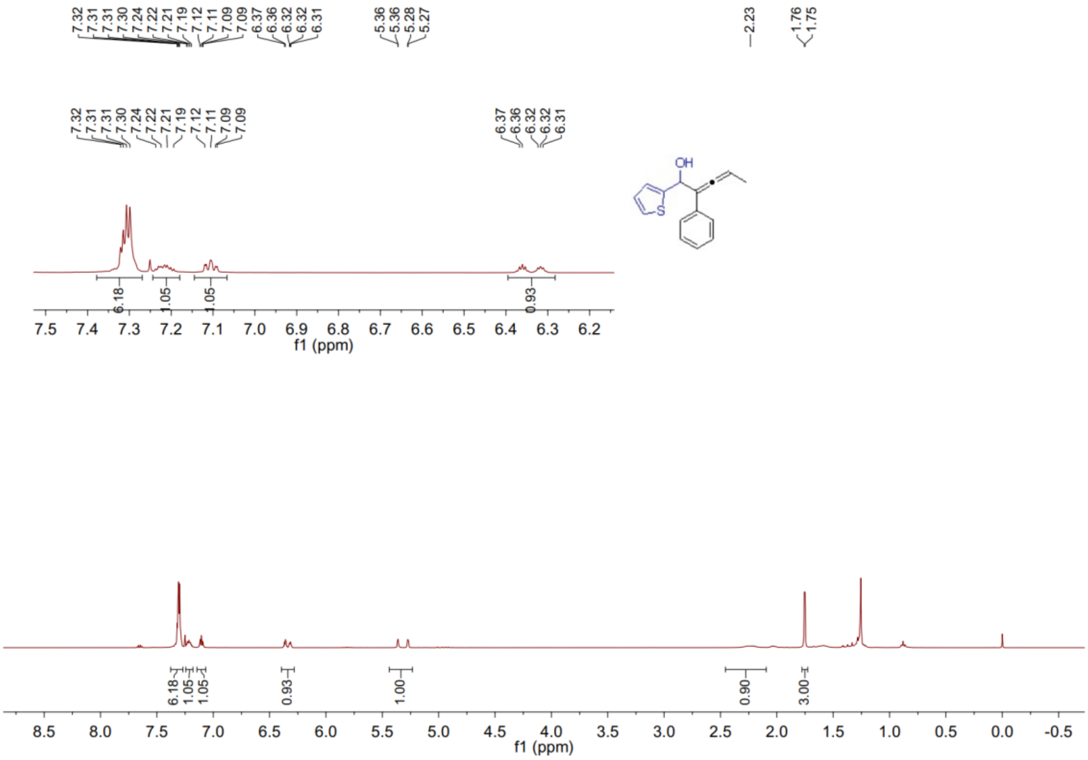

5ae ^{13}C NMR

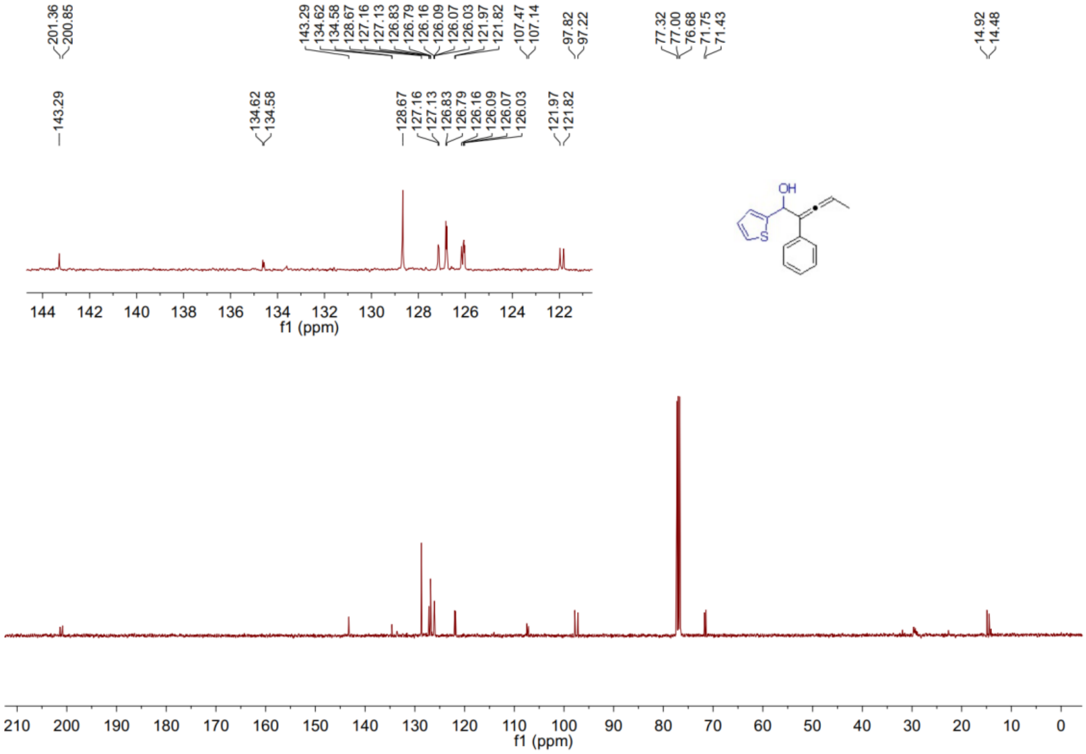

5af ^{1}H NMR

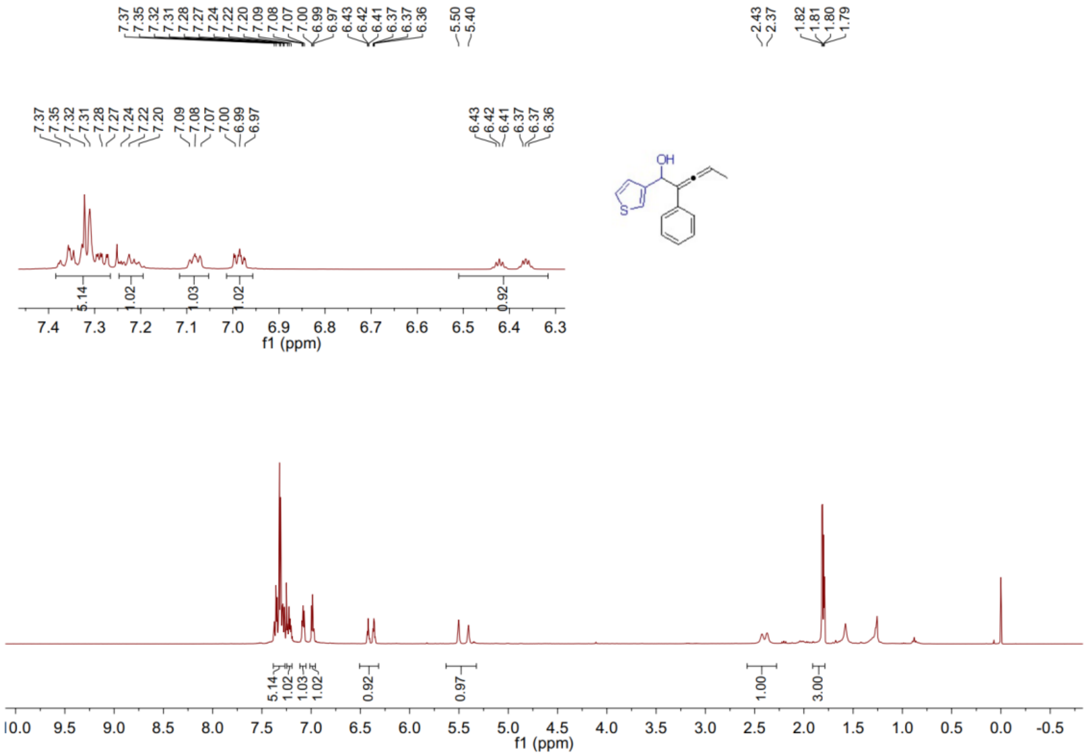

5af ^{13}C NMR

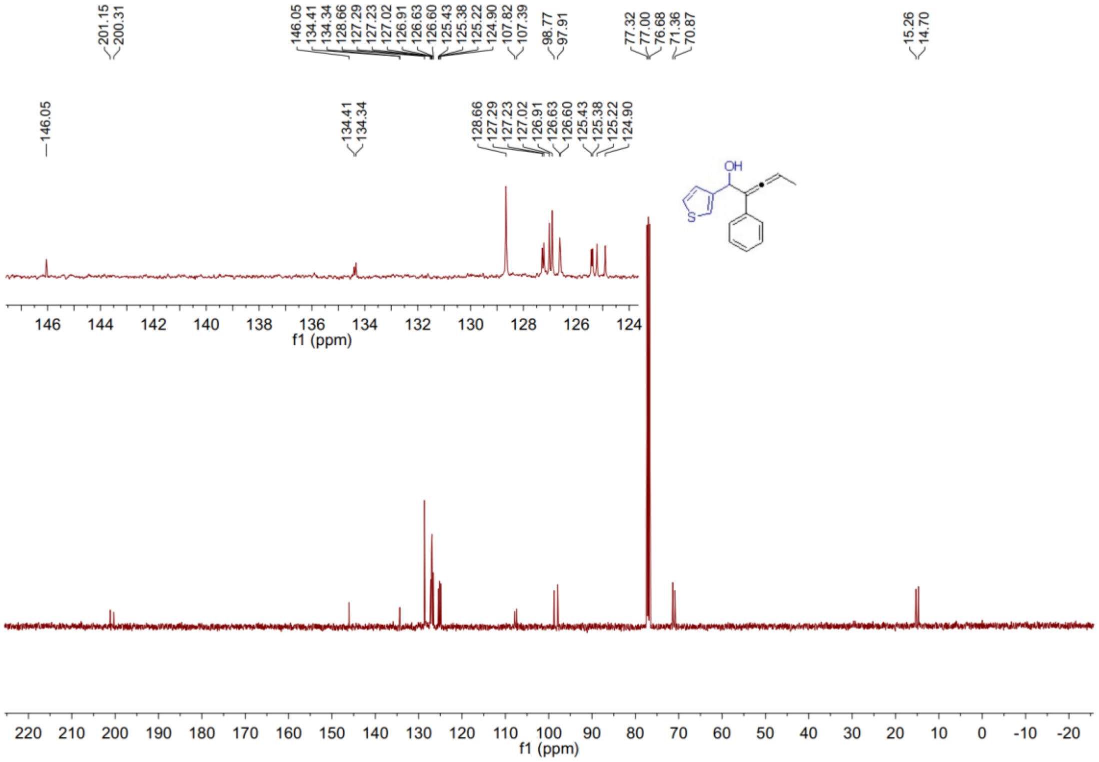

5ag ^{1}H NMR

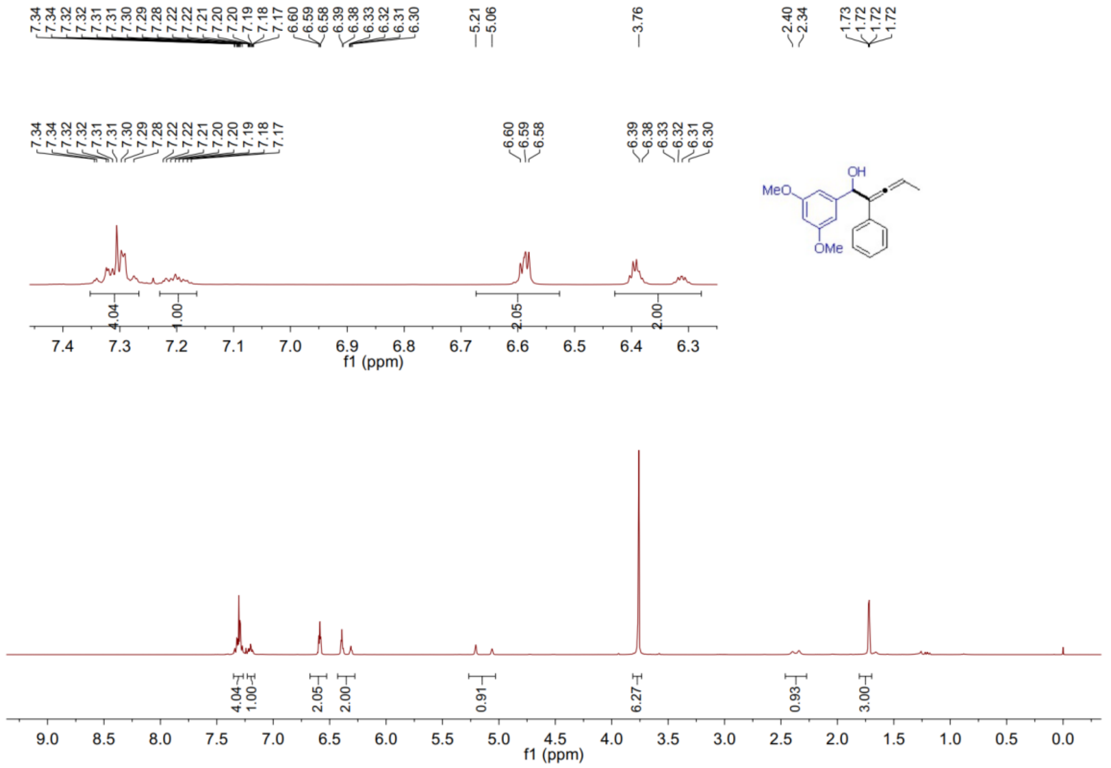

5ag ^{13}C NMR

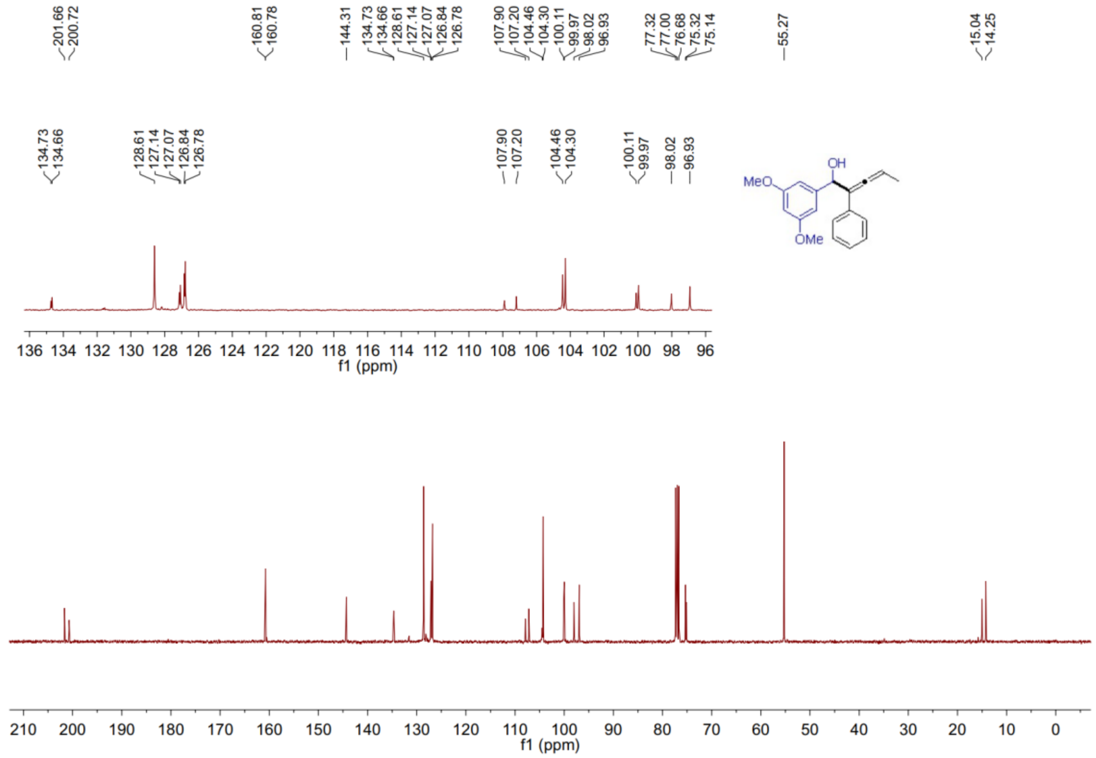

5ah ^{1}H NMR

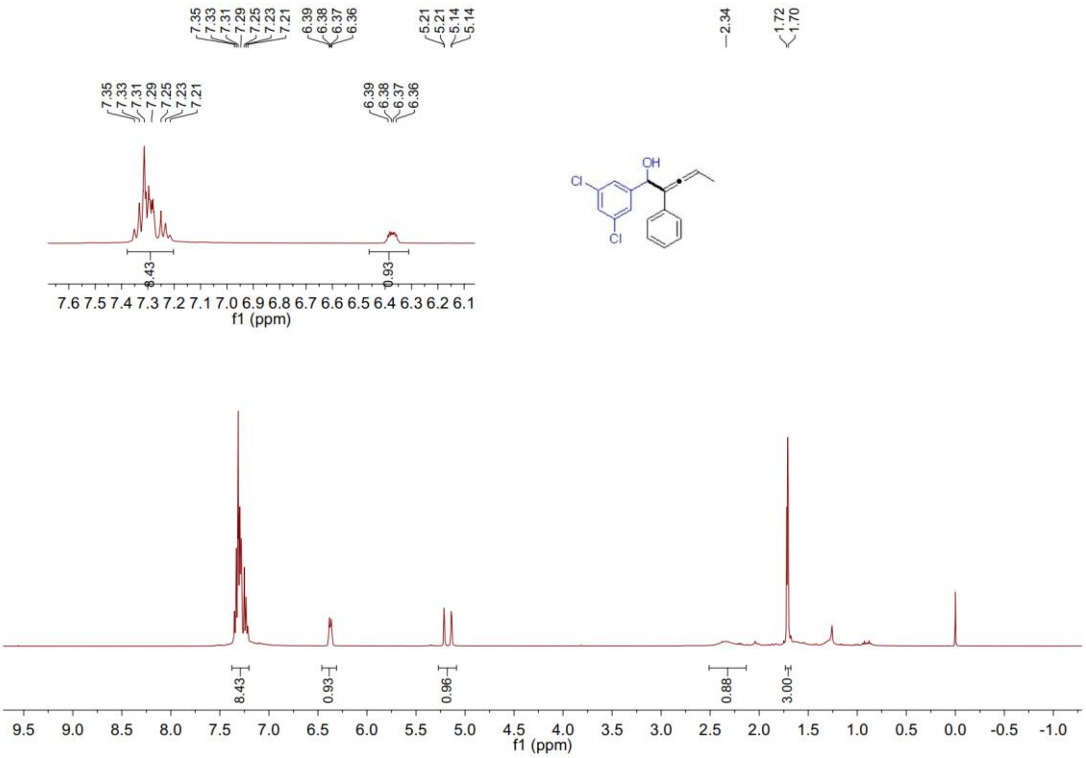

5ah ^{13}C NMR

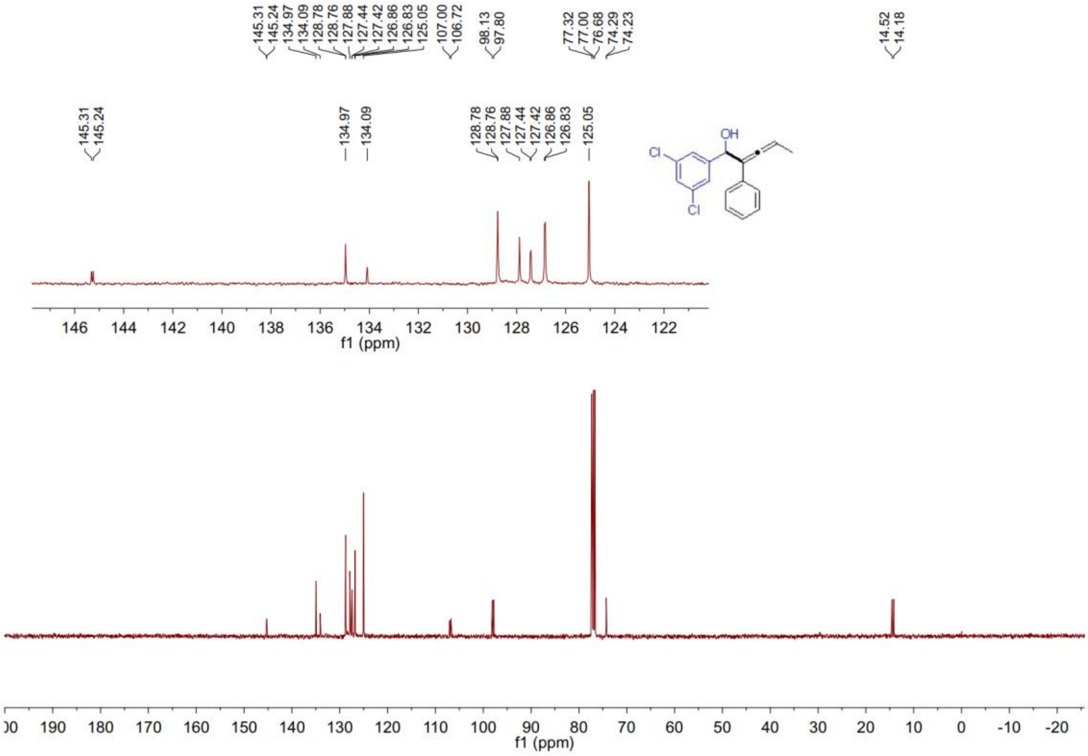

5ai ^{1}H NMR

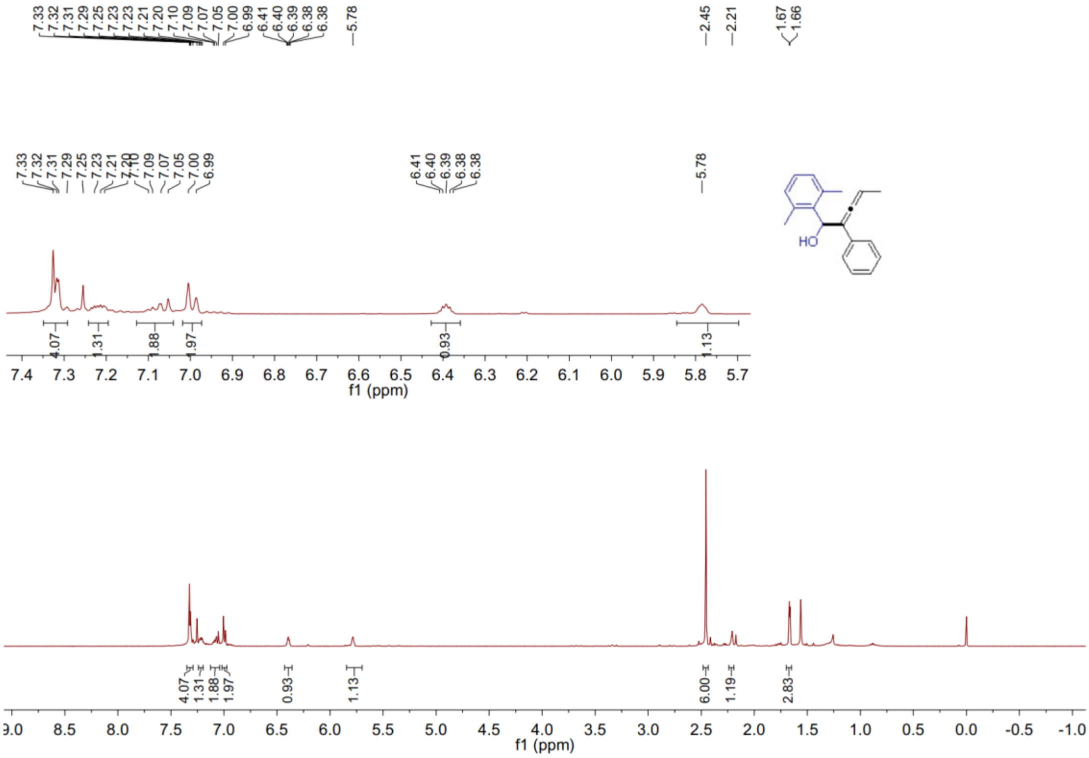

5ai ^{13}C NMR

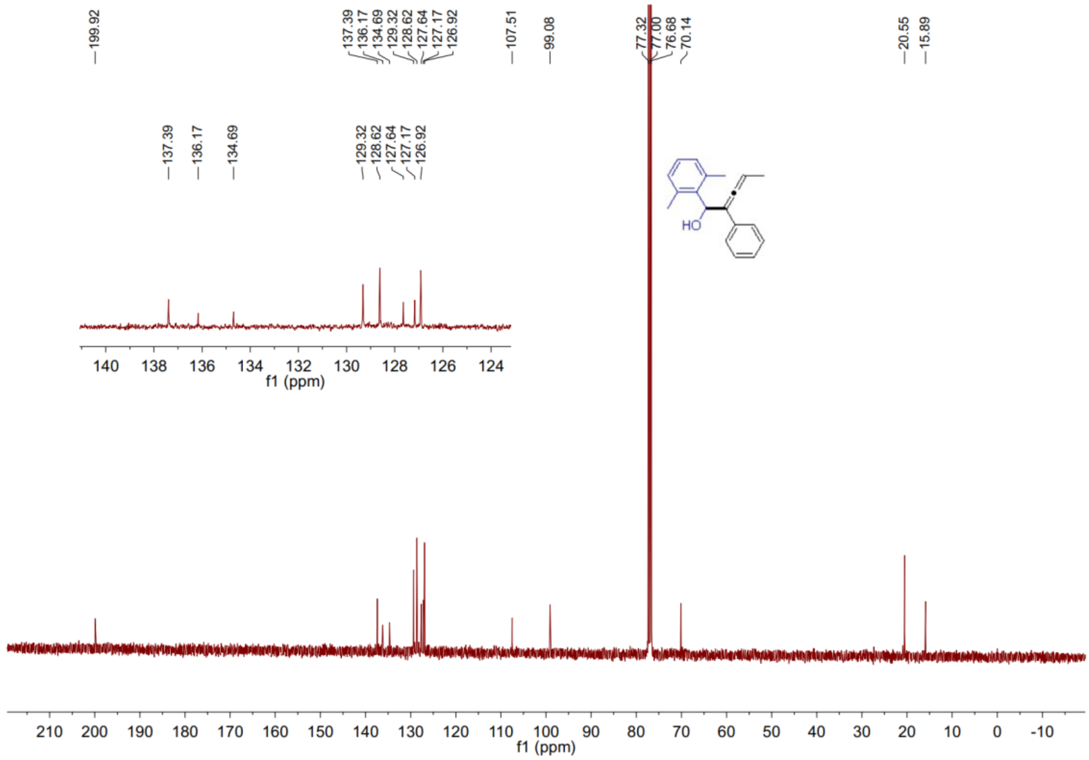

5aj ^{1}H NMR

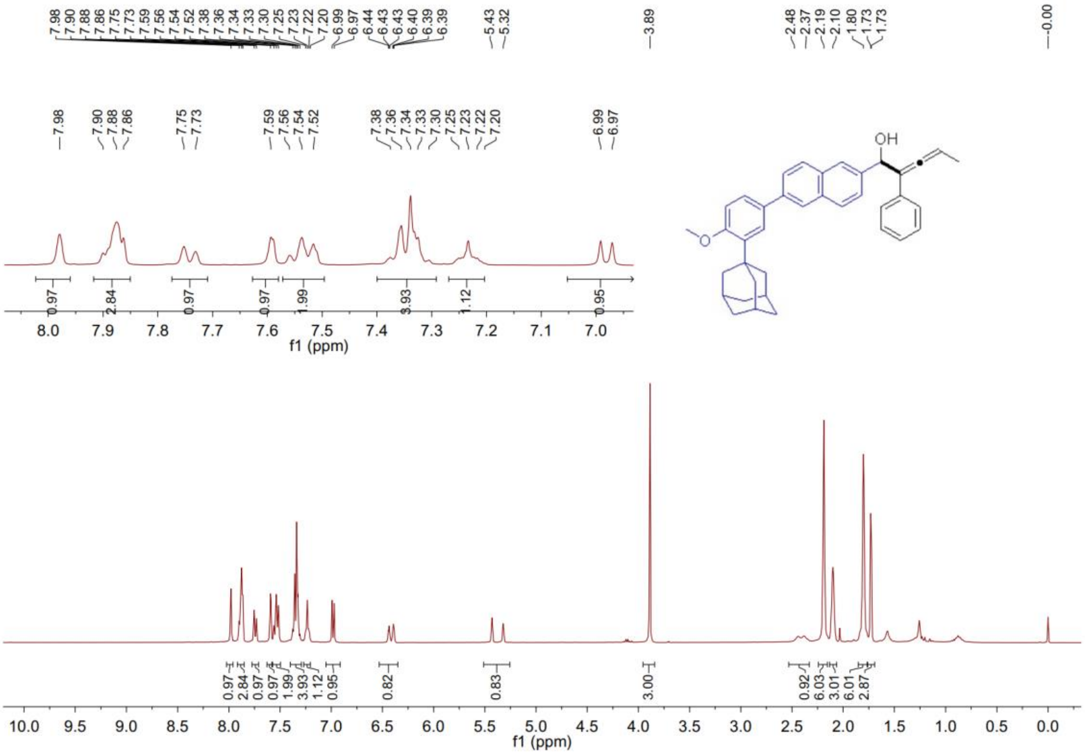

5aj ^{13}C NMR

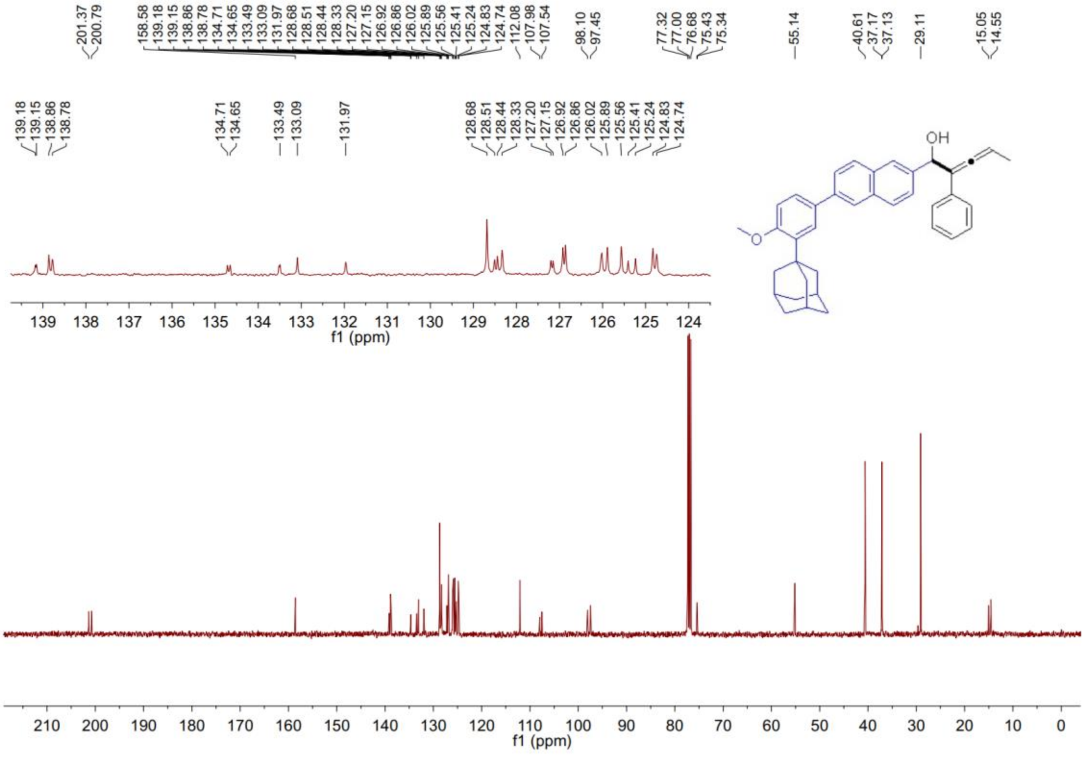

5ak ^{1}H NMR

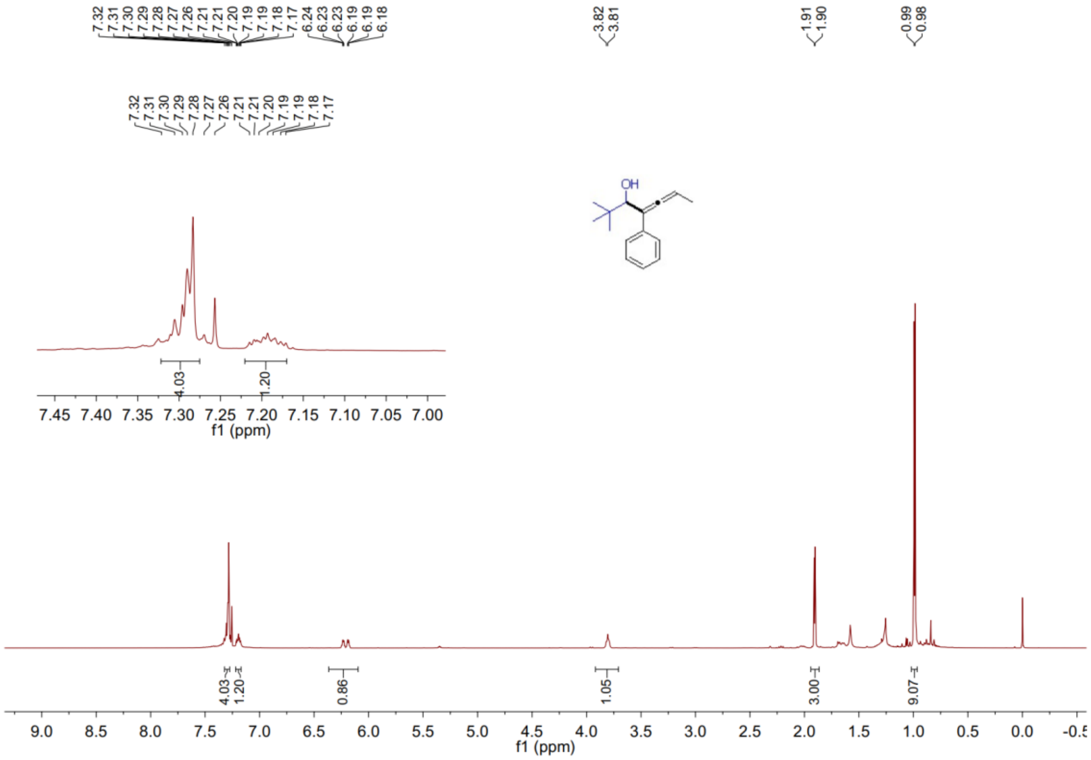

5ak ^{13}C NMR

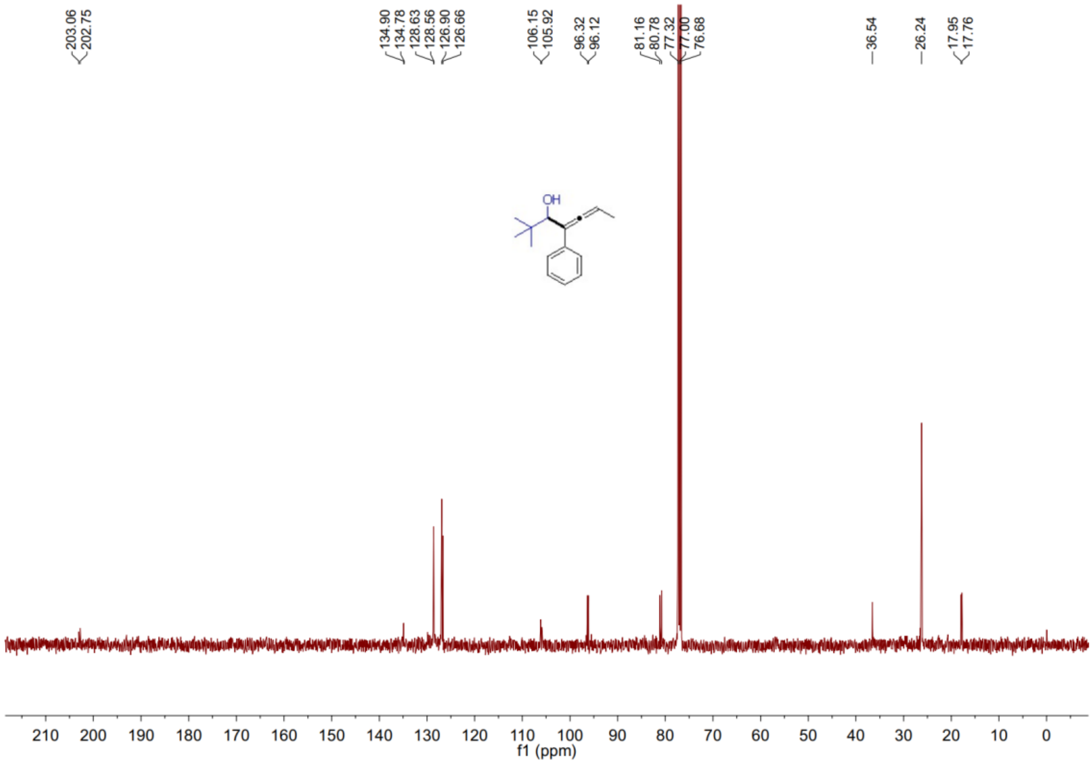

5al ^{1}H NMR

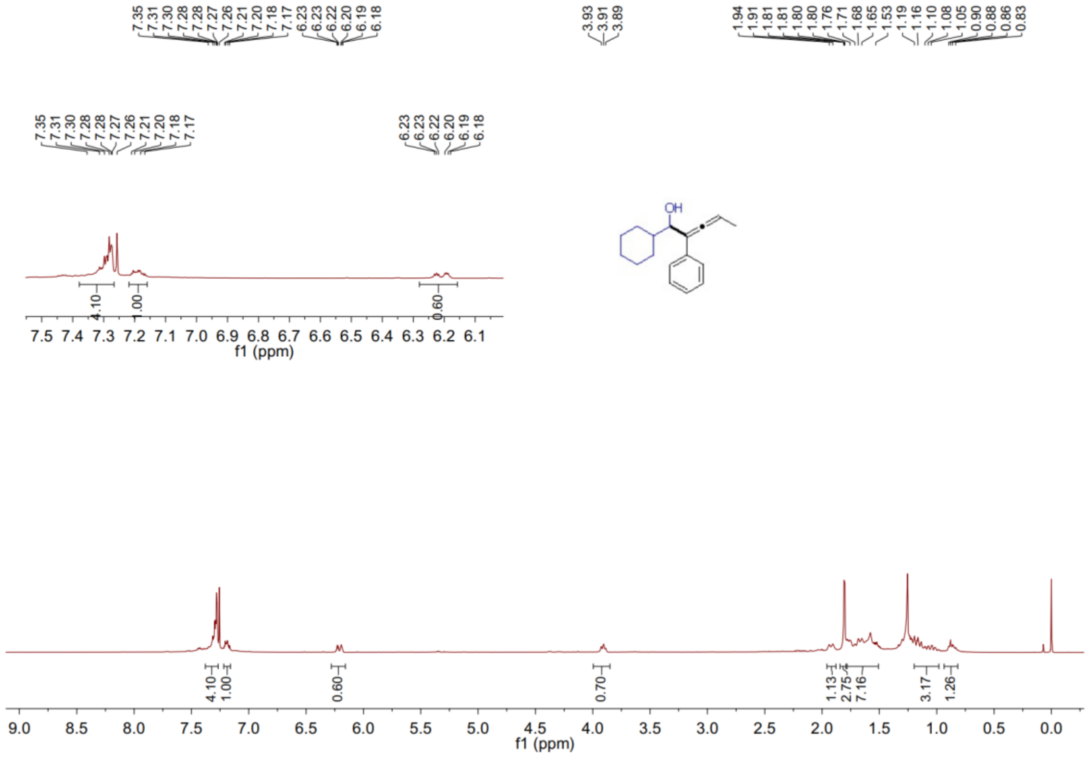

5al ^{13}C NMR

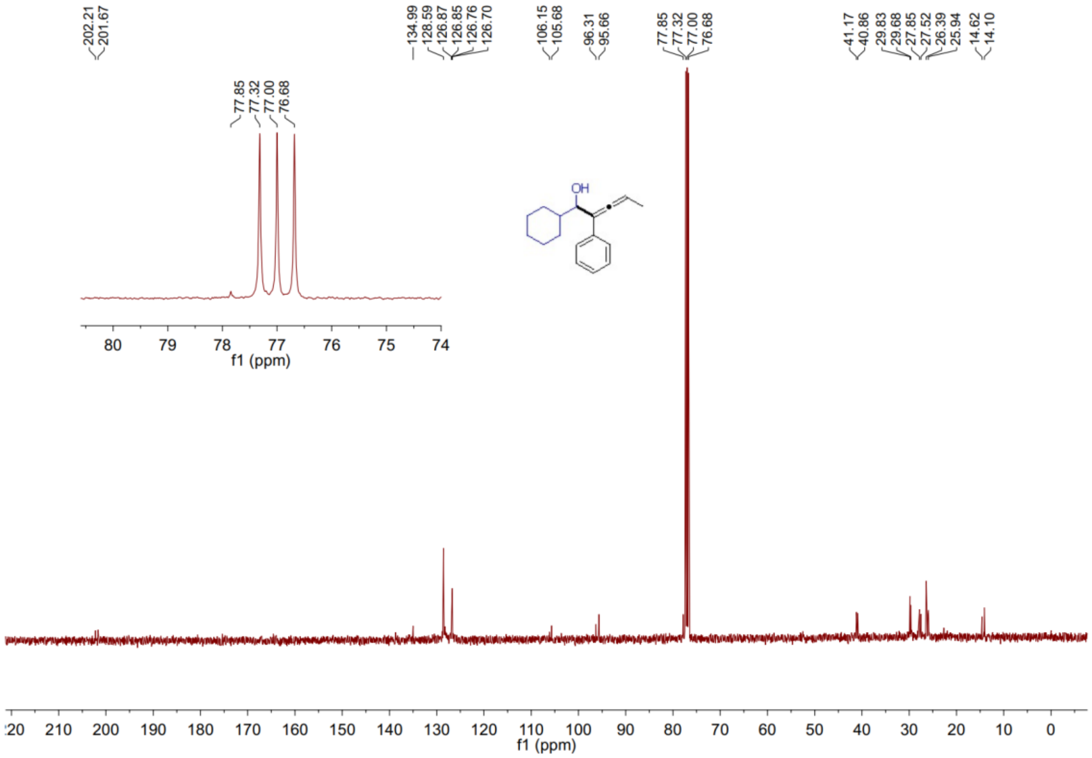

5am ^{1}H NMR

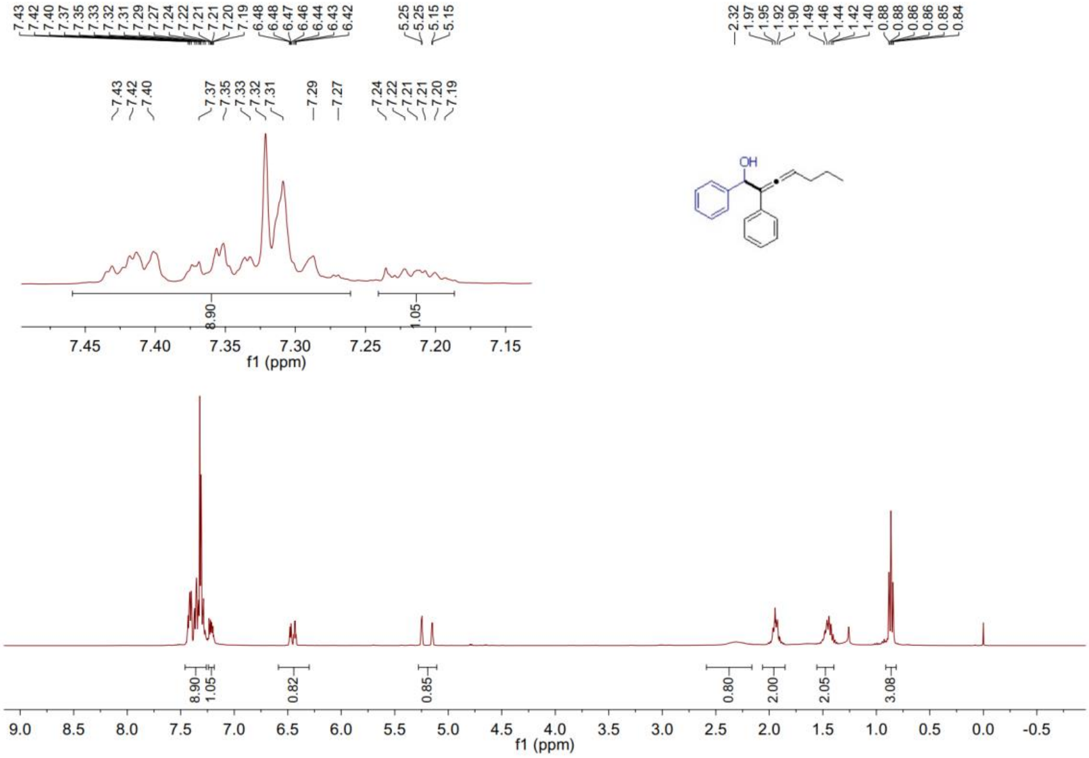

5am ^{13}C NMR

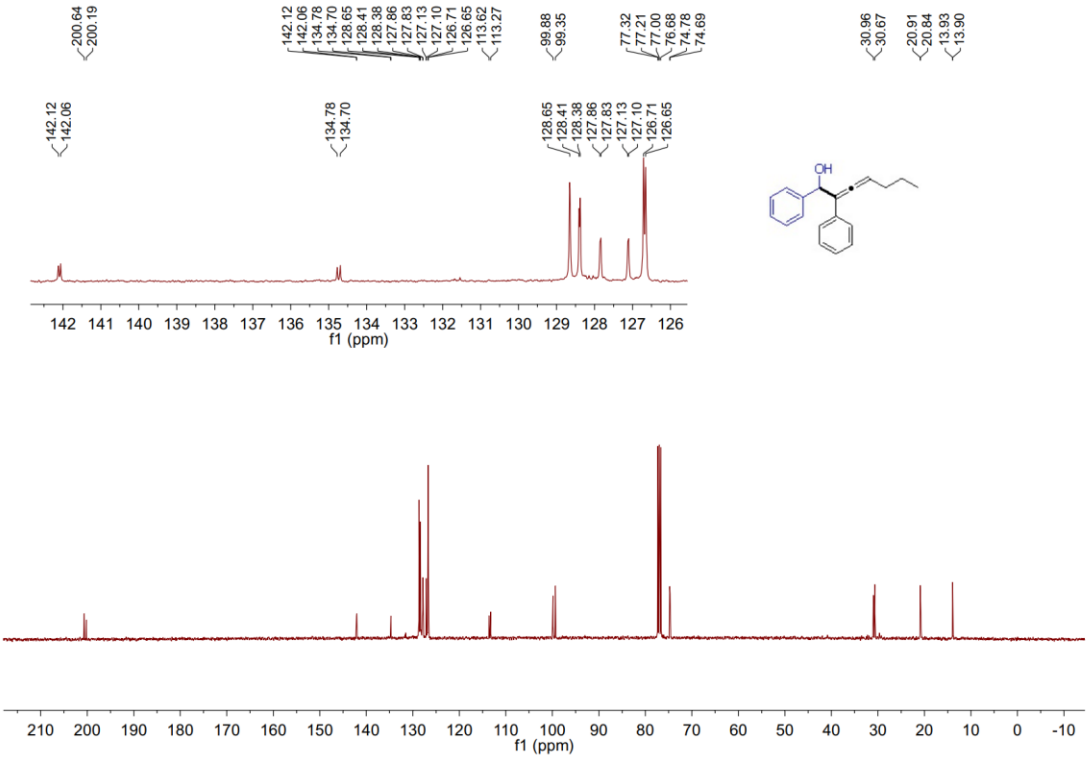

5an ^{1}H NMR

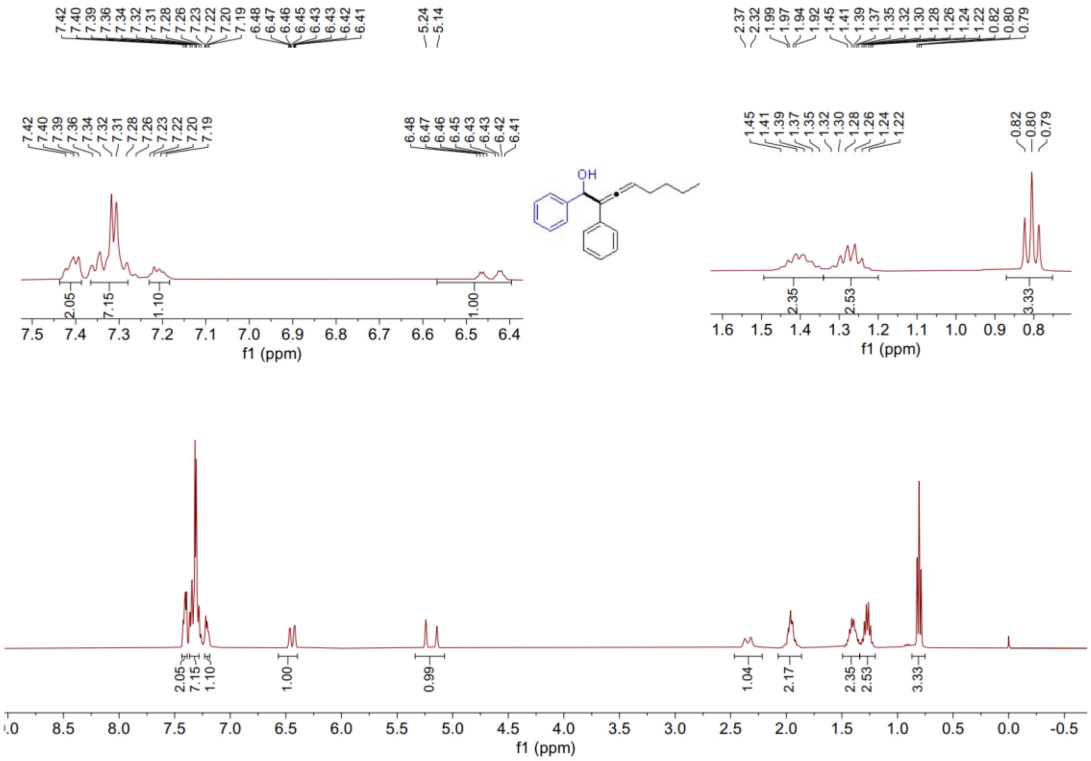

5an ^{13}C NMR

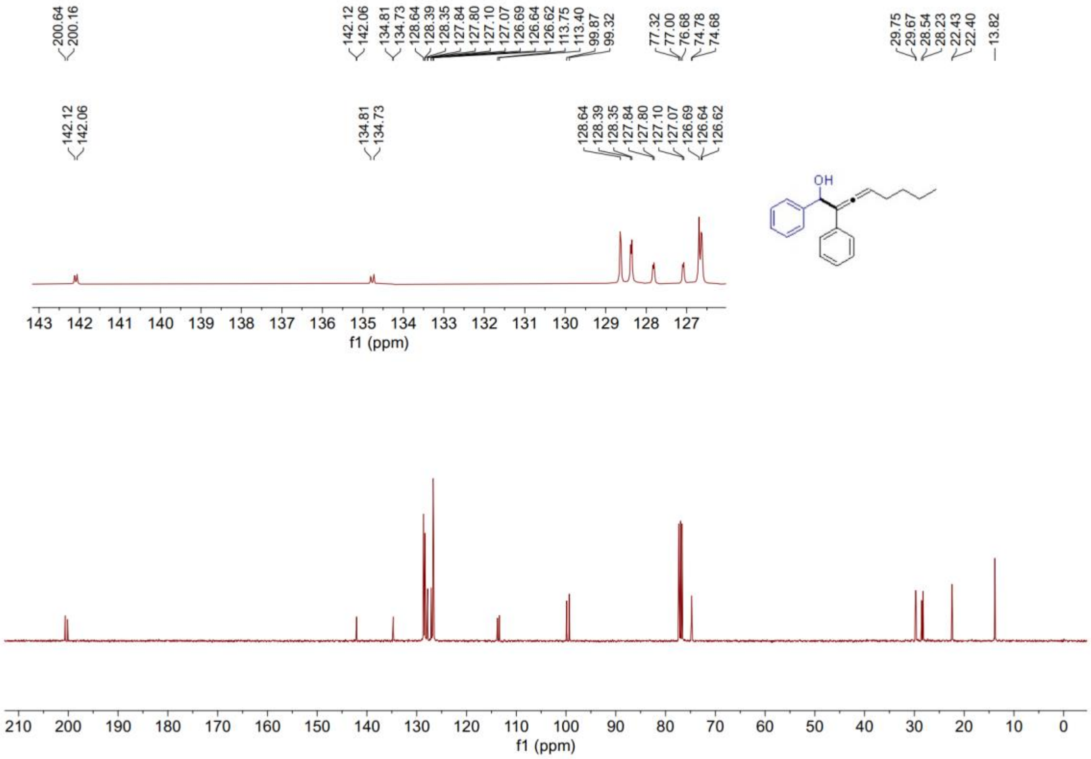

5ao ^{1}H NMR

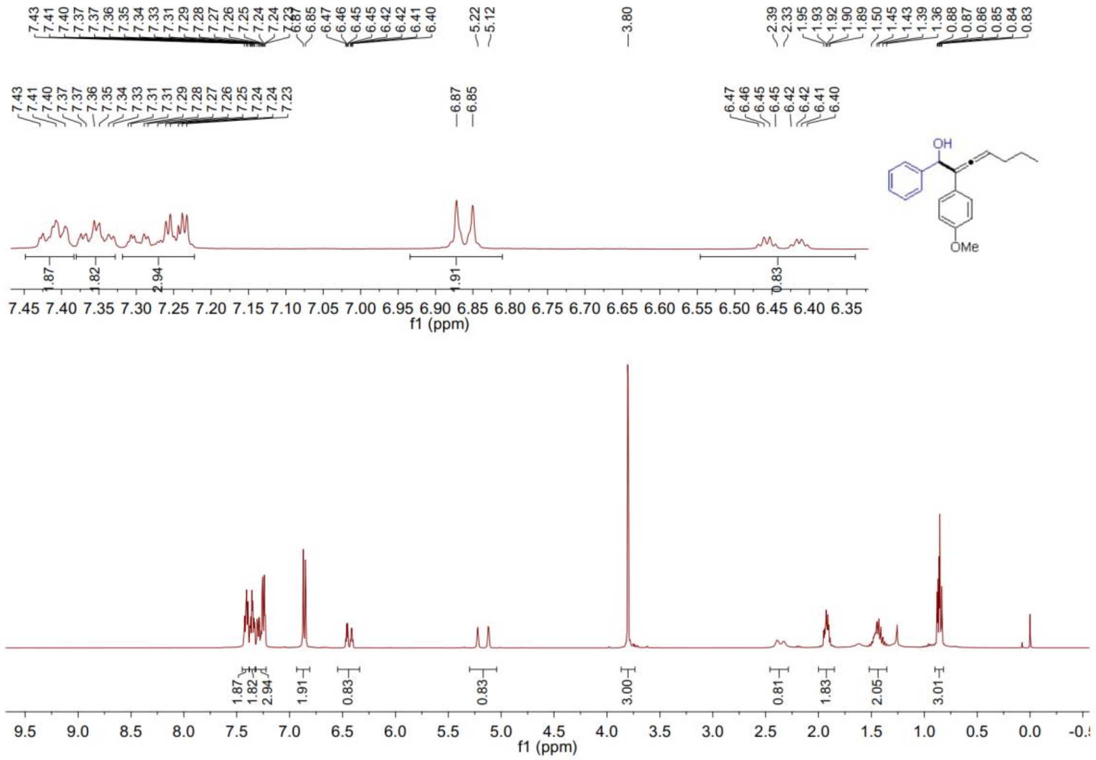

5ao ^{13}C NMR

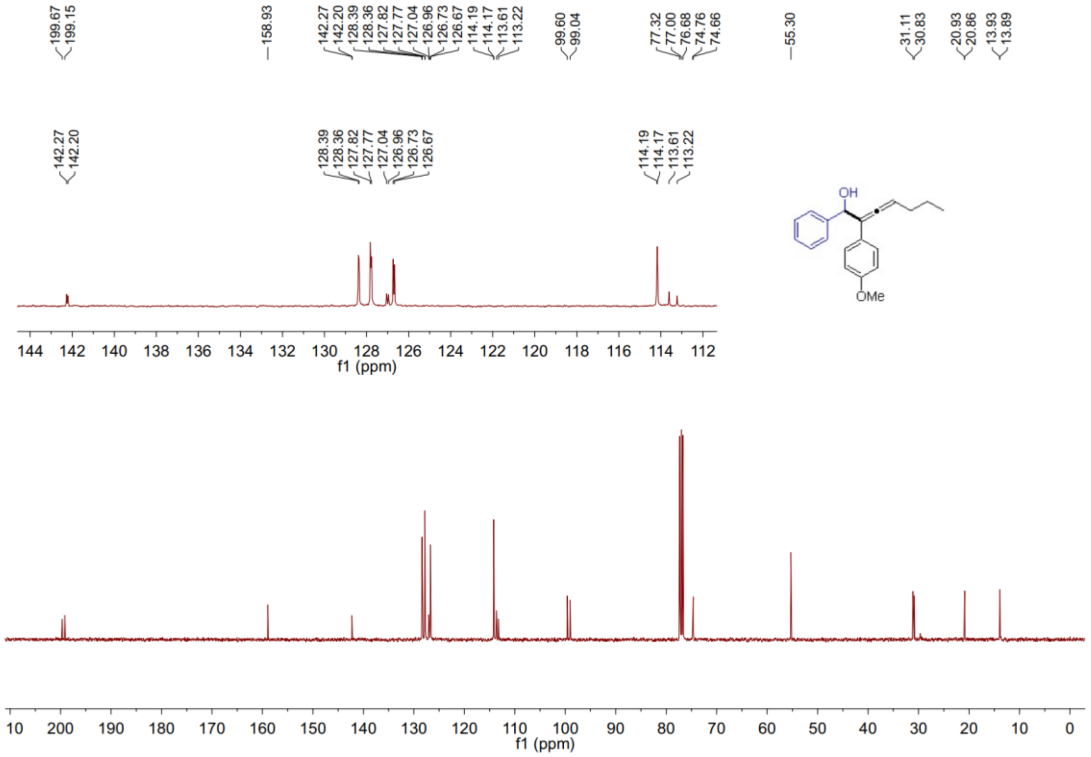

5ap ^{1}H NMR

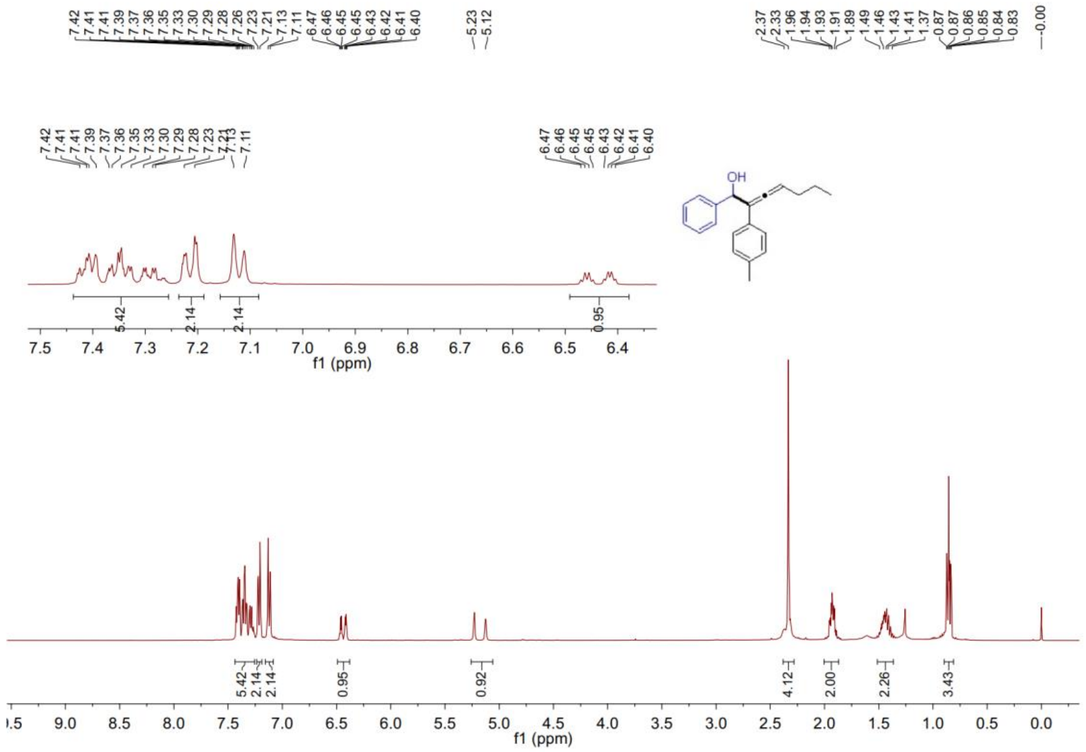

5ap ^{13}C NMR

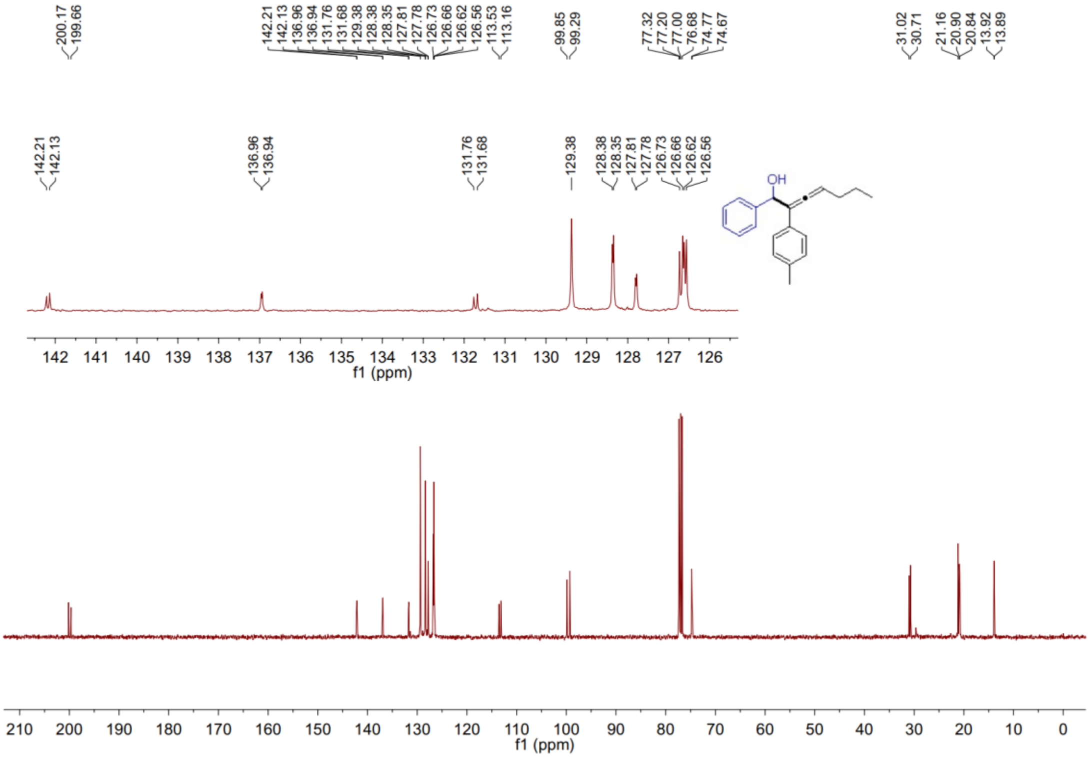

5aq ^{1}H NMR

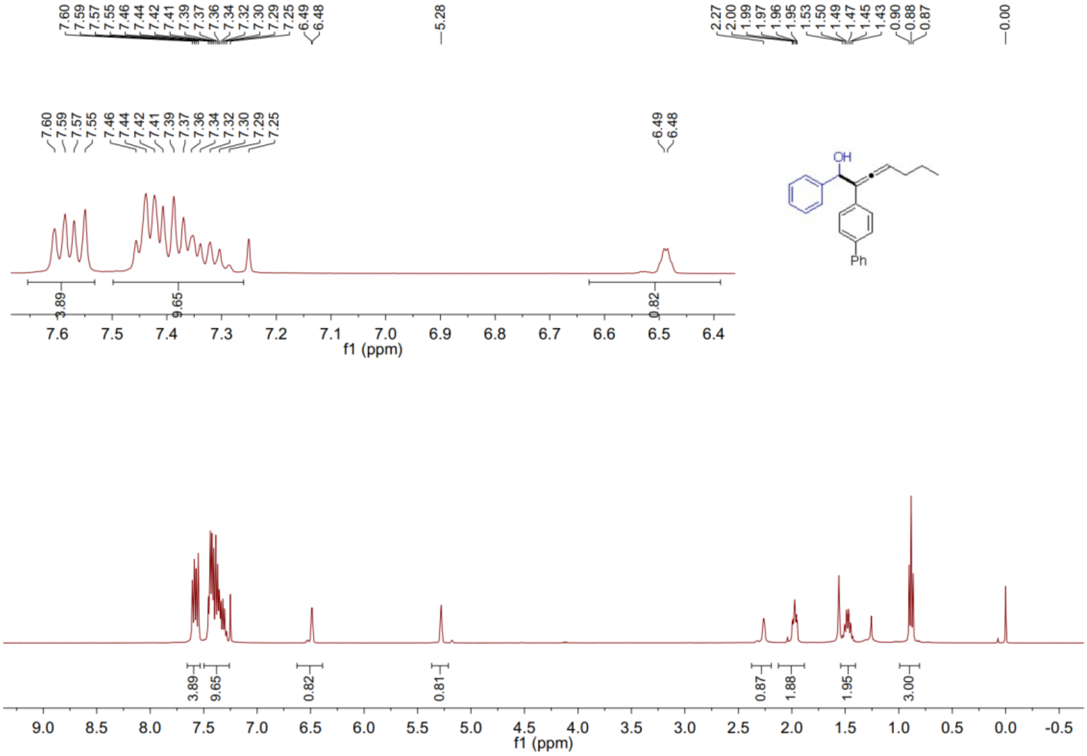

5aq ^{13}C NMR

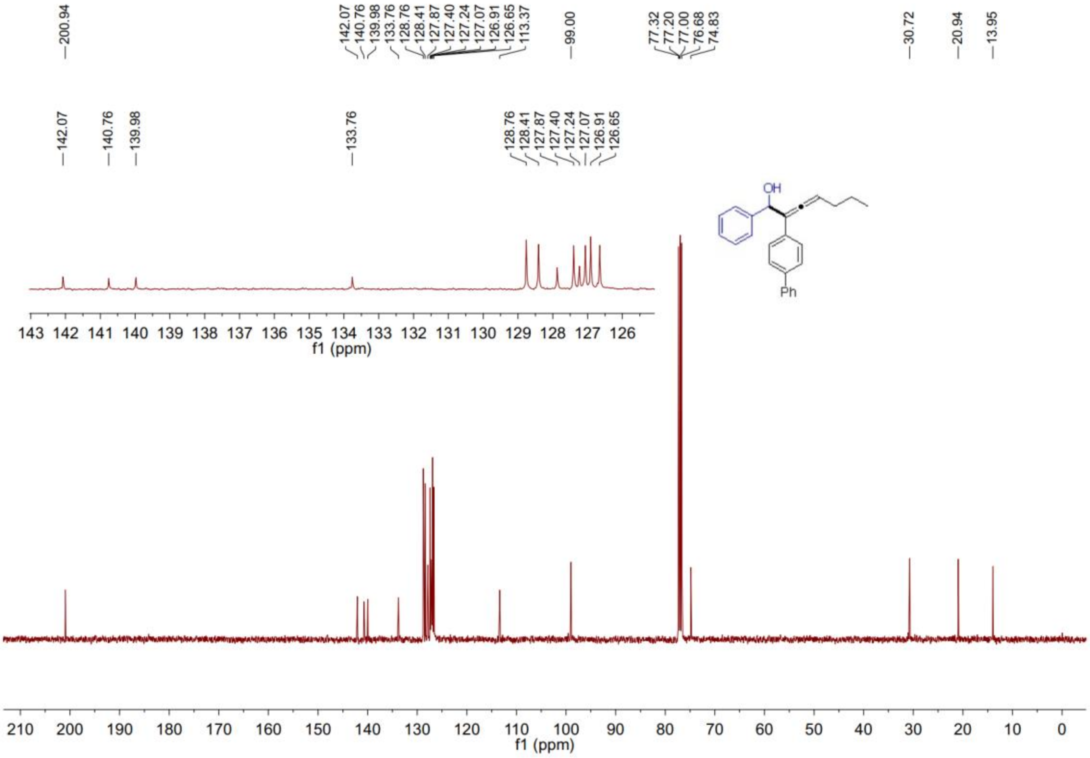

5ar ^{1}H NMR

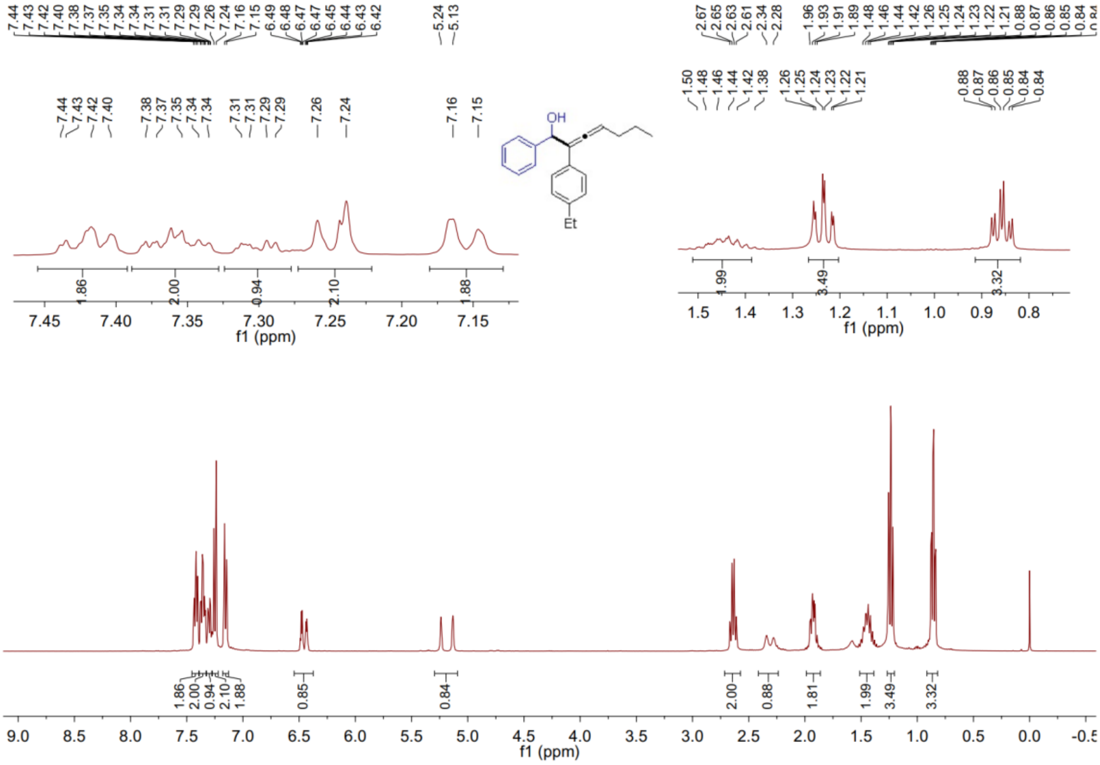

5ar ^{13}C NMR

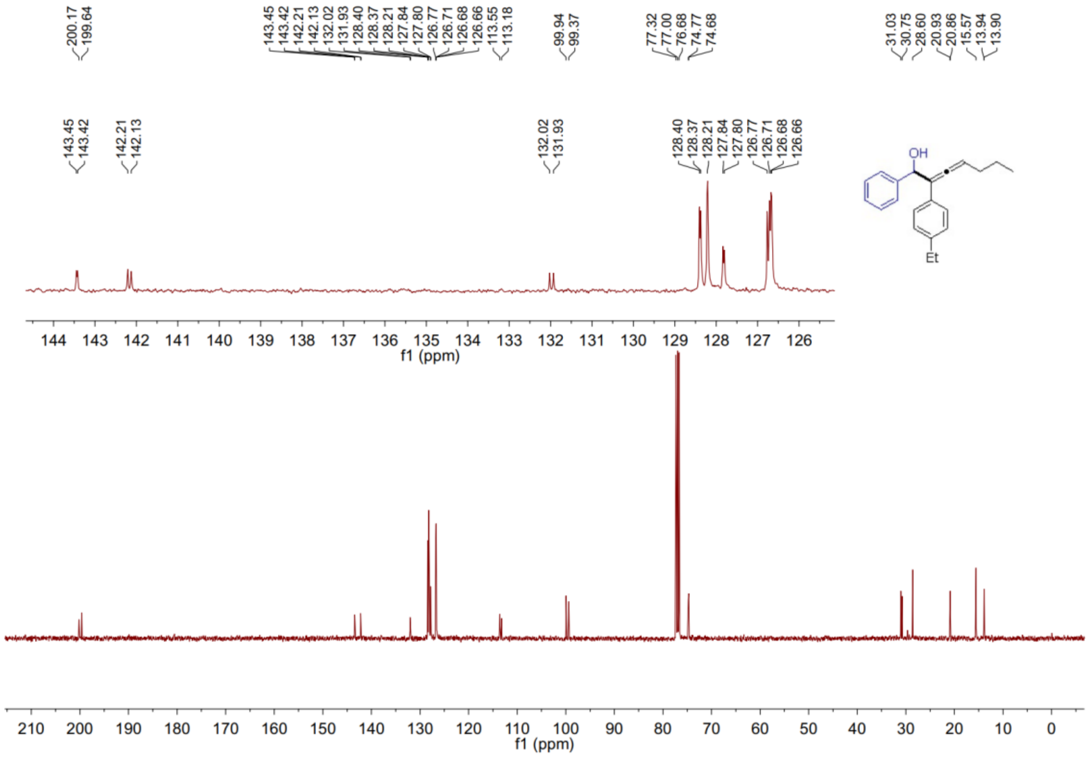

5as ^{1}H NMR

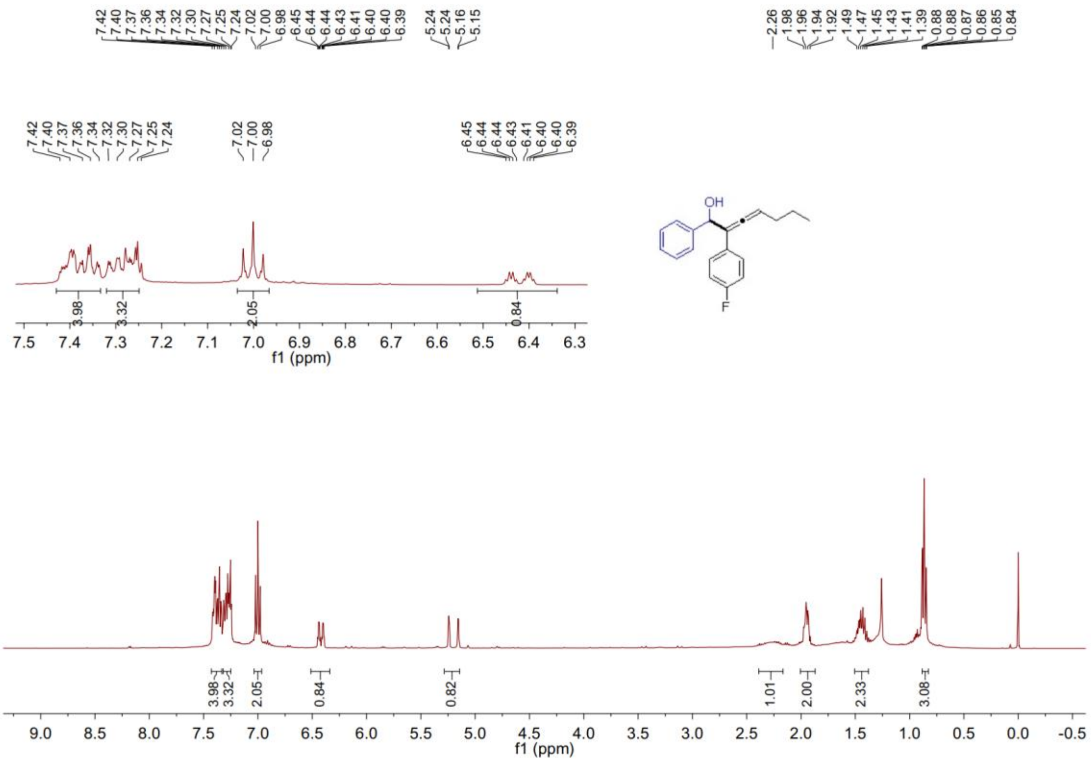

5as ^{13}C NMR

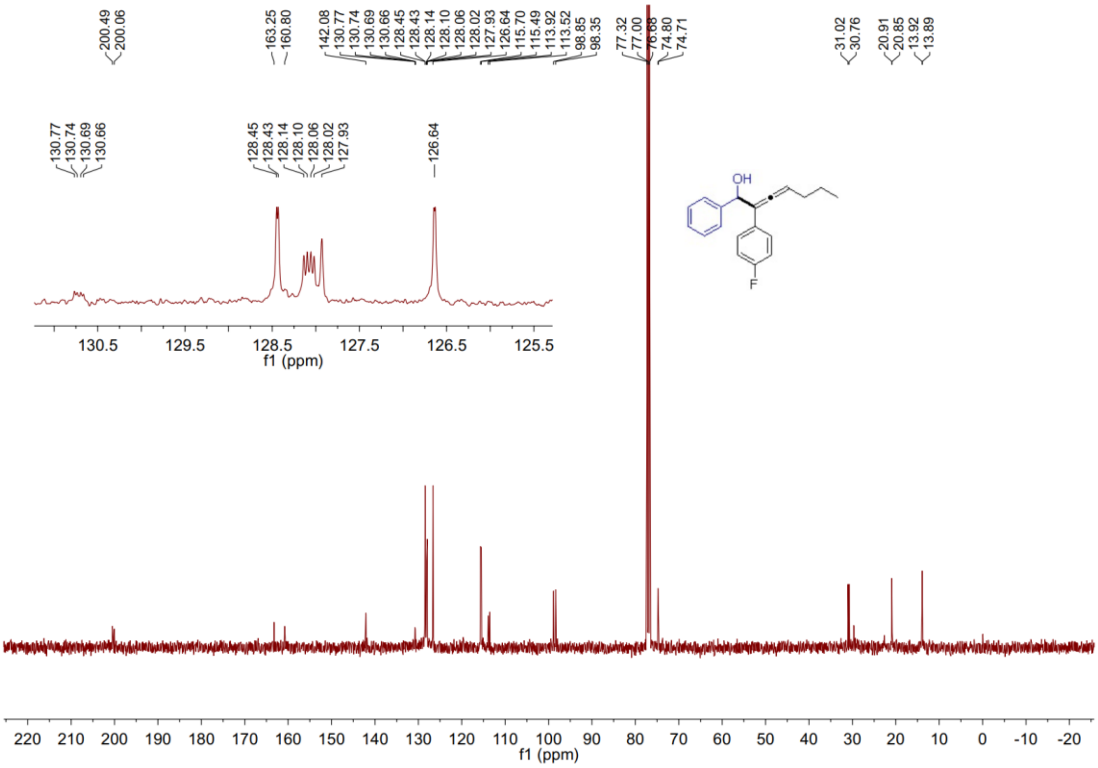

5at ^{1}H NMR

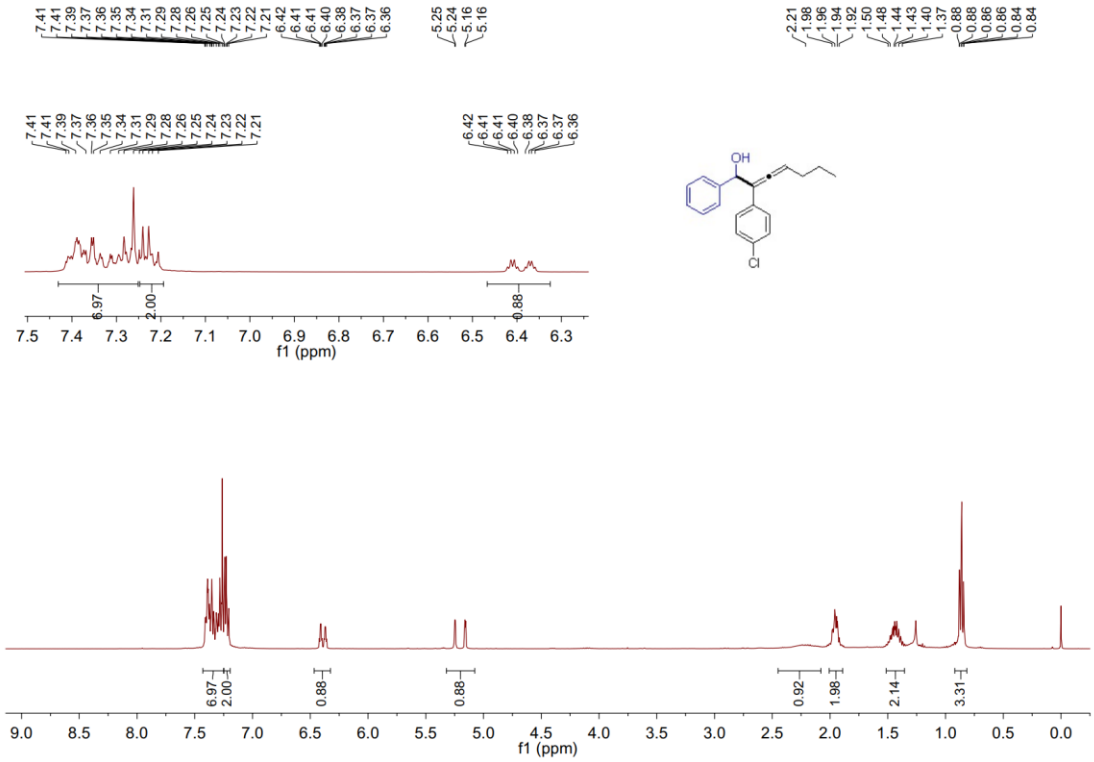

5at ^{13}C NMR

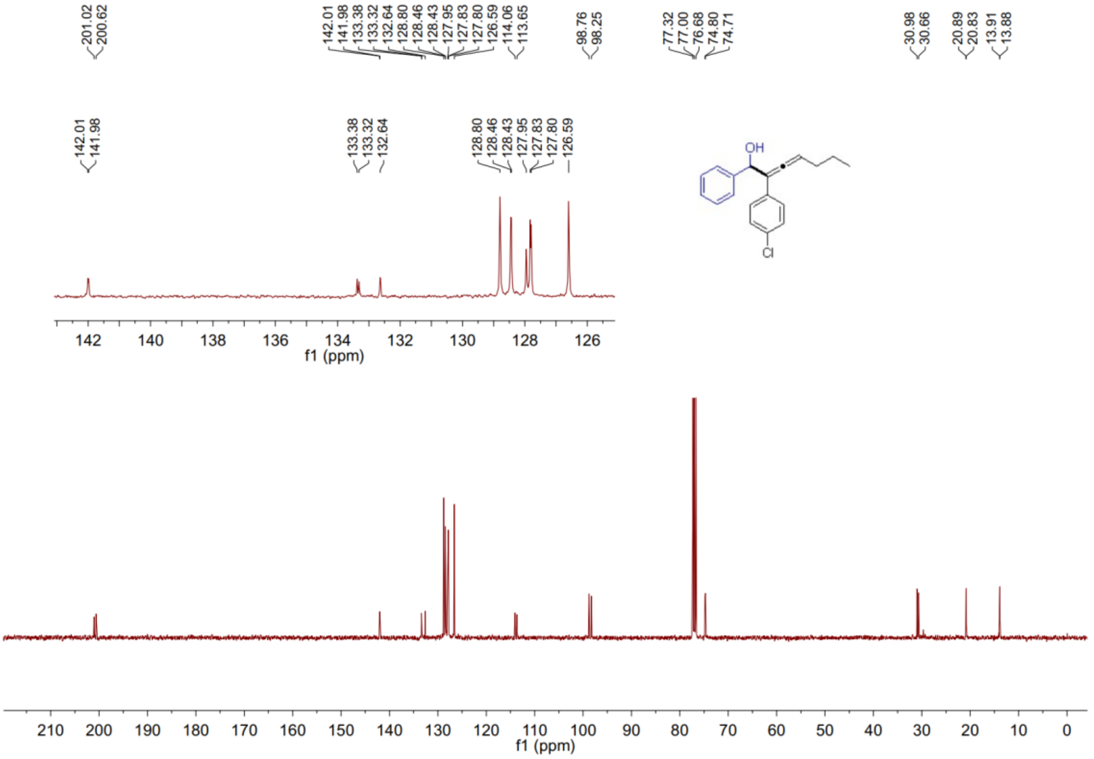

5au ^{1}H NMR

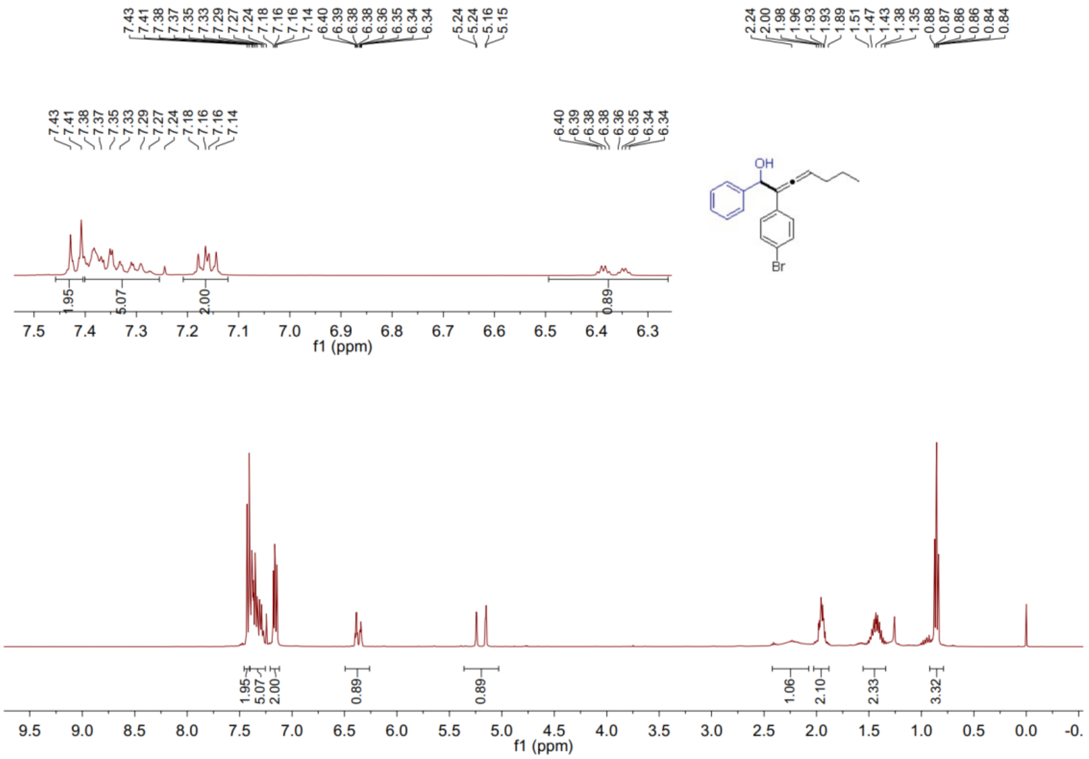

5au ^{13}C NMR

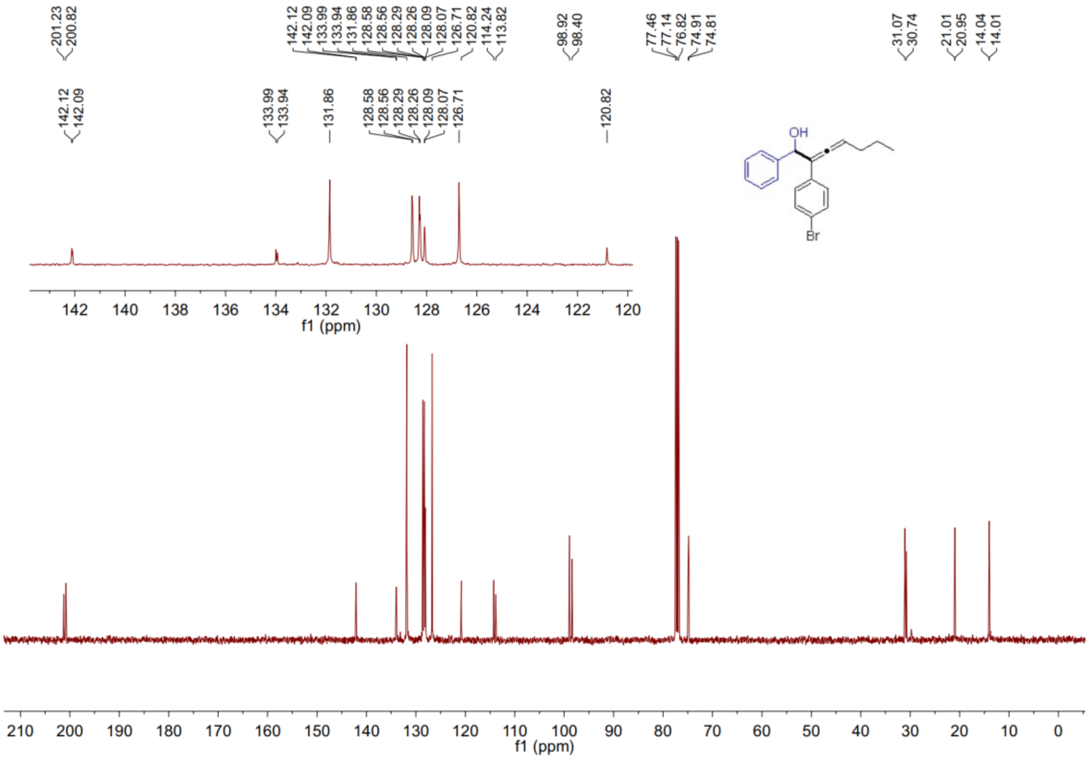

5av ^{1}H NMR

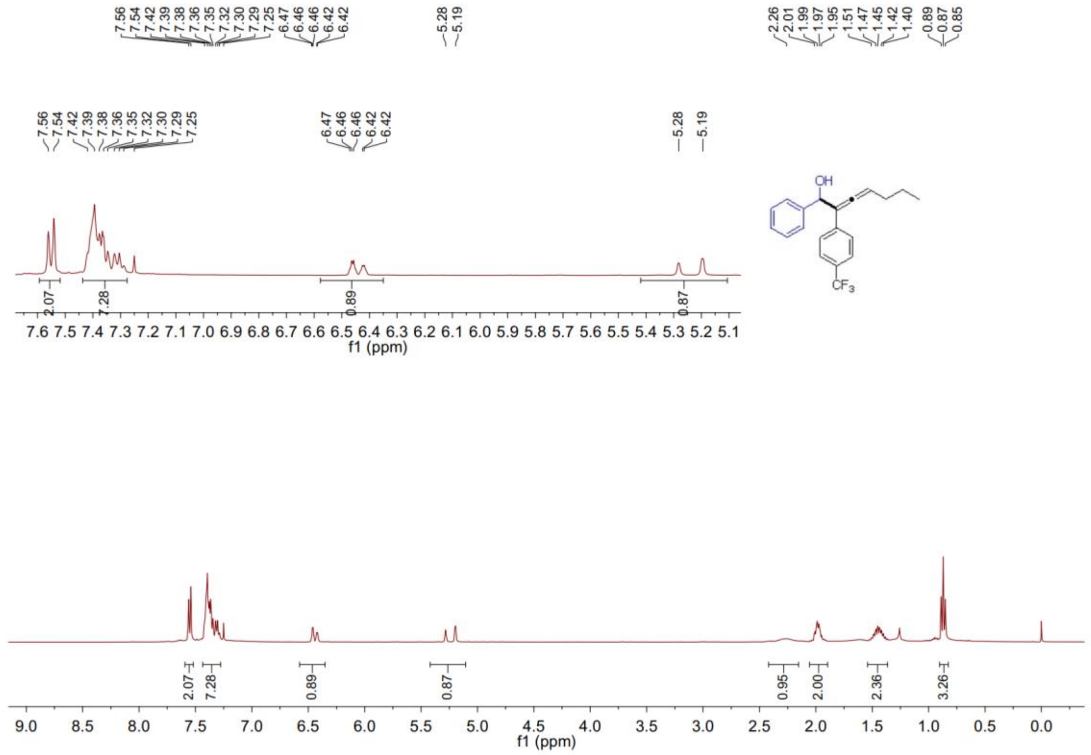

5av ^{13}C NMR

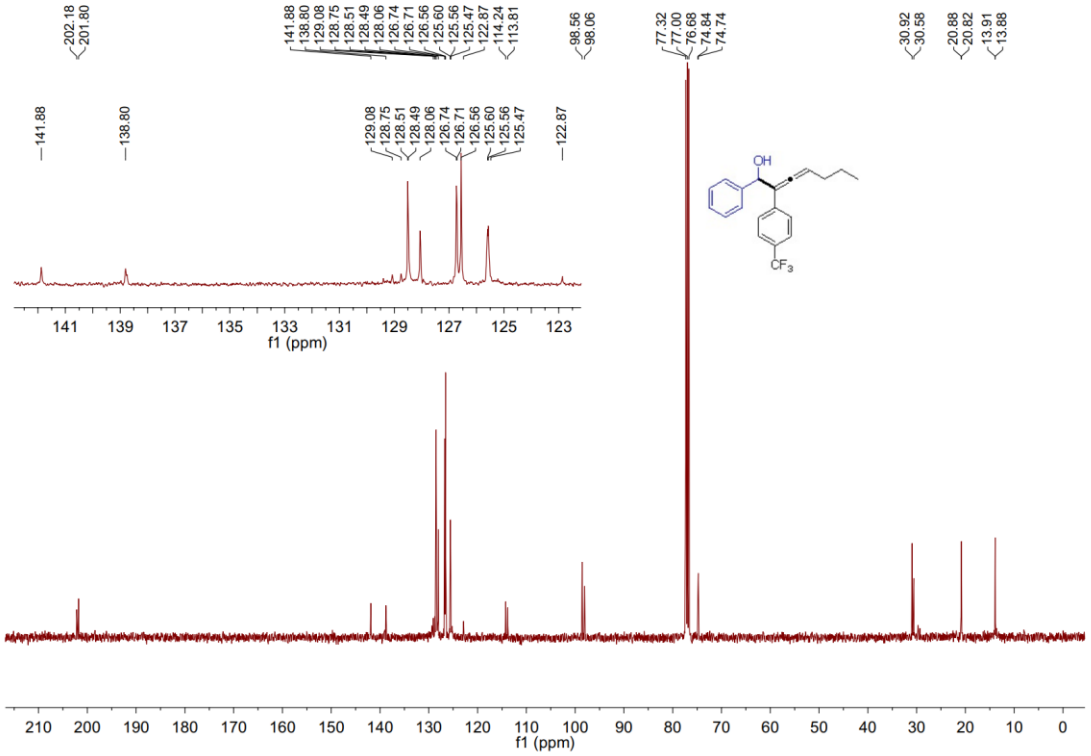

5aw ^{1}H NMR

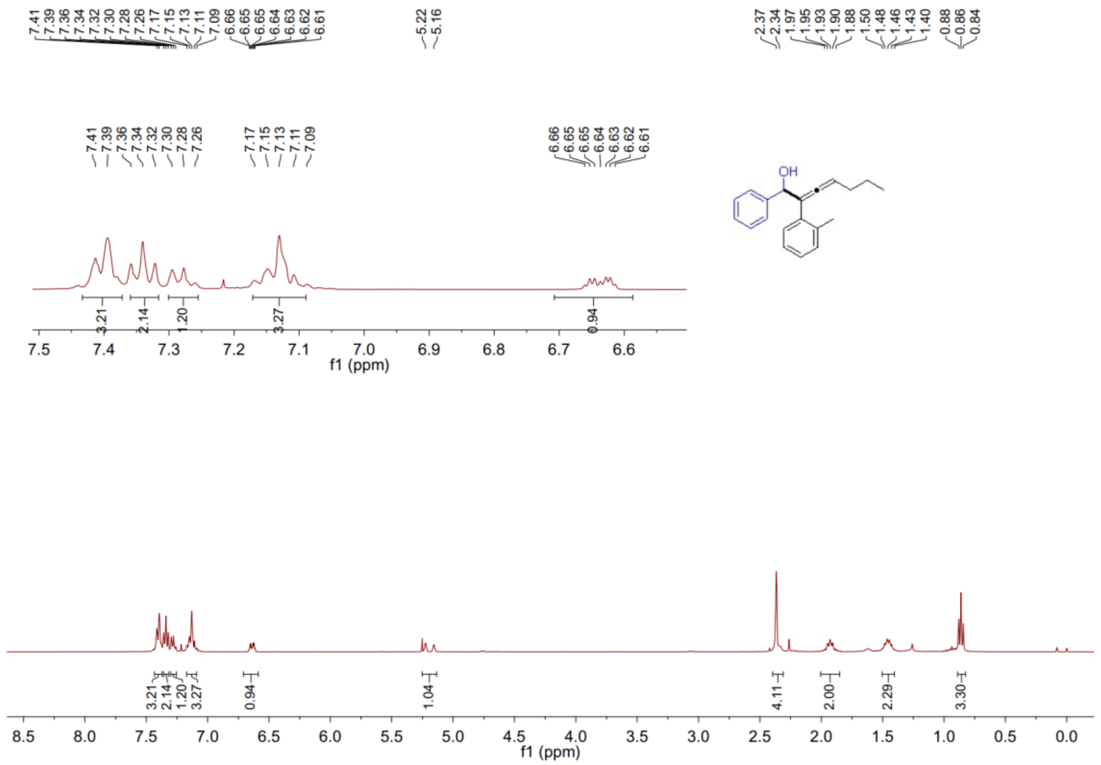

5aw ^{13}C NMR

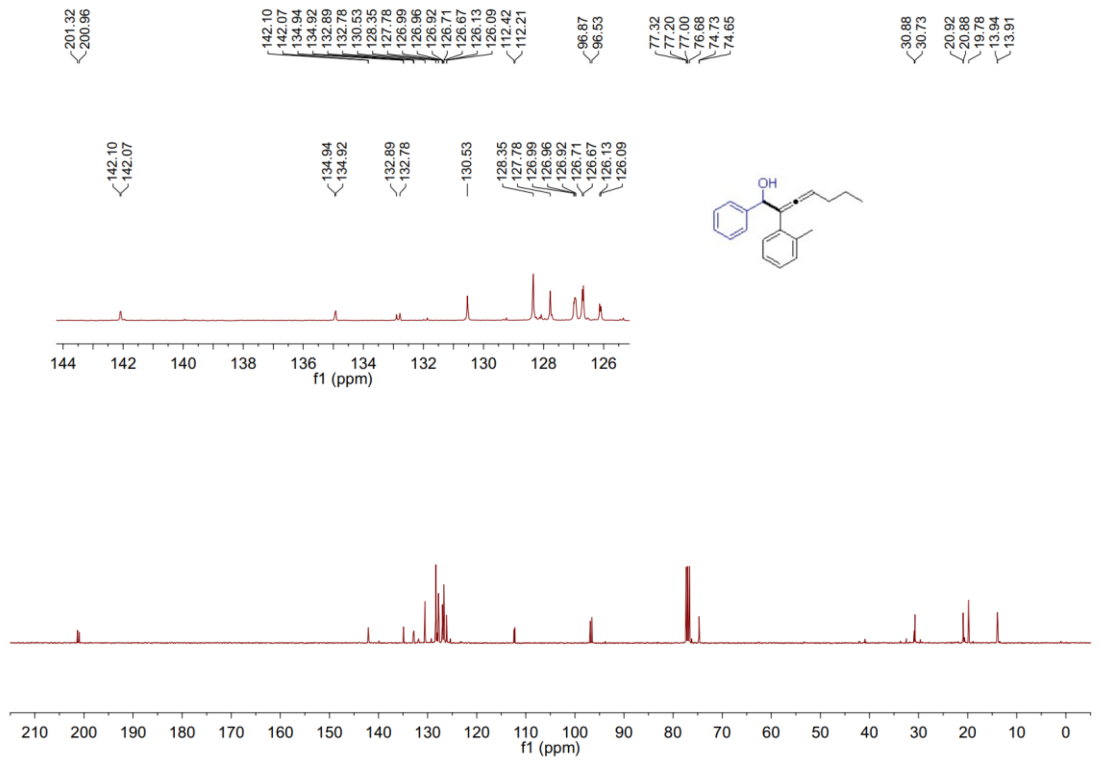

5ax ^{1}H NMR

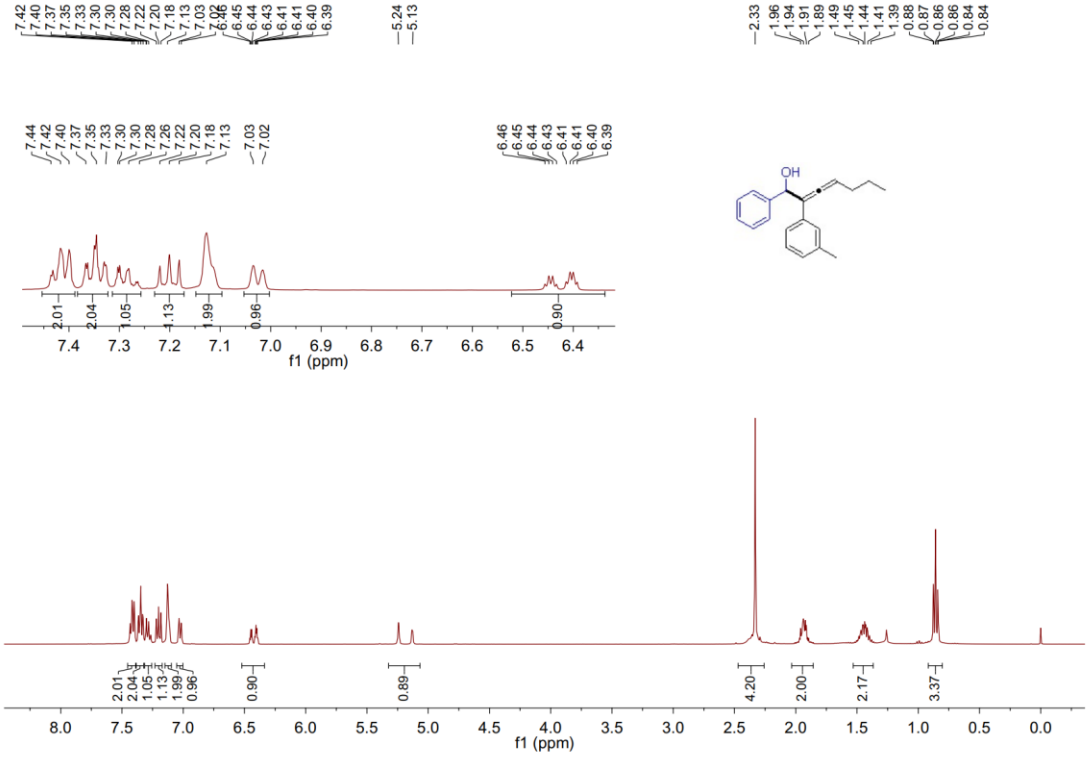

5ax ^{13}C NMR

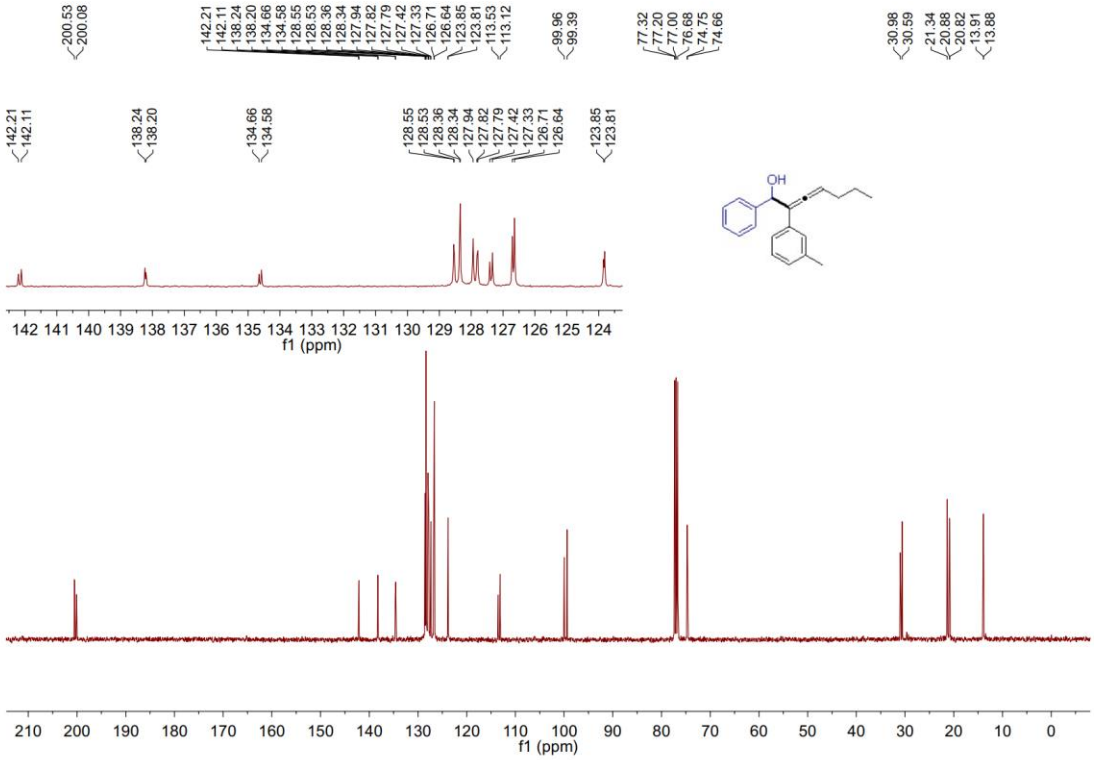

5ay ^{1}H NMR

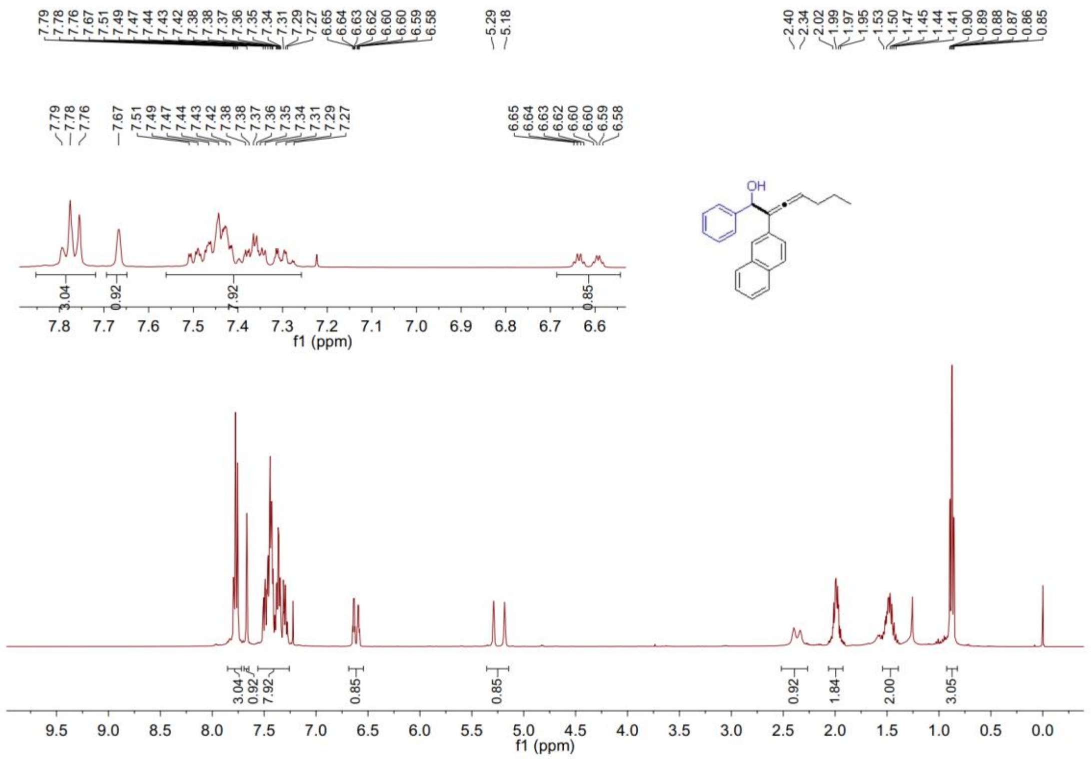

5ay ^{13}C NMR

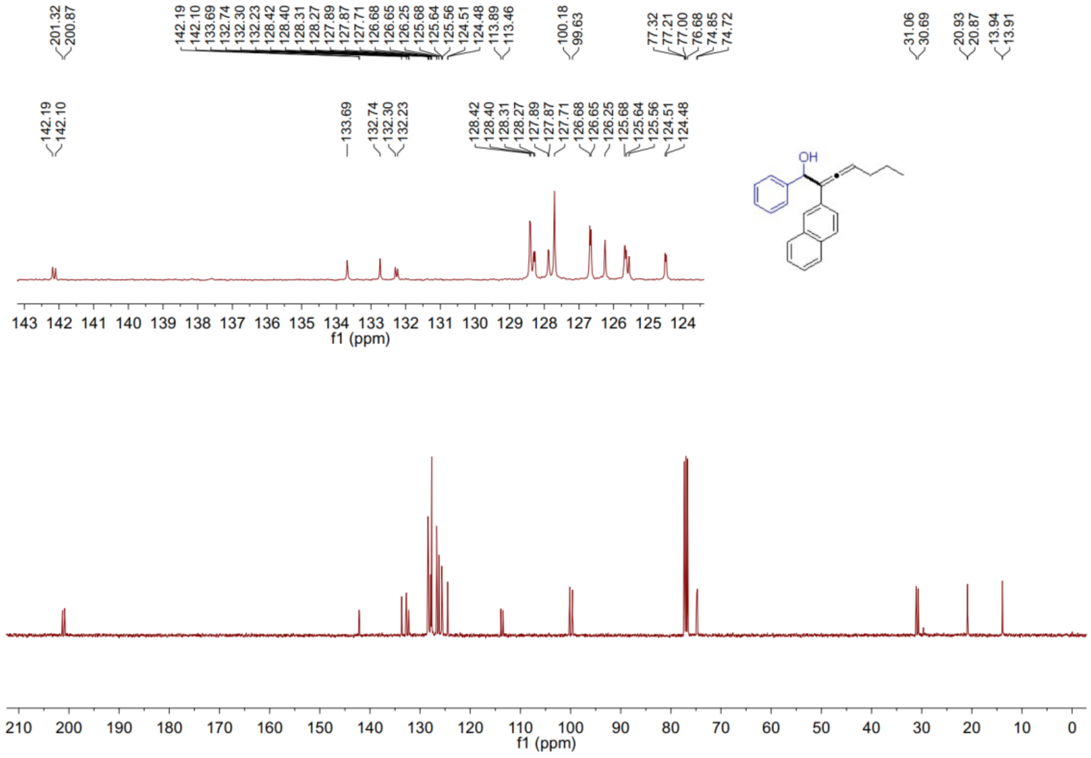

5az ^{1}H NMR

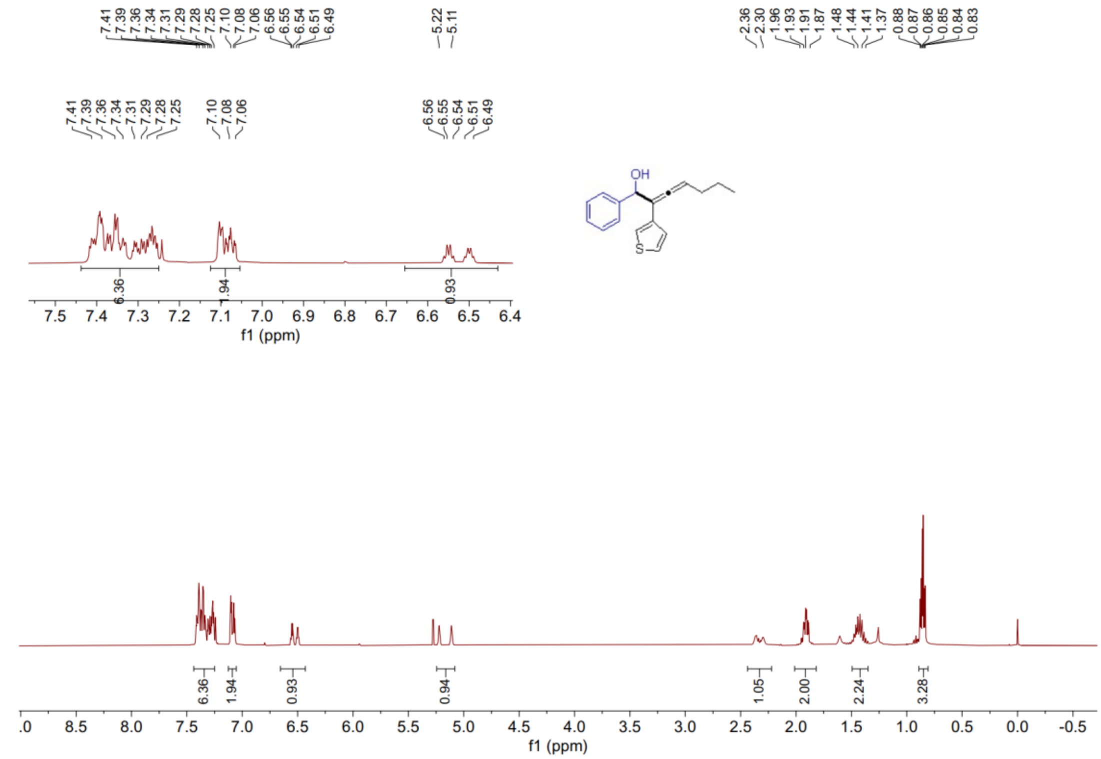

5az ^{13}C NMR

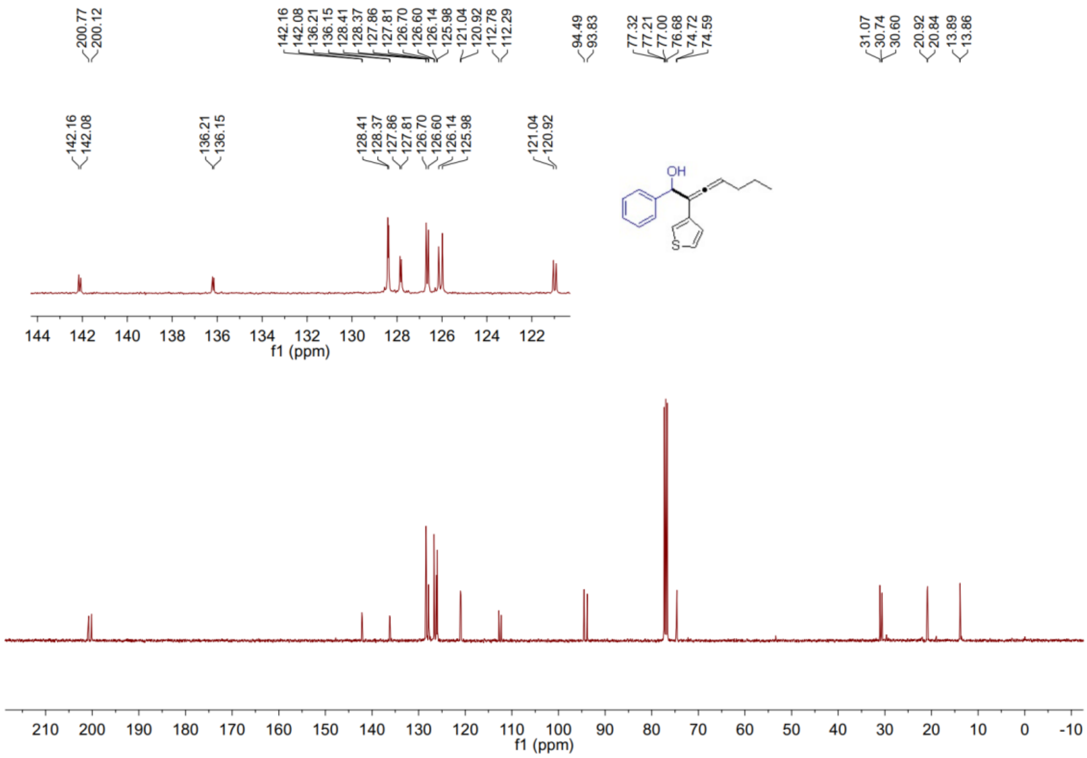

5ba ^{1}H NMR

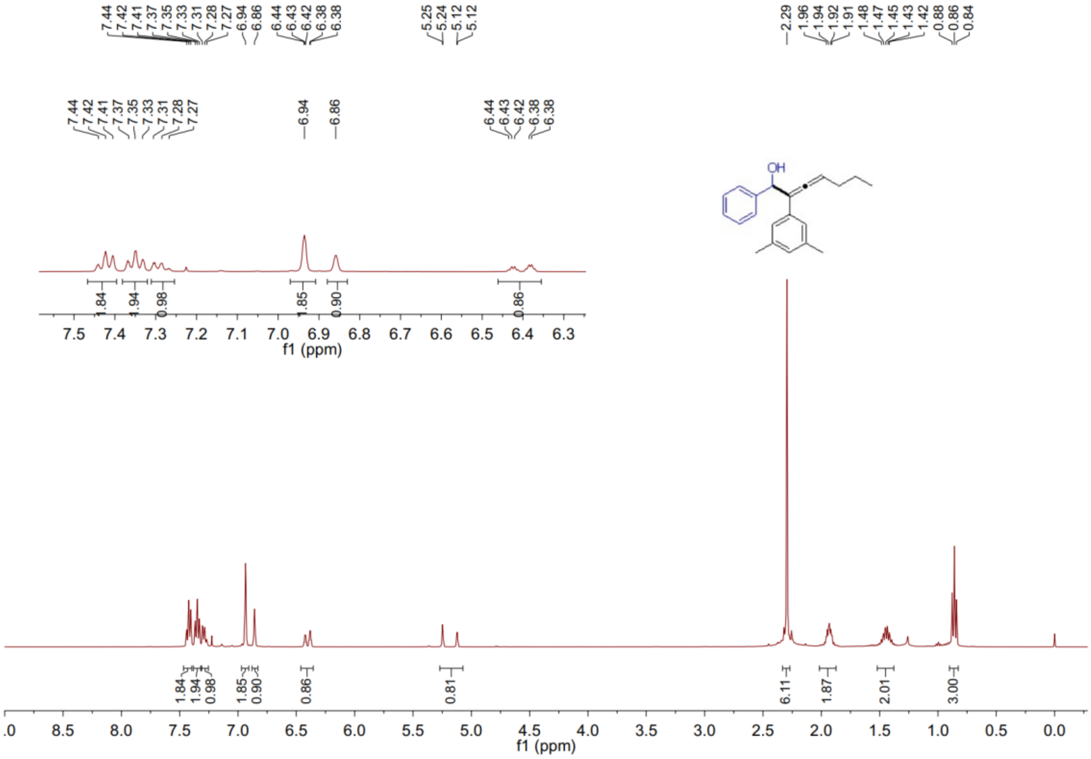

5ba ^{13}C NMR

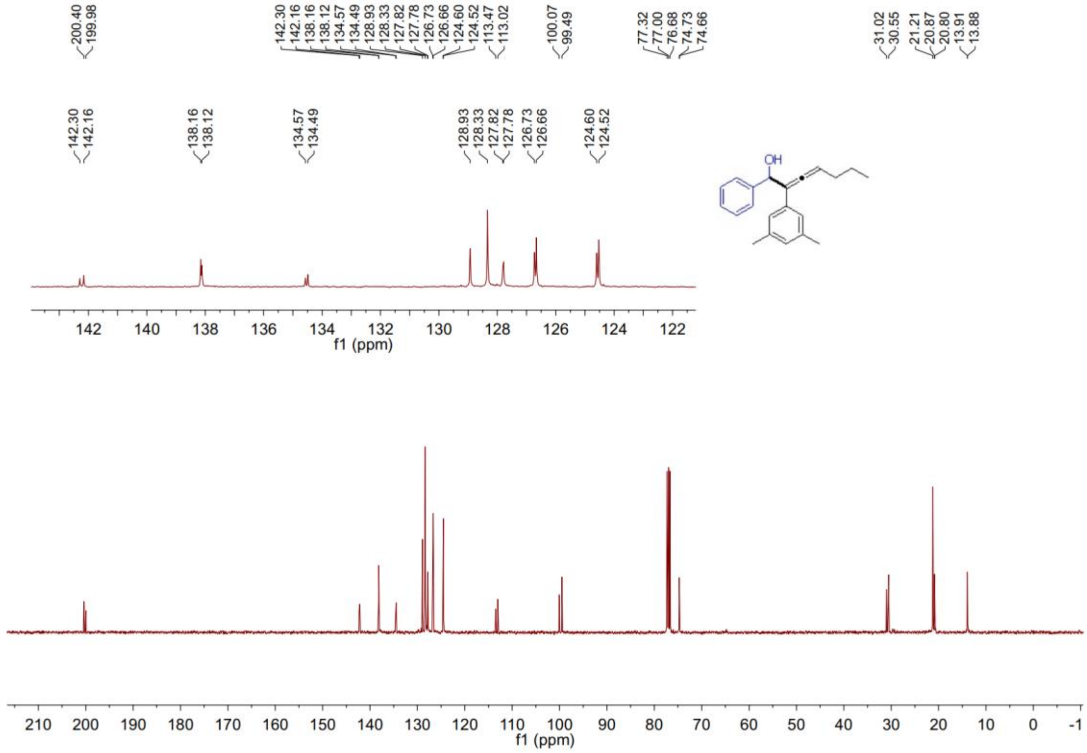

Mixture of 5bb, 5bc ^{1}H NMR

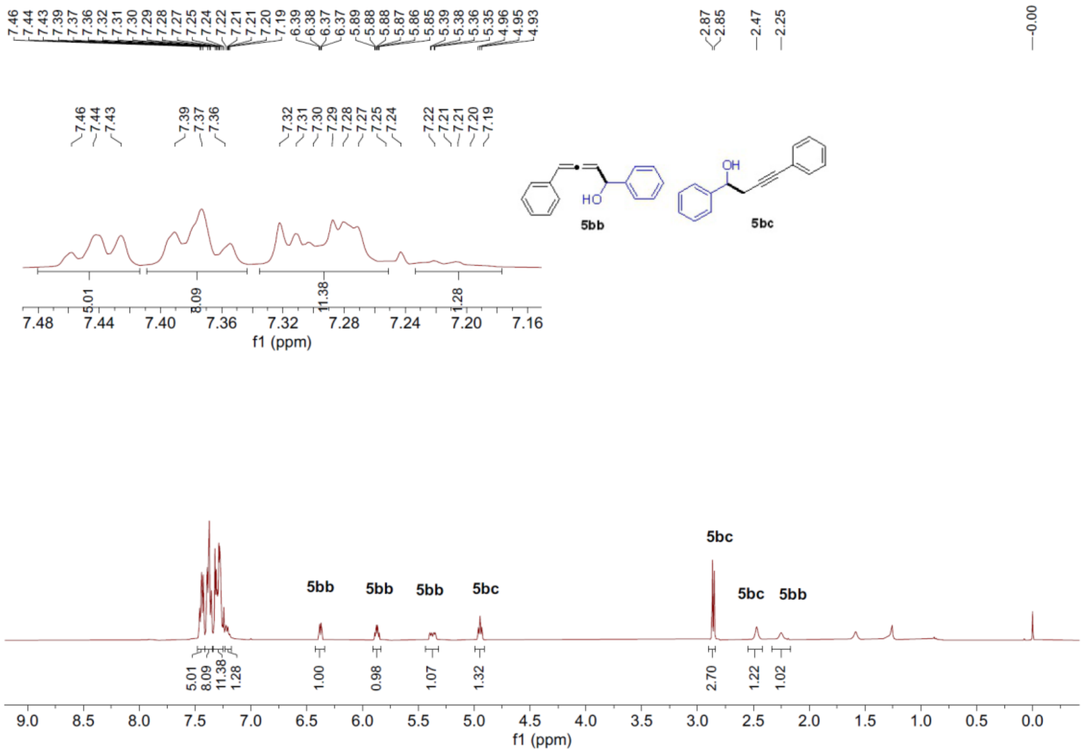

Mixture of 5bb, 5bc ^{13}C NMR

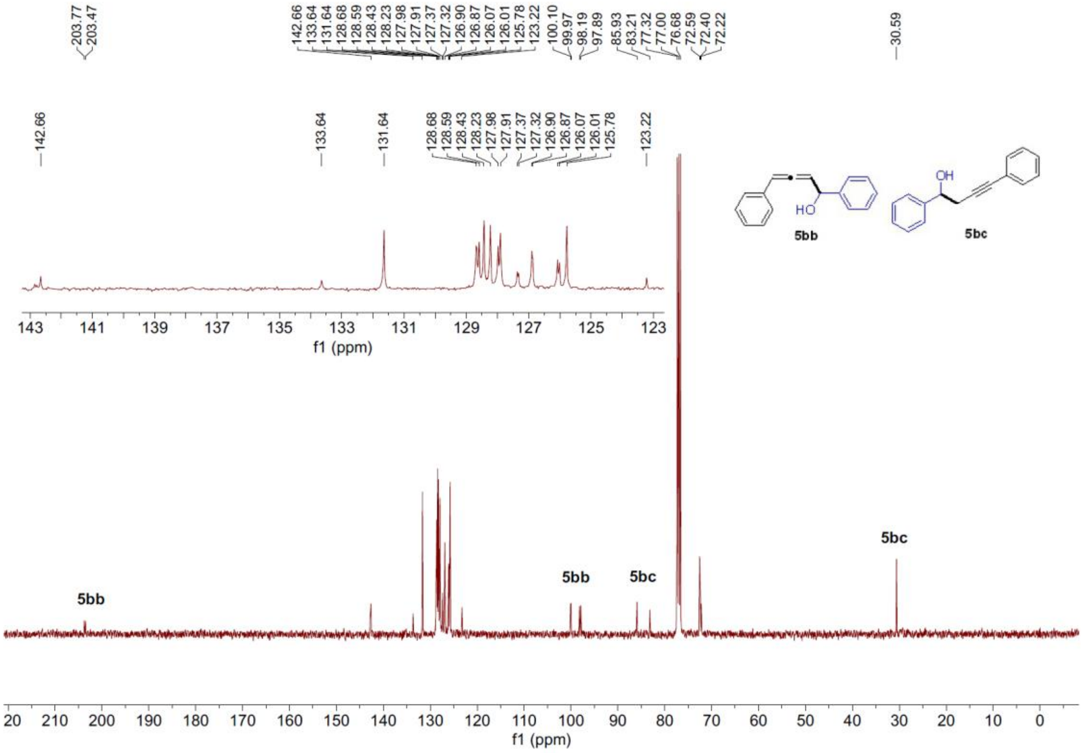

5bd ^{1}H NMR

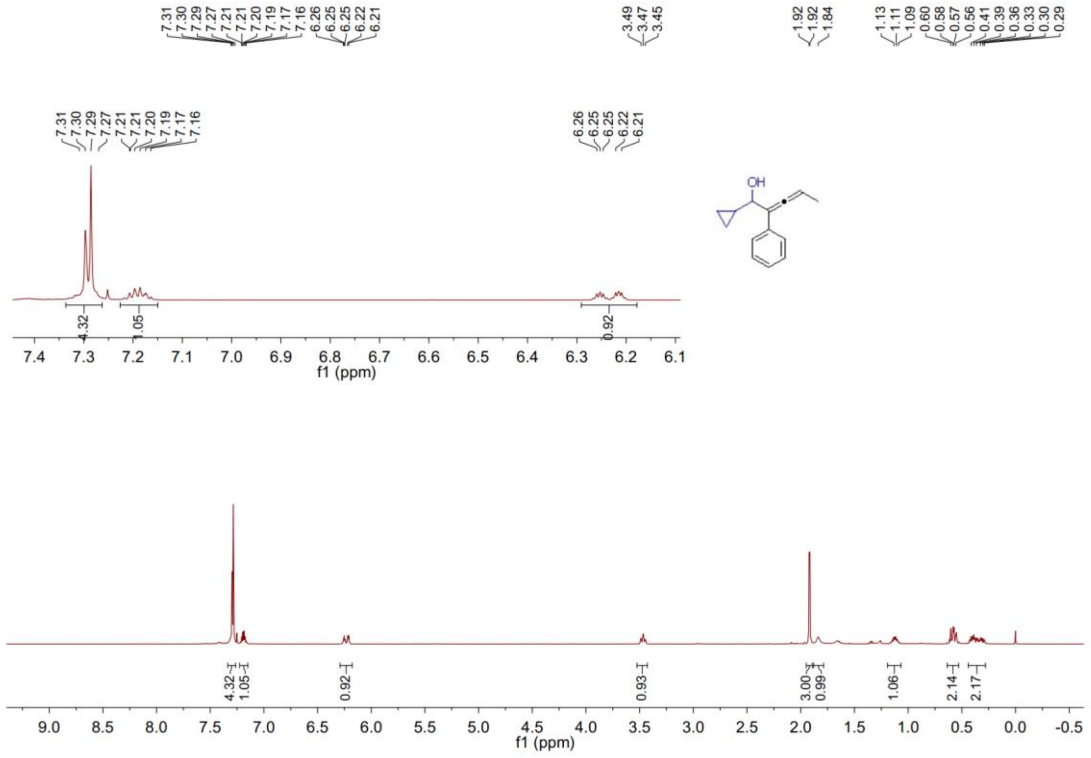

5bd ^{13}C NMR

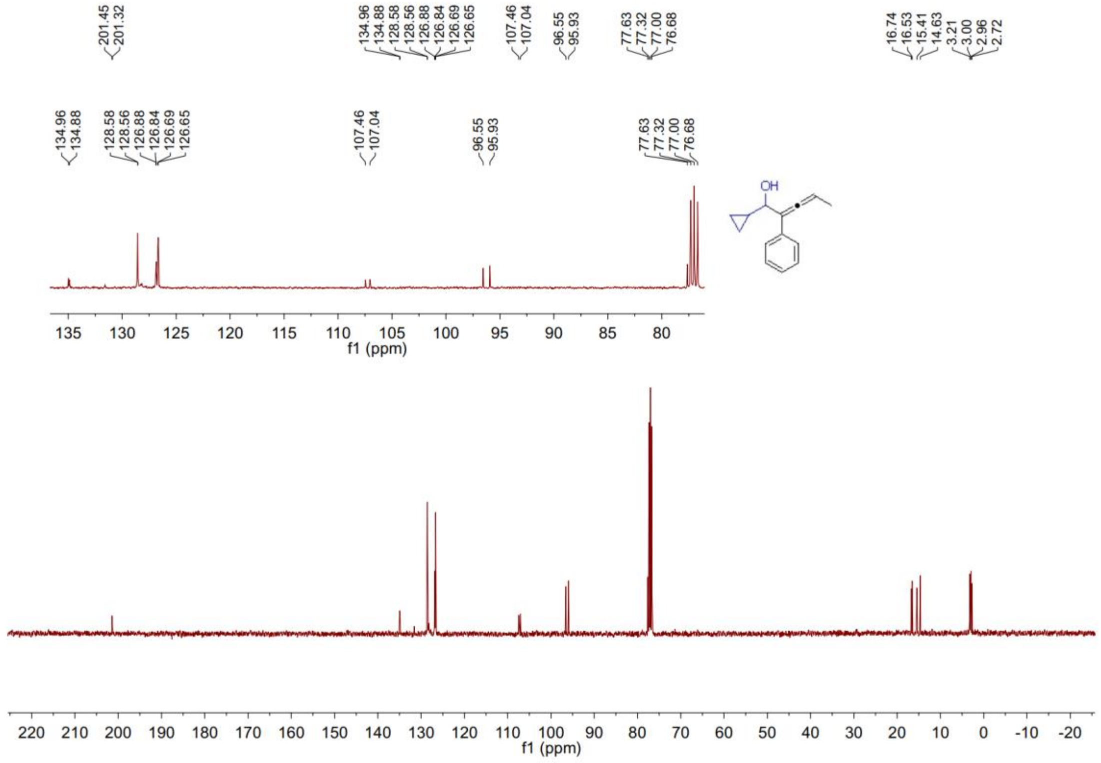

5be ^{1}H NMR

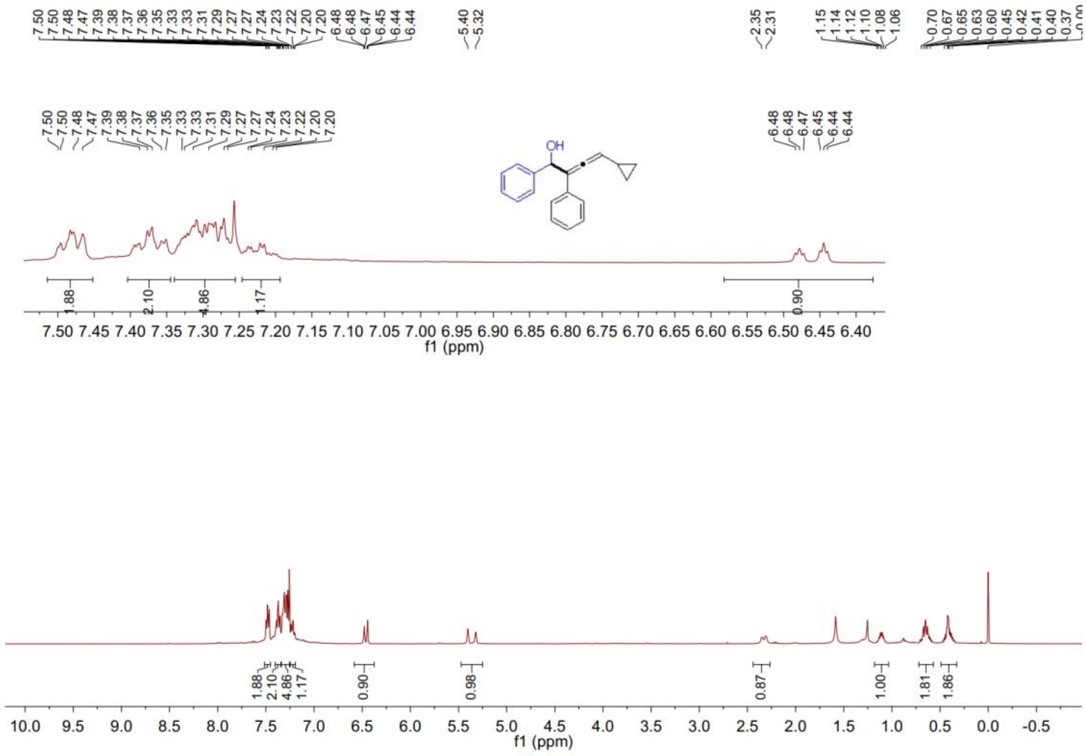

5be ^{13}C NMR

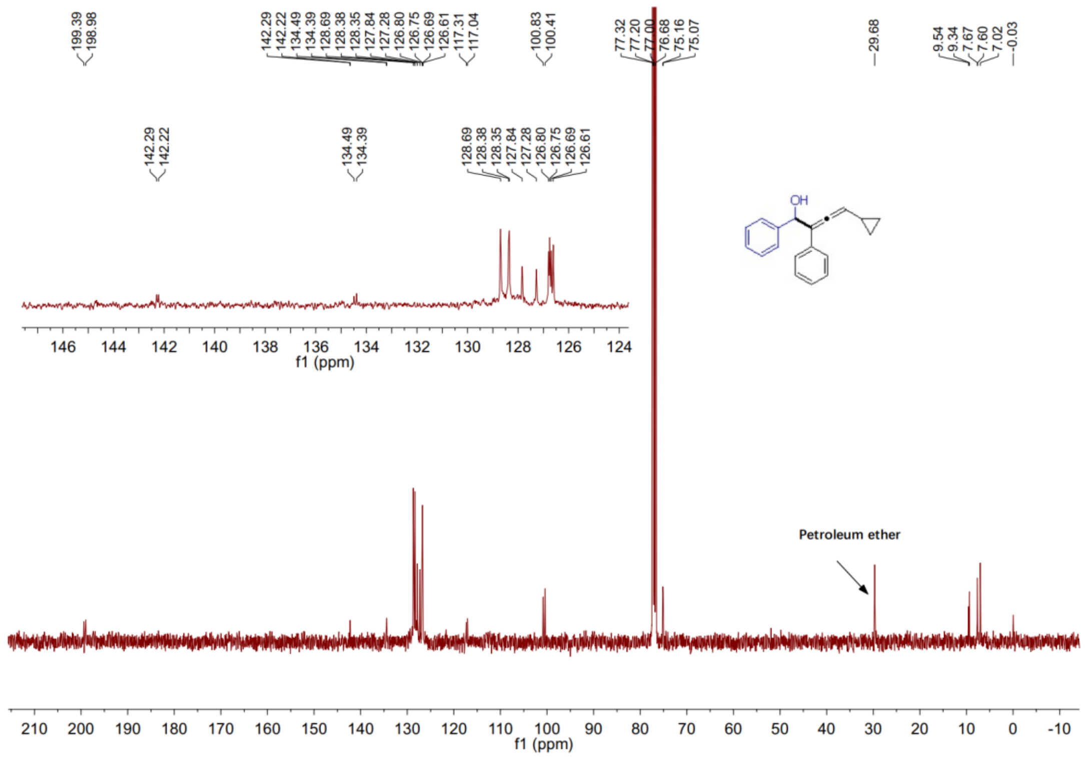

7 ^{1}H NMR

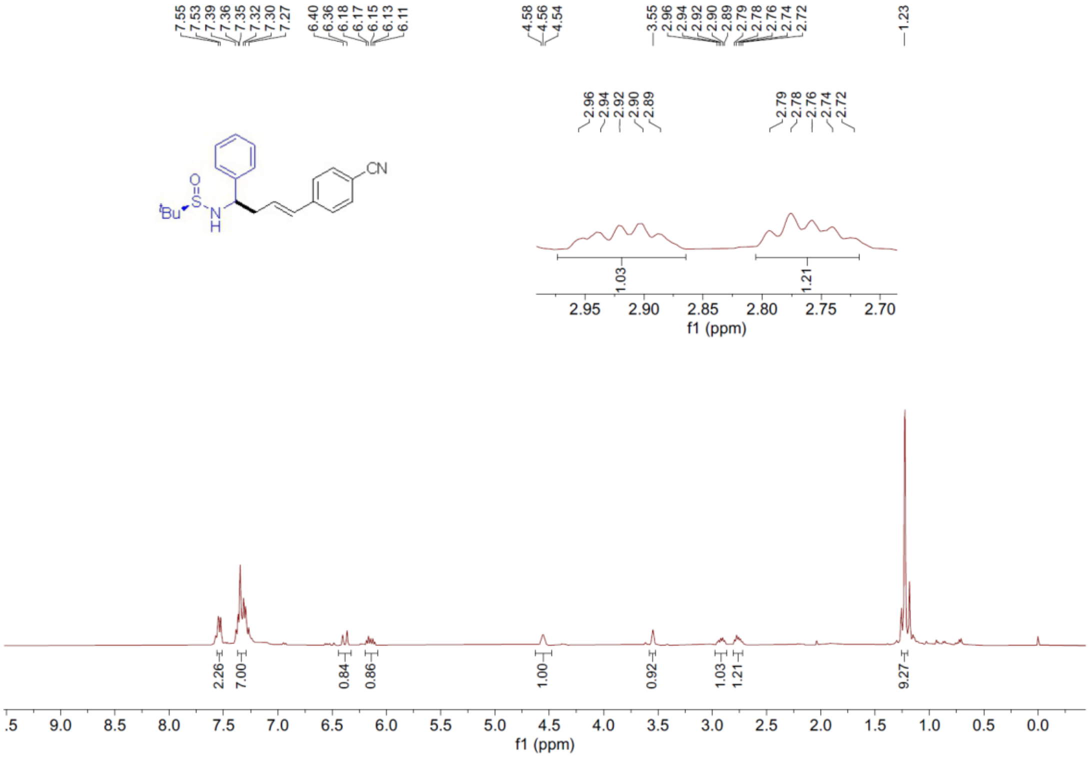

7 dr

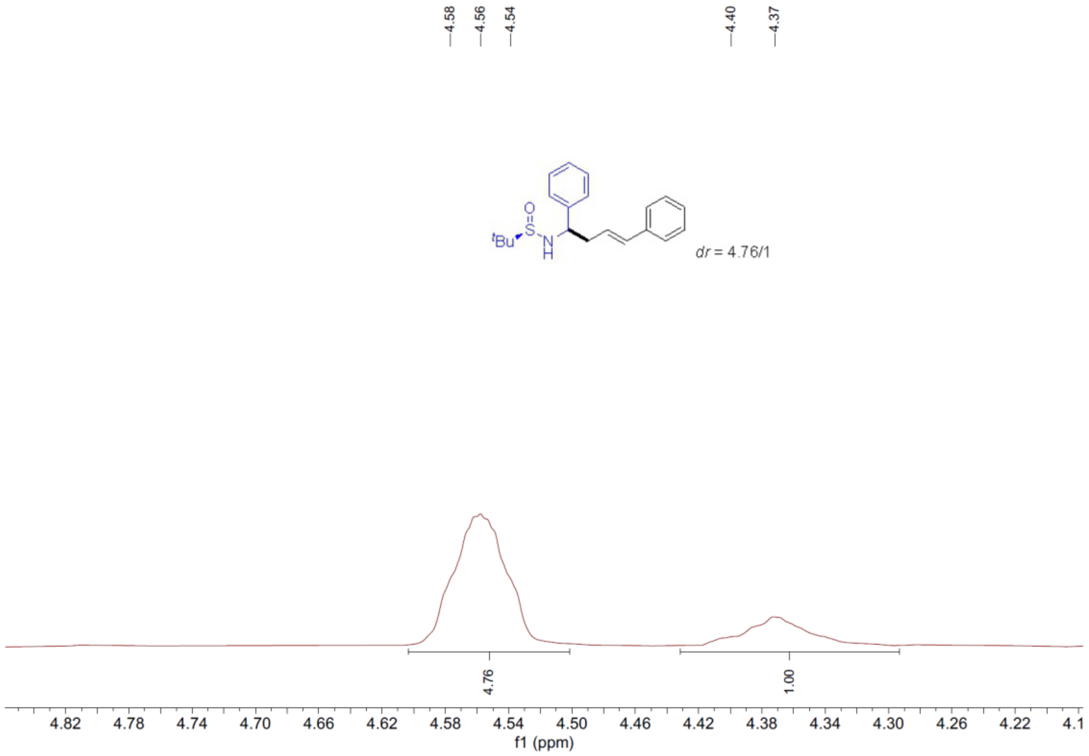

7 ^{13}C NMR

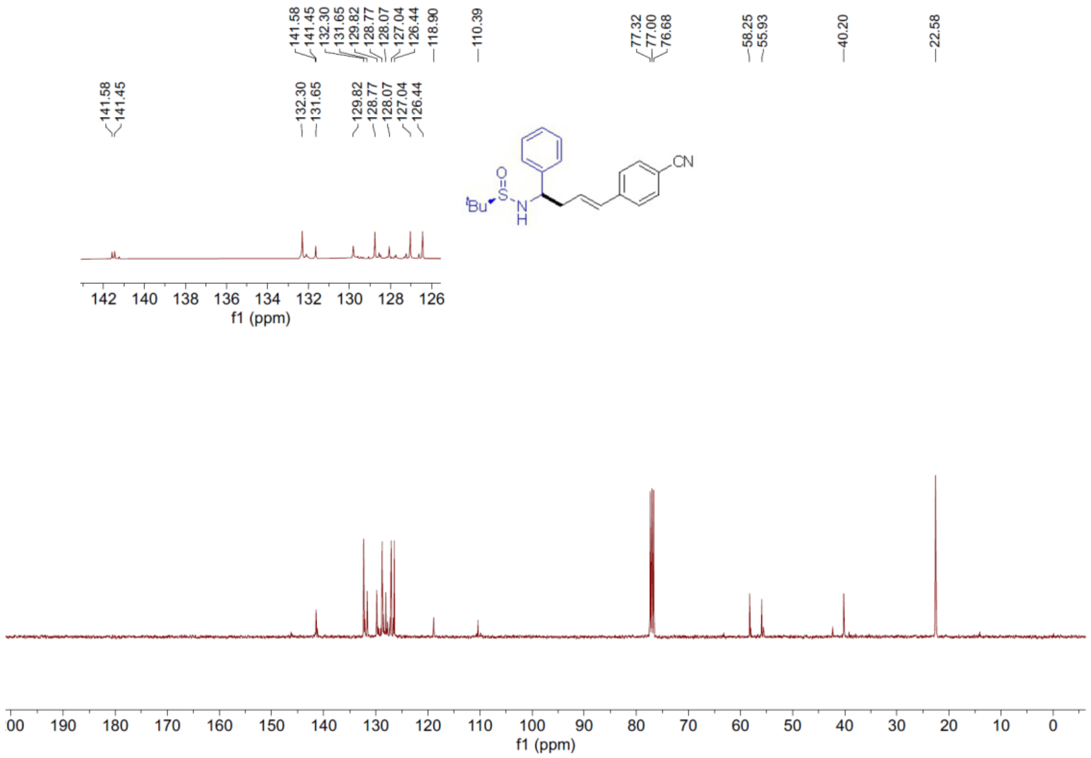

9 ^{1}H NMR

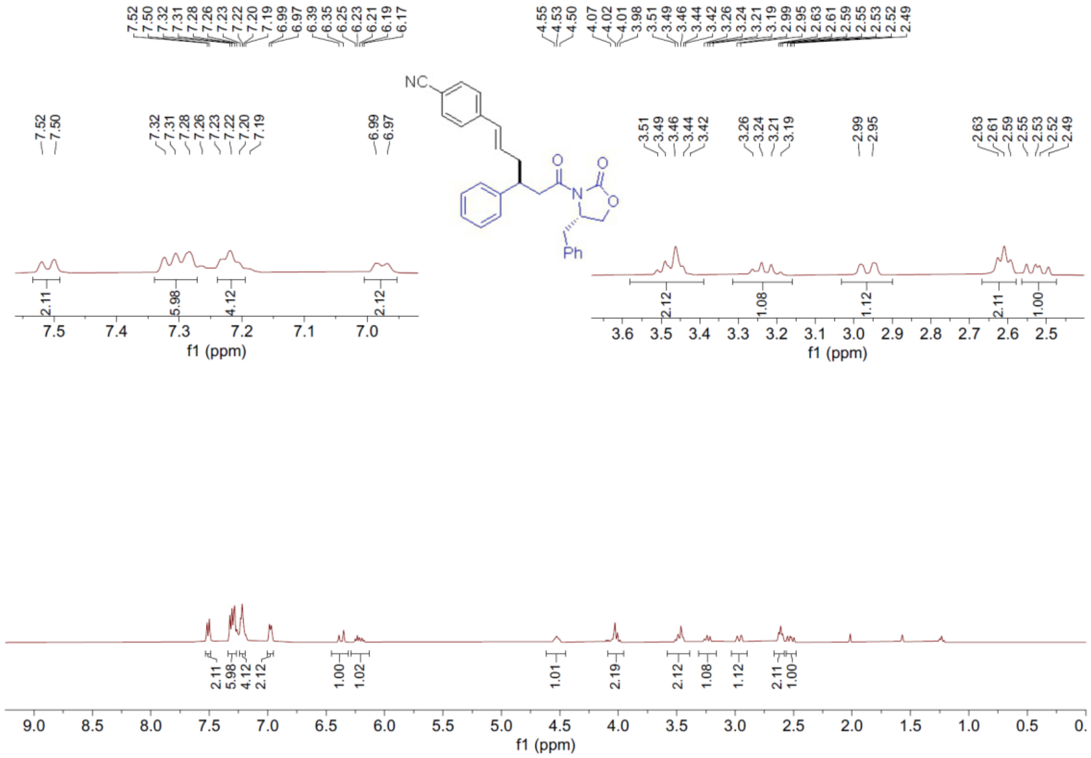

9 ^{13}C NMR

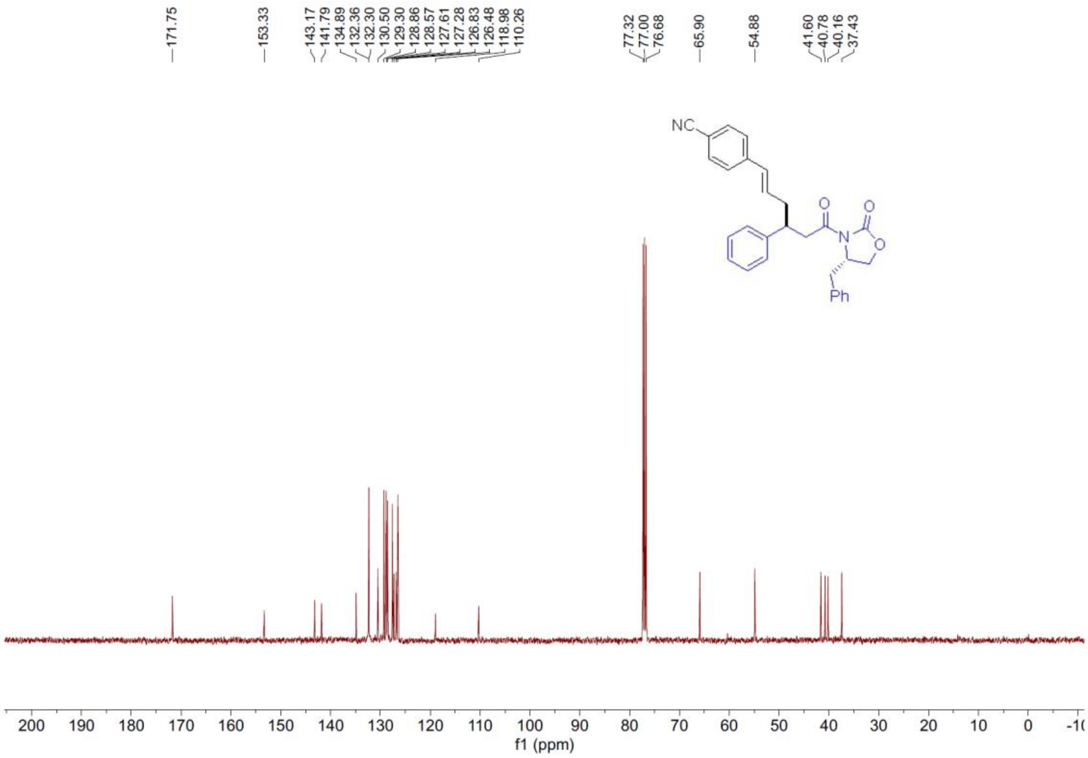

10 ^{1}H NMR

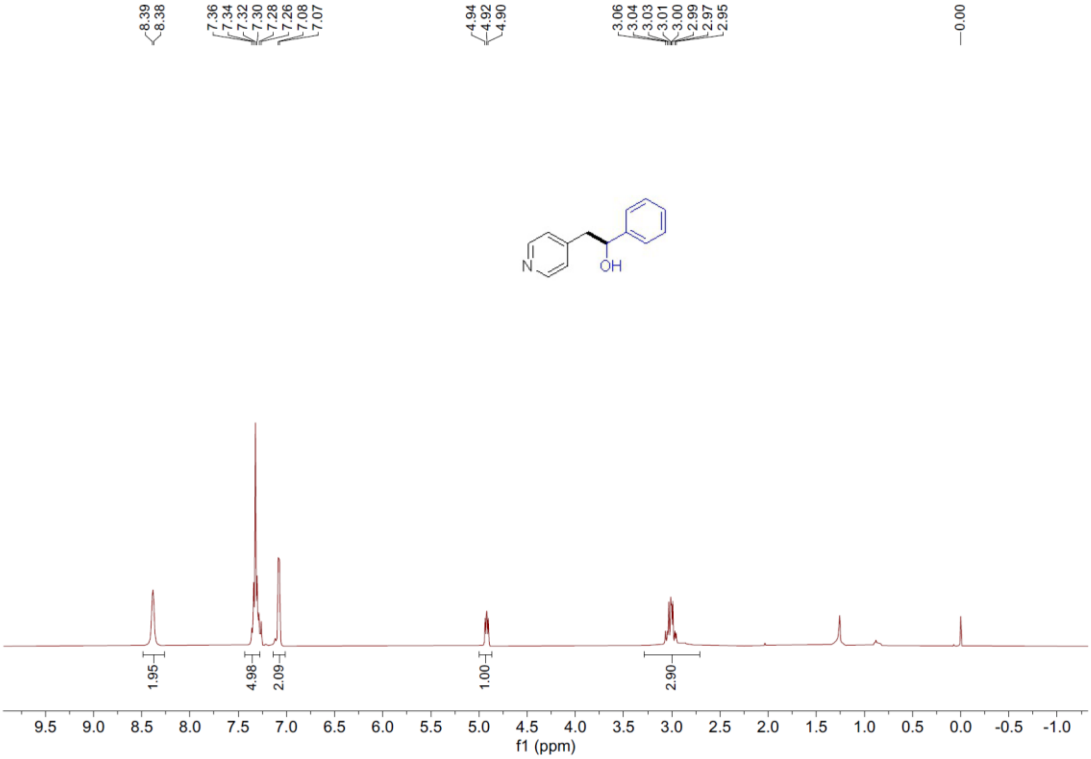

10 ^{13}C NMR

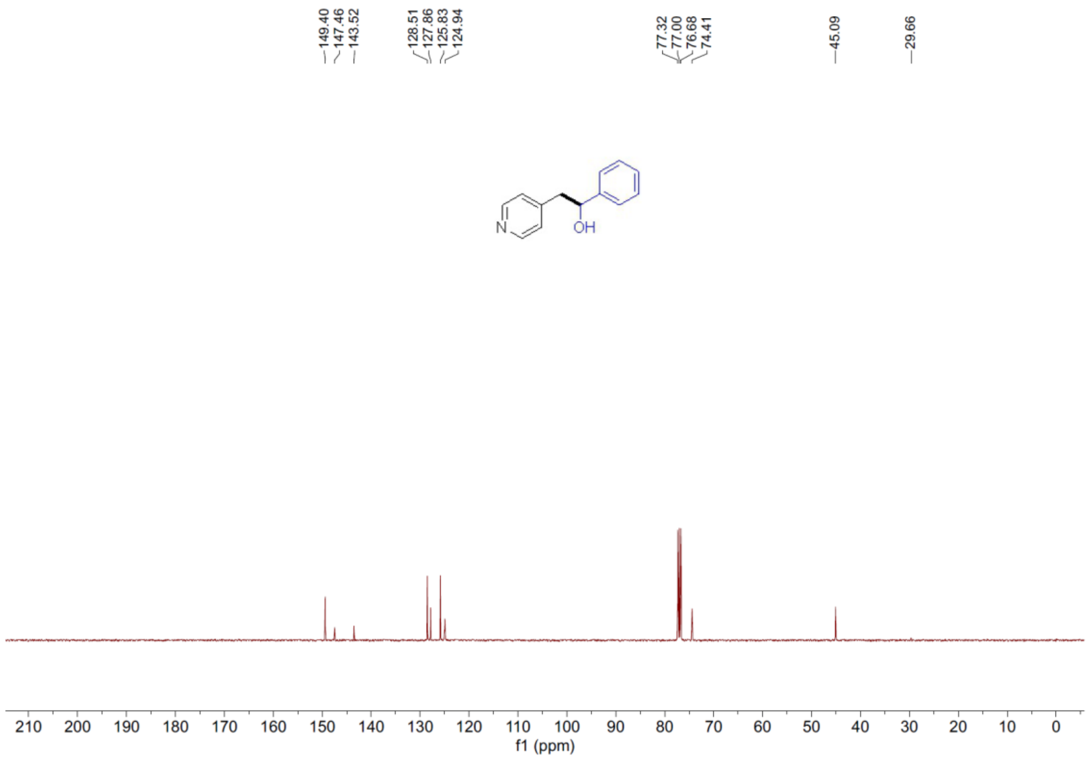

11 ^{1}H NMR

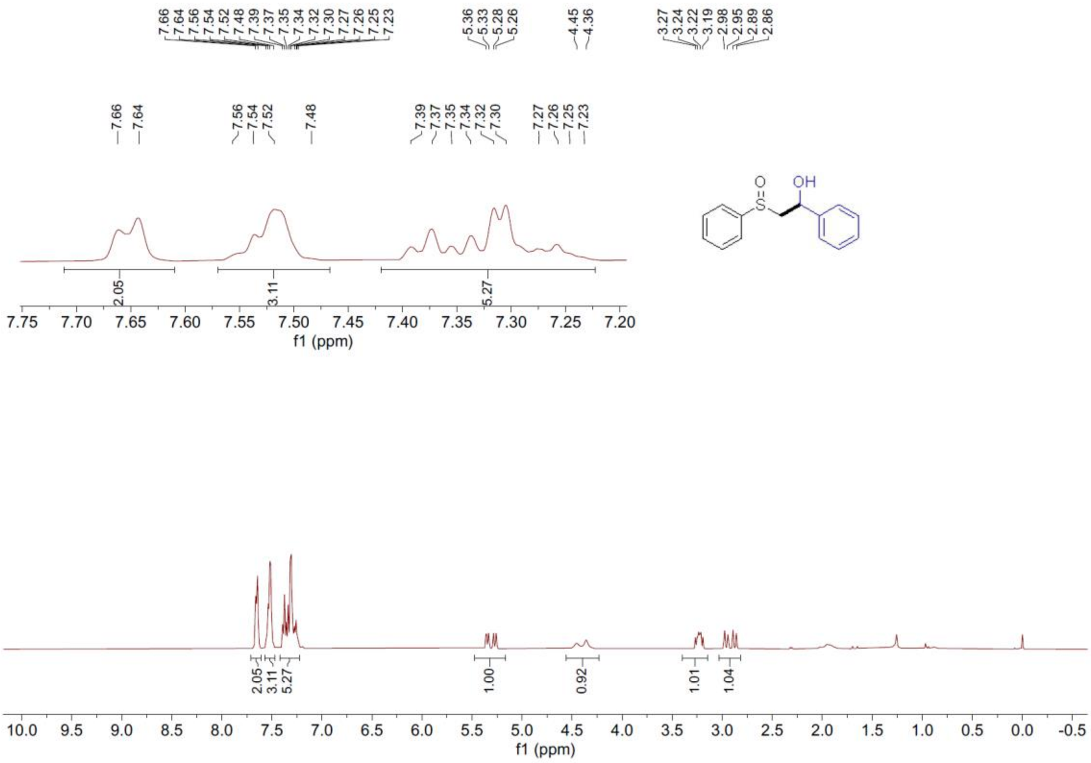

11 *dr*

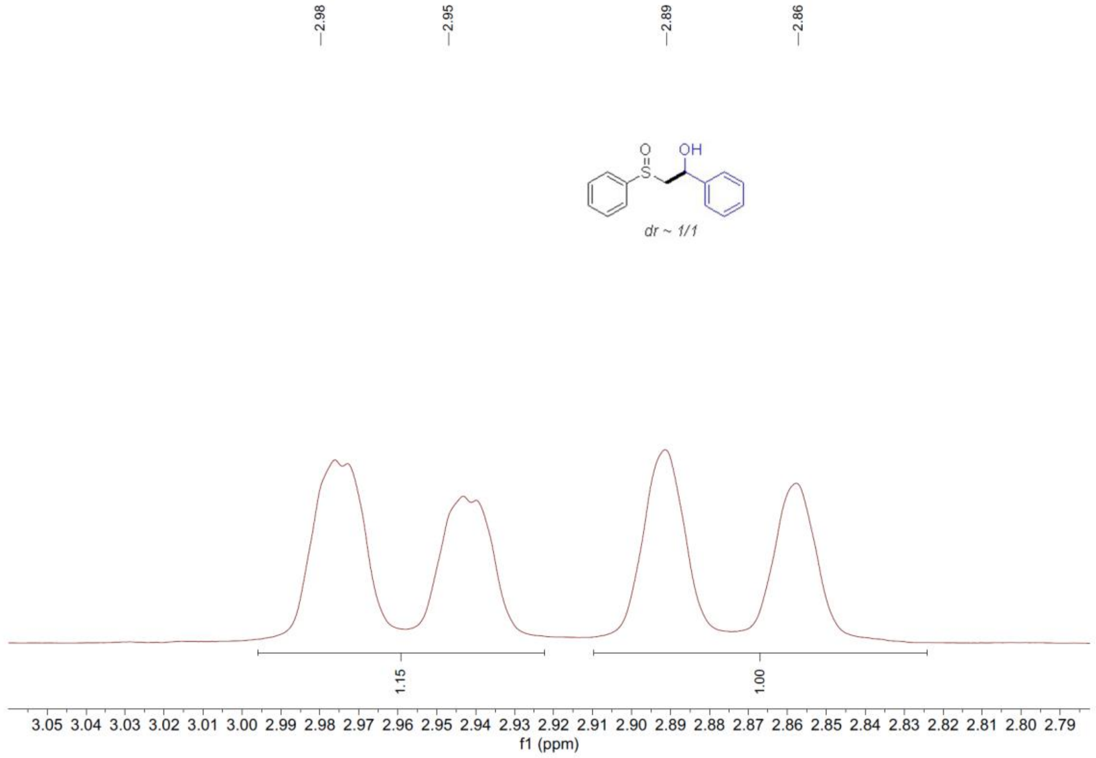

11 ^{13}C NMR

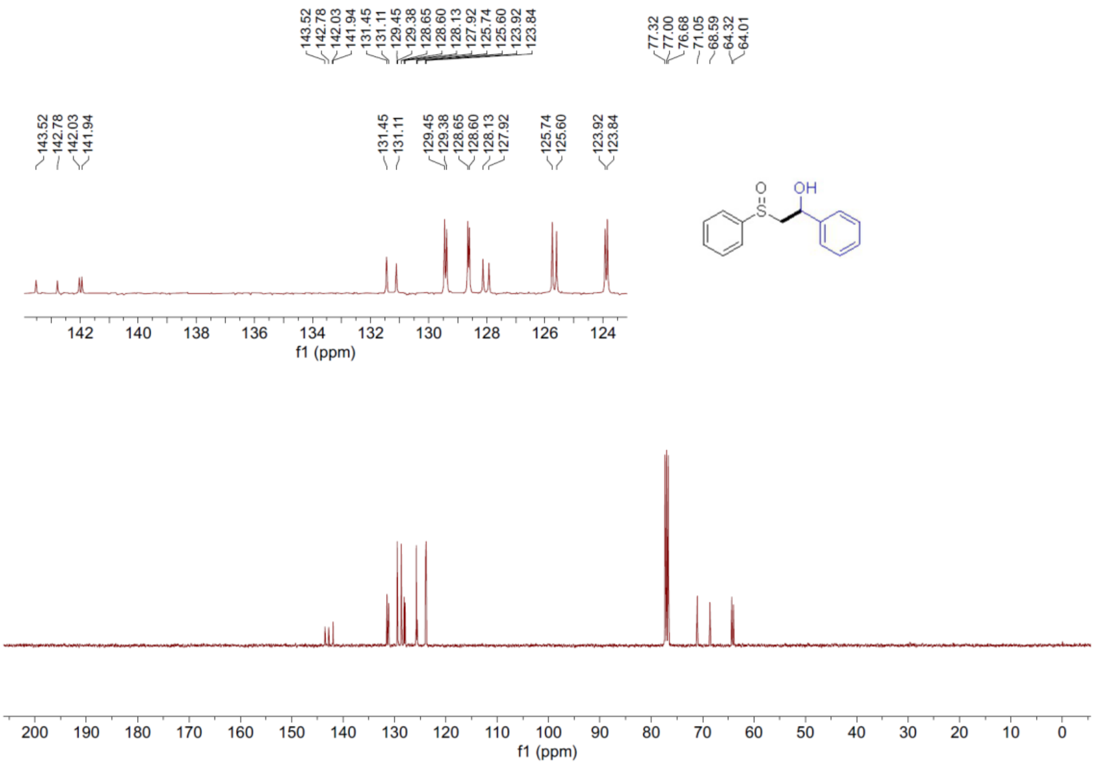

12 ^{1}H NMR

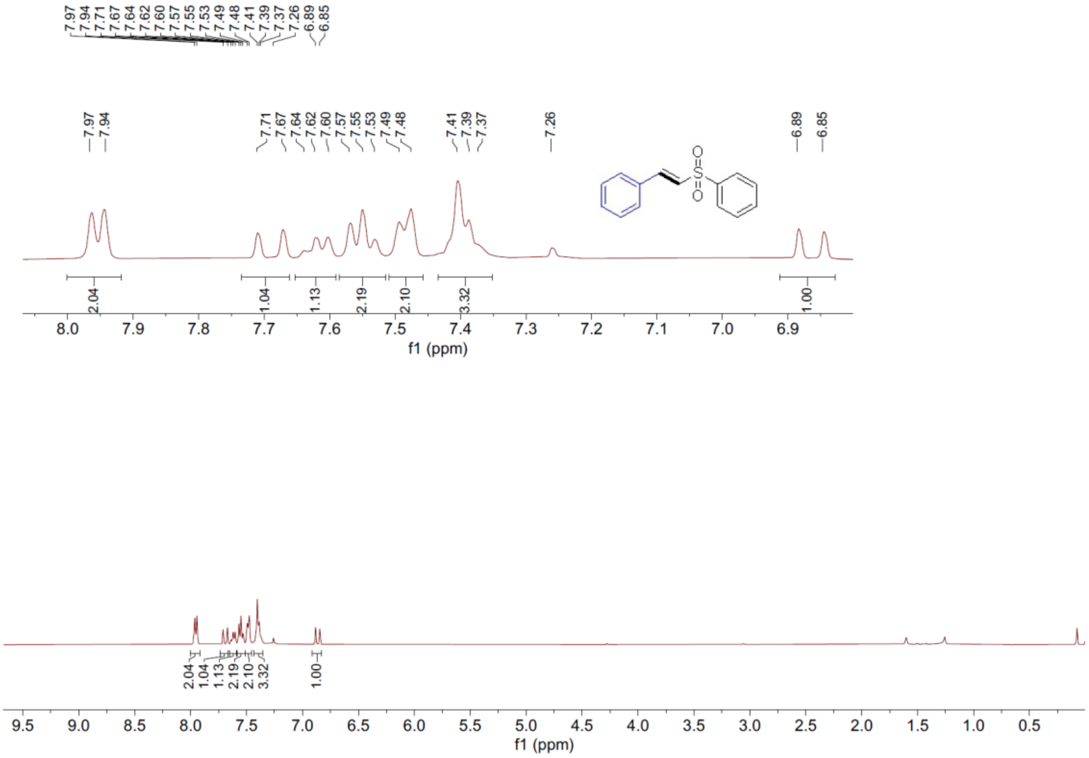

12^{13}C NMR

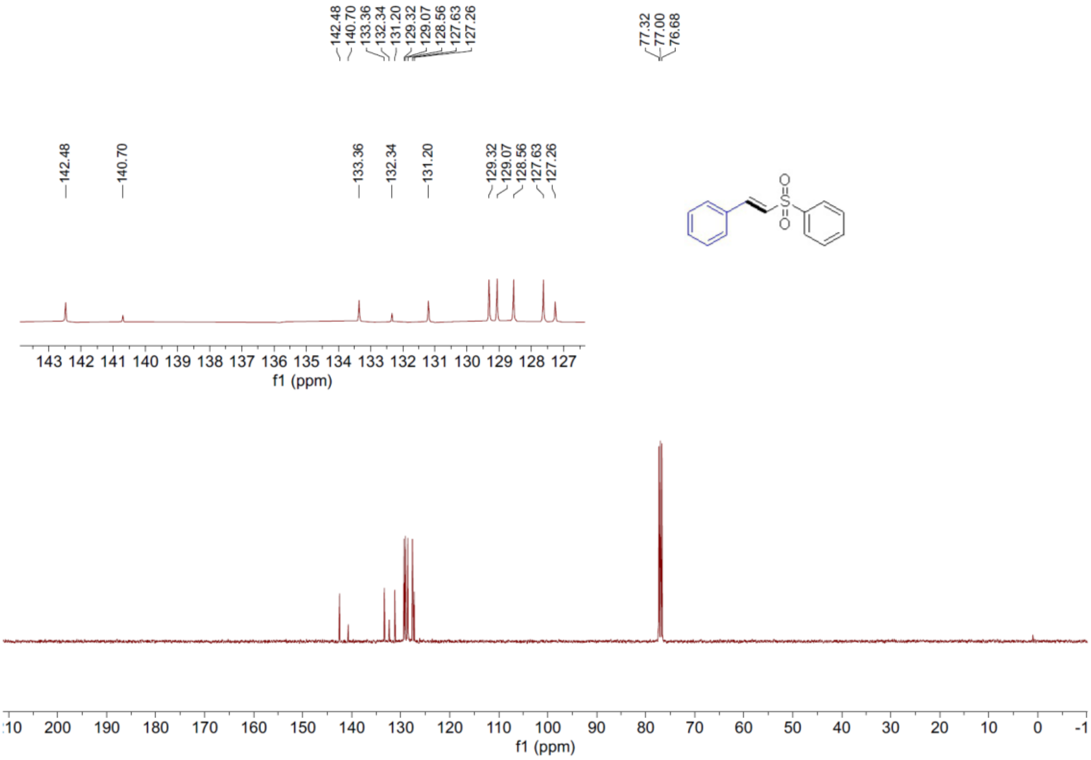

13 ^{1}H NMR

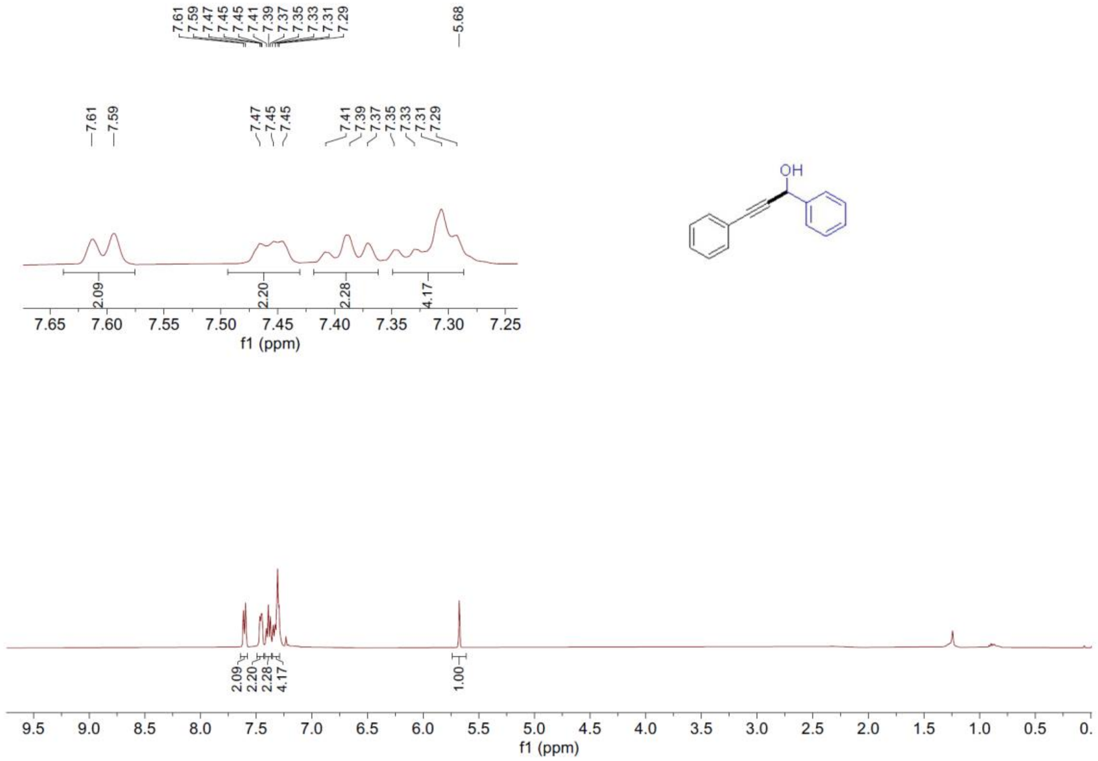

13^{13}C NMR

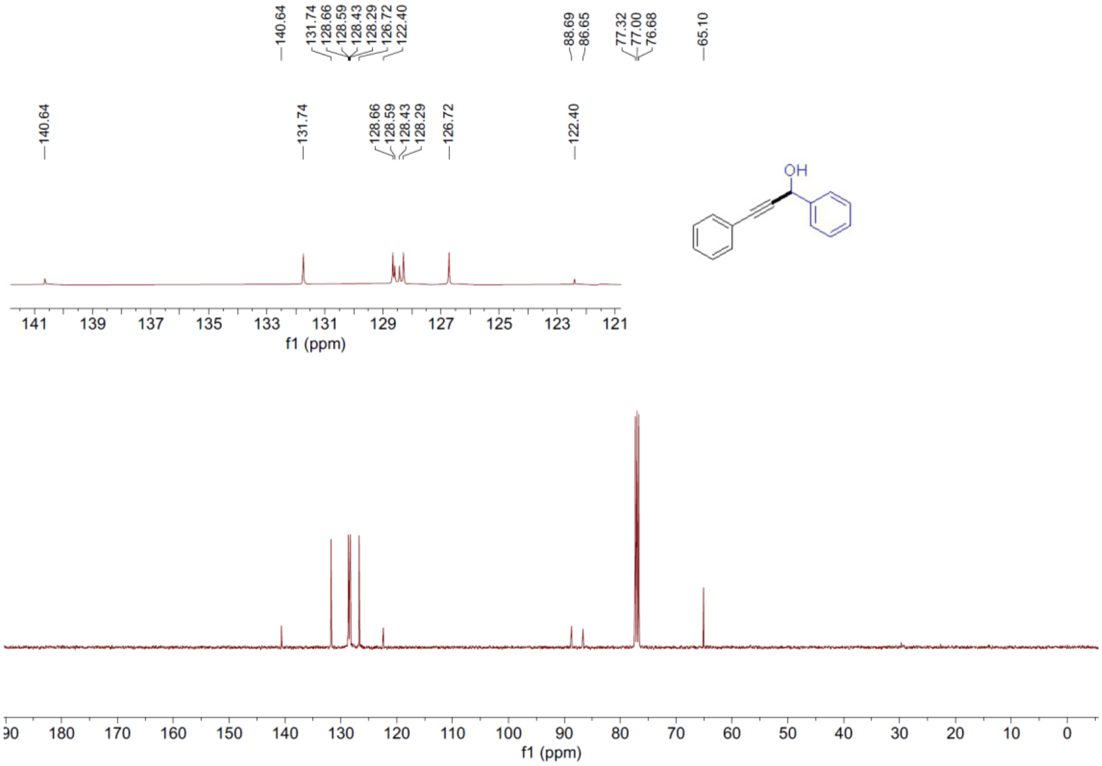

14a ^{1}H NMR

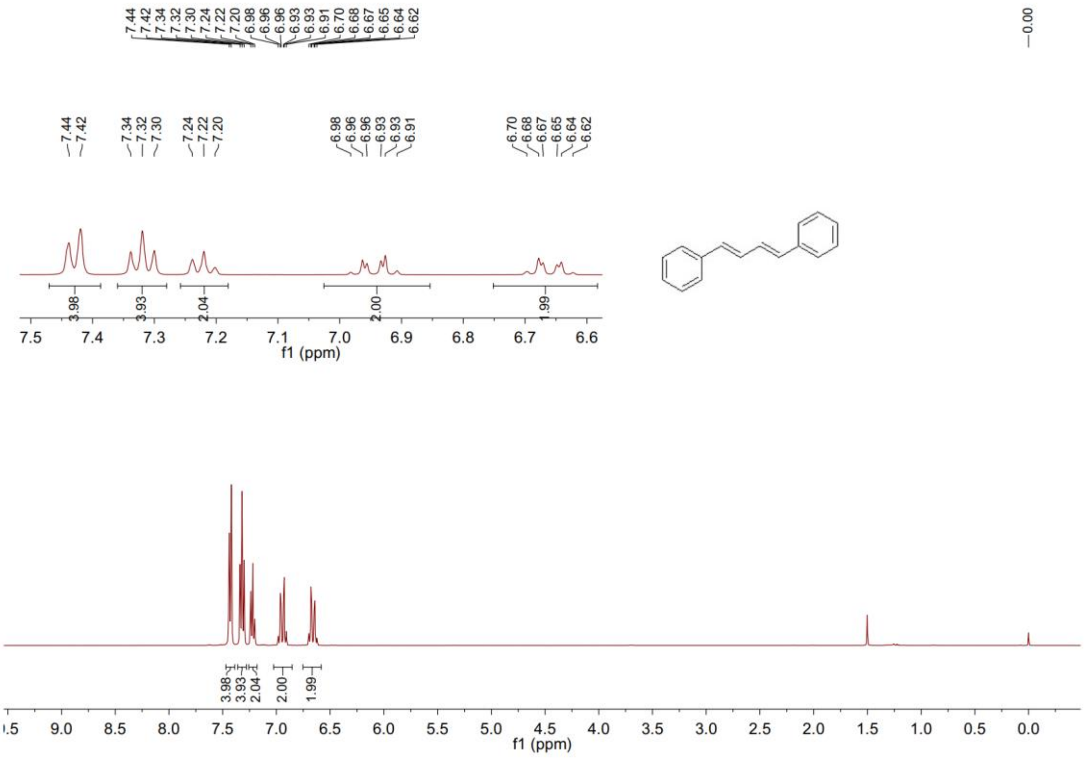

14a ^{13}C NMR

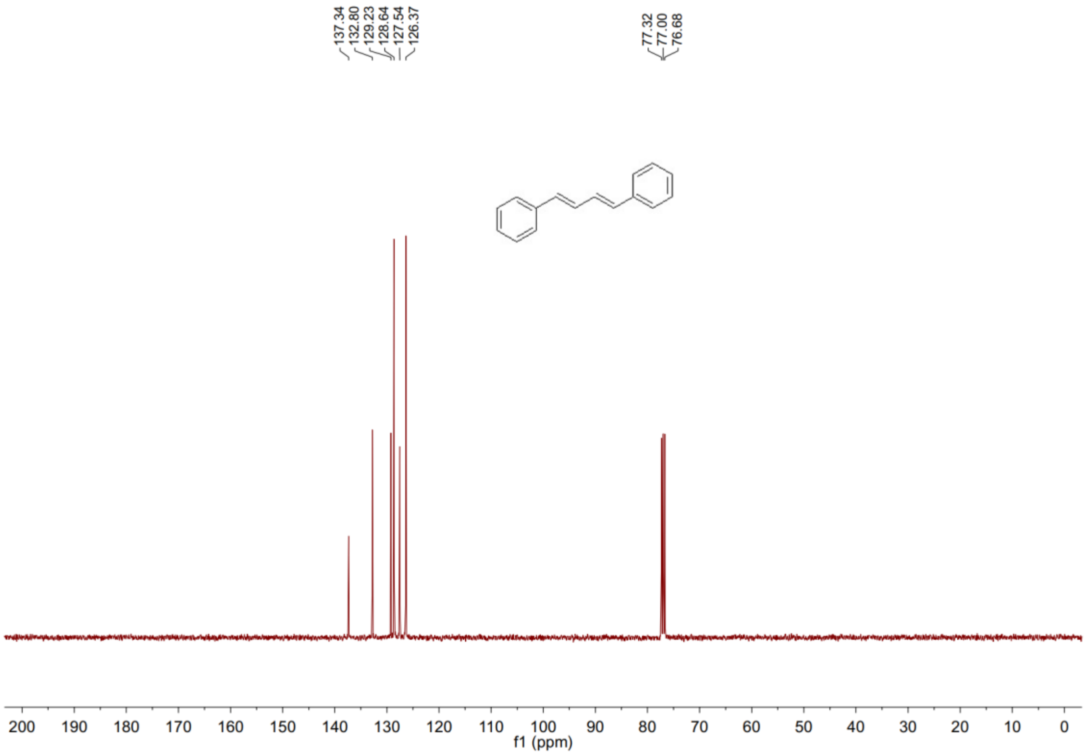

14b ¹H NMR

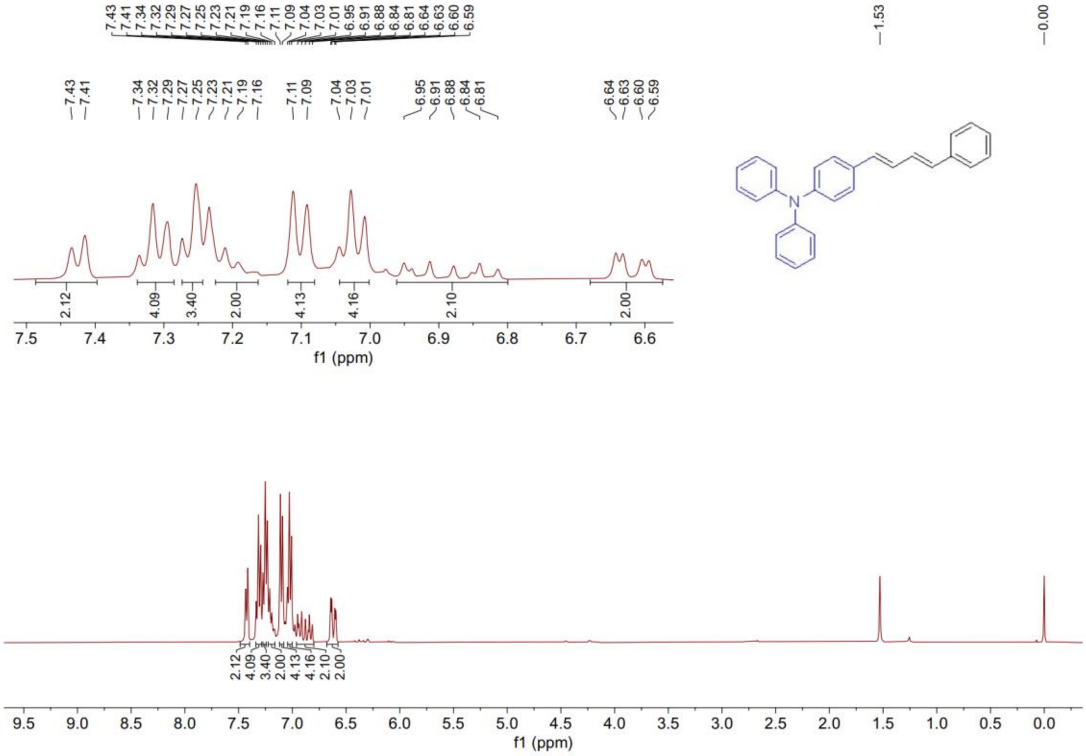

14b ¹³C NMR

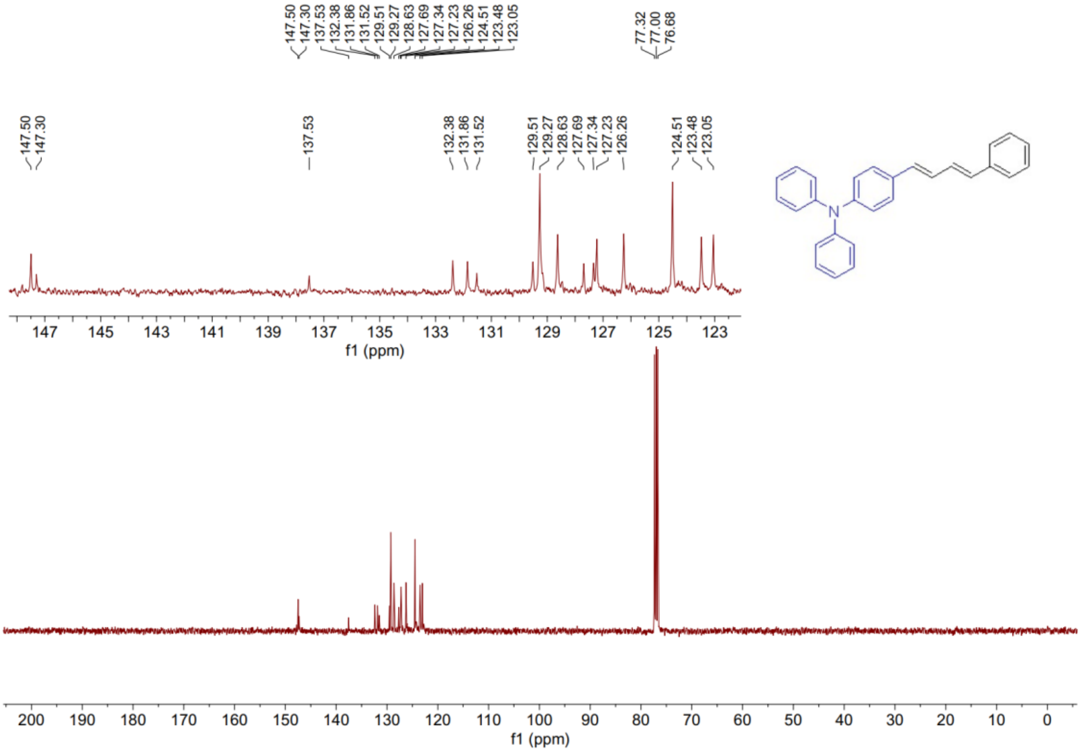

15a ^{1}H NMR

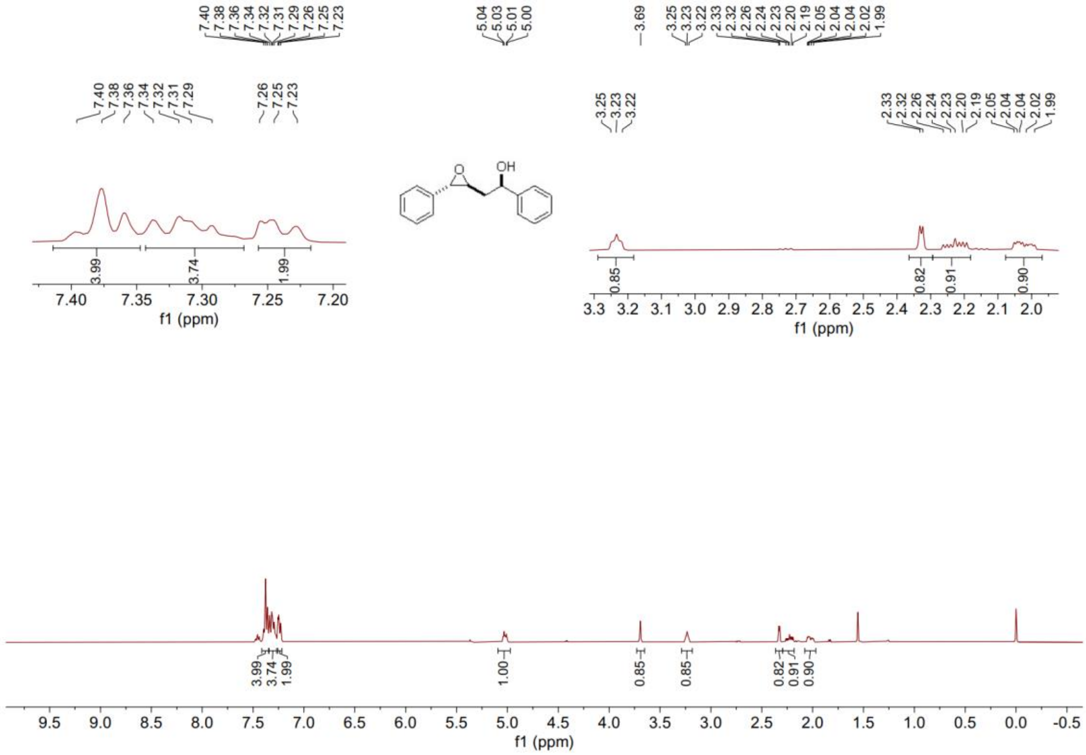

15a ^{13}C NMR

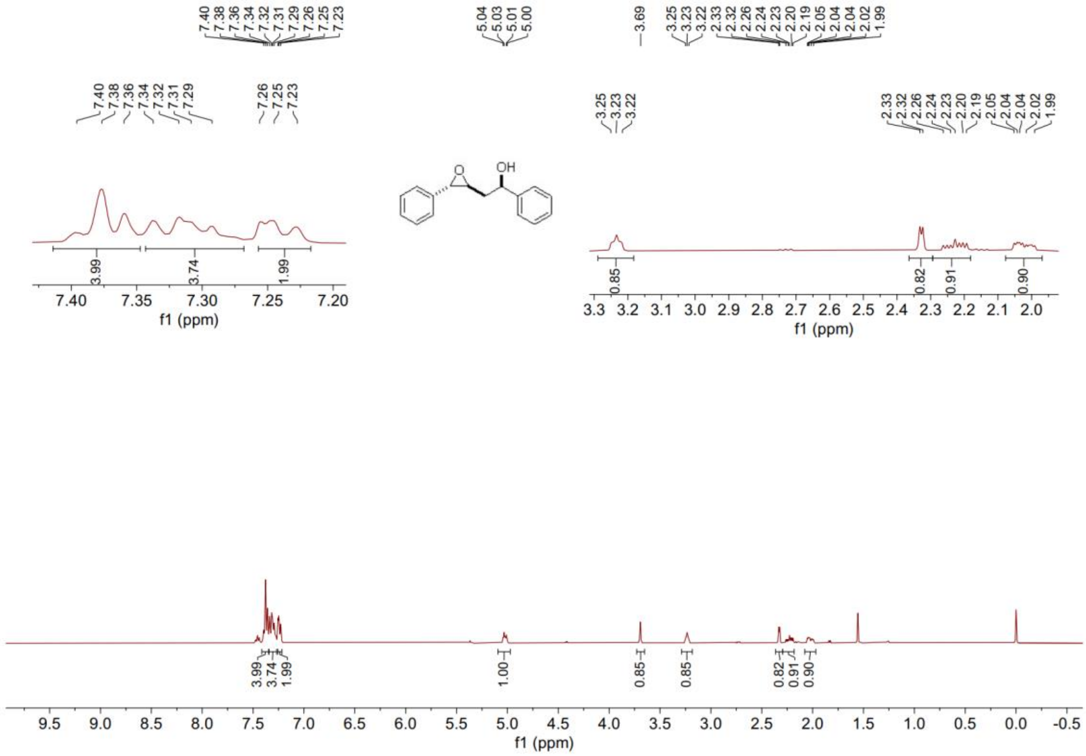

15b ^{1}H NMR

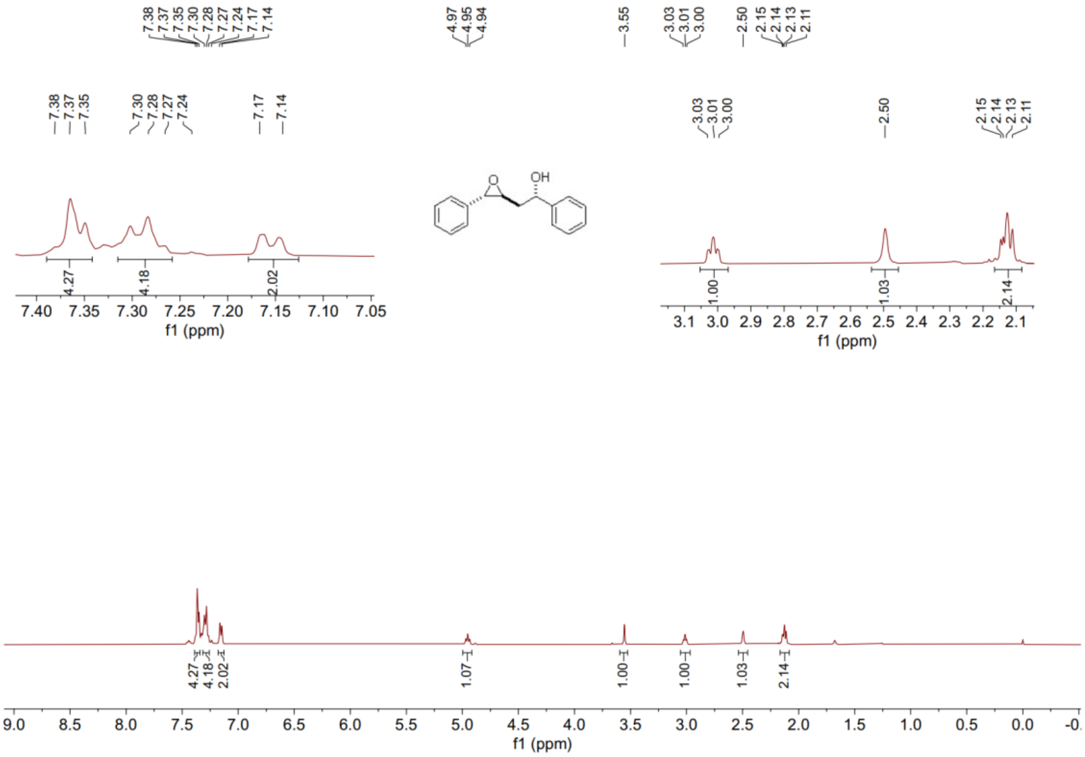

15b ^{13}C NMR

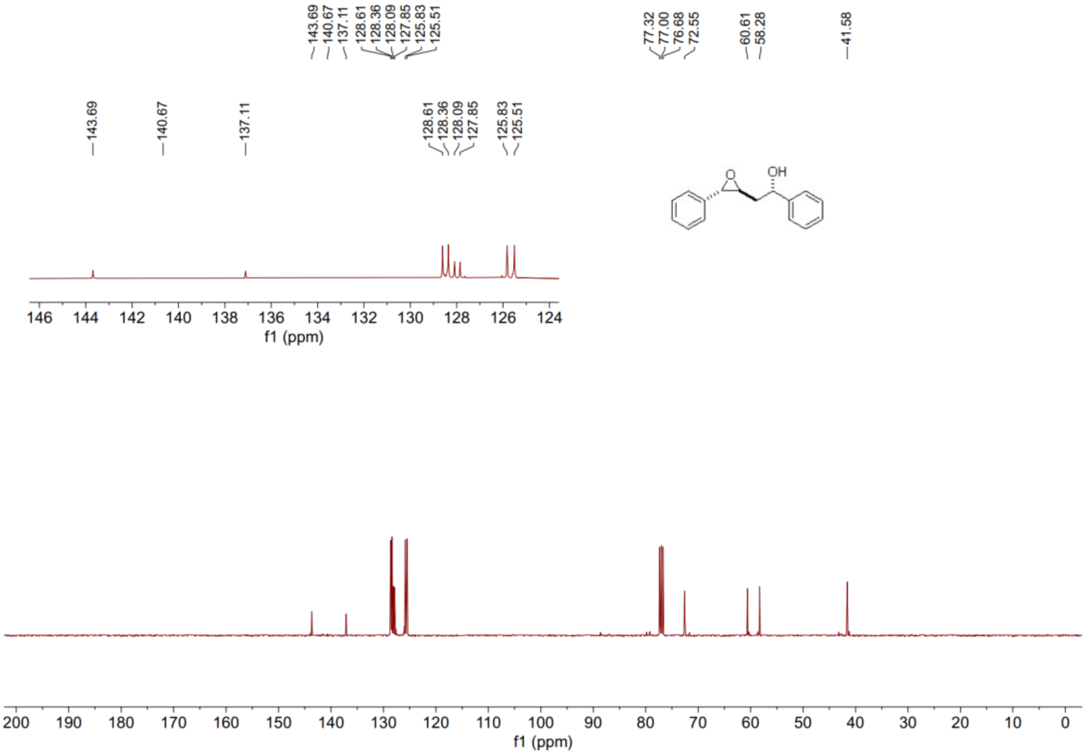

16 ^{1}H NMR

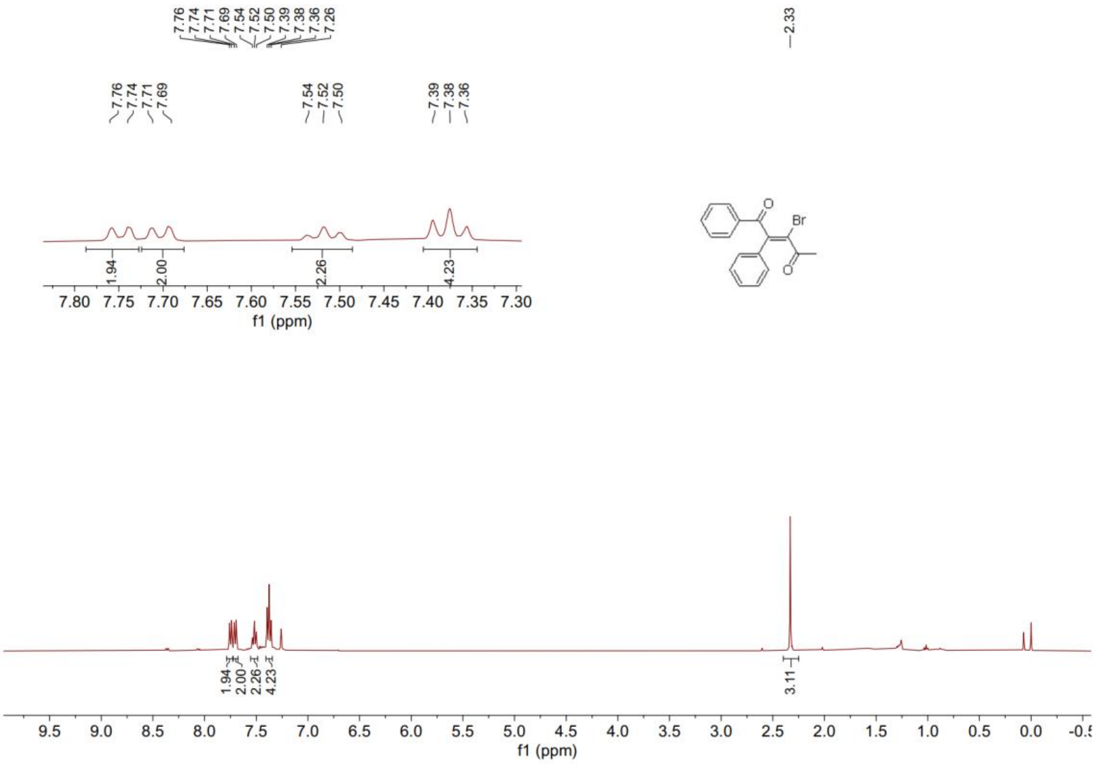

16 ^{13}C NMR

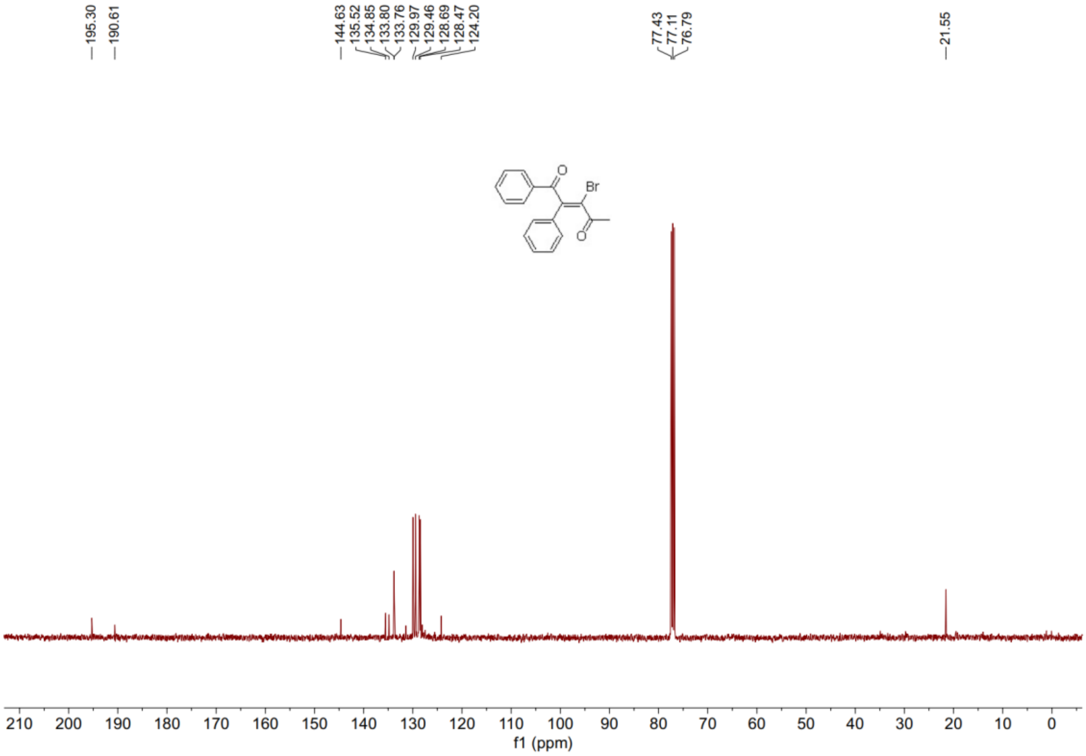

17 ^{1}H NMR

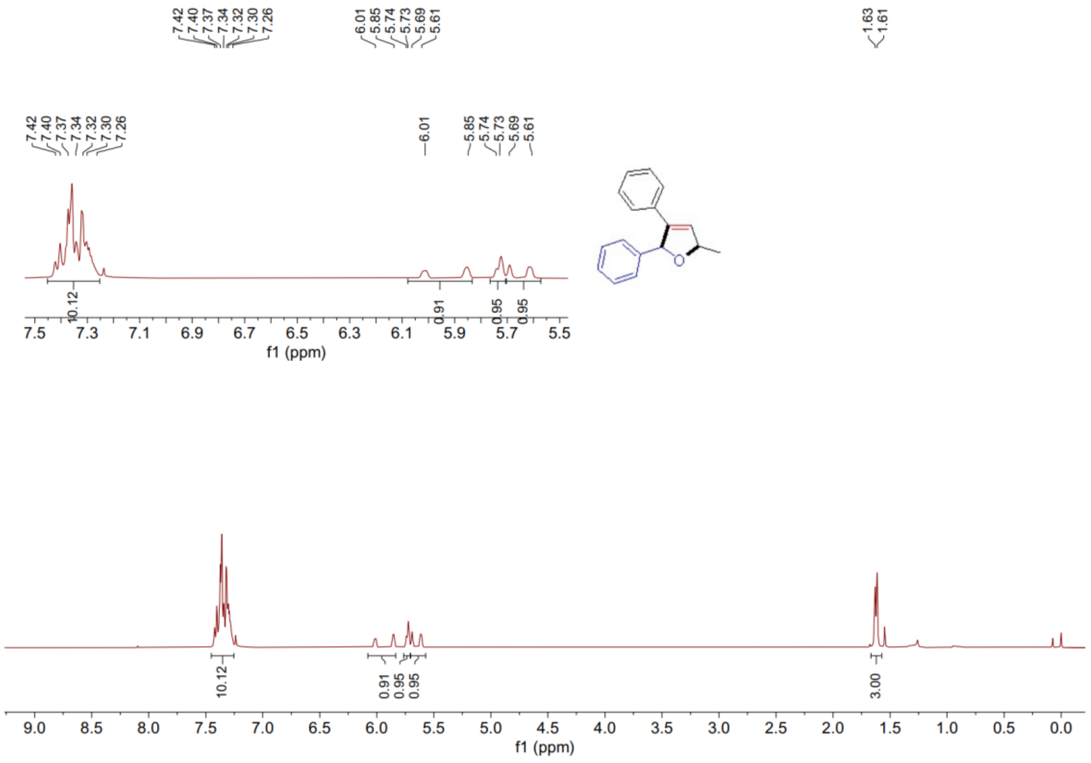

17 ^{13}C NMR

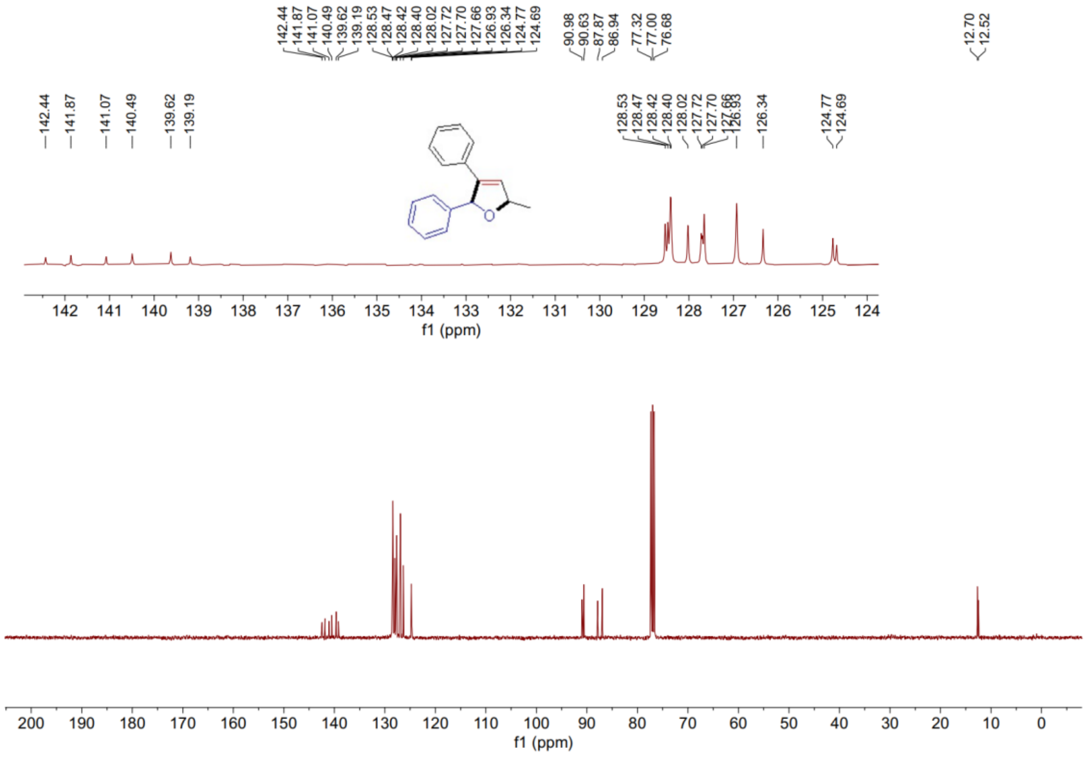

Supplement: Supplementary file 2 — ja3c04864_si_002.pdf [file ja3c04864_si_002.pdf]
